# Stereoselective intermolecular radical cascade reactions of tryptophans or ɤ-alkenyl-α-amino acids with acrylamides via photoredox catalysis

**DOI:** 10.1038/s41467-022-29464-5

**Published:** 2022-04-01

**Authors:** Jiang-Tao Li, Jian-Nan Luo, Jia-Le Wang, De-Ku Wang, Yi-Zhe Yu, Chun-Xiang Zhuo

**Affiliations:** State Key Laboratory of Physical Chemistry of Solid Surfaces, Key Laboratory of Chemical Biology of Fujian Province, College of Chemistry and Chemical Engineering, Xiamen University, Xiamen, 361005 People’s Republic of China

**Keywords:** Synthetic chemistry methodology, Homogeneous catalysis, Photocatalysis

## Abstract

The radical cascade reaction is considered as one of the most powerful methods to build molecular complexity. However, highly stereoselective intermolecular radical cascade reactions that can produce complex cyclic compounds bearing multiple stereocenters via visible-light-induced photocatalysis have been challenging yet desirable. Herein we report a facile and efficient synthesis of multi-substituted *trans*-fused hexahydrocarbazoles via a stereoselective intermolecular radical cascade reaction of readily available tryptophans and acrylamides enabled by visible-light-induced photoredox catalysis. The *trans*-fused hexahydrocarbazoles with up to five stereocenters including two quaternary ones can be accessed in up to 82% yield, >20/1 diastereoselectivity, and 96% ee. Interestingly, the tetrahydrocarbazoles are favorably formed when the reaction is performed under air. Moreover, by simply switching the starting material from tryptophans to ɤ-alkenyl substituted α-amino acids, this protocol can be further applied to the stereoselective syntheses of 1,3,5-trisubstituted cyclohexanes which are otherwise challenging to access. Preliminary mechanistic studies suggest that the reaction goes through radical addition cascade and radical-polar crossover processes.

## Abstract (web summary)

Photocatalytic radical cascade reactions enable the facile construction of diverse cyclic compounds, though they rely on templated precursors. In this paper, the authors report on stereoselective intermolecular radical cascade reaction between tryptophan or ɤ-alkenyl substituted amino acids and acrylamides to synthesise multi-substituted trans-fused hexahydrocarbazoles or 1,3,5-trisubstituted cyclohexanes.

## Introduction

In the pharmaceutical industry, the growing demand for complex three-dimensional cyclic compounds urges the synthetic chemists to develop novel and efficient strategies to construct these motifs^1,2^. Radical cascade reactions are considered as one of the most powerful methods to meet this demand^3–6^. This protocol, which allows the rapid syntheses of valuable complex cyclic molecules, has been widely applied in the total syntheses of natural products^7–9^. However, many radical transformations involving alkyl radical species usually requires the use of organic halides as substrates, the addition of toxic stannanes as the halogen or hydrogen atom transfer agents, and the use of unstable initiators^10,11^. In recent years, photocatalysis using visible light has emerged as a promising strategy for the development of novel radical reactions due to its high efficiency and convenience in the generation of various reactive radical species under mild and environmentally friendly conditions^12–19^. Specifically, the visible-light-induced radical cascade reactions enable the facile construction of diverse cyclic compounds under sustainable conditions^3,20,21^. Despite these advances, plenty of radical cascade cyclization reactions still rely on either the carefully designed precursors or the intramolecular design, which tempers the appeal of their further synthetic applications. Therefore, it is highly desirable to develop stereoselective intermolecular radical cascade reactions that could produce complex cyclic compounds from readily available starting materials via visible-light-induced photocatalysis.

**Fig. 1:**
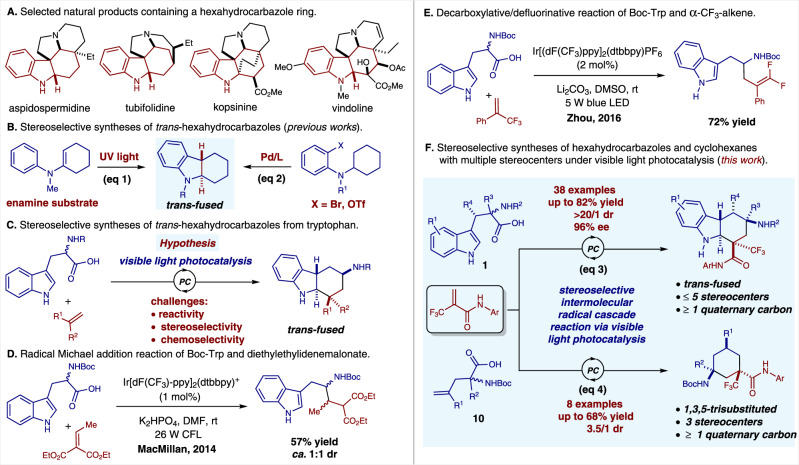
Syntheses of *trans*-fused hexahydrocarbazoles and 1,3,5-trisubstituted cyclohexanes through stereoselective intermolecular radical cascade reaction under visible light photocatalysis. **A** Natural products containing a hexahydrocarbazole ring. **B** Previous works. **C** Hypothesis. **D** Reaction of Boc-Trp and diethylethylidenemalonate. **E** Reaction of Boc-Trp and ɑ-CF_3_-alkene. **F** This work. *PC* photocatalyst, *Trp* tryptophan.

As a pivotal structure unit, hexahydrocarbazole motif widely occurs in many biologically active polycyclic indoline natural products^22–26^, such as kopsinine, tubifolidine, aspidospermidine, and vindoline (Fig. 1A). Consequently, great efforts have been devoted to the preparation of functionalized hexahydrocarbazoles^27–43^. Among those, direct catalytic dearomatization reaction of indole derivatives represents a straightforward and powerful strategy^44–50^. The *cis-*fused hexahydrocarbazoles are usually obtained through the dearomatization of substituted or pre-functionalized indole derivatives^27–33^. In contrast, the syntheses of the *trans-*fused hexahydrocarbazoles are less explored^36–43^. To date, methods to uncover the highly stereoselective synthesis of this structural motif have been limited to UV-light-induced intramolecular photocyclization of reactive enamine precursors^36,37^ (eq 1, Fig. 1B) as well as Pd-catalyzed intramolecular C–H activation/cyclization reaction (eq 2, Fig. 1B) ^38–43^.

Tryptophan is a commercially available, non-toxic, abundant, and naturally occurring α-amino acid. Accordingly, we questioned whether this readily available material could be directly used to synthesize the *trans*-fused hexahydrocarbazole through a one-step visible-light-induced photocatalytic radical cascade reaction (Fig. 1C). In practice, tryptophan was often used as an α-amino alkyl radical precursor in radical addition reactions via visible-light-induced photocatalysis^51–58^. In pioneer work, MacMillan and coworkers reported an elegant radical Michael addition reaction of *N*-Boc-tryptophan and diethylethylidenemalonate via photocatalysis (Fig. 1D)^54^. In 2016, Zhou and coworkers described an interesting photocatalytic decarboxylative/defluorinative reaction of *N*-Boc-tryptophan and α-trifluoromethyl alkenes (Fig. 1E)^56^. Nevertheless, to the best of our knowledge, there is no general method for the generation of *trans*-fused hexahydrocarbazoles bearing multiple stereocenters through a one-step stereoselective radical cascade reaction of tryptophan via visible-light-induced photocatalysis. Herein, we report a facile and efficient synthesis of multi-substituted *trans*-fused hexahydrocarbazoles via a stereoselective intermolecular radical cascade reaction of readily available tryptophans and acrylamides enabled by visible-light-induced photoredox catalysis (eq 3, Fig. 1F). The *trans*-fused hexahydrocarbazoles with up to five stereocenters including two quaternary ones could be accessed in up to 82% yield, >20/1 diastereoselectivity, and 96% ee. Intriguingly, this protocol could further be applied to the stereoselective syntheses of 1,3,5-trisubstituted cyclohexanes, which are otherwise challenging to access in a single operation, by simply switching the starting material from tryptophans **1** to ɤ-alkenyl substituted amino acids **10** (eq 4, Fig. 1F).

## Results

### Reaction optimization

Our studies were initiated with an exploration of reaction conditions for the intermolecular coupling of commercially available *N*-(*tert*-butoxycarbonyl)tryptophan (**1a**) and *N*-phenyl-2-(trifluoromethyl)acrylamide (**2a**) (Table 1). After an extensive investigation of reaction conditions, it was found that the desired radical cascade cyclization product *trans*-fused hexahydro-1*H*-carbazole **3a** was obtained in 75% yield and >20:1 dr when the reaction was performed with 1 mol% of Ir[dF(CF_3_)ppy]_2_(dtbbpy)PF_6_ as the photocatalyst, 3 equiv of Na_2_CO_3_ as the base and blue LEDs as the light source (entry 1, for the experimental setup, see Supplementary Fig. 1). Notably, the probably competitive radical addition product **3–1**, radical addition/β-fluoride elimination product **3–2**, and nucleophilic addition/cyclization product **3–3** were not detected under these photocatalytic conditions, which demonstrated the unique selectivity of the current reaction. The reaction could also proceed when the organic dye 4-CzIPN was used as the photocatalyst (entry 2). However, the use of a ruthenium-based photocatalyst led to low yield of the desired product (entry 3). The reaction also proceeded smoothly when different inorganic bases such as NaHCO_3_ and K_2_CO_3_ were used (entries 4–5). When the reaction was performed in the absence of base, the desired product was obtained with a significantly lower yield (entry 6). Control experiments demonstrated that the photocatalyst and light were indispensable for the product formation (entry 7). Interestingly, when the reaction was performed under air, 4% of the desired product was observed along with 61% of the tetrahydro-1*H*-carbazole **9a** (entry 8, yields were determined by crude ^1^H NMR analysis. For details, see Fig. 4).

**Table 1:** Optimization of the reaction conditions^a^.


entry	variation from the standard conditions	yield^b^ (%)	dr^c^
1	none	75^d^	>20:1
2	4-CzIPN instead of Ir-cat.	40	>20:1
3	Ru(bpy)_3_(PF_6_)_2_ instead of Ir-cat.	8	>20:1
4	NaHCO_3_ instead of Na_2_CO_3_	69	>20:1
5	K_2_CO_3_ instead of Na_2_CO_3_	60	>20:1
6	no base	18	>20:1
7	no Ir-cat., or no light	NR^e^	NA^f^
8	under air	4^g^	>20:1


^a^Reaction conditions: 1 mol% of Ir[dF(CF_3_)ppy]_2_(dtbbpy)PF_6_, 0.3 mmol of Na_2_CO_3_, 0.15 mmol of **1a**, 0.1 mmol of **2a** in *N*,*N*-dimethylacetamide (DMA, 2.0 mL) for 12 h under blue LEDs irradiation at 30 °C.

^b^Yields of the major diastereomer were determined by ^1^H NMR analysis using CH_2_Br_2_ as internal standard.

^c^The diastereomeric ratio (dr) was determined by crude ^1^H NMR analysis.

^d^Isolated yield of the major diastereomer.

^e^NR = no reaction.

^f^NA = not available.

^g^Yield of **9a** (Fig. 4, dr > 20:1) was 61% as determined by ^1^H NMR analysis.

### Evaluation of the substrate scope

**Fig. 2:**
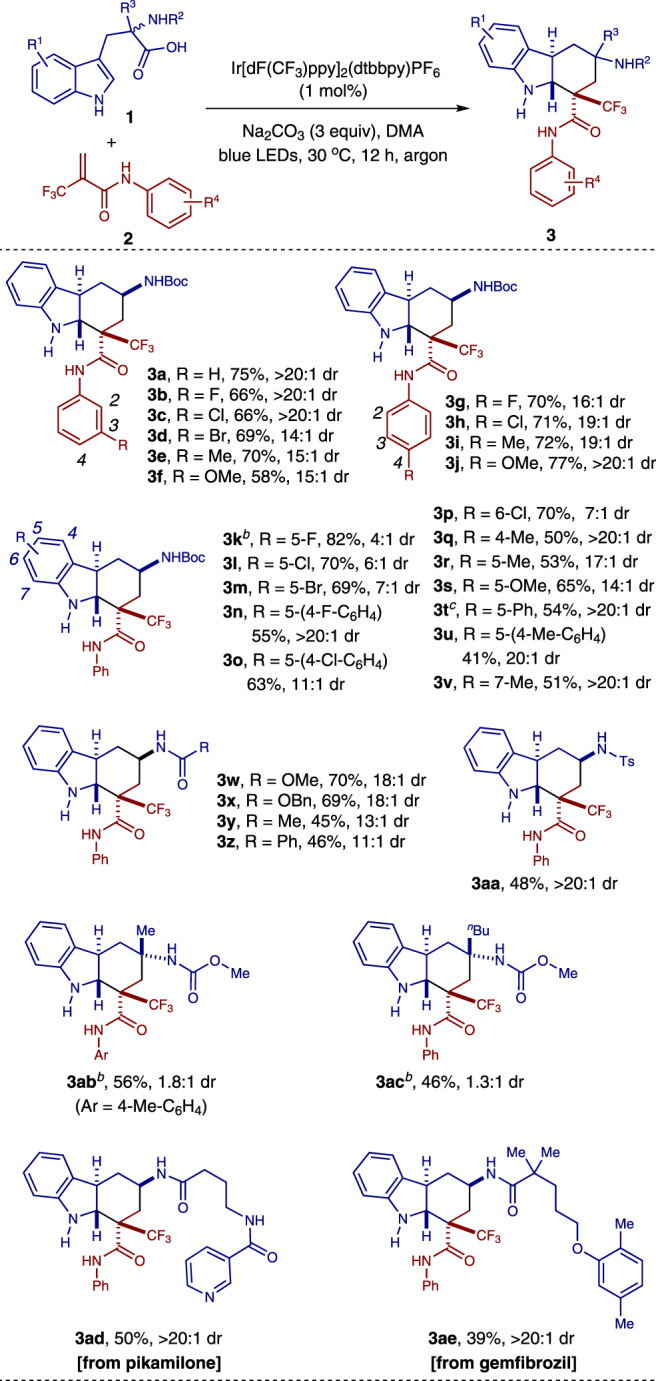
The substrate scope. ^*a*^ Reaction conditions: 1 mol% of Ir[dF(CF_3_)ppy]_2_(dtbbpy)PF_6_, 0.3 mmol of Na_2_CO_3_, 0.15 mmol of **1**, 0.1 mmol of **2** in DMA (2.0 mL) for 12 h under blue LEDs irradiation at 30 °C. Isolated yields of major diastereomers are reported. The dr was determined by crude ^1^H NMR analysis. ^*b*^ The combined yield of both diastereomers. ^*c*^ The reaction was conducted at 40 °C.

With the optimized reaction conditions in hand, various substituted tryptophan derivatives **1** and *N*-aryl-2-(trifluoromethyl)acrylamides **2** were tested to establish the generality of the process (Fig. 2). Reactions of *N*-aryl-2-(trifluoromethyl)acrylamides **2** containing either electron-donating or electron-withdrawing groups on the aromatic ring (R^4^) attached to amide moiety all gave the corresponding products in moderate to good yields and good to excellent diastereoselectivities (**3a** − **3j**). In addition, the tryptophan derivatives **1** containing either electron-donating or electron-withdrawing groups on the phenyl ring of the indole moiety (R^1^) were probed. The reactions occurred smoothly, affording the corresponding *trans*-fused hexahydrocarbazoles **3** in moderate to good yields and moderate to excellent diastereoselectivities (**3k**–**3v**). Moreover, substrates **1** containing different substituents on the nitrogen atom (R^2^) of the amino acid moiety were also tested. It was found that the carbamates, simple amides, and sulfonamide were all tolerated under these reaction conditions, affording the corresponding cyclization products in moderate to good yields and good to excellent diastereoselectivities (**3w**–**3aa**). Furthermore, reactions of ɑ, ɑ-disubstituted ɑ-amino acids also occurred, providing the *trans*-fused hexahydrocarbazoles **3ab**–**3ac** with two quaternary carbon centers in moderate yields. Finally, the present method was applied to the late-stage functionalization of the derivatives of drug molecules, providing easy access to two hexahydrocarbazole-containing complex molecules (**3ad**–**3ae**) in moderate yields and excellent diastereoselectivities. The structure and relative configuration of both diastereomers of the hexahydrocarbazole products were assigned by X-ray crystallographic analyses of compounds **3k** and **3ab** (major diastereomers, see Supplementary Figs. 8 and 9), and compounds **3k**' and **3ab**' (minor diastereomers, see Supplementary Figs. 10 and 11) respectively.

**Fig. 3:**
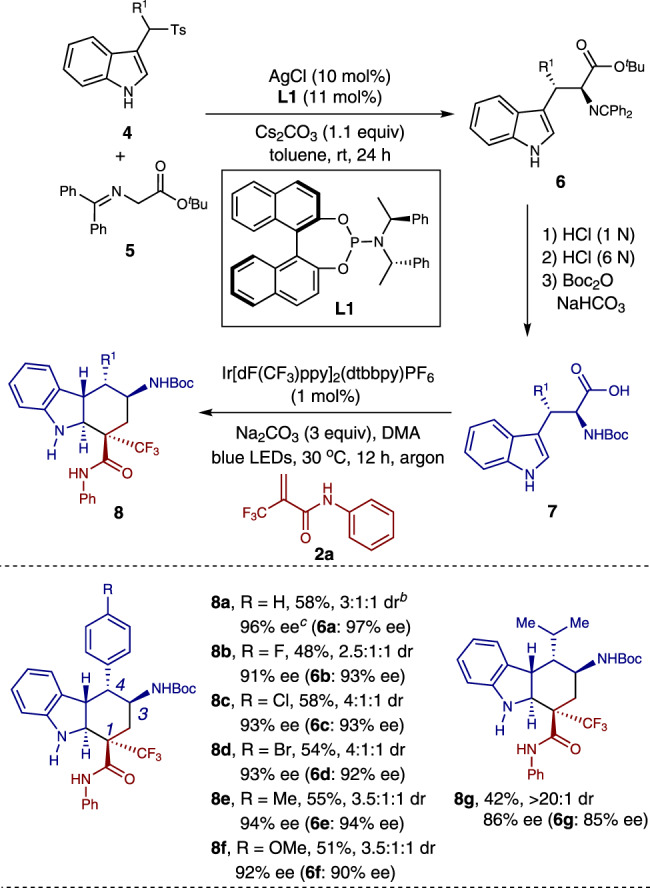
Diastereoselective syntheses of chiral *trans*-fused hexahydrocarbazoles with five contiguous stereogenic centers. ^*a*^ Reaction conditions: 1 mol% of Ir[dF(CF_3_)ppy]_2_(dtbbpy)PF_6_, 0.1 mmol of **2**, 0.15 mmol of **7** and 0.3 mmol of Na_2_CO_3_ in DMA (2.0 mL) for 12 h under blue LEDs irradiation at 30 °C. Combined isolated yields of the diastereomers are reported. ^*b*^ The dr was determined by crude ^1^H NMR analysis. ^*c*^ Enantiomeric excess (ee) of the major diastereomer was reported. The ee value was determined by HPLC analysis on a chiral stationary phase.

Pleasingly, the present method was able to be applied to the diastereoselective syntheses of chiral *trans*-fused hexahydrocarbazoles with five contiguous stereogenic centers (Fig. 3). Reactions of chiral β-aryl-substituted tryptophan derivatives **7** containing either electron-withdrawing or electron-donating groups on the aromatic ring (*R*^1^) attached to β-carbon of the ɑ-amino acid moiety all gave the corresponding chiral *trans*-fused hexahydrocarbazoles bearing five contiguous stereocenters (**8a**–**8f**). In addition, reaction of the β-alkyl-substituted ɑ-amino acid also occurred smoothly, affording the desired product **8g** in moderate yield and excellent diastereoselectivity. Notably, the enantiomeric purity of the starting material **6**, which could be easily prepared via a simple Ag-catalyzed enantioselective reaction of glycine derivatives **5** with sulfonylindoles **4**^59^, was well-preserved under these photocatalytic conditions (**8a**–**8g**). The structure and absolute configuration of the major diastereomers of the hexahydrocarbazole products were assigned by an X-ray crystallographic analysis of compound **8a** (Supplementary Fig. 12). The absolute configuration was determined as (1 *S*, 3 *S*, 4 *R*, 5 *S*, 6 *S*).

**Fig. 4:**
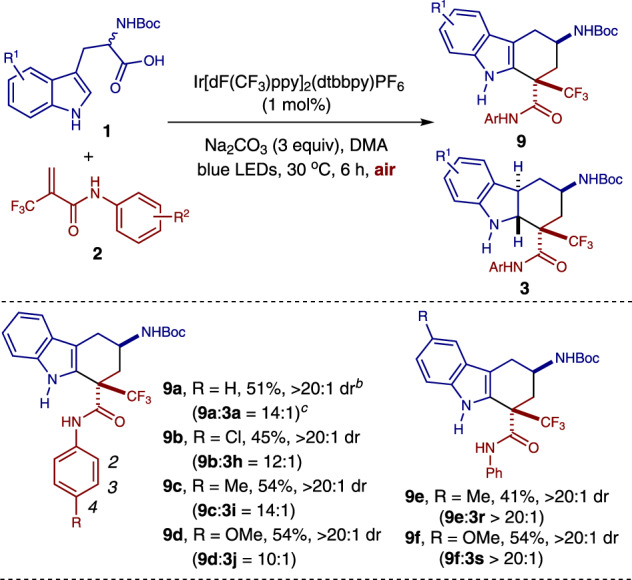
Syntheses of tetrahydrocarbazoles. ^*a*^ Reaction conditions: 1 mol% of Ir[dF(CF_3_)ppy]_2_(dtbbpy)PF_6_, 0.15 mmol of **1**, 0.1 mmol of **2**, and 0.3 mmol of Na_2_CO_3_ in DMA (2.0 mL) for 6 h under blue LEDs irradiation at 30 °C. Isolated yields of compound **9** are reported. ^*b*^ The dr was determined by crude ^1^H NMR analysis. ^*c*^ The ratio was determined by crude ^1^H NMR analysis.

Interestingly, the reaction also proceeded when it was performed under air, affording tetrahydro-1*H*-carbazole **9a** favorably (**9a**:**3a** = 14:1) with moderate yield and excellent diastereoselectivity (Fig. 4, for the experimental setup, see Supplementary Fig. 2). Several substituted tryptophan derivatives **1** and *N*-aryl-2-(trifluoromethyl)acrylamides **2** were tested to establish the generality of the process. Reactions of tryptophan derivatives **1** and *N*-aryl-2-(trifluoromethyl)acrylamides **2** containing either electron-donating or electron-withdrawing groups on the aromatic ring (R^1^ or R^2^) attached to the indole or amide moiety all led to the tetrahydro-1*H*-carbazoles **9** favorably in moderate yields and excellent diastereoselectivities (**9b**–**9f**). The structure and relative configuration of the tetrahydrocarbazole products were assigned by an X-ray crystallographic analysis of compound **9a** (see Supplementary Fig. 13).

### Mechanistic studies

**Fig. 5:**
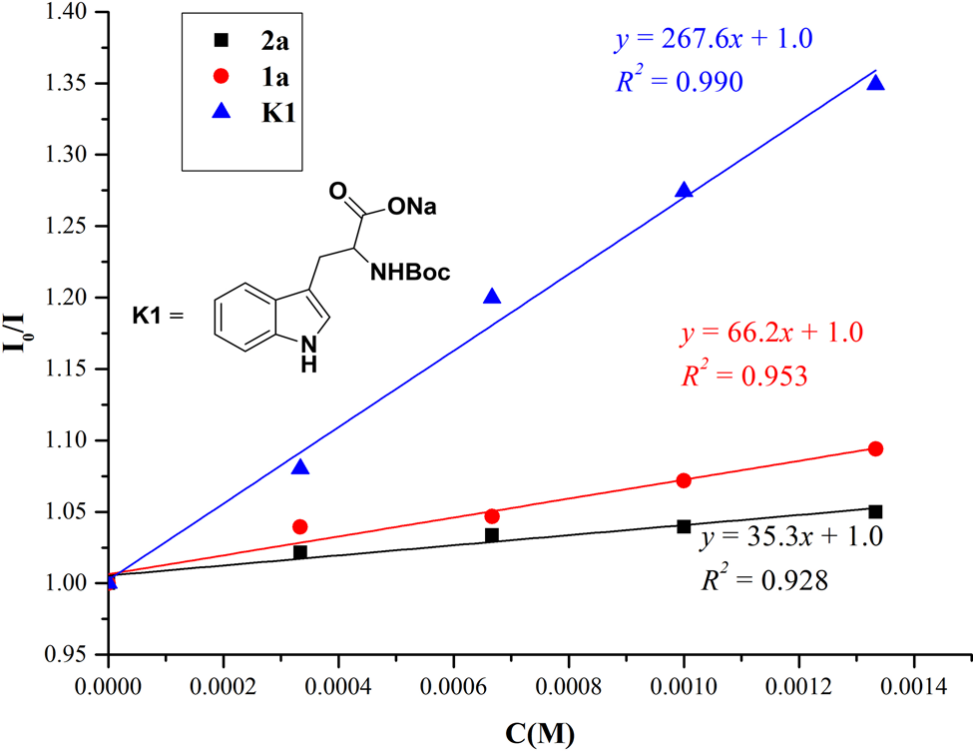
Stern–Volmer quenching experiments of ^*^Ir[dF(CF_3_)ppy]_2_(dtbbpy)PF_6_ with compounds 1a, 2a, and K1. A solution of Ir[dF(CF_3_)ppy]_2_(dtbbpy)PF_6_ in *N*,*N*-dimethylacetamide was excited at 380 nm and the emission intensity at 481 nm was observed.

**Fig. 6:**
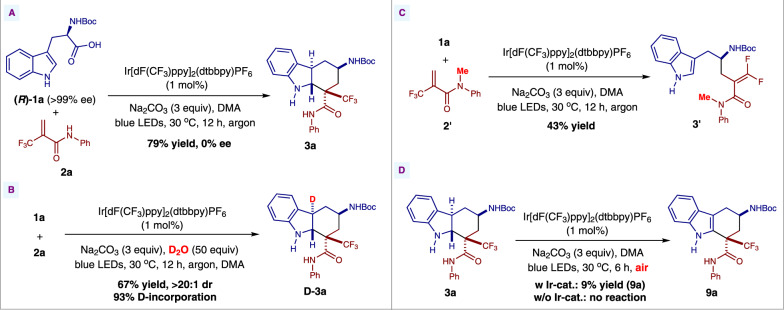
Mechanistic experiments. **A** Reaction of enantiopure (*R*)-*N*-Boc-tryptophan (*R*)-**1a** with substrate **2a**. **B** Deuterium experiment. **C** Reaction of *N*-methyl substituted acrylamide **2'** with substrate **1a**. **D** Control experiments.

**Fig. 7:**
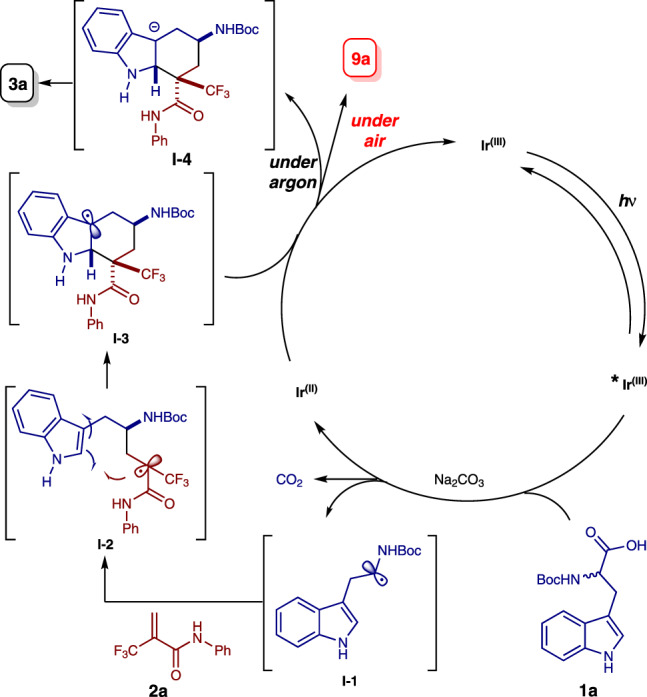
A plausible reaction pathway. A plausible reaction pathway for the formation of products **3a** and **9a** was proposed.

To probe the reaction pathway, preliminary mechanistic studies were carried out (Figs. 5 and 6). It was found that the excited photocatalyst ^*^Ir[dF(CF_3_)ppy]_2_(dtbbpy)PF_6_ could be quenched by acrylamide **2a**, *N*-Boc-tryptophan **1a**, and its sodium salt **K1** respectively according to the Stern–Volmer luminescence quenching studies (Fig. 5, for experimental details, see Supplementary Fig. 7). However, the reductive quenching of ^*^Ir[dF(CF_3_)ppy]_2_(dtbbpy)PF_6_ by either *N*-Boc-tryptophan **1a** or its sodium salt **K1** was more efficient than the oxidative quenching of the excited photocatalyst by substrate **2a**, which indicates the former process is possibly the initiation step of the photoredox catalytic cycle (for the cyclic voltammetry measurement experiments of compounds **1a**, **2a**, and **K1**, see the Supplementary Figs. 3–6). In addition, when the enantiopure (*R*)-*N*-Boc-tryptophan (*R*)-**1a** was utilized under the reaction conditions, the racemic product **3a** was obtained (Fig. 6A). These results suggest that the α-aminoalkyl radical is formed via photoinduced decarboxylation. Furthermore, 93% of deuterium incorporation at the C3-position of the hexahydrocarbazole product was observed when D_2_O was added to the reaction mixture (Fig. 6B). These results suggest that the carbon anion in the benzylic position of indoline moiety is plausibly generated via radical-polar crossover^60,61^. Interestingly, when the *N*-methyl substituted acrylamide **2**' was used as a substrate for the reaction, the competitive radical addition/β-fluoride elimination product **3'** was obtained instead of the hexahydrocarbazole^56^, which indicates that the N–H bond of the amide moiety is very crucial for this unique radical cascade cyclization reaction (Fig. 6C). The plausible hydrogen bonding effect between the amide N–H bond and the CF_3_ moiety might benefit the intramolecular cyclization. Moreover, in order to figure out whether the tetrahydrocarbazole **9a** was formed via oxidation of *trans*-hexahydrocarbazole **3a**, two control experiments were performed. Either low conversion or no reaction was observed when this transformation was carried out using the same conditions as shown in Fig. 4 or the conditions without photocatalyst (Fig. 6D). These results suggest that benzylic radical species **I-3** might be involved as an intermediate during the oxidation process (for details, see Fig. 7) ^62,63^.

Based on the above experimental observations, a plausible reaction pathway was proposed, as shown in Fig. 7. Firstly, the α-aminoalkyl radical **I-1** generated by photoinduced decarboxylation adds to α,β-unsaturated amide **2a** to form radical intermediate **I-2**. This tertiary alkyl radical intermediate would subsequently undergo a radical cyclization onto the indole ring to generate the tertiary benzylic radical intermediate **I-3**. Finally, the hexahydrocarbazole **3a** is obtained via reduction of the benzylic radical intermediate **I-3** by Ir(II) and subsequent protonation under inert atmosphere. Interestingly, when this benzylic radical intermediate **I-3** is exposed to air under the reaction conditions, the tetrahydro-1*H*-carbazole **9a** is formed favorably.

### Rational expansion of the method

**Fig. 8:**
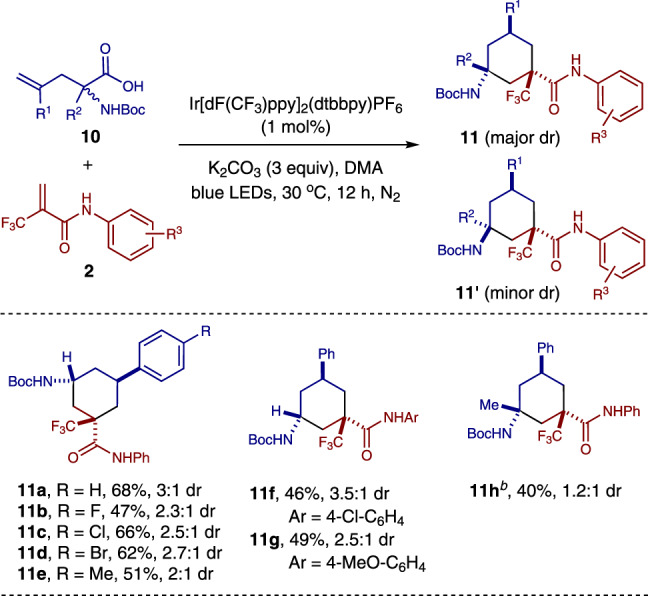
Application to the syntheses of 1,3,5-trisubstituted cyclohexanes. ^*a*^ Reaction conditions: 1 mol% of Ir[dF(CF_3_)ppy]_2_(dtbbpy)PF_6_, 0.1 mmol of **2**, 0.15 mmol of **10** and 0.3 mmol of K_2_CO_3_ in DMA (2.0 mL) for 12 h under blue LEDs irradiation at 30 °C. Combined isolated yields of both diastereomers are reported. The dr was determined by crude ^1^H NMR analysis*.* ^*b*^ The reaction was performed with 2 mol% of Ir[dF(CF_3_)ppy]_2_(dtbbpy)PF_6_, and 2.0 equiv of **10** **h** for 18 h.

To further test the generality of the current method, this radical cascade cyclization strategy was applied to the synthesis of 1,3,5-trisubstituted cyclohexanes (Fig. 8). To our delight, by simply switching the starting material from tryptophans **1** to the alkenyl substituted amino acids **10**, several 1,3,5-trisubstituted cyclohexanes bearing at least one quaternary carbon stereocenter were obtained in moderate yield and up to 3.5:1 dr under the photocatalytic conditions (**11a**–**11h**). Notably, the 1,3,5-trisubstituted cyclohexane with two quaternary carbon stereogenic centers (**11h**) could also be accessed via this one-step radical cascade cyclization reaction. This strategy provides a straightforward and efficient pathway for the construction of 1,3,5-trisubstituted cyclohexanes which are challenging to be synthesized in one step. The structure and relative configuration of both diastereomers of the 1,3,5-trisubstituted cyclohexane products were assigned by X-ray crystallographic analyses of the derivative of compound **11a** (major diastereomer, see Supplementary Fig. 14) and compound **11d**' (minor diastereomer, see Supplementary Fig. 15) respectively.

### Synthetic applications

**Fig. 9:**
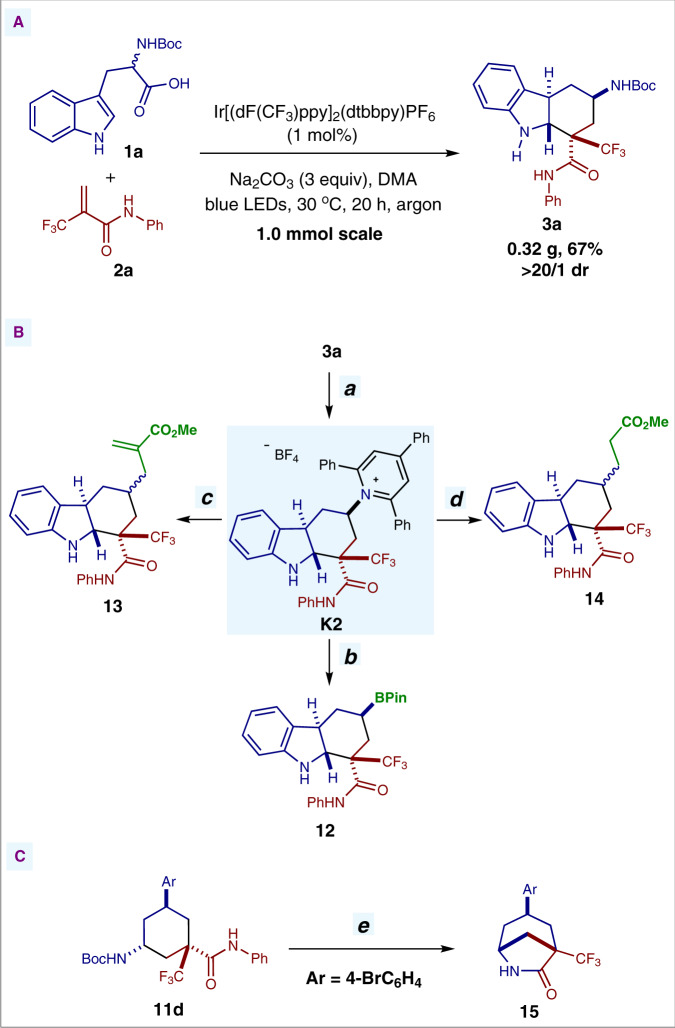
Synthetic application. **A** 1.0 mmol-scale reaction. **B** Synthetic transformations of **3a**. **C** Transformation of **11d**. Reaction conditions: [*a*] (i) CF_3_COOH, CH_2_Cl_2_, 0 °C → rt; (ii) 2,4,6-triphenylpyrylium tetrafluoroborate, EtOH, 140 °C, 68% yield over 2 steps, >20:1 dr. [*b*] (i) Bis(catecholato)diboron, DMA, blue LEDs, 30 °C; (ii) Et_3_N, pinacol, DMA, 30 °C, 57% yield over 2 steps, >20:1 dr. [*c*] Methyl 2-[(phenylsulfonyl)methyl]acrylate, diethyl 2,6-dimethyl-1,4-dihydropyridine-3,5-dicarboxylate, DMA, blue LEDs, 40 °C, 74% yield, 1.8:1 dr. [*d*] Methyl acrylate, diethyl 1,4-dihydro-2,6-dimethyl-3,5-pyridinedicarboxylate, DMA, blue LEDs, 40 °C, 50% yield, 3:1 dr. [*e*] (i) CF_3_COOH, CH_2_Cl_2_, 0 °C → rt; (ii) NaOH, MeOH/H_2_O, 100 °C, 69% yield over 2 steps, >20:1 dr.

Finally, in order to demonstrate the synthetic utility of the present method, a 1.0 mmol-scale reaction and several transformations of the multi-substituted *trans*-fused hexahydrocarbazoles and cyclohexanes were carried out. Under the photocatalytic conditions, 1.0 mmol-scale reaction of substrates **1a** and **2a** occurred smoothly, providing product **3a** in excellent diastereoselectivity and moderate yield (Fig. 9A). In addition, these radical cascade cyclization products are readily transformed to other valuable building blocks. Treatment of the *trans*-fused hexahydrocarbazole **3a** with 2,2,2-trifluoroacetic acid and then 2,4,6-triphenylpyrylium tetrafluoroborate furnished Katritzky pyridinium salt **K2** in excellent diastereoselectivity and moderate yield (path ***a***, Fig. 9B)^64–66^. This pyridinium salt could be further transformed to other structural motifs bearing a *trans*-fused hexahydrocarbazole core through the mild, catalyst-free visible-light-induced deaminative functionalization reactions (paths ***b*-*d***, Fig. 9B)^67,68^. Notably, the *trans*-fused hexahydrocarbazole product **12**, bearing a Bpin group that could be readily applied in the following functionalization, could be easily accessed through this mild photoinduced deaminative borylation in excellent diastereoselectivity and moderate yield (path ***b***, Fig. 9B)^67^. Interestingly, the bridged lactam-containing product **15** could be prepared in excellent diastereoselectivity and moderate yield through a simple two-step transformation of 1,3,5-trisubstituted cyclohexane **11d** (path ***e***, Fig. 9C).

## Discussion

In this work, we have achieved the stereoselective intermolecular radical cascade reactions of readily available tryptophans and acrylamides via visible-light-induced photoredox catalysis, thus providing easy access to an array of *trans*-fused hexahydrocarbazoles with up to five stereogenic centers including two quaternary ones in up to 82% yield, >20/1 diastereoselectivity, and 96% ee. The reaction is distinguished by its broad substrate scope, its power in the rapid generation of molecular complexity from simple starting materials, environmentally friendly reaction conditions, and application to the late-stage functionalization. In addition, the reaction can be diverted to the syntheses of tetrahydrocarbazoles when it is performed under air. Moreover, this radical cascade protocol can further be applied to the stereoselective syntheses of 1,3,5-trisubstituted cyclohexanes by simply switching the starting material from tryptophans to ɤ-alkenyl substituted α-amino acids. This strategy allows the rapid preparation of *trans*-fused hexahydrocarbazoles and cyclohexanes with multiple stereocenters from simple amino acids and acrylamides. Further studies on the reaction mechanism and the development of other stereoselective intermolecular radical cascade reactions via visible-light-induced photoredox catalysis are ongoing in our laboratory.

## Methods

### Representative procedure for the synthesis of *trans*-fused hexahydrocarbazole 3a

To a Young Schlenk tube (10 mL) were added Ir[dF(CF_3_)ppy]_2_(dtbbpy)PF_6_ (1.1 mg, 0.001 mmol, 1 mol%), **1a** (45.7 mg, 0.15 mmol, 1.5 equiv), **2a** (21.5 mg, 0.1 mmol, 1.0 equiv), Na_2_CO_3_ (31.8 mg, 0.3 mmol, 3.0 equiv), and N,N-dimethylacetamide (DMA, 2.0 mL). Subsequently, the reaction mixture was degassed through several freeze-pump-thaw cycles until no bubbles were released. The reaction mixture was stirred under argon at 30 °C, and irradiated by a 5 W blue LED lamp (*λ* = 450–460 nm, the tube was placed at ~2 cm away from the light source). After 12 h, the reaction mixture was passed through a short pad of celite and washed with ethyl acetate. The solvents were evaporated under reduced pressure to give the crude mixture, which was purified by flash column chromatography on silica gel (petroleum ether/ethyl acetate = 40:1 to 15:1, *silica gel was soaked with a solution of petroleum ether and triethylamine (1000/1, v/v) before use*) to afford the title compound **3a** as a white solid (35.7 mg, 75% yield).

## Supplementary information

Supplementary Information

## Data Availability

The X-ray crystallographic coordinates for structures reported in this article have been deposited at the Cambridge Crystallographic Data Centre (CCDC), under deposition number CCDC 2108643 (**3k**), 2108649 (**3ab**), 2108644 (**3k'**), 2108650 (**3ab**'), 2108651 (**8a**), 2108652 (**9a**), 2108653 (the derivative of compound **11a**), and 2108655 (**11d**'). The data can be obtained free of charge from The Cambridge Crystallographic Data Centre via http://www.ccdc.cam.ac.uk/data_request/cif. Complete experimental procedures and compound characterization data are available in the Supplementary Information or from the corresponding author upon request.
