# Supplementary material for: Stereoselective intermolecular radical cascade reactions of tryptophans or ɤ-alkenyl-α-amino acids with acrylamides via photoredox catalysis
Source: Nat Commun. 2022 Apr 1;13:1778. doi: 10.1038/s41467-022-29464-5 (PMC8976070; doi:10.1038/s41467-022-29464-5)
Supplement: Supplementary file 1 — Supplementary Information [file 41467_2022_29464_MOESM1_ESM.pdf]

Stereoselective intermolecular radical cascade reactions of tryptophans or ɤ-alkenyl-α-amino acids with acrylamides via photoredox catalysis

Jiang-Tao Li[1], Jian-Nan Luo[1], Jia-Le Wang[1], De-Ku Wang[1], Yi-Zhe Yu[1] & Chun-Xiang Zhuo[1]*

[1] State Key Laboratory of Physical Chemistry of Solid Surfaces, Key Laboratory of Chemical Biology of Fujian Province, and College of Chemistry and Chemical Engineering, Xiamen University, Xiamen 361005, P. R. China

Email: cxzhuo@xmu.edu.cn

Supplementary Information

Table of Contents

1. General information. S-2
2. Experimental details and characterization data. S-5
3. Mechanistic studies. S-57
4. Crystallographic information. S-62
5. Signal assignment of compounds **3j**, **8e**, **9d**, and **11f**. S-72
6. Copies of HPLC spectra. S-76
7. Copies of NMR spectra. S-85
8. References. S-368

1. General information.

Unless stated otherwise, all reactions were carried out in oven-dried glassware using anhydrous solvents under argon or N_2 atmosphere. The solvents were purified by distillation over the following drying agents and were transferred under argon: toluene (Na); DMA were purchased from Energy chemical and kept in a sealed bottle containing 4 Å molecular sieve under argon. General-Reagent silica gel (300-400 mesh) was used for the flash column chromatography. All substrates whose syntheses were not described herein were either obtained from commercial suppliers or prepared using the referenced literature procedures.[1–8] Unless stated otherwise, all commercially available compounds (Energy Chemical, Bidepharmatech) were used as received. The photoreactor shown in Supplementary Figure 1 was purchased from Wuhan GeAo Chemical.

NMR spectra were recorded on Bruker AV-400 MHz or Bruker AV-500 MHz spectrometers in the solvents indicated; chemical shifts (δ =) are given in ppm, coupling constants (*J*) in Hz. The solvent signals were used as references ($CDCl_3$: δ_C = 77.0 ppm; residual $CHCl_3$ in $CDCl_3$: δ_H = 7.26 ppm; C_3D_6O: δ_C = 206.0 ppm; residual C_3H_6O in C_3D_6O: δ_H = 2.05 ppm; CD_2Cl_2: δ_C = 54.0 ppm; residual CH_2Cl_2 in CD_2Cl_2: δ_H = 5.32 ppm; C_2D_6OS: δ_C = 39.6 ppm; residual C_2H_6OS in C_2D_6OS: δ_H = 2.5 ppm). Infrared (IR) spectra were measured on a Nicolet AVATER FTIR330 spectrometer. High-resolution mass spectra (ESI) were recorded on a Micromass QTOF2 Quadruple/Time-of-Flight Tandem mass spectrometer. Cyclic voltammograms were obtained on a CHI 760E potentiostat. Chiral HPLC analyses were conducted on a Shimadzu LC 20 instrument. Optical rotations ($[\alpha]_D^{25}$ =) were measured with Anton paar MCP500.

The syntheses of compounds **3**, **8**, and **11** were performed in a photoreactor as shown in Supplementary Figure 1.

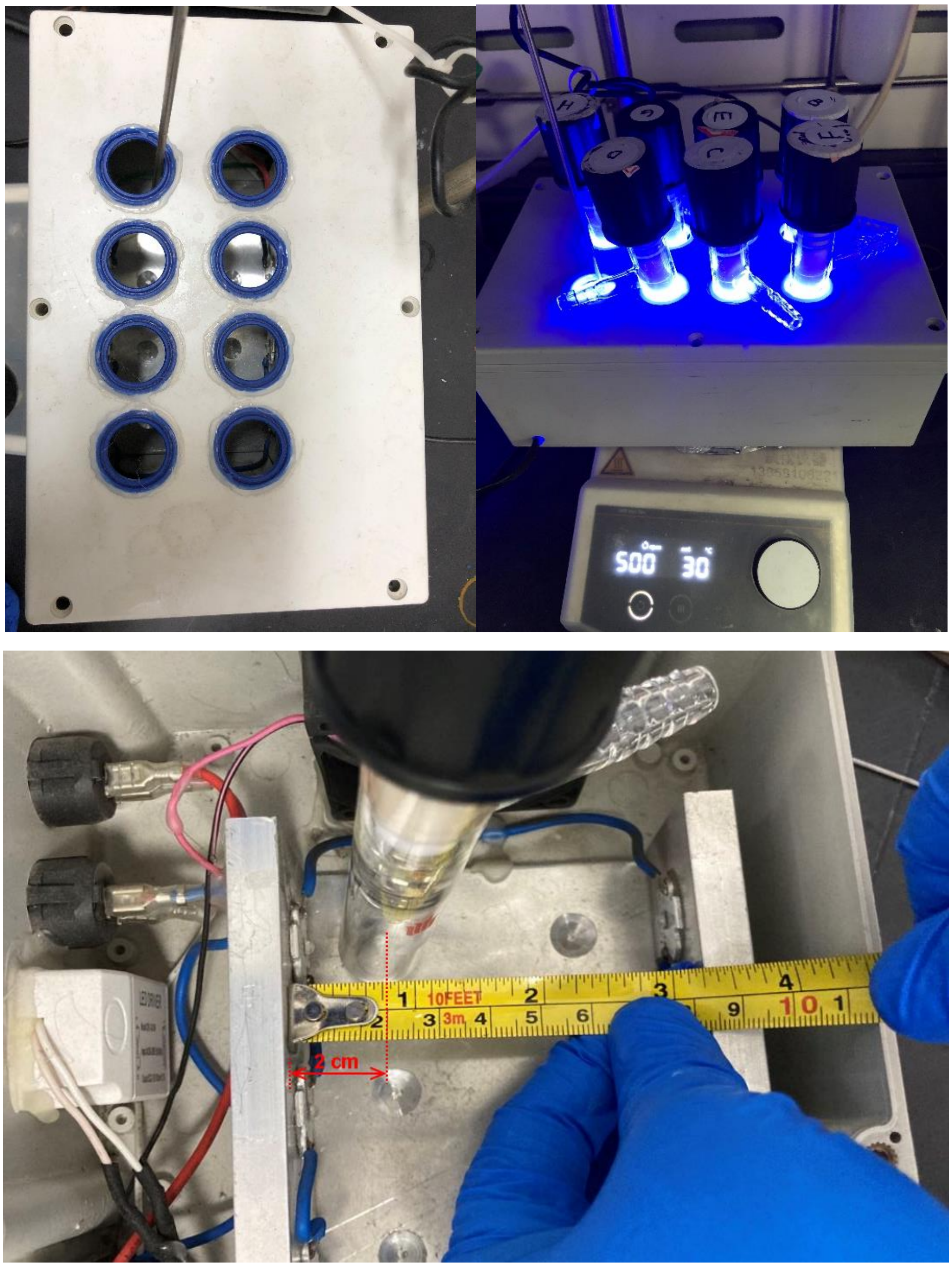

Supplementary Figure 1. Experimental setup for the photochemical reaction.

The syntheses of compounds **9** were performed in a photoreactor as shown in Supplementary Figure 2.

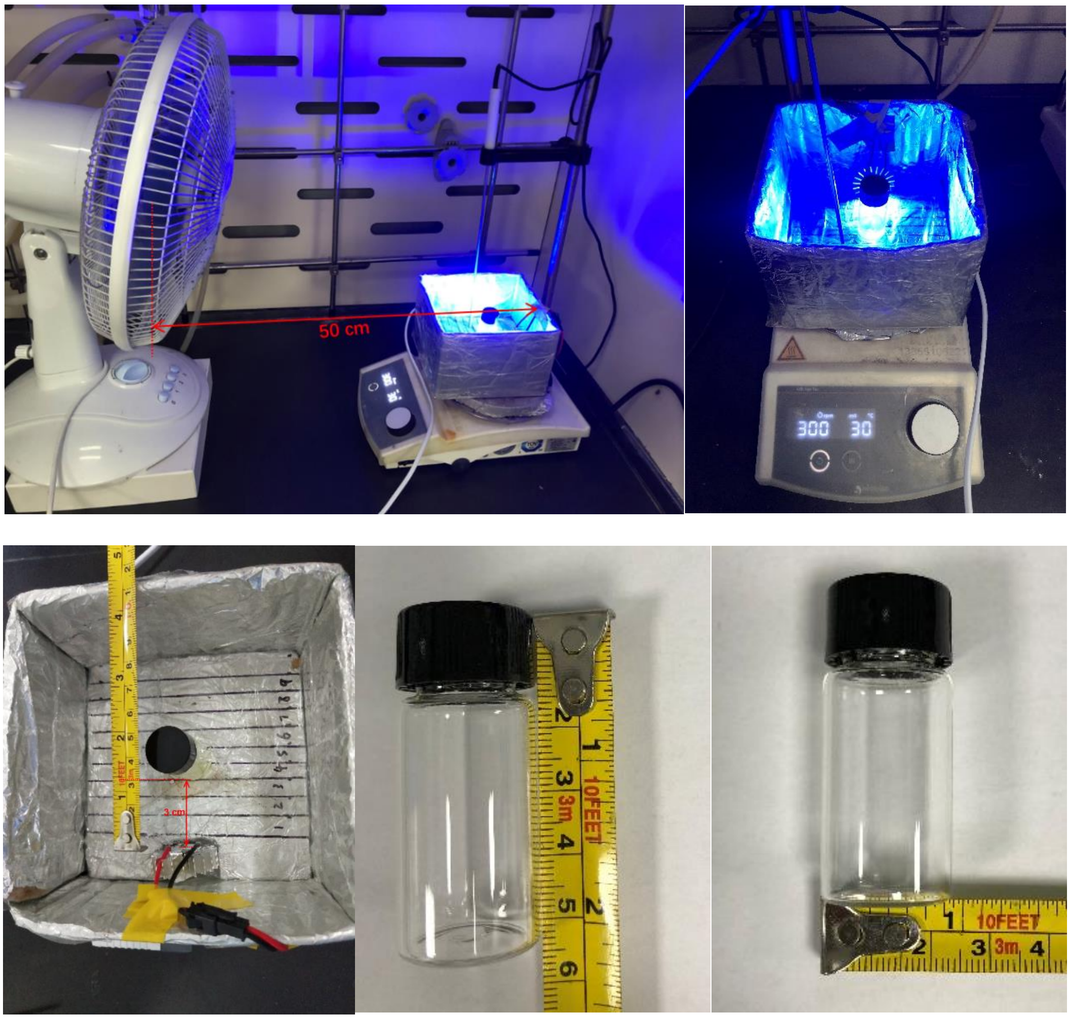

Supplementary Figure 2. Experimental setup for the photochemical reaction.

2. Experimental details and characterization data.

2.1 Representative procedure for the stereoselective radical cascade reaction of tryptophan derivatives 1 and alkenes 2 under photoredox catalysis.

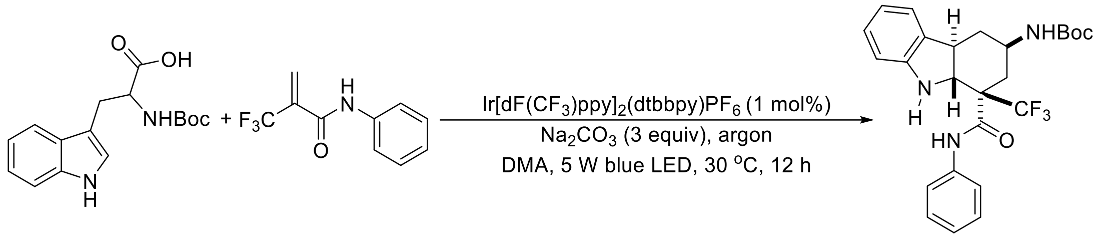

1a 2a 3a

Preparation of compound 3a. To a Young Schlenk tube (10 mL) were added Ir[dF(CF_3)ppy]$_2$(dtbbpy)PF_6 (1.1 mg, 0.001 mmol, 1 mol%), **1a** (45.7 mg, 0.15 mmol, 1.5 equiv), **2a** (21.5 mg, 0.1 mmol, 1.0 equiv), Na_2CO_3 (31.8 mg, 0.3 mmol, 3.0 equiv), and N,N-dimethylacetamide (DMA, 2.0 mL). Subsequently, the reaction mixture was degassed through several freeze-pump-thaw cycles until no bubbles were released. The reaction mixture was stirred under argon at 30 °C, and irradiated by a 5 W blue LED lamp (λ = 450–460 nm, the tube was placed at approximately 2 cm away from the light source). After 12 h, the reaction mixture was passed through a short pad of celite and washed with ethyl acetate. The solvents were evaporated under reduced pressure to give the crude mixture, which was purified by flash column chromatography on silica gel (petroleum ether/ethyl acetate = 40:1 to 15:1, *silica gel was soaked with a solution of petroleum ether and triethylamine (1000/1, v/v) before use*) to afford the title compound **3a** as a white solid (35.7 mg, 75% yield). ^{1}H NMR (400 MHz, C_3D_6O): δ = 10.86 (s, 1H), 7.57 – 7.48 (m, 2H), 7.33 – 7.24 (m, 2H), 7.19 – 7.11 (m, 2H), 7.09 – 7.00 (m, 2H), 6.88 (t, J = 7.4 Hz, 1H), 6.17 (d, J = 6.9 Hz, 1H), 6.04 (d, J = 4.3 Hz, 1H), 3.98 – 3.85 (m, 1H), 3.82 (dd, J = 13.4, 4.5 Hz, 1H), 3.17 (t, J = 11.7 Hz, 1H), 2.96 – 2.87 (m, 1H), 2.68 – 2.58 (m, 1H), 1.70 (t, J = 12.3 Hz, 1H), 1.61 – 1.51 (m, 1H), 1.43 (s, 9H). ^{13}C NMR (100 MHz, C_3D_6O): δ = 163.6, 155.4, 150.2, 138.6, 131.5, 129.4, 128.3, 126.4 (q, $J_{F\text{-}C}$ = 282.1 Hz), 124.8, 123.0, 121.8, 120.1, 113.0, 78.5, 67.2, 55.3 (q, $J_{F\text{-}C}$ = 23.3 Hz), 47.3, 43.8, 34.1, 33.6, 28.3. ^{19}F NMR (376 MHz, $CDCl_3$): δ = -71.2. IR (thin film): ν_{max} (cm^{-1}) = 3341, 2976, 2927, 1690, 1601, 1269, 1167; HRMS (ESI) calcd for $C_{25}H_{28}F_3N_3O_3Na$ $[M+Na]^+$: 498.1975. Found: 498.1975.

The following compounds **3b–3ae** were prepared analogously.

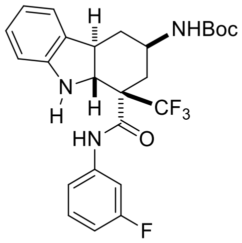

3b. White solid, 32.5 mg, 66% yield. ^{1}H NMR (400 MHz, C_3D_6O): δ = 11.09 (s, 1H), 7.70 – 7.55 (m, 1H), 7.30 (dd, *J* = 14.8, 8.2 Hz, 1H), 7.22 – 7.08 (m, 3H), 7.03 (d, *J* = 7.7 Hz, 1H), 6.95 – 6.76 (m, 2H), 6.16 (d, *J* = 7.6 Hz, 1H), 6.04 (d, *J* = 4.2 Hz, 1H), 4.00 – 3.86 (m, 1H), 3.83 (dd, *J* = 13.4, 4.5 Hz, 1H), 3.18 (t, *J* = 11.9 Hz, 1H), 2.99 – 2.89 (m, 1H), 2.71 – 2.56 (m, 1H), 1.71 (t, *J* = 12.3 Hz, 1H), 1.56 (app q, *J* = 11.9 Hz, 1H), 1.43 (s, 9H). ^{13}C NMR (100 MHz, C_3D_6O): δ = 164.2, 163.5 (d, $J_{F\text{-}C}$ = 241.4 Hz), 155.5, 150.3, 140.4 (d, $J_{F\text{-}C}$ = 10.9 Hz), 131.7, 131.1 (d, $J_{F\text{-}C}$ = 9.5 Hz), 128.5, 126.5 (q, $J_{F\text{-}C}$ = 282.0 Hz), 123.2, 122.1, 115.87 (d, $J_{F\text{-}C}$ = 2.8 Hz), 113.4, 111.4 (d, $J_{F\text{-}C}$ = 21.3 Hz), 107.4 (d, $J_{F\text{-}C}$ = 26.5 Hz), 78.7, 67.3, 55.5 (q, $J_{F\text{-}C}$ = 23.3 Hz), 47.5, 44.1, 34.3, 33.7, 28.5. ^{19}F NMR (376 MHz, $CDCl_3$): δ = -71.6, -111.5. IR (thin film): ν_{max} (cm^{-1}) = 3333, 2977, 2927, 1695, 1615, 1492, 1168; HRMS (ESI) calcd for $C_{25}H_{27}F_4N_3O_3Na$ [M+Na]$^+$: 516.1881. Found: 516.1880.

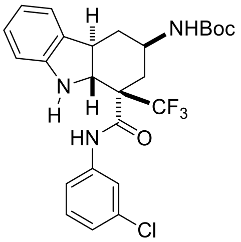

3c. White solid, 33.5 mg, 66% yield. ^{1}H NMR (400 MHz, C_3D_6O): δ = 11.06 (s, 1H), 7.87 – 7.77 (m, 1H), 7.39 – 7.22 (m, 2H), 7.19 – 7.07 (m, 3H), 7.02 (d, *J* = 7.7 Hz, 1H), 6.89 (t, *J* = 7.4 Hz, 1H), 6.15 (d, *J* = 7.3 Hz, 1H), 6.03 (d, *J* = 4.3 Hz, 1H), 4.01 – 3.86 (m, 1H), 3.82 (dd, *J* = 13.4, 4.5 Hz, 1H), 3.19 (t, *J* = 11.9 Hz, 1H), 2.98 – 2.88 (m, 1H), 2.72 – 2.56 (m, 1H), 1.71 (t, *J* = 12.4 Hz, 1H), 1.56 (app q, *J* = 11.9 Hz, 1H), 1.43 (s, 9H). ^{13}C NMR (100 MHz, C_3D_6O): δ = 164.3, 155.5, 150.3, 140.1, 134.7, 131.8 131.1, 128.5, 126.5 (q, $J_{F\text{-}C}$ = 282.0 Hz), 124.8, 123.3, 122.1, 120.2, 118.6, 113.4, 78.7, 67.3, 55.6 (q, $J_{F\text{-}C}$ = 23.2 Hz), 47.5, 44.1, 34.3, 33.7, 28.5. ^{19}F NMR (376 MHz, $CDCl_3$): δ = -71.6. IR (thin film): ν_{max} (cm^{-1}) = 3350, 3053, 2978, 1698, 1596, 1483, 1168; HRMS (ESI) calcd for $C_{25}H_{27}ClF_3N_3O_3Na$ [M+Na]$^+$: 532.1585. Found: 532.1583.

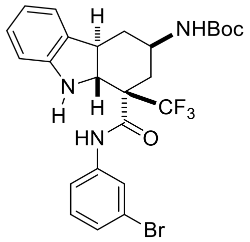

3d. White solid, 38.0 mg, 69% yield. ^{1}H NMR (400 MHz, C_3D_6O): δ = 11.05 (s, 1H), 7.99 – 7.94 (m, 1H), 7.43 – 7.35 (m, 1H), 7.29 – 7.20 (m, 2H), 7.19 – 7.10 (m, 2H), 7.02 (d, *J* = 7.7 Hz, 1H), 6.94 – 6.85 (m, 1H), 6.15 (d, *J* = 7.6 Hz, 1H), 6.03 (d, *J* = 4.3 Hz, 1H), 3.99 – 3.84 (m, 1H), 3.82 (dd, *J* = 13.4, 4.5 Hz, 1H), 3.19 (t, *J* = 11.7 Hz, 1H), 3.01 – 2.87 (m, 1H), 2.70 – 2.58 (m, 1H), 1.70 (t, *J* = 12.4 Hz, 1H), 1.56 (app q, *J* = 11.9 Hz, 1H), 1.43 (s, 9H). ^{13}C NMR (100 MHz, C_3D_6O): δ = 164.3, 155.5, 150.3, 140.2, 131.8, 131.4, 128.5, 127.8, 126.5 (q, $J_{F\text{-}C}$ = 282.1 Hz), 123.3, 123.0, 122.7, 122.1, 119.1, 113.4, 78.7, 67.3, 55.6 (q, $J_{F\text{-}C}$ = 23.3 Hz), 47.5, 44.1, 34.3, 33.7, 28.5. ^{19}F NMR (376 MHz, $CDCl_3$): δ = -71.6. IR (thin film): ν_{max} (cm^{-1}) = 3333, 3050, 2978, 1698, 1593, 1479, 1168; HRMS (ESI) calcd for $C_{25}H_{27}BrF_3N_3O_3Na$ $[M+Na]^+$: 576.1080. Found: 576.1079.

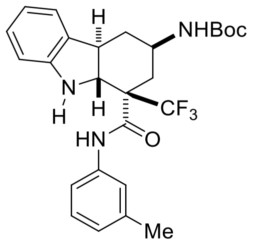

3e. White solid, 34.2 mg, 70% yield. ^{1}H NMR (400 MHz, C_3D_6O): δ = 10.78 (s, 1H), 7.40 (s, 1H), 7.31 (d, *J* = 7.6 Hz, 1H), 7.22 – 7.08 (m, 3H), 7.02 (d, *J* = 7.7 Hz, 1H), 6.96 – 6.83 (m, 2H), 6.14 (d, *J* = 6.6 Hz, 1H), 6.01 (d, *J* = 3.5 Hz, 1H), 3.99 – 3.85 (m, 1H), 3.81 (dd, *J* = 13.4, 4.3 Hz, 1H), 3.17 (t, *J* = 12.5 Hz, 1H), 3.00 – 2.88 (m, 1H), 2.71 – 2.55 (m, 1H), 2.26 (s, 3H), 1.69 (t, *J* = 12.2 Hz, 1H), 1.55 (app q, *J* = 11.9 Hz, 1H), 1.44 (s, 9H). ^{13}C NMR (100 MHz, C_3D_6O): δ = 163.7, 155.5, 150.5, 139.4, 138.8, 131.7, 129.5, 128.5, 126.7 (q, $J_{F\text{-}C}$ = 282.0 Hz), 125.7, 123.2, 122.0, 120.8, 117.4, 113.3, 78.7, 67.5, 55.5 (q, $J_{F\text{-}C}$ = 23.1 Hz), 47.5, 44.0, 34.4, 33.8, 28.5, 21.3. ^{19}F NMR (376 MHz, $CDCl_3$): δ = -71.7. IR (thin film): ν_{max} (cm^{-1}) = 3332, 3054, 2978, 1694, 1564, 1268, 1167; HRMS (ESI) calcd for $C_{26}H_{30}F_3N_3O_3Na$ $[M+Na]^+$: 512.2131. Found: 512.2130.

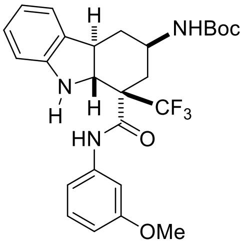

3f. White solid, 29.3 mg, 58% yield. ^{1}H NMR (400 MHz, C_3D_6O): δ = 10.88 (s, 1H), 7.43 – 7.33 (m, 1H), 7.27 – 7.09 (m, 3H), 7.02 (d, *J* = 7.7 Hz, 1H), 6.99 – 6.83 (m, 2H), 6.73 – 6.58 (m, 1H), 6.14 (d, *J* = 7.5 Hz, 1H), 6.01 (d, *J* = 4.3 Hz, 1H), 4.06 – 3.86 (m, 1H), 3.82 (dd, *J* = 13.4, 4.5 Hz, 1H), 3.74 (s, 3H), 3.16 (t, *J* = 12.0 Hz, 1H), 3.01 – 2.88 (m, 1H), 2.72 – 2.54 (m, 1H), 1.70 (t, *J* = 12.3 Hz, 1H), 1.55 (app q, *J* = 11.9 Hz, 1H), 1.43 (s, 9H). ^{13}C NMR (100 MHz, C_3D_6O): δ = 163.8, 161.0, 155.5, 150.4, 140.0, 131.7, 130.4, 128.5, 126.6 (q, $J_{F\text{-}C}$ = 281.9 Hz), 123.3, 122.0, 113.3, 112.4, 110.4, 106.2, 78.7, 67.4, 55.5 (q, $J_{F\text{-}C}$ = 23.4 Hz), 55.3, 47.5, 44.1, 34.4, 33.7, 28.5. ^{19}F NMR (376 MHz, $CDCl_3$): δ = -71.6. IR (thin film): ν_{max} (cm^{-1}) = 3325, 3052, 2977, 1697, 1600, 1229, 1167; HRMS (ESI) calcd for $C_{26}H_{30}F_3N_3O_4Na$ $[M+Na]^+$: 528.2081. Found: 528.2079.

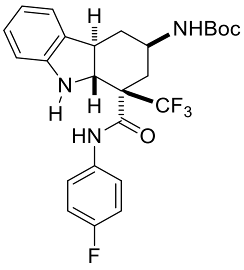

3g. White solid, 34.5 mg, 70% yield. ^{1}H NMR (400 MHz, C_3D_6O): δ = 10.89 (s, 1H), 7.62 – 7.52 (m, 2H), 7.20 – 7.11 (m, 2H), 7.10 – 7.03 (m, 2H), 7.01 (d, *J* = 7.8 Hz, 1H), 6.89 (t, *J* = 7.4 Hz, 1H), 6.15 (d, *J* = 6.9 Hz, 1H), 6.02 (d, *J* = 4.2 Hz, 1H), 3.94 – 3.85 (m, 1H), 3.81 (dd, *J* = 13.4, 4.5 Hz, 1H), 3.17 (t, *J* = 11.8 Hz, 1H), 2.95 – 2.90 (m, 1H), 2.68 – 2.59 (m, 1H), 1.70 (t, *J* = 12.3 Hz, 1H), 1.56 (app q, *J* = 11.9 Hz, 1H), 1.43 (s, 9H). ^{13}C NMR (100 MHz, C_3D_6O): δ = 163.8, 159.8 (d, $J_{F\text{-}C}$ = 240.1 Hz), 155.5, 150.3, 135.0 (d, $J_{F\text{-}C}$ = 2.7 Hz), 131.7, 128.4, 126.5 (q, $J_{F\text{-}C}$ = 282.0 Hz), 123.2, 122.1 (d, $J_{F\text{-}C}$ = 7.9 Hz), 121.9, 116.0 (d, $J_{F\text{-}C}$ = 22.5 Hz), 113.2, 78.6, 67.3, 55.3 (q, $J_{F\text{-}C}$ = 23.2 Hz), 47.4, 44.0, 34.2, 33.7, 28.4. ^{19}F NMR (376 MHz, $CDCl_3$): δ = -71.6, -117.5. IR (thin film): ν_{max} (cm^{-1}) = 3331, 3051, 2978, 1693, 1614, 1509, 1221, 1168; HRMS (ESI) calcd for $C_{25}H_{27}F_4N_3O_3Na$ $[M+Na]^+$: 516.1881. Found: 516.1880.

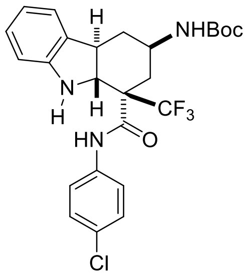

3h. White solid, 36.2 mg, 71% yield. ^{1}H NMR (400 MHz, C_3D_6O): δ = 10.98 (s, 1H), 7.61 – 7.52 (m, 2H), 7.37 – 7.30 (m, 2H), 7.21 – 7.10 (m, 2H), 7.01 (d, *J* = 7.8 Hz, 1H), 6.89 (t, *J* = 7.4 Hz, 1H), 6.14 (d, *J* = 7.0 Hz, 1H), 6.02 (d, *J* = 4.3 Hz, 1H), 3.96 – 3.85(m, 1H), 3.82 (dd, *J* = 13.4, 4.5 Hz, 1H), 3.17 (t, *J* = 11.6 Hz, 1H), 2.94 – 2.87 (m, 1H), 2.68 – 2.59 (m, 1H), 1.70 (t, *J* = 12.4 Hz, 1H), 1.56 (app q, *J* = 11.9 Hz, 1H), 1.43 (s, 9H). ^{13}C NMR (100 MHz, C_3D_6O): δ = 164.0, 155.5, 150.3, 137.6, 131.7, 129.5 129.3, 128.5, 126.5 (q, $J_{F\text{-}C}$ = 282.1 Hz), 123.2, 122.0, 121.8, 113.3, 78.7, 67.3, 55.5 (q, $J_{F\text{-}C}$ = 23.3 Hz), 47.4, 44.0, 34.2, 33.7, 28.4. ^{19}F NMR (376 MHz, $CDCl_3$): δ = -71.6. IR (thin film): ν_{max} (cm^{-1}) = 3324, 2978, 2929, 1696, 1598, 1546, 1492, 1167; HRMS (ESI) calcd for $C_{25}H_{27}ClF_3N_3O_3Na$ $[M+Na]^+$: 532.1585. Found: 532.1585.

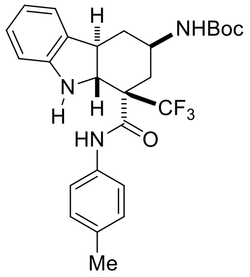

3i. White solid, 35.2 mg, 72% yield. ^{1}H NMR (400 MHz, C_3D_6O): δ = 10.75 (s, 1H), 7.46 – 7.37 (m, 2H), 7.20 – 7.07 (m, 4H), 7.01 (d, *J* = 7.8 Hz, 1H), 6.88 (t, *J* = 7.4 Hz, 1H), 6.14 (d, *J* = 7.3 Hz, 1H), 6.00 (d, *J* = 4.3 Hz, 1H), 3.95 – 3.84 (m, 1H), 3.84 – 3.77 (m, 1H), 3.16 (t, *J* = 11.7 Hz, 1H), 2.94 – 2.89 (m, 1H), 2.69 – 2.59 (m, 1H), 2.25 (s, 3H), 1.68 (t, *J* = 12.3 Hz, 1H), 1.54 (app q, *J* = 11.9 Hz, 1H), 1.43 (s, 9H). ^{13}C NMR (100 MHz, C_3D_6O): δ = 163.5, 155.5, 150.4, 136.3, 134.3, 131.6, 129.9, 128.4, 126.6 (q, $J_{F\text{-}C}$ = 282.1 Hz), 123.1, 121.9, 120.2, 113.1, 78.6, 67.4, 55.3 (q, $J_{F\text{-}C}$ = 23.2 Hz), 47.5, 44.0, 34.3, 33.7, 28.4, 20.5. ^{19}F NMR (376 MHz, $CDCl_3$): δ = -71.7. IR (thin film): ν_{max} (cm^{-1}) = 3335, 3052, 2979, 1694, 1612, 1514, 1269, 1167; HRMS (ESI) calcd for $C_{26}H_{30}F_3N_3O_3Na$ $[M+Na]^+$: 512.2131. Found: 512.2130.

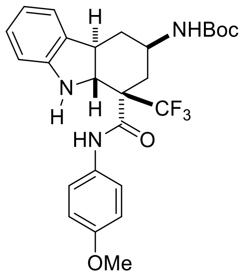

3j. White solid, 38.8 mg, 77% yield. ^{1}H NMR (400 MHz, C_3D_6O): δ =^{1}H NMR (400 MHz, Acetone) δ 10.67 (s, 1H), 7.51 – 7.39 (m, 2H), 7.22 – 7.08 (m, 2H), 7.03 – 6.96 (m, 1H), 6.94 – 6.87 (m, 1H), 6.87 – 6.82 (m, 2H), 6.13 (d, J = 6.8 Hz, 1H), 5.97 (d, J = 4.1 Hz, 1H), 3.95 – 3.85 (m, 1H), 3.81 (dd, J = 13.4, 4.4 Hz, 1H), 3.73 (s, 3H), 3.22 – 3.11 (m, 1H), 2.96 – 2.87 (m, 1H), 2.68 – 2.57 (m, 1H), 1.76 – 1.62 (m, 1H), 1.60 – 1.49 (m, 1H), 1.43 (s, 9H). ^{13}C NMR (100 MHz, C_3D_6O): δ = 163.4, 157.3, 155.5, 150.5, 132.0, 131.8, 128.5, 126.7 (q, $J_{F\text{-}C}$ = 281.7 Hz), 123.2, 121.9, 121.8, 114.7, 113.2, 78.7, 67.5, 55.5, 55.3 (q, $J_{F\text{-}C}$ = 23.0 Hz), 47.6, 44.0, 34.4, 33.8, 28.5. ^{19}F NMR (376 MHz, $CDCl_3$): δ = -71.7. IR (thin film): ν_{max} (cm^{-1}) = 3332, 3054, 2977, 1690, 1613, 1512, 1241, 1168; HRMS (ESI) calcd for $C_{26}H_{30}F_3N_3O_4Na$ $[M+Na]^+$: 528.2081. Found: 528.2080.

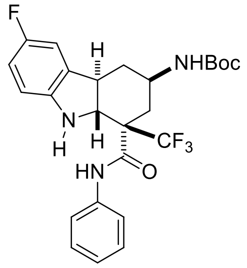

3k (major dr)

3k (major dr). White solid, 33.5 mg, 68% yield. ^{1}H NMR (400 MHz, C_3D_6O): δ = 10.85 (s, 1H), 7.53 (d, J = 7.8 Hz, 2H), 7.29 (t, J = 7.9 Hz, 2H), 7.08 (t, J = 7.4 Hz, 1H), 7.04 – 6.96 (m, 2H), 6.95 – 6.85 (m, 1H), 6.15 (d, J = 7.1 Hz, 1H), 6.00 (d, J = 4.1 Hz, 1H), 3.99 – 3.78 (m, 2H), 3.21 (t, J = 12.3 Hz, 1H), 3.00 – 2.88 (m, 1H), 2.70 – 2.58 (m, 1H), 1.71 (t, J = 12.2 Hz, 1H), 1.58 (app q, J = 11.9 Hz, 1H), 1.43 (s, 9H). ^{13}C NMR (100 MHz, C_3D_6O): δ = 163.7, 159.1 (d, $J_{F\text{-}C}$ = 235.4 Hz), 155.5, 146.5, 138.8, 133.8 (d, $J_{F\text{-}C}$ = 8.2 Hz), 129.6, 126.6 (q, $J_{F\text{-}C}$ = 282.1 Hz), 125.0, 120.3, 114.5 (d, $J_{F\text{-}C}$ = 23.4 Hz), 113.9 (d, $J_{F\text{-}C}$ = 8.4 Hz), 110.9 (d, $J_{F\text{-}C}$ = 23.3 Hz), 78.7, 67.8, 55.5 (d, $J_{F\text{-}C}$ = 23.2 Hz), 47.4, 44.2, 34.2, 33.7, 28.5. ^{19}F NMR (376 MHz, $CDCl_3$): δ = -71.6, -121.1. IR (thin film): ν_{max} (cm^{-1}) = 3392, 3297, 3033, 2979, 1686, 1601, 1561, 1483, 1241, 1174; HRMS (ESI) calcd for $C_{25}H_{27}F_4N_3O_3Na$ $[M+Na]^+$: 516.1881. Found: 516.1880.

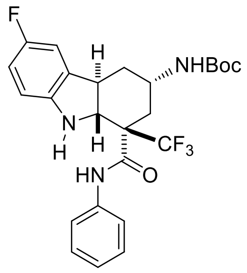

3k' (minor dr)

3k' (minor dr). White solid, 7.0 mg, 14% yield. ^{1}H NMR (400 MHz, C_3D_6O): δ = 11.23 (s, 1H), 7.53 (d, *J* = 7.8 Hz, 2H), 7.33 (t, *J* = 7.9 Hz, 2H), 7.12 (t, *J* = 7.4 Hz, 1H), 7.05 – 6.99 (m, 1H), 6.95 (d, *J* = 8.3 Hz, 1H), 6.90 (dt, *J* = 8.8, 2.4 Hz, 1H), 6.06 (d, *J* = 4.3 Hz, 1H), 5.97 (s, 1H), 4.16 – 4.07 (m, 1H), 3.90 (dd, *J* = 13.5, 4.7 Hz, 1H), 3.38 (t, *J* = 12.2 Hz, 1H), 2.98 (d, *J* = 14.6 Hz, 1H), 2.65 (d, *J* = 12.7 Hz, 1H), 1.97 (dd, *J* = 14.6, 5.2 Hz, 1H), 1.80 (dt, *J* = 13.2, 4.0 Hz, 1H), 1.41 (s, 9H). ^{13}C NMR (100 MHz, C_3D_6O): δ = 165.6, 159.2 (d, $J_{F\text{-}C}$ = 235.4 Hz), 155.6, 146.1, 138.6, 134.3 (d, $J_{F\text{-}C}$ = 8.2 Hz), 129.7, 126.7 (q, $J_{F\text{-}C}$ = 282.6 Hz), 125.4, 120.8, 114.5 (d, $J_{F\text{-}C}$ = 23.5 Hz), 113.9 (d, $J_{F\text{-}C}$ = 8.4 Hz), 110.9 (d, $J_{F\text{-}C}$ = 24.2 Hz), 78.8, 67.8, 55.6 (q, $J_{F\text{-}C}$ = 22.8 Hz), 46.5, 40.2, 33.0, 30.8, 28.5. ^{19}F NMR (376 MHz, $CDCl_3$): δ = -71.6, -121.1. IR (thin film): ν_{max} (cm^{-1}) = 3331, 3034, 2979, 1694, 1601, 1500, 1269, 1240, 1166; HRMS (ESI) calcd for $C_{25}H_{27}F_4N_3O_3Na$ $[M+Na]^+$: 516.1881. Found: 516.1897.

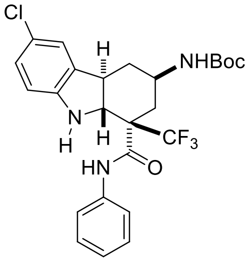

3l. White solid, 35.6 mg, 70% yield. ^{1}H NMR (400 MHz, C_3D_6O): δ = 10.68 (s, 1H), 7.57 – 7.50 (m, 2H), 7.35 – 7.26 (m, 2H), 7.21 (s, 1H), 7.18 – 7.13 (m, 1H), 7.12 – 7.06 (m, 1H), 7.01 (d, *J* = 8.3 Hz, 1H), 6.27 – 6.02 (m, 2H), 3.98 – 3.79 (m, 2H), 3.23 (t, *J* = 12.3 Hz, 1H), 2.95 – 2.86 (m, 1H), 2.69– 2.60 (m, 1H), 1.70 (t, *J* = 12.4 Hz, 1H), 1.58 (app q, *J* = 11.9 Hz, 1H), 1.43 (s, 9H). ^{13}C NMR (100 MHz, C_3D_6O): δ = 163.6, 155.5, 149.4, 138.7, 134.0, 129.6, 128.3, 126.5 (q, $J_{F\text{-}C}$ = 282.1 Hz), 126.4, 125.0, 123.6, 120.4, 114.4, 78.7, 67.6, 55.5 (q, $J_{F\text{-}C}$ = 23.3 Hz), 47.4, 44.1, 34.1, 33.7, 28.5. ^{19}F NMR (376 MHz, $CDCl_3$): δ = -71.6. IR (thin film): ν_{max} (cm^{-1}) = 3343, 3035, 2979, 1694, 1601, 1500, 1242, 1167; HRMS (ESI) calcd for $C_{25}H_{27}ClF_3N_3O_3Na$ $[M+Na]^+$: 532.1585. Found: 532.1585.

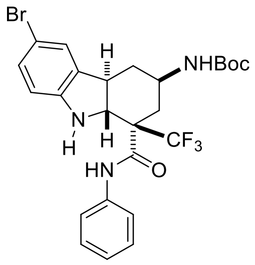

3m. White solid, 38.4 mg, 69% yield. ^{1}H NMR (400 MHz, C_3D_6O): δ = 10.65 (s, 1H), 7.53 (d, *J* = 7.8 Hz, 2H), 7.36 – 7.23 (m, 4H), 7.08 (t, *J* = 7.4 Hz, 1H), 6.97 (d, *J* = 8.3 Hz, 1H), 6.27 – 6.01 (m, 2H), 3.99 – 3.77 (m, 2H), 3.23 (t, *J* = 12.5 Hz, 1H), 2.96 – 2.88 (m, 1H), 2.64 (d, *J* = 11.9 Hz, 1H), 1.70 (t, *J* = 12.3 Hz, 1H), 1.58 (app q, *J* = 11.9 Hz, 1H), 1.43 (s, 9H). ^{13}C NMR (100 MHz, C_3D_6O): δ = 163.6, 155.5, 149.9, 138.7, 134.5, 131.2, 129.6, 126.54 (q, $J_{F\text{-}C}$ = 282.1 Hz), 126.51, 125.1, 120.4, 115.0, 113.6, 78.7, 67.6, 55.5 (q, $J_{F\text{-}C}$ = 23.3 Hz), 47.4, 44.1, 34.1, 33.7, 28.5. ^{19}F NMR (376 MHz, $CDCl_3$): δ = -71.6. IR (thin film): ν_{max} (cm^{-1}) = 3339, 3036, 2979, 1694, 1601, 1501, 1243, 1167; HRMS (ESI) calcd for $C_{25}H_{27}BrF_3N_3O_3Na$ $[M+Na]^+$: 576.1080. Found: 576.1077.

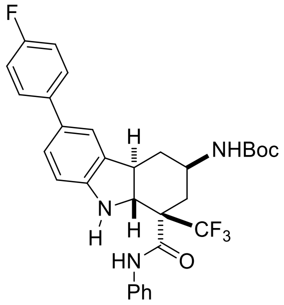

3n. White solid, 31.1 mg, 55% yield. ^{1}H NMR (400 MHz, C_3D_6O): δ = 10.82 (s, 1H), 7.71 – 7.59 (m, 2H), 7.55 (d, *J* = 7.7 Hz, 2H), 7.48 (s, 1H), 7.43 (d, *J* = 8.1 Hz, 1H), 7.30 (t, *J* = 7.9 Hz, 2H), 7.23 – 7.13 (m, 2H), 7.13 – 7.03 (m, 2H), 6.18 – 6.13 (m, 2H), 4.05 – 3.83 (m, 2H), 3.24 (t, *J* = 11.7 Hz, 1H), 3.05 – 2.89 (m, 1H), 2.79 – 2.66 (m, 1H), 1.74 (t, *J* = 12.4 Hz, 1H), 1.61 (app q, *J* = 11.9 Hz, 1H), 1.44 (s, 9H). ^{13}C NMR (100 MHz, C_3D_6O): δ = 163.8, 162.7 (d, $J_{F\text{-}C}$ = 242.1 Hz), 155.6, 150.1, 138.9, 138.2 (d, $J_{F\text{-}C}$ = 3.2 Hz), 134.2, 132.7, 129.7, 129.0 (d, $J_{F\text{-}C}$ = 8.0 Hz), 127.3, 126.7 (q, $J_{F\text{-}C}$ = 281.9 Hz), 125.1, 122.0, 120.4, 116.1 (d, $J_{F\text{-}C}$ = 21.4 Hz), 113.6, 78.7, 67.7, 55.6 (q, $J_{F\text{-}C}$ = 23.2 Hz), 47.6, 44.1, 34.5, 33.8, 28.5. ^{19}F NMR (376 MHz, $CDCl_3$): δ = -71.6, -116.2. IR (thin film): ν_{max} (cm^{-1}) = 3298, 3035, 2979, 1695, 1602, 1483, 1246, 1167; HRMS (ESI) calcd for $C_{31}H_{31}F_4N_3O_3Na$ $[M+Na]^+$: 592.2194. Found: 592.2195.

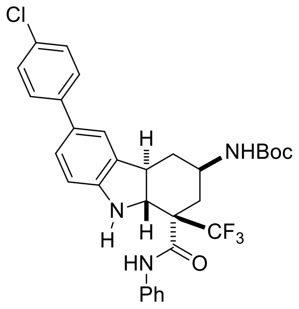

3o. White solid, 37.0 mg, 63% yield. ^{1}H NMR (400 MHz, C_3D_6O): δ = 10.79 (s, 1H), 7.62 (d, *J* = 8.5 Hz, 2H), 7.55 (d, *J* = 7.7 Hz, 2H), 7.50 (s, 1H), 7.45 (d, *J* = 8.1 Hz, 1H), 7.43 – 7.38 (m, 2H), 7.29 (t, *J* = 7.9 Hz, 2H), 7.12 (d, *J* = 8.1 Hz, 1H), 7.07 (t, *J* = 7.4 Hz, 1H), 6.29 – 6.07 (m, 2H), 4.05 – 3.81 (m, 2H), 3.24 (t, *J* = 11.9 Hz, 1H), 3.03 – 2.91 (m, 1H), 2.81 – 2.68 (m, 1H), 1.74 (t, *J* = 12.4 Hz, 1H), 1.62 (app q, *J* = 11.9 Hz, 1H), 1.44 (s, 9H). ^{13}C NMR (100 MHz, C_3D_6O): δ = 163.7, 155.6, 150.4, 140.6, 138.8, 133.7, 132.73, 132.72, 129.6, 129.4, 128.7, 127.3, 126.6 (q, $J_{F\text{-}C}$ = 282.0 Hz), 125.0, 122.0, 120.3, 113.6, 78.7, 67.6, 55.6 (q, $J_{F\text{-}C}$ = 23.3 Hz), 47.6, 44.1, 34.4, 33.7, 28.5. ^{19}F NMR (376 MHz, $CDCl_3$): δ = -71.6. IR (thin film): ν_{max} (cm^{-1}) = 3338, 3035, 2978, 1694, 1601, 1500, 1477, 1245, 1167; HRMS (ESI) calcd for $C_{31}H_{31}ClF_3N_3O_3Na$ $[M+Na]^+$: 608.1898. Found: 608.1897.

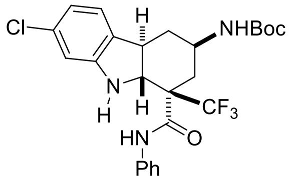

3p. White solid, 35.5 mg, 70% yield. ^{1}H NMR (400 MHz, C_3D_6O): δ = 10.58 (s, 1H), 7.55 (d, *J* = 7.8 Hz, 2H), 7.30 (t, *J* = 7.9 Hz, 2H), 7.17 (d, *J* = 7.8 Hz, 1H), 7.08 (t, *J* = 7.4 Hz, 1H), 7.04 (d, *J* = 1.6 Hz, 1H), 6.90 (dd, *J* = 7.9, 1.6 Hz, 1H), 6.22 (d, *J* = 3.8 Hz, 1H), 6.14 (d, *J* = 7.1 Hz, 1H), 4.01 – 3.73 (m, 2H), 3.16 (t, *J* = 11.8 Hz, 1H), 2.99 – 2.87 (m, 1H), 2.70 – 2.54 (m, 1H), 1.69 (t, *J* = 12.3 Hz, 1H), 1.56 (app q, *J* = 11.9 Hz, 1H), 1.43 (s, 9H). ^{13}C NMR (100 MHz, C_3D_6O): δ = 163.5, 155.5, 152.1, 138.7, 133.5, 130.8, 129.6, 126.5 (q, $J_{F\text{-}C}$ = 282.0 Hz), 125.1, 124.4, 121.6, 120.4, 113.5, 78.7, 67.6, 55.5 (q, $J_{F\text{-}C}$ = 23.2 Hz), 47.5, 43.6, 34.2, 33.7, 28.5. ^{19}F NMR (376 MHz, $CDCl_3$): δ = -71.6. IR (thin film): ν_{max} (cm^{-1}) = 3268, 3035, 2978, 1695, 1602, 1500, 1269, 1168; HRMS (ESI) calcd for $C_{25}H_{27}ClF_3N_3O_3Na$ $[M+Na]^+$: 532.1585. Found: 532.1585.

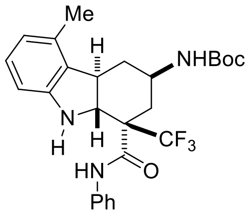

3q. White solid, 24.5 mg, 50% yield. ^{1}H NMR (400 MHz, C_3D_6O): δ = 10.86 (s, 1H), 7.52 (d, *J* = 7.7 Hz, 2H), 7.29 (t, *J* = 7.9 Hz, 2H), 7.07 (t, *J* = 7.4 Hz, 1H), 7.02 (t, *J* = 7.7 Hz, 1H), 6.85 (d, *J* = 7.8 Hz, 1H), 6.64 (d, *J* = 7.6 Hz, 1H), 6.11 (d, *J* = 6.0 Hz, 1H), 5.95 (d, *J* = 4.3 Hz, 1H), 3.98 – 3.86 (m, 1H), 3.83 (dd, *J* = 13.3, 4.6 Hz, 1H), 3.30 (t, *J* = 12.0 Hz, 1H), 3.01 – 2.85 (m, 2H), 2.30 (s, 3H), 1.80 – 1.59 (m, 2H), 1.43 (s, 9H). ^{13}C NMR (100 MHz, C_3D_6O): δ = 163.9, 155.5, 150.4, 138.9, 135.3, 129.7, 129.1, 128.5, 126.7 (q, $J_{F\text{-}C}$ = 282.1 Hz), 125.0, 124.3, 120.3, 111.1, 78.7, 67.1, 55.2 (q, $J_{F\text{-}C}$ = 23.1 Hz), 47.7, 44.7, 36.1, 33.5, 28.5, 19.2. ^{19}F NMR (376 MHz, $CDCl_3$): δ = -71.5. IR (thin film): ν_{max} (cm^{-1}) = 3331, 3032, 2977, 1697, 1600, 1500, 1254, 1169; HRMS (ESI) calcd for $C_{26}H_{30}F_3N_3O_3Na$ $[M+Na]^+$: 512.2131. Found: 512.2144.

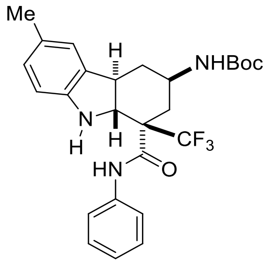

3r. White solid, 25.8 mg, 53% yield. ^{1}H NMR (400 MHz, C_3D_6O): δ = 10.97 (s, 1H), 7.53 (d, *J* = 7.6 Hz, 2H), 7.29 (t, *J* = 7.9 Hz, 2H), 7.07 (t, *J* = 7.4 Hz, 1H), 7.02 – 6.87 (m, 3H), 6.14 (d, *J* = 7.3 Hz, 1H), 5.88 (d, *J* = 4.4 Hz, 1H), 3.88 (d, *J* = 7.5 Hz, 1H), 3.79 (dd, *J* = 13.3, 4.6 Hz, 1H), 3.14 (t, *J* = 11.7 Hz, 1H), 2.91 (dd, *J* = 12.9, 3.1 Hz, 1H), 2.64 – 2.61 (m, 1H), 2.25 (s, 3H), 1.69 (t, *J* = 12.3 Hz, 1H), 1.54 (app q, *J* = 11.9 Hz, 1H), 1.43 (s, 9H). ^{13}C NMR (100 MHz, C_3D_6O): δ = 163.9, 155.5, 148.0, 138.9, 131.9, 131.2, 129.6, 128.7, 126.7 (q, $J_{F\text{-}C}$ = 282.0 Hz), 124.9, 123.9, 120.3, 113.1, 78.7, 67.6, 47.6, 55.5 (q, $J_{F\text{-}C}$ = 23.2 Hz), 44.1, 34.4, 33.8, 28.5, 20.8. ^{19}F NMR (376 MHz, $CDCl_3$): δ = -71.7. IR (thin film): ν_{max} (cm^{-1}) = 3342, 3030, 2977, 1694, 1601, 1500, 1240, 1168; HRMS (ESI) calcd for $C_{26}H_{30}F_3N_3O_3Na$ $[M+Na]^+$: 512.2131. Found: 512.2130.

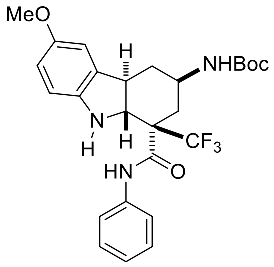

3s. White solid, 32.9 mg, 65% yield. ^{1}H NMR (400 MHz, C_3D_6O): δ = 11.07 (s, 1H), 7.53 (d, *J* = 7.7 Hz, 2H), 7.29 (t, *J* = 7.9 Hz, 2H), 7.07 (t, *J* = 7.4 Hz, 1H), 6.94 (d, *J* = 8.5 Hz, 1H), 6.83 (s, 1H), 6.71 (dd, *J* = 8.5, 2.0 Hz, 1H), 6.13 (d, *J* = 7.3 Hz, 1H), 5.77 (d, *J* = 4.6 Hz, 1H), 3.95 – 3.83 (m, 1H), 3.80 (dd, *J* = 13.4, 4.8 Hz, 1H), 3.73 (s, 3H), 3.15 (t, *J* = 11.9 Hz, 1H), 2.98 – 2.87 (m, 1H), 2.69 – 2.56 (m, 1H), 1.70 (t, *J* = 12.3 Hz, 1H), 1.54 (app q, *J* = 12.1 Hz, 1H), 1.43 (s, 9H). ^{13}C NMR (100 MHz, C_3D_6O): δ = 163.9, 156.1, 155.5, 143.6, 138.9, 133.1, 129.6, 126.7 (q, $J_{F\text{-}C}$ = 282.1 Hz), 124.9, 120.3, 113.8, 113.6, 109.8, 78.7, 67.7, 55.8, 55.5 (q, $J_{F\text{-}C}$ = 23.2 Hz), 47.5, 44.3, 34.4, 33.7, 28.5. ^{19}F NMR (376 MHz, $CDCl_3$): δ = -71.7. IR (thin film): ν_{max} (cm^{-1}) = 3331, 3032, 2978, 1694, 1601, 1488, 1268, 1166; HRMS (ESI) calcd for $C_{26}H_{30}F_3N_3O_4Na$ $[M+Na]^+$: 528.2081. Found: 528.2079.

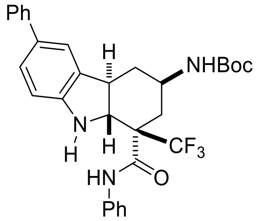

In lieu of the standard conditions, the reaction was performed at 40 °C. **3t**. White solid,30.0 mg, 54% yield. ^{1}H NMR (400 MHz, C_3D_6O): δ = 10.84 (s, 1H), 7.67 – 7.51 (m, 4H), 7.49 (s, 1H), 7.45 (d, *J* = 8.2 Hz, 1H), 7.40 (t, *J* = 7.7 Hz, 2H), 7.35 – 7.21 (m, 3H), 7.17 – 6.99 (m, 2H), 6.36 – 5.96 (m, 2H), 4.12 – 3.75 (m, 2H), 3.25 (t, *J* = 11.9 Hz, 1H), 3.09 – 2.89 (m, 1H), 2.81 – 2.67 (m, 1H), 1.74 (t, *J* = 12.3 Hz, 1H), 1.62 (app q, *J* = 11.9 Hz, 1H), 1.44 (s, 9H). ^{13}C NMR (100 MHz, C_3D_6O): δ = 163.8, 155.6, 150.1, 141.9, 138.9, 135.3, 132.6, 129.7, 129.4, 127.33, 127.26, 127.19, 126.7 (q, $J_{F\text{-}C}$ = 282.0 Hz), 125.0, 122.0, 120.4, 113.6, 78.7, 67.6, 55.6 (q, $J_{F\text{-}C}$ = 23.1 Hz), 47.6, 44.1, 34.4, 33.8, 28.5. ^{19}F NMR (376 MHz, $CDCl_3$): δ = -71.6. IR (thin film): ν_{max} (cm^{-1}) = 3333, 3034, 2979, 1694, 1601, 1501, 1245, 1167; HRMS (ESI) calcd for $C_{31}H_{32}F_3N_3O_3Na$ $[M+Na]^+$: 574.2288. Found: 574.2288.

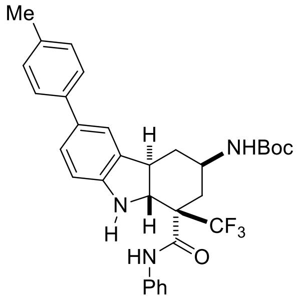

3u. White solid, 23.2 mg, 41% yield. ^{1}H NMR (400 MHz, CD_2Cl_2): δ = 10.47 (s, 1H), 7.52 (d, *J* = 7.9 Hz, 2H), 7.47 – 7.37 (m, 3H), 7.34 (s, 1H), 7.29 (t, *J* = 7.8 Hz, 2H), 7.22 (d, *J* = 7.9 Hz, 2H), 7.10 (t, *J* = 7.3 Hz, 1H), 7.01 (d, *J* = 8.1 Hz, 1H), 4.78 – 4.50 (m, 2H), 4.00 – 3.69 (m, 2H), 3.25 (t, *J* = 12.1 Hz, 1H), 3.11 – 2.90 (m, 1H), 2.78 – 2.63 (m, 1H), 2.37 (s, 3H), 1.57 – 1.36 (m, 11H). ^{13}C NMR (100 MHz, CD_2Cl_2): δ = 163.4, 155.4, 148.5, 138.6, 138.3, 137.3, 135.9, 132.1, 130.0, 129.5, 127.3, 127.0, 126.3 (q, $J_{F\text{-}C}$ = 282.5 Hz), 125.2, 122.0, 120.7, 113.1, 80.0, 67.7, 55.3 (q, $J_{F\text{-}C}$ = 23.4 Hz), 47.5, 43.6, 34.6, 34.2, 28.7, 21.3. ^{19}F NMR (376 MHz, $CDCl_3$): δ = -71.6. IR (thin film): ν_{max} (cm^{-1}) = 3335, 3030, 2978, 1698, 1601, 1500, 1483, 1245, 1166; HRMS (ESI) calcd for $C_{32}H_{34}F_3N_3O_3Na$ $[M+Na]^+$: 588.2444. Found: 588.2445.

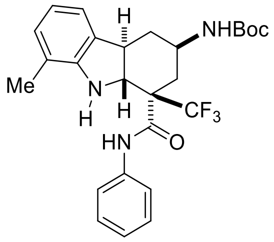

3v. White solid, 24.9 mg, 51% yield. ^{1}H NMR (400 MHz, C_3D_6O): δ = 10.93 (s, 1H), 7.55 (d, *J* = 8.0 Hz, 2H), 7.29 (t, *J* = 7.8 Hz, 2H), 7.07 (t, *J* = 7.3 Hz, 1H), 7.00 (t, *J* = 7.3 Hz, 2H), 6.83 (t, *J* = 7.4 Hz, 1H), 6.11 (d, *J* = 5.8 Hz, 1H), 5.61 (d, *J* = 3.9 Hz, 1H), 3.99 – 3.89 (m, 1H), 3.83 (dd, *J* = 13.5, 4.9 Hz, 1H), 3.15 (t, *J* = 12.4 Hz, 1H), 2.98 – 2.87 (m, 1H), 2.68 – 2.58 (m, 1H), 2.33 (s, 3H), 1.70 (t, *J* = 12.3 Hz, 1H), 1.56 (app q, *J* = 11.9 Hz, 1H), 1.43 (s, 9H). ^{13}C NMR (100 MHz, C_3D_6O): δ = 163.9, 155.6, 148.7, 139.0, 131.3, 129.8, 129.7, 126.7 (q, $J_{F\text{-}C}$ = 282.1 Hz), 125.0, 123.0, 122.3, 120.7, 120.3, 78.7, 67.2, 55.7 (q, $J_{F\text{-}C}$ = 23.0 Hz), 47.6, 44.4, 34.6, 33.9, 28.5, 16.7. ^{19}F NMR (376 MHz, $CDCl_3$): δ = -71.5. IR (thin film): ν_{max} (cm^{-1}) = 3350, 3032, 2978, 1697, 1601, 1500, 1246, 1170; HRMS (ESI) calcd for $C_{26}H_{30}F_3N_3O_3Na$ $[M+Na]^+$: 512.2131. Found: 512.2131.

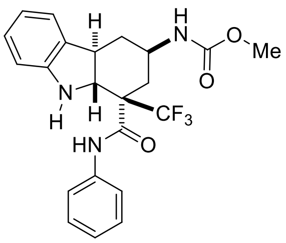

3w. White solid, 30.1 mg, 70% yield. ^{1}H NMR (400 MHz, C_3D_6O): δ = 10.86 (s, 1H), 7.56 – 7.50 (m, 2H), 7.34 – 7.25 (m, 2H), 7.19 – 7.11 (m, 2H), 7.10 – 7.05 (m, 1H), 7.02 (d, J = 7.7 Hz, 1H), 6.88 (t, J = 7.4 Hz, 1H), 6.41 (d, J = 6.6 Hz, 1H), 6.03 (d, J = 4.3 Hz, 1H), 4.00 – 3.87 (m, 1H), 3.83 (dd, J = 13.4, 4.5 Hz, 1H), 3.60 (s, 3H), 3.18 (t, J = 11.6 Hz, 1H), 2.96 – 2.91 (m, 1H), 2.69 – 2.60 (m, 1H), 1.72 (t, J = 12.4 Hz, 1H), 1.58 (app q, J = 11.9 Hz, 1H). ^{13}C NMR (100 MHz, C_3D_6O): δ = 163.7, 156.6, 150.4, 138.7, 131.6, 129.5, 128.4, 126.5 (q, $J_{F\text{-}C}$ = 281.9 Hz), 124.9, 123.1, 121.9, 120.2, 113.2, 67.3, 55.4 (q, $J_{F\text{-}C}$ = 23.2 Hz), 51.6, 48.0, 43.9, 34.2, 33.6. ^{19}F NMR (376 MHz, $CDCl_3$): δ = -71.6. IR (thin film): ν_{max} (cm^{-1}) = 3322, 3033, 2953, 1694, 1601, 1557, 1269, 1236, 1167; HRMS (ESI) calcd for $C_{22}H_{22}F_3N_3O_3Na$ [M+Na]$^+$: 456.1505. Found: 456.1505.

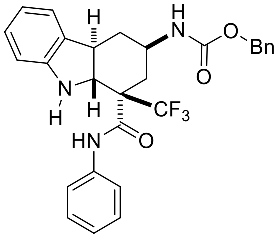

3x. White solid, 35.2 mg, 69% yield. ^{1}H NMR (400 MHz, C_3D_6O): δ = 10.86 (s, 1H), 7.54 (d, J = 7.8 Hz, 2H), 7.46 – 7.33 (m, 4H), 7.33 – 7.25 (m, 3H), 7.20 – 7.11 (m, 2H), 7.08 (t, J = 7.4 Hz, 1H), 7.03 (d, J = 7.7 Hz, 1H), 6.89 (t, J = 7.4 Hz, 1H), 6.53 (d, J = 7.6 Hz, 1H), 6.02 (d, J = 4.3 Hz, 1H), 5.10 (s, 2H), 4.06 – 3.90 (m, 1H), 3.84 (dd, J = 13.4, 4.5 Hz, 1H), 3.19 (t, J = 11.8 Hz, 1H), 2.98 – 2.94 (m, 1H), 2.73 – 2.61 (m, 1H), 1.74 (t, J = 12.4 Hz, 1H), 1.60 (app q, J = 11.9 Hz, 1H). ^{13}C NMR (100 MHz, C_3D_6O): δ = 163.7, 156.1, 150.4, 138.7, 138.1, 131.6, 129.6, 129.0, 128.5, 128.4, 126.5 (q, $J_{F\text{-}C}$ = 282.1 Hz), 125.0, 123.2, 121.9, 120.3, 113.2, 67.3, 66.4, 55.4 (q, $J_{F\text{-}C}$ = 23.2 Hz), 48.0, 43.9, 34.2, 33.7. ^{19}F NMR (376 MHz, $CDCl_3$): δ = -71.6. IR (thin film): ν_{max} (cm^{-1}) = 3331, 3034, 2945, 1694, 1601, 1558, 1269, 1235, 1166; HRMS (ESI) calcd for $C_{28}H_{26}F_3N_3O_3Na$ [M+Na]$^+$: 532.1818. Found: 532.1817.

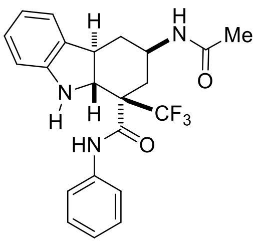

3y. White solid, 18.7 mg, 45% yield. ^{1}H NMR (400 MHz, C_3D_6O): δ = 10.86 (s, 1H), 7.56 – 7.49 (m, 2H), 7.32 – 7.25 (m, 2H), 7.21 (d, *J* = 7.2 Hz, 1H), 7.14 (t, *J* = 8.2 Hz, 2H), 7.07 (t, *J* = 7.4 Hz, 1H), 7.02 (d, *J* = 7.7 Hz, 1H), 6.88 (t, *J* = 7.4 Hz, 1H), 6.04 (d, *J* = 4.2 Hz, 1H), 4.22 – 4.09 (m, 1H), 3.83 (dd, *J* = 13.4, 4.5 Hz, 1H), 3.18 (t, *J* = 11.5 Hz, 1H), 2.89 – 2.84 (m, 1H), 2.68 – 2.59 (m, 1H), 1.90 (s, 3H), 1.66 (t, *J* = 12.4 Hz, 1H), 1.50 (app q, *J* = 11.9 Hz, 1H). ^{13}C NMR (100 MHz, C_3D_6O): δ = 169.1, 163.7, 150.4, 138.8, 131.7, 129.6, 128.5, 126.6 (q, J_{F-C} = 282.1 Hz), 125.0, 123.2, 122.0, 120.3, 113.3, 67.4, 55.5 (q, J_{F-C} = 23.2 Hz), 46.1, 44.0, 34.1, 33.5, 22.9. ^{19}F NMR (376 MHz, $CDCl_3$): δ = -71.6. IR (thin film): ν_{max} (cm^{-1}) = 3262, 3034, 2936, 1662, 1601, 1557, 1270, 1166; HRMS (ESI) calcd for $C_{22}H_{22}F_3N_3O_2Na$ [M+Na]$^+$: 440.1556. Found: 440.1557.

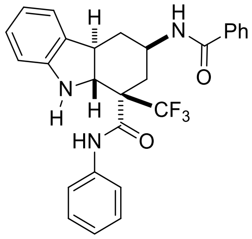

3z. White solid, 22.0 mg, 46% yield. ^{1}H NMR (400 MHz, C_3D_6O): δ = 10.90 (s, 1H), 8.01 – 7.88 (m, 2H), 7.80 (d, *J* = 8.1 Hz, 1H), 7.60 – 7.41 (m, 5H), 7.37 – 7.26 (m, 2H), 7.23 – 6.99 (m, 4H), 6.95 – 6.85 (m, 1H), 6.05 (d, *J* = 4.3 Hz, 1H), 4.53 – 4.35 (m, 1H), 3.88 (dd, *J* = 13.4, 4.5 Hz, 1H), 3.26 (t, *J* = 11.4 Hz, 1H), 3.04 – 2.96 (m, 1H), 2.82 – 2.71 (m, 1H), 1.93 (t, *J* = 12.4 Hz, 1H), 1.69 (app q, *J* = 11.9 Hz, 1H). ^{13}C NMR (100 MHz, C_3D_6O): δ = 166.5, 163.8, 150.4, 138.8, 135.6, 131.74, 131.66, 129.6, 128.9, 128.5, 127.9, 126.6 (q, J_{F-C} = 282.1 Hz), 125.0, 123.2, 122.0, 120.3, 113.2, 67.4, 55.6 (q, J_{F-C} = 23.3 Hz), 46.7, 44.0, 34.0, 33.2. ^{19}F NMR (376 MHz, $CDCl_3$): δ = -71.6. IR (thin film): ν_{max} (cm^{-1}) = 3323, 3033, 2936, 1691, 1641, 1549, 1489, 1271, 1195, 1166; HRMS (ESI) calcd for $C_{27}H_{24}F_3N_3O_2Na$ [M+Na]$^+$: 502.1713. Found: 502.1713.

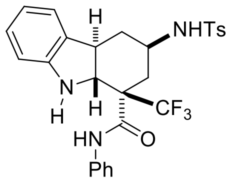

3aa. White solid, 25.3 mg, 48% yield. ^{1}H NMR (400 MHz, C_3D_6O): δ = 10.73 (s, 1H), 7.87 (d, *J* = 8.1 Hz, 2H), 7.50 (d, *J* = 8.1 Hz, 2H), 7.42 (d, *J* = 8.0 Hz, 2H), 7.30 (t, *J* = 7.8 Hz, 2H), 7.16 – 7.03 (m, 3H), 6.99 (d, *J* = 7.8 Hz, 1H), 6.86 (t, *J* = 7.4 Hz, 1H), 6.68 (d, *J* = 7.9 Hz, 1H), 6.00 (d, *J* = 4.0 Hz, 1H), 3.77 (dd, *J* = 13.4, 4.5 Hz, 1H), 3.73 – 3.61 (m, 1H), 3.11 (t, *J* = 12.2 Hz, 1H), 2.86 – 2.79 (m, 1H), 2.59 – 2.48 (m, 1H), 2.43 (s, 3H), 1.66 – 1.51 (m, 2H). ^{13}C NMR (100 MHz, C_3D_6O): δ = 163.5, 150.2, 143.5, 140.1, 138.6, 131.2, 130.1, 129.5, 128.4, 127.5, 126.3 (q, $J_{F\text{-}C}$ = 281.7 Hz), 124.9, 123.0, 121.9, 120.2, 113.2, 67.0, 55.4 (q, $J_{F\text{-}C}$ = 23.4 Hz), 50.5, 43.8, 35.1, 34.3, 21.1. ^{19}F NMR (376 MHz, $CDCl_3$): δ = -71.7. IR (thin film): ν_{max} (cm^{-1}) = 3264, 3034, 2977, 1693, 1601, 1558, 1448, 1194, 1162; HRMS (ESI) calcd for $C_{27}H_{26}F_3N_3O_3SNa$ [M+Na]$^+$: 552.1539. Found: 552.1538.

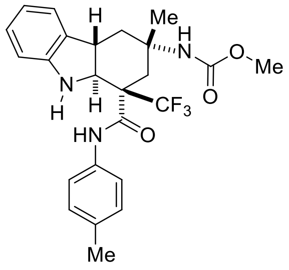

3ab (major dr)

3ab (major dr). White solid, 16.8 mg, 36% yield. ^{1}H NMR (400 MHz, C_3D_6O): δ = 11.29 (s, 1H), 7.40 (d, *J* = 8.4 Hz, 2H), 7.18 – 7.08 (m, 4H), 7.01 (d, *J* = 7.7 Hz, 1H), 6.90 (t, *J* = 7.4 Hz, 1H), 6.21 – 5.95 (m, 2H), 3.87 (dd, *J* = 13.2, 4.6 Hz, 1H), 3.48 (s, 3H), 3.40 – 3.28 (m, 2H), 2.94 (d, *J* = 14.4 Hz, 1H), 2.26 (s, 3H), 1.77 (d, *J* = 14.5 Hz, 1H), 1.55 (s, 3H), 1.54 – 1.46 (m, 1H). ^{13}C NMR (100 MHz, C_3D_6O): δ = 165.6, 155.3, 150.3, 135.9, 135.1, 132.1, 130.2, 128.5, 126.7 (q, $J_{F\text{-}C}$ = 282.9 Hz), 123.2, 122.1, 120.9, 113.4, 67.5, 55.3 (q, $J_{F\text{-}C}$ = 22.7 Hz), 54.1, 51.3, 41.1, 38.5, 36.6, 27.7, 20.7. ^{19}F NMR (376 MHz, $CDCl_3$): δ = -71.8. IR (thin film): ν_{max} (cm^{-1}) = 3370, 3334, 3029, 2962, 1726, 1674, 1613, 1515, 1261, 1174, 815; HRMS (ESI) calcd for $C_{24}H_{26}F_3N_3O_3Na$ [M+Na]$^+$: 484.1818. Found: 484.1820.

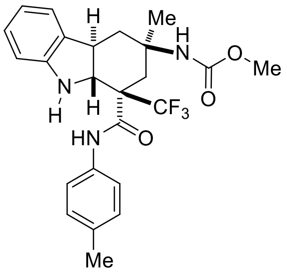

3ab' (minor dr)

3ab' (minor dr). White solid, 9.0 mg, 20% yield. ^{1}H NMR (400 MHz, C_3D_6O): δ = 11.10 (s, 1H), 7.46 – 7.37 (m, 2H), 7.21 – 7.06 (m, 4H), 7.03 (d, *J* = 7.7 Hz, 1H), 6.90 (t, *J* = 7.4 Hz, 1H), 6.31 (s, 1H), 6.02 (d, *J* = 4.1 Hz, 1H), 3.88 (dd, *J* = 13.5, 4.4 Hz, 1H), 3.55 (s, 3H), 3.46 – 3.33 (m, 1H), 2.96 (d, *J* = 14.6 Hz, 1H), 2.49 – 2.32 (m, 3H), 2.25 (s, 3H), 1.40 (s, 3H). ^{13}C NMR (100 MHz, C_3D_6O): δ = 164.2, 155.9, 150.5, 136.5, 134.5, 132.1, 130.1, 128.5, 126.9 (q, $J_{F\text{-}C}$ = 283.0 Hz), 123.2, 122.0, 120.4, 113.3, 67.5, 54.8 (q, $J_{F\text{-}C}$ = 22.4 Hz), 54.2, 51.3, 41.7, 38.2, 36.3, 27.2, 20.7. ^{19}F NMR (376 MHz, $CDCl_3$): δ = -71.7. IR (thin film): ν_{max} (cm^{-1}) = 3331, 3030, 2961, 1685, 1612, 1514, 1260, 1171; HRMS (ESI) calcd for $C_{24}H_{26}F_3N_3O_3Na$ $[M+Na]^+$: 484.1818. Found: 484.1828.

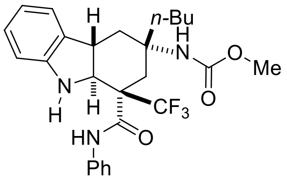

3ac (major dr)

3ac (major dr). White solid, 12.5 mg, 26% yield. ^{1}H NMR (400 MHz, C_3D_6O): δ = 11.39 (s, 1H), 7.52 (d, *J* = 7.7 Hz, 2H), 7.32 (t, *J* = 7.9 Hz, 2H), 7.23 – 7.08 (m, 3H), 7.03 (d, *J* = 7.6 Hz, 1H), 6.90 (t, *J* = 7.4 Hz, 1H), 6.08 (d, *J* = 4.2 Hz, 1H), 5.96 (s, 1H), 3.88 (dd, *J* = 13.4, 4.5 Hz, 1H), 3.48 (s, 3H), 3.39 (t, *J* = 12.7 Hz, 1H), 3.30 (d, *J* = 13.1 Hz, 1H), 3.05 (d, *J* = 14.4 Hz, 1H), 2.21 (t, *J* = 10.8 Hz, 1H), 1.75 – 1.61 (m, 2H), 1.52 (t, *J* = 12.7 Hz, 1H), 1.47 – 1.26 (m, 4H), 0.93 (t, *J* = 7.0 Hz, 3H). ^{13}C NMR (100 MHz, C_3D_6O): δ = 165.8, 155.1, 150.2, 138.5, 132.2, 129.7, 128.4, 126.8 (q, $J_{F\text{-}C}$ = 282.9 Hz), 125.5, 123.2, 122.1, 120.9, 113.4, 67.5, 56.6, 55.2 (q, $J_{F\text{-}C}$ = 22.6 Hz), 51.4, 40.9, 39.8, 36.3, 36.2, 26.2, 23.6, 14.2. ^{19}F NMR (376 MHz, $CDCl_3$): δ = -71.7. IR (thin film): ν_{max} (cm^{-1}) = 3376, 3034, 2957, 1725, 1676, 1519, 1238, 1168; HRMS (ESI) calcd for $C_{26}H_{30}F_3N_3O_3Na$ $[M+Na]^+$: 512.2131. Found: 512.2131.

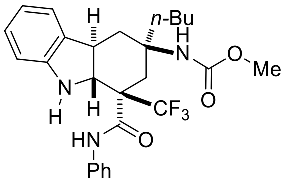

3ac' (minor dr)

3ac' (minor dr). White solid, 9.8 mg, 20% yield. ^{1}H NMR (400 MHz, C_3D_6O): δ = 11.29 (s, 1H), 7.53 (d, *J* = 7.7 Hz, 2H), 7.30 (t, *J* = 7.9 Hz, 2H), 7.21 – 7.11 (m, 2H), 7.11 – 6.99 (m, 2H), 6.90 (t, *J* = 7.4 Hz, 1H), 6.15 (s, 1H), 6.04 (d, *J* = 4.0 Hz, 1H), 3.90 (dd, *J* = 13.5, 4.3 Hz, 1H), 3.55 (s, 3H), 3.38 (t, *J* = 12.1 Hz, 1H), 3.14 (dd, *J* = 13.7, 1.2 Hz, 1H), 2.73 – 2.59 (m, 1H), 2.29 – 2.11 (m, 2H), 1.92 – 1.68 (m, 2H), 1.63 – 1.50 (m, 1H), 1.38 – 1.18 (m, 3H), 0.86 (t, *J* = 7.2 Hz, 3H). ^{13}C NMR (100 MHz, C_3D_6O): δ = 164.6, 155.9, 150.4, 139.0, 132.1, 129.7, 128.5, 126.9 (q, $J_{F\text{-}C}$ = 283.1 Hz), 125.0, 123.3, 122.0, 120.3, 113.3, 67.6, 56.8, 54.8 (q, $J_{F\text{-}C}$ = 22.2 Hz), 51.3, 41.2, 36.6, 35.1, 25.7, 23.5, 14.2. ^{19}F NMR (376 MHz, $CDCl_3$): δ = -71.7. IR (thin film): ν_{max} (cm^{-1}) = 3316, 3033, 2958, 1695, 1602, 1501, 1245, 1166; HRMS (ESI) calcd for $C_{26}H_{30}F_3N_3O_3Na$ $[M+Na]^+$: 512.2131. Found: 512.2147.

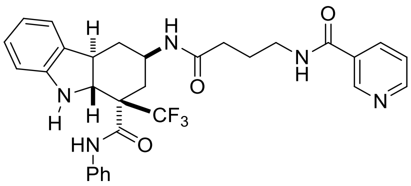

3ad. White solid, 28.1 mg, 50% yield. ^{1}H NMR (400 MHz, C_3D_6O): δ = 10.86 (s, 1H), 9.09 (d, *J* = 1.0 Hz, 1H), 8.67 (d, *J* = 4.5 Hz, 1H), 8.34 – 8.12 (m, 2H), 7.53 (d, *J* = 7.7 Hz, 2H), 7.49 – 7.43 (m, 1H), 7.43 – 7.35 (m, 1H), 7.30 (t, *J* = 7.9 Hz, 2H), 7.18 – 7.11 (m, 2H), 7.08 (t, *J* = 7.4 Hz, 1H), 7.05 – 6.96 (m, 1H), 6.88 (t, *J* = 7.4 Hz, 1H), 6.03 (d, *J* = 4.1 Hz, 1H), 4.25 – 4.14 (m, 1H), 3.83 (dd, *J* = 13.4, 4.5 Hz, 1H), 3.53 – 3.40 (m, 2H), 3.18 (t, *J* = 12.2 Hz, 1H), 2.97 – 2.87 (m, 1H), 2.71 – 2.54 (m, 1H), 2.33 (t, *J* = 6.8 Hz, 2H), 2.01 – 1.88 (m, 2H), 1.69 (t, *J* = 12.4 Hz, 1H), 1.52 (app q, *J* = 11.9 Hz, 1H). ^{13}C NMR (100 MHz, C_3D_6O): δ = 172.3, 165.7, 163.8, 152.5, 150.4, 149.3, 138.8, 135.3, 131.7, 131.1, 129.7, 128.5, 126.6 (q, $J_{F\text{-}C}$ = 281.9 Hz), 125.0, 124.0, 123.3, 122.0, 120.3, 113.3, 67.4, 55.5 (q, $J_{F\text{-}C}$ = 23.1 Hz), 46.2, 44.0, 40.1, 34.2, 34.1, 33.5, 25.9. ^{19}F NMR (376 MHz, $CDCl_3$): δ = -71.6. IR (thin film): ν_{max} (cm^{-1}) = 3269, 3060, 2933, 1652, 1601, 1550, 1448, 1270, 1195, 1165; HRMS (ESI) calcd for $C_{30}H_{30}F_3N_5O_3Na$ $[M+Na]^+$: 588.2193. Found: 588.2193.

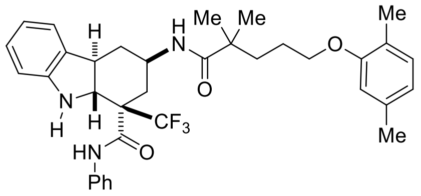

3ae. White solid, 23.9 mg, 39% yield. ^{1}H NMR (400 MHz, C_3D_6O): δ = 10.87 (s, 1H), 7.57 – 7.49 (m, 2H), 7.31 – 7.27 (m, 2H), 7.17 – 7.11 (m, 2H), 7.09 – 7.05 (m, 1H), 7.04 – 6.99 (m, 1H), 6.97 (d, *J* = 7.5 Hz, 1H), 6.92 (d, *J* = 8.0 Hz, 1H), 6.88 (t, *J* = 7.6 Hz, 1H), 6.72 (s, 1H), 6.61 (d, *J* = 7.4 Hz, 1H), 6.01 (d, *J* = 4.3 Hz, 1H), 4.34 – 4.16 (m, 1H), 4.00 – 3.92 (m, 2H), 3.87 – 3.76 (m, 1H), 3.18 (t, *J* = 11.6 Hz, 1H), 2.90 – 2.86 (m, 1H), 2.65 – 2.56 (m, 1H), 2.25 (s, 3H), 2.14 (s, 3H), 1.87 – 1.69 (m, 5H), 1.56 (app q, *J* = 11.9 Hz, 1H), 1.23 (s, 3H), 1.22 (s, 3H). ^{13}C NMR (100 MHz, C_3D_6O): δ = 176.6, 163.8, 157.8, 150.4, 138.9, 137.0, 131.8, 130.8, 129.6, 128.5, 126.7 (q, $J_{F\text{-}C}$ = 282.1 Hz), 125.0, 123.7, 123.3, 122.0, 121.3, 120.3, 113.3, 112.7, 68.7, 67.5, 55.6 (q, $J_{F\text{-}C}$ = 23.0 Hz), 46.3, 44.1, 42.2, 38.2, 34.0, 33.2, 25.8, 25.72, 25.71, 21.3, 15.9. ^{19}F NMR (376 MHz, $CDCl_3$): δ = -71.6. IR (thin film): ν_{max} (cm^{-1}) = 3347, 3265, 3034, 2955, 1693, 1647, 1602, 1501, 1268, 1164; HRMS (ESI) calcd for $C_{35}H_{40}F_3N_3O_3Na$ $[M+Na]^+$: 630.2914. Found: 630.2913.

2.2 Representative procedure for the stereoselective radical cascade reaction of tryptophan derivatives 7 and alkenes 2 under photoredox catalysis.

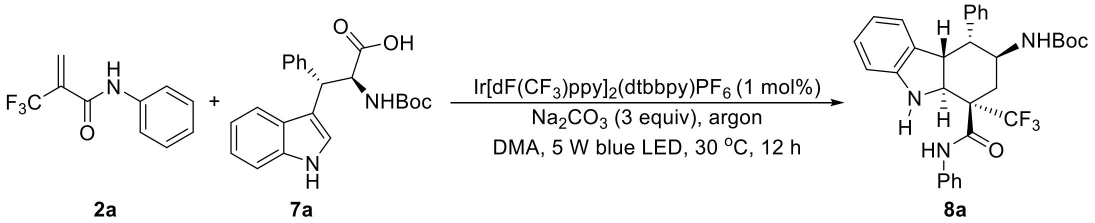

Preparation of compound 8a. To a Young Schlenk tube (10 mL) were added Ir[dF(CF_3)ppy]$_2$(dtbbpy)PF_6 (1.1 mg, 0.001 mmol, 1 mol%), **7a** (57.0 mg, 0.15 mmol, 1.5 equiv), **2a** (21.5 mg, 0.1 mmol, 1.0 equiv), Na_2CO_3 (31.8 mg, 0.3 mmol, 3.0 equiv), and N,N-dimethylacetamide (DMA, 2.0 mL). Subsequently, the reaction mixture was degassed through several freeze-pump-thaw cycles until no bubbles were released. The reaction mixture was stirred under argon at 30 °C, and irradiated by a 5 W blue LED lamp (λ = 450–460 nm, the tube was placed at approximately 2 cm away from the light source). After 12 h, the reaction mixture was passed through a short pad of celite and washed with ethyl acetate. The solvents were

evaporated under reduced pressure to give the crude mixture, which was purified by flash column chromatography on silica gel (petroleum ether/ethyl acetate = 40:1 to 20:1, *silica gel was soaked with a solution of petroleum ether and triethylamine (1000/1, v/v) before use*) to afford the title compound **8a** (major dr) as a white solid (21.0 mg, 38% yield), 96% ee [Daicel Chiralpak IC (0.46 cm x 25 cm), *n*-hexane/2-propanol = 95/5, v = 1.0 mL·min^{-1}, λ = 254 nm, t (minor) = 5.25 min, t (major) = 10.73 min]; $[\alpha]_D^{25}$ = -147.9 (c = 1.0, $CHCl_3$). ^{1}H NMR (400 MHz, C_3D_6O): δ = 11.52 (s, 1H), 7.63 – 7.46 (m, 4H), 7.33 (t, *J* = 7.9 Hz, 2H), 7.25 (t, *J* = 7.5 Hz, 2H), 7.21 – 7.07 (m, 4H), 7.02 (d, *J* = 7.7 Hz, 1H), 6.82 (t, *J* = 7.4 Hz, 1H), 6.35 (d, *J* = 2.1 Hz, 1H), 6.10 (d, *J* = 5.0 Hz, 1H), 4.60 (dd, *J* = 14.1, 5.3 Hz, 1H), 4.41 – 4.25 (m, 1H), 4.08 – 3.98 (m, 1H), 3.90 (dd, *J* = 14.0, 4.8 Hz, 1H), 2.94 (dd, *J* = 15.2, 1.9 Hz, 1H), 2.20 (dd, *J* = 15.2, 5.4 Hz, 1H), 1.43 (s, 9H). ^{13}C NMR (100 MHz, C_3D_6O): δ = 166.1, 155.9, 149.5, 139.8, 138.4, 130.3, 130.2, 129.7, 128.8, 128.5, 127.1, 126.8 (q, $J_{F\text{-}C}$ = 282.6 Hz), 125.5, 124.8, 122.1, 120.8, 113.3, 79.2, 60.3, 56.0 (q, $J_{F\text{-}C}$ = 22.9 Hz), 53.1, 45.9, 44.2, 29.0, 28.5. ^{19}F NMR (376 MHz, $CDCl_3$): δ = -71.6. IR (thin film): ν_{max} (cm^{-1}) = 3384, 2976, 1679, 1602, 1560, 1498, 1245, 1166; HRMS (ESI) calcd for $C_{31}H_{32}F_3N_3O_3Na$ [M+Na]$^+$: 574.2288. Found: 574.2287.

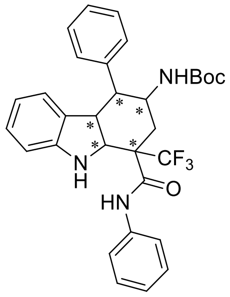

8a' (minor dr-1)

8a' (minor dr-1). White solid, 5.0 mg, 9% yield. ^{1}H NMR (500 MHz, C_3D_6O): δ = 11.14 (s, 1H), 7.56 (d, *J* = 7.7 Hz, 2H), 7.47 (d, *J* = 7.4 Hz, 2H), 7.35 – 7.26 (m, 4H), 7.20 (t, *J* = 7.4 Hz, 1H), 7.17 – 7.13 (m, 1H), 7.09 (t, *J* = 7.4 Hz, 1H), 7.00 (t, *J* = 7.7 Hz, 1H), 6.91 (d, *J* = 7.8 Hz, 1H), 6.75 (t, *J* = 7.4 Hz, 1H), 5.97 (d, *J* = 4.7 Hz, 1H), 5.73 (d, *J* = 7.3 Hz, 1H), 4.66 (dd, *J* = 14.3, 4.8 Hz, 1H), 4.38 – 4.25 (m, 2H), 3.75 (dd, *J* = 14.3, 3.8 Hz, 1H), 2.98 (dd, *J* = 13.6, 4.1 Hz, 1H), 2.30 (t, *J* = 13.1 Hz, 1H), 1.33 (s, 9H). ^{13}C NMR (125 MHz, C_3D_6O): δ = 163.7, 155.5, 150.2, 138.9, 138.2, 132.3, 129.8, 129.7, 129.0, 128.3, 127.7, 126.9 (q, $J_{F\text{-}C}$ = 281.9 Hz), 125.1, 124.9, 121.9, 120.4, 113.2, 78.8, 60.9, 55.7 (q, $J_{F\text{-}C}$ = 23.4 Hz), 50.4, 48.1, 44.9, 30.8, 28.4. ^{19}F NMR (376 MHz, $CDCl_3$): δ = -71.5. IR (thin film): ν_{max} (cm^{-1}) = 3435, 3321, 3032,

2977, 1694, 1601, 1559, 1499, 1253, 1172; HRMS (ESI) calcd for $C_{31}H_{32}F_3N_3O_3Na$ $[M+Na]^+$: 574.2288. Found: 574.2317.

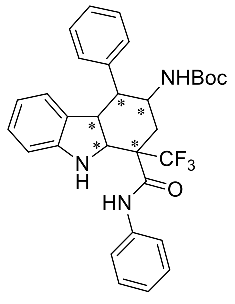

8a" (minor dr-2)

8a” (minor dr-2). White solid, 6.0 mg, 11% yield. ^{1}H NMR (400 MHz, C_3D_6O): δ = 10.93 (s, 1H), 7.59 (d, *J* = 7.8 Hz, 2H), 7.38 – 7.23 (m, 7H), 7.11 (t, *J* = 7.4 Hz, 1H), 7.07 – 6.96 (m, 2H), 6.53 (t, *J* = 7.3 Hz, 1H), 6.09 (d, *J* = 4.3 Hz, 1H), 6.00 (d, *J* = 8.3 Hz, 1H), 5.92 (d, *J* = 7.4 Hz, 1H), 4.14 – 3.98 (m, 2H), 3.61 (t, *J* = 12.3 Hz, 1H), 3.20 (t, *J* = 11.0 Hz, 1H), 3.00 (dd, *J* = 12.9, 3.9 Hz, 1H), 2.03 – 1.93 (m, 1H), 1.22 (s, 9H). ^{13}C NMR (100 MHz, C_3D_6O): δ = 163.9, 155.5, 150.7, 141.0, 139.0, 130.9, 129.8, 129.2, 129.1, 128.5, 127.8, 126.8 (q, $J_{F\text{-}C}$ = 281.9 Hz), 125.1, 124.6, 121.6, 120.4, 113.4, 78.4, 66.9, 55.5 (q, $J_{F\text{-}C}$ = 23.0 Hz), 53.8, 52.7, 48.5, 34.2, 28.3. ^{19}F NMR (376 MHz, $CDCl_3$): δ = -71.2. IR (thin film): ν_{max} (cm^{-1}) = 3280, 3031, 2978, 1696, 1601, 1558, 1499, 1248, 1176; HRMS (ESI) calcd for $C_{31}H_{32}F_3N_3O_3Na$ $[M+Na]^+$: 574.2288. Found: 574.2313.

The following compounds **8b–8g** were prepared analogously.

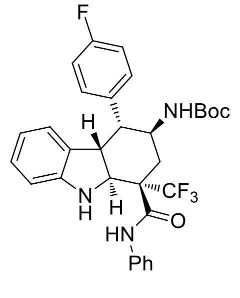

8b (major dr). White solid, 17.0 mg, 30% yield, 91% ee [Daicel Chiralpak IC (0.46 cm x 25 cm), *n*-hexane/2-propanol = 95/5, *v* = 1.0 mL·min^{-1}, λ = 254 nm, t (minor) = 5.82 min, t (major) = 14.14 min]; $[\alpha]_D^{25}$ = -131.8 (c = 1.0, $CHCl_3$). ^{1}H NMR (400 MHz, $CDCl_3$): δ = 11.12 (s, 1H), 7.53 (d, *J* = 7.9 Hz, 2H), 7.47 – 7.38 (m, 2H), 7.33 (t, *J* = 7.7 Hz, 2H), 7.15 (t, *J* = 7.6 Hz, 2H), 7.05 (d, *J* = 7.3 Hz, 1H), 6.99 – 6.81 (m, 4H), 6.30 (d, *J* = 3.2 Hz, 1H), 4.72 – 4.50 (m, 2H), 4.38 – 4.22 (m, 1H), 4.10 – 3.96 (m, 1H), 3.89 (dd, *J* = 13.7, 4.0 Hz, 1H), 2.89 (d, *J* =

15.1 Hz, 1H), 2.00 (dd, J = 15.1, 5.6 Hz, 1H), 1.48 (s, 9H). ^{13}C NMR (100 MHz, $CDCl_3$): δ = 165.2, 161.2 (d, $J_{F\text{-}C}$ = 244.2 Hz), 155.9, 147.2, 137.3, 134.0 (d, $J_{F\text{-}C}$ = 3.2 Hz), 131.0 (d, $J_{F\text{-}C}$ = 7.5 Hz), 129.5, 129.0, 128.0, 125.7 (q, $J_{F\text{-}C}$ = 283.0 Hz), 125.1, 124.2, 122.4, 120.5, 115.1 (d, $J_{F\text{-}C}$ = 20.9 Hz), 112.3, 79.6, 59.8, 55.2 (q, $J_{F\text{-}C}$ = 22.9 Hz), 52.9, 43.9, 42.6, 28.4, 27.6. ^{19}F NMR (376 MHz, $CDCl_3$): δ = -71.6, -116.2. IR (thin film): ν_{max} (cm^{-1}) = 3384, 3032, 2979, 1679, 1601, 1511, 1499, 1244, 1182, 1165; HRMS (ESI) calcd for $C_{31}H_{31}F_4N_3O_3Na$ $[M+Na]^+$: 592.2194. Found: 592.2193.

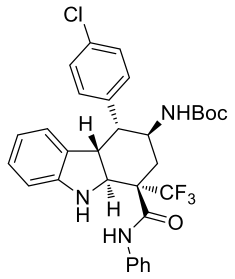

8c (major dr). White solid, 21.5 mg, 37% yield, 93% ee [Daicel Chiralpak IC (0.46 cm x 25 cm), *n*-hexane/2-propanol = 95/5, v = 1.0 mL·min^{-1}, λ = 254 nm, t (minor) = 5.76 min, t (major) = 12.89 min]; $[\alpha]_D^{25}$ = -157.3 (c = 1.0, $CHCl_3$). ^{1}H NMR (400 MHz, C_3D_6O): δ = 11.49 (s, 1H), 7.60 – 7.49 (m, 4H), 7.40 – 7.19 (m, 4H), 7.15 – 7.02 (m, 4H), 6.82 (t, J = 7.4 Hz, 1H), 6.37 (d, J = 3.2 Hz, 1H), 6.11 (d, J = 4.9 Hz, 1H), 4.58 (dd, J = 14.1, 5.3 Hz, 1H), 4.38 – 4.23 (m, 1H), 4.05 (d, J = 3.5 Hz, 1H), 3.90 (dd, J = 14.1, 4.9 Hz, 1H), 2.93 (d, J = 15.1 Hz, 1H), 2.18 (dd, J = 15.3, 5.5 Hz, 1H), 1.43 (s, 9H). ^{13}C NMR (100 MHz, C_3D_6O): δ = 166.1, 156.0, 149.6, 138.8, 138.5, 132.6, 132.1, 129.9, 129.8, 128.9, 128.6, 126.9 (q, $J_{F\text{-}C}$ = 282.5 Hz), 125.6, 124.8, 122.2, 120.9, 113.5, 79.4, 60.4, 56.0 (q, $J_{F\text{-}C}$ = 22.8 Hz), 53.1, 45.5, 44.1, 29.1, 28.5. ^{19}F NMR (376 MHz, $CDCl_3$): δ = -71.6. IR (thin film): ν_{max} (cm^{-1}) = 3384, 3033, 2977, 1677, 1600, 1560, 1495, 1247, 1181, 1167; HRMS (ESI) calcd for $C_{31}H_{31}ClF_3N_3O_3Na$ $[M+Na]^+$: 608.1898. Found: 608.1900.

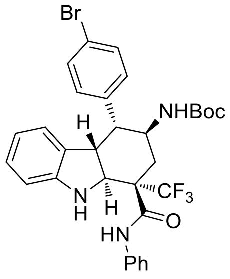

8d (major dr). White solid, 23.2 mg, 37% yield, 93% ee [Daicel Chiralpak IC (0.46 cm x 25 cm), *n*-hexane/2-propanol = 95/5, *v* = 1.0 mL·min^{-1}, λ = 254 nm, t (minor) = 6.03 min, t (major) = 16.08 min]; $[\alpha]_D^{25}$ = -198.0 (c = 1.0, $CHCl_3$). ^1H NMR (400 MHz, $CDCl_3$): δ = 11.09 (s, 1H), 7.52 (d, *J* = 7.8 Hz, 2H), 7.38 – 7.27 (m, 6H), 7.15 (t, *J* = 7.6 Hz, 2H), 7.04 (d, *J* = 7.4 Hz, 1H), 6.96 (d, *J* = 7.8 Hz, 1H), 6.89 (t, *J* = 7.4 Hz, 1H), 6.30 (d, *J* = 3.5 Hz, 1H), 4.63 – 4.47 (m, 2H), 4.34 – 4.25 (m, 1H), 4.00 (d, *J* = 3.5 Hz, 1H), 3.89 (dd, *J* = 13.7, 4.3 Hz, 1H), 2.88 (d, *J* = 15.0 Hz, 1H), 1.97 (dd, *J* = 15.1, 5.7 Hz, 1H), 1.47 (s, 9H). ^{13}C NMR (100 MHz, $CDCl_3$): δ = 165.1, 155.9, 147.2, 137.3, 137.2, 131.3, 131.2, 129.3, 129.0, 128.1, 125.7 (q, $J_{F\text{-}C}$ = 283.6 Hz), 125.1, 124.1, 122.4, 120.5, 112.3, 79.6, 59.9, 55.2 (q, $J_{F\text{-}C}$ = 22.8 Hz), 52.9, 44.0, 42.5, 28.4, 27.6. ^{19}F NMR (376 MHz, $CDCl_3$): δ = -71.6. IR (thin film): ν_{max} (cm^{-1}) = 3383, 3033, 2979, 1705, 1679, 1601, 1560, 1491, 1246, 1182, 1167; HRMS (ESI) calcd for $C_{31}H_{31}BrF_3N_3O_3Na$ $[M+Na]^+$: 652.1393. Found: 652.1392.

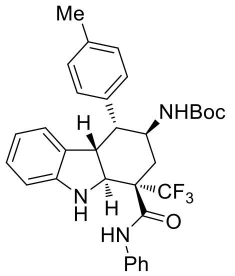

8e (major dr). White solid, 18.0 mg, 32% yield, 94% ee [Daicel Chiralpak IC (0.46 cm x 25 cm), *n*-hexane/2-propanol = 95/5, *v* = 1.0 mL·min^{-1}, λ = 254 nm, t (minor) = 6.67 min, t (major) = 18.95 min]; $[\alpha]_D^{25}$ = -173.1 (c = 1.0, $CHCl_3$). ^1H NMR (500 MHz, $CDCl_3$): δ = 11.17 (s, 1H), 7.53 (d, *J* = 7.8 Hz, 2H), 7.39 – 7.28 (m, 4H), 7.19 – 7.11 (m, 2H), 7.08 (d, *J* = 7.4 Hz, 1H), 7.02 (d, *J* = 7.9 Hz, 2H), 6.94 (d, *J* = 7.8 Hz, 1H), 6.88 (t, *J* = 7.4 Hz, 1H), 6.27 (d, *J* = 3.2 Hz, 1H), 4.61 (dd, *J* = 14.0, 4.8 Hz, 1H), 4.54 (d, *J* = 4.8 Hz, 1H), 4.39 – 4.27 (m, 1H), 4.05 – 3.96 (m, 1H), 3.88 (dd, *J* = 13.9, 4.2 Hz, 1H), 2.88 (d, *J* = 15.0 Hz, 1H), 2.26 (s, 3H), 2.05 (dd, *J* = 15.1, 5.7 Hz, 1H), 1.48 (s, 9H). ^{13}C NMR (125 MHz, $CDCl_3$): δ = 165.4, 155.8, 147.3, 137.3, 135.9, 135.1, 129.9, 129.4, 129.0, 128.9, 127.8, 125.8 (q, $J_{F\text{-}C}$ = 283.3 Hz), 125.0, 124.3, 122.3, 120.5, 112.2, 79.4, 59.9, 55.3 (q, $J_{F\text{-}C}$ = 22.8 Hz), 52.9, 44.2, 42.7, 28.5, 27.7, 20.8. ^{19}F NMR (376 MHz, $CDCl_3$): δ = -71.6. IR (thin film): ν_{max} (cm^{-1}) = 3382, 3029, 2978, 1707, 1678, 1601, 1560, 1498, 1248, 1167; HRMS (ESI) calcd for $C_{32}H_{34}F_3N_3O_3Na$ $[M+Na]^+$: 588.2444. Found: 588.2444.

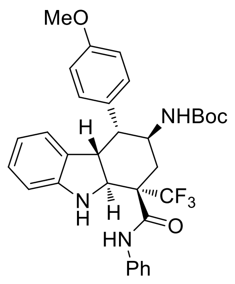

8f (major dr). White solid, 18.5 mg, 32% yield, 92% ee [Daicel Chiralpak IC (0.46 cm x 25 cm), *n*-hexane/2-propanol = 90/10, v = 1.0 mL·min^{-1}, λ = 254 nm, t (minor) = 6.23 min, t (major) = 13.83 min]; $[\alpha]_D^{25}$ = -98.6 (c = 1.0, $CHCl_3$). ^1H NMR (500 MHz, $CDCl_3$): δ = 11.14 (s, 1H), 7.52 (d, *J* = 7.8 Hz, 2H), 7.39 – 7.28 (m, 4H), 7.17 – 7.10 (m, 2H), 7.07 (d, *J* = 7.4 Hz, 1H), 6.94 (d, *J* = 7.8 Hz, 1H), 6.90 – 6.86 (m, 1H), 6.78 – 6.72 (m, 2H), 6.26 (d, *J* = 3.2 Hz, 1H), 4.63 – 4.46 (m, 2H), 4.35 – 4.24 (m, 1H), 4.05 – 3.95 (m, 1H), 3.86 (dd, *J* = 13.5, 4.3 Hz, 1H), 3.73 (s, 3H), 2.87 (d, *J* = 15.0 Hz, 1H), 2.04 (dd, *J* = 15.1, 5.7 Hz, 1H), 1.47 (s, 9H). ^{13}C NMR (125 MHz, $CDCl_3$): δ = 165.3, 157.8, 155.8, 147.3, 137.3, 130.5, 130.2, 129.9, 129.0, 127.8, 125.8 (q, $J_{F\text{-}C}$ = 283.1 Hz), 125.0, 124.3, 122.3, 120.5, 113.6, 112.2, 79.4, 59.8, 55.3 (q, $J_{F\text{-}C}$ = 22.5 Hz), 55.1, 52.9, 43.8, 42.8, 28.5, 27.6. ^{19}F NMR (376 MHz, $CDCl_3$): δ = -71.6. IR (thin film): ν_{max} (cm^{-1}) = 3385, 3302, 3031, 2978, 1678, 1601, 1514, 1499, 1249, 1167; HRMS (ESI) calcd for $C_{32}H_{34}F_3N_3O_4Na$ $[M+Na]^+$: 604.2394. Found: 604.2394.

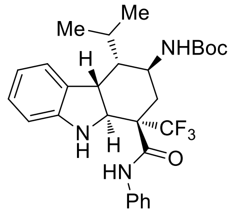

8g (major dr). White solid, 21.7 mg, 42% yield, 86% ee [Daicel Chiralpak IC (0.46 cm x 25 cm), *n*-hexane/2-propanol = 95/5, v = 1.0 mL·min^{-1}, λ = 254 nm, t (minor) = 7.00 min, t (major) = 13.67 min]; $[\alpha]_D^{25}$ = -159.3 (c = 1.0, $CHCl_3$). ^1H NMR (400 MHz, $CDCl_3$): δ = 11.23 (s, 1H), 7.47 (d, *J* = 7.8 Hz, 2H), 7.30 (t, *J* = 7.7 Hz, 2H), 7.19 – 7.07 (m, 3H), 6.96 – 6.86 (m, 2H), 5.97 (s, 1H), 4.56 (s, 1H), 4.26 (dd, *J* = 14.0, 3.7 Hz, 1H), 4.16 – 4.01 (m, 1H), 3.63 (d, *J* = 13.9 Hz, 1H), 2.83 (d, *J* = 15.0 Hz, 1H), 2.40 (d, *J* = 7.8 Hz, 1H), 2.04 (dd, *J* = 14.8, 4.8 Hz, 1H), 1.86 – 1.70 (m, 1H), 1.44 (s, 9H), 1.21 (d, *J* = 6.3 Hz, 3H), 0.83 (d, *J* = 6.8 Hz, 3H). ^{13}C NMR (100 MHz, $CDCl_3$): δ = 165.3, 155.5, 146.7, 137.4, 131.3, 128.9, 127.6, 125.8 (q, $J_{F\text{-}C}$ = 283.3 Hz), 124.9, 124.7, 121.9, 120.4, 112.2, 79.1, 59.1, 55.6 (q, $J_{F\text{-}C}$ = 22.6 Hz), 48.7, 46.0,

43.3, 28.4, 27.5, 26.8, 23.9, 22.4. ^{19}F NMR (376 MHz, $CDCl_3$): δ = -71.6. IR (thin film): ν_{max} (cm^{-1}) = 3383, 3299, 3030, 2976, 1698, 1678, 1601, 1560, 1498, 1248, 1166; HRMS (ESI) calcd for $C_{28}H_{34}F_3N_3O_3Na$ $[M+Na]^+$: 540.2444. Found: 540.2444.

2.3 Representative procedure for the photocatalytic syntheses of tetrahydro-1H-carbazoles via radical cascade reaction of tryptophan derivatives 1 and alkenes 2 under air.

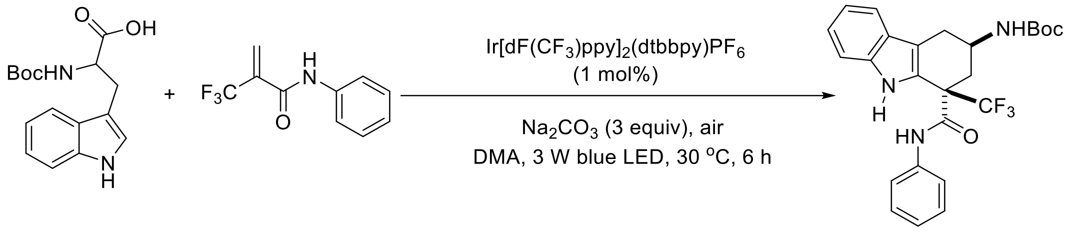

1a **2a** **9a**

Preparation of compound 9a. To a vial (10 mL) were added $Ir[dF(CF_3)ppy]_2(dtbbpy)PF_6$ (1.1 mg, 0.001 mmol, 1 mol%), **1a** (45.7 mg, 0.15 mmol, 1.5 equiv), 2**a** (21.5 mg, 0.1 mmol, 1.0 equiv), Na_2CO_3 (31.8 mg, 0.3 mmol, 3.0 equiv), and N,N-dimethylacetamide (DMA, 2.0 mL). The vial was closed with a cap and the reaction mixture was stirred under air at 30 °C (*note: the stirring speed of the stir bar in the reaction mixture should not be too fast or too slow, and 300 RPM was found to be optimal for this transformation*), and irradiated by a 3 W blue LED lamp (λ = 450–460 nm, the vial was placed at approximately 3 cm away from the light source). After 6 h, the reaction mixture was passed through a short pad of celite and washed with ethyl acetate. The solvents were evaporated under reduced pressure to give the crude mixture, which was purified by flash column chromatography on silica gel (petroleum ether/ethyl acetate = 40:1 to 20:1, *silica gel was soaked with a solution of petroleum ether and triethylamine (1000/1, v/v) before use*) to afford the title compound **9a** as a white solid (24.0 mg, 51% yield). ^{1}H NMR (400 MHz, C_3D_6O): δ = 10.31 (s, 1H), 9.25 (s, 1H), 7.69 (d, J = 7.7 Hz, 2H), 7.54 (d, J = 8.0 Hz, 2H), 7.29 (t, J = 7.8 Hz, 2H), 7.21 (t, J = 7.5 Hz, 1H), 7.17 – 7.02 (m, 2H), 6.73 (d, J = 5.8 Hz, 1H), 4.23 – 4.06 (m, 1H), 3.24 (dd, J = 15.1, 4.3 Hz, 1H), 3.02 (d, J = 13.6 Hz, 1H), 2.82 (dd, J = 14.6, 11.4 Hz, 1H), 2.28 (t, J = 12.8 Hz, 1H), 1.50 (s, 9H). ^{13}C NMR (100 MHz, C_3D_6O): δ = 165.2, 156.9, 138.8, 138.5, 129.1, 126.9, 126.3 (q, $J_{F\text{-}C}$ = 282.6 Hz), 125.2, 123.5, 121.5, 120.0, 119.1, 114.2, 112.5, 79.6, 55.4 (q, $J_{F\text{-}C}$ = 25.3 Hz), 46.4, 34.6, 28.4, 27.7. ^{19}F NMR (376 MHz, $CDCl_3$): δ = -70.6. IR (thin film): ν_{max} (cm^{-1}) = 3340, 3061,

2979, 1682, 1600, 1501, 1253, 1162; HRMS (ESI) calcd for $C_{25}H_{26}F_3N_3O_3Na$ $[M+Na]^+$: 496.1818. Found: 496.1819.

The following compounds **9b–9f** were prepared analogously.

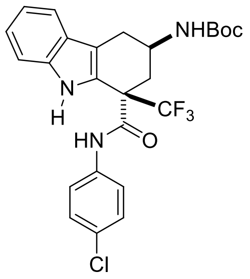

9b. White solid, 22.9 mg, 45% yield. ^1H NMR (400 MHz, C_3D_6O): δ = 10.30 (s, 1H), 9.41 (s, 1H), 7.70 (d, *J* = 8.5 Hz, 2H), 7.59 – 7.43 (m, 2H), 7.32 (d, *J* = 8.8 Hz, 2H), 7.20 (t, *J* = 7.6 Hz, 1H), 7.08 (t, *J* = 7.5 Hz, 1H), 6.75 (d, *J* = 5.8 Hz, 1H), 4.20 – 4.04 (m, 1H), 3.22 (dd, *J* = 15.1, 4.0 Hz, 1H), 2.98 (d, *J* = 13.4 Hz, 1H), 2.81 (dd, *J* = 14.8, 11.3 Hz, 1H), 2.27 (t, *J* = 12.8 Hz, 1H), 1.49 (s, 9H). ^{13}C NMR (100 MHz, C_3D_6O): δ = 165.5, 157.0, 138.6, 137.7, 129.8, 129.2, 126.9, 126.3 (q, $J_{F\text{-}C}$ = 282.3 Hz), 125.1, 123.6, 123.2, 123.1, 120.0, 119.2, 114.5, 112.62, 112.58, 79.7, 55.5 (q, $J_{F\text{-}C}$ = 25.3 Hz), 46.5, 34.7, 28.5, 27.7. ^{19}F NMR (376 MHz, $CDCl_3$): δ = -70.1. IR (thin film): ν_{max} (cm^{-1}) = 3344, 3061, 2980, 1683, 1495, 1253, 1162; HRMS (ESI) calcd for $C_{25}H_{25}ClF_3N_3O_3Na$ $[M+Na]^+$: 530.1429. Found: 530.1428.

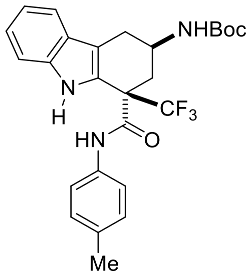

9c. White solid, 26.1 mg, 54% yield. ^1H NMR (400 MHz, C_3D_6O): δ = 10.28 (s, 1H), 9.16 (s, 1H), 7.64 – 7.44 (m, 4H), 7.20 (t, *J* = 7.7 Hz, 1H), 7.14 – 7.00 (m, 3H), 6.71 (d, *J* = 5.9 Hz, 1H), 4.17 – 4.06 (m, 1H), 3.26 – 3.17 (m, 1H), 2.98 (d, *J* = 13.3 Hz, 1H), 2.79 (dd, *J* = 14.9, 11.2 Hz, 1H), 2.32 – 2.20 (m, 4H), 1.49 (s, 9H). ^{13}C NMR (100 MHz, C_3D_6O): δ = 165.1, 156.9, 138.6, 136.4, 134.8, 129.7, 127.0, 126.4 (q, $J_{F\text{-}C}$ = 282.6 Hz), 125.5, 123.5, 121.6, 120.0, 119.2, 114.3, 112.6, 79.6, 55.4 (q, $J_{F\text{-}C}$ = 25.2 Hz), 46.5, 34.7, 28.5, 27.8, 20.6. ^{19}F NMR (376 MHz, $CDCl_3$): δ = -70.6. IR (thin film): ν_{max} (cm^{-1}) = 3343, 3059, 2979, 1682, 1515, 1254, 1161; HRMS (ESI) calcd for $C_{26}H_{28}F_3N_3O_3Na$ $[M+Na]^+$: 510.1975. Found: 510.1974

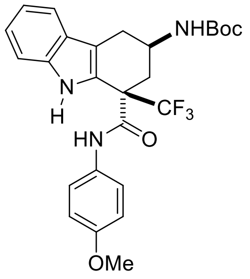

9d. White solid, 27.0 mg, 54% yield. ^{1}H NMR (400 MHz, C_3D_6O): δ = 10.27 (s, 1H), 9.12 (s, 1H), 7.65 – 7.39 (m, 4H), 7.19 (t, *J* = 7.6 Hz, 1H), 7.08 (t, *J* = 7.4 Hz, 1H), 6.96 – 6.79 (m, 2H), 6.69 (d, *J* = 5.9 Hz, 1H), 4.22 – 4.03 (m, 1H), 3.74 (s, 3H), 3.34 – 3.09 (m, 1H), 2.97 (d, *J* = 13.3 Hz, 1H), 2.79 (dd, *J* = 14.8, 11.3 Hz, 1H), 2.25 (t, *J* = 12.8 Hz, 1H), 1.49 (s, 9H). ^{13}C NMR (100 MHz, C_3D_6O): δ = 165.0, 157.6, 156.9, 138.6, 131.4, 127.0, 126.4 (q, $J_{F\text{-}C}$ = 282.2 Hz), 125.6, 123.5, 123.3, 120.0, 119.2, 114.3, 112.6, 79.6, 55.5, 55.4 (q, $J_{F\text{-}C}$ = 23.7 Hz), 46.4, 34.8, 28.5, 27.9. ^{19}F NMR (376 MHz, $CDCl_3$): δ = -70.6. IR (thin film): ν_{max} (cm^{-1}) = 3335, 3061, 2978, 1682, 1513, 1249, 1162; HRMS (ESI) calcd for $C_{26}H_{28}F_3N_3O_4Na$ $[M+Na]^+$: 526.1924. Found: 526.1924.

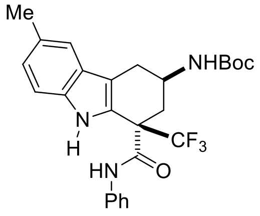

9e. White solid, 20.0 mg, 41% yield. ^{1}H NMR (500 MHz, C_3D_6O): δ = 10.15 (s, 1H), 9.17 (s, 1H), 7.67 (d, *J* = 7.8 Hz, 2H), 7.40 (d, *J* = 8.3 Hz, 1H), 7.38 – 7.24 (m, 3H), 7.10 (t, *J* = 7.4 Hz, 1H), 7.04 (d, *J* = 8.2 Hz, 1H), 6.69 (d, *J* = 5.7 Hz, 1H), 4.16 – 4.05 (m, 1H), 3.23 – 3.16 (m, 1H), 2.98 (d, *J* = 13.3 Hz, 1H), 2.77 (dd, *J* = 14.8, 11.3 Hz, 1H), 2.42 (s, 3H), 2.25 (t, *J* = 12.8 Hz, 1H), 1.48 (s, 9H). ^{13}C NMR (125 MHz, C_3D_6O): δ = 164.4, 156.0, 137.9, 136.1, 128.3, 128.1, 126.3, 125.4 (q, $J_{F\text{-}C}$ = 282.1 Hz), 124.4, 124.2, 120.6, 117.9, 112.9, 111.4, 78.6, 54.6 (q, $J_{F\text{-}C}$ = 25.0 Hz), 45.5, 33.8, 27.5, 26.9, 20.4. ^{19}F NMR (376 MHz, $CDCl_3$): δ = -70.6. IR (thin film): ν_{max} (cm^{-1}) = 3347, 3061, 2979, 1683, 1601, 1500, 1254, 1159; HRMS (ESI) calcd for $C_{26}H_{28}F_3N_3O_3Na$ $[M+Na]^+$: 510.1975. Found: 510.1975.

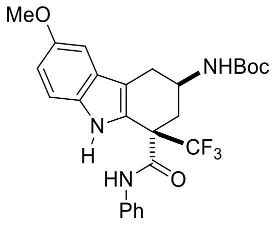

9f. White solid, 27.0 mg, 54% yield. ^{1}H NMR (400 MHz, C_3D_6O): δ = 10.15 (s, 1H), 9.16 (s, 1H), 7.67 (d, *J* = 7.7 Hz, 2H), 7.41 (d, *J* = 8.8 Hz, 1H), 7.30 (t, *J* = 7.8 Hz, 2H), 7.11 (t, *J* = 7.4 Hz, 1H), 7.03 (s, 1H), 6.86 (dd, *J* = 8.8, 2.0 Hz, 1H), 6.69 (d, *J* = 6.1 Hz, 1H), 4.19 – 4.05 (m, 1H), 3.82 (s, 3H), 3.21 (dd, *J* = 15.1, 4.2 Hz, 1H), 2.98 (d, *J* = 13.3 Hz, 1H), 2.76 (dd, *J* = 14.8, 11.2 Hz, 1H), 2.26 (t, *J* = 12.8 Hz, 1H), 1.48 (s, 9H). ^{13}C NMR (100 MHz, C_3D_6O): δ = 165.3, 156.9, 155.0, 138.8, 133.7, 129.2, 127.3, 126.4 (q, $J_{F\text{-}C}$ = 282.4 Hz), 125.8, 125.3, 121.5, 114.1, 114.0, 113.4, 100.8, 79.6, 55.7, 55.6 (q, $J_{F\text{-}C}$ = 23.8 Hz), 46.4, 34.7, 28.5, 28.0. ^{19}F NMR (376 MHz, $CDCl_3$): δ = -70.6. IR (thin film): ν_{max} (cm^{-1}) = 3361, 3061, 2978, 1684, 1600, 1500, 1442, 1157; HRMS (ESI) calcd for $C_{26}H_{28}F_3N_3O_4Na$ [M+Na]$^+$: 526.1924. Found: 526.1924.

2.4 Representative procedure for the stereoselective radical cascade reaction of substrates 10 and alkenes 2 under photoredox catalysis.

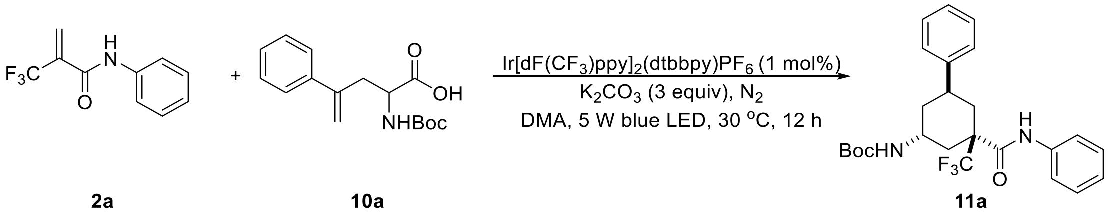

Preparation of compound 11a. To a Young Schlenk tube (10 mL) were added Ir[dF(CF_3)ppy]$_2$(dtbbpy)PF_6 (1.1 mg, 0.001 mmol, 1 mol%), **10a** (43.7 mg, 0.15 mmol, 1.5 equiv), **2a** (21.5 mg, 0.1 mmol, 1.0 equiv), K_2CO_3 (41.5 mg, 0.3 mmol, 3.0 equiv), and N,N-dimethylacetamide (DMA, 2.0 mL). Subsequently, the reaction mixture was degassed through several freeze-pump-thaw cycles until no bubbles were released. The reaction mixture was stirred under N_2 atmosphere at 30 °C, and irradiated by a 5 W blue LED lamp (*λ* = 450−460 nm, the tube was placed at approximately 2 cm away from the light source). After 12 h, the reaction mixture was passed through a short pad of celite and washed with ethyl acetate. The solvents were evaporated under reduced pressure to give the crude mixture, which was purified by flash column chromatography on silica gel (petroleum ether/ethyl acetate = 40:1 to 20:1, *silica gel*

was soaked with a solution of petroleum ether and triethylamine (1000/1, v/v) before use) to afford compound **11a** (major dr) as white solid (24.3 mg, 53% yield) and compound **11a'** (minor dr) as white solid (7.0 mg, 15% yield).

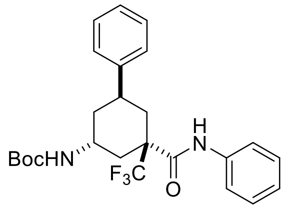

11a (major dr)

11a (major dr). ^{1}H NMR (400 MHz, $CDCl_3$): δ = 7.69 (s, 1H), 7.54 (d, *J* = 7.7 Hz, 2H), 7.40 – 7.32 (m, 4H), 7.30 – 7.26 (m, 2H), 7.26 – 7.22 (m, 1H), 7.21 – 7.13 (m, 1H), 5.19 (d, *J* = 4.1 Hz, 1H), 4.24 – 4.07 (m, 1H), 3.20 (t, *J* = 12.7 Hz, 1H), 2.78 (d, *J* = 14.5 Hz, 1H), 2.60 (d, *J* = 12.9 Hz, 1H), 2.04 (d, *J* = 13.8 Hz, 1H), 1.97 (dd, *J* = 14.8, 4.1 Hz, 1H), 1.83 (dd, *J* = 13.2, 3.2 Hz, 1H), 1.79 – 1.67 (m, 1H), 1.20 (s, 9H). ^{13}C NMR (100 MHz, $CDCl_3$): δ = 166.4, 155.6, 144.3, 137.0, 129.0, 128.7, 126.8, 126.4 (q, $J_{F\text{-}C}$ = 281.7 Hz), 125.2, 120.9, 79.6, 52.2 (q, $J_{F\text{-}C}$ = 24.4 Hz), 45.4, 36.4, 34.5, 33.9, 30.4, 28.0. ^{19}F NMR (376 MHz, $CDCl_3$): δ = -73.9. IR (thin film): ν_{max} (cm^{-1}) = 3364, 2972, 2928, 1670, 1499, 1443, 1244, 1168; HRMS (ESI) calcd for $C_{25}H_{29}F_3N_2O_3Na$ [M+Na]$^+$: 485.2022. Found: 485.2027.

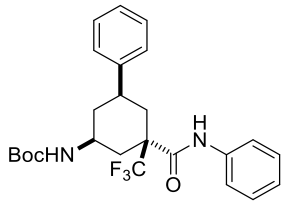

11a' (minor dr)

11a' (minor dr). ^{1}H NMR (400 MHz, $CDCl_3$): δ = 8.53 (s, 1H), 7.78 (d, *J* = 7.0 Hz, 2H), 7.44 – 7.36 (m, 2H), 7.35 – 7.28 (m, 2H), 7.26 – 7.13 (m, 4H), 4.77 (s, 1H), 3.88 – 3.72 (m, 1H), 3.03 – 2.72 (m, 3H), 2.14 – 2.02 (m, 1H), 1.66 (t, *J* = 12.7 Hz, 2H), 1.57 – 1.44 (m, 10H). ^{13}C NMR (100 MHz, $CDCl_3$): δ = 164.5, 155.7, 143.7, 137.7, 128.9, 128.6, 126.8, 126.7, 125.7 (q, $J_{F\text{-}C}$ = 281.7 Hz), 124.9, 120.7, 80.2, 54.1 (q, $J_{F\text{-}C}$ = 24.4 Hz), 47.6, 39.1, 37.8, 34.7, 33.6, 28.4. ^{19}F NMR (376 MHz, $CDCl_3$): δ = -74.5. IR (thin film): ν_{max} (cm^{-1}) = 3350, 2978, 2934, 1675, 1500, 1264, 1235, 1163; HRMS (ESI) calcd for $C_{25}H_{29}F_3N_2O_3Na$ [M+Na]$^+$: 485.2022. Found: 485.2026.

The following compounds **11b–11g** and **11b'–11g'** were prepared analogously.

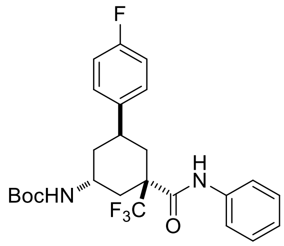

11b (major dr)

11b (major dr). White solid, 16.0 mg, 33% yield. ^{1}H NMR (400 MHz, $CDCl_3$): δ = 7.65 (s, 1H), 7.53 (d, *J* = 8.0 Hz, 2H), 7.36 (t, *J* = 8.0 Hz, 2H), 7.26 – 7.20 (m, 2H), 7.17 (t, *J* = 7.2 Hz, 1H), 7.08 – 6.97 (m, 2H), 5.11 (d, *J* = 2.8 Hz, 1H), 4.27 – 4.05 (m, 1H), 3.21 (t, *J* = 12.6 Hz, 1H), 2.78 (d, *J* = 14.4 Hz, 1H), 2.57 (d, *J* = 13.2 Hz, 1H), 2.12 – 1.91 (m, 2H), 1.82 – 1.72 (m, 1H), 1.72 – 1.62 (m, 1H), 1.18 (s, 9H). ^{13}C NMR (100 MHz, $CDCl_3$): δ = 166.3, 161.7 (d, $J_{F\text{-}C}$ = 243.5 Hz), 155.6, 140.0 (d, $J_{F\text{-}C}$ = 3.2 Hz), 137.0, 129.0, 128.2 (d, $J_{F\text{-}C}$ = 7.8 Hz), 126.3 (q, $J_{F\text{-}C}$ = 281.7 Hz), 125.2, 120.9, 115.5 (d, $J_{F\text{-}C}$ = 21.1 Hz), 79.7, 52.2 (q, $J_{F\text{-}C}$ = 24.0 Hz), 45.4, 36.5, 34.6, 33.3, 30.5, 28.0. ^{19}F NMR (376 MHz, $CDCl_3$): δ = -73.9, -116.1. IR (thin film): ν_{max} (cm^{-1}) = 3447, 3364, 2978, 2932, 1698, 1511, 1443, 1231, 1162; HRMS (ESI) calcd for $C_{25}H_{28}F_4N_2O_3Na$ $[M+Na]^+$: 503.1928. Found: 503.1932.

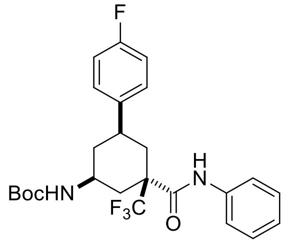

11b' (minor dr)

11b’ (minor dr). White solid, 6.5 mg, 14% yield. ^{1}H NMR (400 MHz, $CDCl_3$): δ = 8.46 (s, 1H), 7.75 (d, *J* = 8.0 Hz, 2H), 7.44 – 7.32 (m, 2H), 7.24 – 7.11 (m, 3H), 7.06 – 6.93 (m, 2H), 4.67 (d, *J* = 4.8 Hz, 1H), 3.86 – 3.70 (m, 1H), 3.06 – 2.58 (m, 3H), 2.05 (d, *J* = 11.6 Hz, 1H), 1.69 – 1.63 (m, 1H), 1.63 – 1.59 (m, 1H), 1.49 (s, 9H), 1.45 – 1.36 (m, 1H). ^{13}C NMR (100 MHz, $CDCl_3$): δ = 164.4, 161.7 (d, $J_{F\text{-}C}$ = 243.5 Hz), 155.7, 139.4 (d, $J_{F\text{-}C}$ = 3.2 Hz), 137.6, 129.0, 128.2 (d, $J_{F\text{-}C}$ = 7.9 Hz), 125.6 (q, $J_{F\text{-}C}$ = 281.7 Hz), 125.0, 120.7, 115.4 (d, $J_{F\text{-}C}$ = 21.1 Hz), 80.3, 54.1 (q, $J_{F\text{-}C}$ = 24.3 Hz), 47.6, 38.5, 38.0, 34.9, 33.7, 28.4. ^{19}F NMR (376 MHz, $CDCl_3$): δ = -74.5, -116.0. IR (thin film): ν_{max} (cm^{-1}) = 3343, 2979, 2933, 1675, 1512, 1262, 1234, 1160; HRMS (ESI) calcd for $C_{25}H_{28}F_4N_2O_3Na$ $[M+Na]^+$: 503.1928. Found: 503.1926.

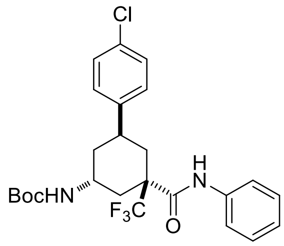

11c (major dr)

11c (major dr). White solid, 23.5 mg, 47% yield. ^{1}H NMR (400 MHz, $CDCl_3$): δ = 7.71 (s, 1H), 7.52 (d, *J* = 7.6 Hz, 2H), 7.40 – 7.27 (m, 4H), 7.24 – 7.13 (m, 3H), 5.13 (d, *J* = 4.1 Hz, 1H), 4.24 – 4.02 (m, 1H), 3.20 (t, *J* = 12.7 Hz, 1H), 2.78 (d, *J* = 14.4 Hz, 1H), 2.57 (d, *J* = 13.2 Hz, 1H), 2.02 – 1.91 (m, 2H), 1.76 (td, *J* = 13.2, 3.6 Hz, 1H), 1.65 (t, *J* = 12.9 Hz, 1H), 1.17 (s, 9H). ^{13}C NMR (100 MHz, $CDCl_3$): δ = 166.2, 155.6, 142.7, 137.0, 132.5, 128.9, 128.8, 128.2, 126.3 (q, $J_{F\text{-}C}$ = 281.7 Hz), 125.2, 121.0, 79.7, 52.1 (q, $J_{F\text{-}C}$ = 24.1 Hz), 45.3, 36.3, 34.3, 33.4, 30.5, 28.0. ^{19}F NMR (376 MHz, $CDCl_3$): δ = -73.9. IR (thin film): ν_{max} (cm^{-1}) = 3442, 3352, 2978, 2935, 1698, 1533, 1494, 1245, 1169; HRMS (ESI) calcd for $C_{25}H_{28}ClF_3N_2O_3Na$ $[M+Na]^+$: 519.1633. Found: 519.1634.

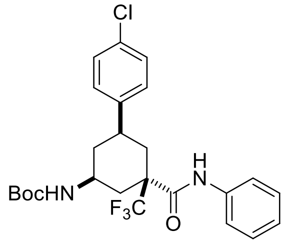

11c' (minor dr)

11c’ (minor dr). White solid, 9.3 mg, 19% yield. ^{1}H NMR (400 MHz, $CDCl_3$): δ = 8.48 (s, 1H), 7.75 (d, *J* = 7.6 Hz, 2H), 7.41 – 7.33 (m, 2H), 7.30 – 7.24 (m, 2H), 7.21 – 7.09 (m, 3H), 4.70 (d, *J* = 5.2 Hz, 1H), 3.83 – 3.70 (m, 1H), 2.97 – 2.62 (m, 3H), 2.12 – 1.92 (m, 1H), 1.74 – 1.54 (m, 3H), 1.49 (s, 9H). ^{13}C NMR (100 MHz, $CDCl_3$): δ = 164.3, 155.7, 142.2, 137.6, 132.6, 128.9, 128.8, 128.1, 125.6 (q, $J_{F\text{-}C}$ = 282.0 Hz), 125.0, 120.7, 80.3, 54.1 (q, $J_{F\text{-}C}$ = 24.3 Hz), 47.6, 38.6, 37.8, 34.6, 33.7, 28.4. ^{19}F NMR (376 MHz, $CDCl_3$): δ = -74.5. IR (thin film): ν_{max} (cm^{-1}) = 3351, 2977, 2931, 1675, 1538, 1495, 1263, 1235, 1162; HRMS (ESI) calcd for $C_{25}H_{28}ClF_3N_2O_3Na$ $[M+Na]^+$: 519.1633. Found: 519.1632.

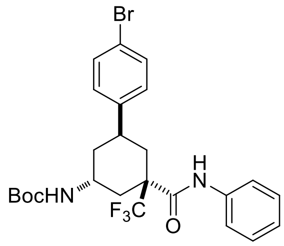

11d (major dr)

11d (major dr). White solid, 24.5 mg, 45% yield. ^{1}H NMR (400 MHz, $CDCl_3$): δ = 7.67 (s, 1H), 7.52 (d, *J* = 8.0 Hz, 2H), 7.48 – 7.42 (m, 2H), 7.39 – 7.31 (m, 2H), 7.21 – 7.10 (m, 3H), 5.10 (d, *J* = 3.4 Hz, 1H), 4.25 – 4.02 (m, 1H), 3.32 – 3.08 (m, 1H), 2.77 (d, *J* = 14.8 Hz, 1H), 2.57 (d, *J* = 13.2 Hz, 1H), 2.07 – 1.89 (m, 2H), 1.85 – 1.72 (m, 1H), 1.65 (t, *J* = 12.9 Hz, 1H), 1.17 (s, 9H). ^{13}C NMR (100 MHz, $CDCl_3$): δ = 166.2, 155.5, 143.3, 137.0, 131.8, 129.0, 128.6, 126.3 (q, $J_{F\text{-}C}$ = 281.6 Hz), 125.2, 120.9, 120.5, 79.7, 52.1 (q, $J_{F\text{-}C}$ = 23.9 Hz), 45.3, 36.3, 34.3, 33.5, 30.5, 28.0. ^{19}F NMR (376 MHz, $CDCl_3$): δ = -73.9. IR (thin film): ν_{max} (cm^{-1}) = 3361, 2978, 2934, 1697, 1491, 1245, 1169; HRMS (ESI) calcd for $C_{25}H_{28}BrF_3N_2O_3Na$ $[M+Na]^+$: 563.1128. Found: 563.1127.

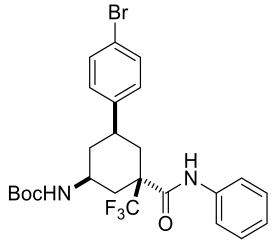

11d' (minor dr)

11d’ (minor dr). White solid, 9.0 mg, 17% yield. ^{1}H NMR (400 MHz, $CDCl_3$): δ = 8.46 (s, 1H), 7.75 (d, *J* = 7.8 Hz, 2H), 7.46 – 7.40 (m, 2H), 7.40 – 7.34 (m, 2H), 7.21 – 7.13 (m, 1H), 7.11 – 7.04 (m, 2H), 4.69 (d, *J* = 5.4 Hz, 1H), 3.85 – 3.69 (m, 1H), 2.92 – 2.65 (m, 3H), 2.04 (d, *J* = 11.6 Hz, 1H), 1.68 – 1.63 (m, 1H), 1.63 – 1.58 (m, 1H), 1.49 (s, 9H), 1.45 – 1.38 (m, 1H). ^{13}C NMR (100 MHz, $CDCl_3$): δ = 164.3, 155.7, 142.7, 137.6, 131.8, 129.0, 128.5, 125.6 (q, $J_{F\text{-}C}$ = 282.4 Hz), 125.0, 120.7, 120.6, 80.3, 54.1 (q, $J_{F\text{-}C}$ = 24.2 Hz), 47.6, 38.7, 37.7, 34.6, 33.7, 28.4. ^{19}F NMR (376 MHz, $CDCl_3$): δ = -74.5. IR (thin film): ν_{max} (cm^{-1}) = 3350, 2978, 2929, 1686, 1536, 1264, 1234, 1161; HRMS (ESI) calcd for $C_{25}H_{28}BrF_3N_2O_3Na$ $[M+Na]^+$: 563.1128. Found: 563.1122.

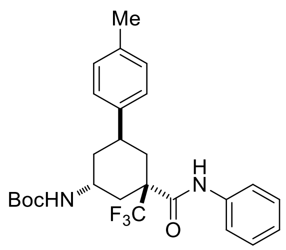

11e (major dr)

11e (major dr). White solid, 17.0 mg, 36% yield. ^1H NMR (400 MHz, $CDCl_3$): δ = 7.68 (s, 1H), 7.59 – 7.50 (m, 2H), 7.42 – 7.31 (m, 2H), 7.21 – 7.13 (m, 5H), 5.17 (d, *J* = 3.9 Hz, 1H), 4.21 – 4.10 (m, 1H), 3.16 (t, *J* = 12.7 Hz, 1H), 2.78 (d, *J* = 14.4 Hz, 1H), 2.58 (d, *J* = 12.8 Hz, 1H), 2.34 (s, 3H), 2.04 – 1.92 (m, 2H), 1.85 – 1.65 (m, 2H), 1.19 (s, 9H). ^{13}C NMR (100 MHz, $CDCl_3$): δ = 166.4, 155.6, 141.3, 137.1, 136.4, 129.3, 129.0, 126.7, 126.4 (q, J_{F-C} = 281.6 Hz), 125.2, 120.9, 79.6, 52.2 (q, J_{F-C} = 23.3 Hz), 45.4, 36.5, 34.6, 33.5, 30.5, 28.0, 20.9. ^{19}F NMR (376 MHz, $CDCl_3$): δ = -73.9. IR (thin film): ν_{max} (cm^{-1}) = 3367, 2977, 2932, 1698, 1500, 1267, 1245, 1168; HRMS (ESI) calcd for $C_{26}H_{31}F_3N_2O_3Na$ $[M+Na]^+$: 499.2179. Found: 499.2178.

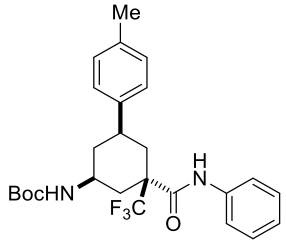

11e' (minor dr)

11e' (minor dr). White solid, 7.2 mg, 15% yield. ^1H NMR (400 MHz, $CDCl_3$): δ = 8.48 (s, 1H), 7.76 (d, *J* = 7.9 Hz, 2H), 7.45 – 7.33 (m, 2H), 7.22 – 7.05 (m, 5H), 4.68 (d, *J* = 5.4 Hz, 1H), 3.88 – 3.71 (m, 1H), 2.98 – 2.61 (m, 3H), 2.32 (s, 3H), 2.12 – 1.99 (m, 1H), 1.74 – 1.57 (m, 3H), 1.49 (s, 9H). ^{13}C NMR (100 MHz, $CDCl_3$): δ = 164.5, 155.7, 140.8, 137.7, 136.4, 129.3, 128.9, 125.7 (q, J_{F-C} = 281.9 Hz), 126.6, 124.9, 120.6, 80.2, 54.1 (q, J_{F-C} = 24.2 Hz), 47.7, 38.7, 37.9, 34.9, 33.6, 28.4, 21.0. ^{19}F NMR (376 MHz, $CDCl_3$): δ = -74.5. IR (thin film): ν_{max} (cm^{-1}) = 3348, 2980, 2926, 1675, 1501, 1262, 1235, 1163; HRMS (ESI) calcd for $C_{26}H_{31}F_3N_2O_3Na$ $[M+Na]^+$: 499.2179. Found: 499.2176.

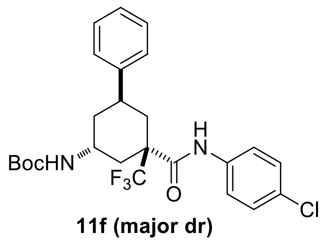

11f (major dr)

11f (major dr). White solid, 17.0 mg, 34% yield. ^{1}H NMR (400 MHz, $CDCl_3$): δ = 7.68 (s, 1H), 7.55 – 7.46 (m, 2H), 7.37 – 7.31 (m, 4H), 7.30 – 7.25 (m, 2H), 7.26 – 7.21 (m, 1H), 5.03 (d, *J* = 2.0 Hz, 1H), 4.19 – 4.05 (m, 1H), 3.26 – 3.12 (m, 1H), 2.80 (d, *J* = 14.8 Hz, 1H), 2.60 (d, *J* = 12.8 Hz, 1H), 2.06 – 2.00 (m, 1H), 2.00 – 1.93 (m, 1H), 1.88 – 1.78 (m, 1H), 1.71 (t, *J* = 12.9 Hz, 1H), 1.20 (s, 9H). ^{13}C NMR (100 MHz, $CDCl_3$): δ = 166.4, 155.7, 144.1, 135.6, 130.3, 129.0, 128.7, 126.9, 126.8, 126.3 (q, $J_{F\text{-}C}$ = 281.7 Hz), 122.2, 79.8, 52.2 (q, $J_{F\text{-}C}$ = 23.6 Hz), 45.5, 36.4, 34.4, 33.9, 30.5, 28.1. ^{19}F NMR (376 MHz, $CDCl_3$): δ = -73.9. IR (thin film): ν_{max} (cm^{-1}) = 3346, 2976, 2932, 1698, 1494, 1243, 1167; HRMS (ESI) calcd for $C_{25}H_{28}ClF_3N_2O_3Na$ [M+Na]$^+$: 519.1633. Found: 519.1627.

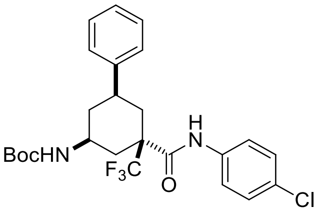

11f' (minor dr)

11f' (minor dr). White solid, 6.0 mg, 12% yield. ^{1}H NMR (400 MHz, $CDCl_3$): δ = 8.59 (s, 1H), 7.73 (d, *J* = 8.8 Hz, 2H), 7.40 – 7.27 (m, 4H), 7.25 – 7.13 (m, 3H), 4.71 (d, *J* = 5.4 Hz, 1H), 3.82 – 3.66 (m, 1H), 2.92 – 2.68 (m, 3H), 2.11 – 2.01 (m, 1H), 1.74 – 1.60 (m, 3H), 1.49 (s, 9H). ^{13}C NMR (100 MHz, $CDCl_3$): δ = 164.6, 155.9, 143.6, 136.3, 130.0, 129.0, 128.7, 126.9, 126.7, 125.6 (q, $J_{F\text{-}C}$ = 281.5 Hz), 121.9, 80.4, 54.3 (q, $J_{F\text{-}C}$ = 24.5 Hz), 47.8, 39.3, 37.7, 34.8, 33.7, 28.4. ^{19}F NMR (376 MHz, $CDCl_3$): δ = -74.5. IR (thin film): ν_{max} (cm^{-1}) = 3347, 2979, 2931, 1675, 1494, 1263, 1235, 1161; HRMS (ESI) calcd for $C_{25}H_{28}ClF_3N_2O_3Na$ [M+Na]$^+$: 519.1633. Found: 519.1629.

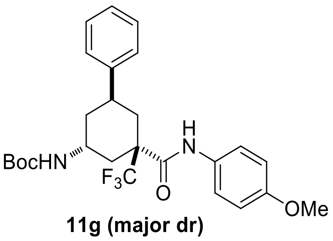

11g (major dr)

11g (major dr). White solid, 17.0 mg, 35% yield. ^{1}H NMR (400 MHz, $CDCl_3$): δ = 7.62 (s, 1H), 7.47 – 7.39 (m, 2H), 7.37 – 7.30 (m, 2H), 7.29 – 7.26 (m, 2H), 7.25 – 7.21 (m, 1H), 6.94 – 6.85 (m, 2H), 5.28 (d, *J* = 3.7 Hz, 1H), 4.23 – 4.09 (m, 1H), 3.81 (s, 3H), 3.17 (t, *J* = 12.4 Hz, 1H), 2.74 (d, *J* = 14.4 Hz, 1H), 2.57 (d, *J* = 13.2 Hz, 1H), 2.12 – 2.00 (m, 1H), 1.97 (dd, *J* = 14.7, 4.2 Hz, 1H), 1.84 – 1.76 (m, 1H), 1.76 – 1.68 (m, 1H), 1.25 (s, 9H). ^{13}C NMR

(100 MHz, $CDCl_3$): δ = 166.4, 157.2, 155.6, 144.3, 130.0, 128.7, 126.8, 126.4 (q, J_{F-C} = 281.7 Hz), 123.0, 114.2, 79.6, 55.5, 52.1 (q, J_{F-C} = 23.8 Hz), 45.3, 36.5, 34.7, 33.9, 30.4, 28.1. ^{19}F NMR (376 MHz, $CDCl_3$): δ = -74.0. IR (thin film): ν_{max} (cm^{-1}) = 3369, 2976, 2932, 1691, 1514, 1247, 1167; HRMS (ESI) calcd for $C_{26}H_{31}F_3N_2O_4Na$ [M+Na]$^+$: 515.2128. Found: 515.2127.

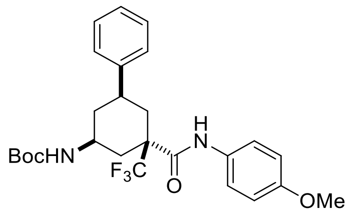

11g' (minor dr)

11g' (minor dr). White solid, 7.0 mg, 14% yield. ^{1}H NMR (400 MHz, $CDCl_3$): δ = 8.37 (s, 1H), 7.66 (d, *J* = 8.4 Hz, 2H), 7.31 (t, *J* = 7.3 Hz, 2H), 7.25 – 7.17 (m, 3H), 6.97 – 6.87 (m, 2H), 4.68 (d, *J* = 5.2 Hz, 1H), 3.88 – 3.70 (m, 4H), 2.92 – 2.69 (m, 3H), 2.13 – 2.03 (m, 1H), 1.72 – 1.57 (m, 3H), 1.49 (s, 9H). ^{13}C NMR (100 MHz, $CDCl_3$): δ = 164.2, 156.9, 155.7, 143.8, 130.8, 128.6, 126.8, 126.7, 125.7 (q, J_{F-C} = 281.7 Hz), 122.3, 114.1, 80.2, 55.5, 54.0 (q, J_{F-C} = 23.2 Hz), 47.6, 39.1, 37.9, 34.8, 33.8, 28.4. ^{19}F NMR (376 MHz, $CDCl_3$): δ = -74.5. IR (thin film): ν_{max} (cm^{-1}) = 3339, 2976, 2932, 1672, 1513, 1248, 1235, 1160; HRMS (ESI) calcd for $C_{26}H_{31}F_3N_2O_4Na$ [M+Na]$^+$: 515.2128. Found: 515.2125.

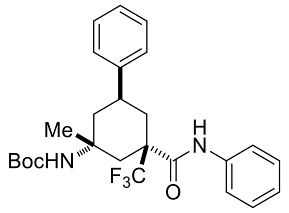

11h (major dr)

In lieu of the standard conditions, the reaction was performed with 2 mol% of Ir[dF(CF_3)ppy]$_2$(dtbbpy)PF_6, and 2.0 equiv of **10h** for 18 h. **11h** (major dr). White solid, 10.0 mg, 21% yield. ^{1}H NMR (400 MHz, $CDCl_3$): δ = 7.75 (s, 1H), 7.57 – 7.49 (m, 2H), 7.41 – 7.27 (m, 6H), 7.26 – 7.21 (m, 1H), 7.21 – 7.15 (m, 1H), 4.65 (s, 1H), 3.27 – 3.16 (m, 1H), 2.92 (d, *J* = 14.2 Hz, 1H), 2.66 (d, *J* = 13.6 Hz, 1H), 2.32 (d, *J* = 14.4 Hz, 1H), 1.92 (t, *J* = 12.7 Hz, 1H), 1.83 – 1.79 (m, 1H), 1.65 (t, *J* = 13.0 Hz, 1H), 1.45 (s, 9H), 1.43 (s, 3H). ^{13}C NMR (100 MHz, $CDCl_3$): δ = 165.6, 154.2, 144.3, 136.8, 129.1, 128.6, 126.9, 126.8, 126.3 (q, J_{F-C} = 282.0 Hz), 125.3, 121.0, 79.6, 53.3 (q, J_{F-C} = 23.5 Hz), 52.9, 43.9, 36.9, 35.8, 33.9, 28.4, 24.1. ^{19}F NMR (376 MHz, $CDCl_3$): δ = -73.8. IR (thin film): ν_{max} (cm^{-1}) = 3352, 2979, 2929, 1686, 1528, 1499, 1233, 1162; HRMS (ESI) calcd for $C_{26}H_{31}F_3N_2O_3Na$ [M+Na]$^+$: 499.2179. Found: 499.2176.

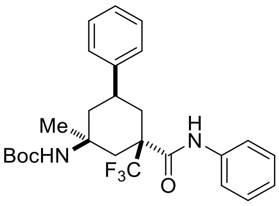

11h' (minor dr)

11h' (minor dr). White solid, 9.0 mg, 19% yield. ^{1}H NMR (400 MHz, $CDCl_3$): δ = 7.75 (s, 1H), 7.58 (d, *J* = 7.6 Hz, 2H), 7.39 – 7.26 (m, 6H), 7.26 – 7.20 (m, 1H), 7.13 (d, *J* = 7.6 Hz, 1H), 4.68 (s, 1H), 3.27 – 3.12 (m, 2H), 2.67 (d, *J* = 12.8 Hz, 1H), 2.20 – 2.08 (m, 1H), 1.72 – 1.62 (m, 2H), 1.56 (t, *J* = 13.6 Hz, 1H), 1.47 (s, 3H), 1.12 (s, 9H). ^{13}C NMR (100 MHz, $CDCl_3$): δ = 165.2, 155.5, 144.2, 137.4, 128.9, 128.7, 126.3 (q, $J_{F\text{-}C}$ = 282.4 Hz), 126.9, 126.8, 124.8, 120.7, 79.7, 53.1 (q, $J_{F\text{-}C}$ = 23.2 Hz), 52.3, 45.1, 35.1, 34.8, 34.0, 28.4, 28.0. ^{19}F NMR (376 MHz, $CDCl_3$): δ = -74.2. IR (thin film): ν_{max} (cm^{-1}) = 3363, 2967, 2929, 1713, 1533, 1259, 1233, 1153; HRMS (ESI) calcd for $C_{26}H_{31}F_3N_2O_3Na$ $[M+Na]^+$: 499.2179. Found: 499.2183.

2.5 Representative procedure for Ag-catalyzed asymmetric reaction of sulfonylindoles 4 with glycine derivative 5.

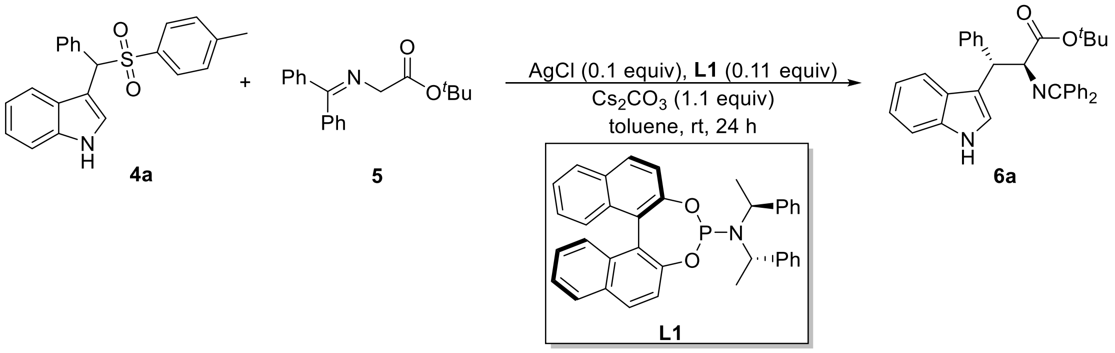

Preparation of compound 6a.[9] To a Schlenk tube were added AgCl (2.9 mg, 0.02 mmol, 0.1 equiv), (*R, S, S*)-**L1** (11.8 mg, 0.022 mmol, 0.11 equiv), and toluene (2.0 mL). The reaction mixture was stirred for 10 mins. Then the glycine imine **5** (64.9 mg, 0.22 mmol, 1.1 equiv) was added to the mixture and the resulting mixture was stirred for further 5 mins. Sulfonylindole **4a** (72.2 mg, 0.2 mmol, 1.0 equiv) and Cs_2CO_3 (71.7 mg, 0.22 mmol, 1.1 equiv) were added subsequently and the reaction mixture was stirred at room temperature for 24 h. Then the reaction mixture was passed through a short pad of celite and washed with CH_2Cl_2. The solvents were evaporated under reduced pressure to give the crude mixture, which was purified by flash column chromatography on silica gel (petroleum ether/ethyl acetate = 40:1 to 15:1) to afford

the title compound **6a** as a white solid (80.1 mg, 80% yield, >20/1 dr), 97% ee [Daicel Chiralpak OD-H (0.46 cm x 25 cm), *n*-hexane/2-propanol = 95/5, v = 1.0 mL·min^{-1}, λ = 254 nm, t (minor) = 6.58 min, t (major) = 9.66 min]; $[\alpha]_D^{25}$ = -108.4 (c = 1.0, $CHCl_3$). ^1H NMR (400 MHz, $CDCl_3$): δ = 8.65 (s, 1H), 8.15 – 8.04 (m, 1H), 7.73 – 7.63 (m, 2H), 7.55 – 7.45 (m, 1H), 7.43 – 7.26 (m, 9H), 7.25 – 7.07 (m, 4H), 7.06 – 6.96 (m, 1H), 6.72 – 6.52 (m, 2H), 5.22 – 5.11 (m, 1H), 4.72 – 4.63 (m, 1H), 1.18 (s, 9H). ^{13}C NMR (100 MHz, $CDCl_3$): δ = 170.7, 170.6, 141.9, 139.7, 136.3, 135.8, 123.0, 129.0, 128.7, 128.2, 128.0, 127.9, 127.8, 127.6, 126.1, 124.3, 121.3, 118.9, 118.8, 114.8, 110.9, 81.1, 71.2, 45.9, 27.5.

The following compounds **6b**–**6g** were prepared analogously.

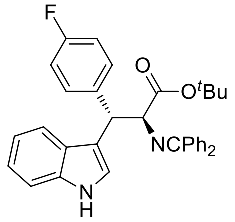

6b. White solid, 90.2 mg, 87% yield, >20/1 dr, 93% ee [Daicel Chiralpak OD-H (0.46 cm x 25 cm), *n*-hexane/2-propanol = 95/5, v = 0.6 mL·min^{-1}, λ = 254 nm, t (minor) = 6.71 min, t (major) = 11.38 min]; $[\alpha]_D^{25}$ = -51.2 (c = 1.0, $CHCl_3$). ^1H NMR (400 MHz, C_3D_6O): δ = 10.13 (s, 1H), 7.91 (d, J = 2.3 Hz, 1H), 7.61 – 7.56 (m, 2H), 7.45 – 7.36 (m, 6H), 7.36 – 7.27 (m, 4H), 7.08 – 7.02 (m, 1H), 6.99 – 6.88 (m, 3H), 6.66 (d, J = 6.5 Hz, 2H), 5.14 (d, J = 5.5 Hz, 1H), 4.58 (d, J = 5.5 Hz, 1H), 1.17 (s, 9H). ^{13}C NMR (100 MHz, C_3D_6O): δ = 170.8, 170.2, 162.1 (d, $J_{F\text{-}C}$ = 241.1 Hz), 140.4, 139.4 (d, $J_{F\text{-}C}$ = 3.1 Hz), 137.0, 137.0, 131.4 (d, $J_{F\text{-}C}$ = 7.9 Hz), 130.9, 129.2, 129.0, 128.7, 128.6, 128.2, 124.9, 121.9, 119.4, 119.2, 115.3, 115.0 (d, $J_{F\text{-}C}$ = 21.0 Hz), 111.8, 81.0, 72.1, 46.1, 27.7. ^{19}F NMR (376 MHz, C_3D_6O): δ = -118.6. IR (thin film): ν_{max} (cm^{-1}) = 3358, 3058, 2974, 1719, 1507, 1457, 1221, 1155, 742, 697; HRMS (ESI) calcd for $C_{34}H_{31}FN_2O_2Na$ $[M+Na]^+$: 541.2262. Found: 541.2263.

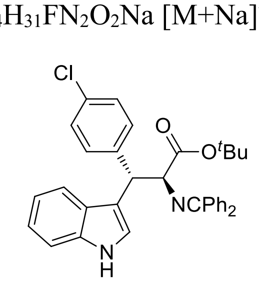

6c[9]. White solid, 83.4 mg, 78% yield, >20/1 dr, 93% ee [Daicel Chiralpak OD-H (0.46 cm x 25 cm), *n*-hexane/2-propanol = 94/6, v = 1.0 mL·min^{-1}, λ = 254 nm, t (minor) = 11.43 min, t

(major) = 17.41 min]. ^{1}H NMR (400 MHz, $CDCl_3$): δ = 8.59 (s, 1H), 8.07 (d, *J* = 1.5 Hz, 1H), 7.63 (d, *J* = 7.4 Hz, 2H), 7.42 – 7.27 (m, 8H), 7.20 – 7.09 (m, 5H), 6.99 (t, *J* = 7.3 Hz, 1H), 6.65 (d, *J* = 5.3 Hz, 2H), 5.18 – 5.00 (m, 1H), 4.62 (d, *J* = 2.8 Hz, 1H), 1.16 (s, 9H). ^{13}C NMR (100 MHz, $CDCl_3$): δ = 171.24, 170.23, 140.6, 139.5, 136.1, 135.8, 131.8, 130.3, 130.2, 128.8, 128.4, 128.2, 127.9, 127.7, 127.6, 124.3, 121.6, 119.0, 118.8, 114.4, 111.0, 81.3, 70.9, 45.3, 27.6.

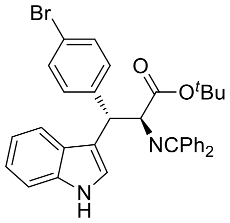

6d[9]. White solid, 81.4 mg, 70% yield, >20/1 dr, 92% ee [Daicel Chiralpak OD-H (0.46 cm x 25 cm), *n*-hexane/2-propanol = 95/5, *v* = 1.0 mL·min^{-1}, λ = 254 nm, t (minor) = 6.76 min, t (major) = 11.38 min]. ^{1}H NMR (400 MHz, $CDCl_3$): δ = 8.22 (s, 1H), 7.97 (d, *J* = 1.8 Hz, 1H), 7.61 – 7.53 (m, 2H), 7.40 – 7.26 (m, 10H), 7.14 – 7.07 (m, 3H), 6.96 (t, *J* = 7.6 Hz, 1H), 6.60 (d, *J* = 6.0 Hz, 2H), 5.15 – 4.98 (m, 1H), 4.63 – 4.53 (m, 1H), 1.16 (s, 9H). ^{13}C NMR (100 MHz, $CDCl_3$): δ = 171.1, 170.3, 141.1, 139.5, 136.1, 135.8, 130.9, 130.8, 130.2, 128.7, 128.4, 128.2, 127.9, 127.7, 127.5, 124.3, 121.6, 119.9, 119.0, 118.7, 114.3, 111.0, 81.3, 70.9, 45.4, 27.6.

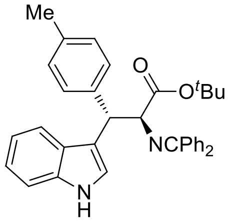

6e. White solid, 79.2 mg, 77% yield, >20/1 dr, 94% ee [Daicel Chiralpak OD-H (0.46 cm x 25 cm), *n*-hexane/2-propanol = 95/5, *v* = 0.6 mL·min^{-1}, λ = 254 nm, t (minor) = 6.77 min, t (major) = 11.52 min]; $[\alpha]_D^{25}$ = -92.1 (c = 1.0, $CHCl_3$). ^{1}H NMR (400 MHz, $CDCl_3$): δ = 8.08 (s, 1H), 7.90 (d, *J* = 2.2 Hz, 1H), 7.61 – 7.54 (m, 2H), 7.41 (d, *J* = 8.0 Hz, 1H), 7.37 – 7.27 (m, 6H), 7.26 – 7.24 (m, 1H), 7.13 – 7.05 (m, 3H), 6.99 – 6.92 (m, 3H), 6.55 (d, *J* = 6.0 Hz, 2H), 5.04 (d, *J* = 4.9 Hz, 1H), 4.56 (d, *J* = 5.1 Hz, 1H), 2.24 (s, 3H), 1.15 (s, 9H). ^{13}C NMR (100 MHz, $CDCl_3$): δ = 170.8, 139.7, 138.9, 136.3, 135.8, 135.3, 130.0, 128.9, 128.7, 128.5, 128.2, 128.0, 127.8, 127.7, 124.3, 121.2, 118.8, 118.6, 114.7, 110.9, 81.1, 71.2, 45.5, 27.5, 20.9. IR

(thin film): ν_{max} (cm^{-1}) = 3360, 2976, 2928, 1717, 1627, 1456, 1367, 1155; 741, 702; HRMS (ESI) calcd for $C_{35}H_{34}N_2O_2Na$ $[M+Na]^+$: 537.2512. Found: 537.2512.

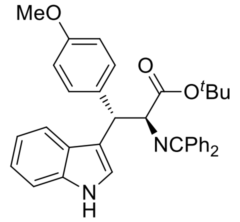

6f[9]. White solid, 75.3 mg, 71% yield, >20/1 dr, 90% ee [Daicel Chiralpak OD-H (0.46 cm x 25 cm), *n*-hexane/2-propanol = 95/5, *v* = 1.0 mL·min^{-1}, λ = 254 nm, t (minor) = 9.45 min, t (major) = 14.55 min]. ^{1}H NMR (400 MHz, $CDCl_3$): δ = 8.56 (s, 1H), 8.08 – 8.02 (m, 1H), 7.66 (d, *J* = 7.4 Hz, 2H), 7.47 (d, *J* = 7.7 Hz, 1H), 7.43 – 7.27 (m, 7H), 7.18 (d, *J* = 7.7 Hz, 2H), 7.11 (t, *J* = 7.3 Hz, 1H), 7.00 (t, *J* = 7.2 Hz, 1H), 6.75 (d, *J* = 8.0 Hz, 2H), 6.71 – 6.57 (m, 2H), 5.21 – 5.03 (m, 1H), 4.71 – 4.49 (m, 1H), 3.75 (s, 3H), 1.17 (s, 9H). ^{13}C NMR (100 MHz, $CDCl_3$): δ = 170.6, 157.9, 139.7, 136.4, 135.8, 134.2, 129.9, 128.7, 128.2, 128.0, 127.9, 127.8, 127.7, 124.1, 121.3, 119.0, 118.8, 115.2, 113.2, 110.8, 81.0, 71.3, 55.2, 45.2, 27.6.

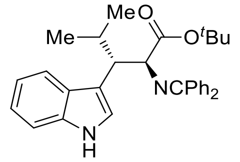

6g[9]. White solid, 67.2 mg, 72% yield, >20/1 dr, 85% ee [Daicel Chiralpak IC (0.46 cm x 25 cm), *n*-hexane/2-propanol = 95/5, *v* = 0.6 mL·min^{-1}, λ = 254 nm, t (minor) = 7.34 min, t (major) = 12.39 min]. ^{1}H NMR (400 MHz, $CDCl_3$): δ = 8.70 (s, 1H), 7.96 – 7.90 (m, 1H), 7.89 – 7.79 (m, 2H), 7.67 – 7.57 (m, 1H), 7.53 – 7.46 (m, 3H), 7.44 – 7.35 (m, 3H), 7.35 – 7.26 (m, 3H), 7.14 – 7.02 (m, 2H), 4.44 (d, *J* = 3.3 Hz, 1H), 3.32 (d, *J* = 5.9 Hz, 1H), 2.13 – 1.99 (m, 1H), 1.02 (s, 9H), 0.93 (d, *J* = 6.5 Hz, 3H), 0.80 (d, *J* = 6.7 Hz, 3H). ^{13}C NMR (100 MHz, $CDCl_3$): δ = 171.7, 170.6, 140.1, 136.9, 135.3, 130.1, 129.2, 128.8, 128.6, 128.3, 128.0, 127.8, 123.6, 120.7, 119.0, 118.3, 115.7, 110.9, 80.8, 67.9, 46.2, 32.3, 27.3, 21.5, 21.2.

2.6. Representative procedure for the syntheses of compounds 7.

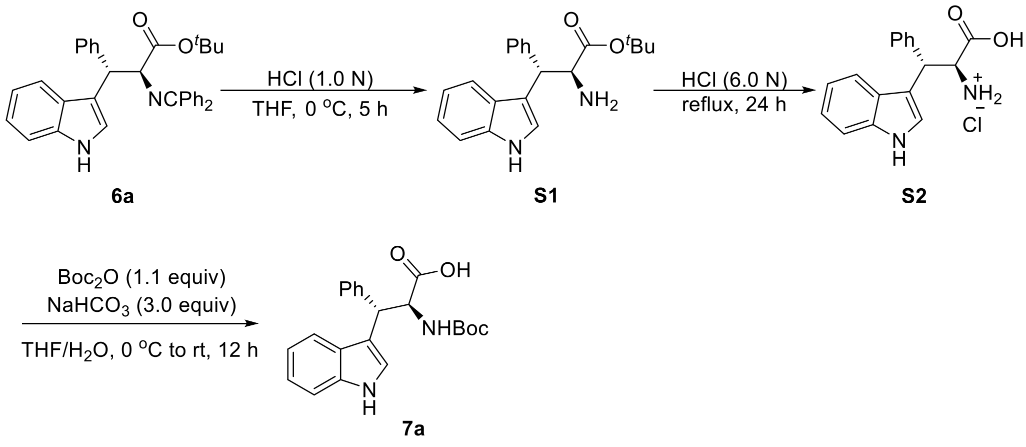

Preparation of compound 7a. Step 1: Compound **6a** (100.0 mg, 0.2 mmol) was dissolved in THF (2.0 mL) and aq. HCl (1 N, 2.0 mL) was added at 0 °C. After stirring for 5 h, the reaction mixture was quenched with sat. aq. $NaHCO_3$ and extracted with EtOAc for three times. The organic layers were combined and dried over anhydrous Na_2SO_4. The solvents were evaporated under reduced pressure to give the crude mixture, which was purified by flash column chromatography on silica gel (CH_2Cl_2/MeOH = 100:1 to 20:1) to afford the compound **S1**, which was directly used for the next step.

Step 2: To a reaction flask with refluxing condenser were added compound **S1** and aq. HCl (6 N, 10.0 mL) under argon. The reaction mixture was heated to reflux for 24 h. After that, the reaction mixture was cooled, and the solvents were removed in vacuo to give crude compound **S2**, which was used for the next step without further purification.

Step 3: Crude compound **S2** was dissolved in THF/H_2O (2/1, 6.0 mL). Then $NaHCO_3$ (50.4 mg, 0.6 mmol, 3.0 equiv), and Boc_2O (48.0 mg, 0.22 mmol, 1.1 equiv) was added to the resulting solution at 0 °C. The reaction mixture was stirred for 12 h at ambient temperature. After that, the pH of the aqueous layer was adjusted to 3 upon addition of aq. HCl (1 N) and the resulting mixture was extracted with EtOAc for three times. The organic layers were combined and dried over anhydrous Na_2SO_4. The solvents were evaporated under reduced pressure to give the crude mixture, which was purified by flash column chromatography on silica gel (CH_2Cl_2/MeOH = 100:1 to 30:1) to afford compound **7a** as a pale yellow solid (31.2 mg, 41% yield over 3 steps); $[\alpha]_D^{25}$ = +45.9 (c = 1.0, $CHCl_3$). ^{1}H NMR (400 MHz, C_3D_6O): δ = 10.19 (s, 1H), 7.49 – 7.36 (m, 5H), 7.24 (t, J = 7.3 Hz, 2H), 7.19 – 7.12 (m, 1H), 7.06 (t, J = 7.6 Hz, 1H), 6.92 (t, J = 7.5 Hz, 1H), 6.17 (d, J = 8.5 Hz, 1H), 5.04 (t, J = 8.8 Hz, 1H), 4.84 (d,

J = 8.6 Hz, 1H), 1.35 (s, 9H). ^{13}C NMR (100 MHz, C_3D_6O): δ = 173.0, 156.0, 142.3, 137.3, 129.2, 128.6, 128.0, 127.0, 122.9, 121.9, 119.5, 119.2, 114.9, 111.8, 79.1, 58.3, 45.8, 28.2. IR (thin film): ν_{max} (cm^{-1}) = 3416, 3339, 3059, 2979, 1717, 1654, 1406, 1368, 1161, 741; HRMS (ESI) calcd for $C_{22}H_{24}N_2O_4Na$ [M+Na]$^+$: 403.1628. Found: 403.1630.

The following substrates **7b**–**7g** were prepared analogously

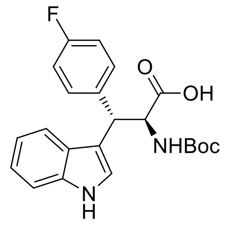

7b. Pale yellow solid, 35.0 mg, 44% yield over 3 steps; $[\alpha]_D^{25}$ = +39.4 (c = 1.0, $CHCl_3$). ^{1}H NMR (500 MHz, C_3D_6O): δ = 10.21 (s, 1H), 7.48 (s, 1H), 7.47 – 7.42 (m, 2H), 7.38 (t, J = 7.6 Hz, 2H), 7.08 (t, J = 7.6 Hz, 1H), 7.00 (t, J = 8.8 Hz, 2H), 6.93 (t, J = 7.5 Hz, 1H), 6.22 (d, J = 8.9 Hz, 1H), 5.02 (t, J = 8.8 Hz, 1H), 4.85 (d, J = 8.7 Hz, 1H), 1.35 (s, 9H). ^{13}C NMR (125 MHz, C_3D_6O): δ = 172.8, 162.0 (d, $J_{F\text{-}C}$ = 241.4 Hz), 155.9, 138.2 (d, $J_{F\text{-}C}$ = 3.1 Hz), 137.2, 130.9 (d, $J_{F\text{-}C}$ = 7.9 Hz), 127.8, 122.8, 121.9, 119.3, 119.2, 115.1 (d, $J_{F\text{-}C}$ = 21.3 Hz), 114.6, 111.8, 79.1, 58.3, 44.9, 28.1. ^{19}F NMR (376 MHz, $CDCl_3$): δ = -118.1. IR (thin film): ν_{max} (cm^{-1}) = 3415, 3357, 3060, 2980, 1717, 1508, 1224, 1159, 741; HRMS (ESI) calcd for $C_{22}H_{23}FN_2O_4Na$ [M+Na]$^+$: 421.1534. Found: 421.1536.

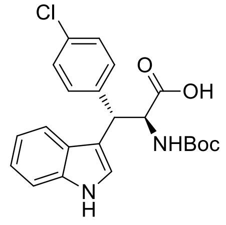

7c. Pale yellow solid, 33.2 mg, 40% yield over 3 steps; $[\alpha]_D^{25}$ = +36.6 (c = 1.0, $CHCl_3$). ^{1}H NMR (400 MHz, C_3D_6O): δ = 10.23 (s, 1H), 7.48 (s, 1H), 7.45 – 7.33 (m, 4H), 7.26 (d, J = 8.2 Hz, 2H), 7.07 (t, J = 7.5 Hz, 1H), 6.92 (t, J = 7.5 Hz, 1H), 6.23 (d, J = 8.7 Hz, 1H), 5.01 (t, J = 8.7 Hz, 1H), 4.84 (d, J = 8.7 Hz, 1H), 1.34 (s, 9H). ^{13}C NMR (100 MHz, C_3D_6O): δ = 172.8, 156.1, 141.4, 137.4, 132.4, 131.0, 128.7, 127.9, 123.1, 122.1, 119.4, 114.5, 112.0, 79.2, 58.3, 45.3, 28.3. IR (thin film): ν_{max} (cm^{-1}) = 3417, 3059, 2980, 1717, 1491, 1369, 1160, 741; HRMS (ESI) calcd for $C_{22}H_{23}ClN_2O_4Na$ [M+Na]$^+$: 437.1239. Found: 437.1241.

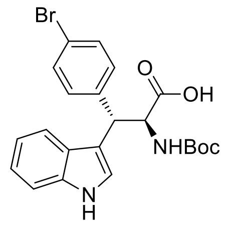

7d. Pale yellow solid, 44.0 mg, 48% yield over 3 steps; $[\alpha]_D^{25}$ = +12.4 (c = 1.0, $CHCl_3$). ^1H NMR (400 MHz, C_3D_6O): δ = 10.22 (s, 1H), 7.48 (s, 1H), 7.45 – 7.33 (m, 7H), 7.12 – 7.00 (m, 1H), 6.99 – 6.87 (m, 1H), 6.22 (d, *J* = 9.0 Hz, 1H), 5.02 (t, *J* = 8.8 Hz, 1H), 4.83 (d, *J* = 8.7 Hz, 1H), 1.34 (s, 9H). ^{13}C NMR (100 MHz, C_3D_6O): δ = 172.7, 156.0, 141.8, 137.3, 131.6, 131.3, 127.8, 123.0, 122.1, 120.5, 119.4, 119.3, 114.4, 111.9, 79.2, 58.1, 45.3, 28.2. IR (thin film): ν_{max} (cm^{-1}) = 3416, 3058, 2980, 1718, 1489, 1369, 1160, 741; HRMS (ESI) calcd for $C_{22}H_{23}BrN_2O_4Na$ $[M+Na]^+$: 481.0733. Found: 481.0735.

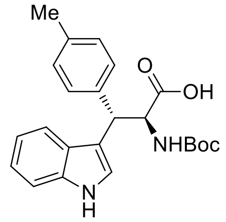

In lieu of standard reaction conditions of step 2 in the reaction sequence, the reaction was performed with 6 N HCl (10 mL) and 1,4-dioxane (2 mL) under reflux. **7e**. Pale yellow solid, 36.2 mg, 46% yield over 3 steps; $[\alpha]_D^{25}$ = +12.2 (c = 1.0, $CHCl_3$). ^1H NMR (400 MHz, C_3D_6O): δ = 10.18 (s, 1H), 7.43 (s, 1H), 7.41 – 7.34 (m, 2H), 7.29 (d, *J* = 7.8 Hz, 2H), 7.13 – 7.01 (m, 3H), 6.91 (t, *J* = 7.5 Hz, 1H), 6.15 (d, *J* = 8.7 Hz, 1H), 5.00 (t, *J* = 8.9 Hz, 1H), 4.78 (d, *J* = 8.8 Hz, 1H), 2.23 (s, 3H), 1.34 (s, 9H). ^{13}C NMR (100 MHz, C_3D_6O): δ = 173.0, 156.1, 139.3, 137.4, 136.4, 129.3, 129.2, 128.1, 122.9, 122.0, 119.6, 119.3, 115.2, 111.9, 79.1, 58.4, 45.5, 28.3, 20.8. IR (thin film): ν_{max} (cm^{-1}) = 3419, 3056, 2979, 1717, 1512, 1395, 1369, 1160, 740; HRMS (ESI) calcd for $C_{23}H_{26}N_2O_4Na$ $[M+Na]^+$: 417.1785. Found: 417.1786.

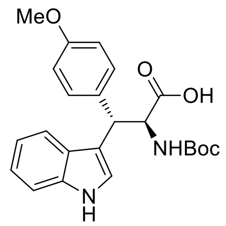

In lieu of standard reaction conditions of step 2 in the reaction sequence, the reaction was performed with 6 N HCl (10 mL) and 1,4-dioxane (2 mL) under reflux. **7f**. Pale yellow solid,

32.0 mg, 39% yield over 3 steps; $[\alpha]_D^{25}$ = +27.9 (c = 1.0, $CHCl_3$). ^{1}H NMR (500 MHz, C_3D_6O): δ = 10.15 (s, 1H), 7.43 (s, 1H), 7.40 – 7.25 (m, 4H), 7.05 (t, *J* = 7.5 Hz, 1H), 6.91 (t, *J* = 7.2 Hz, 1H), 6.79 (d, *J* = 7.6 Hz, 2H), 6.24 – 6.00 (m, 1H), 5.07 – 4.90 (m, 1H), 4.83 – 4.68 (m, 1H), 3.71 (s, 3H), 1.34 (s, 9H). ^{13}C NMR (125 MHz, C_3D_6O): δ = 173.1, 159.1, 137.5, 134.3, 130.2, 128.1, 122.9, 122.0, 119.7, 119.2, 115.4, 114.0, 111.9, 79.1, 58.6, 55.1, 45.1, 28.3. IR (thin film): ν_{max} (cm^{-1}) = 3415, 3059, 2978, 1717, 1510, 1248, 1175, 1161; HRMS (ESI) calcd for $C_{23}H_{26}N_2O_5Na$ $[M+Na]^+$: 433.1734. Found: 433.1735.

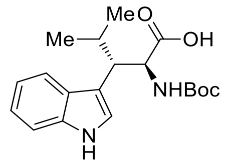

7g. White solid, 41.0 mg, 59% yield over 3 steps; $[\alpha]_D^{25}$ = +39.7 (c = 1.0, $CHCl_3$). ^{1}H NMR (400 MHz, C_3D_6O): δ = 10.17 (s, 1H), 7.63 (d, *J* = 8.0 Hz, 1H), 7.38 (d, *J* = 8.1 Hz, 1H), 7.25 – 7.18 (m, 1H), 7.08 (t, *J* = 7.5 Hz, 1H), 7.00 (t, *J* = 7.5 Hz, 1H), 5.29 (d, *J* = 9.5 Hz, 1H), 4.88 – 4.74 (m, 1H), 3.34 (dd, *J* = 9.6, 4.1 Hz, 1H), 2.31 – 2.19 (m, 1H), 1.41 (s, 9H), 1.17 (d, *J* = 6.5 Hz, 3H), 0.82 (d, *J* = 6.6 Hz, 3H). ^{13}C NMR (100 MHz, C_3D_6O): δ = 173.7, 156.3, 137.3, 128.7, 123.6, 121.8, 119.9, 119.3, 113.2, 111.9, 79.2, 67.3, 55.8, 47.0, 28.2, 21.6, 21.1. IR (thin film): ν_{max} (cm^{-1}) = 3380, 2961, 2927, 1719, 1649, 1403, 1160; HRMS (ESI) calcd for $C_{19}H_{26}N_2O_4Na$ $[M+Na]^+$: 369.1785. Found: 369.1786.

2.7 Representative procedure for the syntheses of substrates 10.

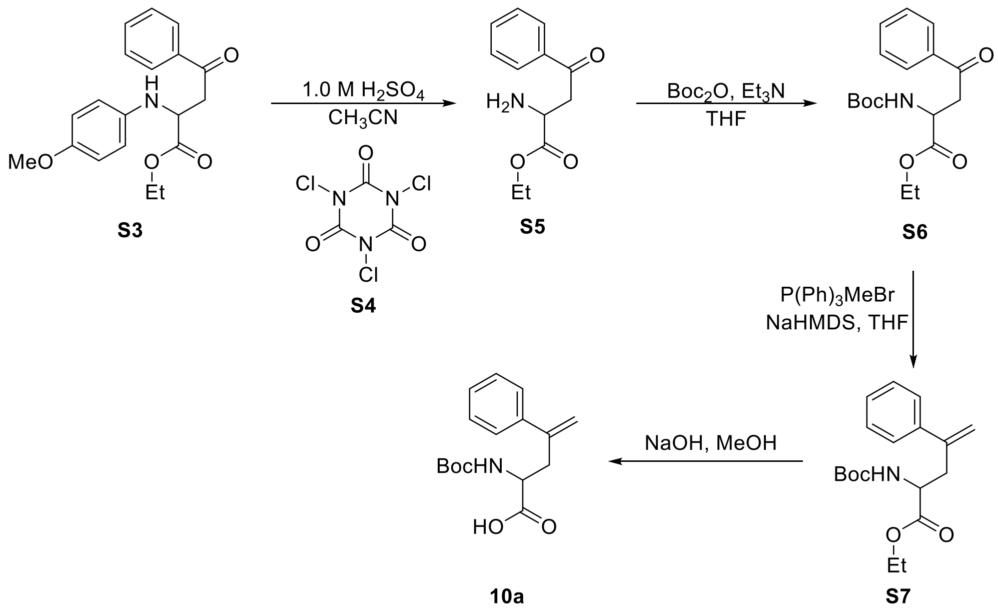

Preparation of compound 10a.[10−12] **Step 1**:[11] Compound **S3**[10] (1.64 g, 5.0 mmol, 1.0 equiv) was dissolved in acetonitrile (45 mL). Then water (15 mL), reagent **S4** (1.16 g, 5.0 mmol, 1.0 equiv) and aq. H_2SO_4 (1.0 M, 5 mL) were added at 0 °C. The resulting mixture was allowed to stir at ambient temperature for 12 h. After that, acetonitrile was evaporated under reduced pressure to give the crude mixture, which was washed with CH_2Cl_2 (20 mL × 3). The pH of the resulting aqueous solution was adjusted to 8.0 through addition of sat. aq. $NaHCO_3$ solution. The aqueous solution was extracted with EtOAc (30 mL × 3). The combined organic layers were dried over Na_2SO_4 and concentrated in vacuo to give compound **S5**, which was used for the next step without further purification.

Step 2: Compound **S5** was dissolved in THF (20 mL). Boc_2O (3.5 mL, 15.0 mmol, 3.0 equiv) and Et_3N (2.1 mL, 15.0 mmol, 3.0 equiv) were added at 0 °C and the resulting mixture was allowed to stir at ambient temperature for 12 h. After that, the solvents were evaporated under reduced pressure to give the crude mixture, which was purified by flash column chromatography on silica gel (petroleum ether/ethyl acetate = 50:1 to 10:1) to give compound **S6** (963.0 mg).

Step 3:[12] NaHMDS (2.3 mL, 2 M solution in THF, 1.5 equiv) was added to a stirring suspension of Ph_3PCH_3Br (1.60 g, 4.5 mmol, 1.5 equiv) in THF (15 mL) at 0 °C under a nitrogen atmosphere. The resulting yellow mixture was stirred at 0 °C for 0.5 h. Then, a solution of **S6** (963.0 mg, 3.0 mmol, 1.0 equiv) in THF (10.0 mL) was added dropwise at 0 °C. The mixture was stirred and warmed to room temperature. After the reaction was complete, the reaction mixture was quenched with sat. aq. NH_4Cl and diluted with H_2O. The aqueous layer was extracted with EtOAc (30 mL × 3). The combined organic layers were washed with brine, dried over Na_2SO_4, and concentrated to give the crude mixture, which was purified by flash chromatography on silica gel (petroleum ether/ethyl acetate = 50:1 to 10:1) to provide the title compound **S7** (622.1 mg).

Step 4: Compound **S7** (622.1 mg, 1.95 mmol, 1.0 equiv) was dissolved in MeOH (10 mL). Then aq. NaOH (2.9 mL, 2 M solution in H_2O, 3.0 equiv) was added at 0 °C and the resulting mixture was allowed to stir at ambient temperature for 3 h. After the reaction was complete (monitored by TLC), MeOH was evaporated under reduced pressure to give the crude mixture. The pH of the resulting aqueous solution was adjusted to 2.0 through addition of aq. HCl (1 N).

The aqueous solution was extracted with EtOAc (30 mL × 3). The combined organic layers were dried over anhydrous Na_2SO_4, and concentrated in vacuo to give the crude mixture, which was purified by flash column chromatography on silica gel (DCM/MeOH = 100:1 to 30:1) to afford the title compound **10a** as white solid (509.4 mg, 35% yield over 4 steps). ^{1}H NMR (400 MHz, C_2D_6OS, 80 °C): δ = 7.46 – 7.40 (m, 2H), 7.38 – 7.32 (m, 2H), 7.31 – 7.25 (m, 1H), 6.47 (s, 1H), 5.35 (d, J = 1.2 Hz, 1H), 5.17 (d, J = 1.1 Hz, 1H), 4.12 – 3.98 (m, 1H), 3.04 – 2.94 (m, 1H), 2.76 (dd, J = 14.8, 9.2 Hz, 1H), 1.36 (s, 9H). ^{13}C NMR (100 MHz, C_2D_6OS, 80 °C): δ = 172.8, 154.6, 143.7, 139.8, 127.8, 127.0, 125.5, 114.3, 77.7, 52.3, 36.5, 27.7. IR (thin film): ν_{max} (cm^{-1}) = 3324, 2979, 2931, 1719, 1395, 1368, 1252, 1167; HRMS (ESI) calcd for $C_{16}H_{21}NO_4Na$ $[M+Na]^+$: 314.1363. Found: 314.1356.

The following substrates **10b**–**10e** were prepared analogously.

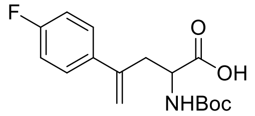

10b. White solid, 571.8 mg, 37% yield over 4 steps. ^{1}H NMR (400 MHz, C_2D_6OS, 80 °C): δ = 7.15 – 6.89 (m, 2H), 6.79 – 6.58 (m, 2H), 6.03 (s, 1H), 4.87 (d, J = 0.5 Hz, 1H), 4.71 (s, 1H), 3.67 – 3.51 (m, 1H), 2.51 (dd, J = 14.5, 4.6 Hz, 1H), 2.30 (dd, J = 14.7, 9.4 Hz, 1H), 0.90 (s, 9H). ^{13}C NMR (100 MHz, C_2D_6OS, 80 °C): δ = 172.7, 161.4 (d, $J_{F\text{-}C}$ = 243.1 Hz), 154.6, 142.8, 136.2 (d, $J_{F\text{-}C}$ = 2.9 Hz), 127.5 (d, $J_{F\text{-}C}$ = 8.0 Hz), 114.50 (d, $J_{F\text{-}C}$ = 21.2 Hz), 114.49, 77.7, 52.3, 36.6, 27.7. ^{19}F NMR (376 MHz, $CDCl_3$): δ = -114.4. IR (thin film): ν_{max} (cm^{-1}) = 3316, 2980, 2934, 1720, 1509, 1396, 1369, 1235, 1164, 841; HRMS (ESI) calcd for $C_{16}H_{20}FNO_4Na$ $[M+Na]^+$: 332.1269. Found: 332.1270.

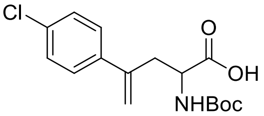

10c. White solid, 552.5 mg, 34% yield over 4 steps. ^{1}H NMR (400 MHz, C_2D_6OS, 80 °C): δ = 7.50 – 7.40 (m, 2H), 7.40 – 7.32 (m, 2H), 6.45 (s, 1H), 5.37 (s, 1H), 5.19 (s, 1H), 4.15 – 3.90 (m, 1H), 2.97 (dd, J = 14.6, 4.4 Hz, 1H), 2.74 (dd, J = 14.5, 9.4 Hz, 1H), 1.35 (s, 9H). ^{13}C NMR (100 MHz, C_2D_6OS, 80 °C): δ = 172.8, 154.6, 142.7, 138.7, 131.8, 127.8, 127.3, 115.1, 77.7, 52.4, 36.5, 27.7. IR (thin film): ν_{max} (cm^{-1}) = 3313, 2979, 2933, 1718, 1493, 1395, 1368, 1252, 1167, 836; HRMS (ESI) calcd for $C_{16}H_{20}ClNO_4Na$ $[M+Na]^+$: 348.0973. Found: 348.0973.

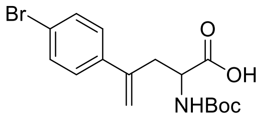

10d. White solid, 645.8 mg, 35% yield over 4 steps. ^{1}H NMR (400 MHz, C_2D_6OS, 80 °C): δ = 7.56 – 7.48 (m, 2H), 7.42 – 7.34 (m, 2H), 6.49 (s, 1H), 5.38 (d, *J* = 1.0 Hz, 1H), 5.20 (d, *J* = 1.0 Hz, 1H), 4.09 – 3.96 (m, 1H), 3.03 – 2.90 (m, 1H), 2.75 (dd, *J* = 14.7, 9.4 Hz, 1H), 1.35 (s, 9H). ^{13}C NMR (100 MHz, C_2D_6OS, 80 °C): δ = 172.7, 154.6, 142.7, 139.0, 130.7, 127.7, 120.3, 115.2, 77.7, 52.2, 36.3, 27.7. IR (thin film): ν_{max} (cm^{-1}) = 3326, 2978, 2933, 1719, 1490, 1394, 1368, 1252, 1163; HRMS (ESI) calcd for $C_{16}H_{20}BrNO_4Na$ $[M+Na]^+$: 392.0468. Found: 392.0467.

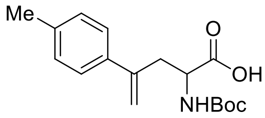

10e. White solid, 457.8 mg, 30% yield over 4 steps. ^{1}H NMR (400 MHz, C_2D_6OS, 80 °C): δ = 7.39 – 7.25 (m, 2H), 7.21 – 7.11 (m, 2H), 6.46 (s, 1H), 5.31 (d, *J* = 1.3 Hz, 1H), 5.12 (d, *J* = 1.1 Hz, 1H), 4.11 – 3.95 (m, 1H), 3.06 – 2.88 (m, 1H), 2.73 (dd, *J* = 14.6, 9.3 Hz, 1H), 2.31 (s, 3H), 1.35 (s, 9H). ^{13}C NMR (100 MHz, C_2D_6OS, 80 °C): δ = 172.8, 154.6, 143.5, 136.8, 136.3, 128.4, 125.4, 113.5, 77.7, 52.3, 36.5, 27.7, 20.1. IR (thin film): ν_{max} (cm^{-1}) = 3318, 2979, 2931, 1720, 1514, 1396, 1368, 1254, 1167, 824; HRMS (ESI) calcd for $C_{17}H_{23}NO_4Na$ $[M+Na]^+$: 328.1519. Found: 328.1514.

2.8 Procedure for the synthesis of substrate 10h.

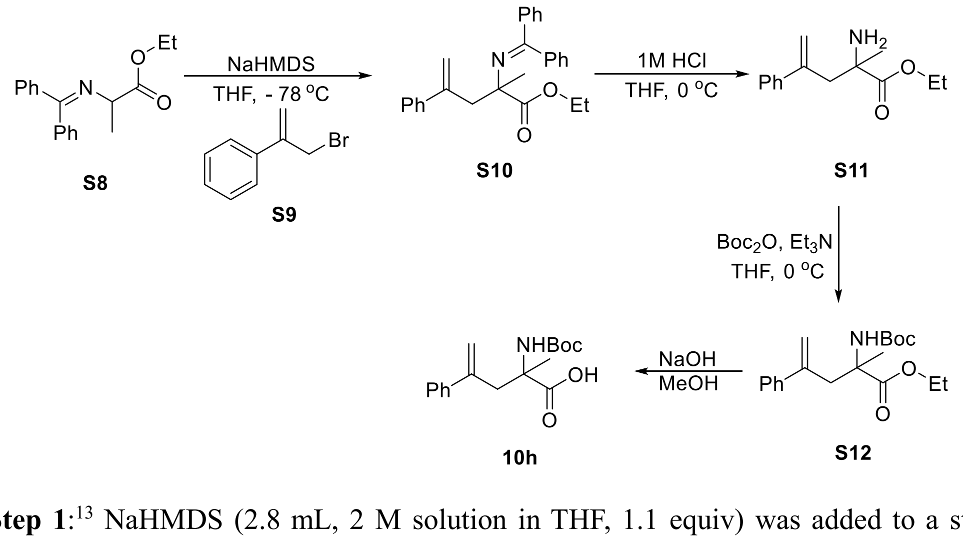

Step 1:[13] NaHMDS (2.8 mL, 2 M solution in THF, 1.1 equiv) was added to a stirring solution of compound **S8** (1.41 g, 5.0 mmol, 1.0 equiv) in THF (5 mL) at -78 °C under a nitrogen

atmosphere. The resulting reaction mixture was stirred at -78 ºC for 1 h. Then, a solution of **S9** (1.18 g, 6.0 mmol, 1.2 equiv) in THF (10.0 mL) was added dropwise at -78 ºC. The reaction mixture was stirred at -78 °C for 30 minutes and then allowed to warm to room temperature and stirred for 12 h. After the reaction was complete (monitored by TLC), the reaction mixture was quenched with sat. aq. NH_4Cl solution and diluted with H_2O. The aqueous solution was extracted with EtOAc (30 mL × 3). The combined organic layers were washed with brine and dried over Na_2SO_4, concentrated and purified by flash chromatography on silica gel (petroleum ether/ethyl acetate = 50:1 to 10:1) to give compound **S10** (1.45 g).

Step 2: Compound **S10** (1.45 g, 3.65 mmol, 1.0 equiv) was dissolved in THF (20.0 mL). Then aq. HCl (1 N, 11.0 mL) was added at 0 ℃ and the resulting mixture was stirred at this temperature for 5 h. After that, THF was evaporated under reduced pressure to give the crude mixture, which was washed with Et_2O (20 mL × 3). The pH of the resulting aqueous solution was adjusted to 8.0 through addition of sat. aq. $NaHCO_3$ solution. The aqueous solution was extracted with EtOAc (30 mL × 3). The combined organic layers were dried over Na_2SO_4 and concentrated in vacuo to give the crude compound **S11**, which was used for the next step without further purification.

Step 3: Compound **S11** was dissolved in THF (20 mL). Boc_2O (2.5 mL,11.0 mmol, 3.0 equiv) and Et_3N (1.5 mL, 11.0 mmol, 3.0 equiv) were added at 0 ºC. The reaction mixture was allowed to stir at ambient temperature for 12 h. After that, the solvents were evaporated under reduced pressure to give the crude mixture, which was purified by flash column chromatography on silica gel (petroleum ether/ethyl acetate = 50:1 to 10:1) to give compound **S12** (729.7 mg).

Step 4: Compound **S12** (729.7 mg, 2.15 mmol, 1.0 equiv) was dissolved in MeOH (10 mL). Then aq. NaOH (2 M, 3.2 mL, 3.0 equiv) was added at 0 ºC and the resulting mixture was allowed to stir at ambient temperature for 3 h. After the reaction was complete (monitored by TLC), MeOH was evaporated under reduced pressure to give the crude mixture. The pH of the resulting aqueous solution was adjusted to 2.0 through addition of aq. HCl (1 N). The aqueous solution was extracted with EtOAc (30 mL × 3). The combined organic layers were dried over anhydrous Na_2SO_4, and concentrated in vacuo to give the crude mixture, which was purified by flash column chromatography on silica gel (DCM/MeOH = 100:1 to 30:1) to afford the title

compound **10h** as white solid (635.0 mg, 42% yield over 4 steps). ^{1}H NMR (400 MHz, $CDCl_3$): δ = 8.49 (br s, 1H), 7.36 – 7.26 (m, 4H), 7.25 – 7.20 (m, 1H), 5.30 (s, 1H), 5.15 (s, 1H), 5.06 (s, 1H), 3.39 – 3.04 (m, 2H), 1.48 (s, 3H), 1.37 (s, 9H). ^{13}C NMR (100 MHz, $CDCl_3$): δ = 179.1, 154.3, 144.7, 141.9, 128.2, 127.4, 126.6, 118.3, 79.5, 59.4, 41.3, 28.3, 23.8. IR (thin film): ν_{max} (cm^{-1}) = 3429, 2979, 2932, 1713, 1495, 1401, 1368, 1248, 1167; HRMS (ESI) calcd for $C_{17}H_{23}NO_4Na$ $[M+Na]^+$: 328.1519. Found: 328.1505.

2.9 Procedure for the 1.0 mmol-scale reaction for the synthesis of compound 3a.

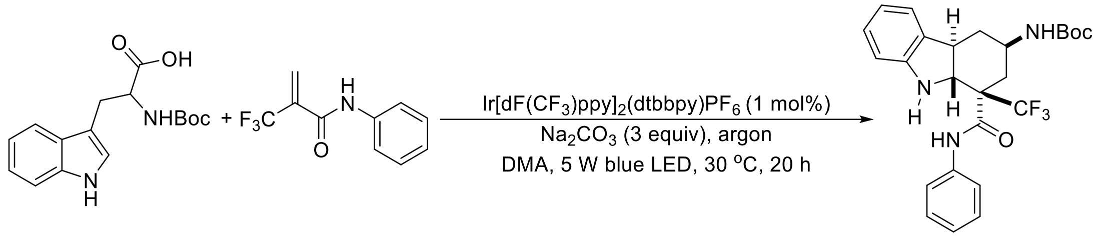

1a 2a 3a

To a Young Schlenk tube (50 mL) were added Ir[dF(CF_3)ppy]$_2$(dtbbpy)PF_6 (11.2 mg, 0.01 mmol, 1 mol%), **1a** (456.5 mg, 1.5 mmol, 1.5 equiv), **2a** (215.1 mg, 1.0 mmol, 1.0 equiv), Na_2CO_3 (318.0 mg, 3.0 mmol, 3.0 equiv), and N,N-dimethylacetamide (DMA, 20.0 mL). Subsequently, the reaction mixture was degassed through several freeze-pump-thaw cycles until no bubbles were released. The reaction mixture was stirred under inert atmosphere at 30 °C, and irradiated by a 5 W blue LED lamp (λ = 450−460 nm, the tube was placed at approximately 2 cm away from the light source). After 20 h, the reaction mixture was passed through a short pad of celite and washed with ethyl acetate. The solvents were evaporated under reduced pressure to give the crude mixture, which was purified by flash column chromatography on silica gel (petroleum ether/ethyl acetate = 40:1 to 15:1, *silica gel was soaked with a solution of petroleum ether and triethylamine (1000/1, v/v) before use*) to afford the title compound **3a** as white solid (320.0 mg, 67% yield).

2.10 Procedure for the synthesis of compound K2.

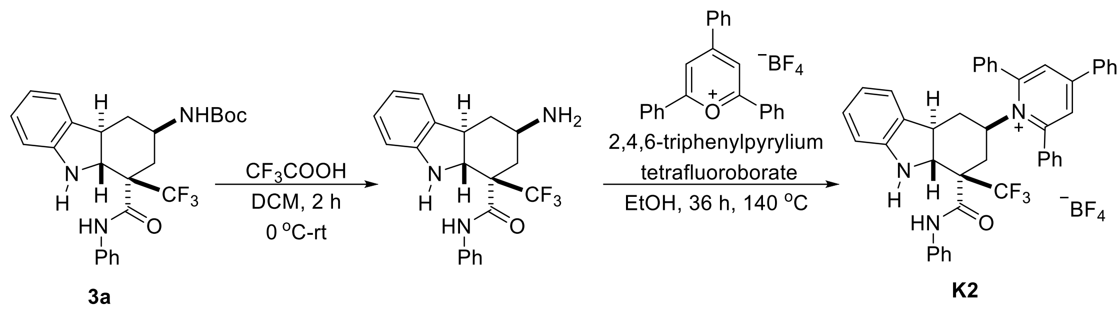

Step 1: Compound **3a** (237.8 mg, 0.5 mmol, 1.0 equiv) was dissolved in DCM (5 mL) and CF_3COOH (0.5 mL, 6.5 mmol, 13.0 equiv) was added at 0 °C. The reaction mixture was allowed to stir at ambient temperature for 2 h. After that, the solvents were evaporated under reduced pressure to give the crude mixture. The pH of the resulting mixture was adjusted to 8.0 through addition of sat. aq. $NaHCO_3$. The aqueous solution was extracted with EtOAc (10 mL × 3). The organic layers were combined and dried over anhydrous Na_2SO_4. The solvents were evaporated under reduced pressure to give the crude mixture, which was purified by flash column chromatography on silica gel (CH_2Cl_2/MeOH = 100:1 to 20:1, *silica gel was soaked with a solution of petroleum ether and triethylamine (1000/1, v/v) before use*) to give the crude amine product, which was used for the next step.

Step 2:[14] To a Young Schlenk tube (100 mL) were added the crude amine product from **Step 1**, 2,4,6-triphenylpyrylium tetrafluoroborate (495.1 mg,1.25 mmol) and EtOH (20 mL) under N_2 atmosphere. The reaction mixture was stir at 140 °C for 36 h. After that, the solvents were evaporated under reduced pressure to give the crude mixture, which was purified by flash column chromatography on silica gel (DCM/MeOH = 200:1 to 30:1) to afford the title compound **K2** as green solid (258.0 mg, 68% yield over 2 steps). ^{1}H NMR (400 MHz, $CDCl_3$): δ = 10.08 (s, 1H), 8.17 – 7.79 (m, 4H), 7.75 – 7.65 (m, 3H), 7.62 – 7.40 (m, 10H), 7.28 – 7.19 (m, 5H), 7.15 – 7.03 (m, 2H), 6.91 – 6.80 (m, 2H), 5.21 – 5.01 (m, 1H), 4.42 (s, 1H), 3.25 (d, J = 12.0 Hz, 1H), 3.06 (d, J = 13.2 Hz, 1H), 2.81 (d, J = 11.2 Hz, 1H), 2.44 (t, J = 12.2 Hz, 1H), 2.11 (app q, J = 12.3 Hz, 1H), 1.80 (t, J = 12.5 Hz, 1H). ^{13}C NMR (100 MHz, $CDCl_3$): δ = 161.5, 155.7, 147.9, 136.7, 133.6, 133.0, 132.2, 131.0, 129.6, 129.0, 128.7, 128.3, 128.2, 125.0, 124.6 (q, $J_{F\text{-}C}$ = 284.0 Hz), 123.4, 122.3, 120.2, 112.3, 65.9, 65.5, 54.8 (q, $J_{F\text{-}C}$ = 23.8 Hz), 43.5, 33.9, 32.4. ^{19}F NMR (376 MHz, $CDCl_3$): δ = -72.0, -152.28, -152.34. IR (thin film): ν_{max} (cm^{-1}) = 3341, 3055, 1687, 1619, 1560, 1236, 1166; HRMS (ESI) calcd for $C_{43}H_{35}F_3N_3O$ [M]$^+$:

666.2727. Found: 666.2804.

2.11 Procedure for the synthesis of compound 12.[14]

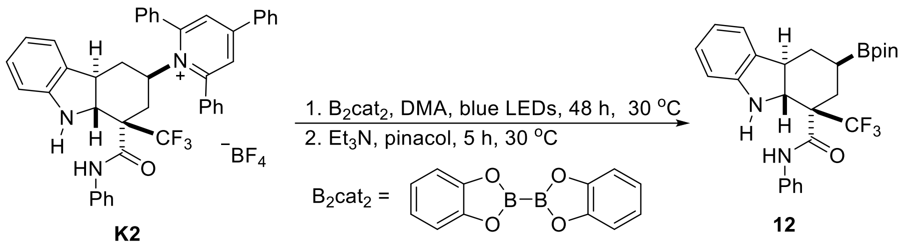

To a Young Schlenk tube (10 mL) were added **K2** (150.7 mg, 0.2 mmol, 1.0 equiv), B_2cat_2 (142.7 mg, 0.6 mmol, 3.0 equiv) and N,N-dimethylacetamide (DMA, 1.0 mL). Subsequently, the reaction mixture was degassed through several freeze-pump-thaw cycles until no bubbles were released. The reaction mixture was stir under inert atmosphere at 30 °C, and irradiated by a 5 W blue LED lamp (λ = 450–460 nm, the tube was placed at approximately 2 cm away from the light source) for 48 h. Then pinacol (212.8 mg, 1.8 mmol, 9.0 equiv), and Et_3N (0.25 mL, 1.8 mmol, 9.0 equiv) were added to the reaction mixture. The resulting mixture was stirred at 30 °C without further irradiation for 5 h. After that, the reaction was quenched with water, and then extracted with EtOAc (15 mL × 3). The combined organic layers were washed with water (15 mL) and then brine (15 mL), dried over anhydrous Na_2SO_4. The solvents were evaporated under reduced pressure to give the crude mixture, which was purified by flash column chromatography on silica gel (petroleum ether/ethyl acetate = 50:1 to 10:1, *silica gel was soaked with a solution of petroleum ether and triethylamine (1000/1, v/v) before use*) to give compound **12** as white solid (55.0 mg, 57% yield over 2 steps). ^{1}H NMR (400 MHz, $CDCl_3$): δ = 10.54 (s, 1H), 7.56 – 7.47 (m, 2H), 7.32 – 7.24 (m, 2H), 7.19 – 7.11 (m, 2H), 7.10 – 7.03 (m, 1H), 6.98 – 6.89 (m, 2H), 4.48 (d, J = 4.0 Hz, 1H), 3.71 (dd, J = 13.3, 4.1 Hz, 1H), 3.17 – 3.00 (m, 1H), 2.95 – 2.81 (m, 1H), 2.49 – 2.39 (m, 1H), 1.59 – 1.49 (m, 1H), 1.45 – 1.38 (m, 2H), 1.27 (s, 12H). ^{13}C NMR (100 MHz, $CDCl_3$): δ = 163.7, 148.1, 137.7, 131.8, 128.9, 127.8, 126.2 (q, J_{F-C} = 283.1 Hz), 124.4, 122.9, 121.8, 120.1, 112.0, 83.4, 67.5, 55.7 (q, J_{F-C} = 22.6 Hz), 45.1, 28.8, 28.7, 24.8, 24.7, 18.4 (br). ^{19}F NMR (376 MHz, $CDCl_3$): δ = -71.1. ^{11}B NMR (128 MHz, $CDCl_3$): δ = 33.8. IR (thin film): ν_{max} (cm^{-1}) = 3262, 2982, 2925, 1677, 1562, 1379, 1267, 1156; HRMS (ESI) calcd for $C_{26}H_{30}BF_3N_2O_3Na$ $[M+Na]^+$: 509.2198. Found: 509.2198. The relative configuration of this compound was assigned by 2D NOE analysis. For details, see pages S-

346→351.

2.12 Procedure for the synthesis of compound 13.[15]

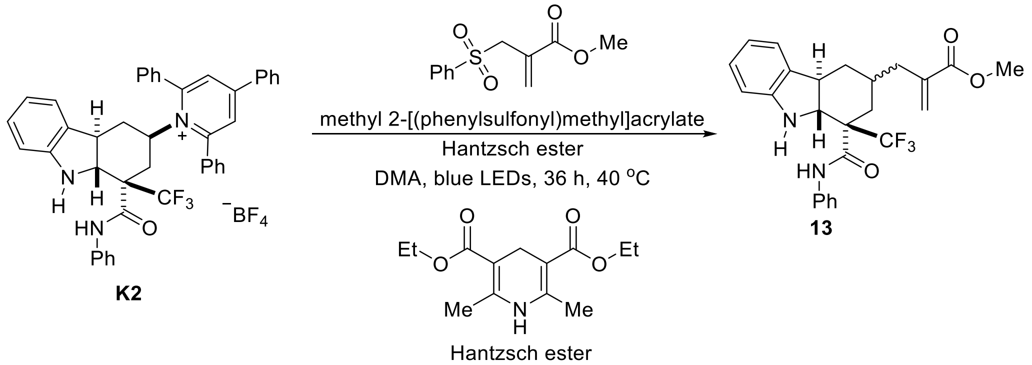

To a Young Schlenk tube (10 mL) were added **K2** (150.7 mg, 0.2 mmol, 1.0 equiv), Hantzsch ester (152.0 mg, 0.6 mmol, 3.0 equiv), methyl 2-[(phenylsulfonyl)methyl]acrylate (288.4 mg, 1.2 mmol, 6.0 equiv) and N,N-dimethylacetamide (DMA, 2.0 mL). Subsequently, the reaction mixture was degassed through several freeze-pump-thaw cycles until no bubbles were released. The reaction mixture was stir under inert atmosphere at 40 °C, and irradiated by a 5 W blue LED lamp (λ = 450–460 nm, the tube was placed at approximately 2 cm away from the light source) for 36 h. After that, the solvents were evaporated under reduced pressure to give the crude mixture, which was purified by flash column chromatography on silica gel (petroleum ether/ethyl acetate = 100:1 to 20:1, *silica gel was soaked with a solution of petroleum ether and triethylamine (1000/1, v/v) before use*) to give compound **13** as white solid (68.0 mg, 74% yield, 1.8:1 dr). ^{1}H NMR (400 MHz, $CDCl_3$): δ = 10.96 (s, 0.32H), 10.53 (s, 0.59H), 7.56 – 7.45 (m, 2H), 7.34 – 7.26 (m, 2H), 7.23 – 7.14 (m, 1H), 7.14 – 7.05 (m, 2H), 7.01 – 6.90 (m, 2H), 6.33 (d, J = 0.9 Hz, 0.63H), 6.29 (d, J = 1.4 Hz, 0.36H), 5.77 – 5.69 (m, 1H), 4.65 – 4.45 (m, 1H), 3.85 – 3.67 (m, 4H), 3.49 – 3.31 (m, 0.36H), 3.19 – 3.10 (m, 0.66H), 2.97 – 2.79 (m, 1H), 2.65 – 2.57 (m, 0.36H), 2.49 – 2.25 (m, 3H), 2.13 – 2.00 (m, 0.75H), 1.84 – 1.78 (m, 0.37H), 1.71 – 1.62 (m, 0.37H), 1.27 – 1.16 (m, 1.37H). ^{13}C NMR (100 MHz, $CDCl_3$): δ = 167.6, 167.5, 164.3, 163.5, 148.5, 148.2, 138.8, 137.8, 137.7, 137.5, 131.6, 131.4, 128.90, 128.86, 127.91, 127.86, 127.3, 126.7, 126.1 (q, $J_{F\text{-}C}$ = 283.9 Hz), 124.6, 124.5, 122.9, 122.8, 121.9, 121.8, 120.2, 120.1, 112.2, 112.1, 67.6, 67.4, 55.1 (q, $J_{F\text{-}C}$ = 23.1 Hz), 54.0 (q, $J_{F\text{-}C}$ = 22.3 Hz), 51.9, 51.8, 43.9, 38.7, 37.9, 36.2, 33.4, 33.3, 33.1, 31.7, 30.9, 30.6. ^{19}F NMR (376 MHz, $CDCl_3$): δ = -71.5, -71.7. IR (thin film): ν_{max} (cm^{-1}) = 3325, 2929, 2858, 1718, 1692, 1601, 1235, 1164, 1038; HRMS (ESI) calcd for $C_{25}H_{25}F_3N_2O_3Na$ [M+Na]$^+$: 481.1709. Found: 481.1732.

2.13 Procedure for the synthesis of compound 14.[15]

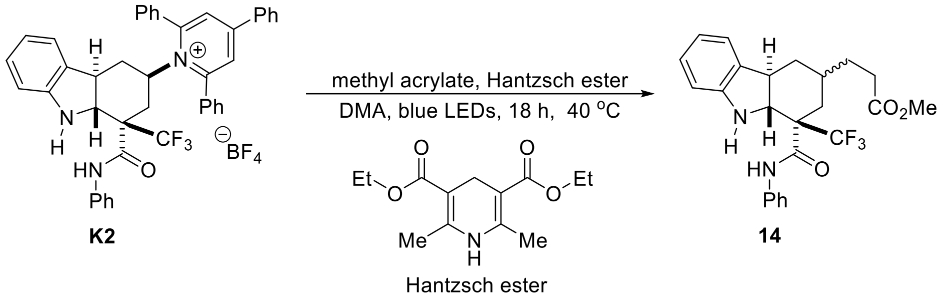

To a Young Schlenk tube (10 mL) were added **K2** (150.7 mg, 0.2 mmol, 1.0 equiv), Hantzsch ester (152.0 mg, 0.6 mmol, 3.0 equiv) and N,N-dimethylacetamide (DMA, 1.0 mL). Subsequently, the reaction mixture was degassed through several freeze-pump-thaw cycles until no bubbles were released. After that, methyl acrylate (27.0 μL, 0.3 mmol, 1.5 equiv) was added under N_2 atmosphere. The reaction mixture was stir under inert atmosphere at 40 °C, and irradiated by a 5 W blue LED lamp (λ = 450–460 nm, the tube was placed at approximately 2 cm away from the light source) for 18 h. Then the solvents were evaporated under reduced pressure to give the crude mixture, which was purified by flash column chromatography on silica gel (petroleum ether/ethyl acetate = 100:1 to 20:1, *silica gel was soaked with a solution of petroleum ether and triethylamine (1000/1, v/v) before use*) to give compound **14** as white solid (45.0 mg, 50% yield, 3:1 dr). ^{1}H NMR (400 MHz, $CDCl_3$): δ = 10.86 (s, 0.23H), 10.52 (s, 0.69H), 7.56 – 7.41 (m, 2H), 7.34 – 7.23 (m, 2H), 7.21 – 7.14 (m, 1H), 7.14 – 7.04 (m, 2H), 7.01 – 6.89 (m, 2H), 4.61 – 4.41 (m, 1H), 3.81 – 3.61 (m, 4H), 3.38 – 3.29 (m, 0.26H), 3.20 – 3.08 (m, 0.72H), 2.92 – 2.75 (m, 1H), 2.71 – 2.58 (m, 0.32H), 2.55 – 2.33 (m, 2.53H), 2.29 – 2.22 (m, 0.29H), 2.17 – 2.10 (m, 0.27H), 1.90 – 1.77 (m, 1.49H), 1.74 – 1.60 (m, 1H), 1.32 – 1.12 (m, 2H). ^{13}C NMR (100 MHz, $CDCl_3$): δ = 174.0, 173.8, 164.2, 163.5, 148.5, 148.3, 137.64, 137.55, 131.7, 131.4, 128.9, 127.92, 127.90, 126.1 (q, $J_{F\text{-}C}$ = 283.6 Hz), 125.9 (q, $J_{F\text{-}C}$ = 282.3 Hz), 124.6, 122.9, 122.7, 121.94, 121.90, 120.3, 120.1, 112.2, 112.1, 67.6, 55.1 (q, $J_{F\text{-}C}$ = 23.1 Hz), 53.9 (q, $J_{F\text{-}C}$ = 22.5 Hz), 51.6, 51.4, 43.9, 39.0, 34.1, 33.6, 33.0, 32.5, 32.4, 32.3, 31.7, 31.1, 29.4, 28.7. ^{19}F NMR (376 MHz, $CDCl_3$): δ = -71.5, -71.8. IR (thin film): ν_{max} (cm^{-1}) = 3327, 2928, 2863, 1733, 1692, 1601, 1558, 1235, 1159, 755; HRMS (ESI) calcd for $C_{24}H_{25}F_3N_2O_3Na$ $[M+Na]^+$: 469.1709. Found: 469.1740.

2.14 Procedure for the synthesis of compound 15.

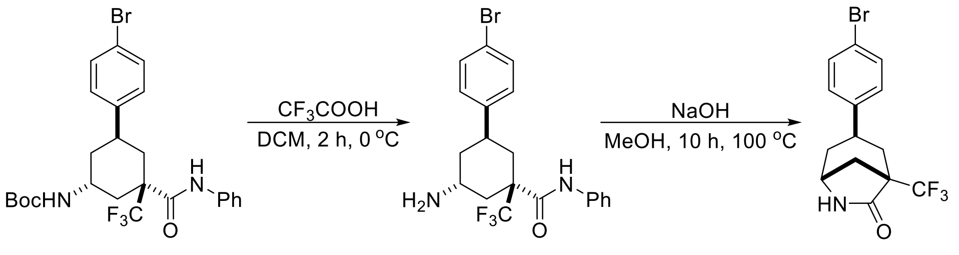

Step 1: Compound **11d** (108.3 mg, 0.2 mmol, 1.0 equiv) was dissolved in DCM (5 mL) and CF_3COOH (0.2 mL, 2.6 mmol, 13 equiv) was added at 0 °C. The reaction mixture was allowed to stir at ambient temperature for 2 h. After that, the solvents were evaporated under reduced pressure to give the crude mixture. The pH of the resulting mixture was adjusted to 8.0 through addition of sat. aq. $NaHCO_3$. The aqueous solution was extracted with EtOAc (10 mL × 3). The organic layers were combined and dried over anhydrous Na_2SO_4. The solvents were evaporated under reduced pressure to give the crude mixture, which was purified by flash column chromatography on silica gel (CH_2Cl_2/MeOH = 100:1 to 20:1, *silica gel was soaked with a solution of petroleum ether and triethylamine (1000/1, v/v) before use*) to give the crude amine product, which was used for the next step.

Step 2: To a Young Schlenk tube (10 mL) were added the crude amine product from **Step 1**, MeOH (2 mL), aq. NaOH (2 M, 0.8 mL) under N_2 atmosphere. The reaction mixture was stir at 100 °C for 10 h. After that, MeOH was evaporated under reduced pressure to give the crude mixture, which was diluted with H_2O (10 mL). The aqueous layer was extracted with EtOAc (15 mL × 3). The combined organic layers were dried over anhydrous Na_2SO_4, and concentrated in vacuo to give the crude mixture, which was purified by flash chromatography on silica gel (petroleum ether/ethyl acetate = 20:1 to 3:1) to afford the title compound **15** as white solid (48.0 mg, 69% yield over 2 steps). ^1H NMR (400 MHz, $CDCl_3$): δ = 7.54 (s, 1H), 7.47 – 7.40 (m, 2H), 7.15 – 7.06 (m, 2H), 4.08 – 3.92 (m, 1H), 3.20 – 3.01 (m, 1H), 2.70 – 2.53 (m, 1H), 2.39 – 2.22 (m, 1H), 2.14 – 2.00 (m, 1H), 1.87 (d, J = 10.9 Hz, 1H), 1.75 (t, J = 12.7 Hz, 1H), 1.65 (t, J = 12.5 Hz, 1H). ^{13}C NMR (100 MHz, $CDCl_3$): δ = 172.9, 141.7, 131.8, 129.0, 125.2 (q, $J_{F\text{-}C}$ = 276.6 Hz), 120.7, 51.5 (q, $J_{F\text{-}C}$ = 28.2 Hz), 49.3, 38.7, 36.3, 35.1, 32.8. ^{19}F NMR (376 MHz, $CDCl_3$): δ = -74.2. IR (thin film): ν_{max} (cm^{-1}) = 3287, 2956, 2924, 2853, 1712, 1490, 1154; HRMS (ESI) calcd for $C_{14}H_{13}BrF_3NONa$ $[M+Na]^+$: 370.0025. Found: 370.0022.

3. Mechanistic studies.

3.1 Reaction of 1a and 2'.

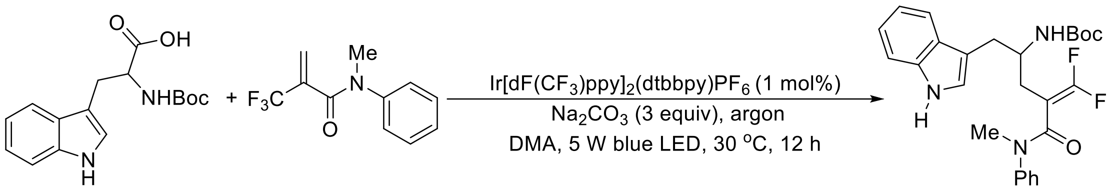

1a **2'** **3'**

To a Young Schlenk tube (10 mL) were added Ir[dF(CF_3)ppy]$_2$(dtbbpy)PF_6 (1.1 mg, 0.001 mmol, 1 mol%), **1a** (45.7 mg, 0.15 mmol, 1.5 equiv), **2'** (22.9 mg, 0.1 mmol, 1.0 equiv), Na_2CO_3 (31.8 mg, 0.3 mmol, 3.0 equiv), and N,N-dimethylacetamide (DMA, 2.0 mL). Subsequently, the reaction mixture was degassed through several freeze-pump-thaw cycles until no bubbles were released. The reaction mixture was stirred under argon at 30 °C, and irradiated by a 5 W blue LED lamp (λ = 450–460 nm, the tube was placed at approximately 2 cm away from the light source). After 12 h, the reaction mixture was passed through a short pad of celite and washed with ethyl acetate. The solvents were evaporated under reduced pressure to give the crude mixture, which was purified by flash column chromatography on silica gel (petroleum ether/ethyl acetate = 40:1 to 15:1, *silica gel was soaked with a solution of petroleum ether and triethylamine (1000/1, v/v) before use*) to afford the title compound **3'** as a white solid (20.3 mg, 43% yield). ^{1}H NMR (400 MHz, C_3D_6O): δ = 10.07 (s, 1H), 7.73 (d, J = 7.8 Hz, 1H), 7.41 (d, J = 8.0 Hz, 1H), 7.36 – 7.23 (m, 3H), 7.17 – 7.03 (m, 5H), 5.94 (d, J = 8.0 Hz, 1H), 4.14 – 4.02 (m, 1H), 3.26 (s, 3H), 3.11 – 2.99 (m, 1H), 2.99 – 2.93 (m, 1H), 2.39 – 2.09 (m, 2H), 1.41 (s, 9H). ^{13}C NMR (100 MHz, C_3D_6O): δ = 165.1 – 164.8 (m, 1C), 156.1, 154.7 (t, $J_{F\text{-}C}$ = 289.4 Hz), 143.9, 137.4, 129.7, 128.7, 127.8, 127.0, 123.9, 121.9, 119.5, 119.2, 112.2, 111.9, 89.5 (t, $J_{F\text{-}C}$ = 18.5 Hz), 78.5, 50.9, 37.6, 31.0, 30.5, 28.5. ^{19}F NMR (376 MHz, $CDCl_3$): δ = -77.7, -88.0 (m). IR (thin film): ν_{max} (cm^{-1}) = 3334, 3057, 2976, 1735, 1692, 1595, 1496, 1391, 1168, 742, 700; HRMS (ESI) calcd for $C_{26}H_{30}F_2N_3O_3$ [M+H]$^+$: 470.2250. Found: 470.2249.

3.2 Deuteration experiment.

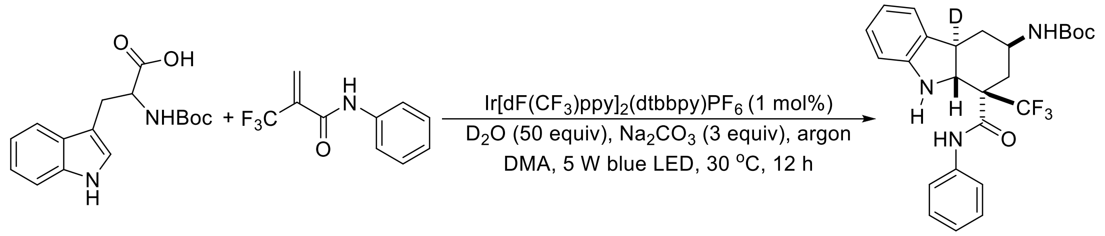

To a Young Schlenk tube (10 mL) were added Ir[dF(CF_3)ppy]$_2$(dtbbpy)PF_6 (1.1 mg, 0.001 mmol, 1 mol %), **1a** (45.7 mg, 0.15 mmol, 1.5 equiv), **2a** (21.5 mg, 0.1 mmol, 1.0 equiv), Na_2CO_3 (31.8 mg, 0.3 mmol, 3.0 equiv), D_2O (90.5 μL, 5 mmol, 50.0 equiv), and N,N-dimethylacetamide (DMA, 2.0 mL). Subsequently, the reaction mixture was degassed through several freeze-pump-thaw cycles until no bubbles were released. The reaction mixture was stirred under argon at 30 °C, and irradiated by a 5 W blue LED lamp (λ = 450−460 nm, the tube was placed at approximately 2 cm away from the light source). After 12 h, the reaction mixture was passed through a short pad of celite and washed with ethyl acetate. The solvents were evaporated under reduced pressure to give the crude mixture, which was purified by flash column chromatography on silica gel (petroleum ether/ethyl acetate = 40:1 to 15:1, *silica gel was soaked with a solution of petroleum ether and triethylamine (1000/1, v/v) before use*) to afford the title compound **D-3a** as a white solid (31.9 mg, 67% yield). ^{1}H NMR (400 MHz, C_3D_6O): δ = 10.87 (s, 1H), 7.53 (d, J = 7.9 Hz, 2H), 7.29 (t, J = 7.9 Hz, 2H), 7.22 – 7.11 (m, 2H), 7.08 (t, J = 7.4 Hz, 1H), 7.02 (d, J = 7.7 Hz, 1H), 6.89 (t, J = 7.4 Hz, 1H), 6.14 (d, J = 7.0 Hz, 1H), 6.02 (d, J = 4.1 Hz, 1H), 4.04 – 3.72 (m, 2H), 3.18 (t, J = 11.7 Hz, 0.07H), 3.03 – 2.88 (m, 1H), 2.63 (dd, J = 11.9, 4.0 Hz, 1H), 1.70 (t, J = 12.3 Hz, 1H), 1.60 – 1.50 (m, 1H), 1.43 (s, 9H).

3.3 Cyclic voltammetry measurement.

Electrochemical potentials were obtained with a standard set of conditions to main internal consistency. Cyclic voltammograms were collected with a potentiostat. Samples were prepared with 0.01 mmol of **1a**, **K1, S13,** and **2a** respectively in anhydrous acetonitrile solution of tetrabutylammonium hexafluorophosphate (0.1 M, 10 mL). Measurements employed a radium glassy carbon working electrode, platinum wire counter electrode, and a SCE reference electrode.

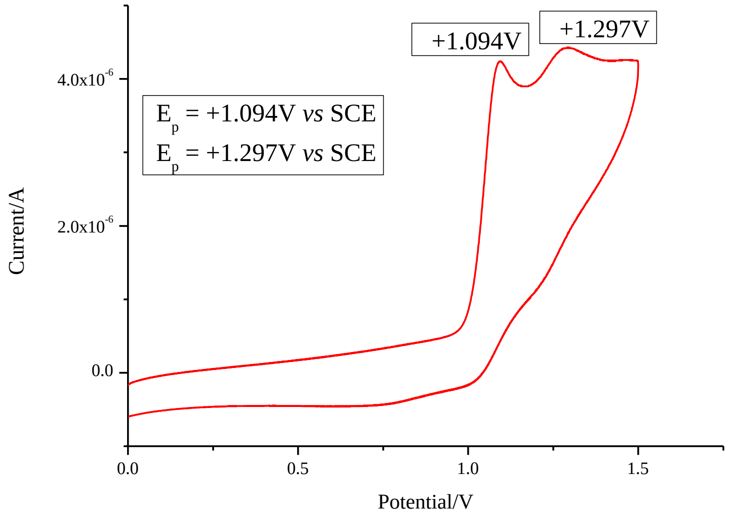

Supplementary Figure 3. Cyclic voltammogram of **1a** in MeCN.

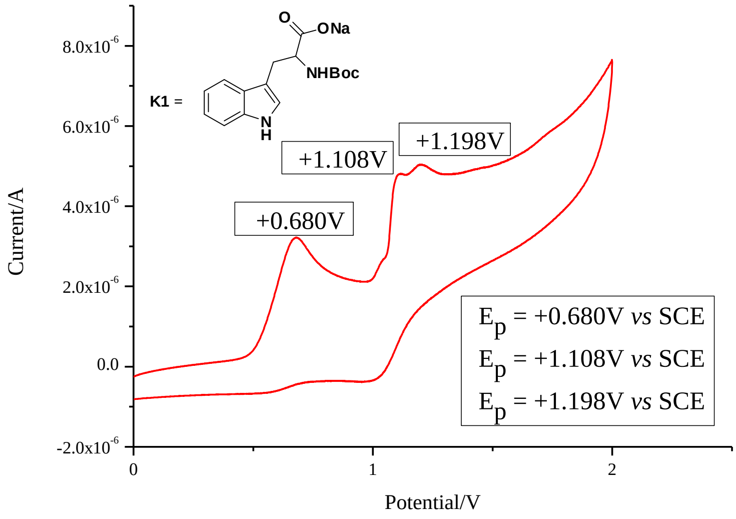

Supplementary Figure 4. Cyclic voltammogram of **K1** in MeCN.

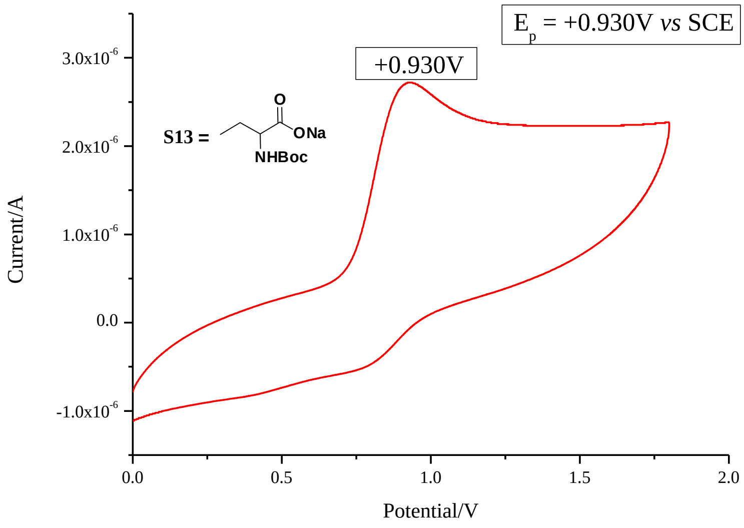

Supplementary Figure 5. Cyclic voltammogram of **S13** in MeCN.

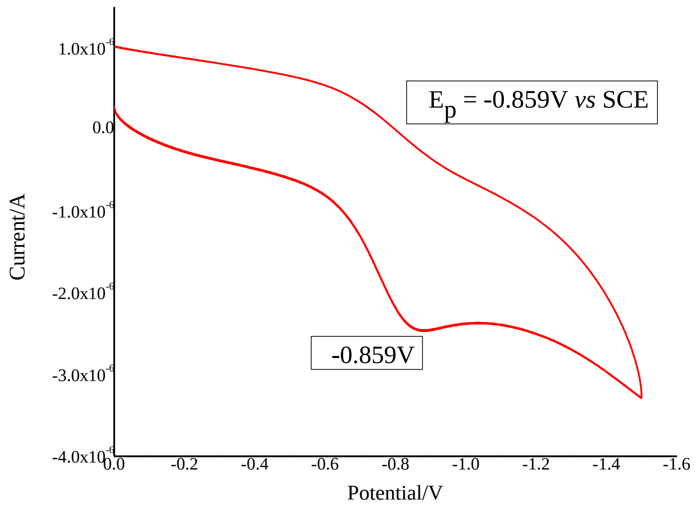

Supplementary Figure 6. Cyclic voltammogram of **2a** in MeCN.

Cyclic voltammetry measurement experiments were carried out. As shown above, compound **1a** [E_P = +0.68 V *vs* SCE in CH_3CN for the COO^- moiety; **S13** (for comparation): E_P = +0.93 V *vs*. SCE in CH_3CN for the COO^- moiety] could serve as the precursor of α-amino radical given the prominent ability to generate the radical species via single electron oxidative decarboxylation by the visible-light-activated Ir[dF(CF_3)ppy]$_2$(dtbbpy)PF_6 (Ir^{III}*/Ir^{II} = +1.21 V *vs* SCE).

3.4 Stern-Volmer quenching experiments.

Emission intensities were recorded on a spectrofluorometer. Ir[dF(CF_3)ppy]$_2$(dtbbpy)PF_6 solution was excited at 380 nm and the emission intensity at 481 nm was observed. A solution of Ir[dF(CF_3)ppy]$_2$(dtbbpy)PF_6 (5.0×10^{-5} M) in N,N-dimethylacetamide was added to the appropriate amount of quencher in volumetric flask (5.0 mL) under N_2. The solution was transferred to a 1.5 mL quartz cell and the emission spectra of the samples were collected.

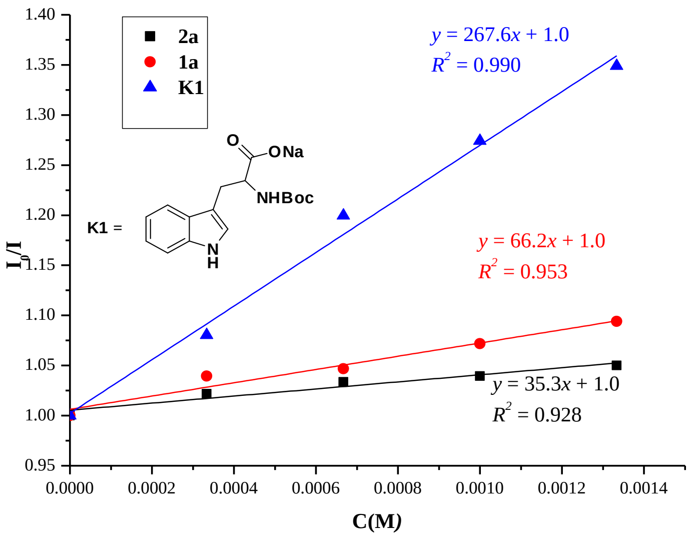

Supplementary Figure 7. Stern–Volmer quenching experiment of *Ir[dF(CF_3)ppy]$_2$(dtbbpy)PF_6 with **1a, 2a,** and **K1**.

4. Crystallographic information.

4.1 Structure of compound 3k in the solid state.

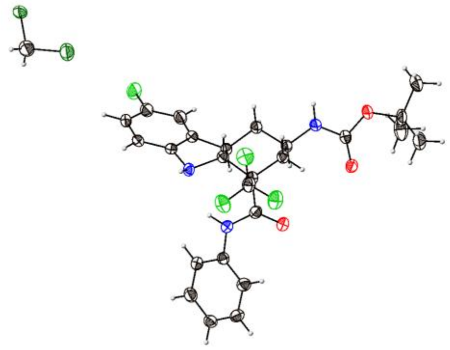

Supplementary Figure 8. X-ray crystal structure of **3k**.

Supplementary Table 1. Crystal data and structure refinement for exp_12771.

Identification code	exp_12771
Empirical formula	$C_{51}H_{56}Cl_2F_8N_6O_6$
Formula weight	1071.91
Temperature/K	100.01(10)
Crystal system	orthorhombic
Space group	$P2_12_12_1$
a/Å	16.9972(4)
b/Å	17.0684(3)
c/Å	18.5662(3)
α /°	90
β /°	90
γ /°	90
Volume/Å^3	5386.30(18)
Z	4
ρ_{calc}g/cm^3	1.322
μ /mm^{-1}	0.201
F(000)	2232.0
Crystal size/mm^3	0.5 × 0.4 × 0.2
Radiation	Mo Kα (λ = 0.71073)
2Θ range for data collection/°	6.766 to 61.47
Index ranges	-22 ≤ h ≤ 12, -23 ≤ k ≤ 24, -26 ≤ l ≤ 17
Reflections collected	21208
Independent reflections	13403 [R_{int} = 0.0239, R_{sigma} = 0.0462]
Data/restraints/parameters	13403/146/723
Goodness-of-fit on F^2	1.019
Final R indexes [I>=2σ (I)]	R_1 = 0.0413, wR_2 = 0.0882
Final R indexes [all data]	R_1 = 0.0527, wR_2 = 0.0961

Largest diff. peak/hole / e Å^{-3}	0.27/-0.46
Flack parameter	-0.02(2)

4.2 Structure of compound 3ab in the solid state.

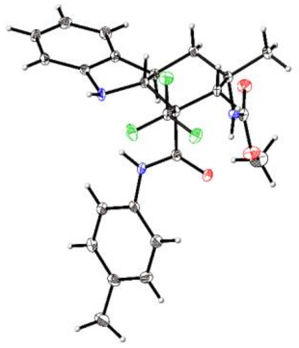

Supplementary Figure 9. X-ray crystal structure of **3ab**.

Supplementary Table 2. Crystal data and structure refinement for exp_11878.

Identification code	exp_11878
Empirical formula	$C_{24}H_{26}F_3N_3O_3$
Formula weight	461.48
Temperature/K	100.01(10)
Crystal system	monoclinic
Space group	$P2_1/n$
a/Å	11.95987(18)
b/Å	11.27458(15)
c/Å	16.7226(2)
α /°	90
β /°	95.6234(13)
γ /°	90
Volume/Å^3	2244.06(6)
Z	4
ρ_{calc}g/cm^3	1.366
μ /mm^{-1}	0.909
F(000)	968.0
Crystal size/mm^3	0.4 × 0.17 × 0.1
Radiation	CuKα (λ = 1.54184)
2Θ range for data collection/°	8.7 to 146.636
Index ranges	-11 ≤ h ≤ 14, -13 ≤ k ≤ 12, -16 ≤ l ≤ 20
Reflections collected	8493
Independent reflections	4377 [R_{int} = 0.0349, R_{sigma} = 0.0361]
Data/restraints/parameters	4377/0/301
Goodness-of-fit on F^2	1.034
Final R indexes [I>=2σ (I)]	R_1 = 0.0504, wR_2 = 0.1335

Final R indexes [all data]	$R_1 = 0.0550$, $wR_2 = 0.1400$
Largest diff. peak/hole / e Å^{-3}	0.41/-0.49

4.3 Structure of compound 3k' in the solid state.

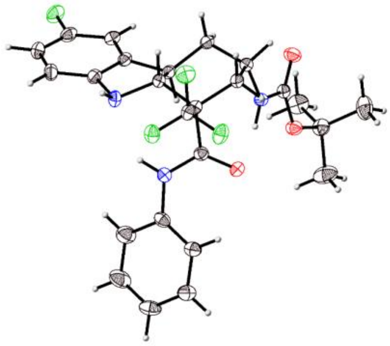

Supplementary Figure 10. X-ray crystal structure of **3k'**.

Supplementary Table 3. Crystal data and structure refinement for exp_652.

Identification code	exp_652
Empirical formula	$C_{25}H_{27}F_4N_3O_3$
Formula weight	493.49
Temperature/K	100.00(10)
Crystal system	monoclinic
Space group	$P2_1/c$
a/Å	15.73185(8)
b/Å	16.10722(8)
c/Å	19.75902(11)
α /°	90
β /°	99.3509(5)
γ /°	90
Volume/Å^3	4940.33(4)
Z	8
ρ_{calc}g/cm^3	1.327
μ /mm^{-1}	0.921
F(000)	2064.0
Crystal size/mm^3	0.3 × 0.2 × 0.2
Radiation	CuKα (λ = 1.54184)
2Θ range for data collection/°	5.694 to 148.802
Index ranges	$-19 \leq h \leq 19$, $-20 \leq k \leq 20$, $-24 \leq l \leq 23$
Reflections collected	129543
Independent reflections	9986 [$R_{int} = 0.0992$, $R_{sigma} = 0.0299$]
Data/restraints/parameters	9986/227/695
Goodness-of-fit on F^2	1.061
Final R indexes [I>=2σ (I)]	$R_1 = 0.0388$, $wR_2 = 0.1036$

Final R indexes [all data]	R_1 = 0.0409, wR_2 = 0.1051
Largest diff. peak/hole / e Å^{-3}	0.40/-0.28

4.4 Structure of compound 3ab' in the solid state.

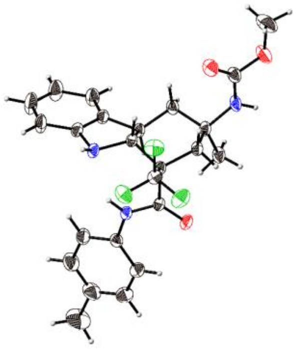

Supplementary Figure 11. X-ray crystal structure of **3ab'**.

Supplementary Table 4. Crystal data and structure refinement for exp_11912.

Identification code	exp_11912
Empirical formula	$C_{24}H_{26}F_3N_3O_3$
Formula weight	461.48
Temperature/K	293(2)
Crystal system	monoclinic
Space group	$P2_1/c$
a/Å	12.0153(3)
b/Å	8.33879(19)
c/Å	23.3346(4)
α /°	90
β /°	91.1759(19)
γ /°	90
Volume/Å^3	2337.48(9)
Z	4
ρ_{calc}g/cm^3	1.311
μ /mm^{-1}	0.873
F(000)	968.0
Crystal size/mm^3	0.5 × 0.3 × 0.2
Radiation	CuKα (λ = 1.54184)
2Θ range for data collection/°	7.358 to 137.122
Index ranges	-14 ≤ h ≤ 14, -9 ≤ k ≤ 9, -27 ≤ l ≤ 19
Reflections collected	8610
Independent reflections	4196 [R_{int} = 0.0616, R_{sigma} = 0.0555]
Data/restraints/parameters	4196/0/302
Goodness-of-fit on F^2	1.038

Final R indexes [I>=2σ (I)]	R_1 = 0.0672, wR_2 = 0.1718
Final R indexes [all data]	R_1 = 0.0776, wR_2 = 0.1902
Largest diff. peak/hole / e Å^{-3}	0.34/-0.35

4.5 Structure of compound 8a in the solid state.

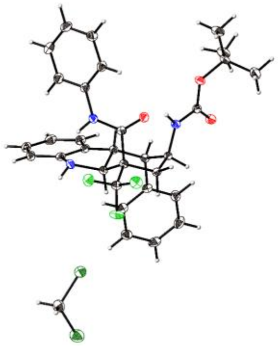

Supplementary Figure 12. X-ray crystal structure of **8a**.

Supplementary Table 5. Crystal data and structure refinement for exp_5177.

Identification code	exp_5177
Empirical formula	$C_{32}H_{34}Cl_2F_3N_3O_3$
Formula weight	636.52
Temperature/K	99.9(3)
Crystal system	orthorhombic
Space group	$P2_12_12_1$
a/Å	10.32267(6)
b/Å	16.55479(8)
c/Å	17.70825(11)
α/°	90
β/°	90
γ/°	90
Volume/Å^3	3026.16(3)
Z	4
ρ_{calc}g/cm^3	1.397
μ/mm^{-1}	2.419
F(000)	1328.0
Crystal size/mm^3	0.5 × 0.14 × 0.11
Radiation	CuKα (λ = 1.54184)
2Θ range for data collection/°	7.31 to 143.06
Index ranges	$-12 \leq h \leq 12$, $-20 \leq k \leq 12$, $-21 \leq l \leq 21$
Reflections collected	34356

Independent reflections	5754 [R_{int} = 0.0465, R_{sigma} = 0.0271]
Data/restraints/parameters	5754/0/392
Goodness-of-fit on F^2	1.031
Final R indexes [I>=2σ (I)]	R_1 = 0.0243, wR_2 = 0.0631
Final R indexes [all data]	R_1 = 0.0246, wR_2 = 0.0634
Largest diff. peak/hole / e Å^{-3}	0.28/-0.23
Flack parameter	-0.001(4)

By using the CuKα radiation source and the high sensitive Rigaku Synergy Hypix 6000 detector, the intensity differences between inversion-related Bragg reflections h k l and -h -k -l (Friedel pairs) could be measured accurately even for crystals containing only light atoms. As a result, the absolute structure could be determined as indicated by the Flack parameter (close to zero) with low standard deviation.

4.6 Structure of compound 9a in the solid state.

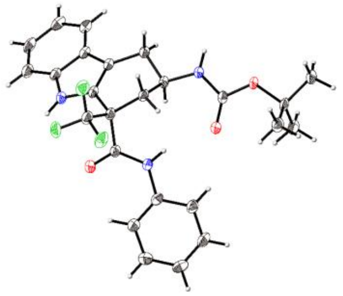

Supplementary Figure 13. X-ray crystal structure of **9a**.

Supplementary Table 6. Crystal data and structure refinement for exp_985.

Identification code	exp_985
Empirical formula	$C_{25}H_{26}F_3N_3O_3$
Formula weight	473.49
Temperature/K	100.00(10)
Crystal system	triclinic
Space group	P-1
a/Å	10.45461(16)
b/Å	10.96921(16)
c/Å	11.03150(14)
α/°	82.4248(11)
β/°	68.9586(13)
γ/°	74.7782(13)

Volume/Å^3	1138.31(3)
Z	2
ρ_{calc}g/cm^3	1.381
μ/mm^{-1}	0.912
F(000)	496.0
Crystal size/mm^3	0.3 × 0.2 × 0.18
Radiation	CuKα (λ = 1.54184)
2Θ range for data collection/°	8.362 to 143.854
Index ranges	-12 ≤ h ≤ 11, -13 ≤ k ≤ 13, -13 ≤ l ≤ 13
Reflections collected	23789
Independent reflections	4226 [R_{int} = 0.0382, R_{sigma} = 0.0222]
Data/restraints/parameters	4226/0/311
Goodness-of-fit on F^2	1.060
Final R indexes [I>=2σ (I)]	R_1 = 0.0346, wR_2 = 0.0895
Final R indexes [all data]	R_1 = 0.0364, wR_2 = 0.0911
Largest diff. peak/hole / e Å^{-3}	0.28/-0.21

4.7 Structure of compound S16 in the solid state.

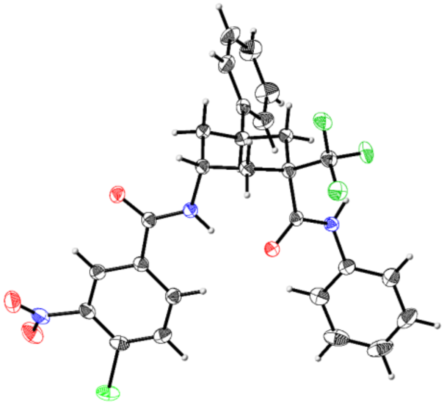

Supplementary Figure 14. X-ray crystal structure of **S16**.

Supplementary Table 7. Crystal data and structure refinement for exp_4367.

Identification code	exp_4367
Empirical formula	$C_{27}H_{23}ClF_3N_3O_4$
Formula weight	545.93
Temperature/K	100.0(3)
Crystal system	monoclinic
Space group	P2$_1$/n
a/Å	9.84155(15)
b/Å	14.1048(2)
c/Å	17.9133(3)

α/°	90
β/°	97.4498(15)
γ/°	90
Volume/Å^3	2465.62(7)
Z	4
ρ_{calc}g/cm^3	1.471
μ/mm^{-1}	1.932
F(000)	1128.0
Crystal size/mm^3	0.15 × 0.15 × 0.15
Radiation	CuKα (λ = 1.54184)
2Θ range for data collection/°	8.004 to 143.402
Index ranges	$-11 \le h \le 6$, $-15 \le k \le 17$, $-22 \le l \le 22$
Reflections collected	15634
Independent reflections	4547 [R_{int} = 0.0644, R_{sigma} = 0.0558]
Data/restraints/parameters	4547/0/344
Goodness-of-fit on F^2	1.050
Final R indexes [I>=2σ (I)]	R_1 = 0.0354, wR_2 = 0.0835
Final R indexes [all data]	R_1 = 0.0609, wR_2 = 0.0940
Largest diff. peak/hole / e Å^{-3}	0.45/-0.59

Preparation of compound **S16.**

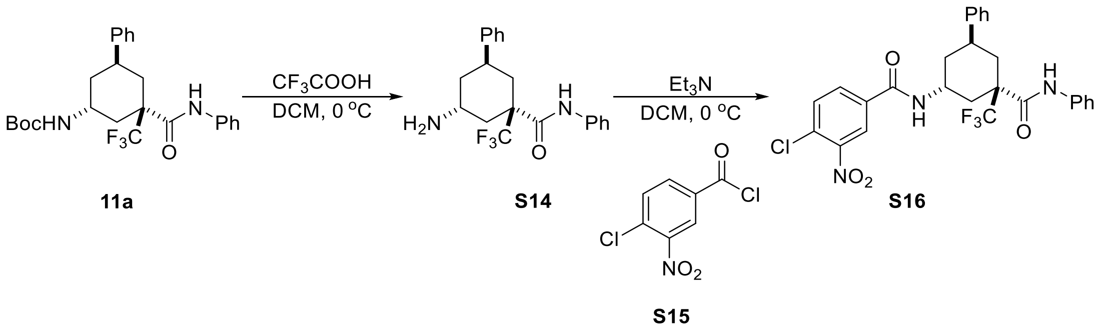

Step 1: Compound **11a** (46.2 mg, 0.1 mmol, 1.0 equiv) was dissolved in DCM (5 mL). Then CF_3COOH (0.1 mL, 1.3 mmol, 13 equiv) was added at 0 °C and the resulting mixture was allowed to stir at ambient temperature for 2 h. After that, the solvents were evaporated under reduced pressure to give the crude mixture. The pH of the resulting mixture was adjusted to 8.0 through addition of sat. aq. $NaHCO_3$. The aqueous solution was extracted with EtOAc (10 mL × 3). The combined organic layers were dried over Na_2SO_4 and concentrated in vacuo to give **S14**, which was used without further purification.

Step 2: Compound **S14** was dissolved in DCM (5 mL). Then reagent **S15** (32.8 mg, 0.15 mmol, 1.5 equiv) and Et_3N (20.9 μL, 0.15 mmol, 1.5 equiv) were added at 0 °C. The reaction mixture was allowed to stir at ambient temperature for 12 h. After that, the solvents were evaporated under reduced pressure to give the crude mixture, which was purified by flash column chromatography on silica gel (petroleum ether/ethyl acetate = 50:1 to 10:1, *silica gel was soaked with a solution of petroleum ether and triethylamine (1000/1, v/v) before use*) to afford the title compound **S16** as a white solid (38.2 mg, 70% yield over 2 steps). ^{1}H NMR (400 MHz, C_2D_6OS): δ = 9.64 (s, 1H), 8.38 (d, *J* = 4.7 Hz, 1H), 8.21 (d, *J* = 1.7 Hz, 1H), 7.89 (dd, *J* = 8.4, 1.8 Hz, 1H), 7.63 (d, *J* = 8.4 Hz, 1H), 7.47 (d, *J* = 7.8 Hz, 2H), 7.44 – 7.31 (m, 4H), 7.24 (t, *J* = 6.9 Hz, 1H), 7.11 (t, *J* = 7.8 Hz, 2H), 6.98 (t, *J* = 7.3 Hz, 1H), 4.47 – 4.29 (m, 1H), 3.40 (t, *J* = 11.5 Hz, 1H), 3.16 (d, *J* = 14.4 Hz, 1H), 2.71 (d, *J* = 12.8 Hz, 1H), 2.06 – 1.84 (m, 3H), 1.75 (t, *J* = 13.0 Hz, 1H). ^{13}C NMR (100 MHz, C_2D_6OS): δ = 165.4, 163.7, 146.8, 145.2, 138.0, 134.5, 132.5, 131.2, 128.3, 128.0, 127.3, 127.0, 126.5 (q, J_{F-C} = 282.4 Hz), 126.3, 124.3, 123.9, 121.0, 52.0 (q, J_{F-C} = 23.1 Hz), 45.0, 34.9, 33.1, 32.9, 28.2. ^{19}F NMR (376 MHz, $CDCl_3$): δ = -73.5. IR (thin film): ν_{max} (cm^{-1}) = 3316, 2923, 2852, 1652, 1533, 1443, 1230, 1166; HRMS (ESI) calcd for $C_{27}H_{24}ClF_3N_3O_4$ $[M+H]^+$: 546.1402. Found: 546.1397.

4.8 Structure of compound 11d' in the solid state.

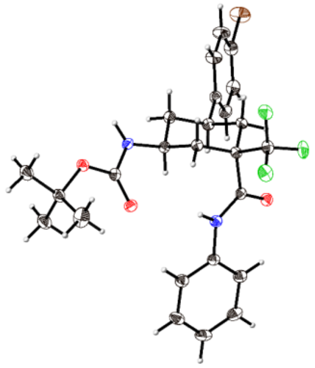

Supplementary Figure 15. X-ray crystal structure of **11d'**.

Supplementary Table 8. Crystal data and structure refinement for exp_4995.

Identification code	exp_4995
Empirical formula	$C_{25}H_{28}BrF_3N_2O_3$
Formula weight	541.40
Temperature/K	99.9(6)
Crystal system	monoclinic
Space group	$P2_1/c$
a/Å	13.7347(2)

b/Å	14.63550(10)
c/Å	13.3829(2)
α/°	90
β/°	111.752(2)
γ/°	90
Volume/Å^3	2498.60(6)
Z	4
ρ_{calc}g/cm^3	1.439
μ/mm^{-1}	2.692
F(000)	1112.0
Crystal size/mm^3	0.5 × 0.15 × 0.15
Radiation	CuKα (λ = 1.54184)
2Θ range for data collection/°	6.93 to 148.266
Index ranges	-16 ≤ h ≤ 17, -18 ≤ k ≤ 6, -16 ≤ l ≤ 15
Reflections collected	15869
Independent reflections	4914 [R_{int} = 0.0366, R_{sigma} = 0.0328]
Data/restraints/parameters	4914/0/310
Goodness-of-fit on F^2	1.044
Final R indexes [I>=2σ (I)]	R_1 = 0.0355, wR_2 = 0.0954
Final R indexes [all data]	R_1 = 0.0368, wR_2 = 0.0968
Largest diff. peak/hole / e Å^{-3}	0.60/-0.88

5. Signal assignment of compounds 3j, 8e, 9d, and 11f.

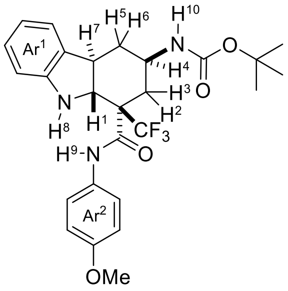

3j

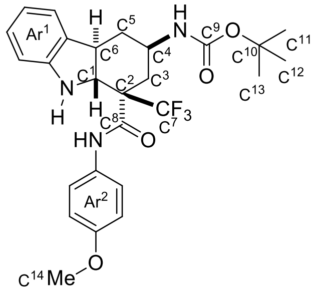

3j

Supplementary Table 9. ^{1}H NMR signal assignment of compound **3j.**

Position	^{1}H NMR, δ (ppm)/*J* (Hz)
t-Bu (9H)	1.43, s
H^{5} (1H)	1.60 – 1.49, m
H^{2} (1H)	1.76 – 1.62, m
H^{6} (1H)	2.68 – 2.57, m
H^{3} (1H)	2.96 – 2.87, m
H^{7} (1H)	3.22 – 3.11, m
OMe (3H)	3.73, s
H^{1} (1H)	3.81, dd, 13.4, 4.4
H^{4} (1H)	3.95 – 3.85, m
H^{8} (1H)	5.97, d, 4.1
H^{10} (1H)	6.13, d, 6.8
$Ar^{2}H$ (2H)	6.87 – 6.82, m
$Ar^{1}H$ (1H)	6.94 – 6.87, m
$Ar^{1}H$ (1H)	7.03 – 6.96, m
$Ar^{1}H$ (2H)	7.22 – 7.08, m
$Ar^{2}H$ (2H)	7.51 – 7.39, m
H^{9} (1H)	10.67, s

Supplementary Table 10. ^{13}C NMR signal assignment of compound **3j.**

Position	^{13}C NMR, δ (ppm)/*J* (Hz)
C^{11-13}	28.5
C^{3}	33.8
C^{5}	34.4
C^{6}	40.0
C^{4}	47.6
C^{2}	55.3, q, 23.0
C^{14}	55.5
C^{1}	67.5
C^{10}	78.7
C^{7}	126.7, q, 281.7
C^{9}	155.5
C^{8}	163.4
$Ar^{1}C$	113.2, 121.9, 123.2, 128.5, 131.8, 150.5
$Ar^{2}C$	114.7, 121.8, 132.0, 157.3

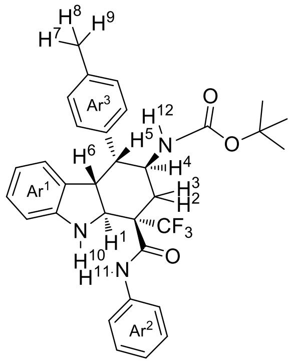

8e

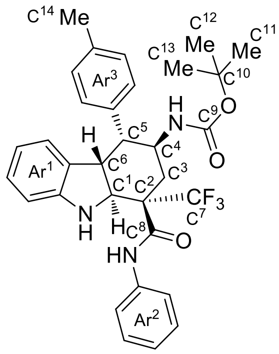

8e

Supplementary Table 11. ^{1}H NMR signal assignment of compound **8e.**

Position	^{1}H NMR δ (ppm)/*J* (Hz)
t-Bu (9H)	1.48, s
H^{2} (1H)	2.05, dd, 15.1, 5.7
$H^{7\text{-}9}$ (3H)	2.26, s
H^{3} (1H)	2.88, d, 15.0
H^{6} (1H)	3.88, dd, 13.9, 4.2
H^{5} (1H)	4.05 – 3.96, m
H^{4} (1H)	4.39 – 4.27, m
H^{10} (1H)	4.54, d, 4.8
H^{1} (1H)	4.61, dd, 14.0, 4.8
H^{12} (1H)	6.27, d, 3.2
ArH (1H)	6.88, t, 7.4
ArH (1H)	6.94, d, 7.8
Ar^{3}H (2H)	7.02, d, 7.9
ArH (2H)	7.08, d, 7.4
ArH (2H)	7.19 – 7.11, m
Ar^{1}H (1H), Ar^{2}H (1H), Ar^{3}H (2H)	7.39 – 7.28, m
Ar^{2}H (2H)	7.53, d, 7.8
H^{11} (1H)	11.17, s

Supplementary Table 12. ^{13}C NMR signal assignment of compound **8e.**

Position	^{13}C NMR, δ (ppm)/*J* (Hz)
C^{14}	20.8
C^{3}	27.7
$C^{11\text{-}13}$	28.5
C^{6}	42.7
C^{5}	44.2
C^{4}	52.9
C^{2}	55.3, q, 22.8
C^{1}	59.9
C^{10}	79.4
C^{7}	125.8, q, 283.3
C^{9}	155.8
C^{8}	165.4
ArC	112.2, 122.3, 124.3, 125.0, 127.8, 128.9, 129.9, 135.9, 137.3,
Ar^{1}C	129.4, 135.1
Ar^{2}C	120.5
Ar^{3}C	129.0, 147.3

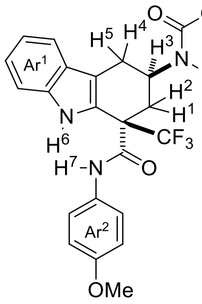

9d

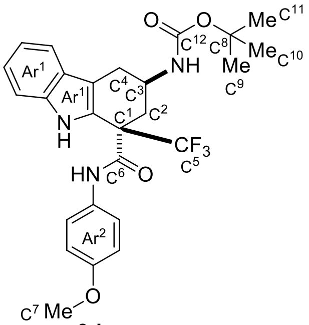

9d

Supplementary Table 13. ^{1}H NMR signal assignment of compound **9d.**

Position	^{1}H NMR δ (ppm)/*J* (Hz)
t-Bu (9H)	1.49, s
H^{1} (1H)	2.25, t, 12.8
H^{4} (1H)	2.79, dd, 14.8, 11.3
H^{2} (1H)	2.97, d, 13.3
H^{5} (1H)	3.34 – 3.09, m
OMe (3H)	3.74, s
H^{3} (1H)	4.22 – 4.03, m
H^{8} (1H)	6.69, d, 5.9
Ar^{2}H (2H)	6.96 – 6.79, m
Ar^{1}H (1H)	7.08, t, 7.4
Ar^{1}H (1H)	7.19, t, 7.6
Ar^{1}H (2H), Ar^{2}H (2H)	7.65 – 7.39, m
H^{6} (1H)	9.12, s
H^{7} (1H)	10.27, s

Supplementary Table 14. ^{13}C NMR signal assignment of compound **9d.**

Position	^{13}C NMR, δ (ppm)/*J* (Hz)
C^{4}	27.9
C$^{9-11}$	28.5
C^{2}	34.8
C^{3}	46.4
C^{1}	55.4, q, 23.7
C^{7}	55.5
C^{8}	79.6
C^{5}	126.4, q, 282.2
C^{12}	156.9
C^{6}	165.0
ArC	112.6, 114.3, 119.2, 123.3
Ar^{1}C	120.0, 123.5, 125.6, 127.0, 138.6
Ar^{2}C	131.4, 157.6

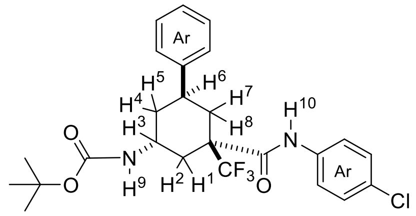

11f

Supplementary Table 15. ¹H NMR signal assignment of compound **11f.**

Position	¹H NMR δ (ppm)/*J* (Hz)
t-Bu (9H)	1.20, s
H^7 (1H)	1.71, t, 12.9
H^4 (1H)	1.88 – 1.78, m
H^1 (1H)	2.00 – 1.93, m
H^5 (1H)	2.06 – 2.00, m
H^8 (1H)	2.60, d, 12.8
H^2 (1H)	2.80, d, 14.8
H^6 (1H)	3.26 – 3.12, m
H^3 (1H)	4.19 – 4.05, m
H^9 (1H)	5.03, d, 2.0
ArH (1H)	7.26 – 7.21, m
ArH (2H)	7.30 – 7.25, m
ArH (4H)	7.37 – 7.31, m
ArH (2H)	7.55 – 7.46, m
H^{10} (1H)	7.68, s

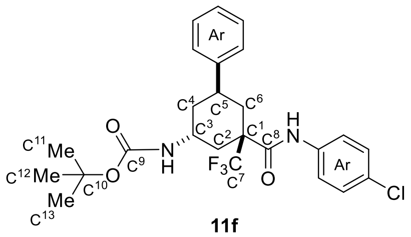

11f

Supplementary Table 16. ¹³C NMR signal assignment of compound **11f.**

Position	¹³C NMR, δ (ppm)/*J* (Hz)
C^{11-13}	28.1
C^2	30.5
C^5	33.9
C^6	34.4
C^4	36.4
C^3	45.5
C^1	52.2, q, 23.6
C^{10}	79.8
C^7	126.3, q, 281.7
C^9	155.7
C^8	166.4
ArC	122.2, 126.8, 126.9, 128.7, 129.0, 130.3, 135.6, 144.1

6. Copies of HPLC spectra.

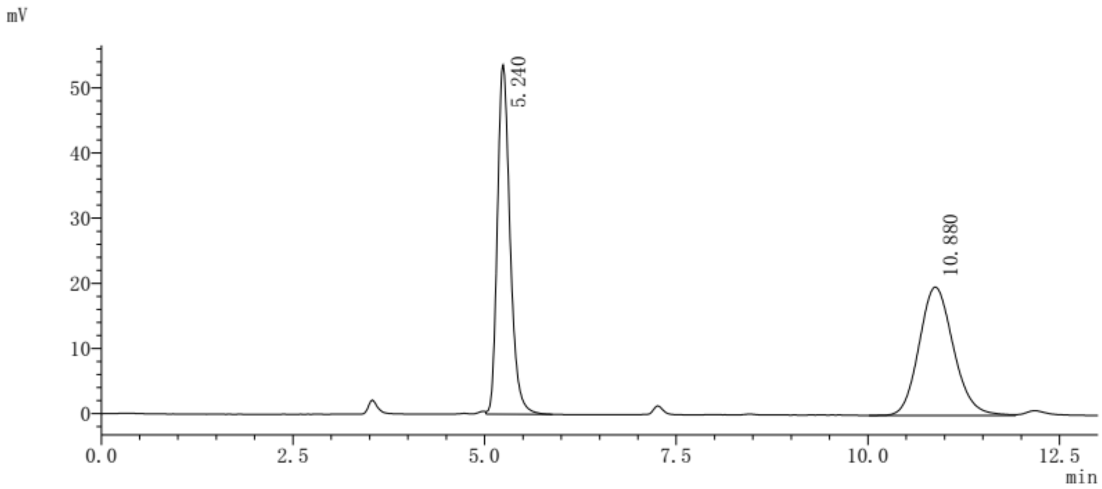

检测器A Ch2 254nm

Peak#	Ret.Time	Area	Area%
1	5.240	617284	50.118
2	10.880	614380	49.882
总计		1231664	100.000

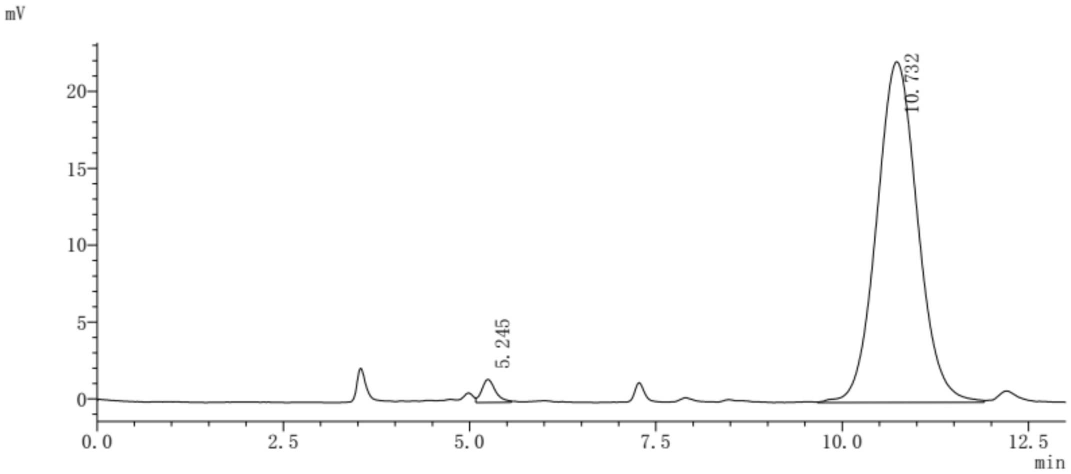

检测器A Ch2 254nm

Peak#	Ret.Time	Area	Area%
1	5.245	19358	2.230
2	10.732	848773	97.770
总计		868131	100.000

Supplementary Figure 16. HPLC spectra for compound **8a**.

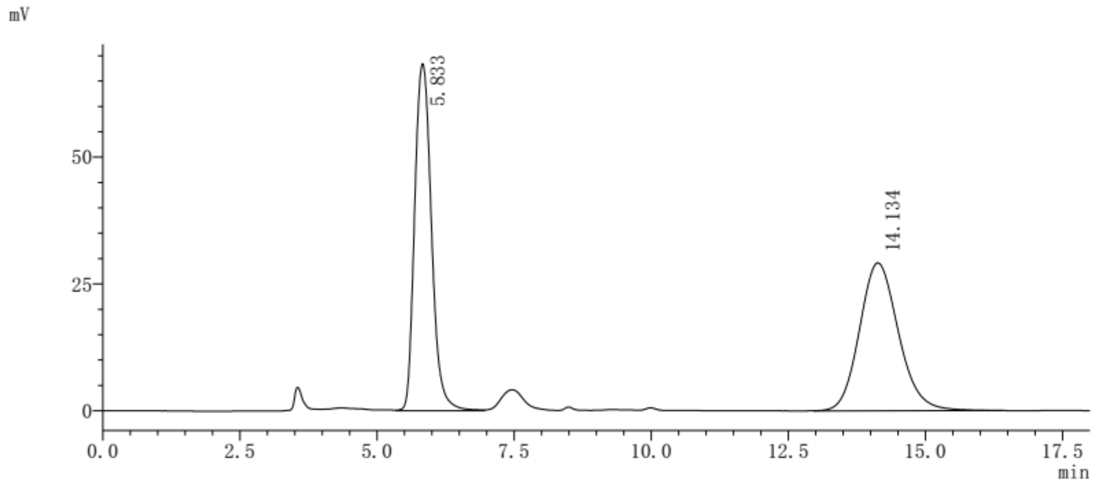

检测器A Ch2 254nm

Peak#	Ret.Time	Area	Area%
1	5.833	1461598	50.293
2	14.134	1444543	49.707
总计		2906141	100.000

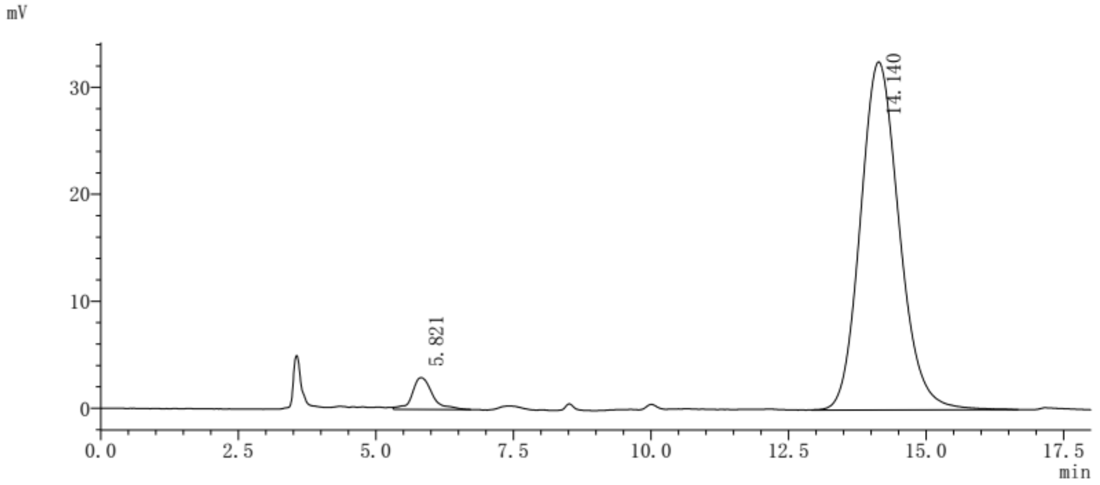

检测器A Ch2 254nm

Peak#	Ret.Time	Area	Area%
1	5.821	75779	4.527
2	14.140	1598255	95.473
总计		1674034	100.000

Supplementary Figure 17. HPLC spectra for compound **8b.**

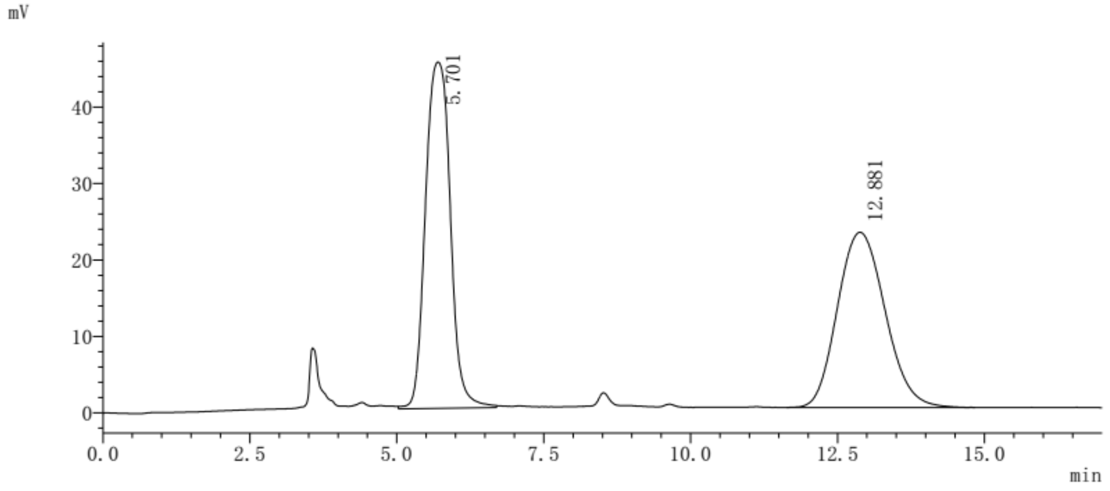

检测器A Ch2 254nm

Peak#	Ret.Time	Area	Area%
1	5.701	1334784	50.774
2	12.881	1294083	49.226
总计		2628867	100.000

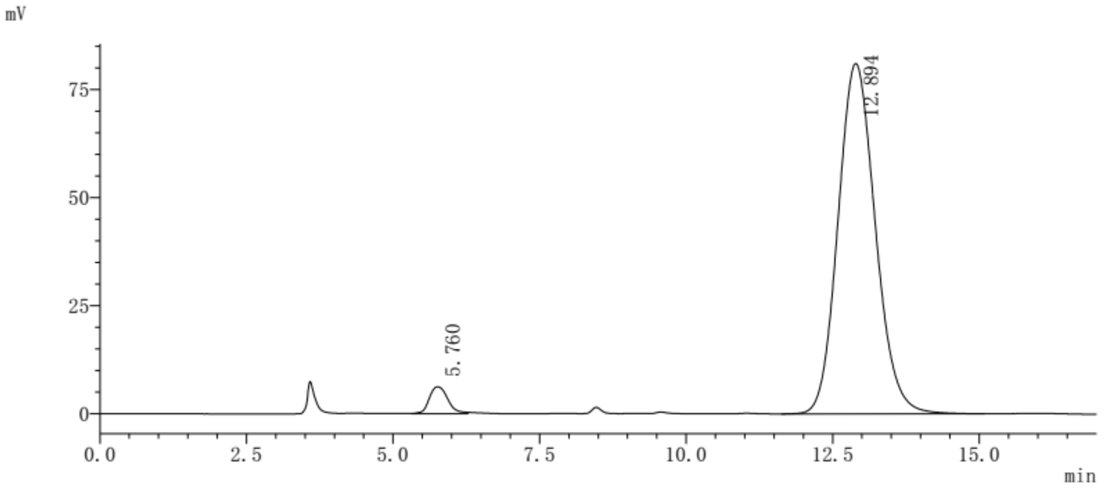

检测器A Ch2 254nm

Peak#	Ret.Time	Area	Area%
1	5.760	138630	3.718
2	12.894	3589606	96.282
总计		3728236	100.000

Supplementary Figure 18. HPLC spectra for compound **8c**.

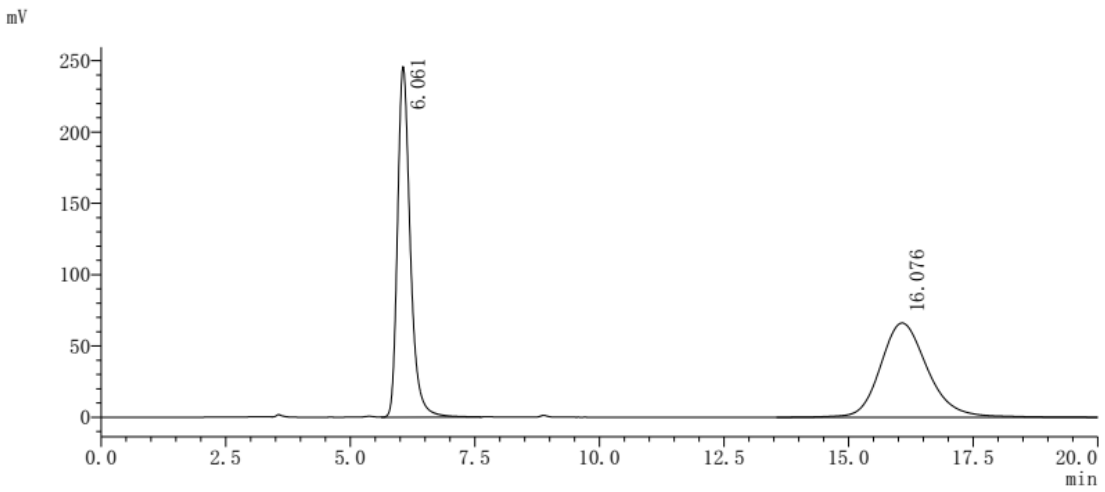

检测器A Ch2 254nm

Peak#	Ret.Time	Area	Area%
1	6.061	4384600	50.067
2	16.076	4372840	49.933
总计		8757441	100.000

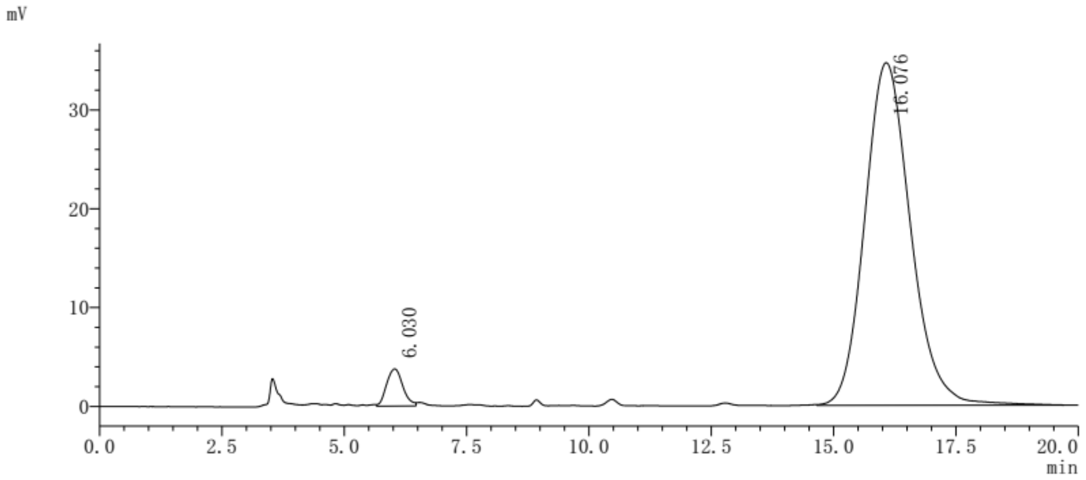

检测器A Ch2 254nm

Peak#	Ret.Time	Area	Area%
1	6.030	86927	3.715
2	16.076	2252775	96.285
总计		2339701	100.000

Supplementary Figure 19. HPLC spectra for compound **8d**.

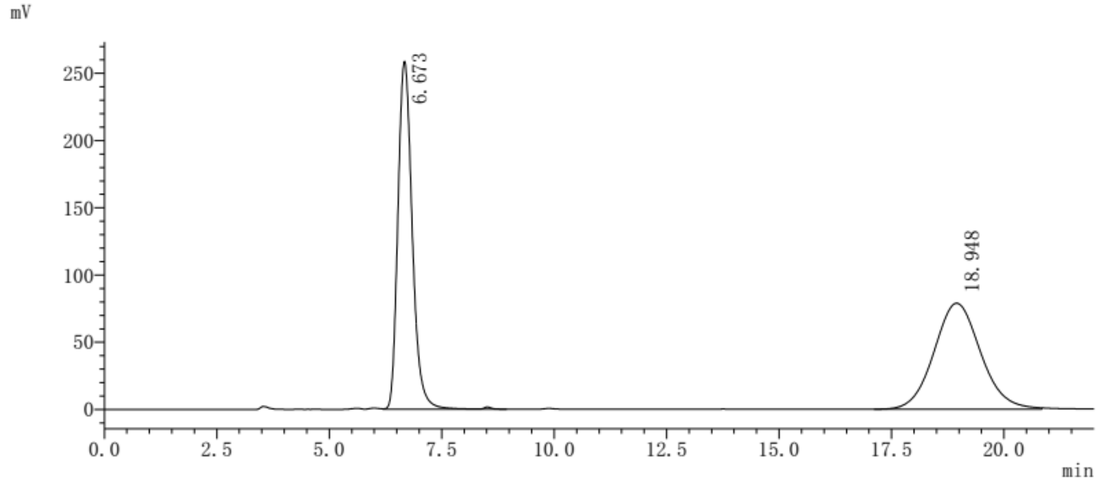

检测器A Ch2 254nm

Peak#	Ret.Time	Area	Area%
1	6.673	5750697	50.280
2	18.948	5686738	49.720
总计		11437435	100.000

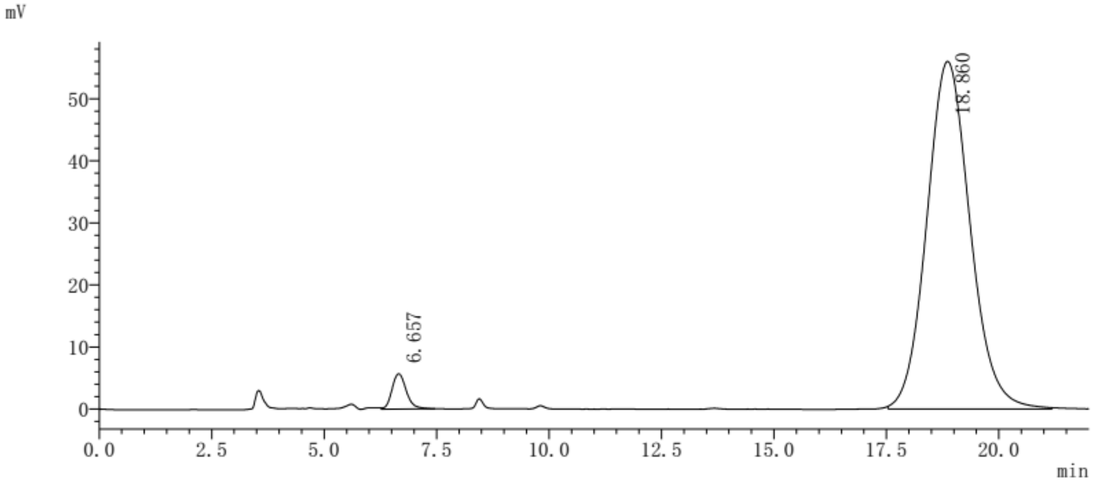

检测器A Ch2 254nm

Peak#	Ret.Time	Area	Area%
1	6.657	125986	3.249
2	18.860	3752276	96.751
总计		3878262	100.000

Supplementary Figure 20. HPLC spectra for compound **8e**.

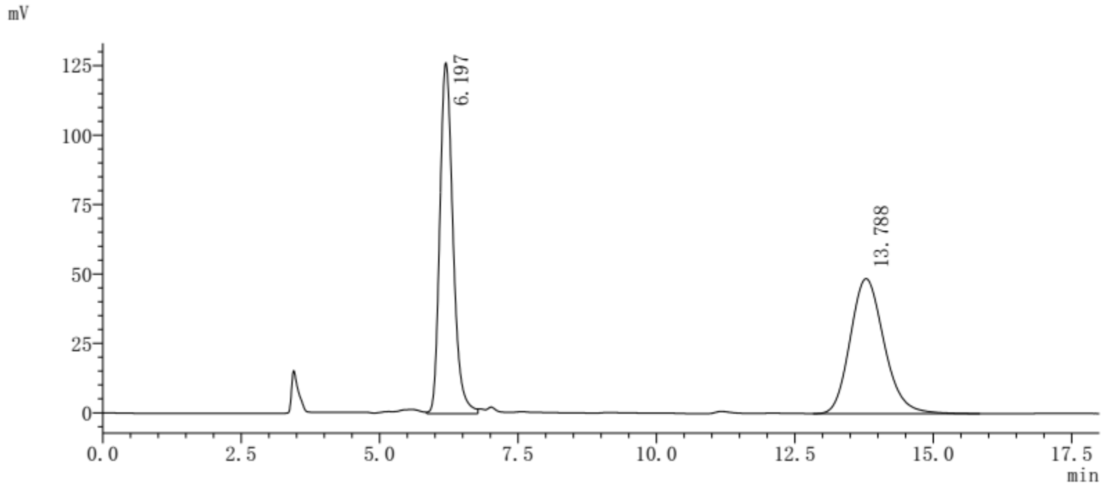

检测器A Ch2 254nm

Peak#	Ret.Time	Area	Area%
1	6.197	2091729	50.218
2	13.788	2073603	49.782
总计		4165332	100.000

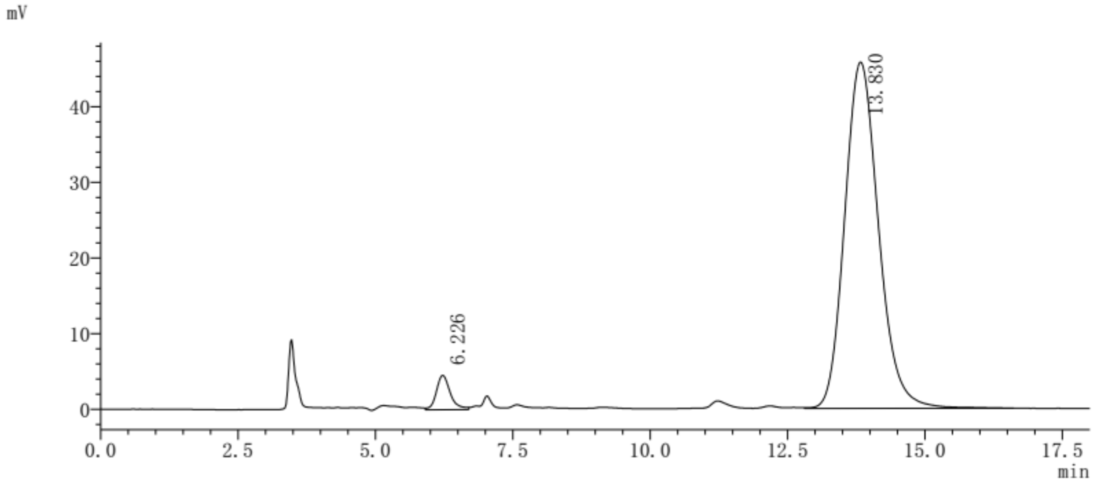

检测器A Ch2 254nm

Peak#	Ret.Time	Area	Area%
1	6.226	79836	3.908
2	13.830	1963081	96.092
总计		2042917	100.000

Supplementary Figure 21. HPLC spectra for compound **8f**.

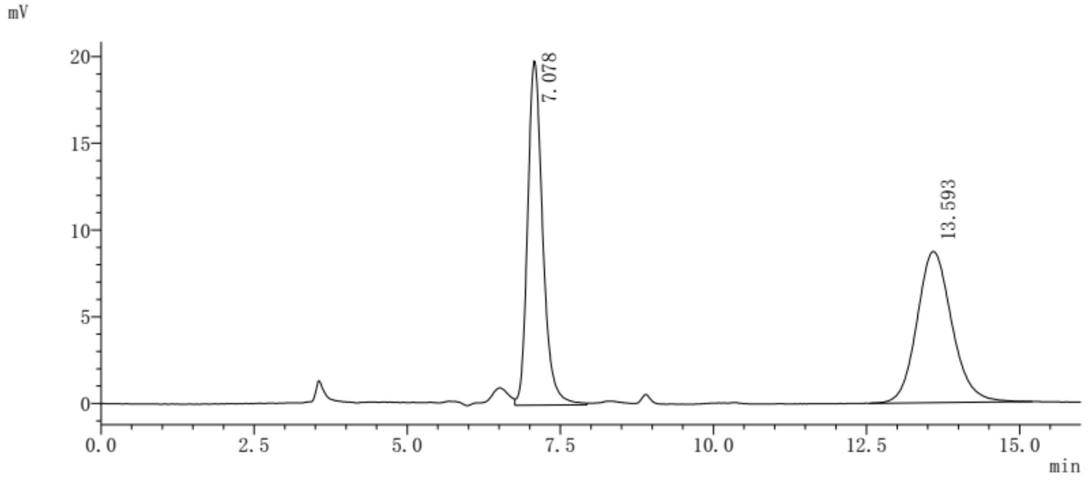

检测器A Ch2 254nm

Peak#	Ret.Time	Area	Area%
1	7.078	339777	49.535
2	13.593	346161	50.465
总计		685939	100.000

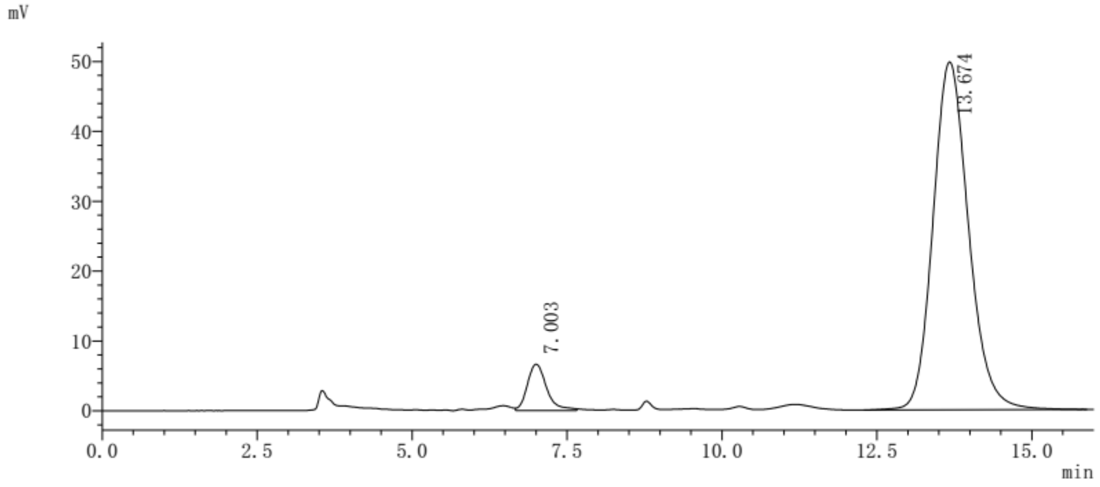

检测器A Ch2 254nm

Peak#	Ret.Time	Area	Area%
1	7.003	144280	6.765
2	13.674	1988435	93.235
总计		2132715	100.000

Supplementary Figure 22. HPLC spectra for compound **8g**.

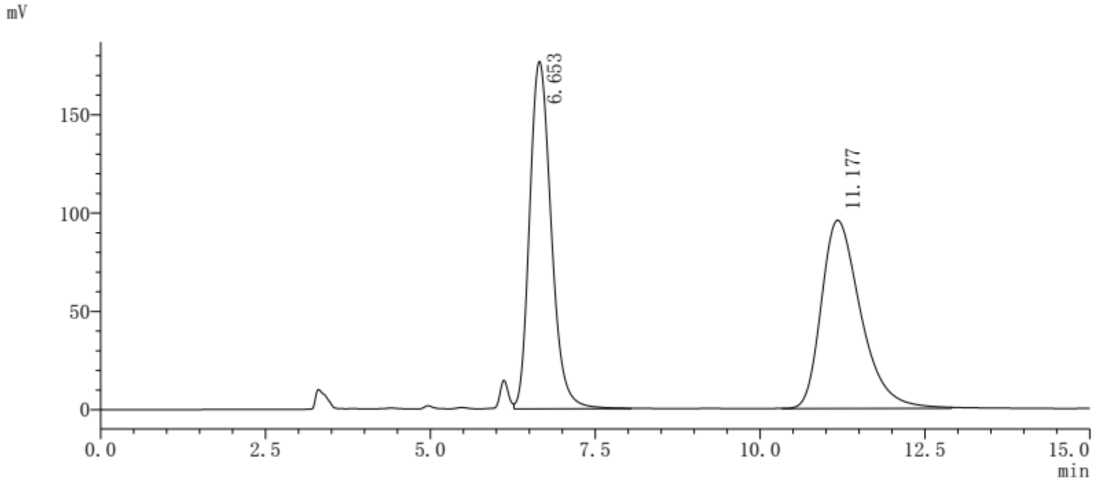

检测器A Ch2 254nm

Peak#	Ret.Time	Area	Area%
1	6.653	4025346	50.241
2	11.177	3986777	49.759
总计		8012123	100.000

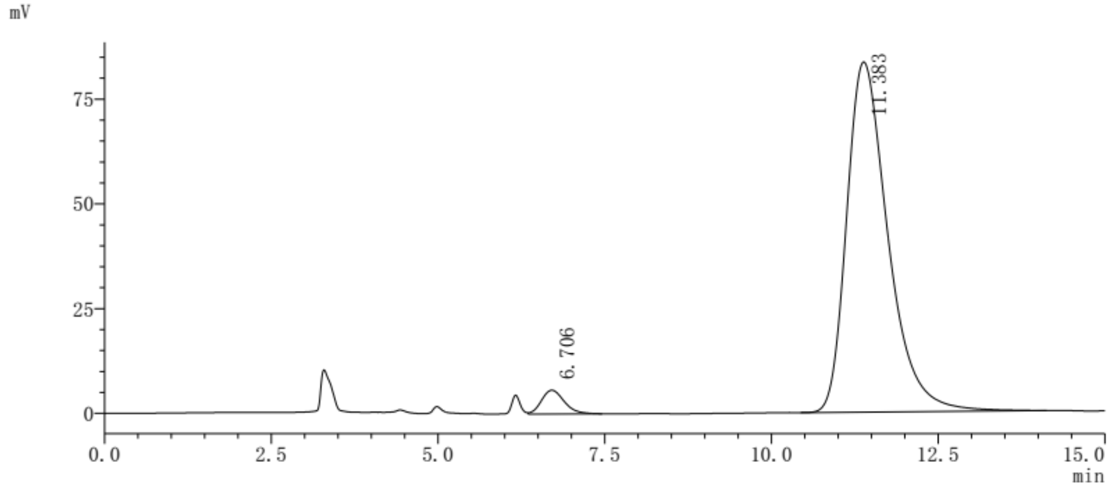

检测器A Ch2 254nm

Peak#	Ret.Time	Area	Area%
1	6.706	136035	3.639
2	11.383	3602719	96.361
总计		3738753	100.000

Supplementary Figure 23. HPLC spectra for compound **6b**.

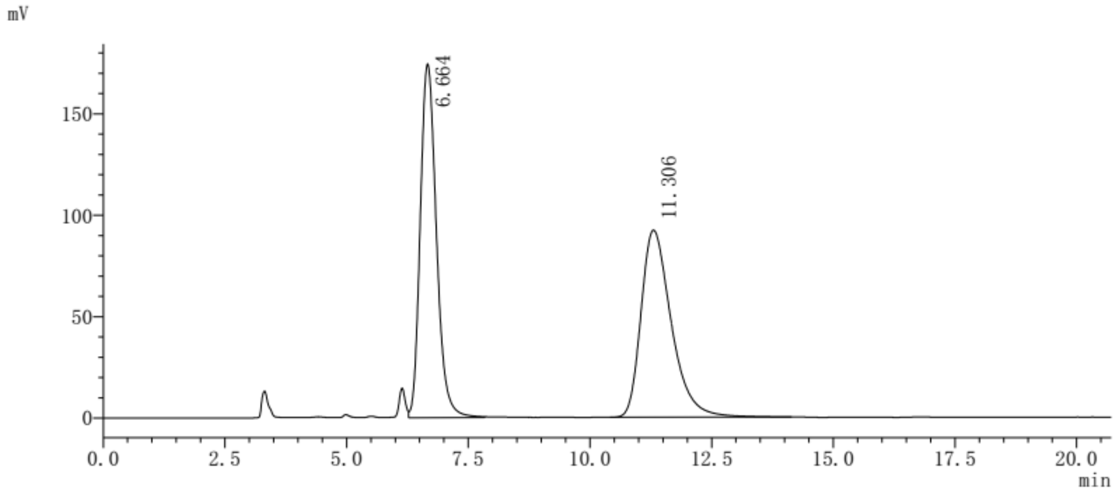

检测器A Ch2 254nm

Peak#	Ret.Time	Area	Area%
1	6.664	4018751	50.137
2	11.306	3996830	49.863
总计		8015581	100.000

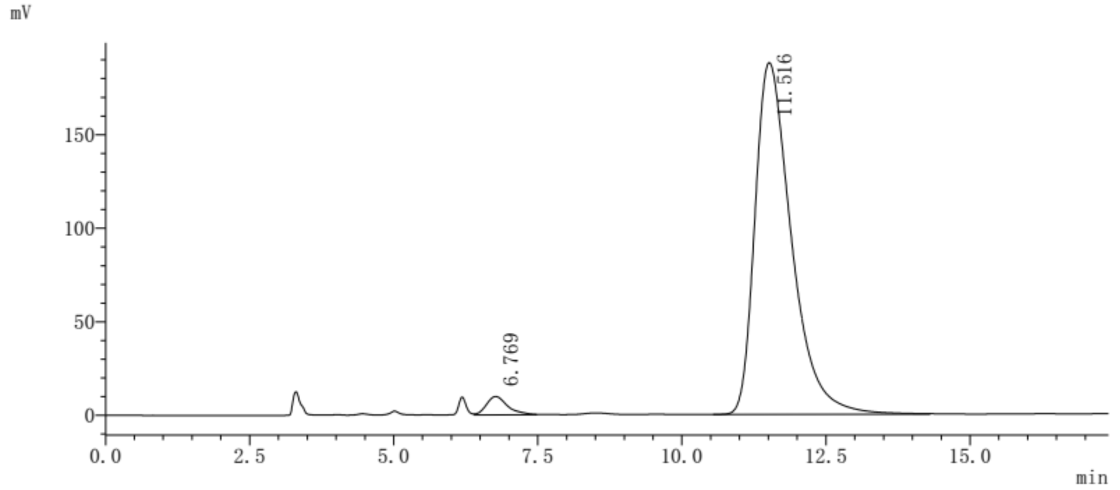

检测器A Ch2 254nm

Peak#	Ret.Time	Area	Area%
1	6.769	253521	2.967
2	11.516	8292387	97.033
总计		8545908	100.000

Supplementary Figure 24. HPLC spectra for compound **6e**.

7. Copies of NMR spectra.

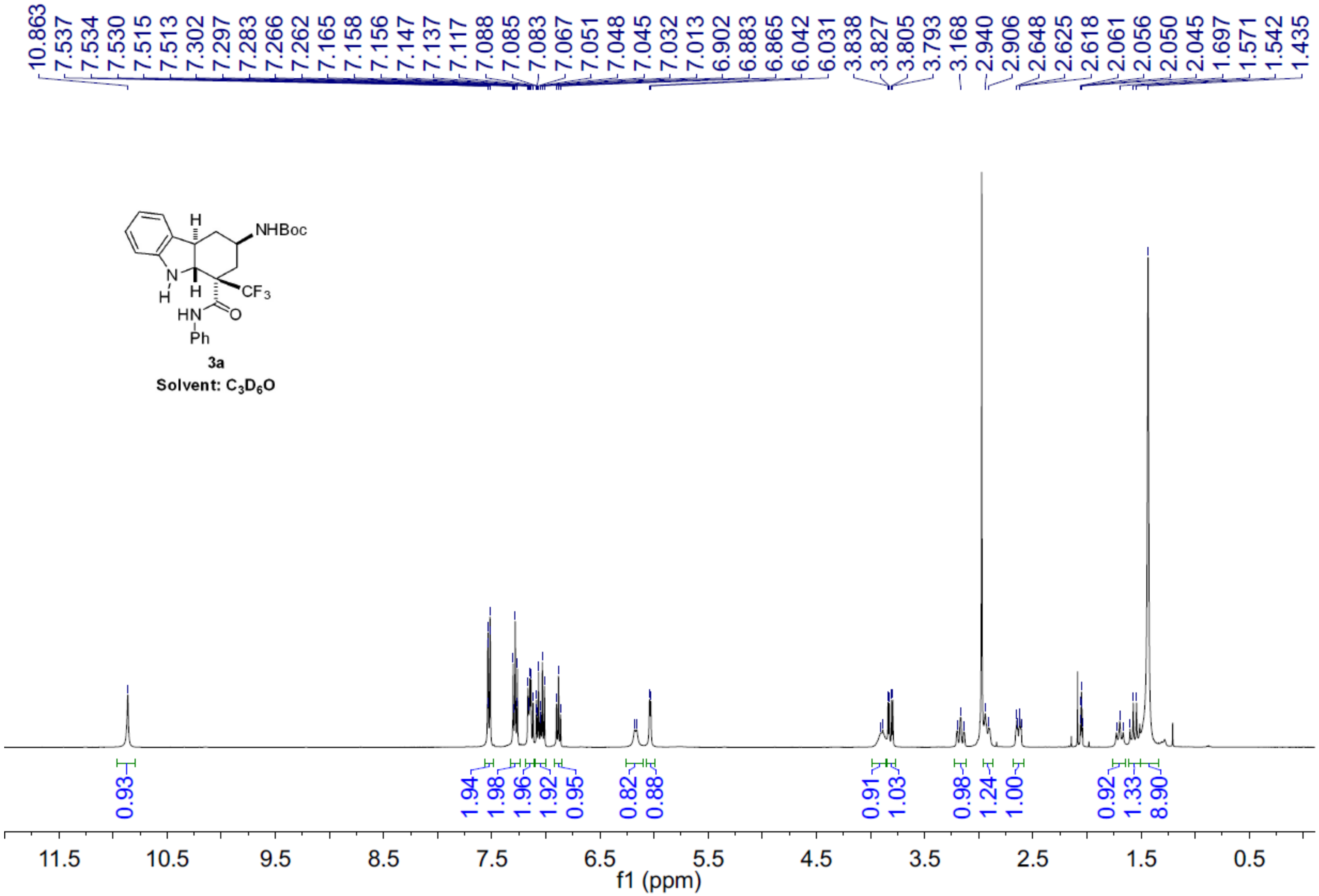

Supplementary Figure 25. ^{1}H NMR spectrum for compound **3a**

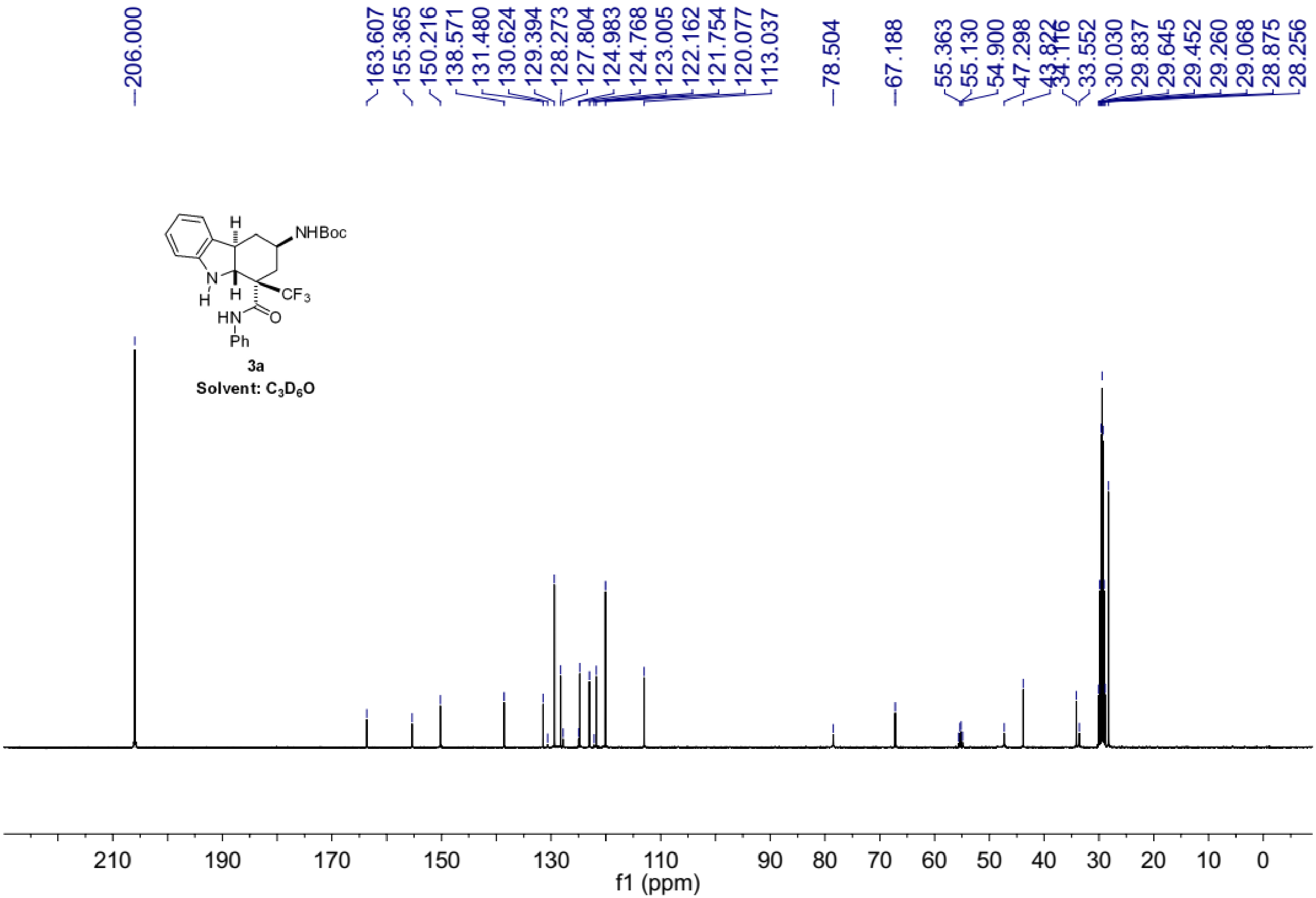

Supplementary Figure 26. ^{13}C NMR spectrum for compound **3a**

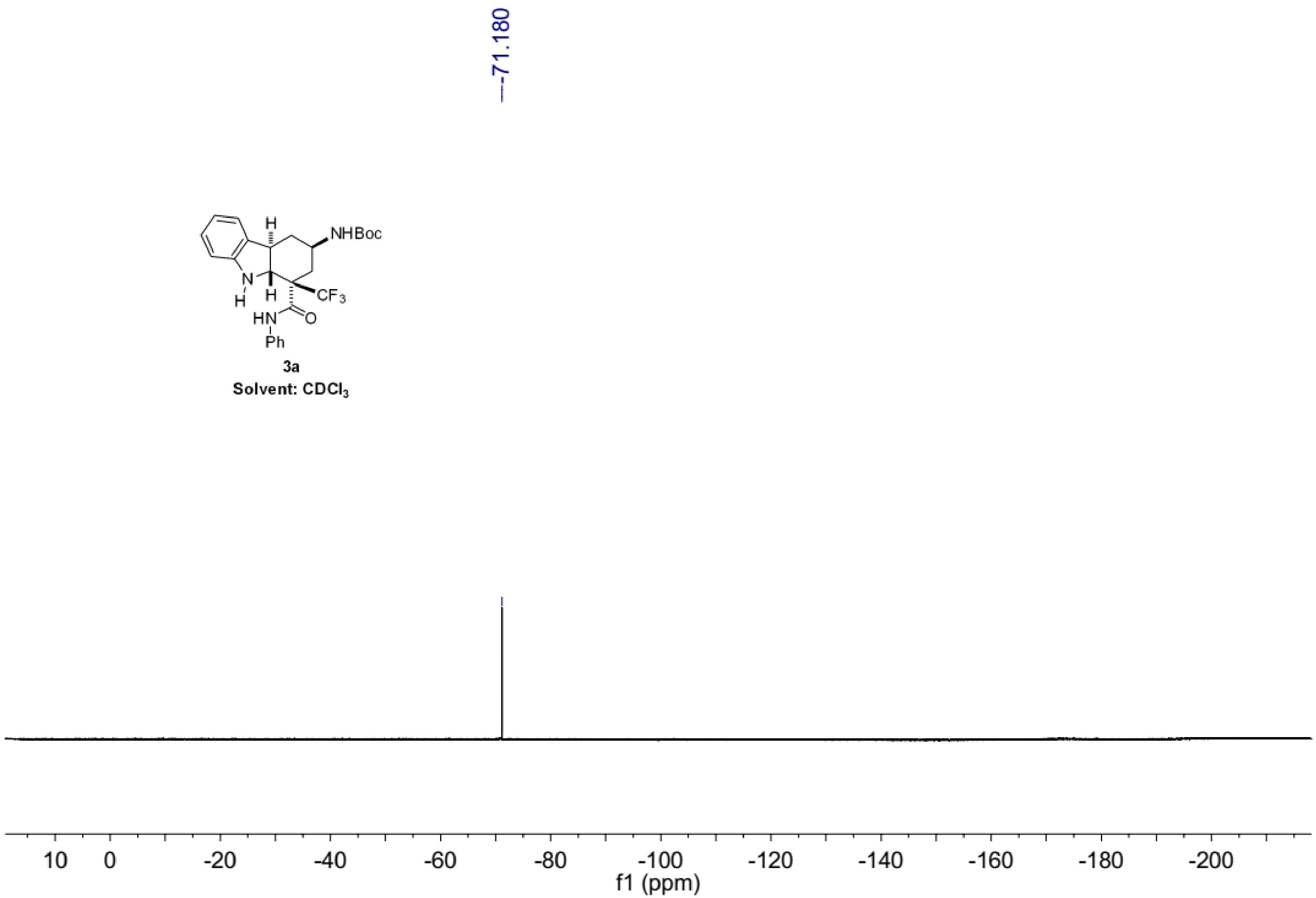

Supplementary Figure 27. ^{19}F NMR spectrum for compound **3a**

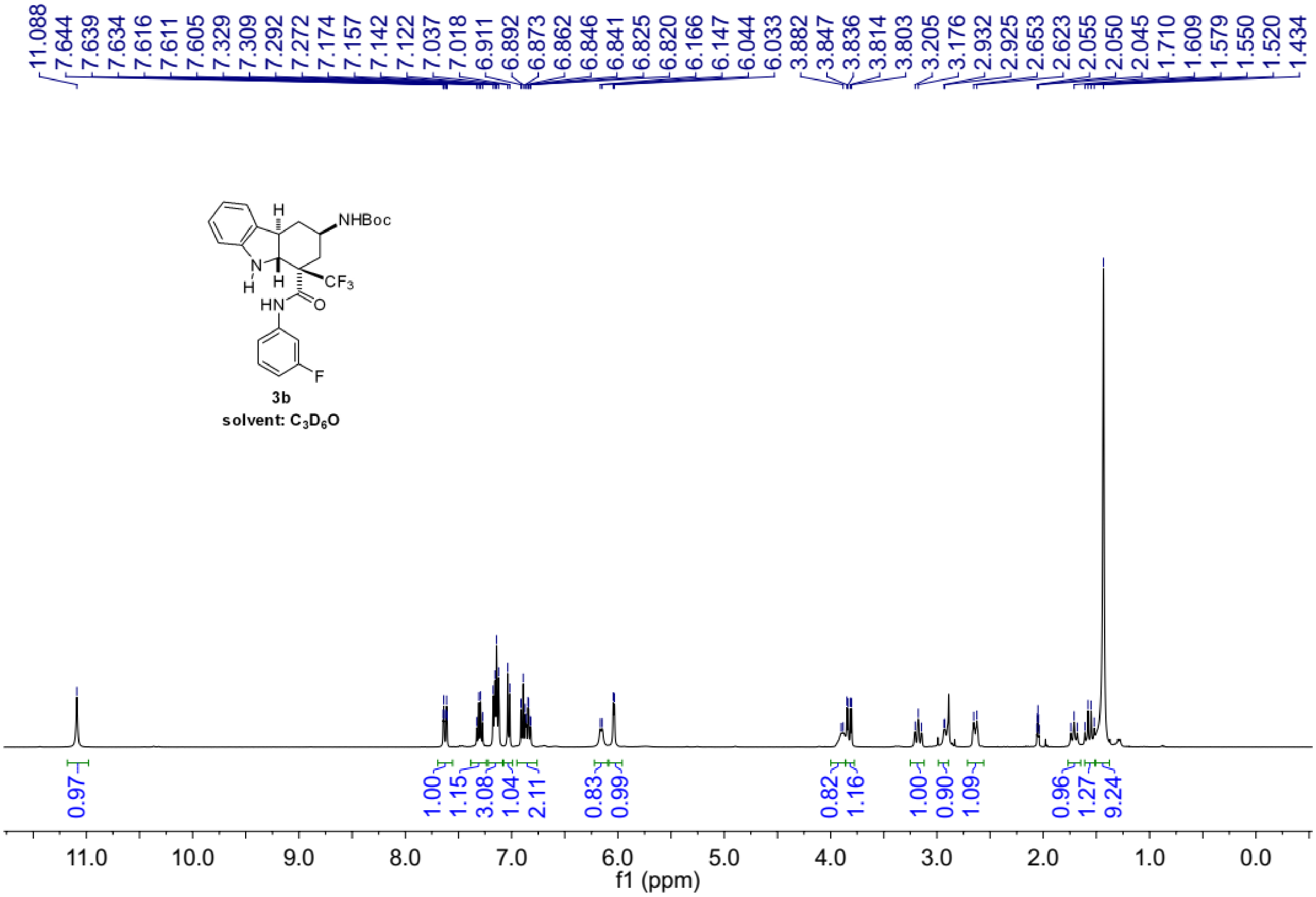

Supplementary Figure 28. ^{1}H NMR spectrum for compound **3b**

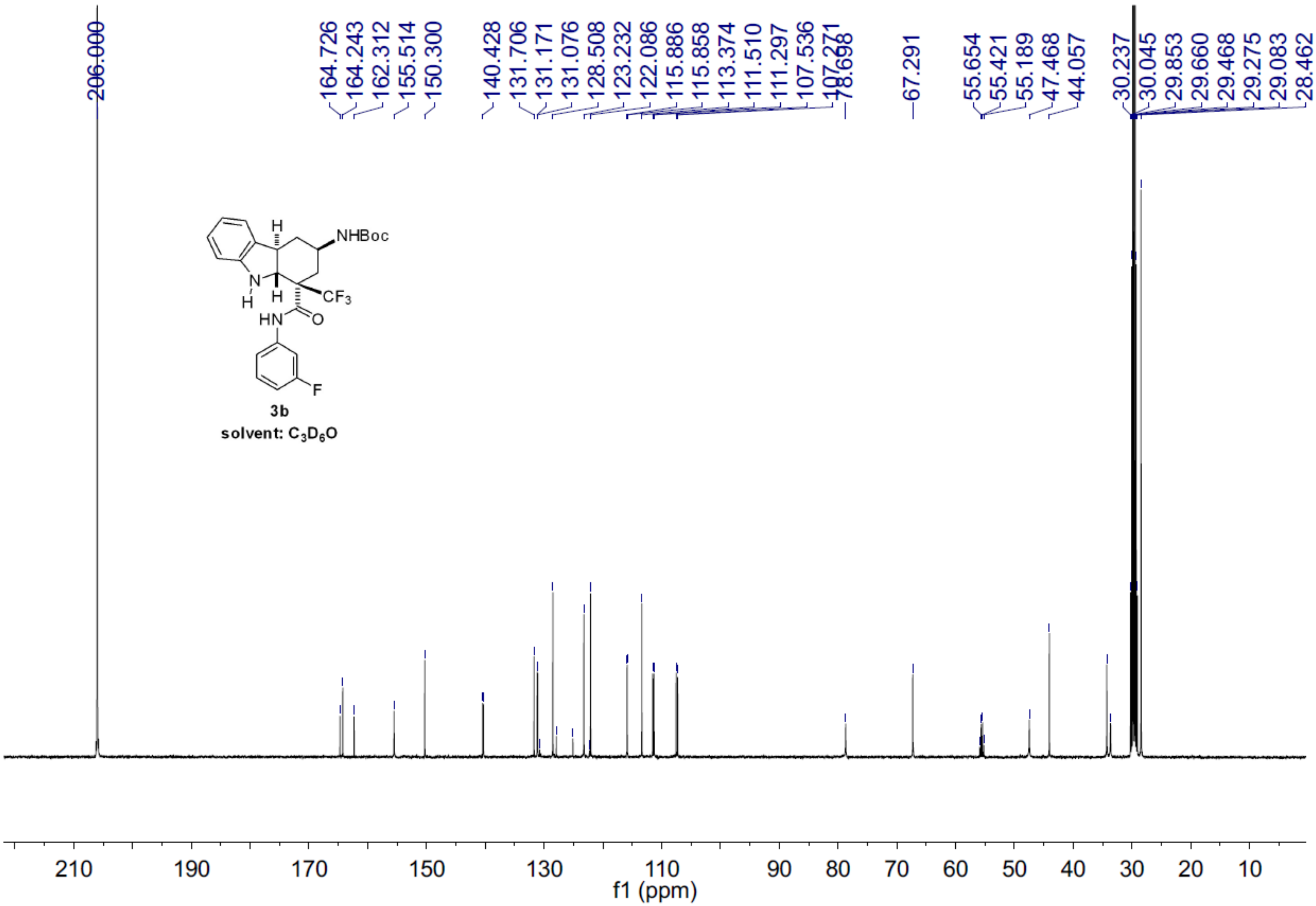

Supplementary Figure 29. ^{13}C NMR spectrum for compound **3b**

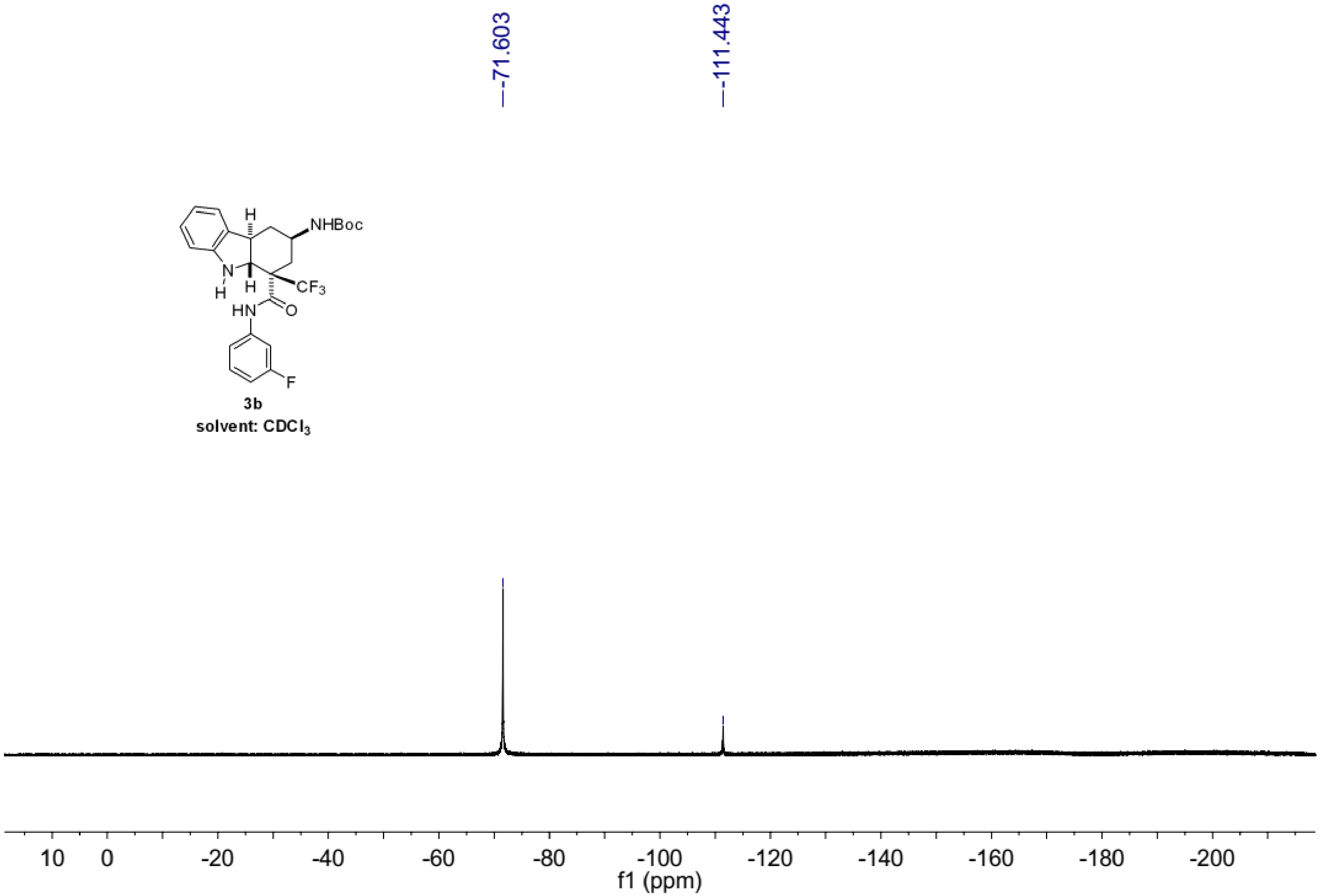

Supplementary Figure 30. ^{19}F NMR spectrum for compound **3b**

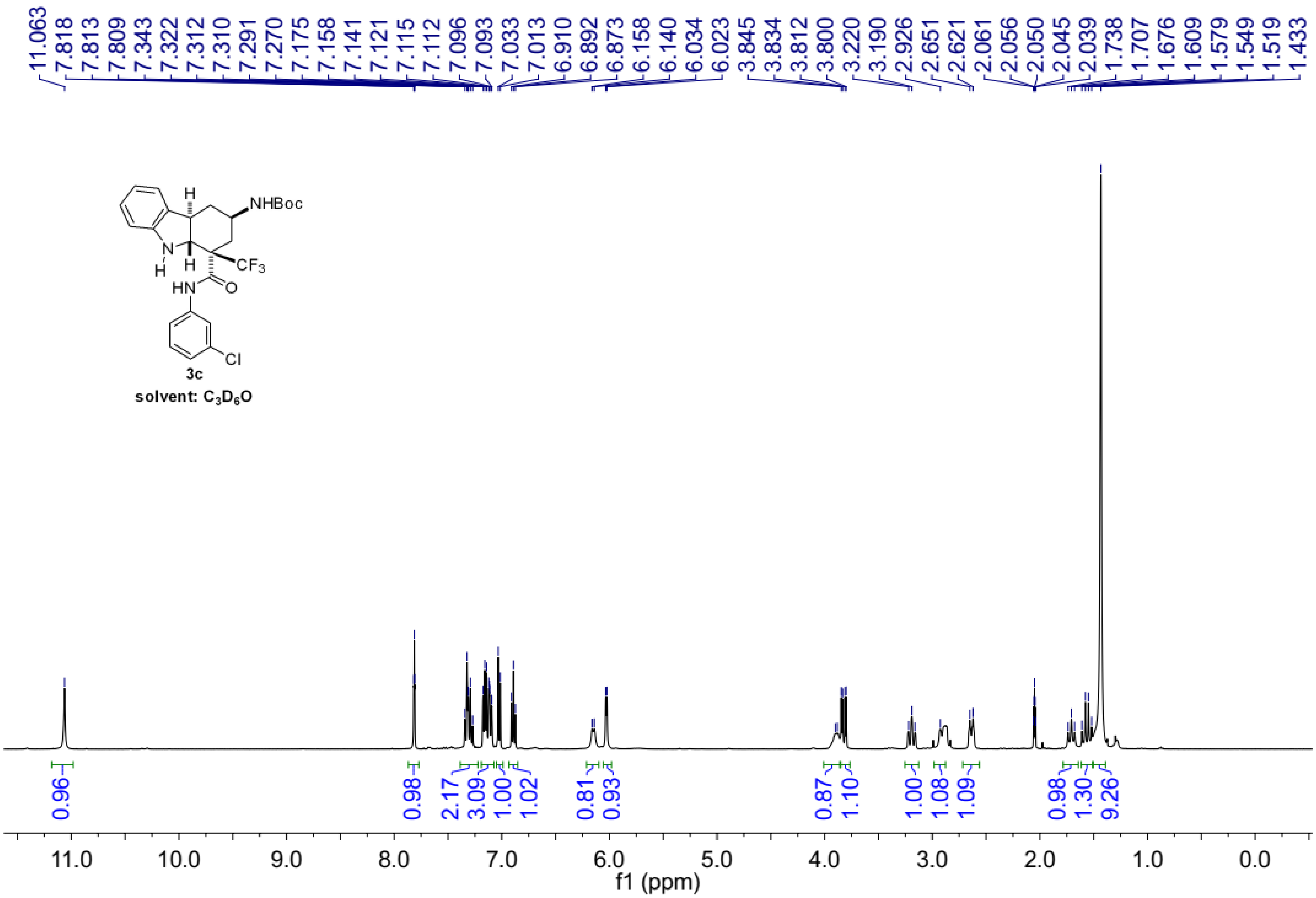

Supplementary Figure 31. ^{1}H NMR spectrum for compound **3c**

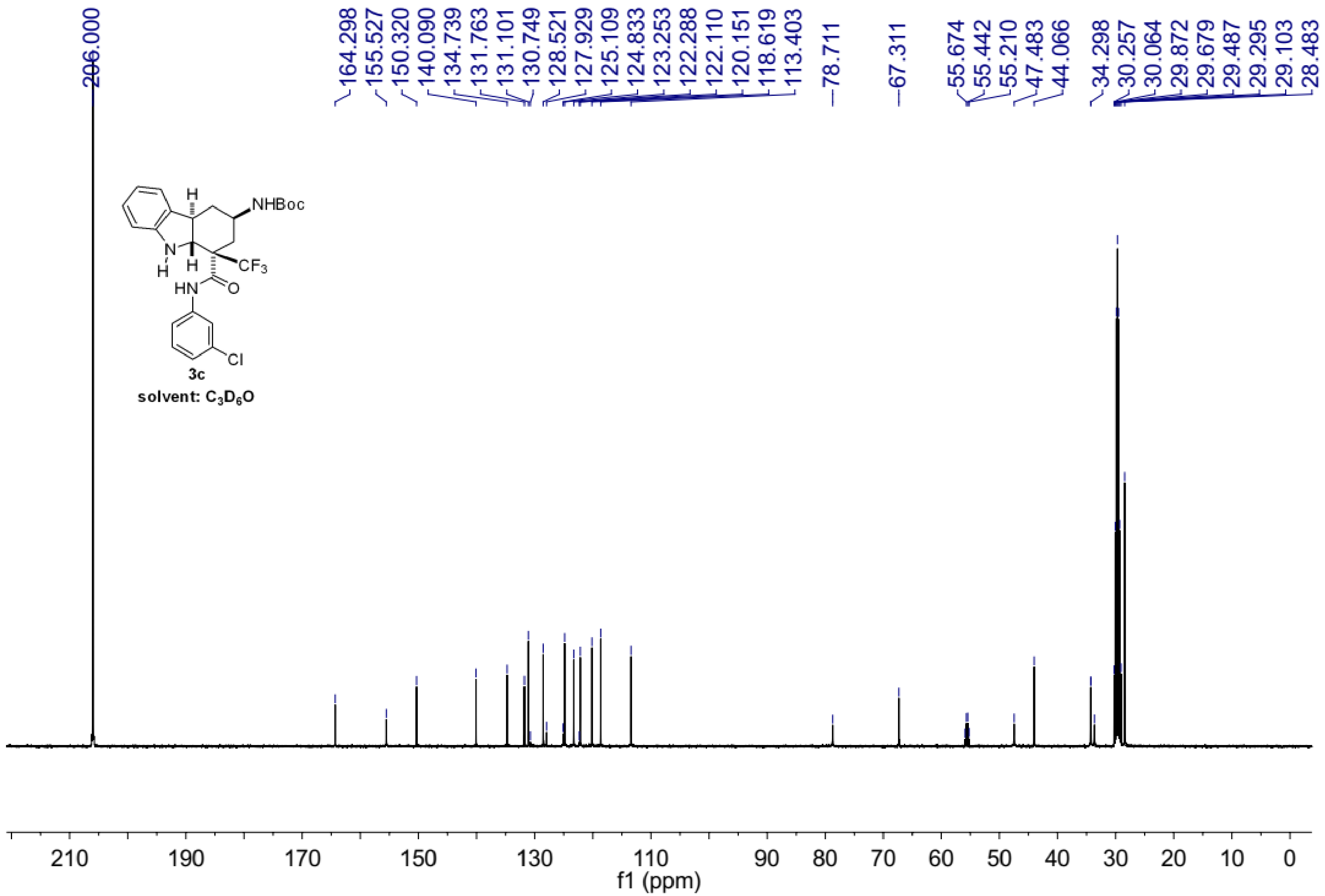

Supplementary Figure 32. ^{13}C NMR spectrum for compound **3c**

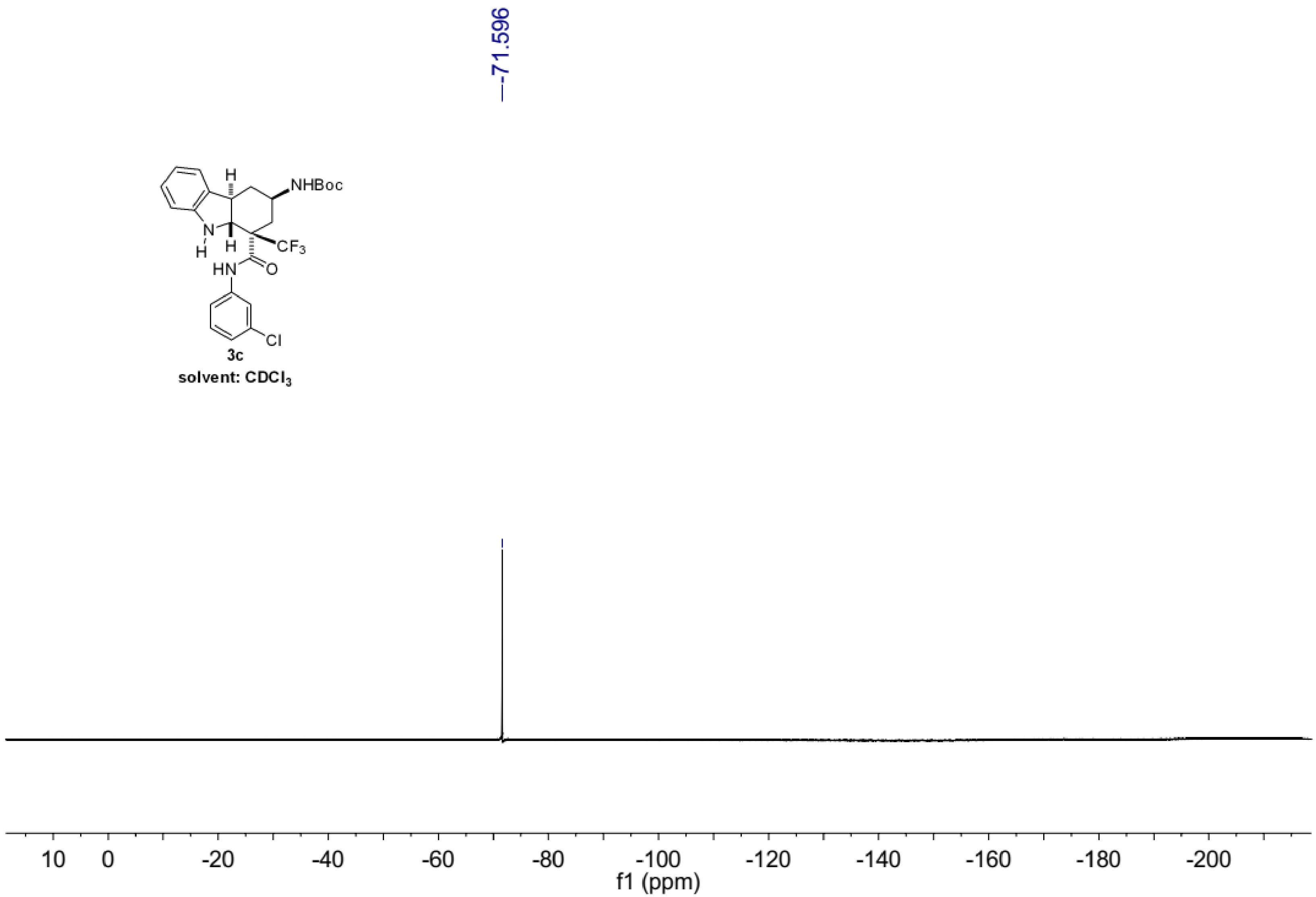

Supplementary Figure 33. ^{19}F NMR spectrum for compound **3c**

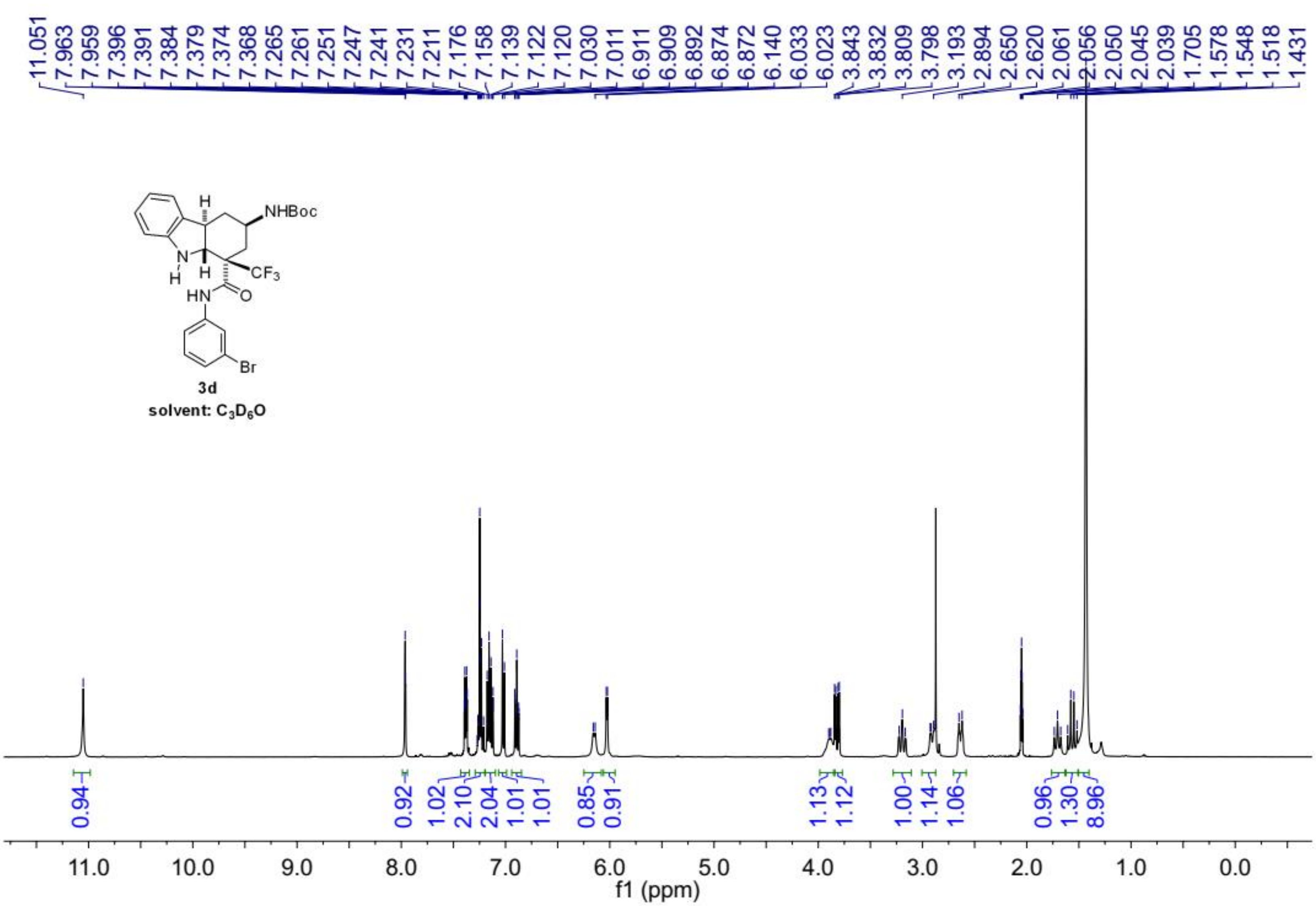

Supplementary Figure 34. ^{1}H NMR spectrum for compound **3d**

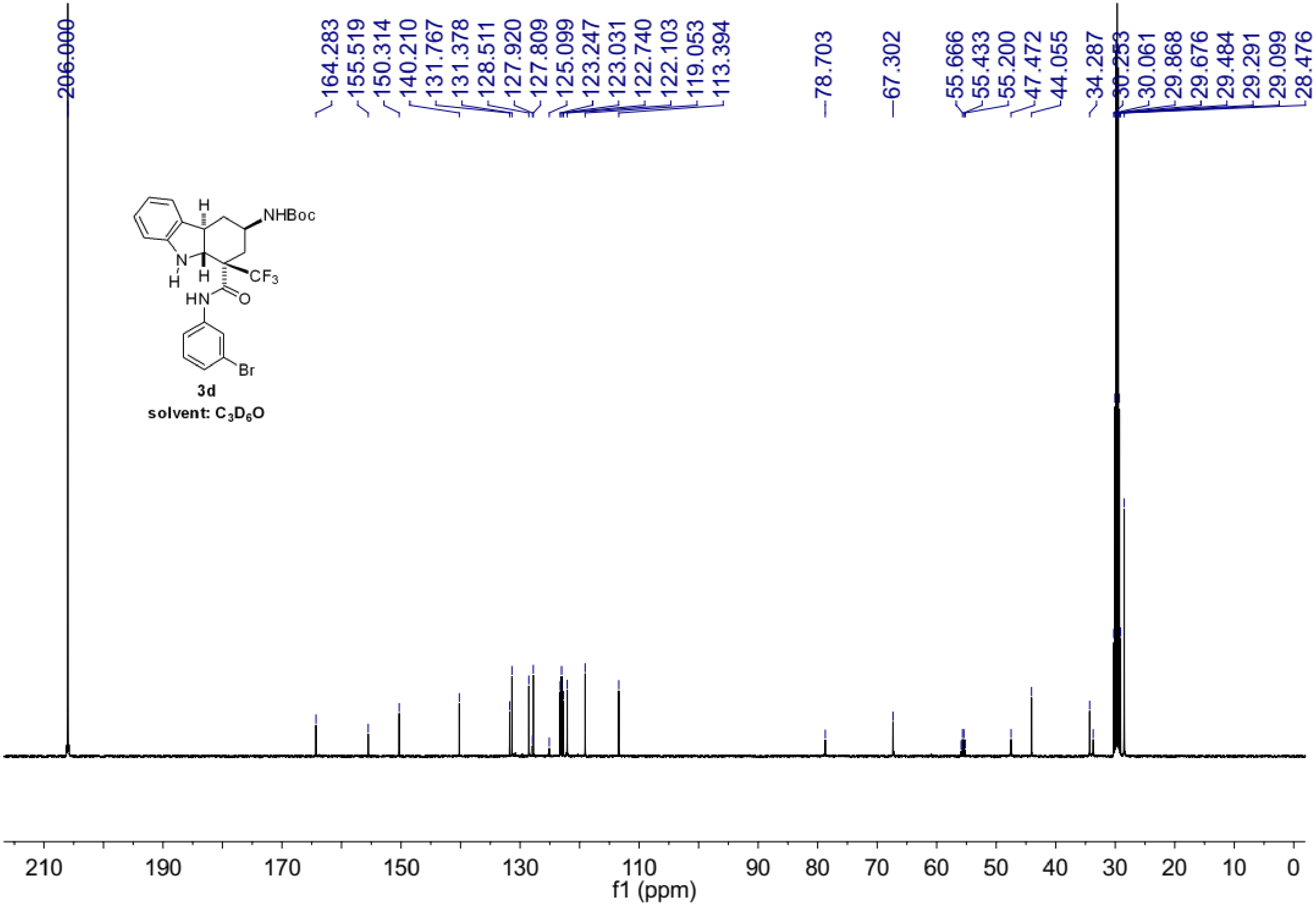

Supplementary Figure 35. ^{13}C NMR spectrum for compound **3d**

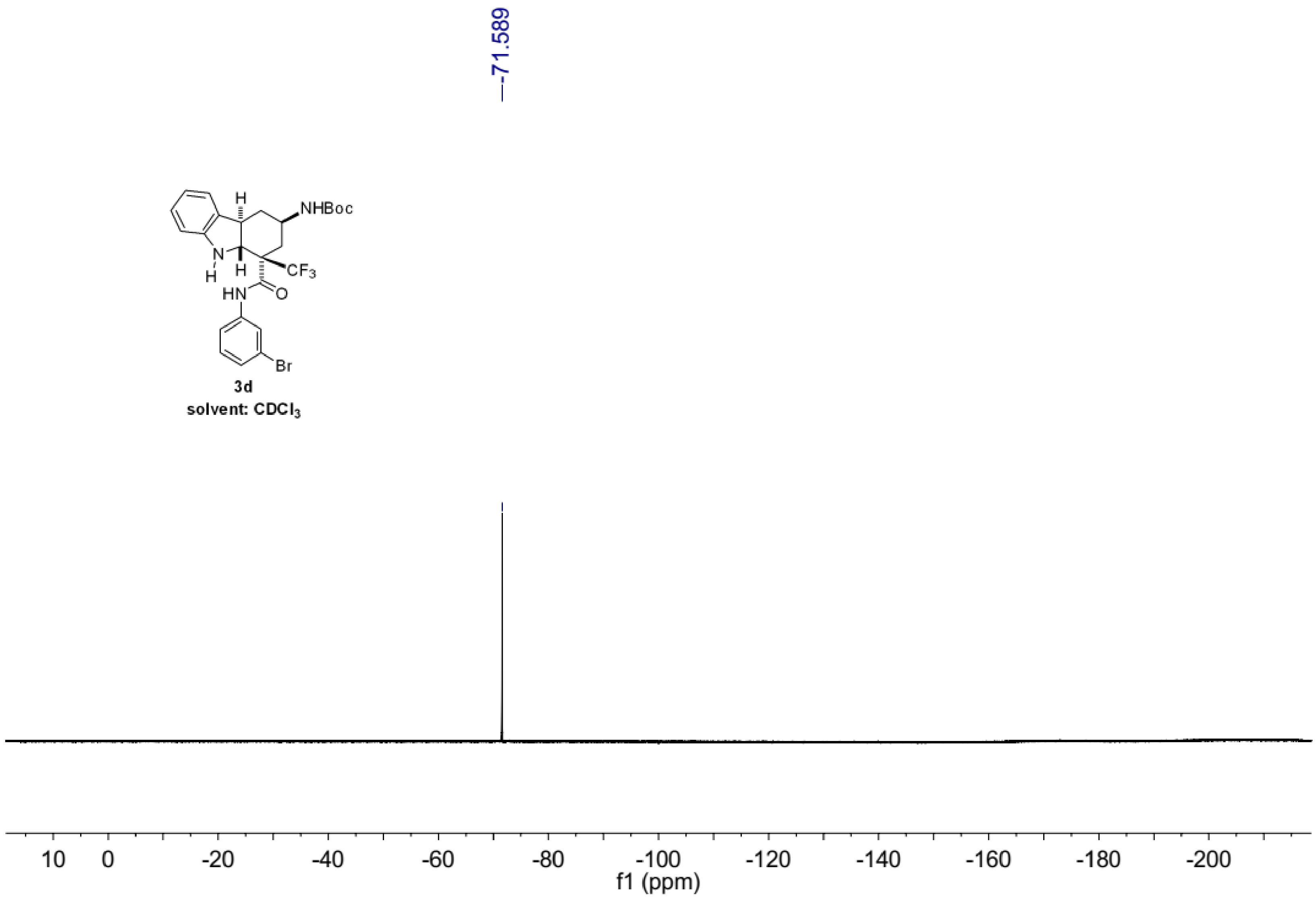

Supplementary Figure 36. ^{19}F NMR spectrum for compound **3d**

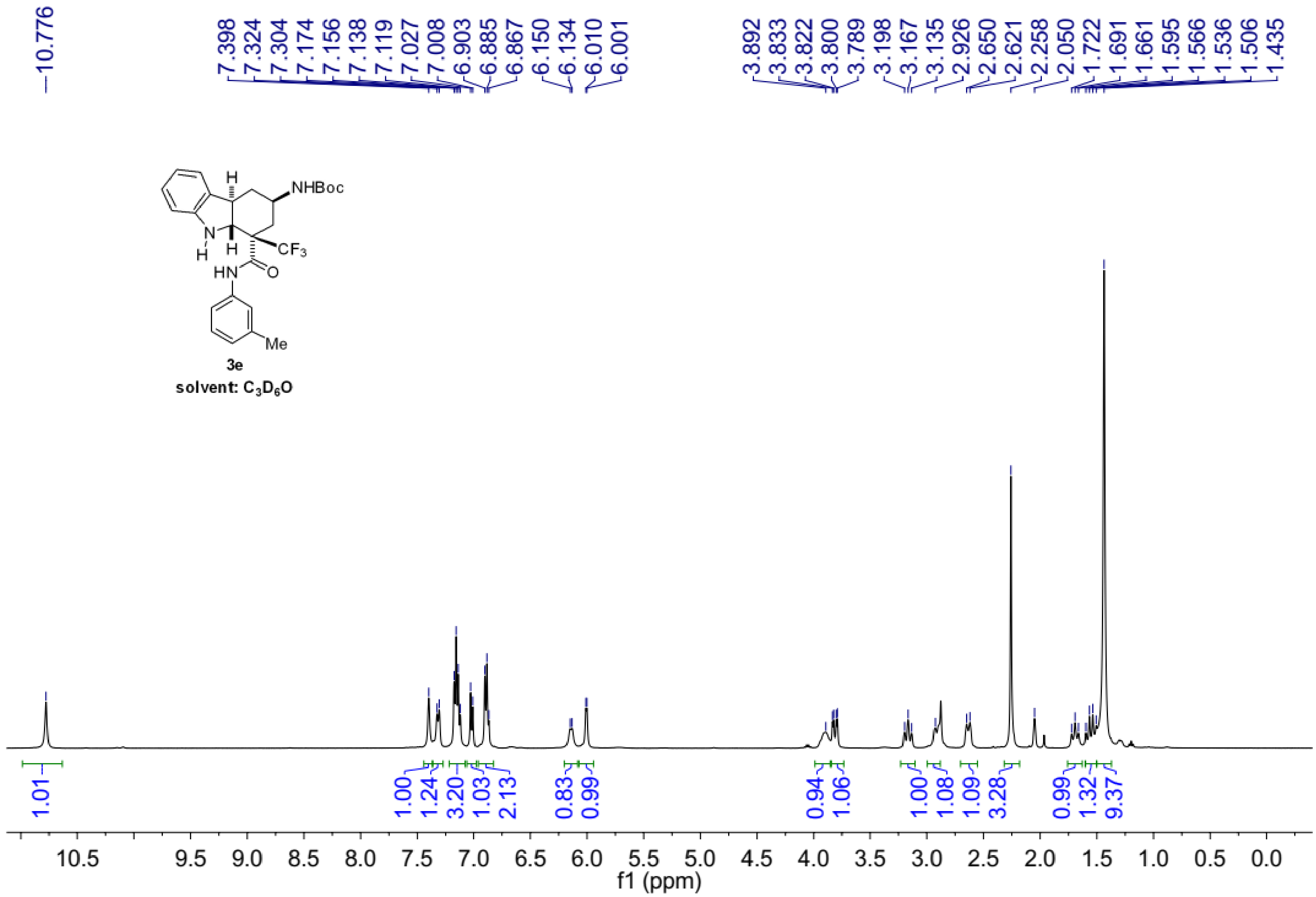

Supplementary Figure 37. ^{1}H NMR spectrum for compound **3e**

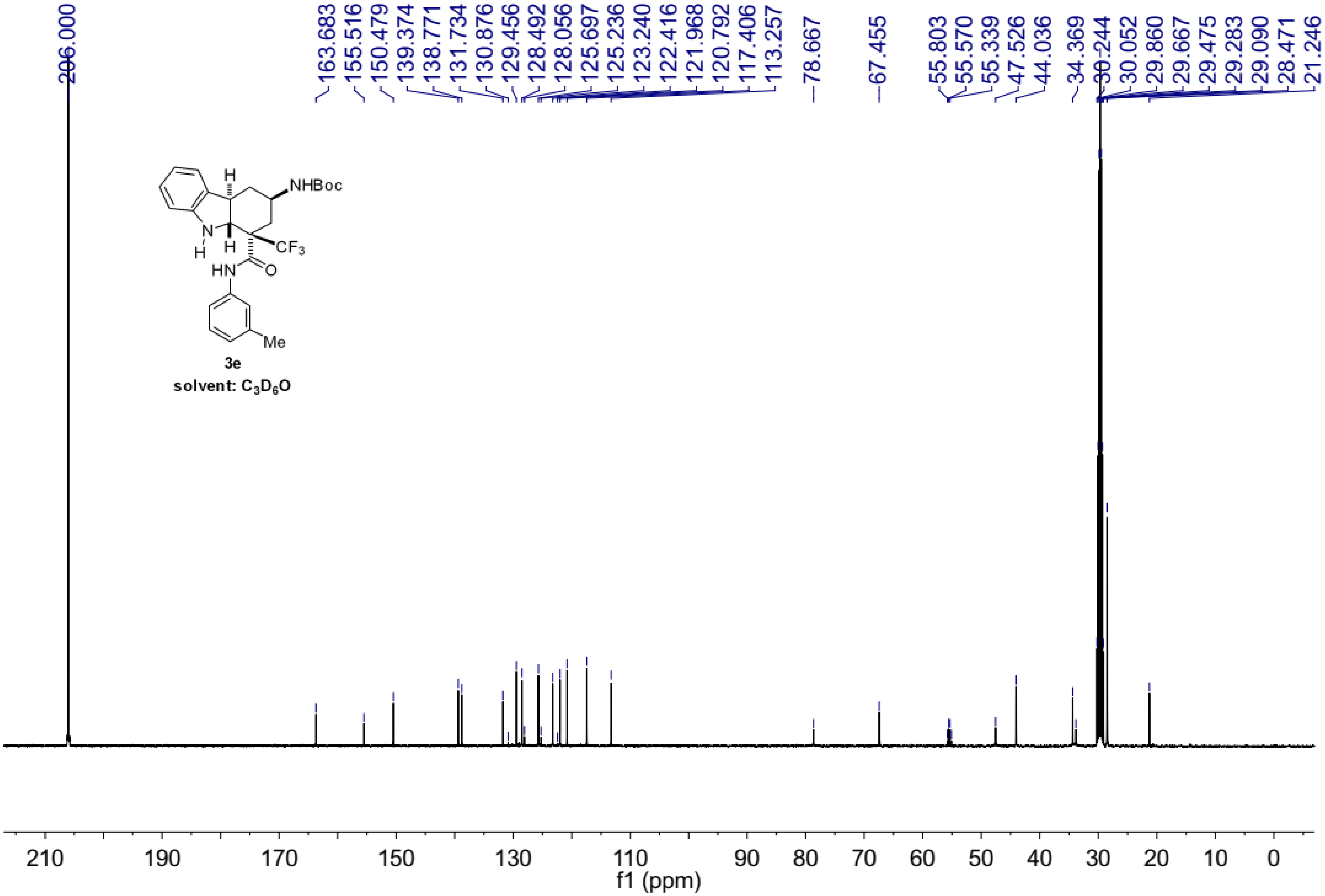

Supplementary Figure 38. ^{13}C NMR spectrum for compound **3e**

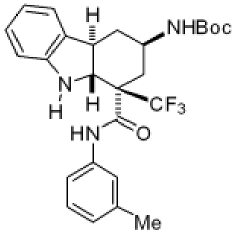

3e
solvent: CDCl$_3$

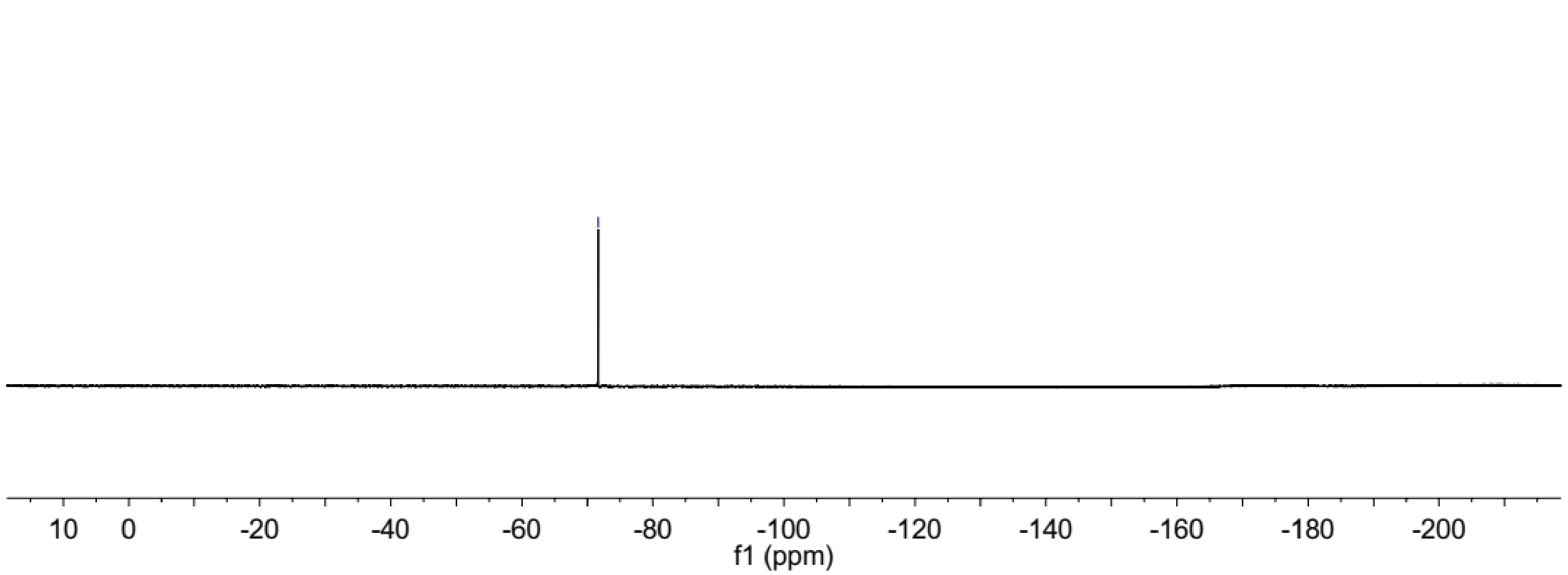

Supplementary Figure 39. ^{19}F NMR spectrum for compound **3e**

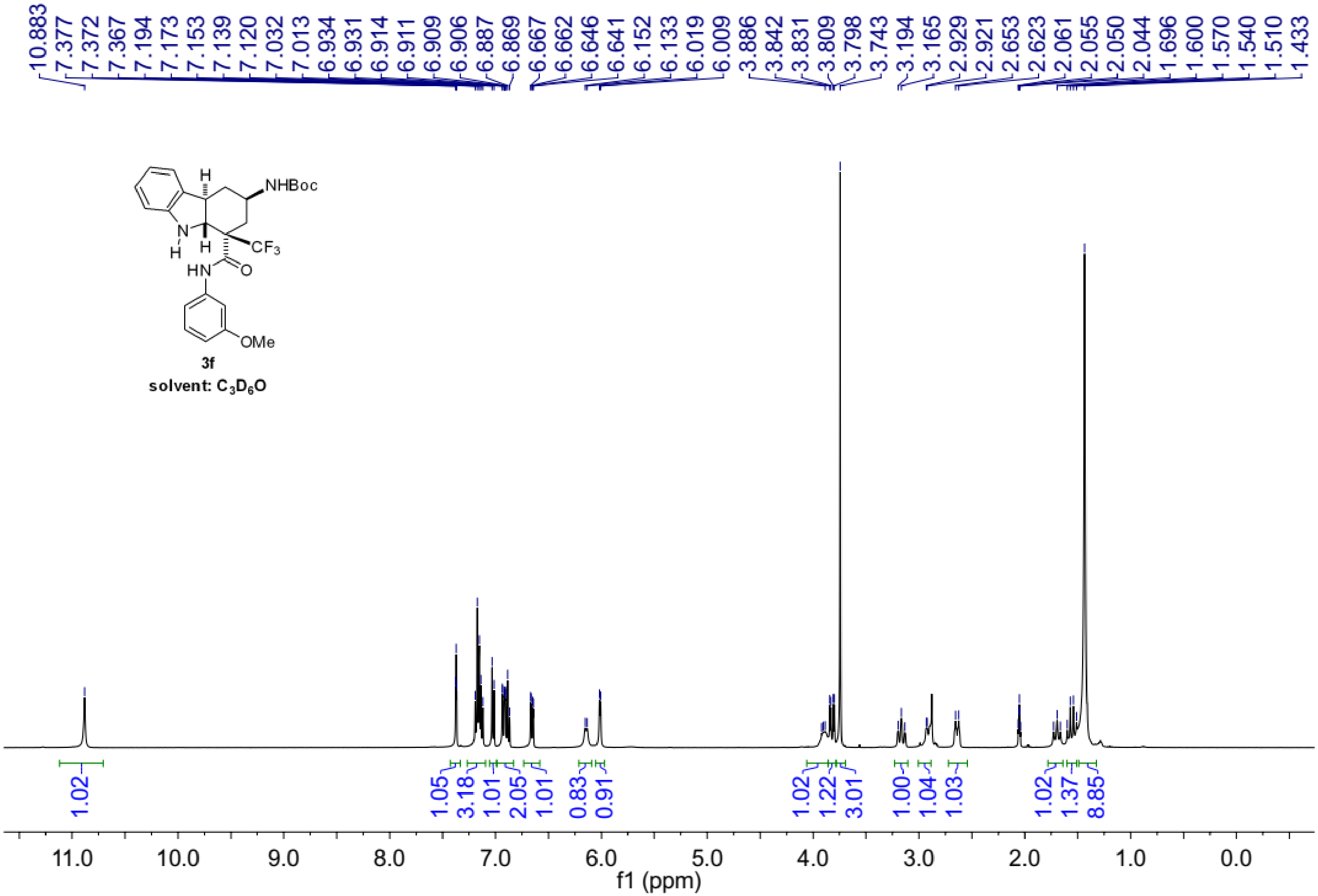

Supplementary Figure 40. ^{1}H NMR spectrum for compound **3f**

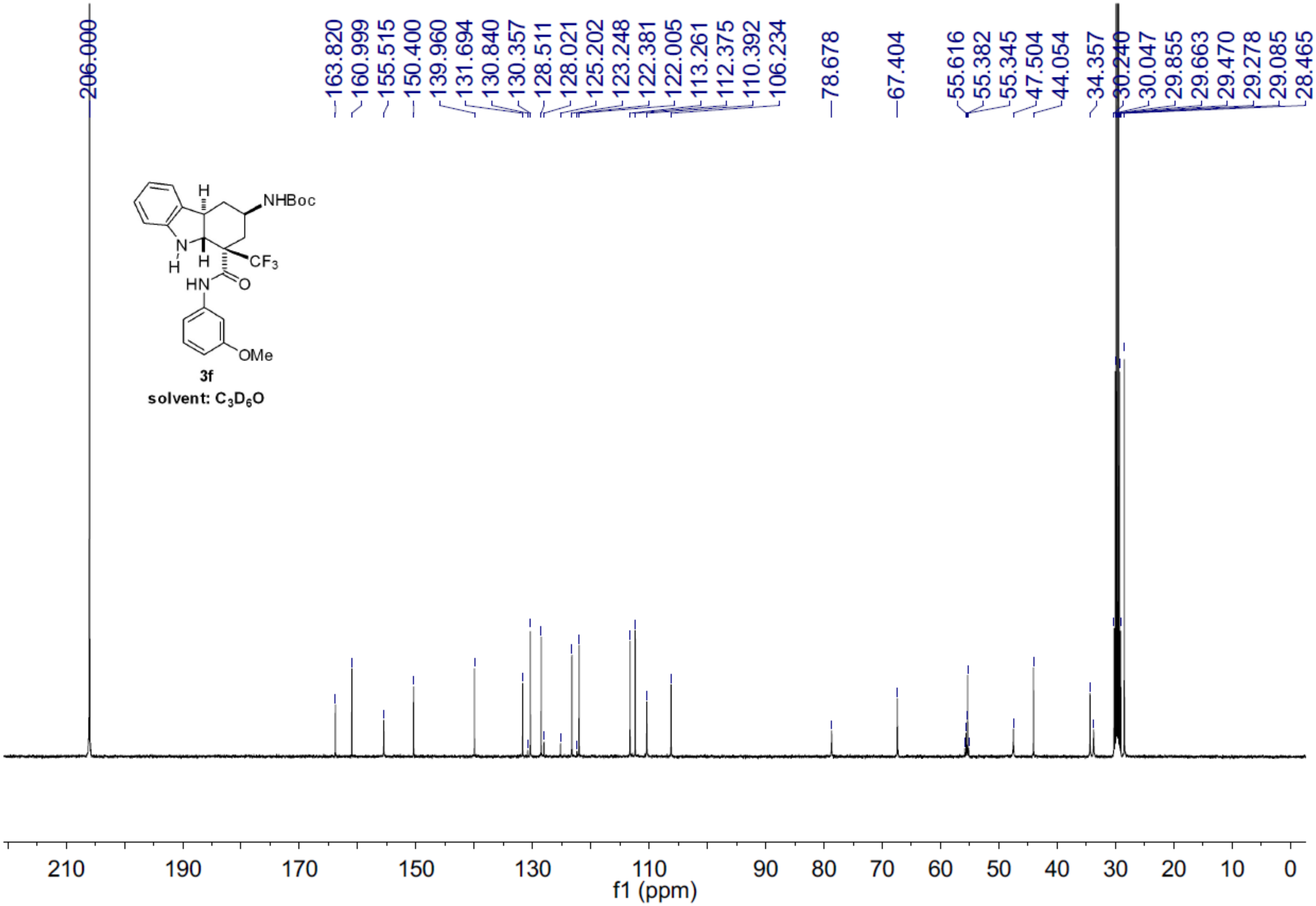

Supplementary Figure 41. ^{13}C NMR spectrum for compound **3f**

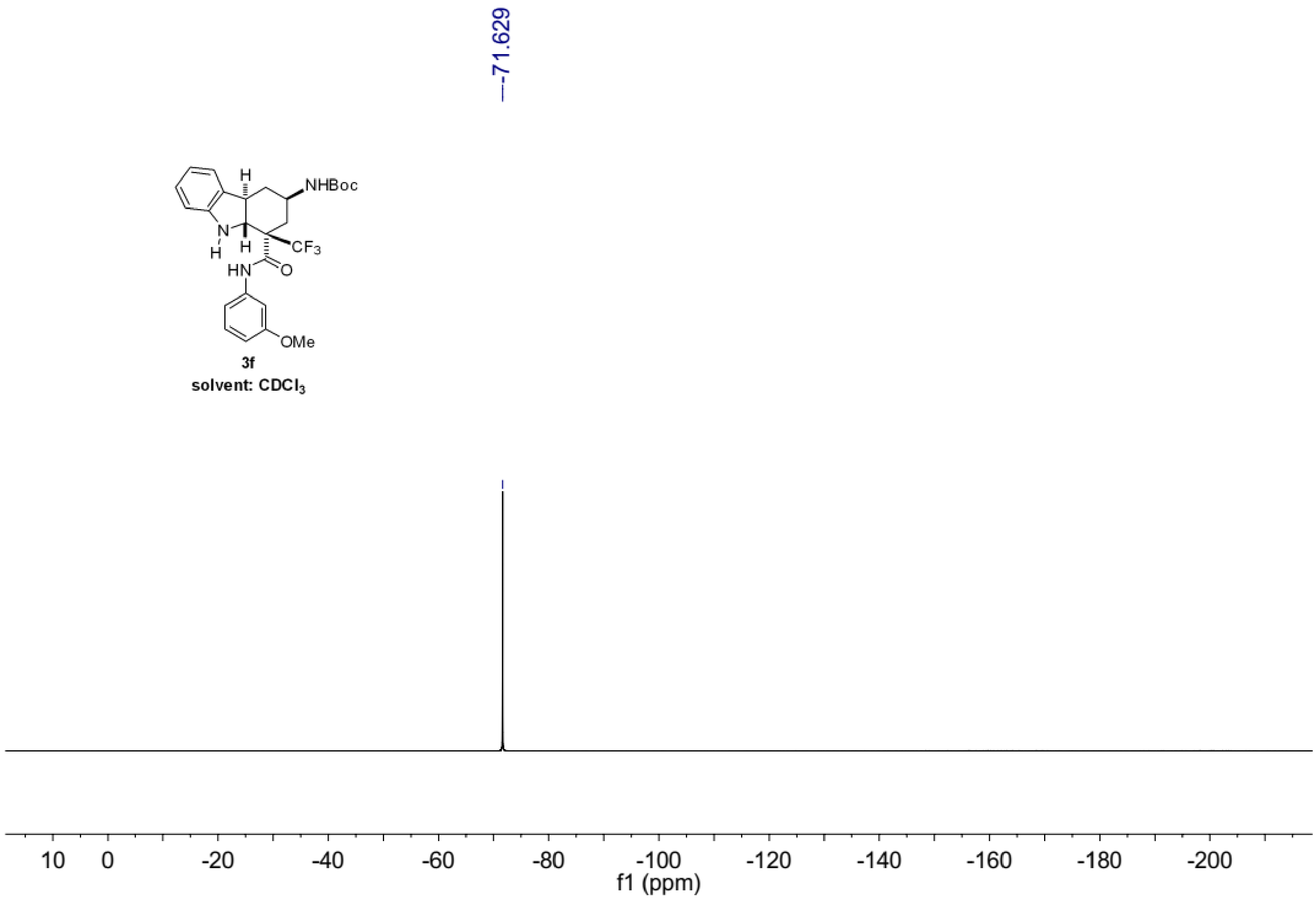

Supplementary Figure 42. ^{19}F NMR spectrum for compound **3f**

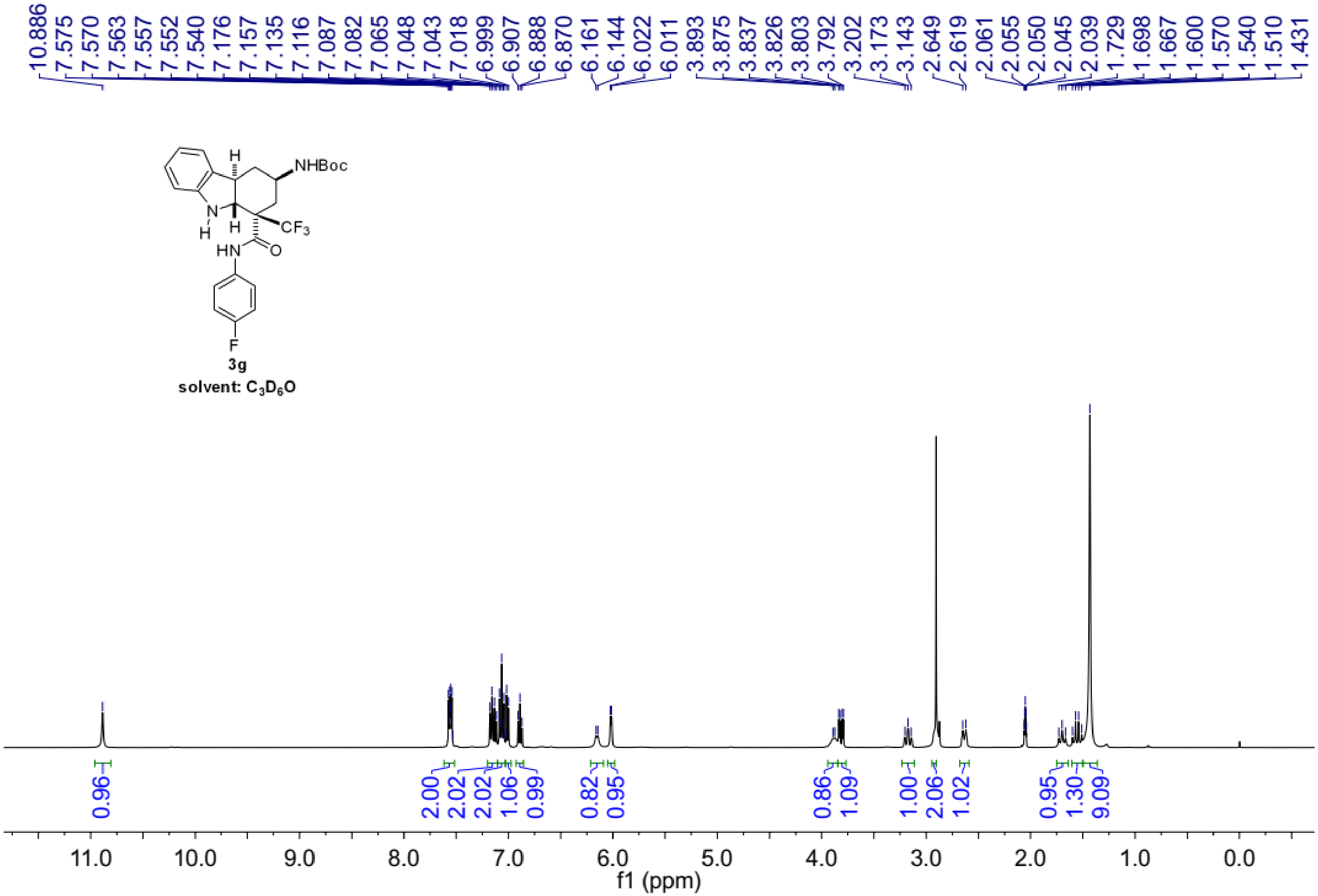

Supplementary Figure 43. ^{1}H NMR spectrum for compound **3g**

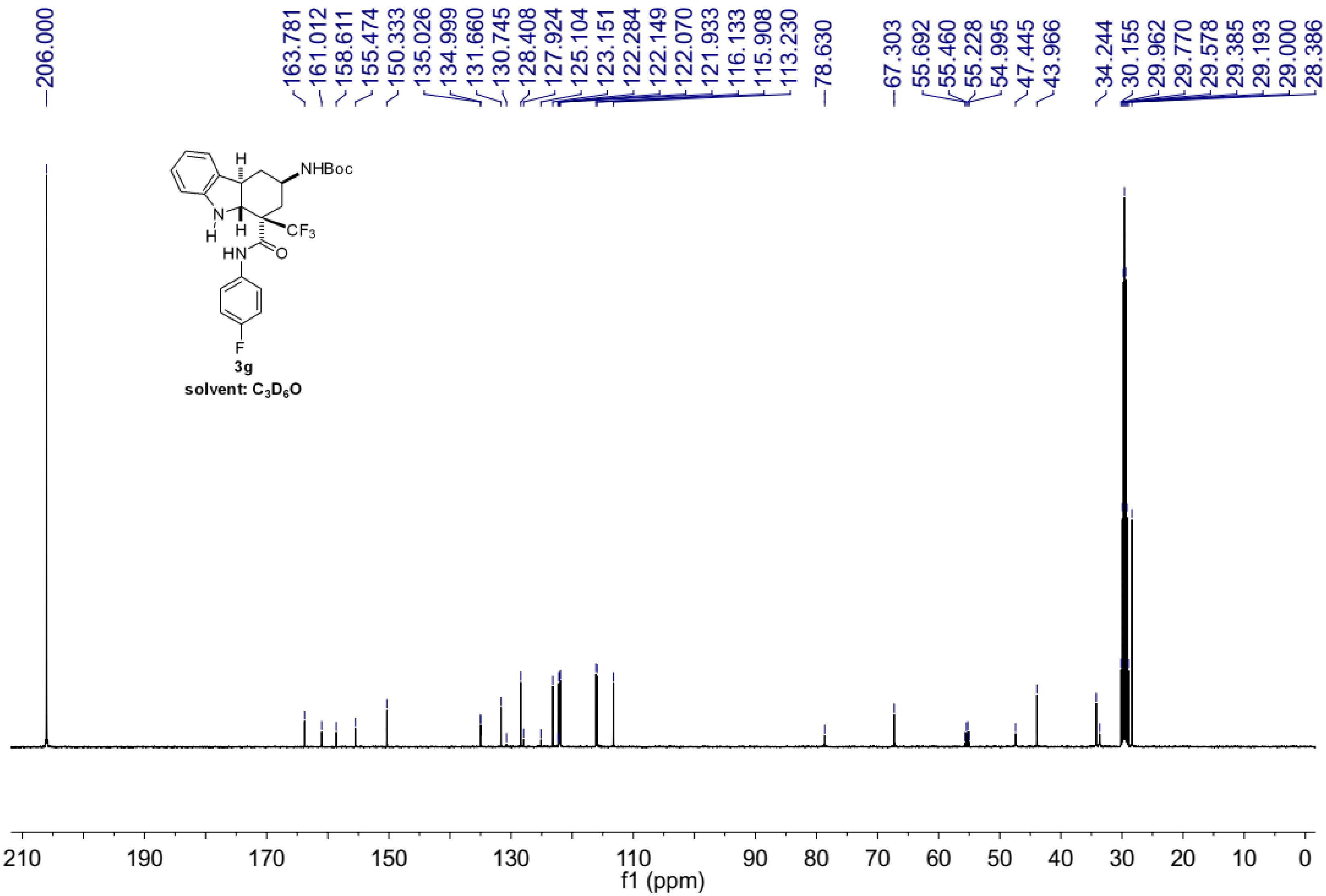

Supplementary Figure 44. ^{13}C NMR spectrum for compound **3g**

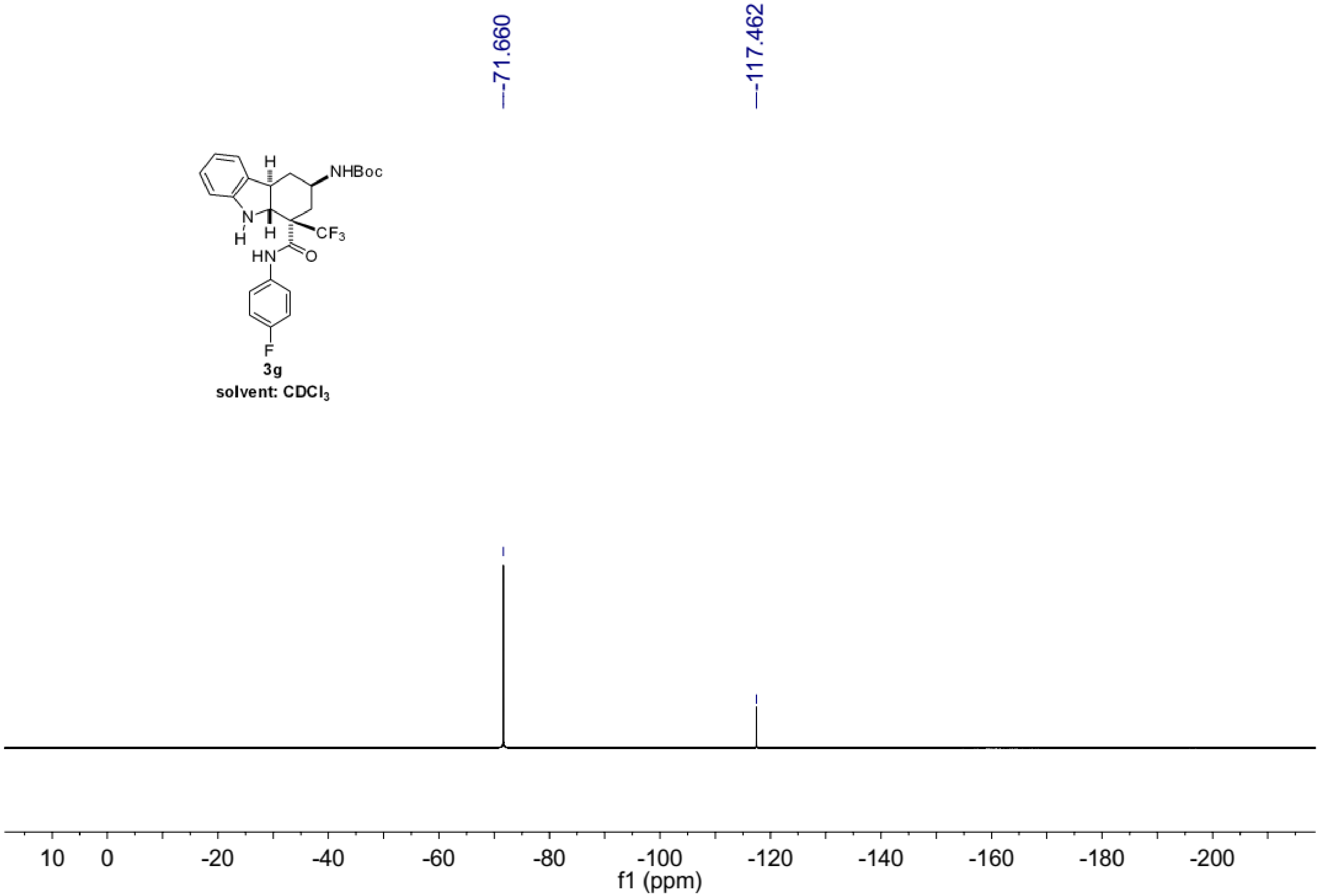

3g

solvent: $CDCl_3$

Supplementary Figure 45. ^{19}F NMR spectrum for compound **3g**

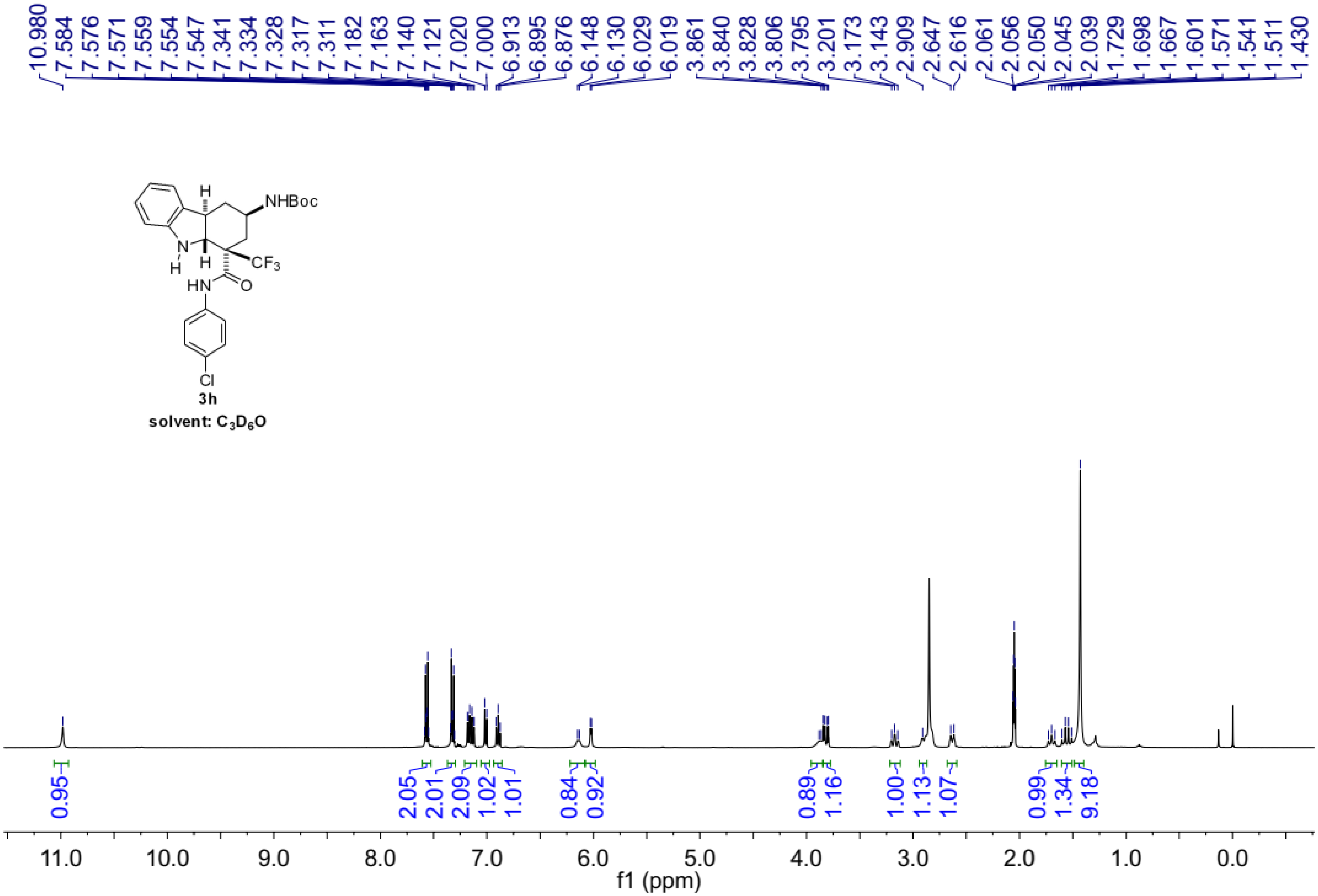

Supplementary Figure 46. ^{1}H NMR spectrum for compound **3h**

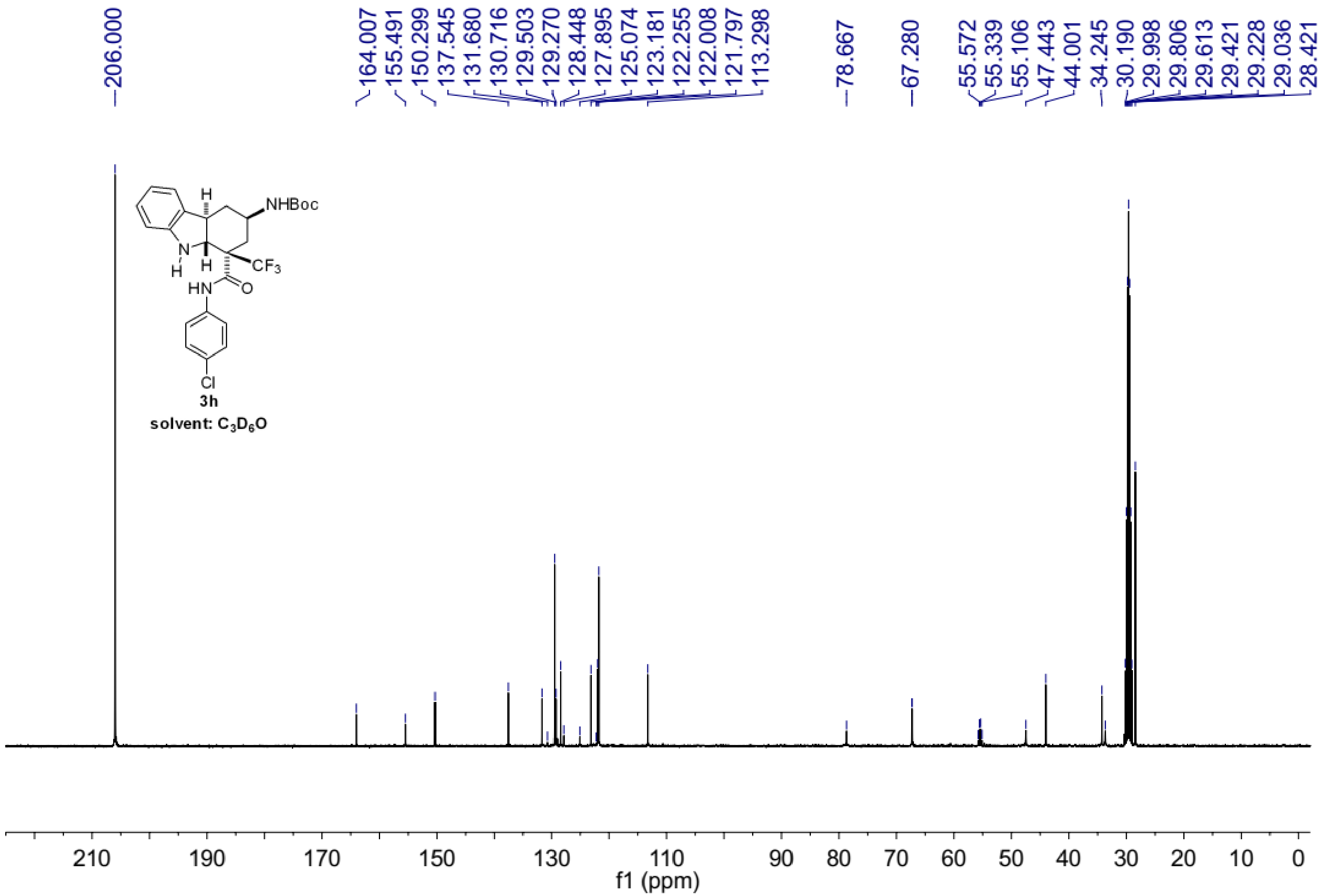

Supplementary Figure 47. ^{13}C NMR spectrum for compound **3h**

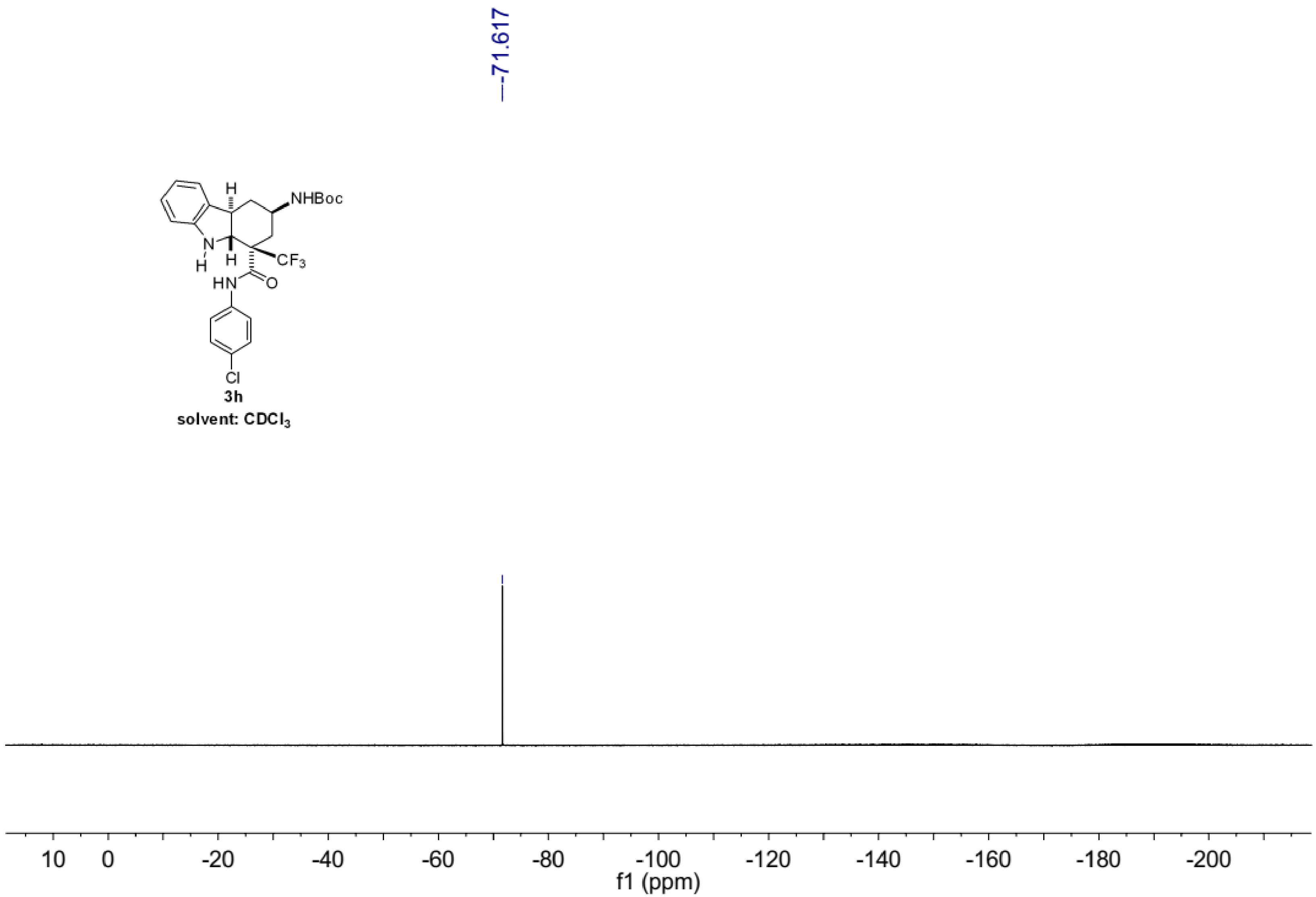

Supplementary Figure 48. ^{19}F NMR spectrum for compound **3h**

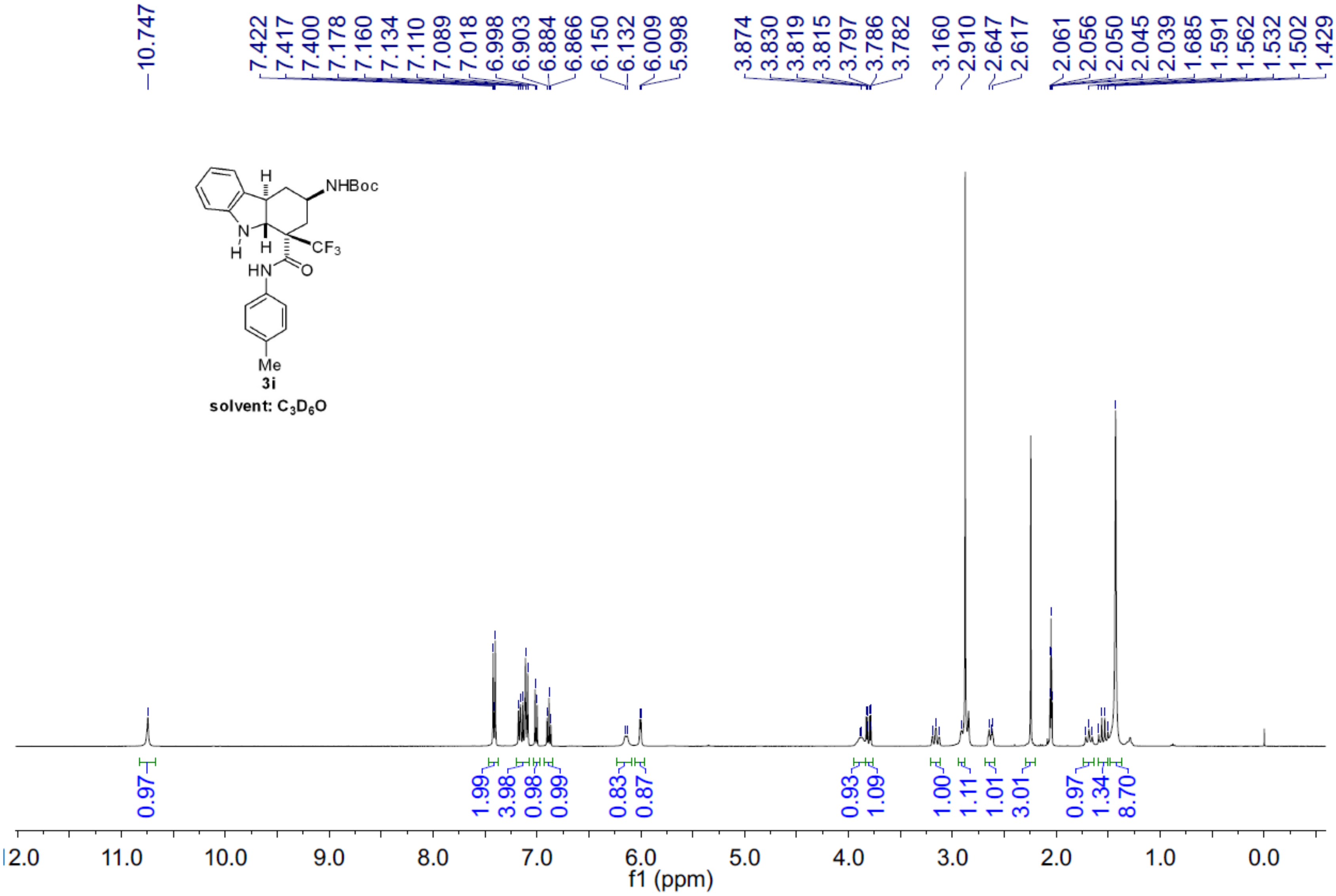

Supplementary Figure 49. ^{1}H NMR spectrum for compound **3i**

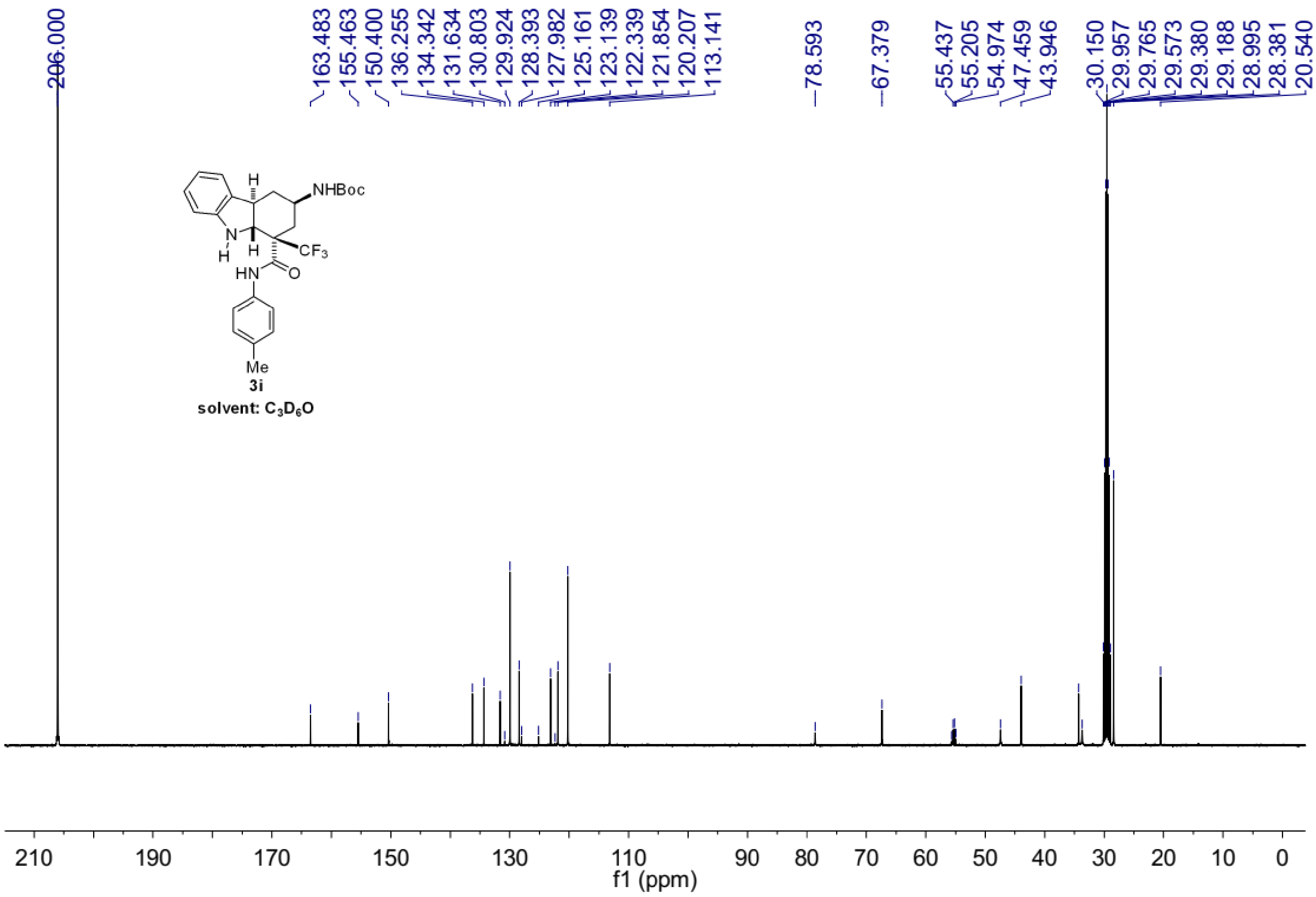

Supplementary Figure 50. ^{13}C NMR spectrum for compound **3i**

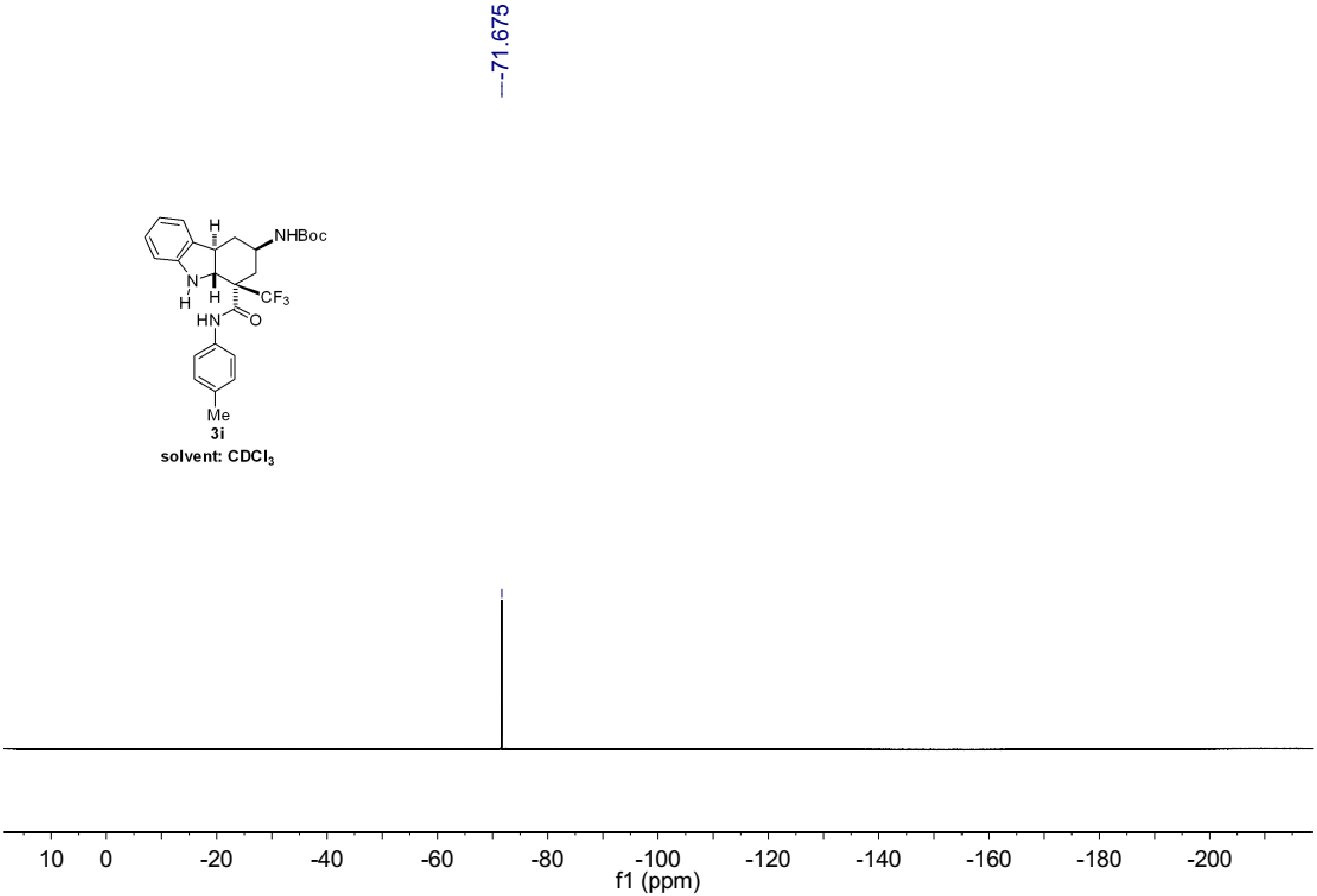

Supplementary Figure 51. ^{19}F NMR spectrum for compound **3i**

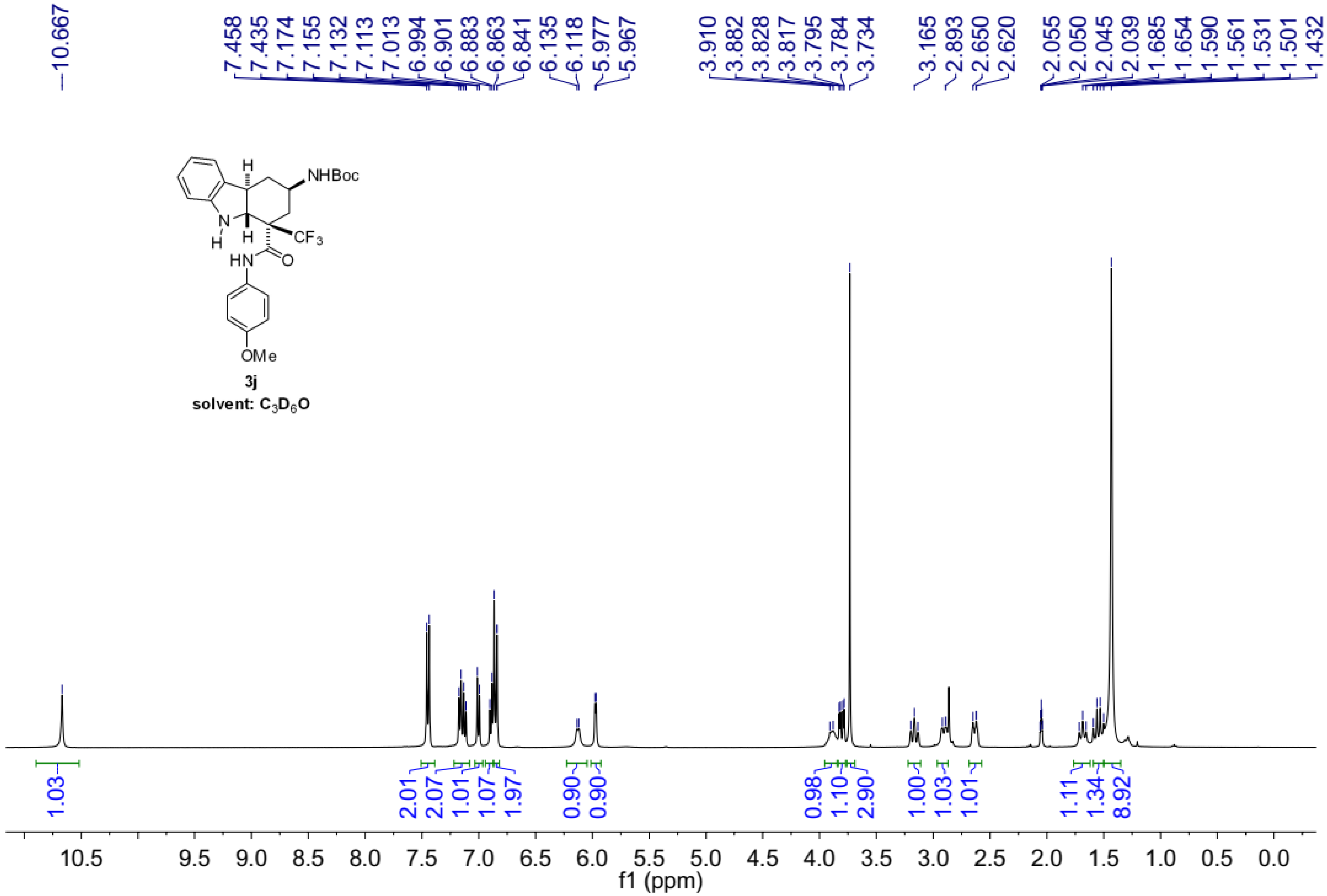

Supplementary Figure 52. ^{1}H NMR spectrum for compound **3j**

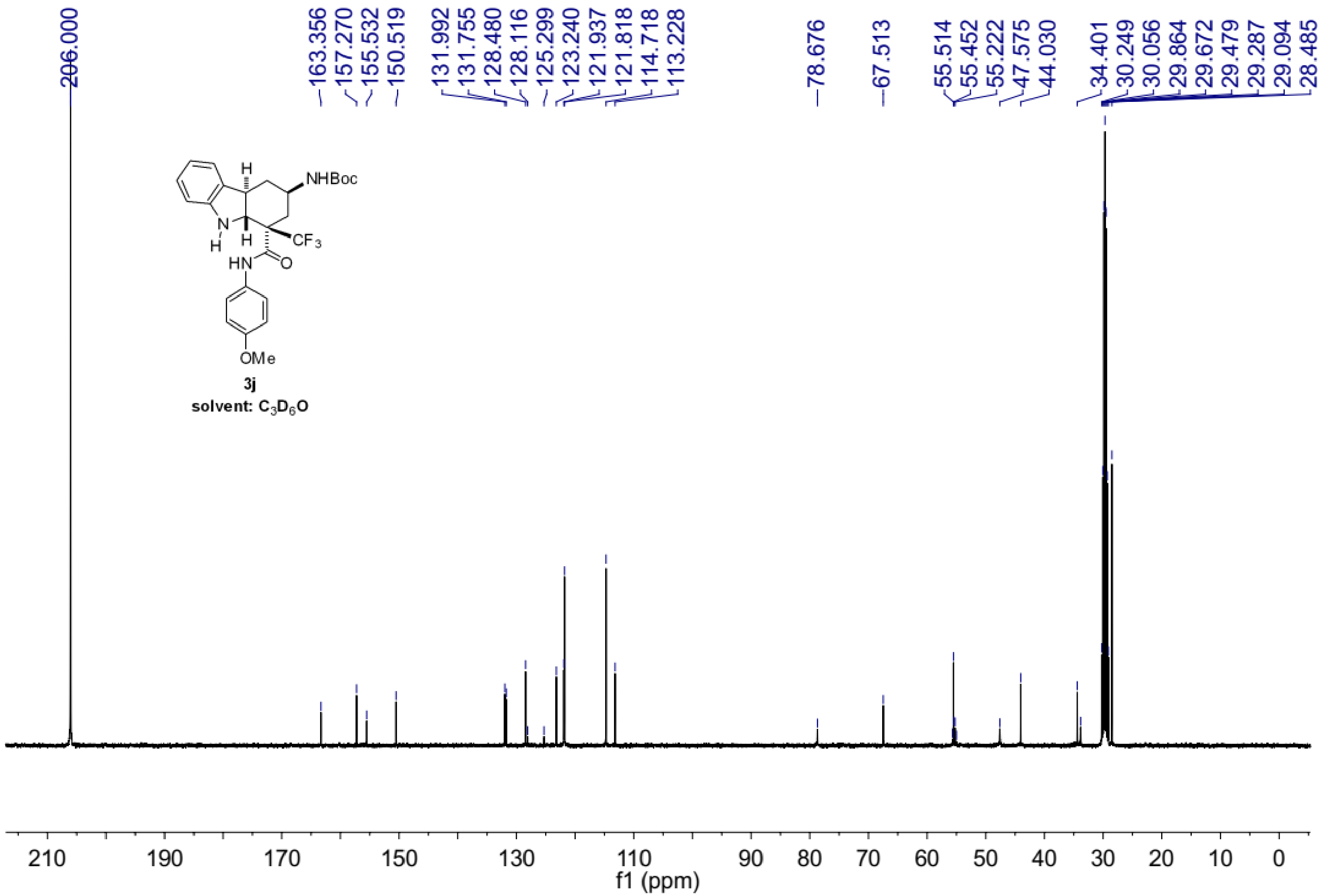

Supplementary Figure 53. ^{13}C NMR spectrum for compound **3j**

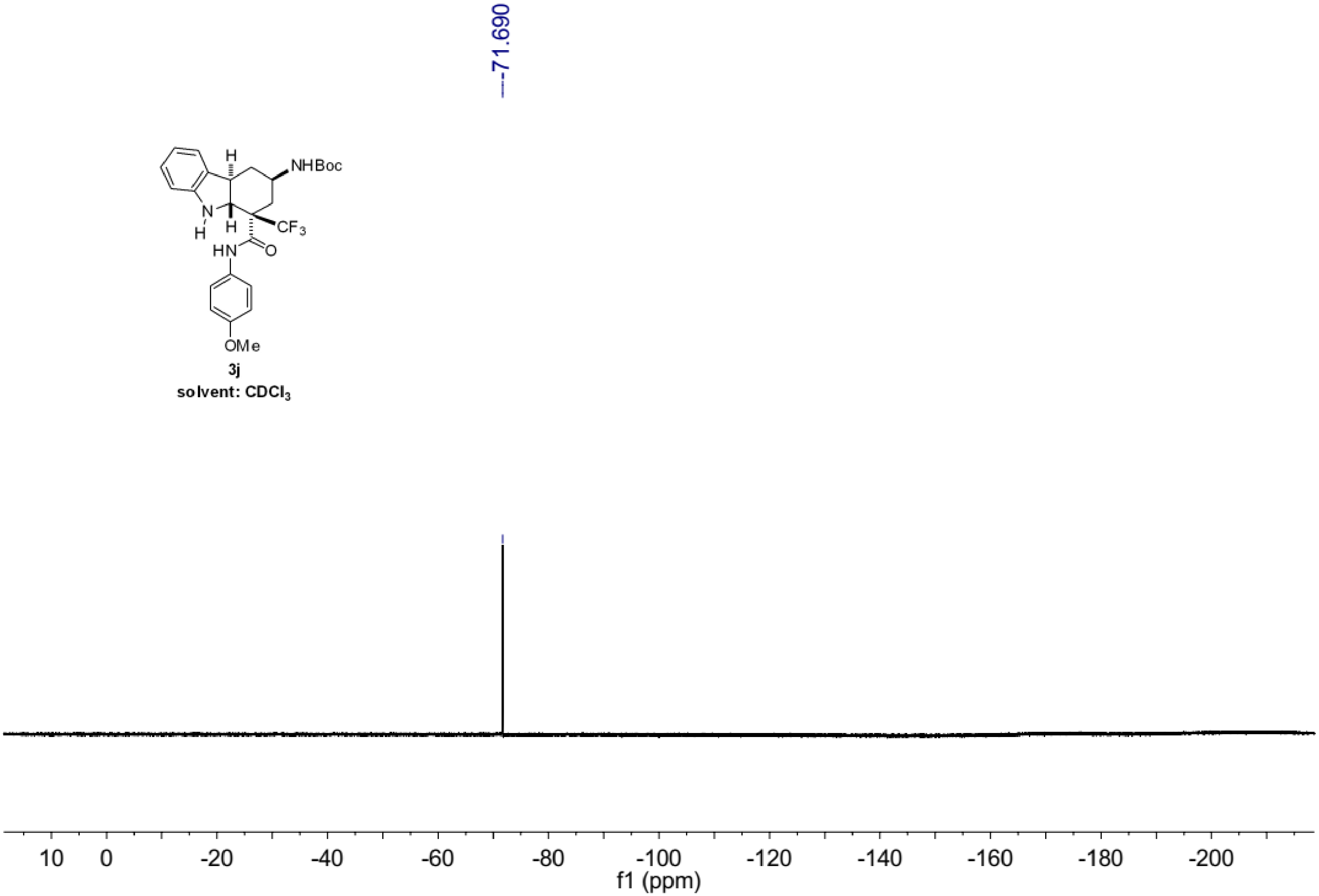

Supplementary Figure 54. ^{19}F NMR spectrum for compound **3j**

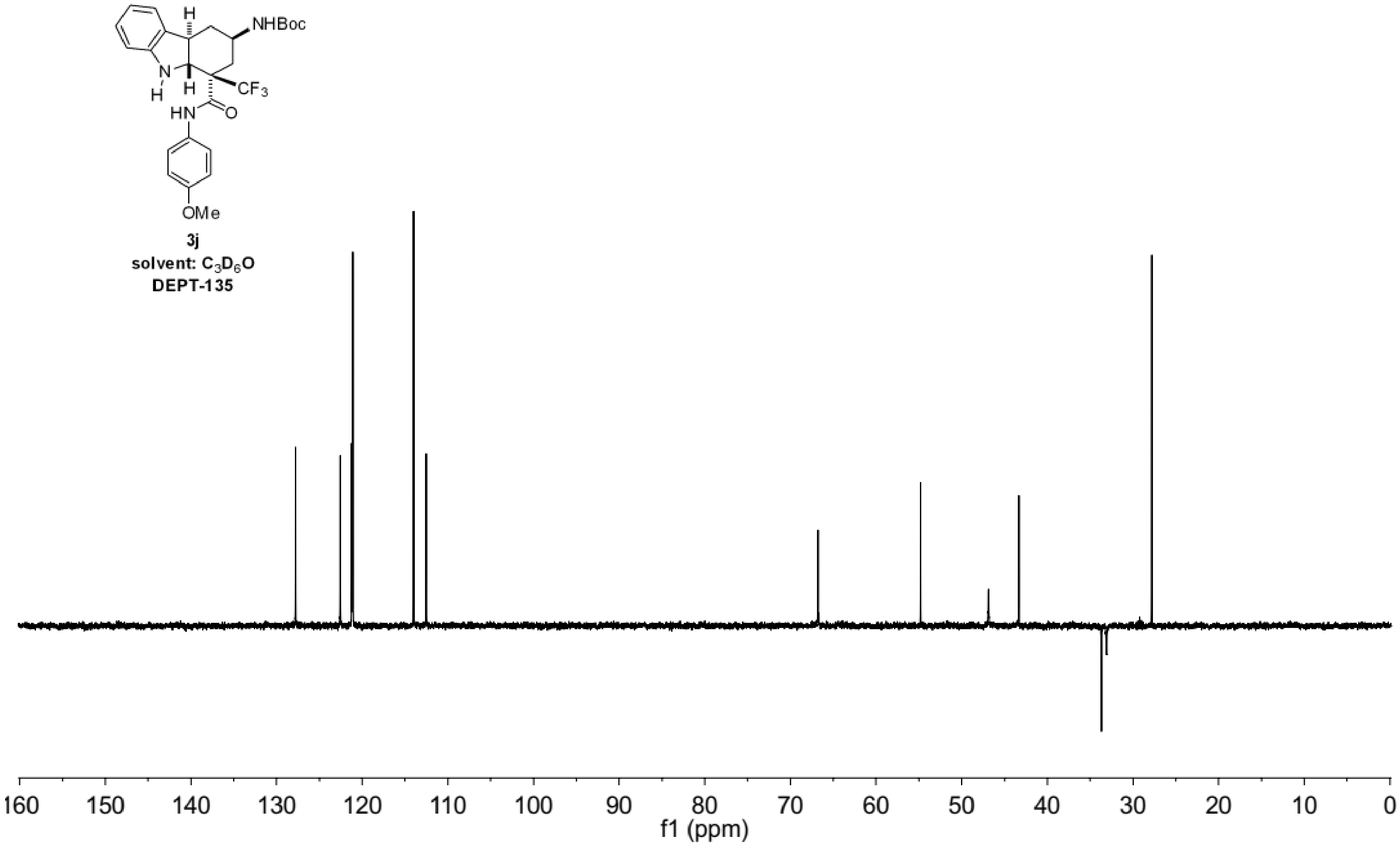

Supplementary Figure 55. DEPT-135 spectrum for compound **3j**

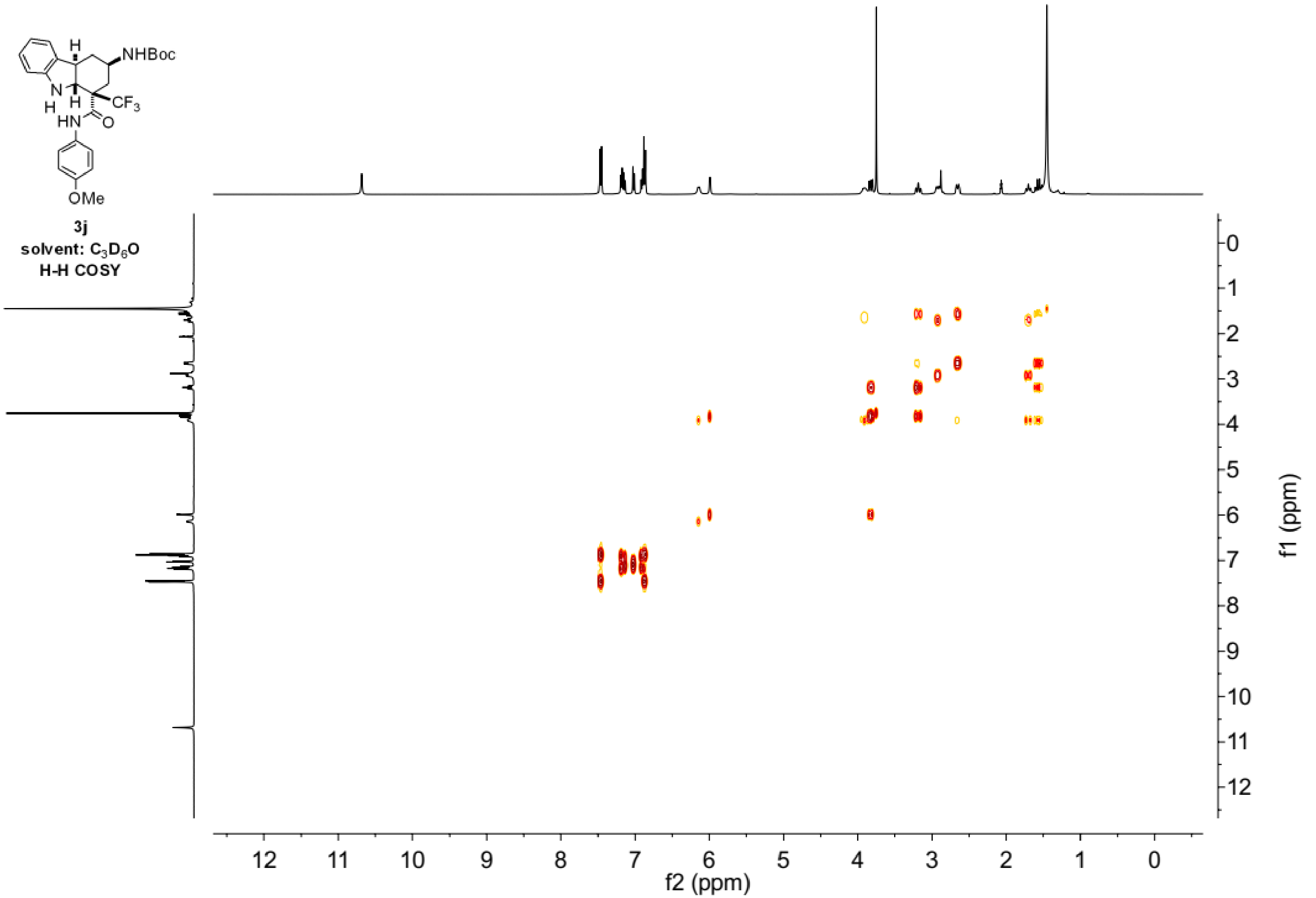

Supplementary Figure 56. H-H COSY spectrum for compound **3j**

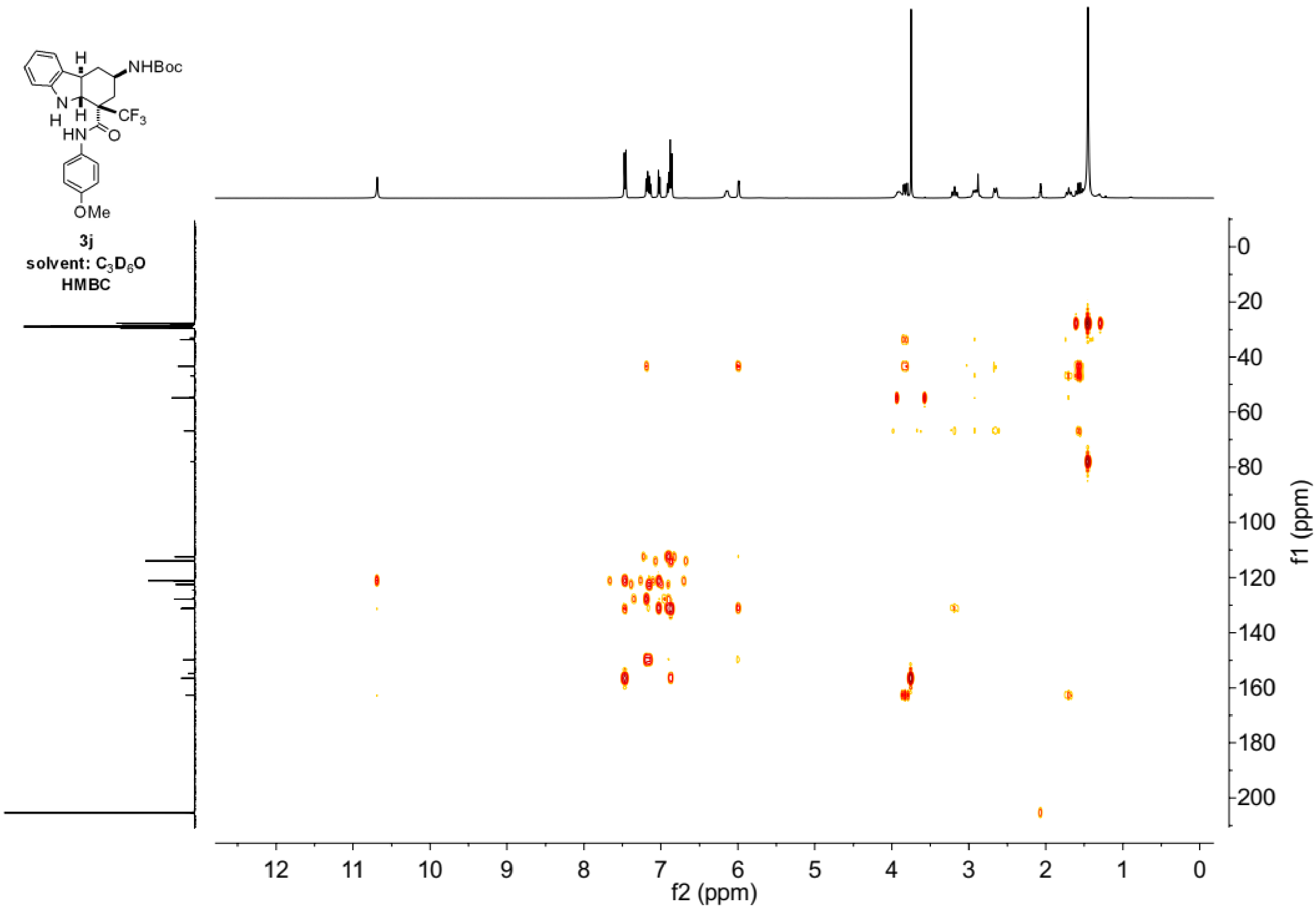

Supplementary Figure 57. HMBC spectrum for compound **3j**

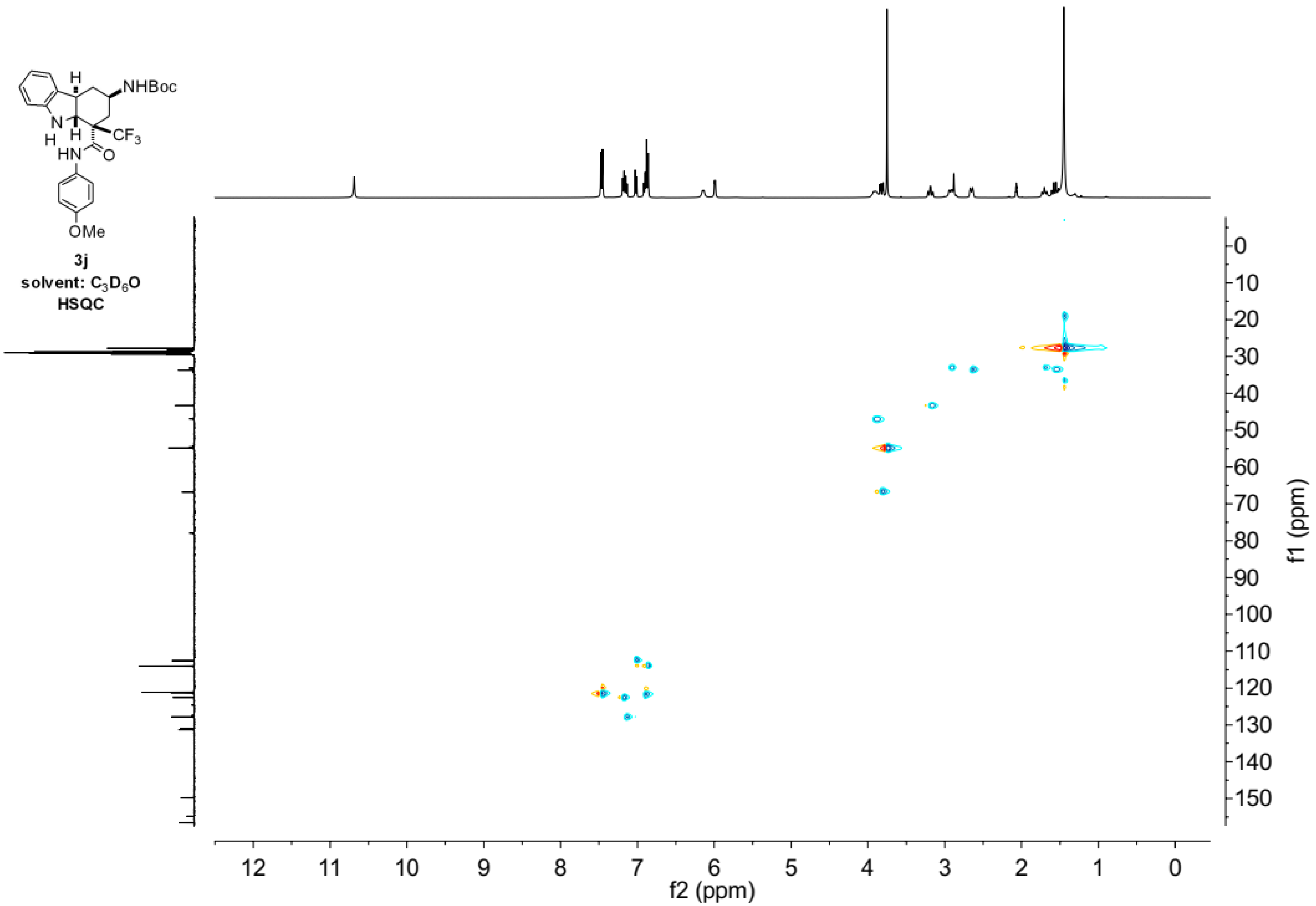

Supplementary Figure 58. HSQC spectrum for compound **3j**

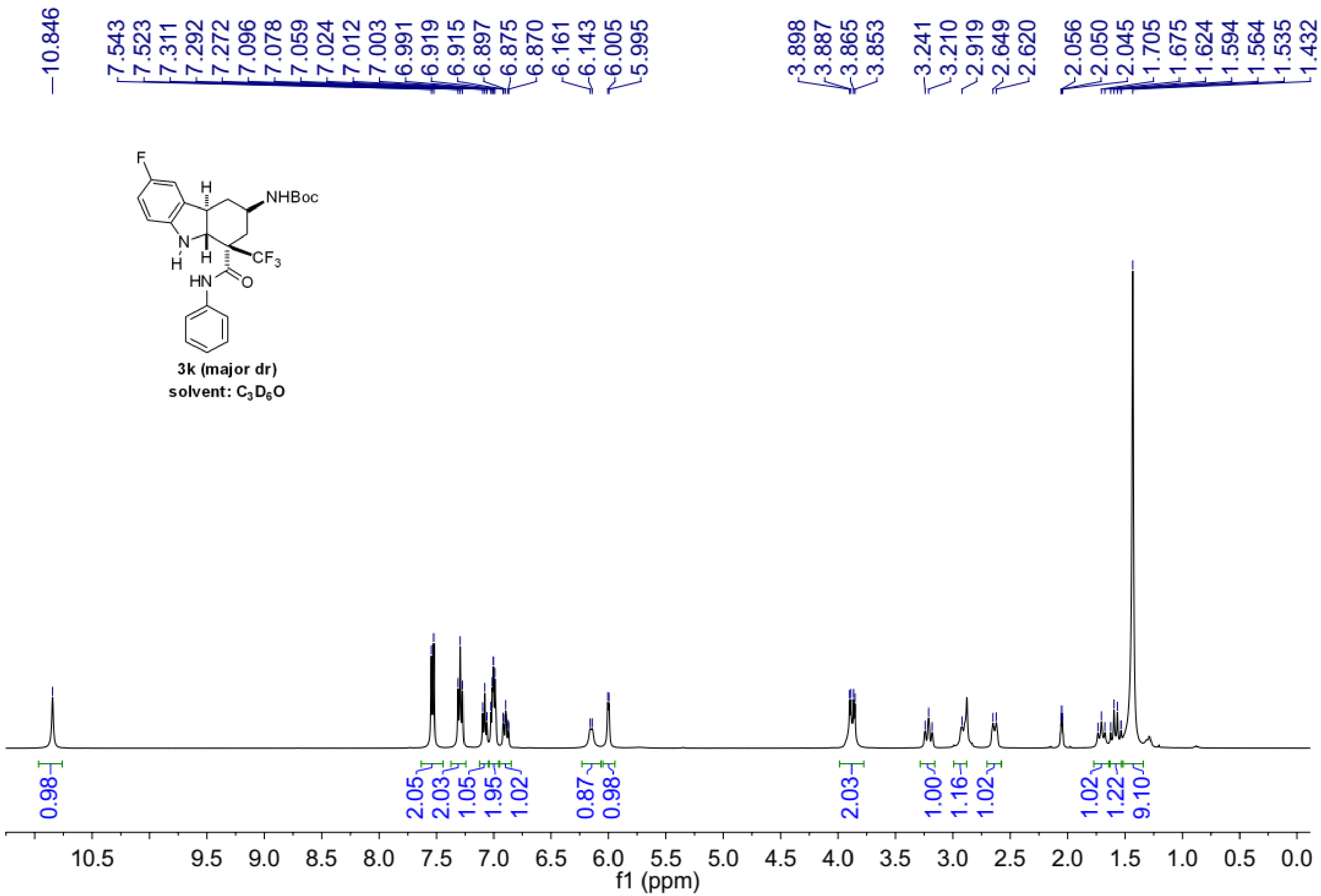

Supplementary Figure 59. ^{1}H NMR spectrum for compound **3k**

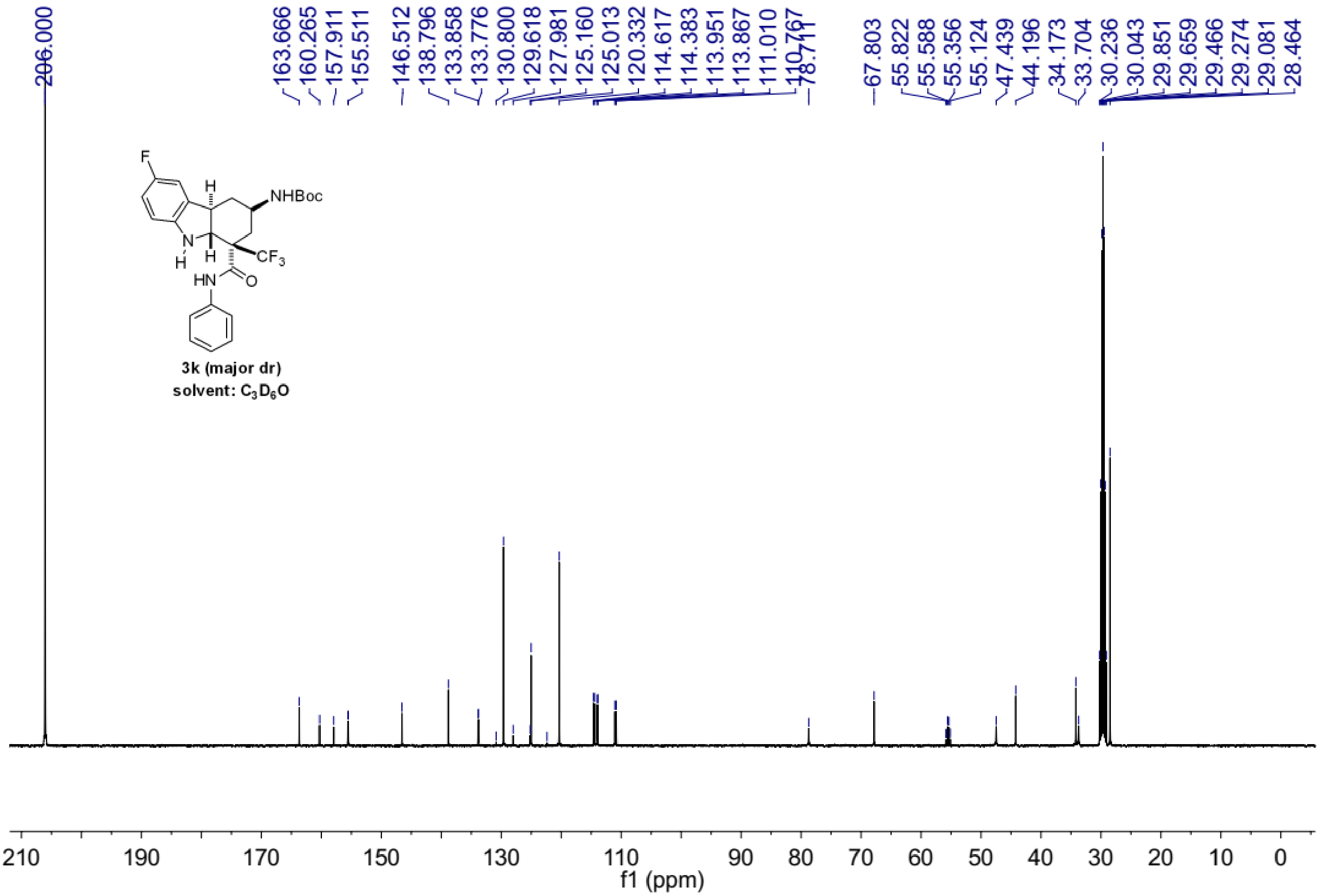

Supplementary Figure 60. ^{13}C NMR spectrum for compound **3k**

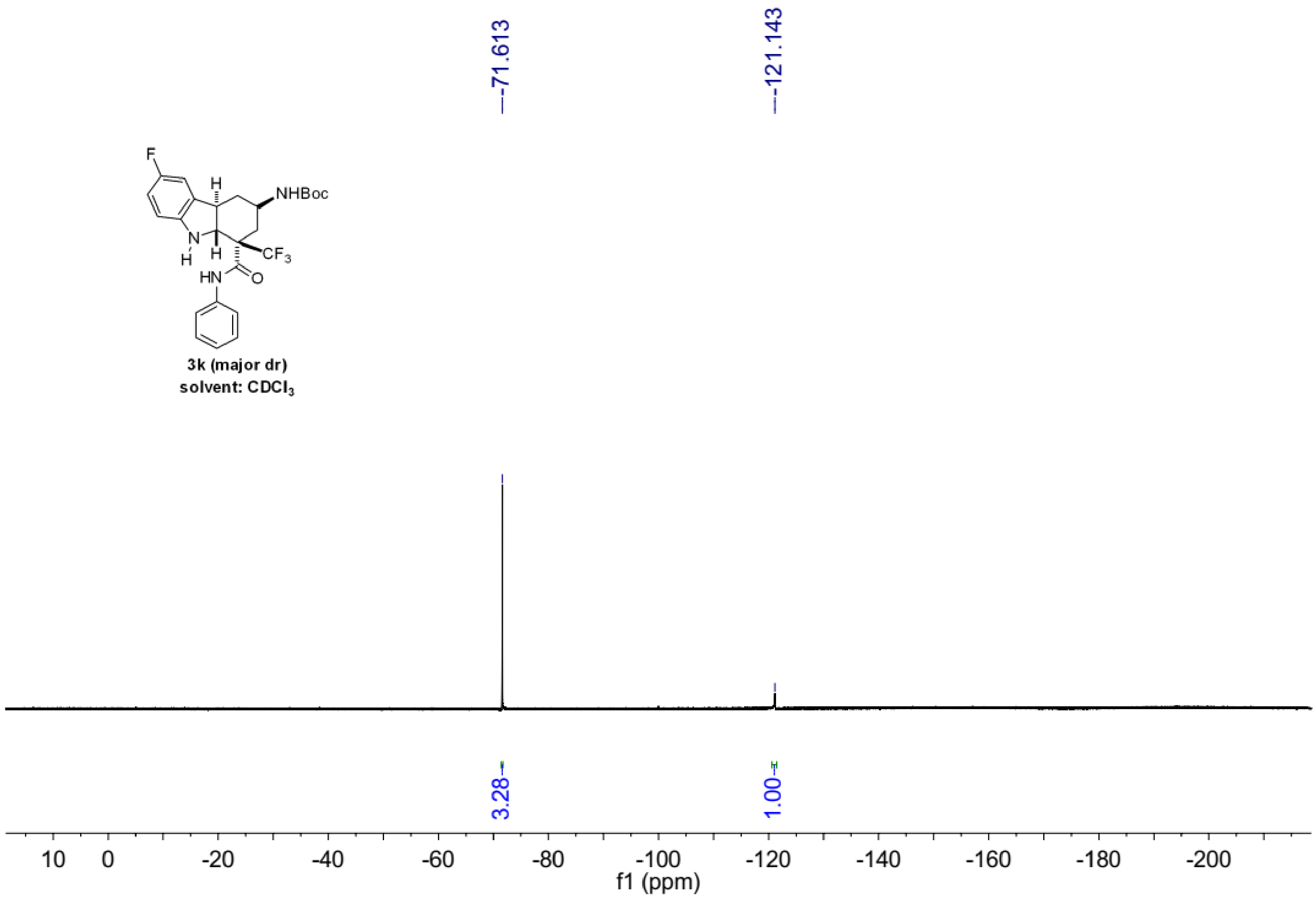

Supplementary Figure 61. ^{19}F NMR spectrum for compound **3k**

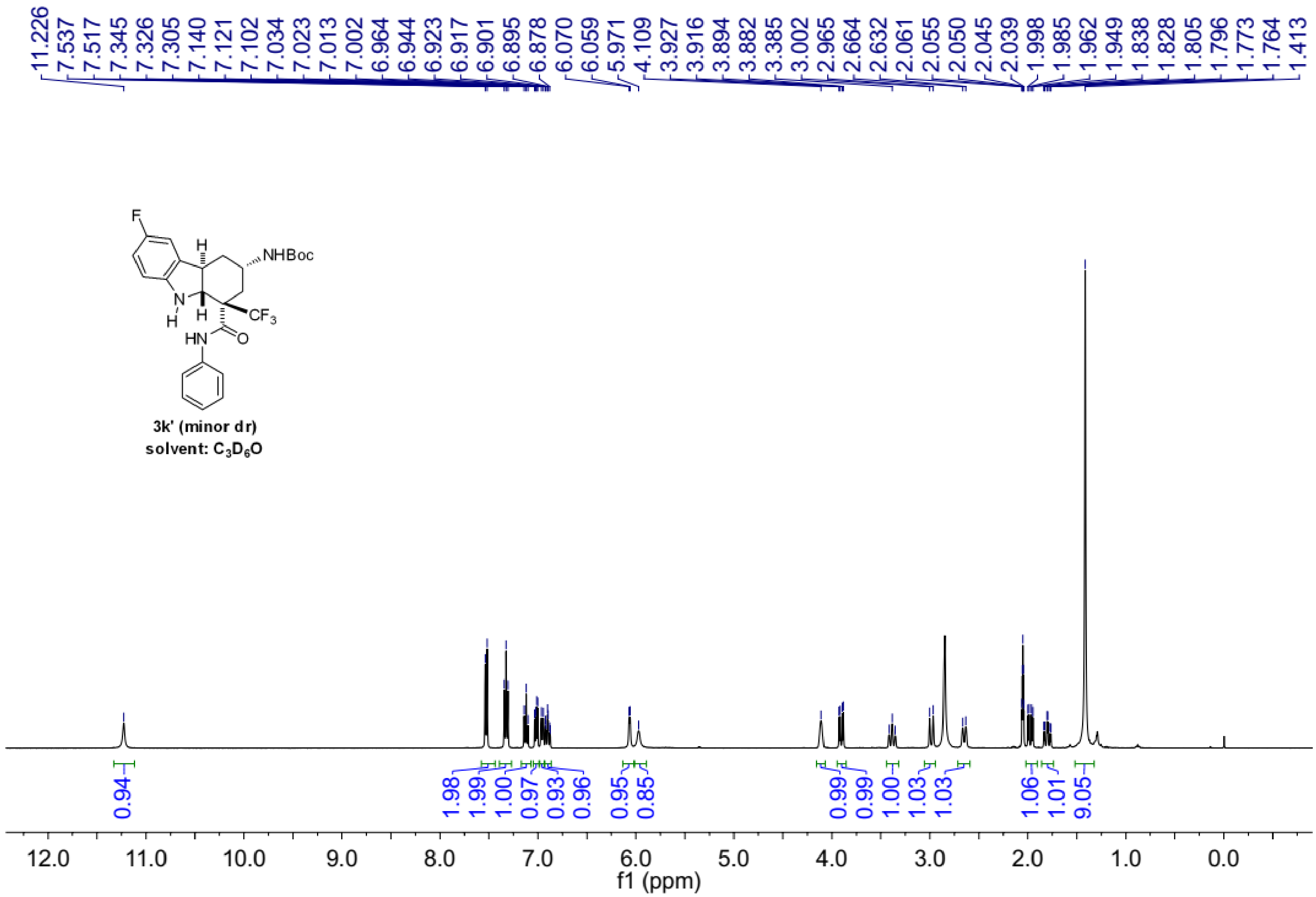

Supplementary Figure 62. ^{1}H NMR spectrum for compound **3k'**

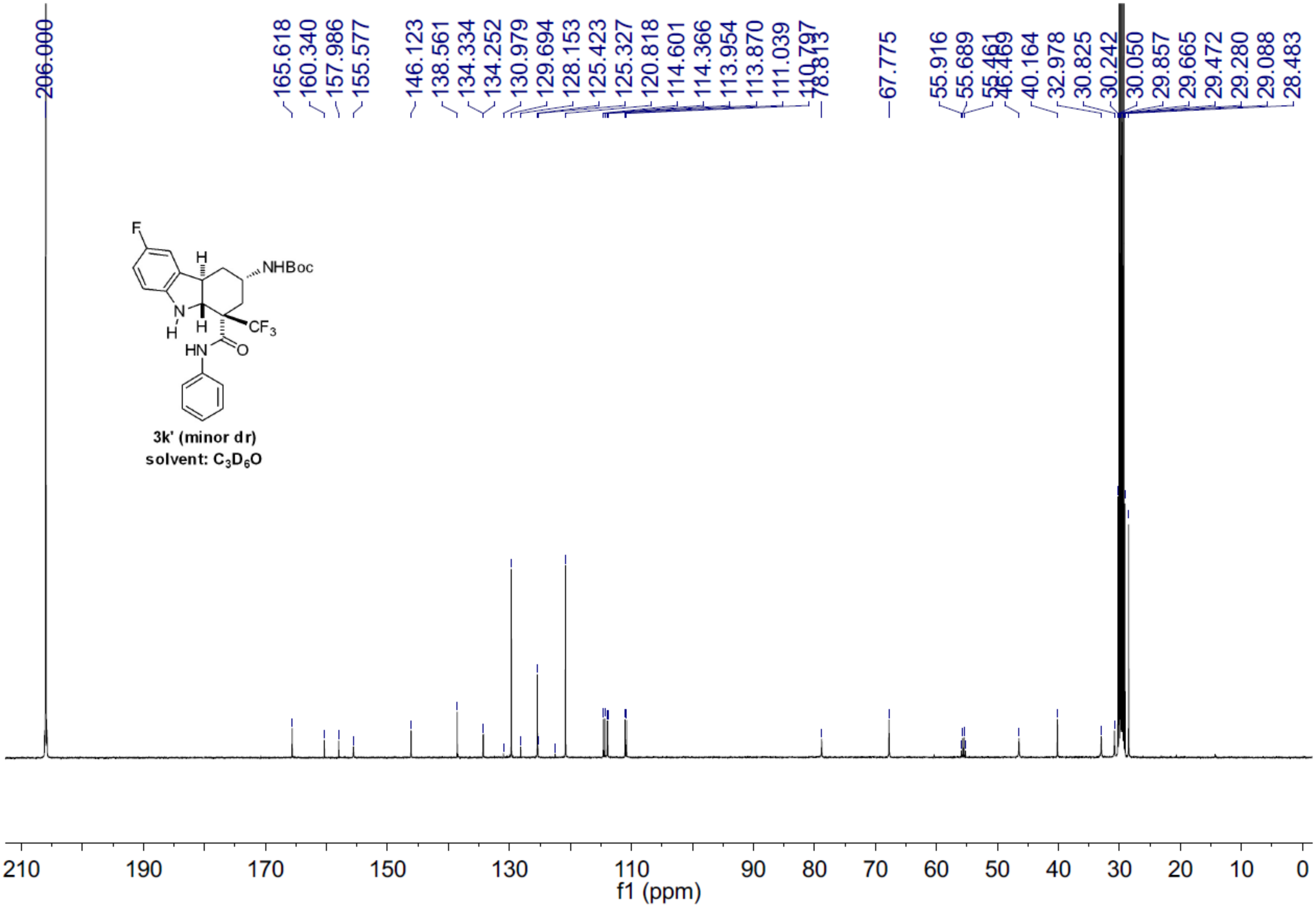

Supplementary Figure 63. ^{13}C NMR spectrum for compound **3k'**

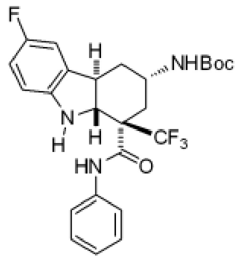

3k' (minor dr)
solvent: $CDCl_3$

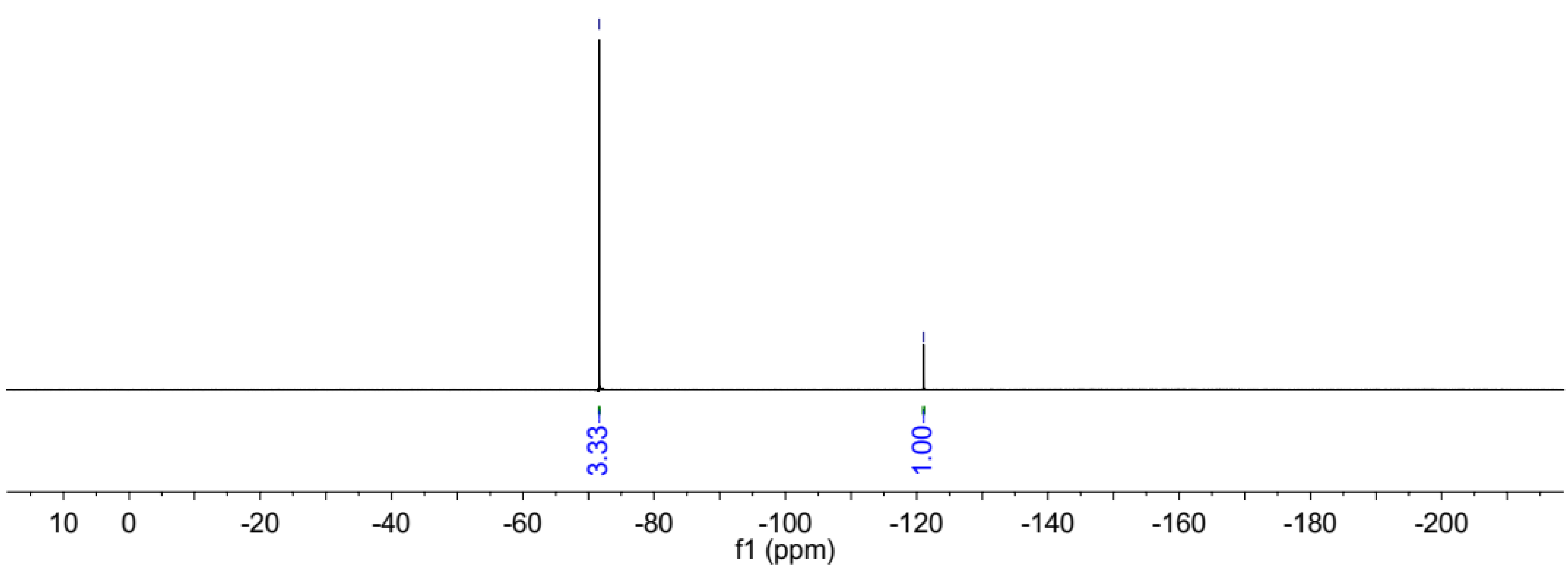

Supplementary Figure 64. ^{19}F NMR spectrum for compound **3k'**

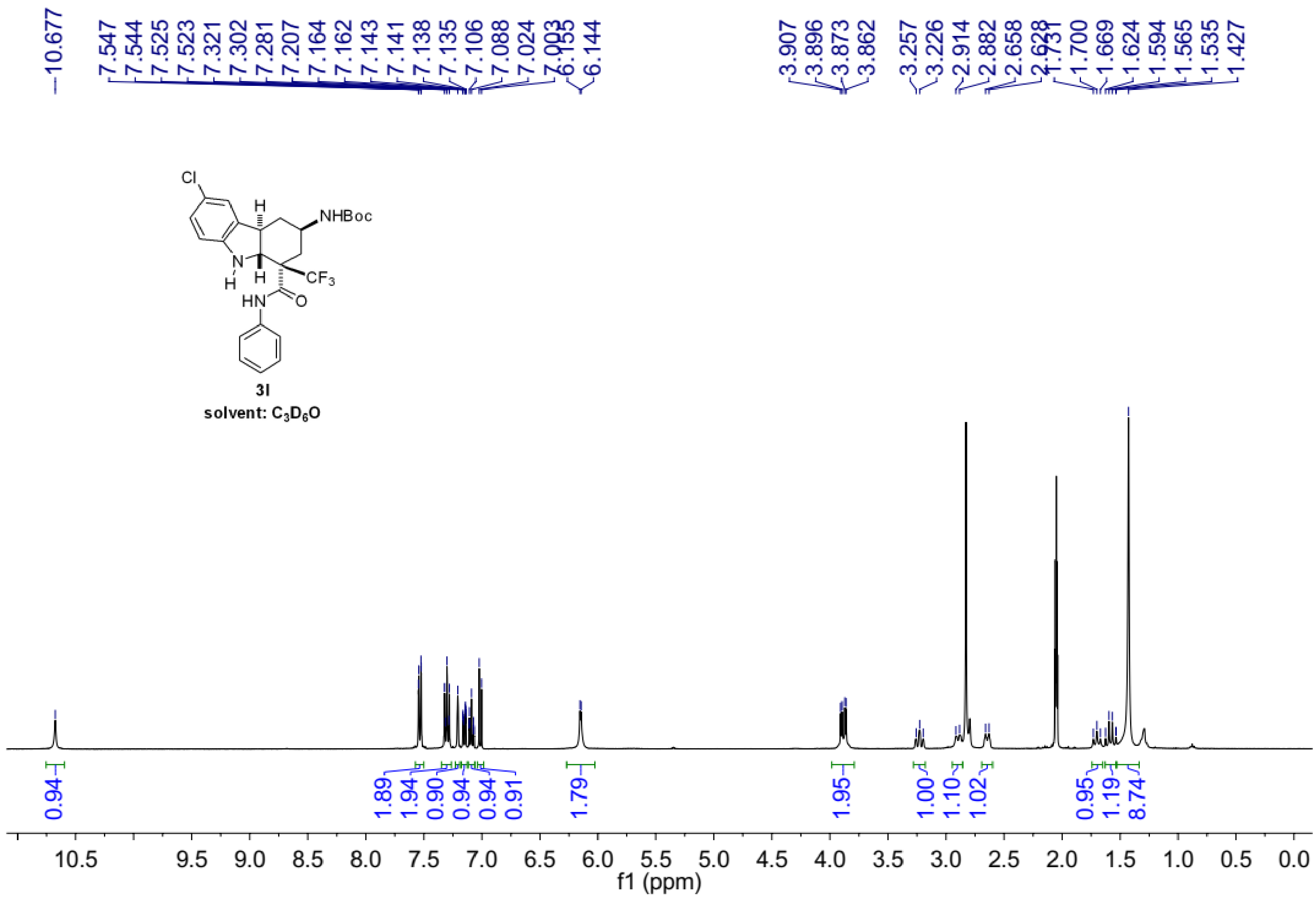

Supplementary Figure 65. ^{1}H NMR spectrum for compound **3l**

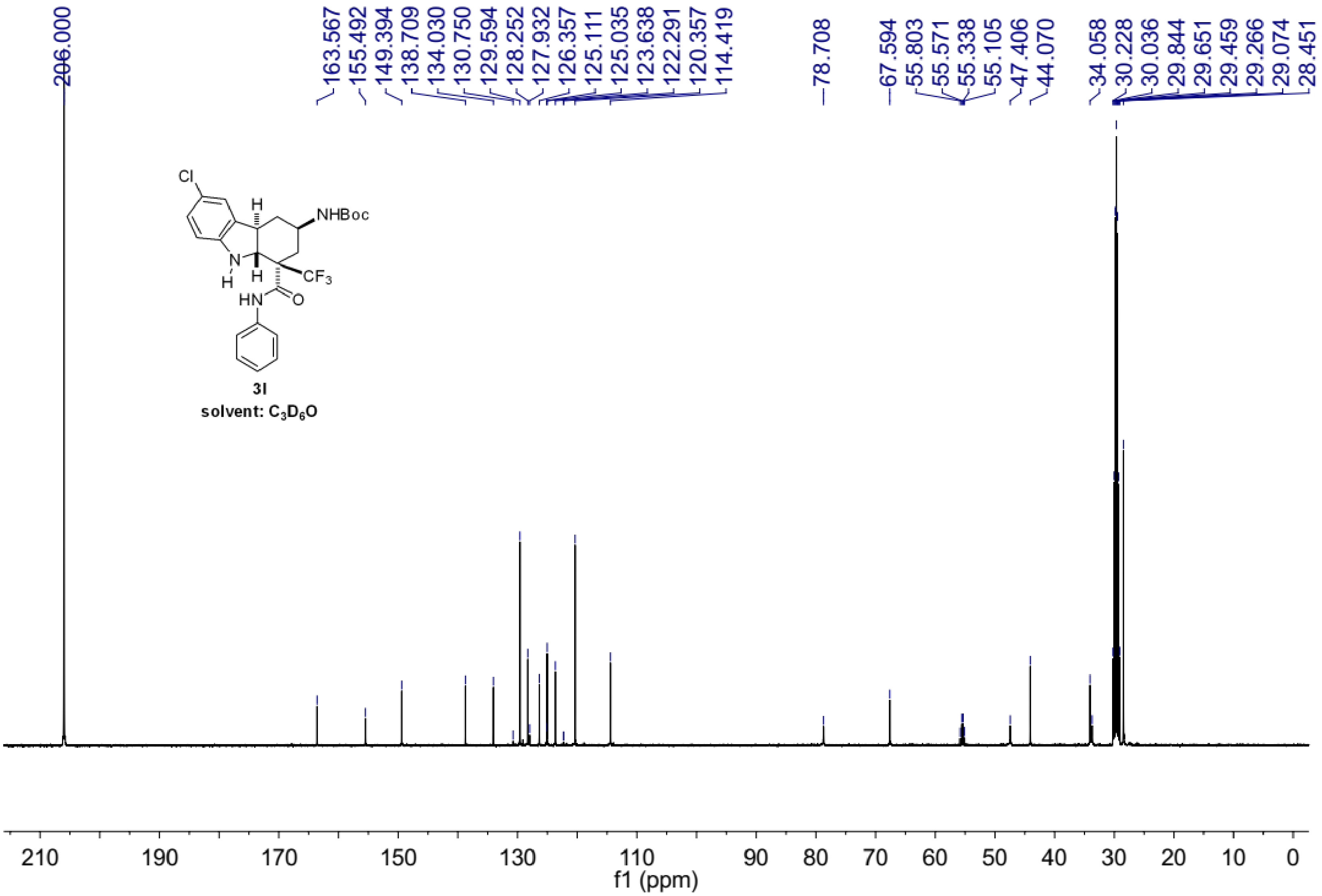

Supplementary Figure 66. ^{13}C NMR spectrum for compound **3l**

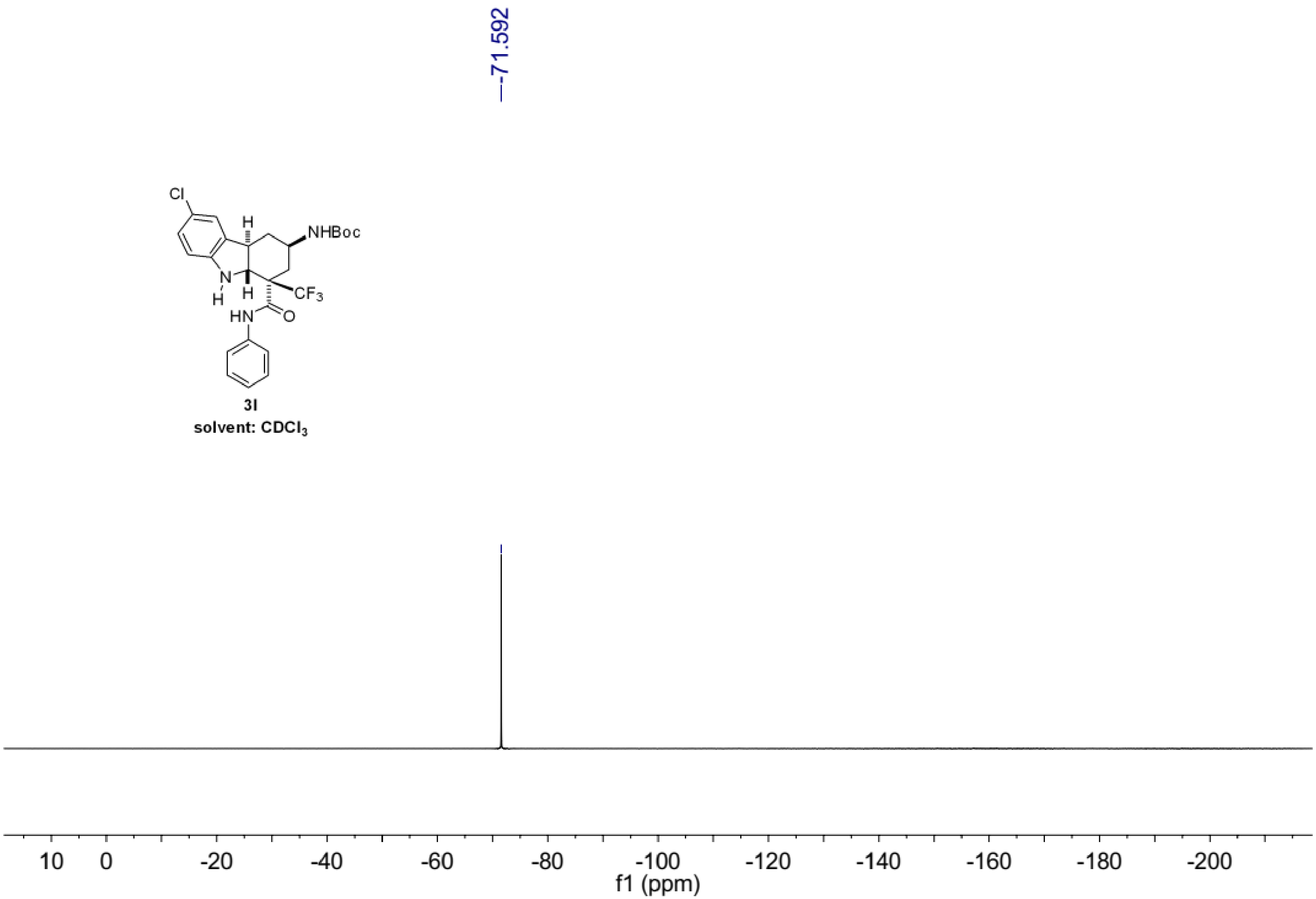

Supplementary Figure 67. ^{19}F NMR spectrum for compound **3l**

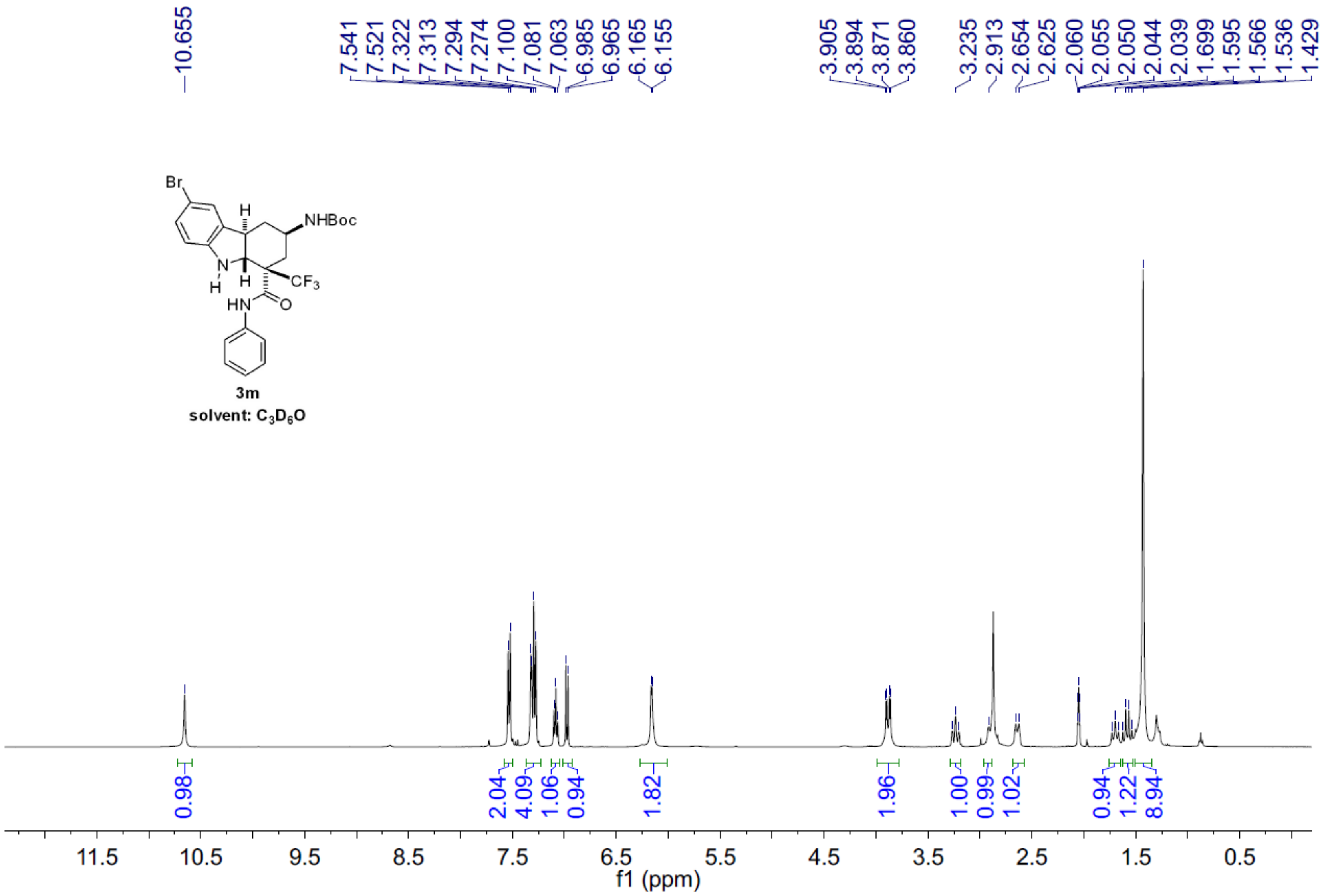

Supplementary Figure 68. ^{1}H NMR spectrum for compound **3m**

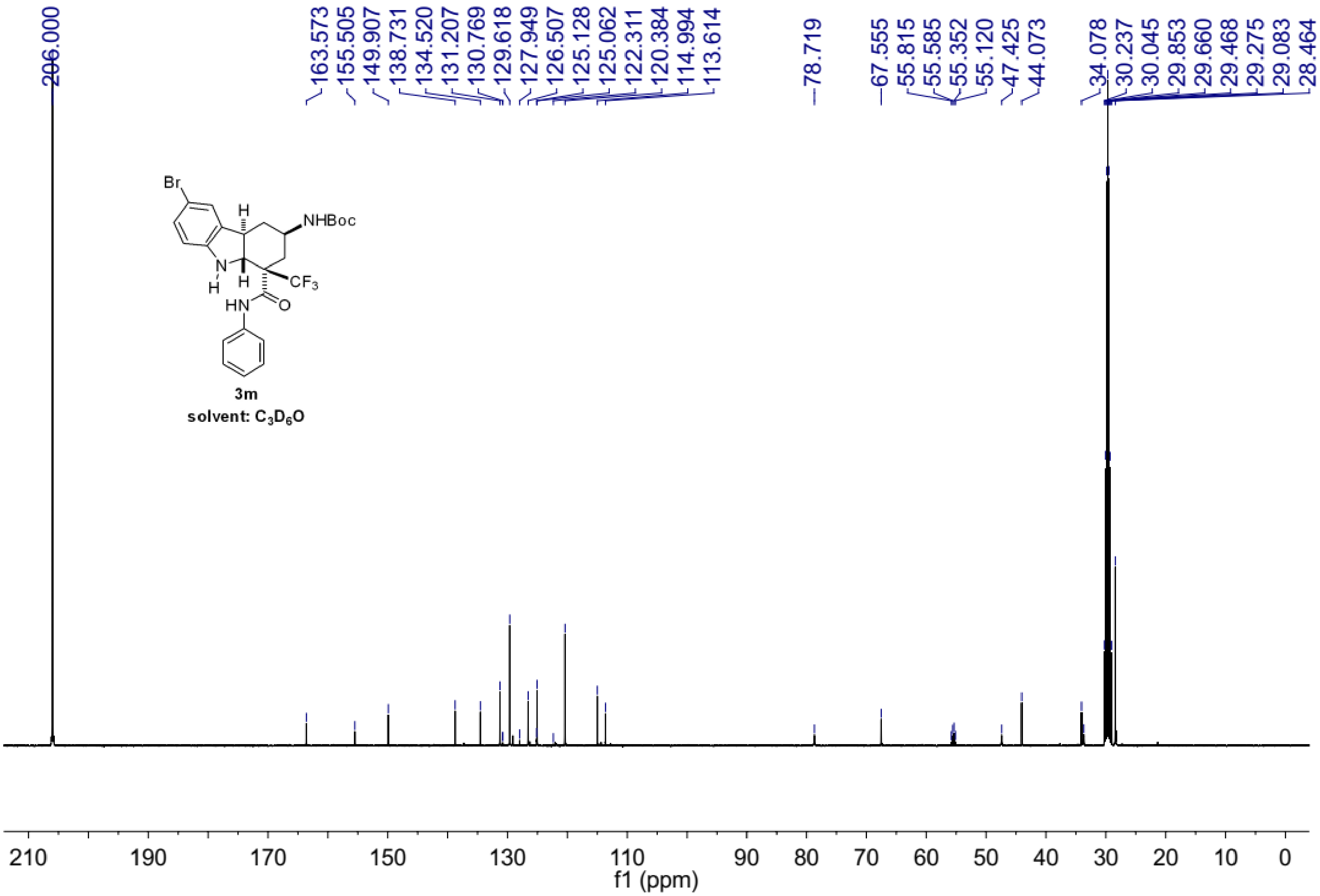

Supplementary Figure 69. ^{13}C NMR spectrum for compound **3m**

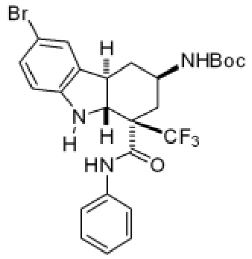

3m
solvent: $CDCl_3$

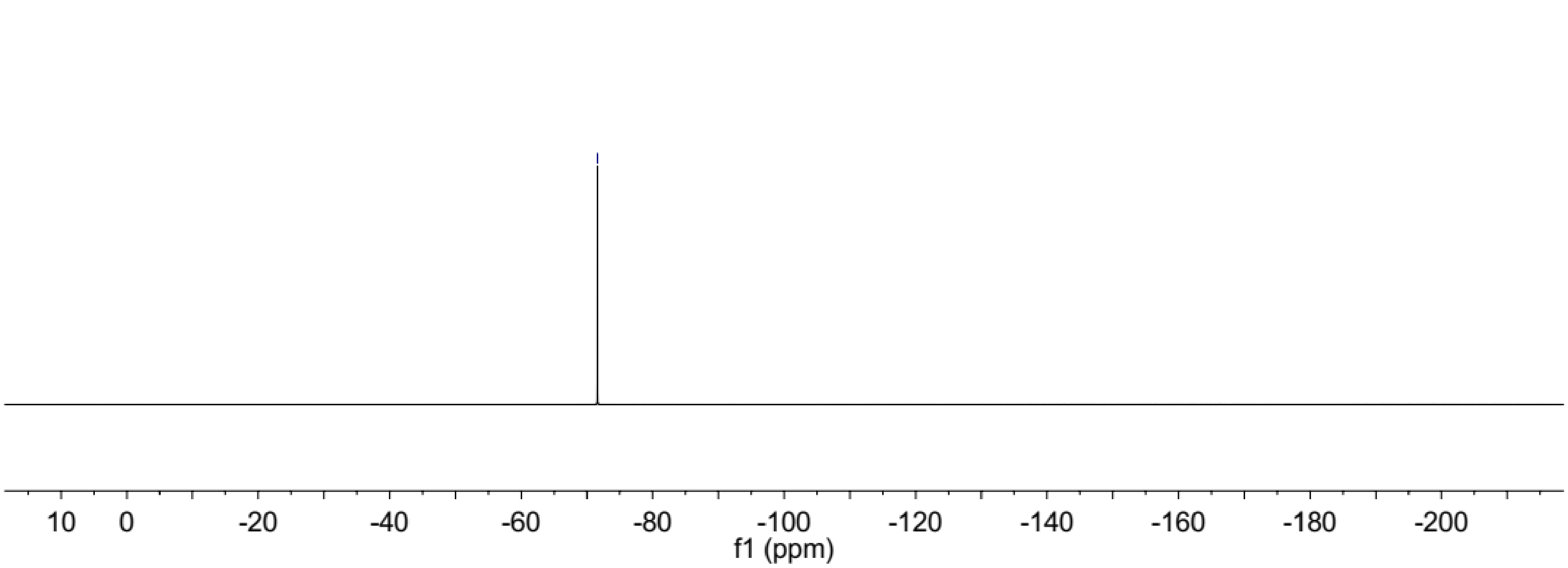

Supplementary Figure 70. ^{19}F NMR spectrum for compound **3m**

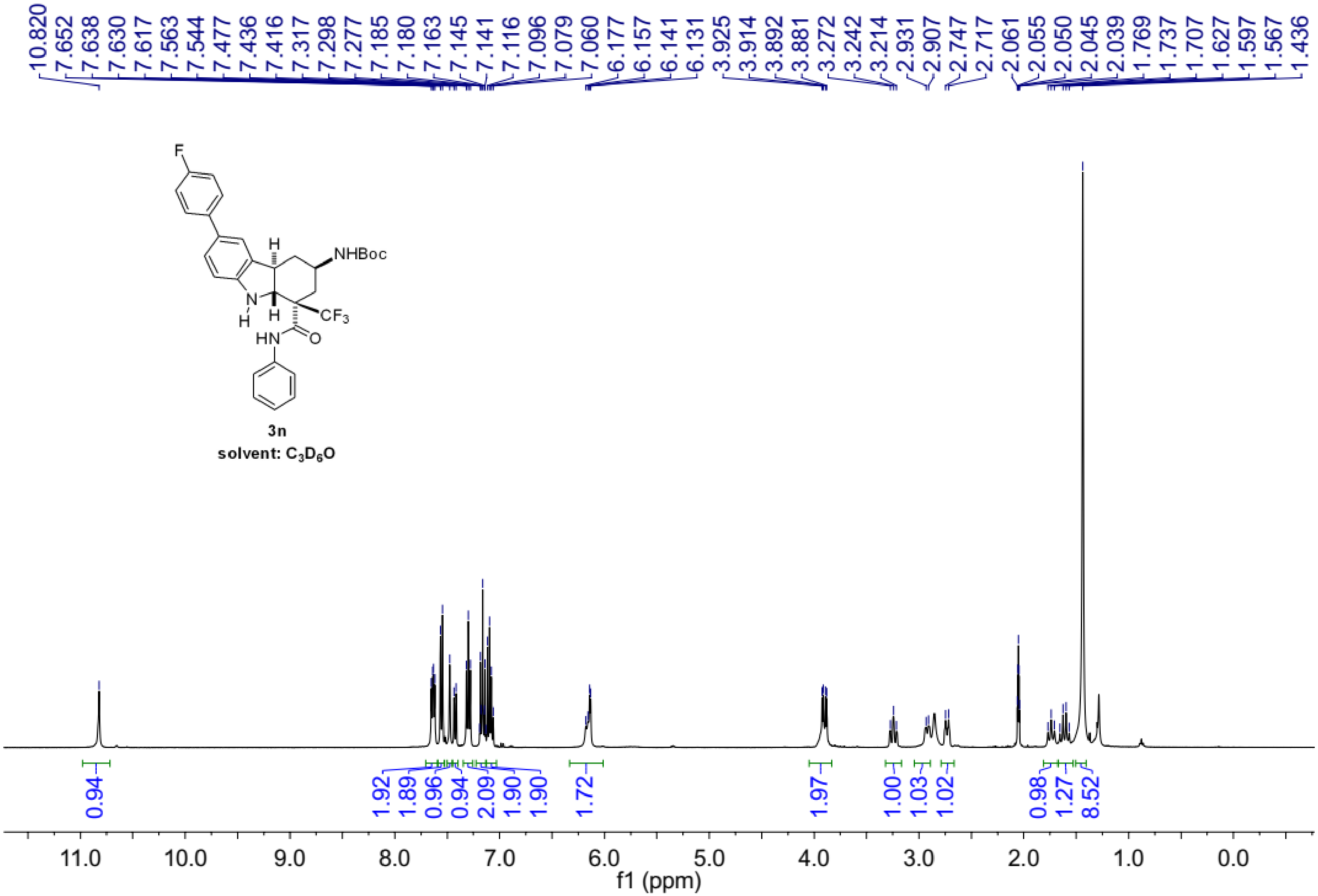

Supplementary Figure 71. ^{1}H NMR spectrum for compound **3n**

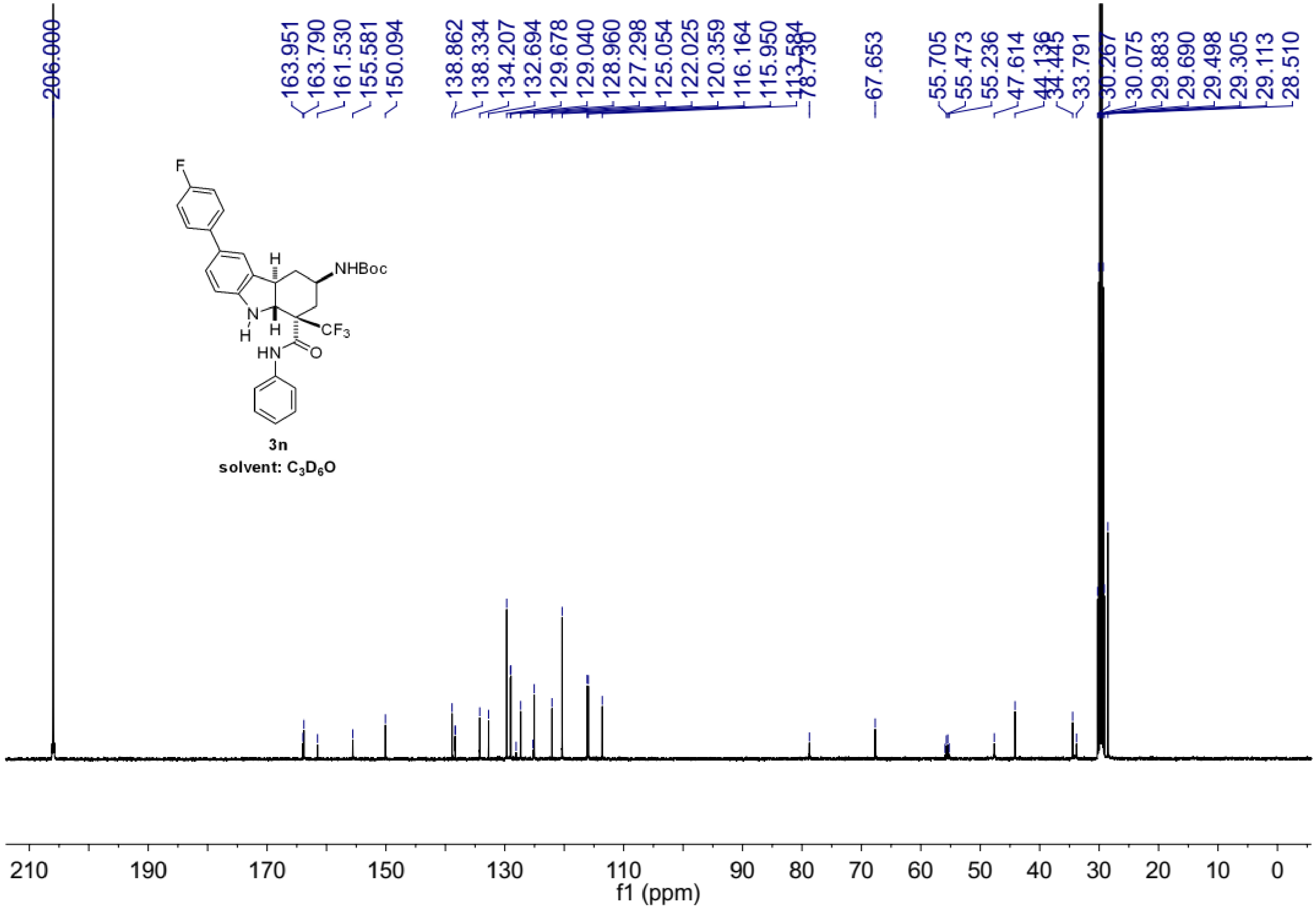

Supplementary Figure 72. ^{13}C NMR spectrum for compound **3n**

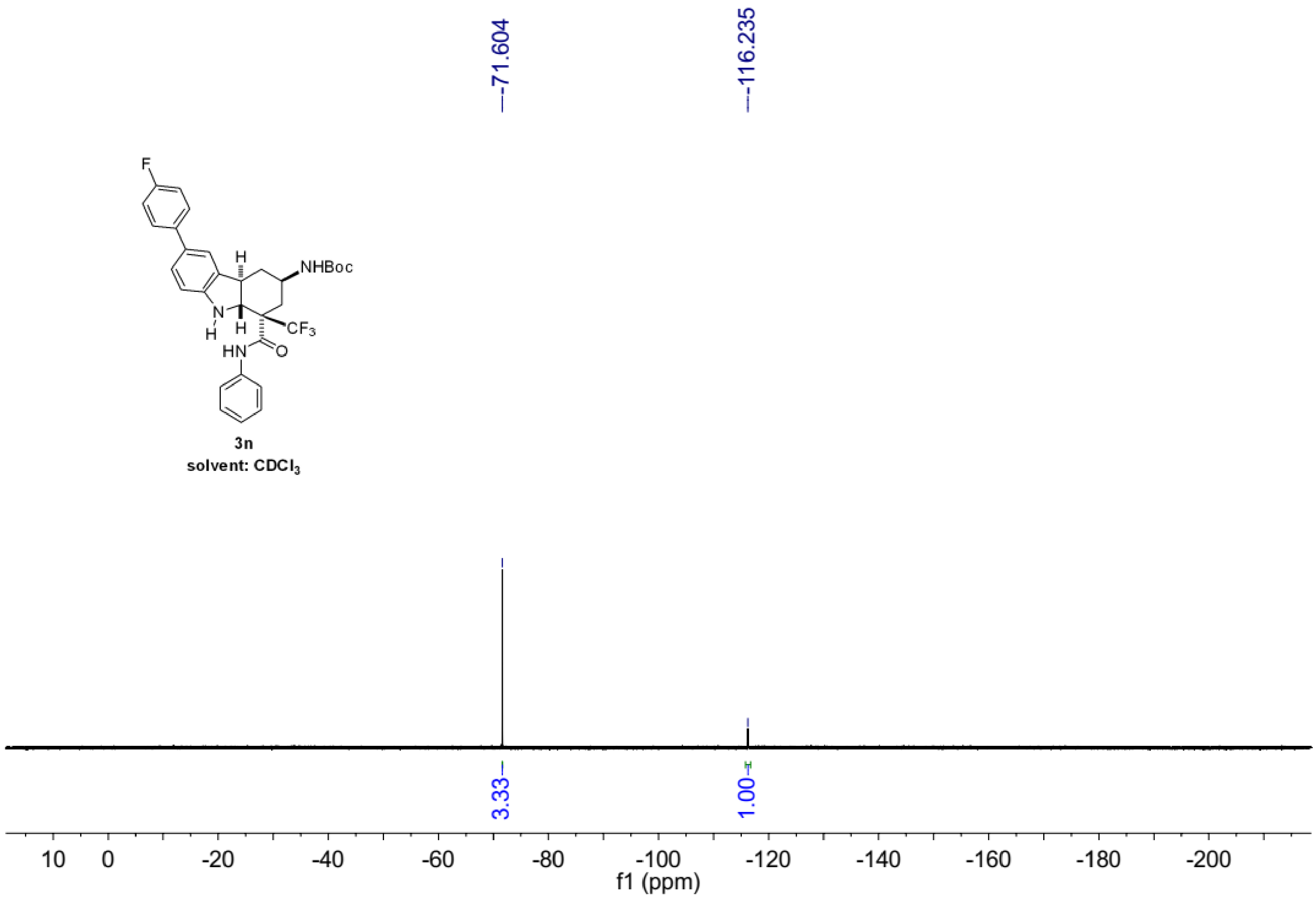

Supplementary Figure 73. ^{19}F NMR spectrum for compound **3n**

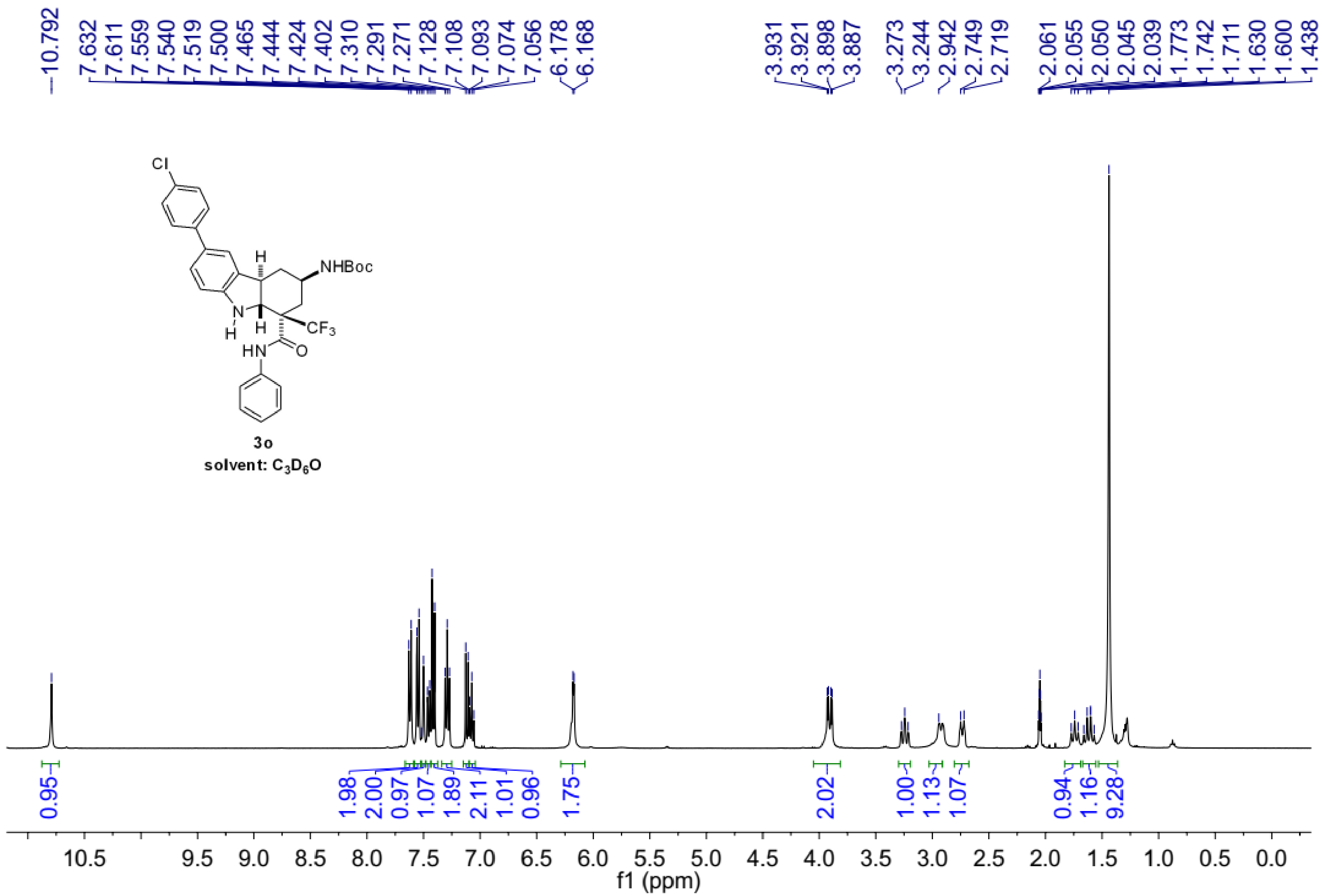

Supplementary Figure 74. ^{1}H NMR spectrum for compound **3o**

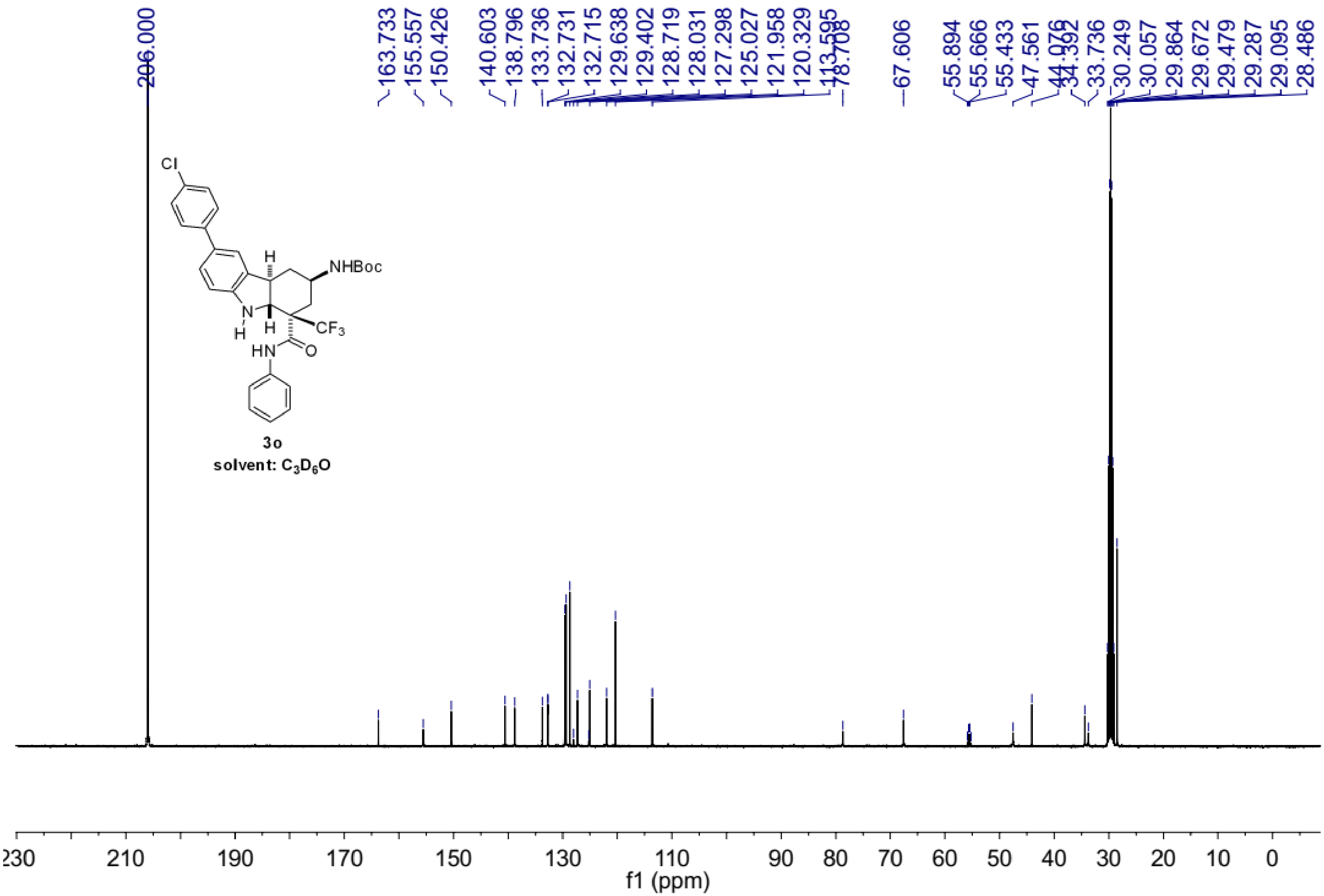

Supplementary Figure 75. ^{13}C NMR spectrum for compound **3o**

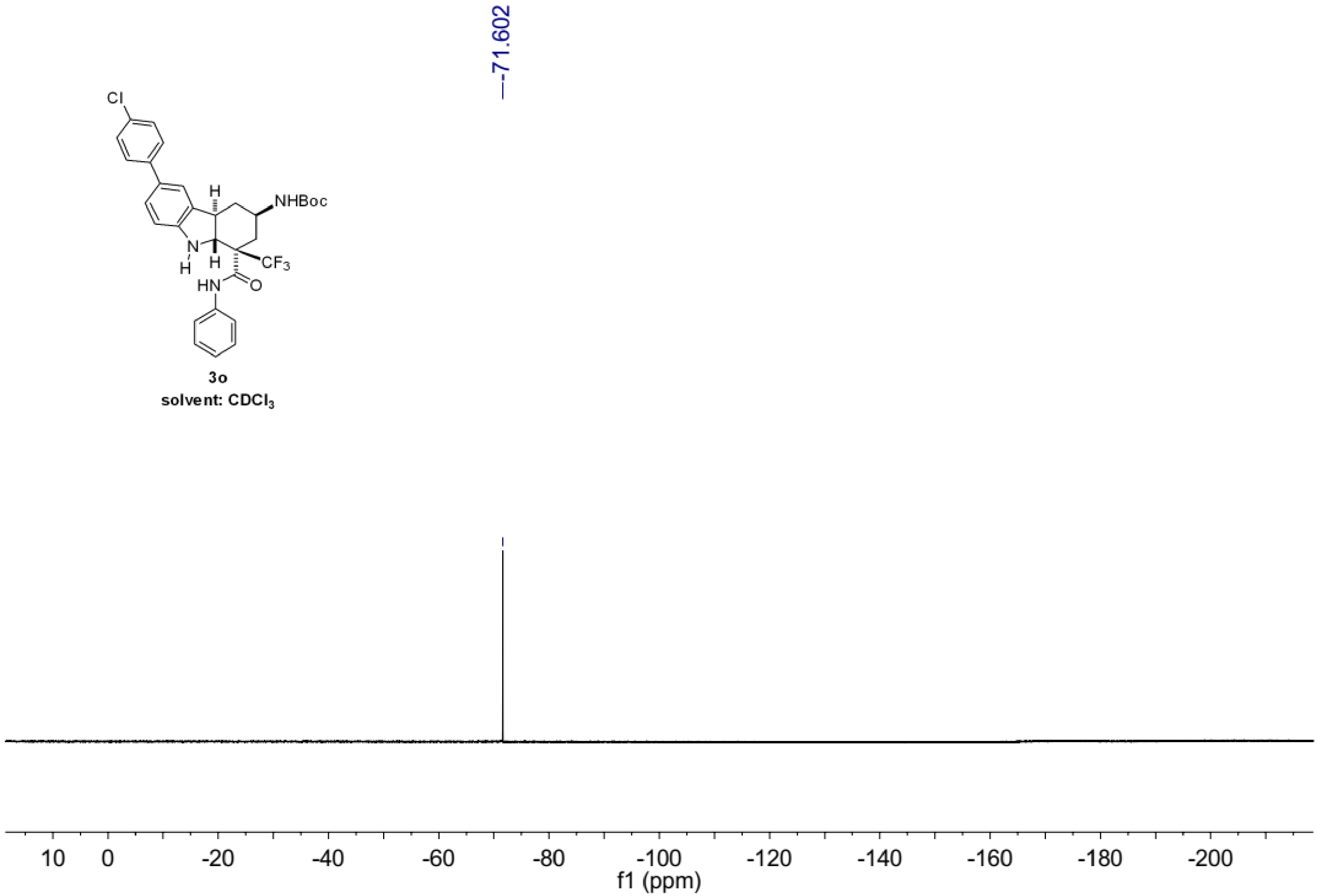

Supplementary Figure 76. ^{19}F NMR spectrum for compound **3o**

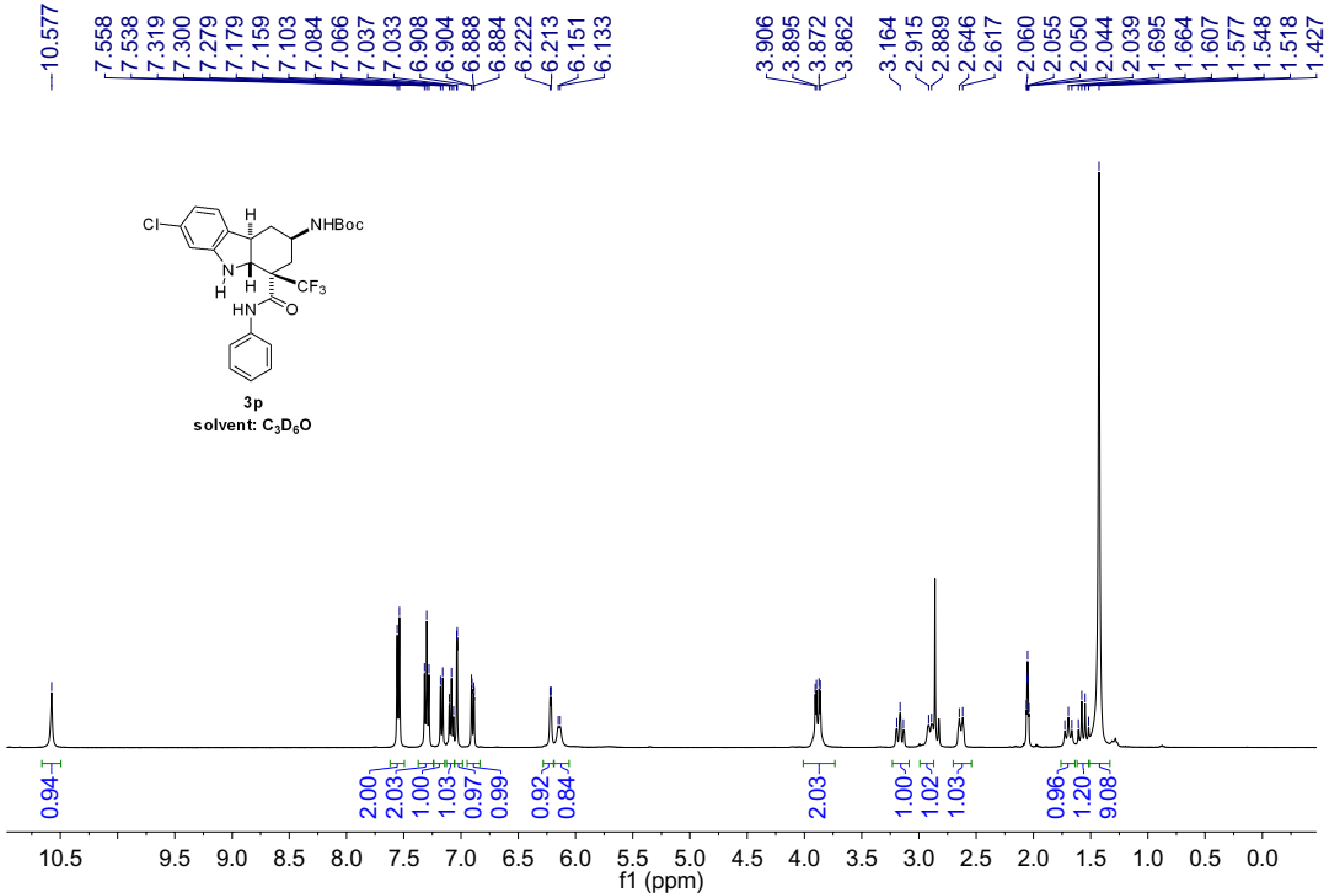

Supplementary Figure 77. ^{1}H NMR spectrum for compound **3p**

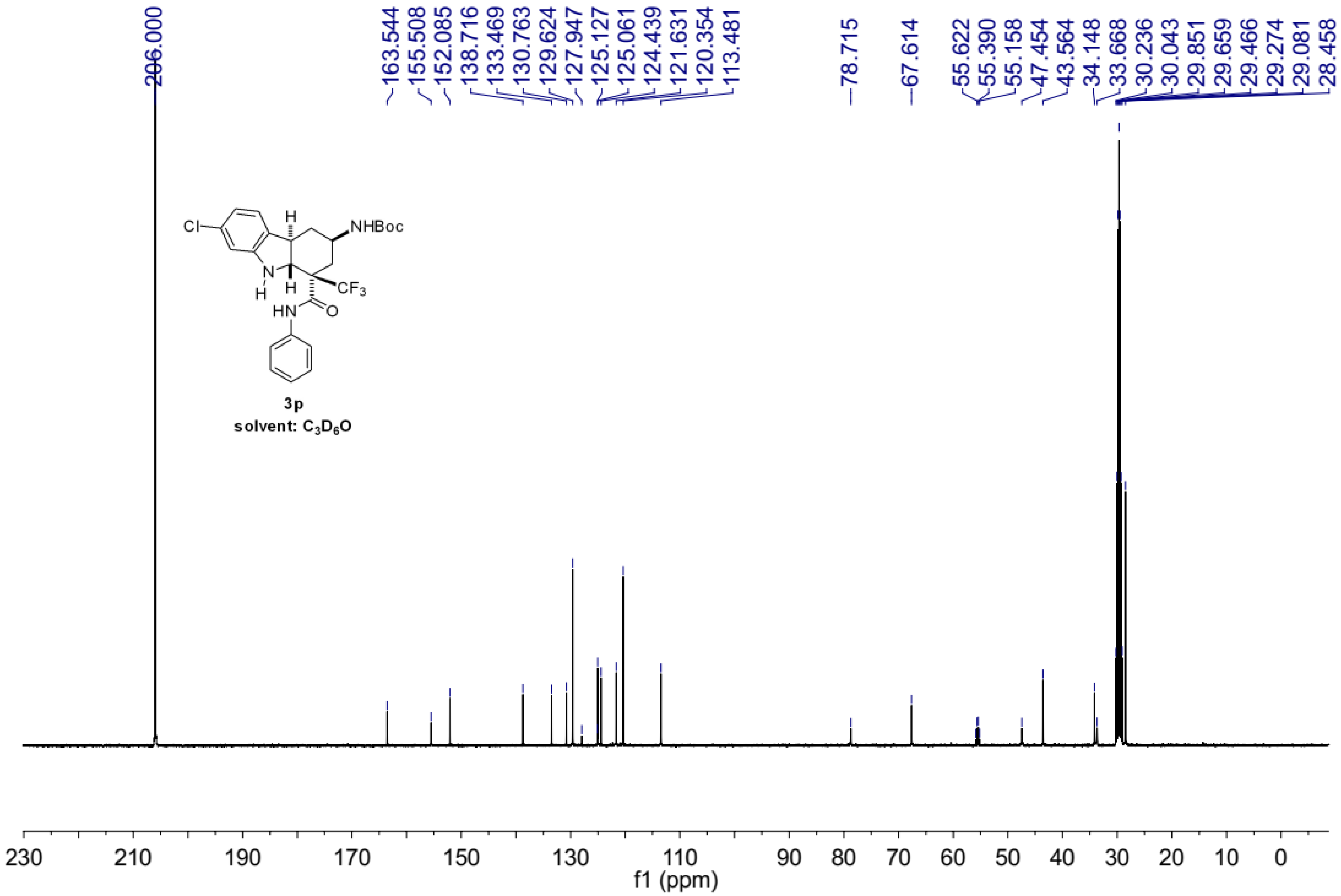

Supplementary Figure 78. ^{13}C NMR spectrum for compound **3p**

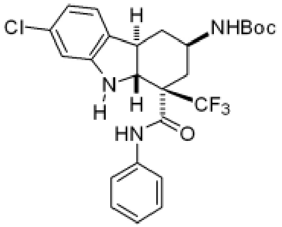

3p
solvent: $CDCl_3$

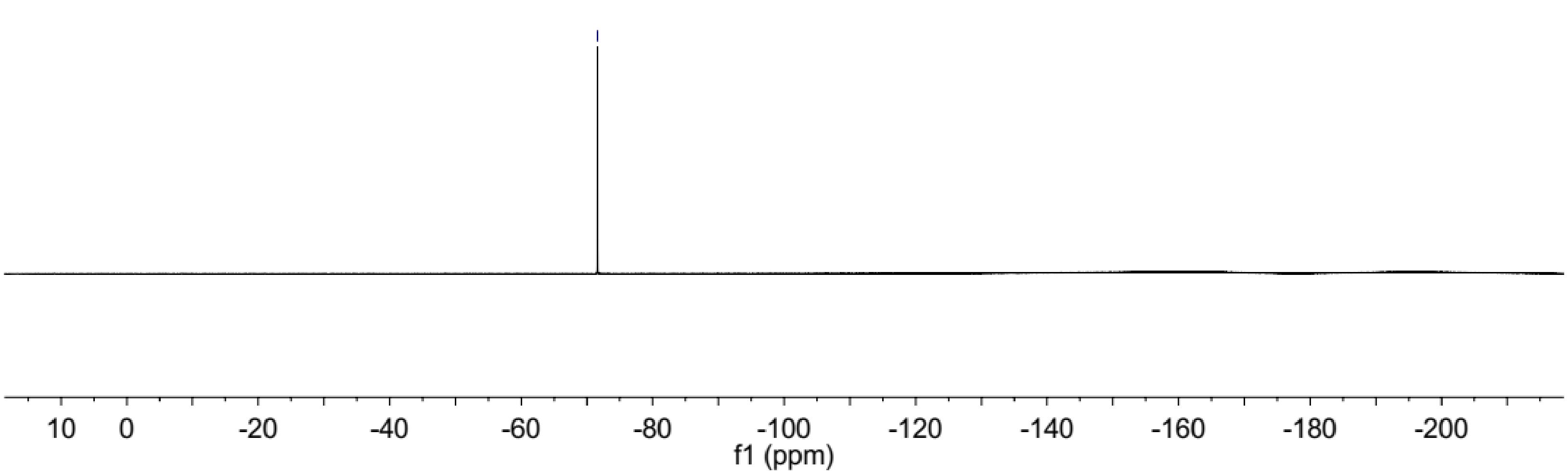

Supplementary Figure 79. ^{19}F NMR spectrum for compound **3p**

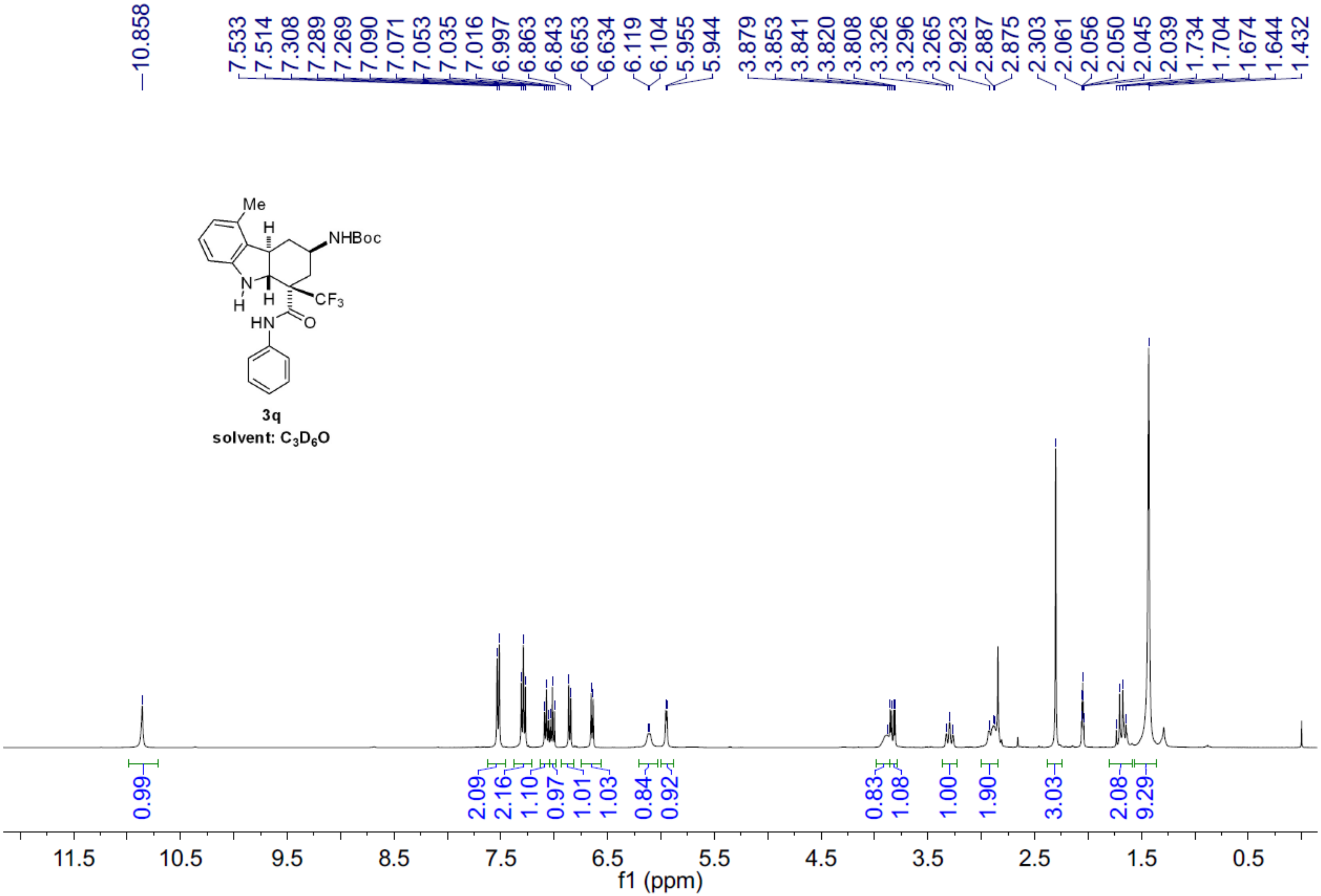

Supplementary Figure 80. ^{1}H NMR spectrum for compound **3q**

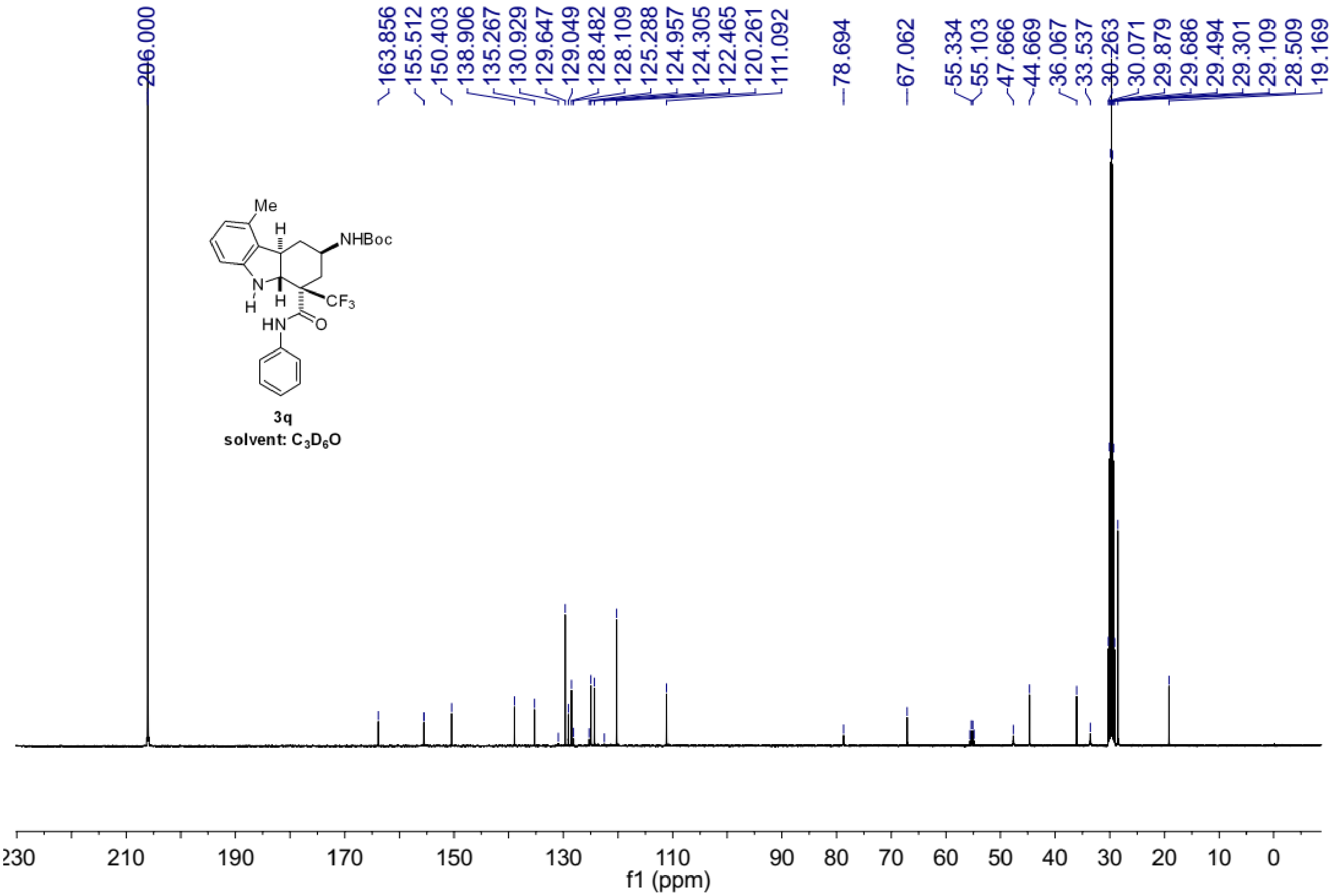

Supplementary Figure 81. ^{13}C NMR spectrum for compound **3q**

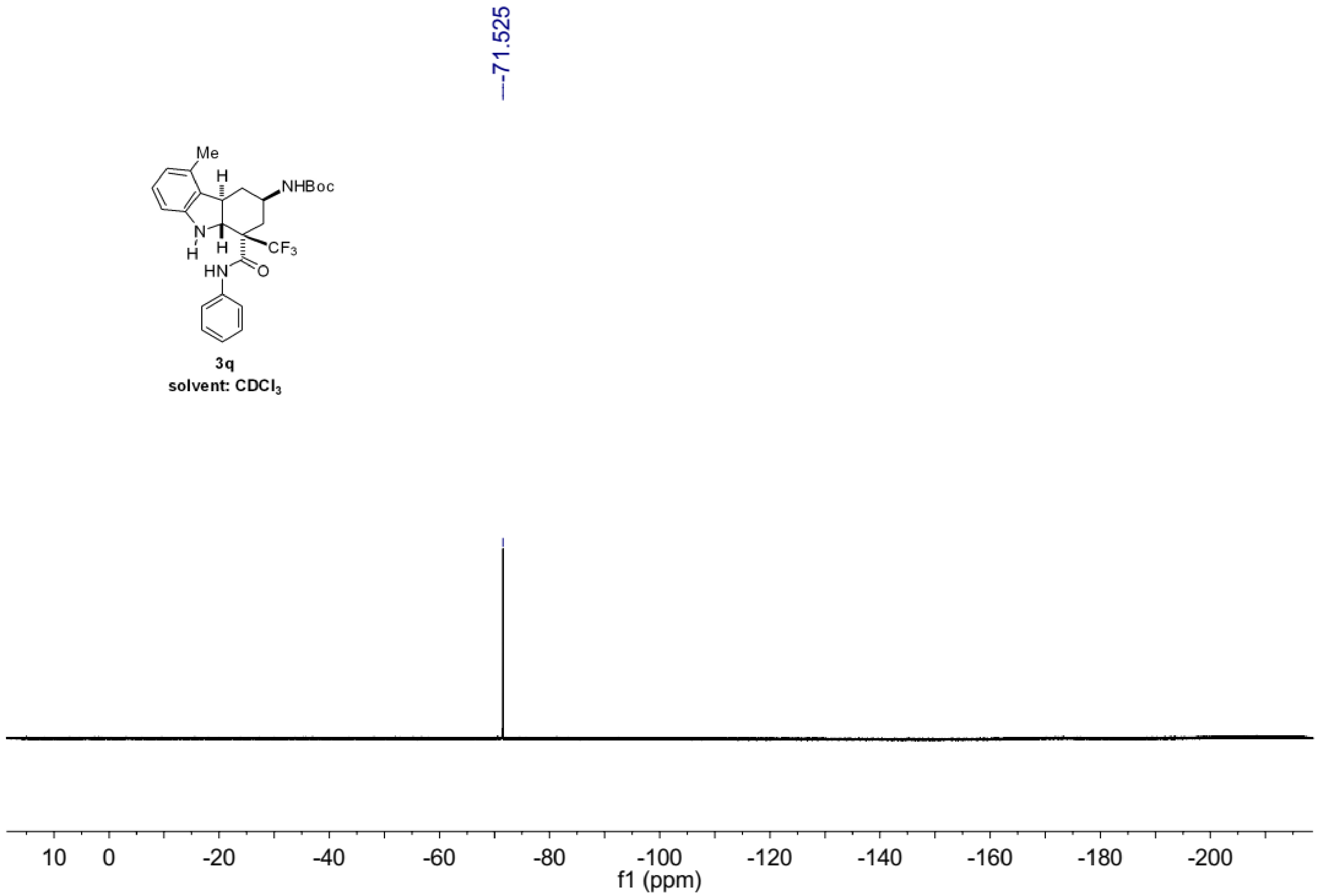

Supplementary Figure 82. ^{19}F NMR spectrum for compound **3q**

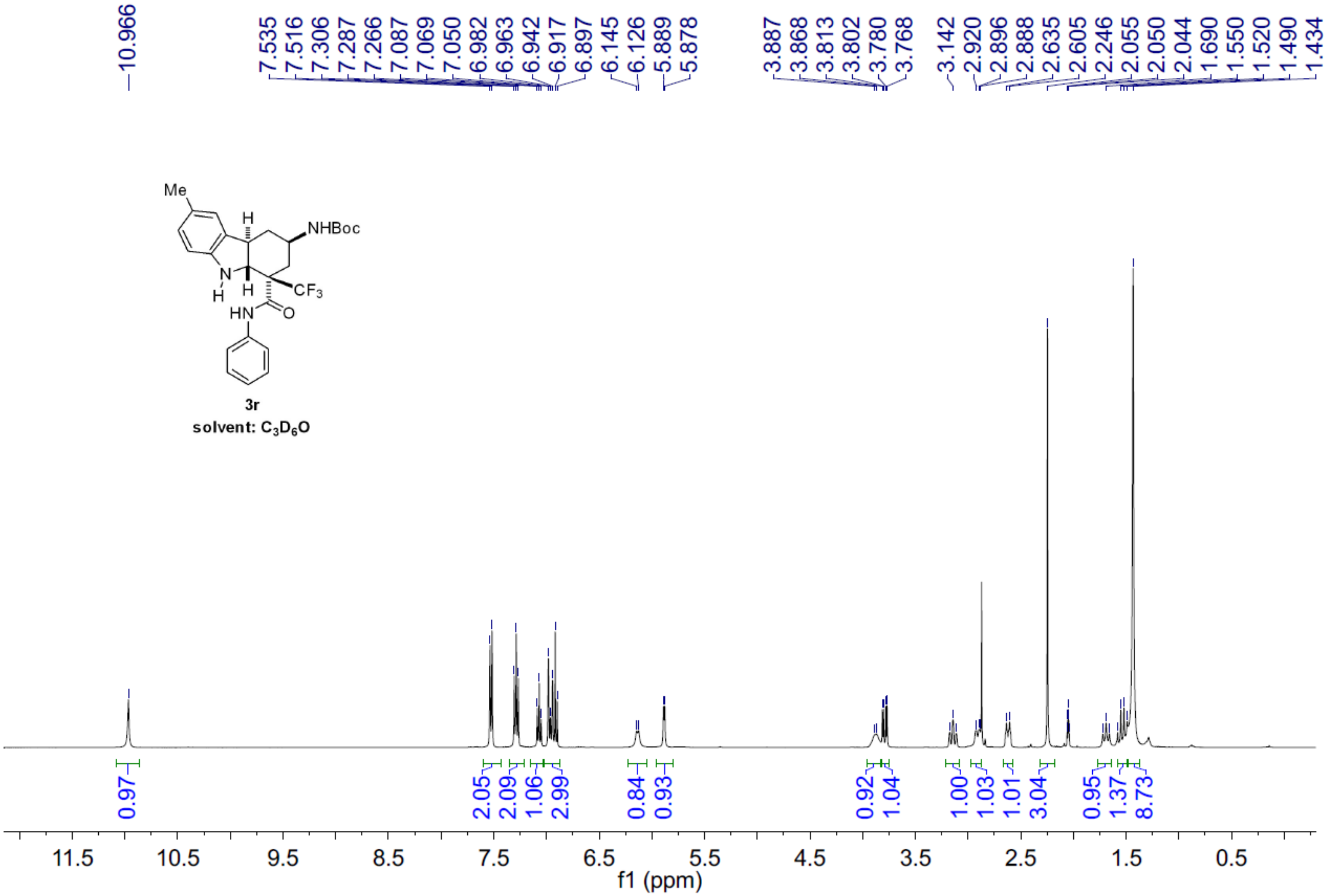

Supplementary Figure 83. ^{1}H NMR spectrum for compound **3r**

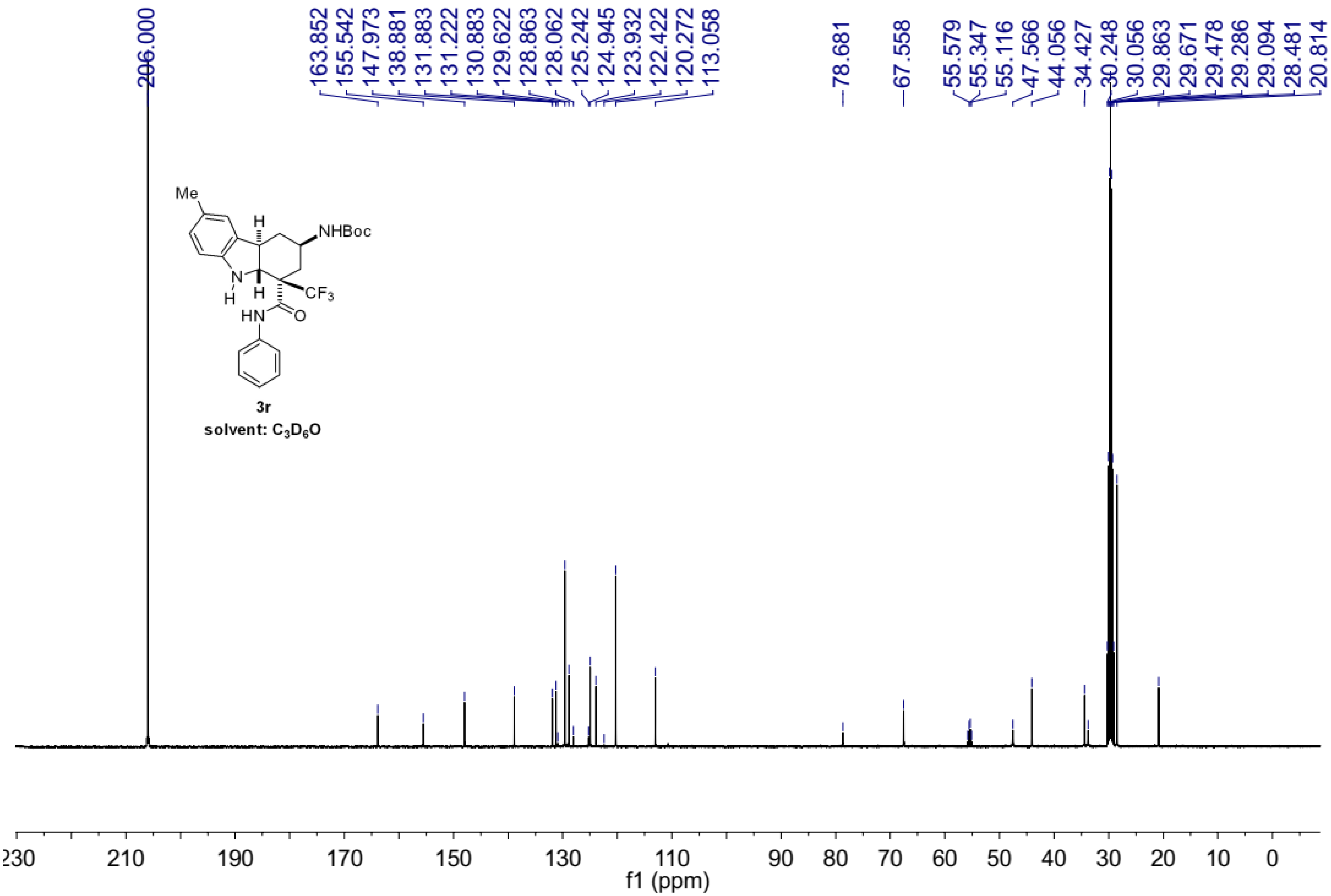

Supplementary Figure 84. ^{13}C NMR spectrum for compound **3r**

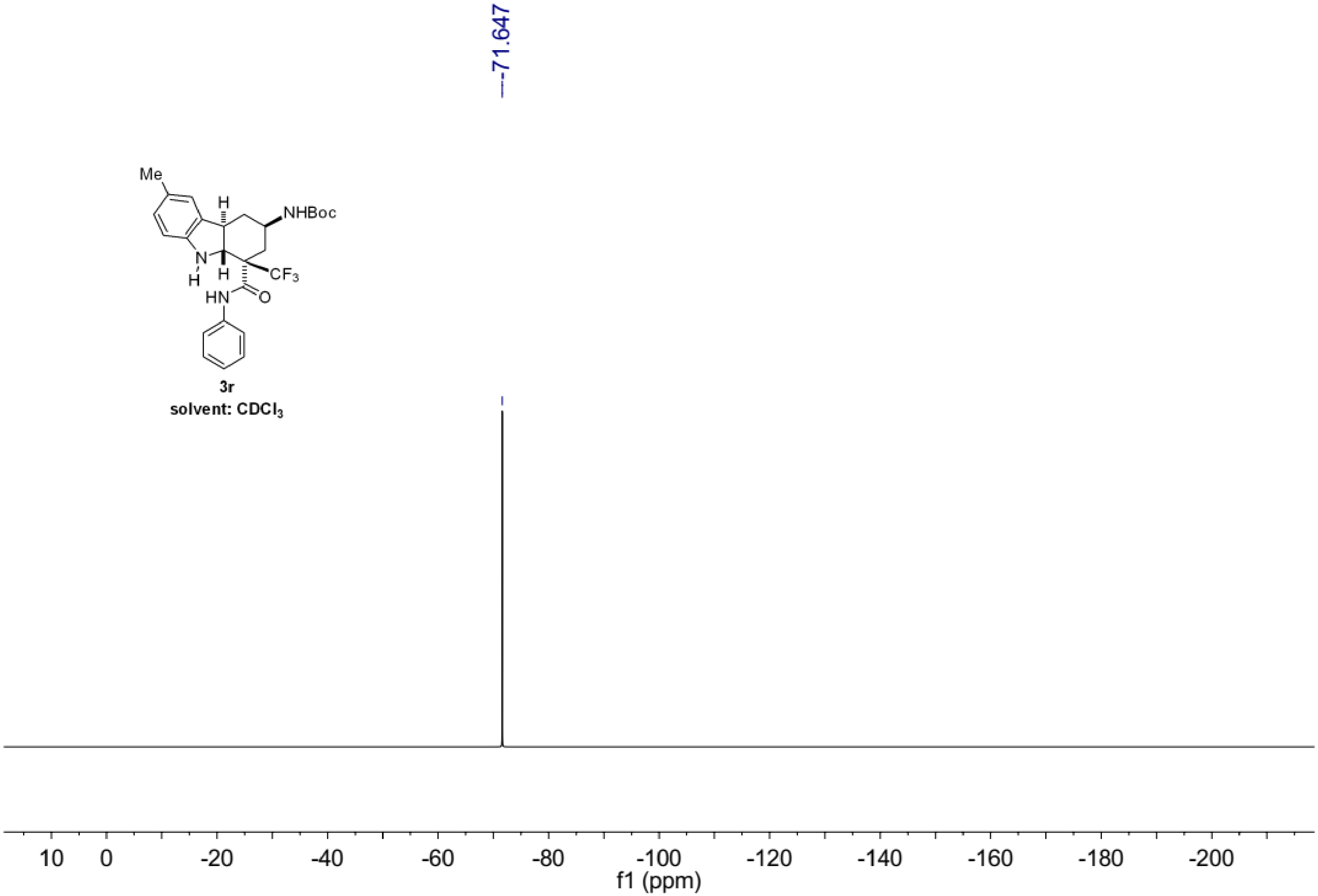

Supplementary Figure 85. ^{19}F NMR spectrum for compound **3r**

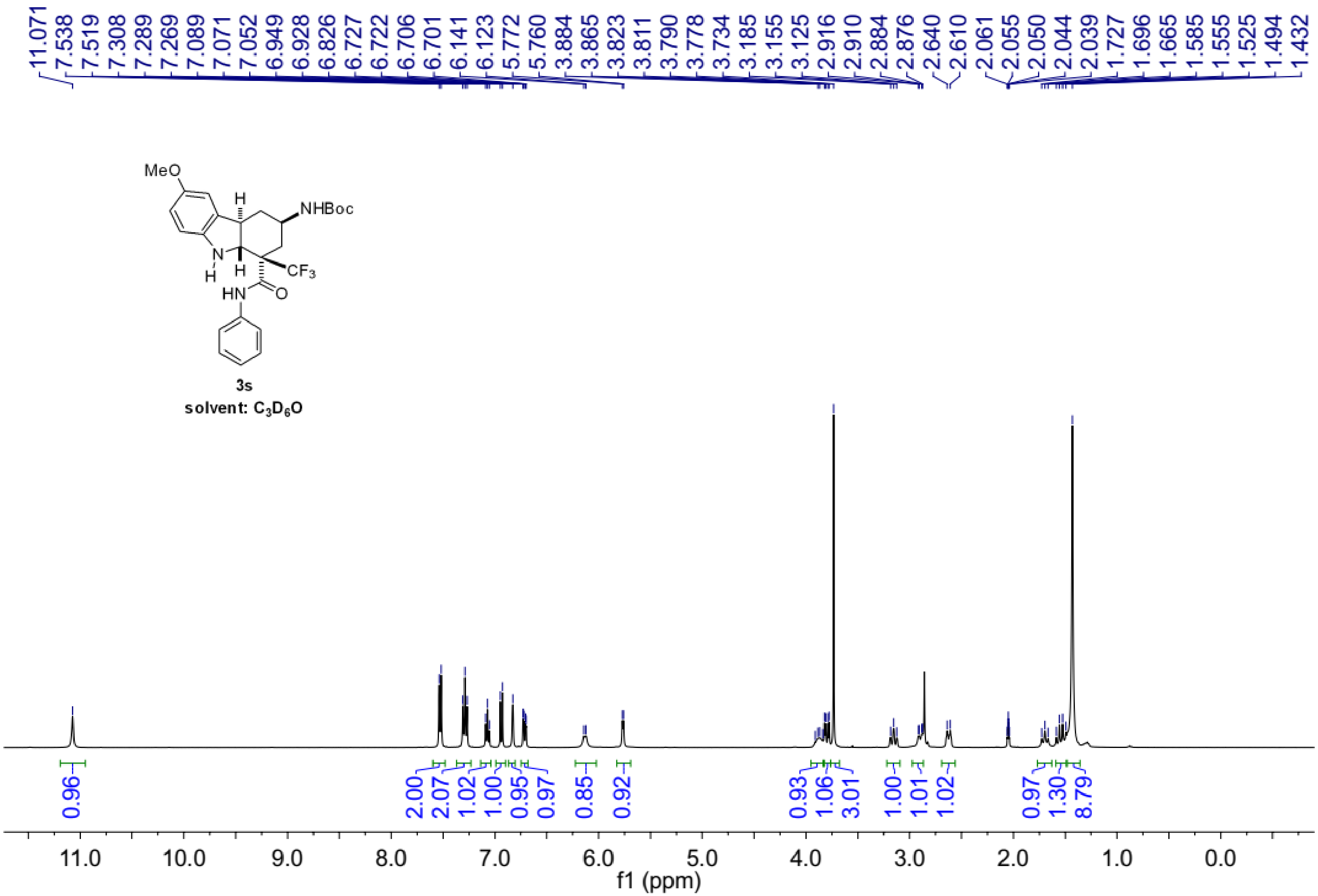

Supplementary Figure 86. ^{1}H NMR spectrum for compound **3s**

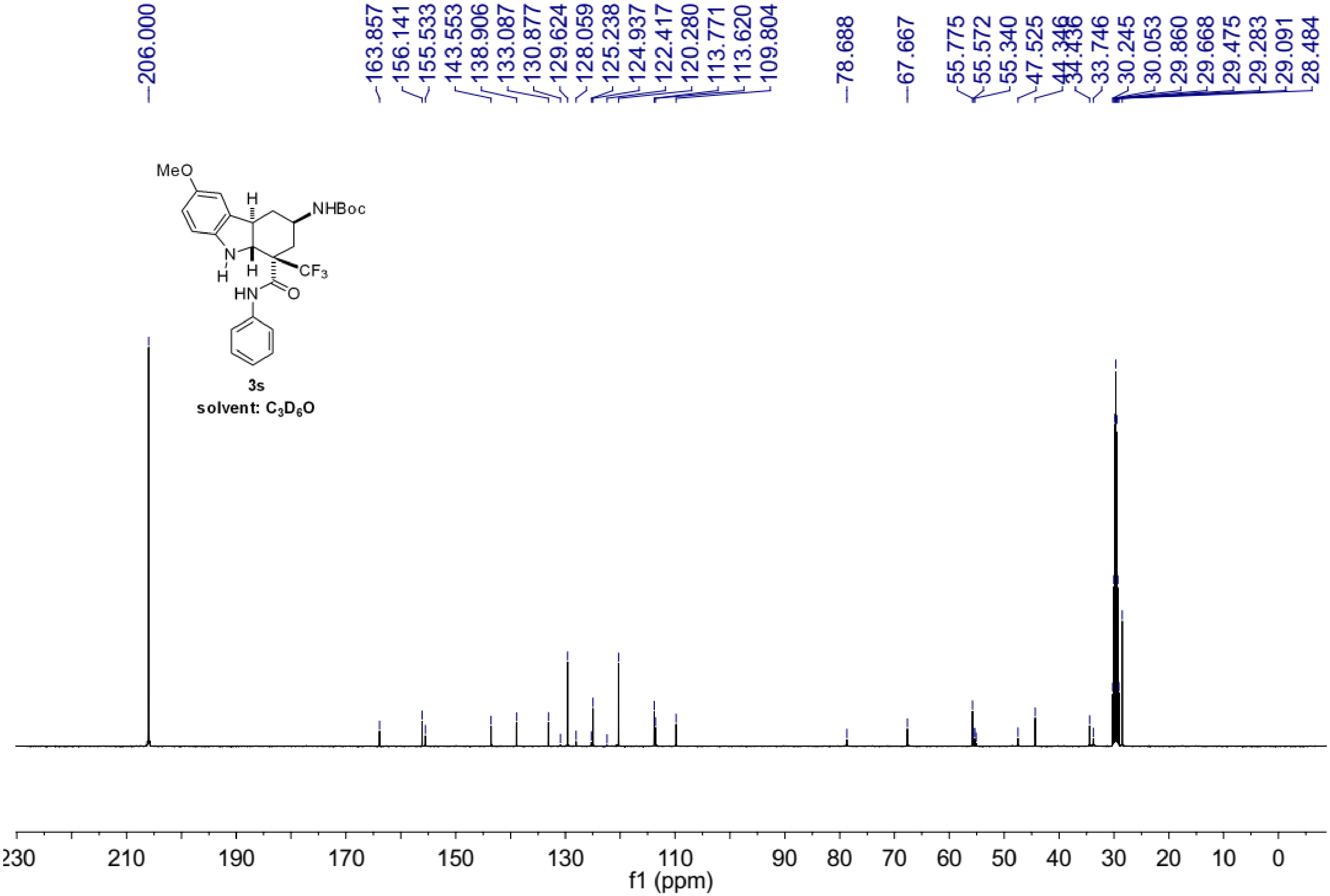

Supplementary Figure 87. ^{13}C NMR spectrum for compound **3s**

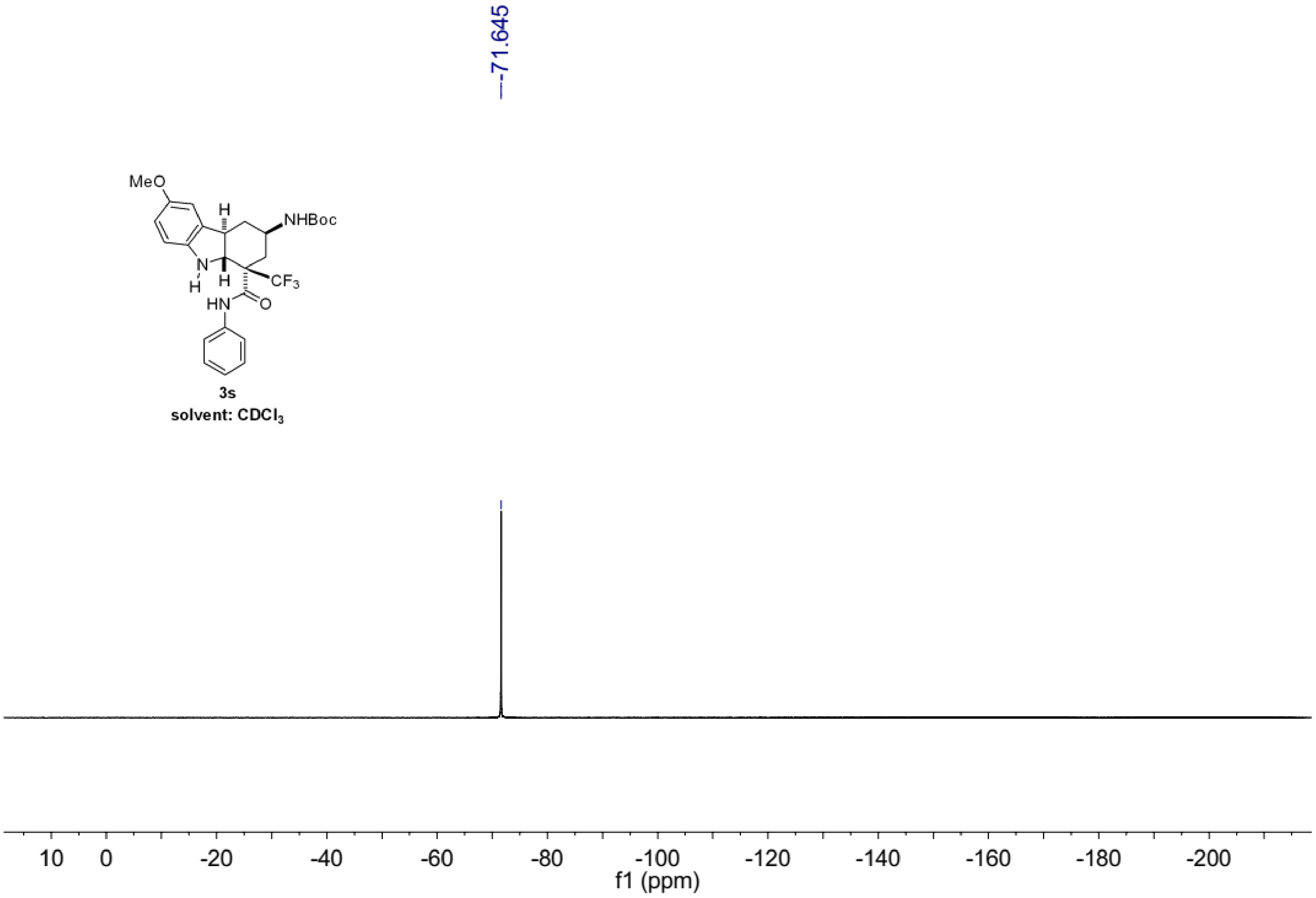

Supplementary Figure 88. ^{19}F NMR spectrum for compound **3s**

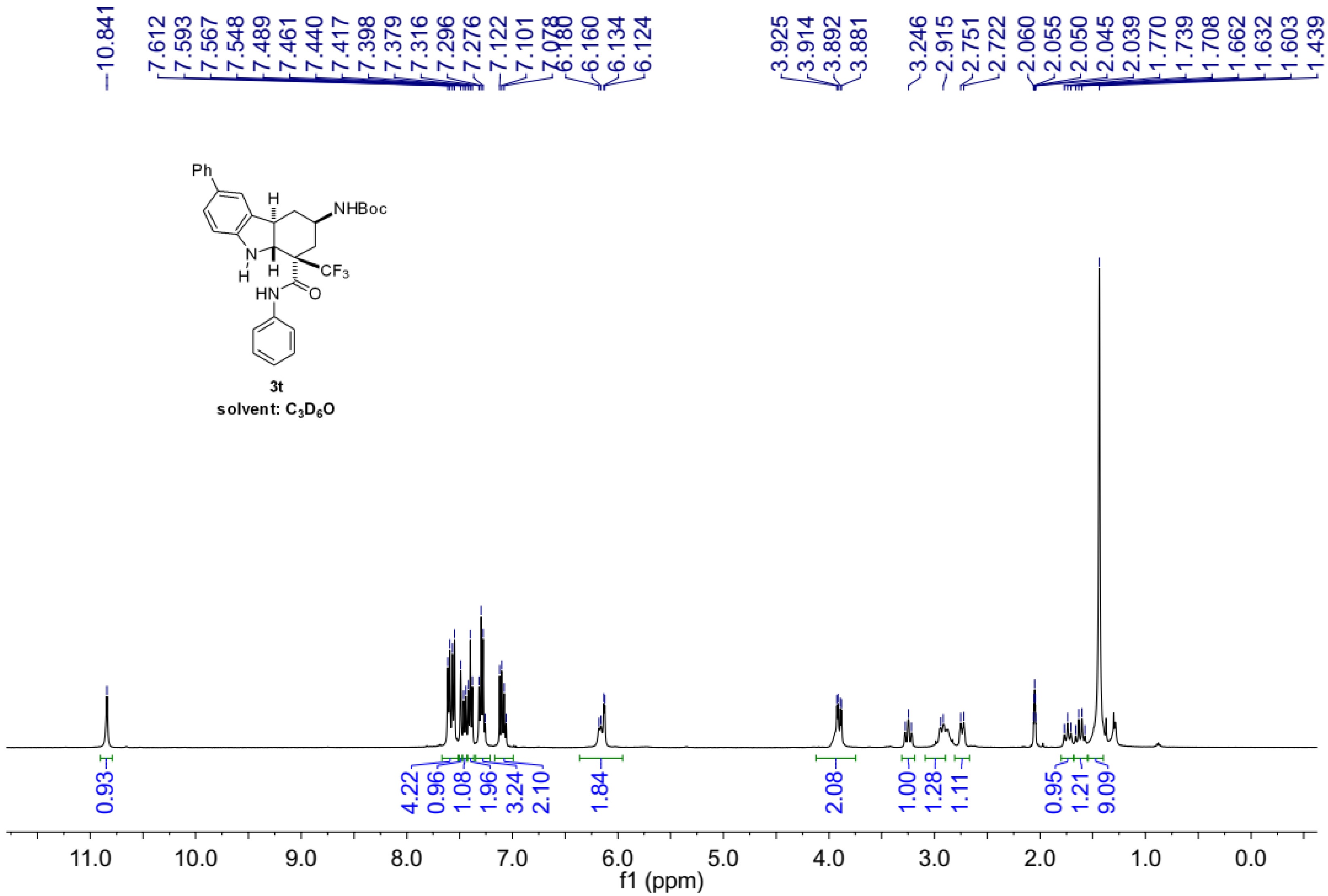

Supplementary Figure 89. ^{1}H NMR spectrum for compound **3t**

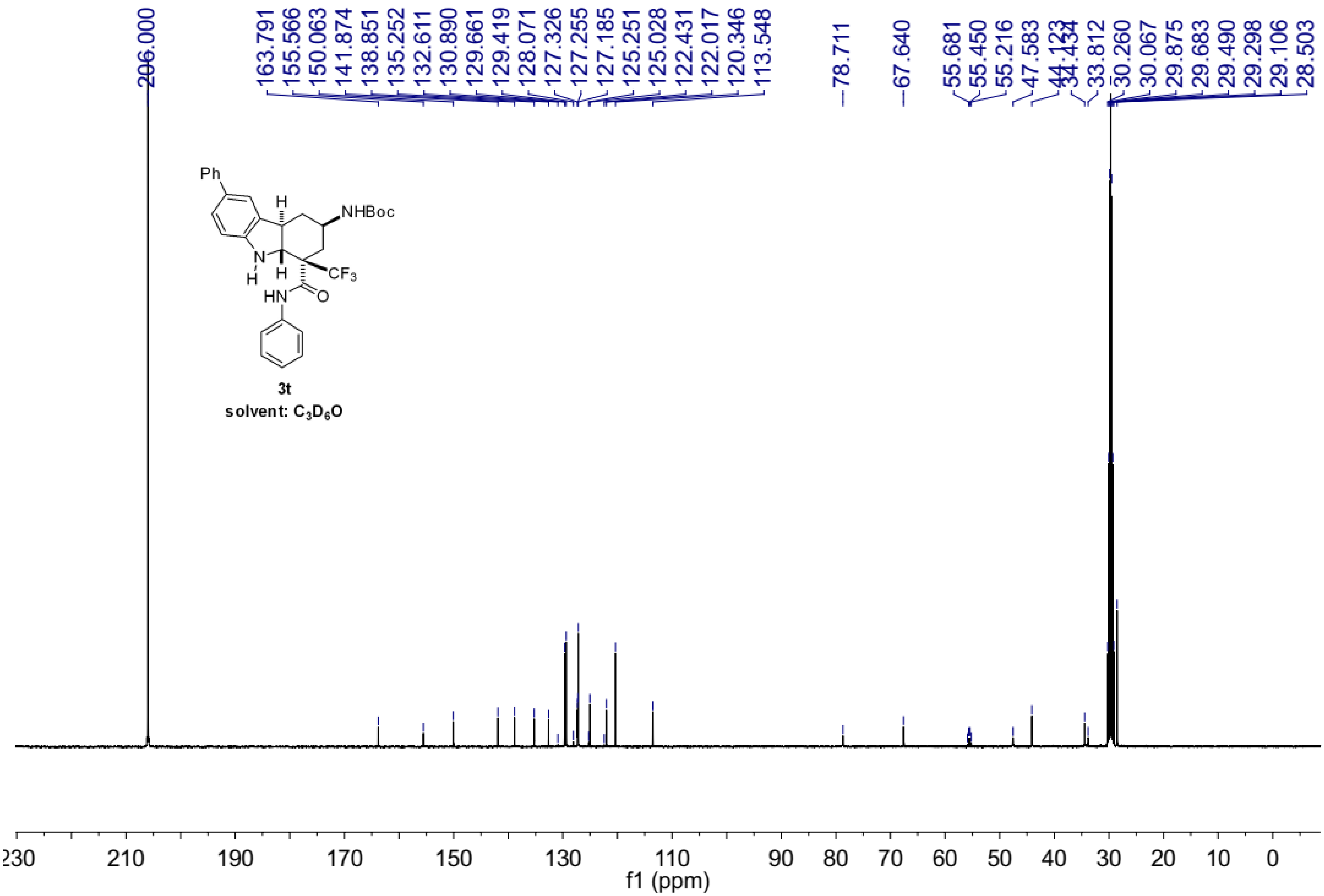

Supplementary Figure 90. ^{13}C NMR spectrum for compound **3t**

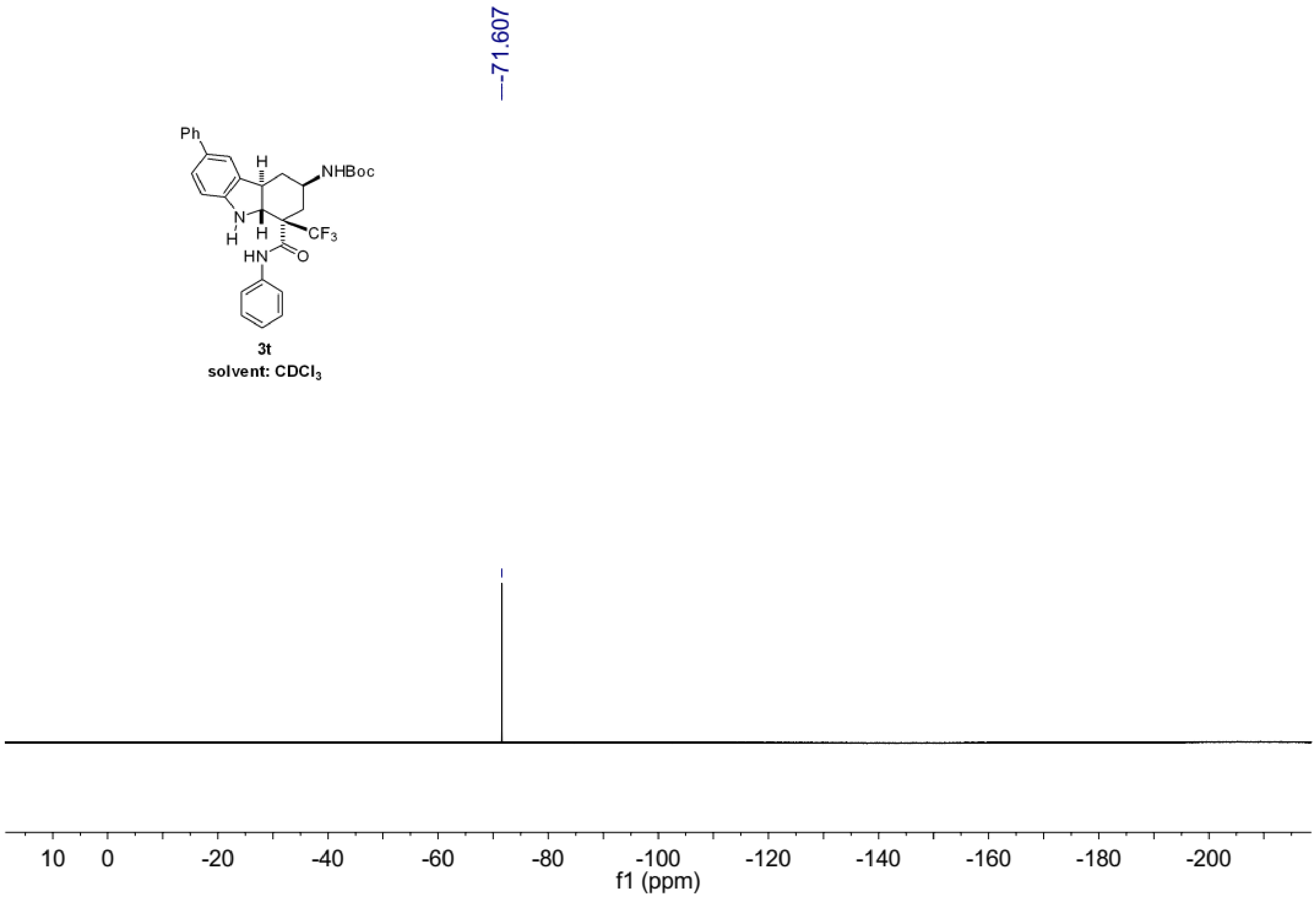

Supplementary Figure 91. ^{19}F NMR spectrum for compound **3t**

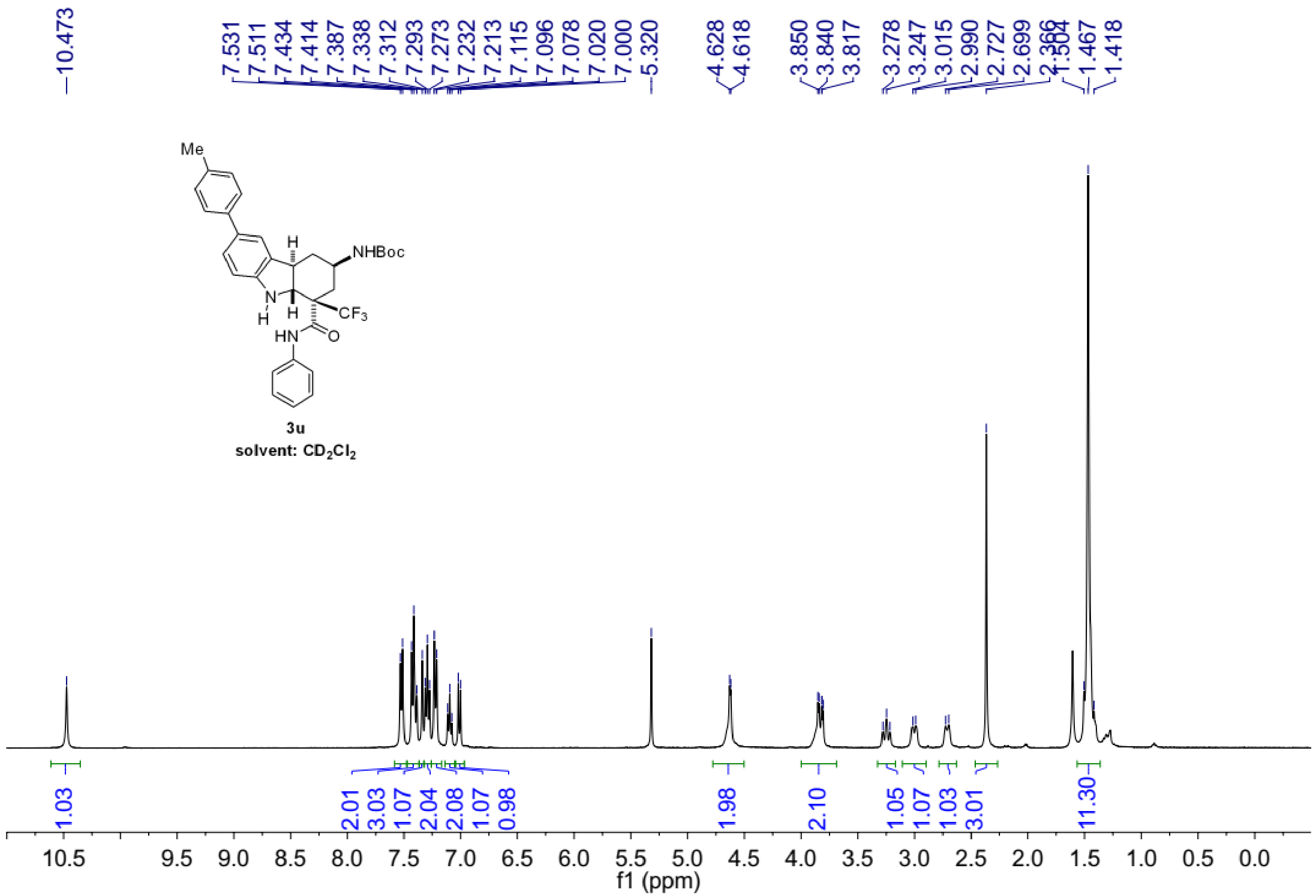

Supplementary Figure 92. ^{1}H NMR spectrum for compound **3u**

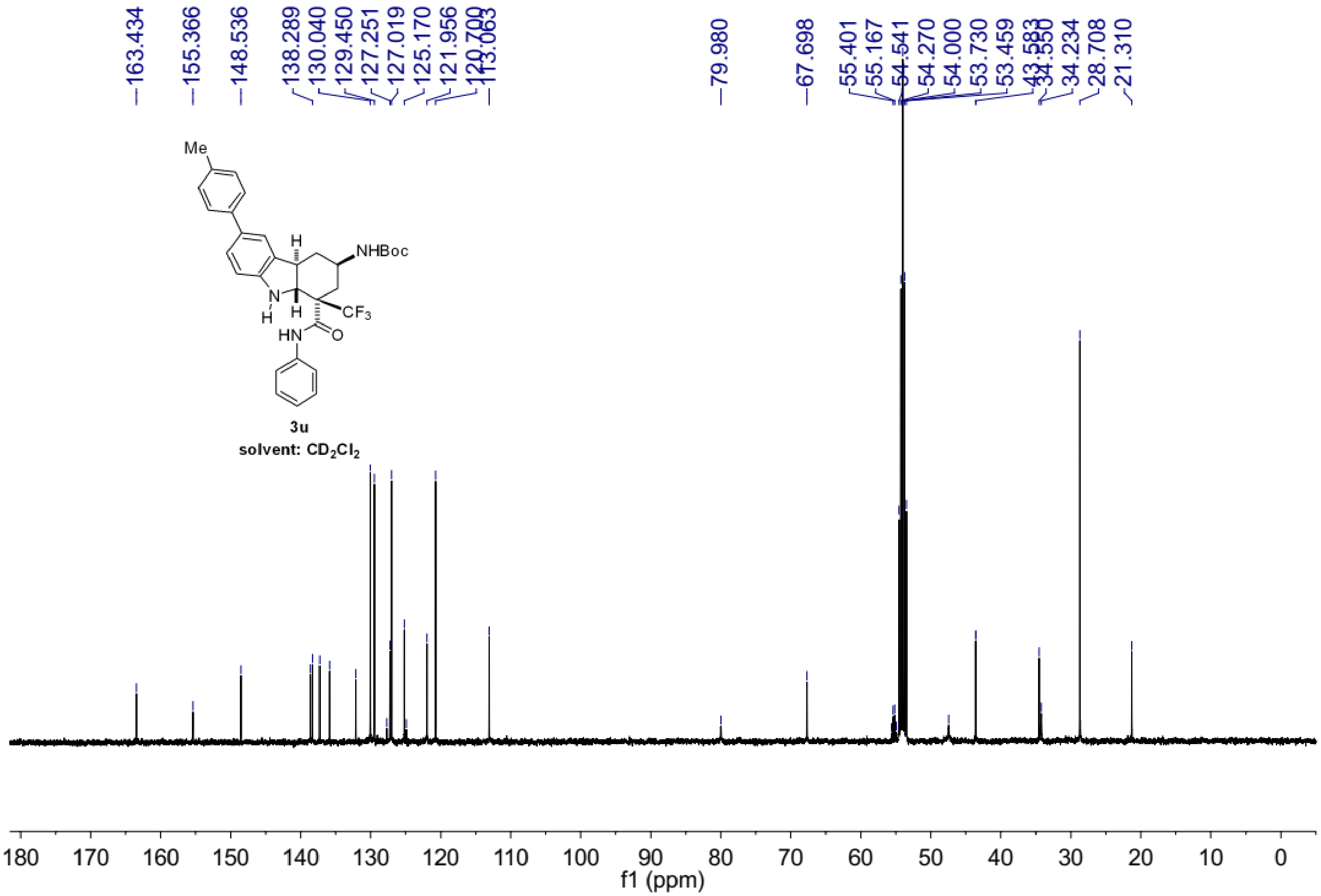

Supplementary Figure 93. ^{13}C NMR spectrum for compound **3u**

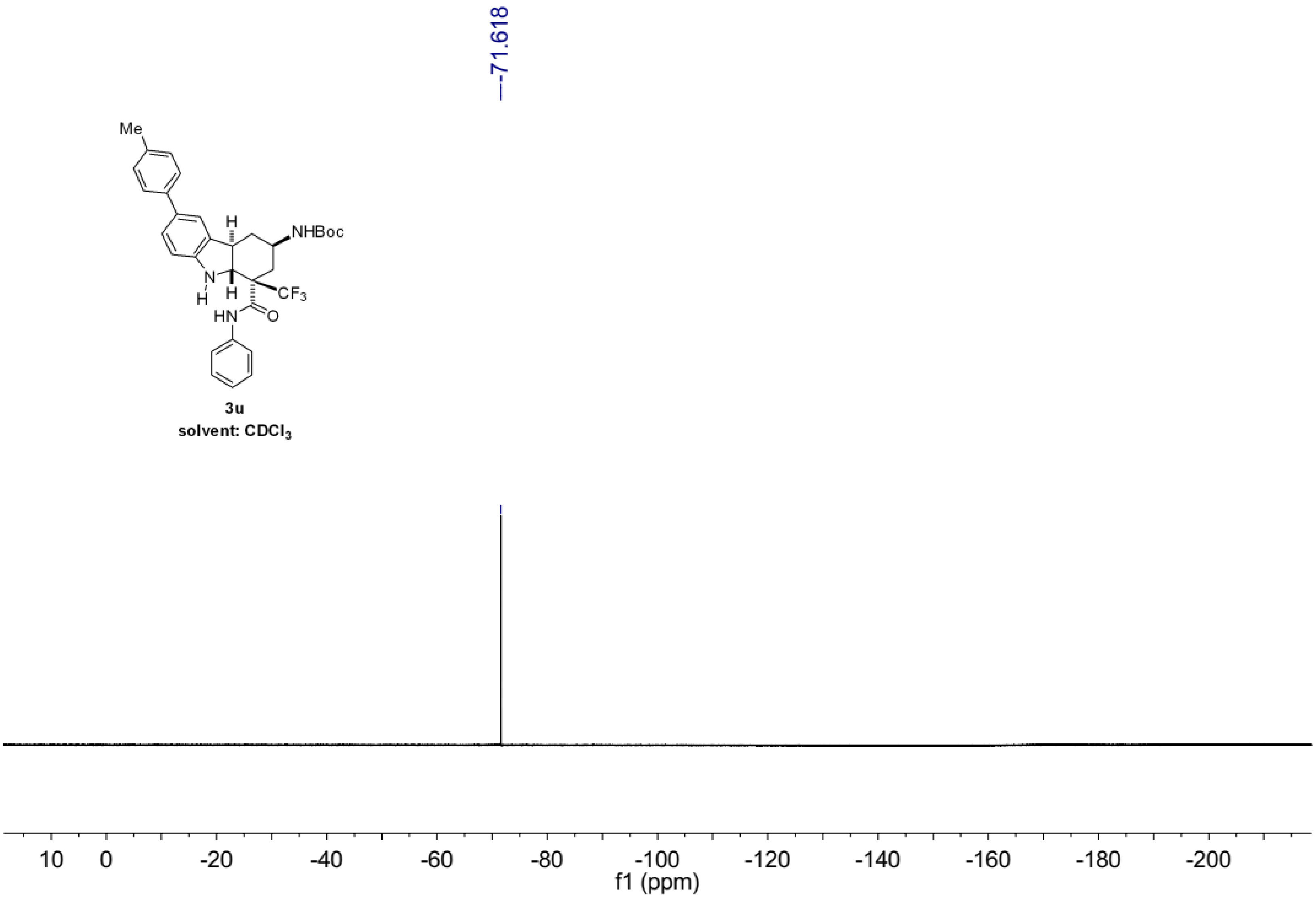

Supplementary Figure 94. ^{19}F NMR spectrum for compound **3u**

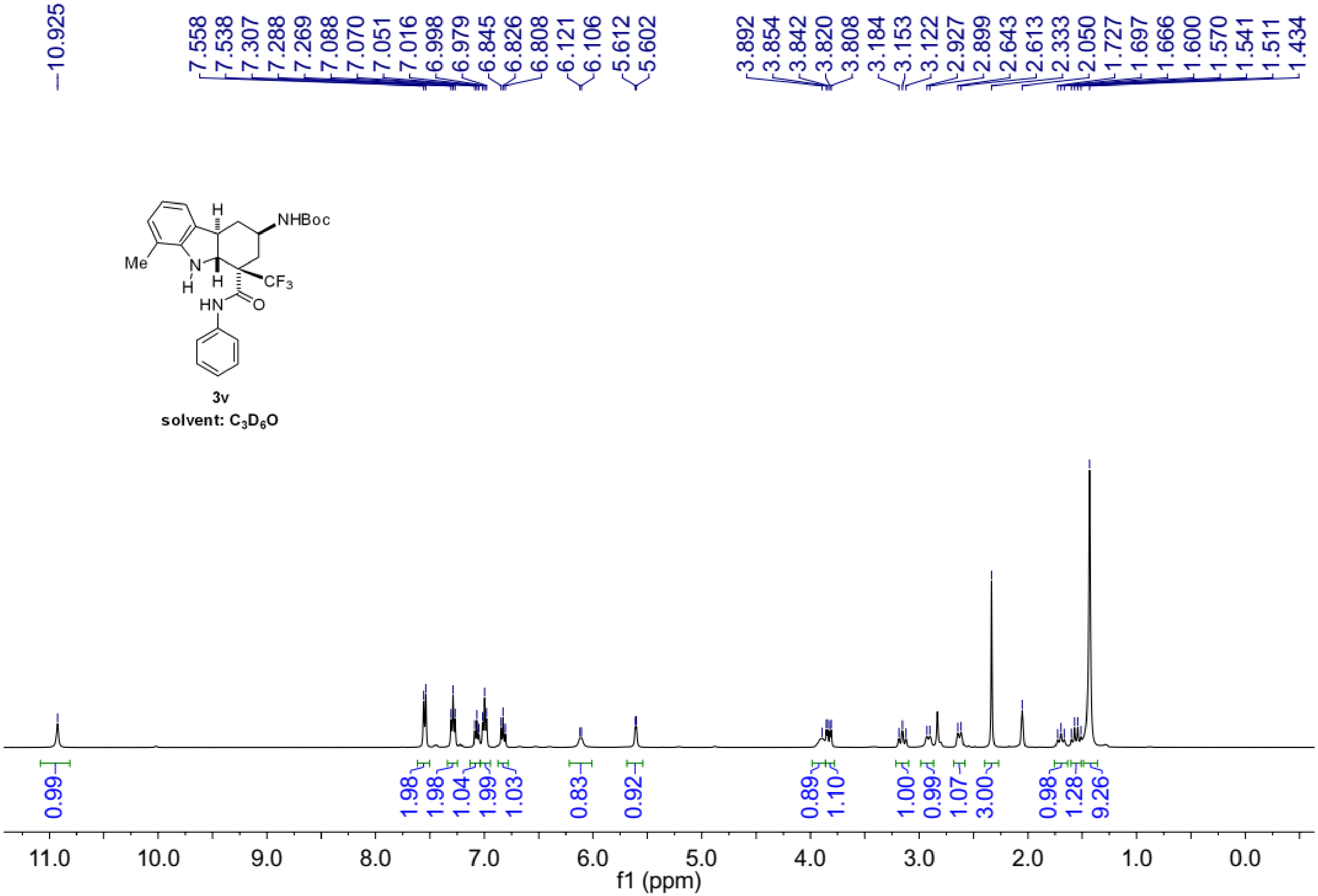

Supplementary Figure 95. ^{1}H NMR spectrum for compound **3v**

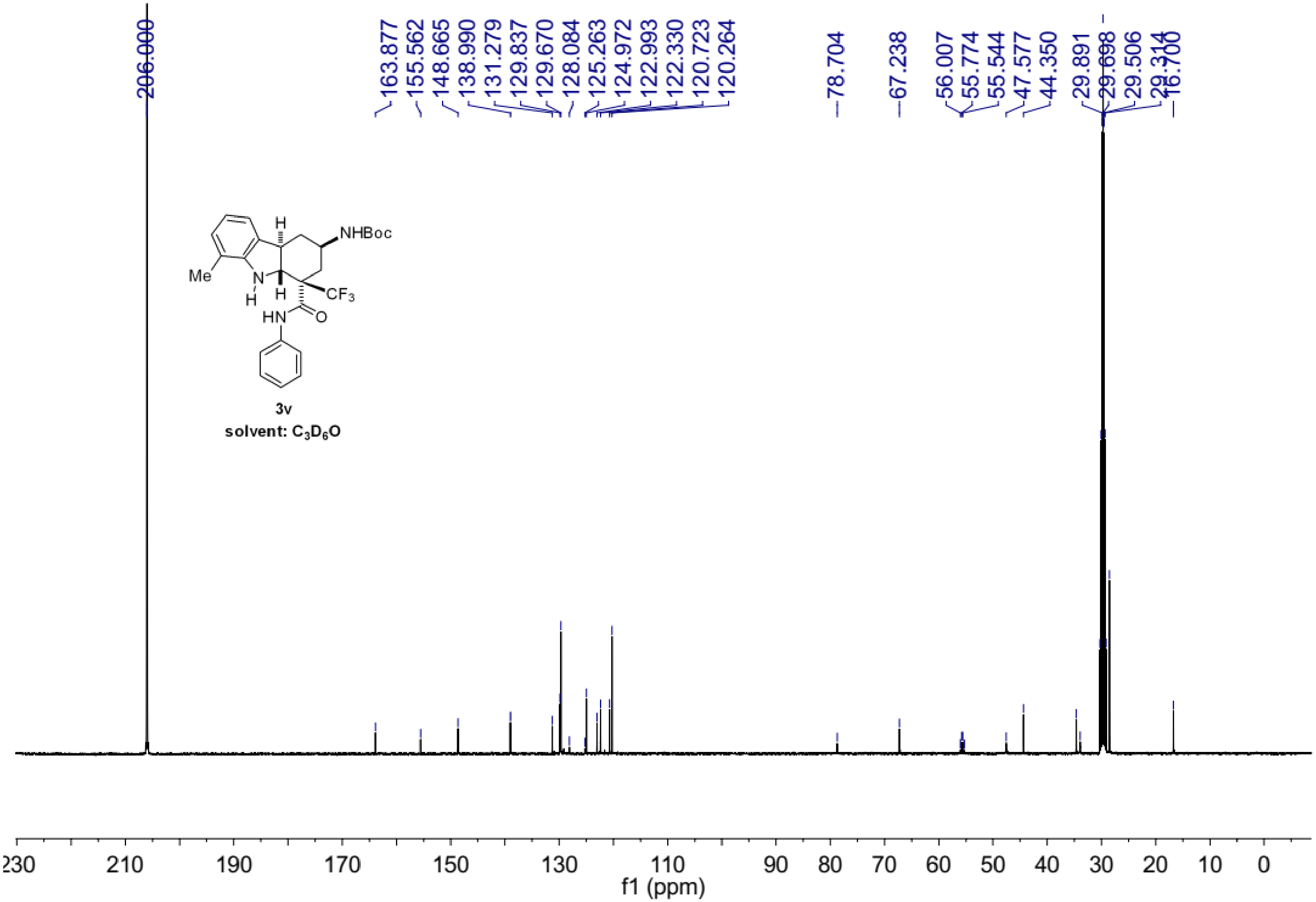

Supplementary Figure 96. ^{13}C NMR spectrum for compound **3v**

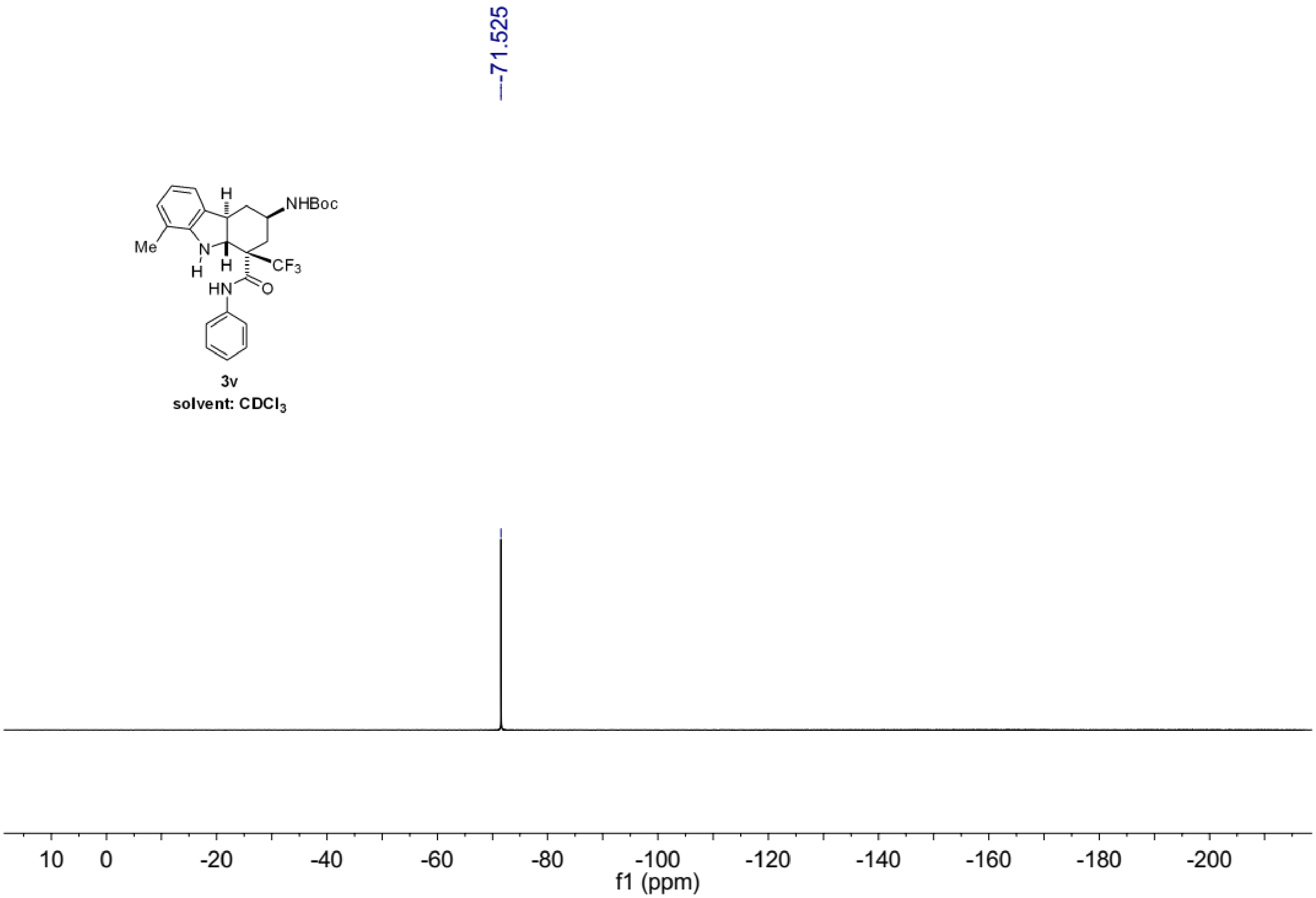

Supplementary Figure 97. ^{19}F NMR spectrum for compound **3v**

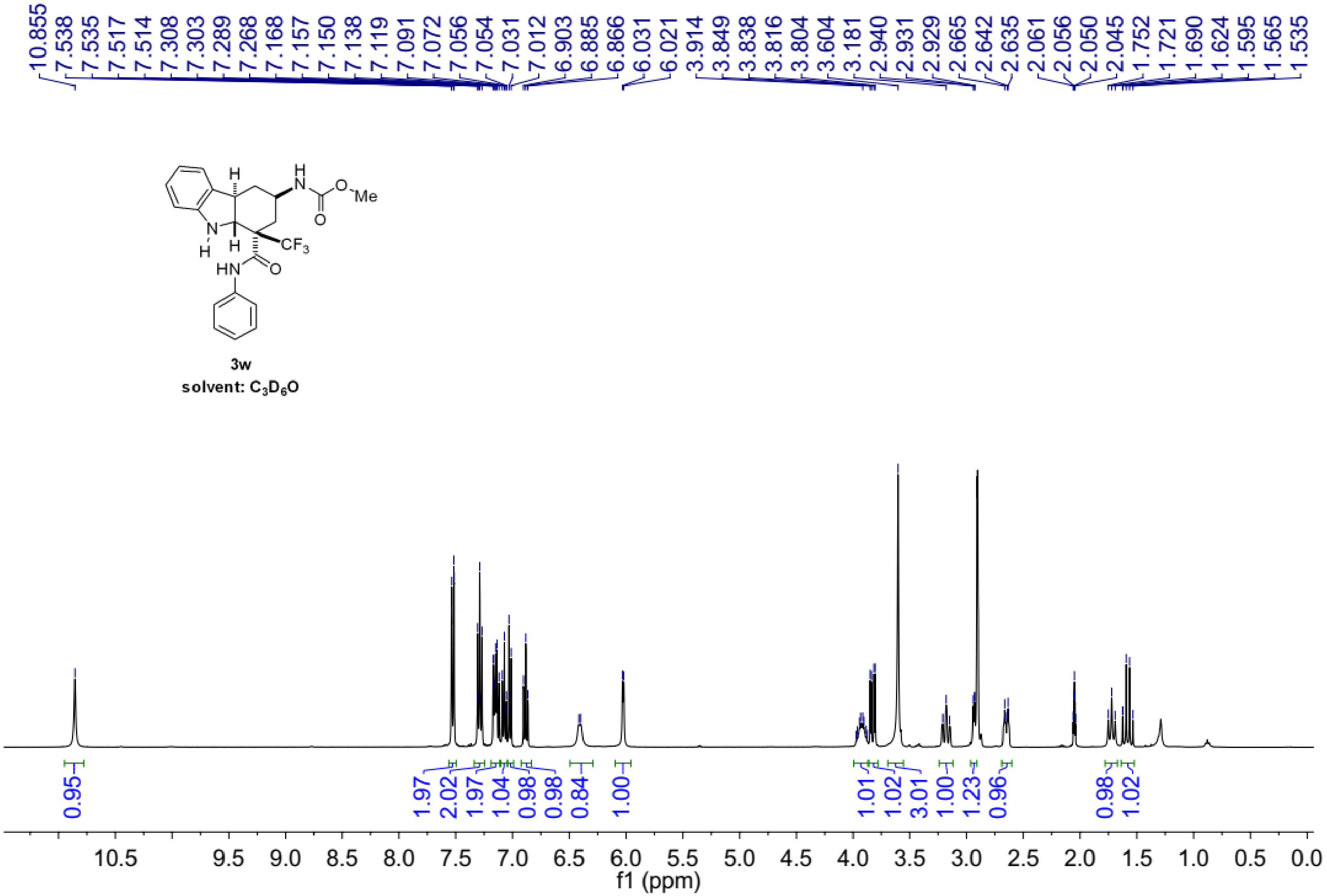

Supplementary Figure 98. ^{1}H NMR spectrum for compound **3w**

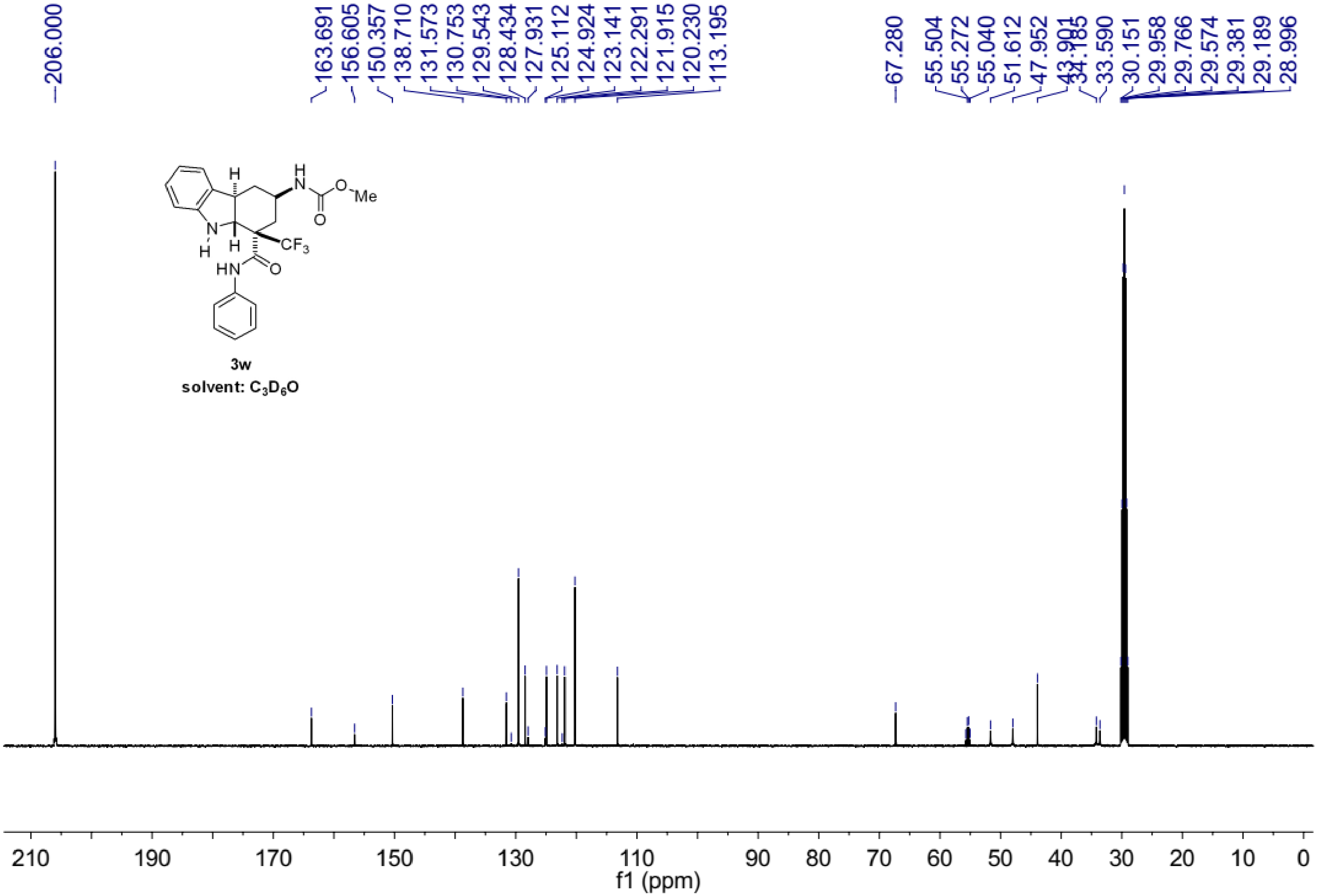

Supplementary Figure 99. ^{13}C NMR spectrum for compound **3w**

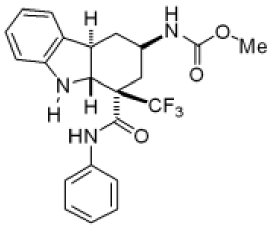

3w
solvent: $CDCl_3$

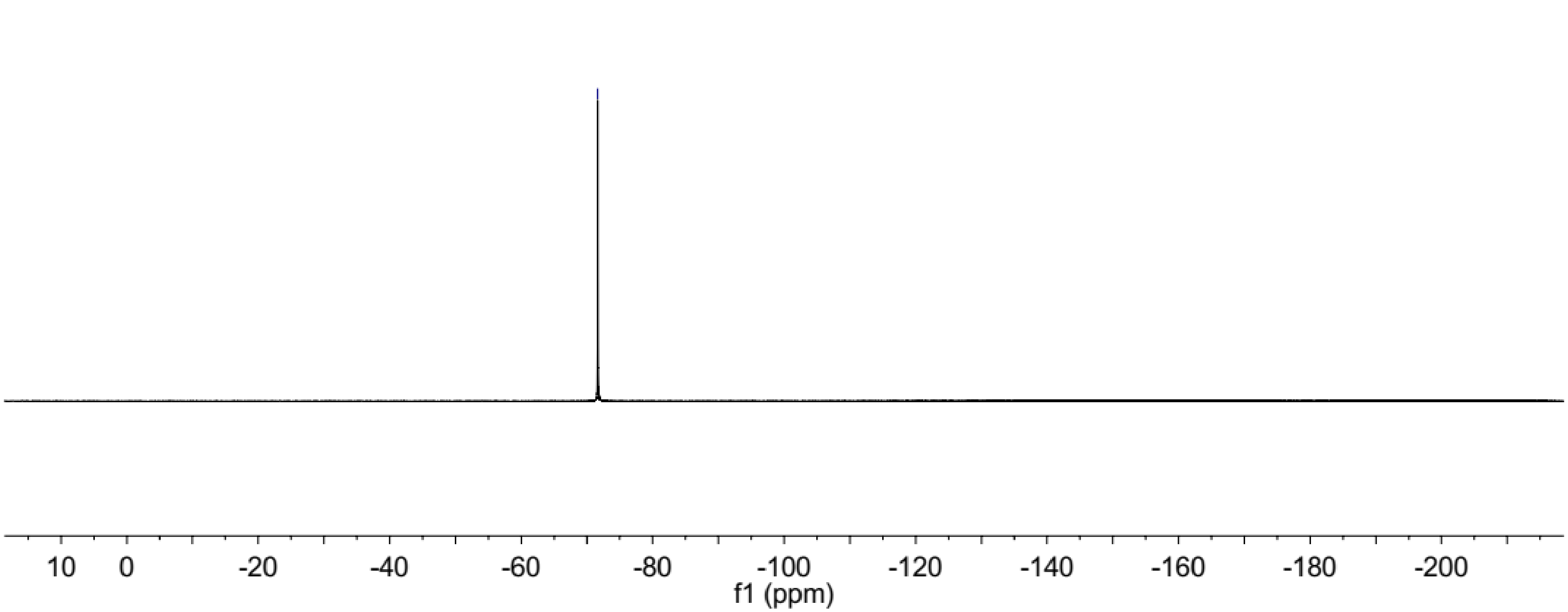

Supplementary Figure 100. ^{19}F NMR spectrum for compound **3w**

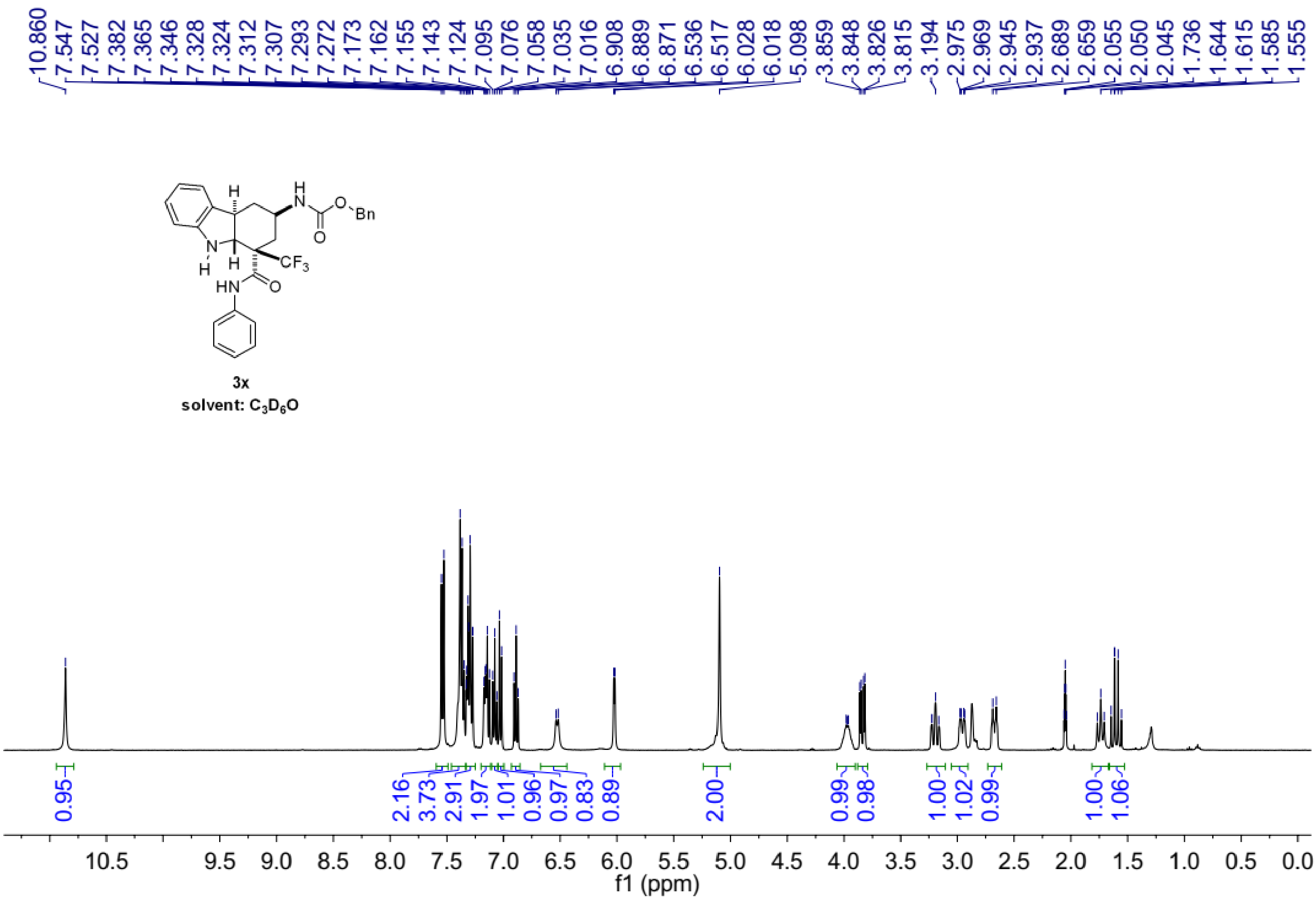

Supplementary Figure 101. ^{1}H NMR spectrum for compound **3x**

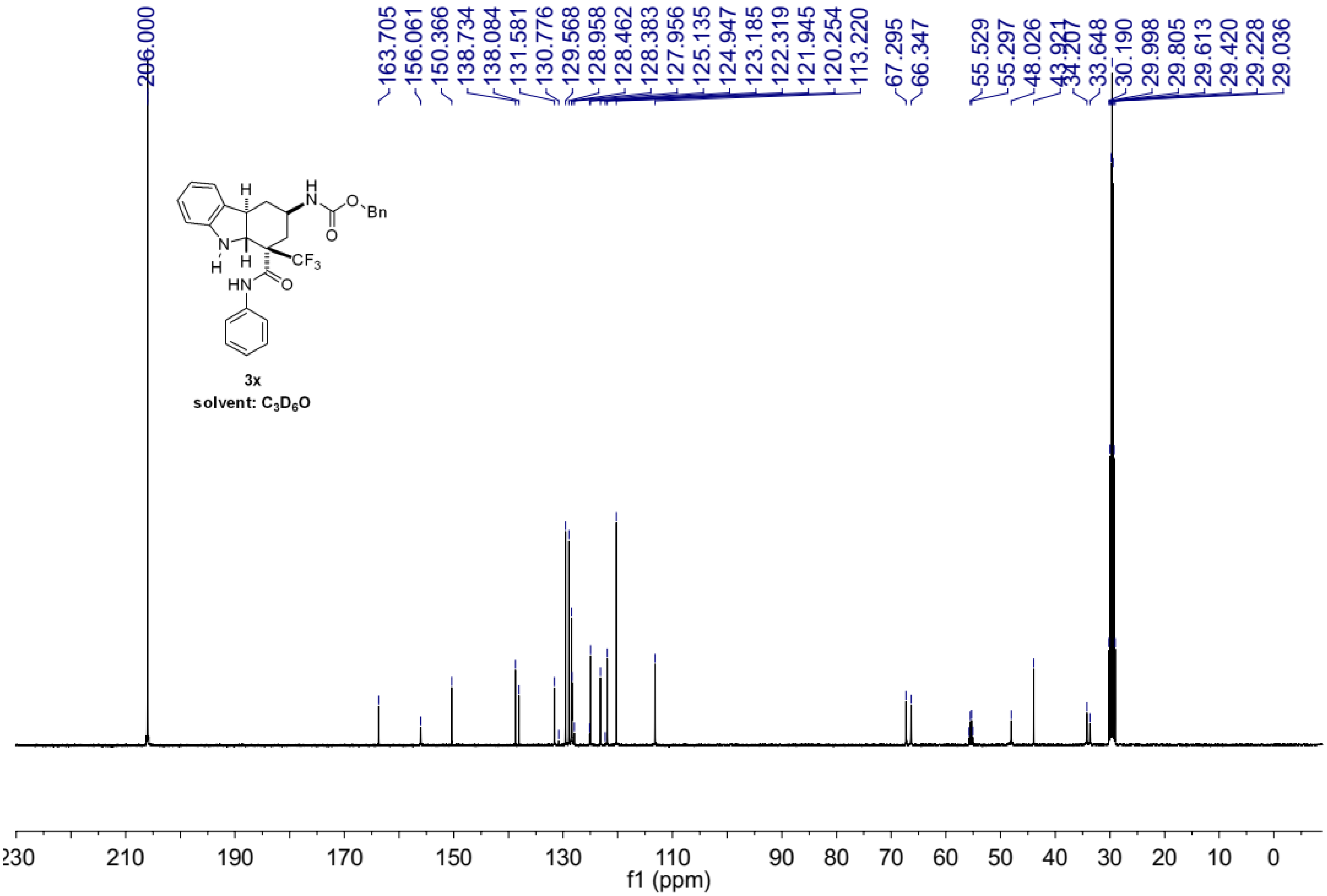

Supplementary Figure 102. ^{13}C NMR spectrum for compound **3x**

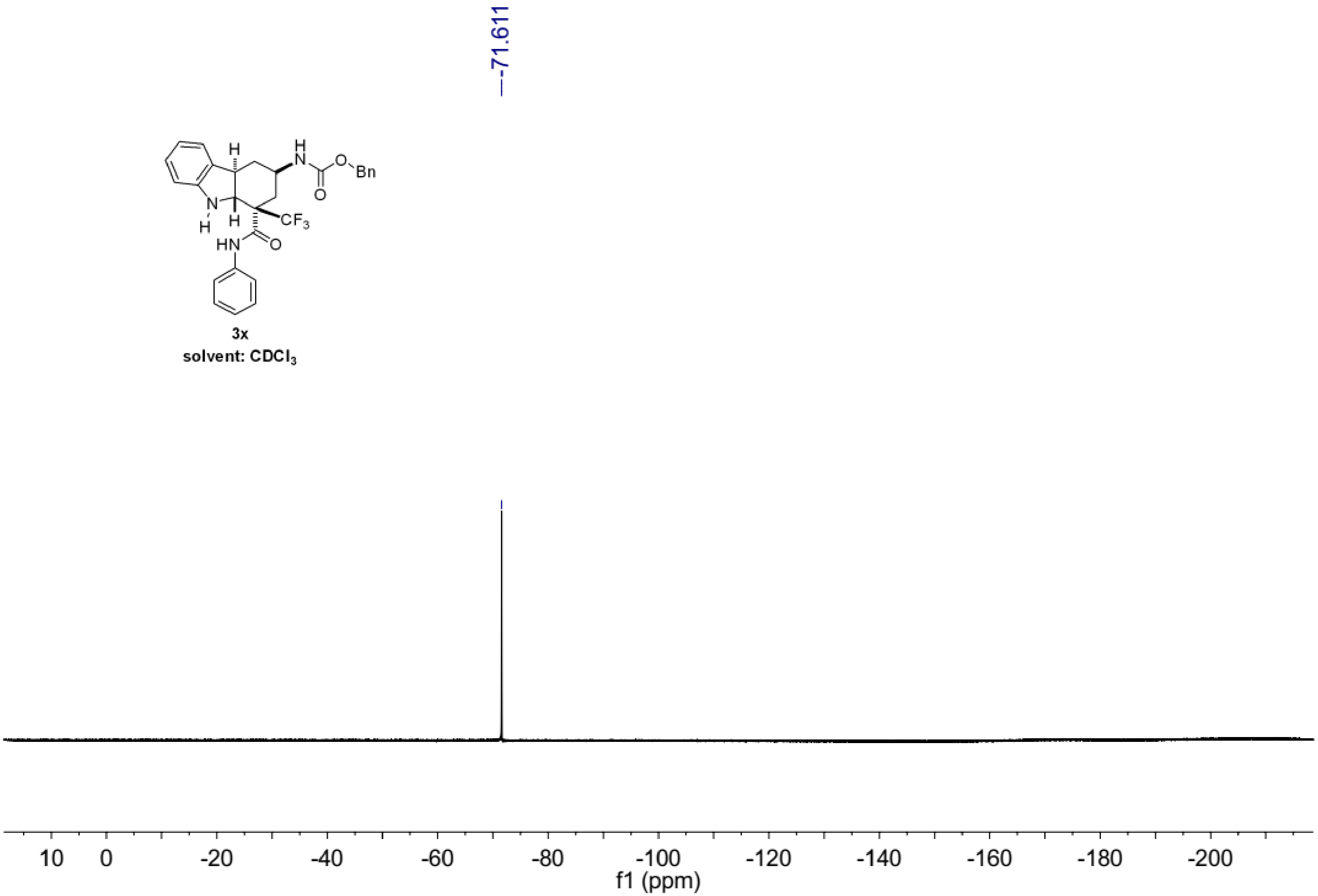

Supplementary Figure 103. ^{19}F NMR spectrum for compound **3x**

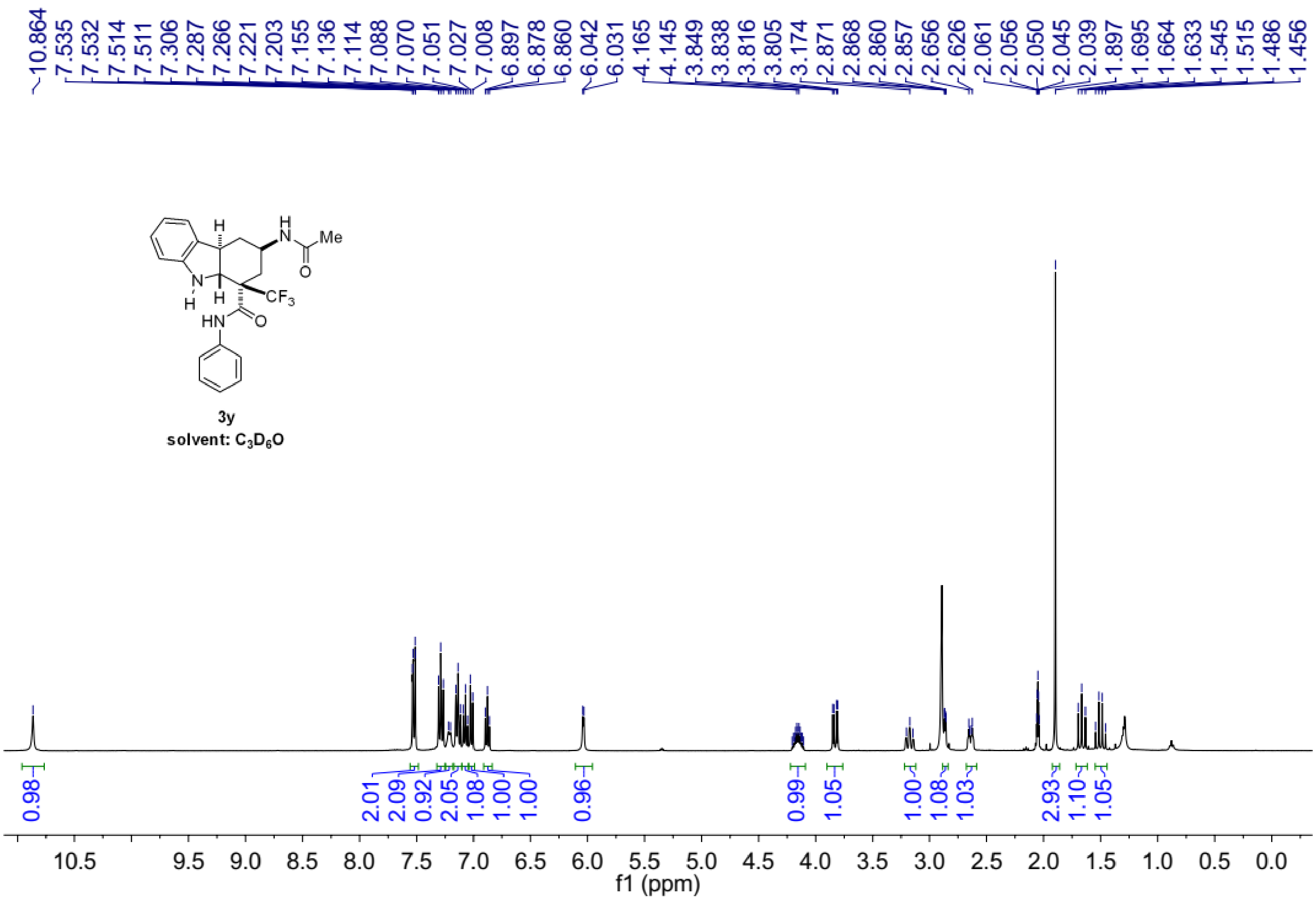

Supplementary Figure 104. ^{1}H NMR spectrum for compound **3y**

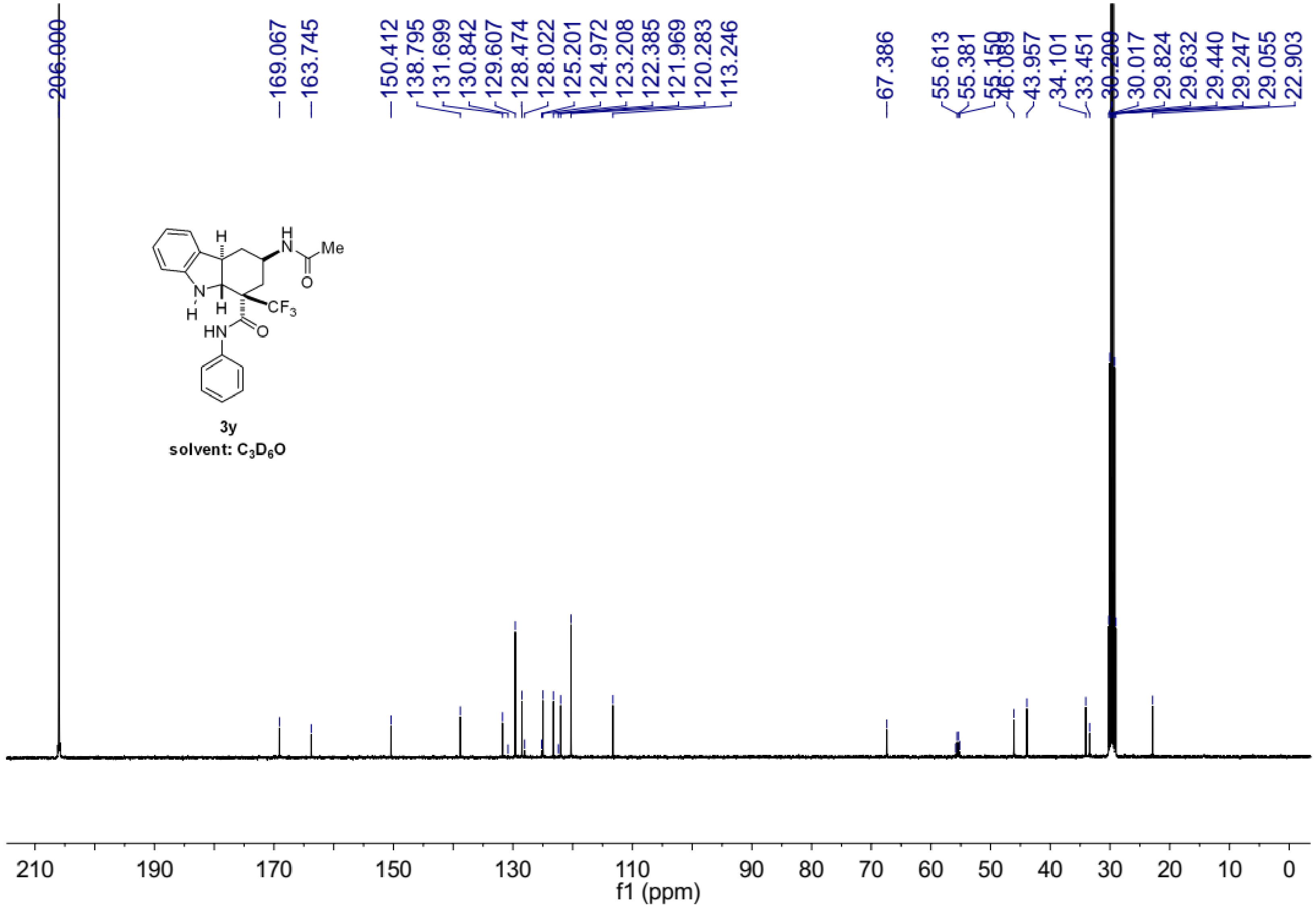

Supplementary Figure 105. ^{13}C NMR spectrum for compound **3y**

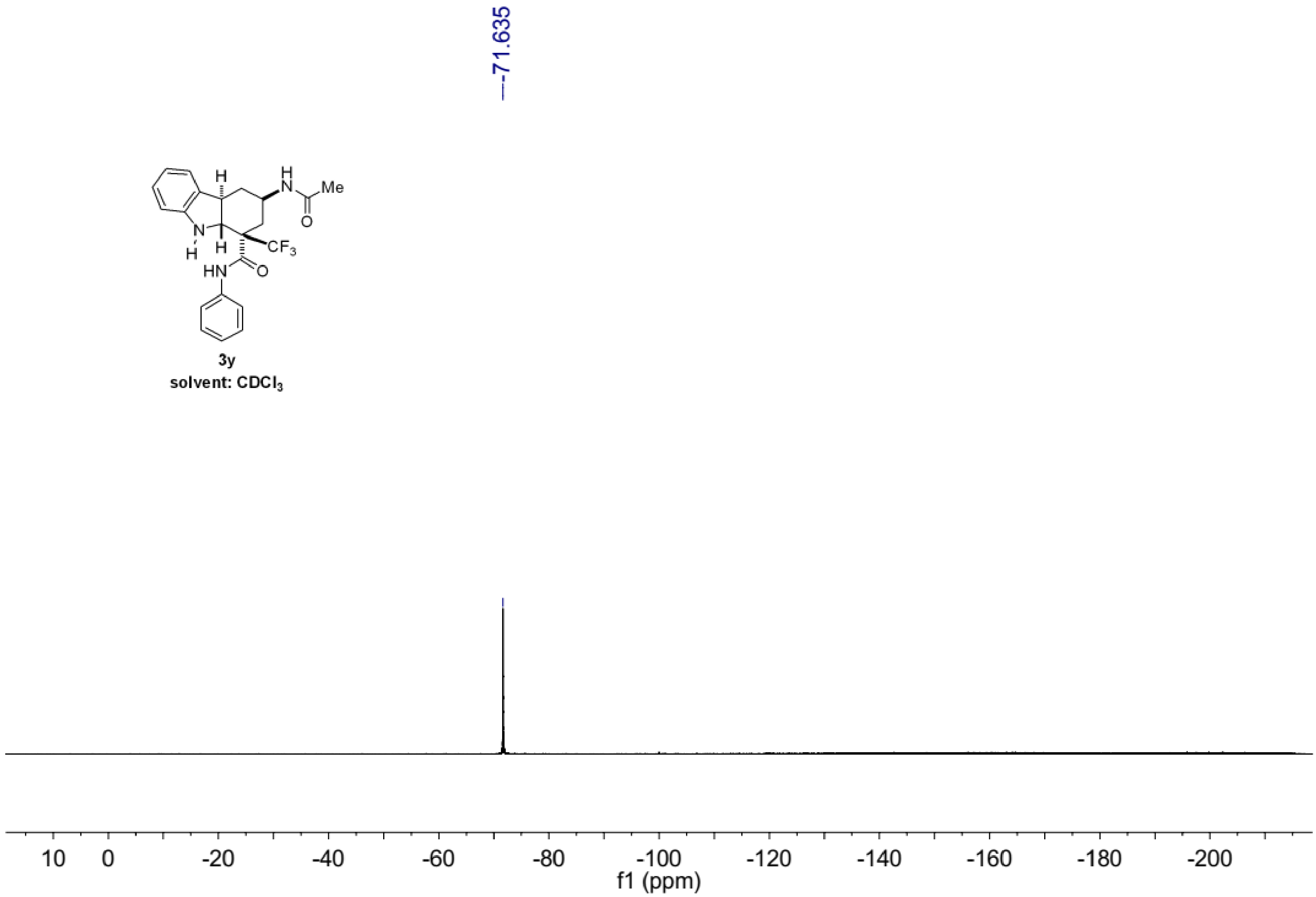

Supplementary Figure 106. ^{19}F NMR spectrum for compound **3y**

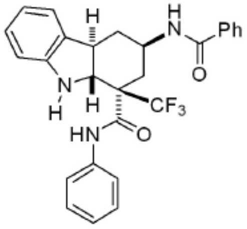

3z

solvent: C_3D_6O

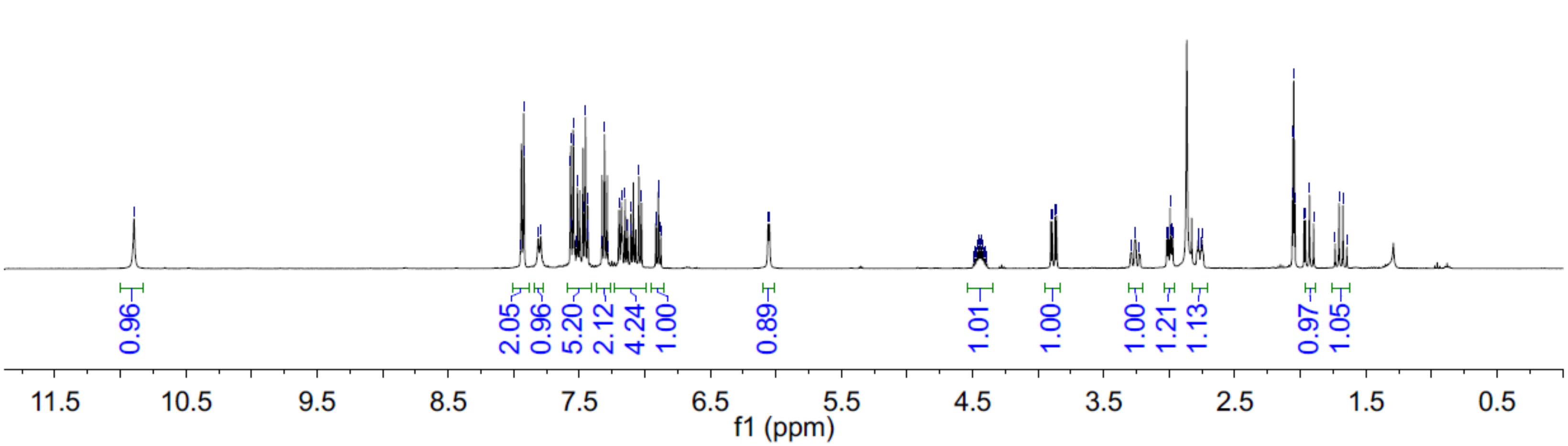

Supplementary Figure 107. ^{1}H NMR spectrum for compound **3z**

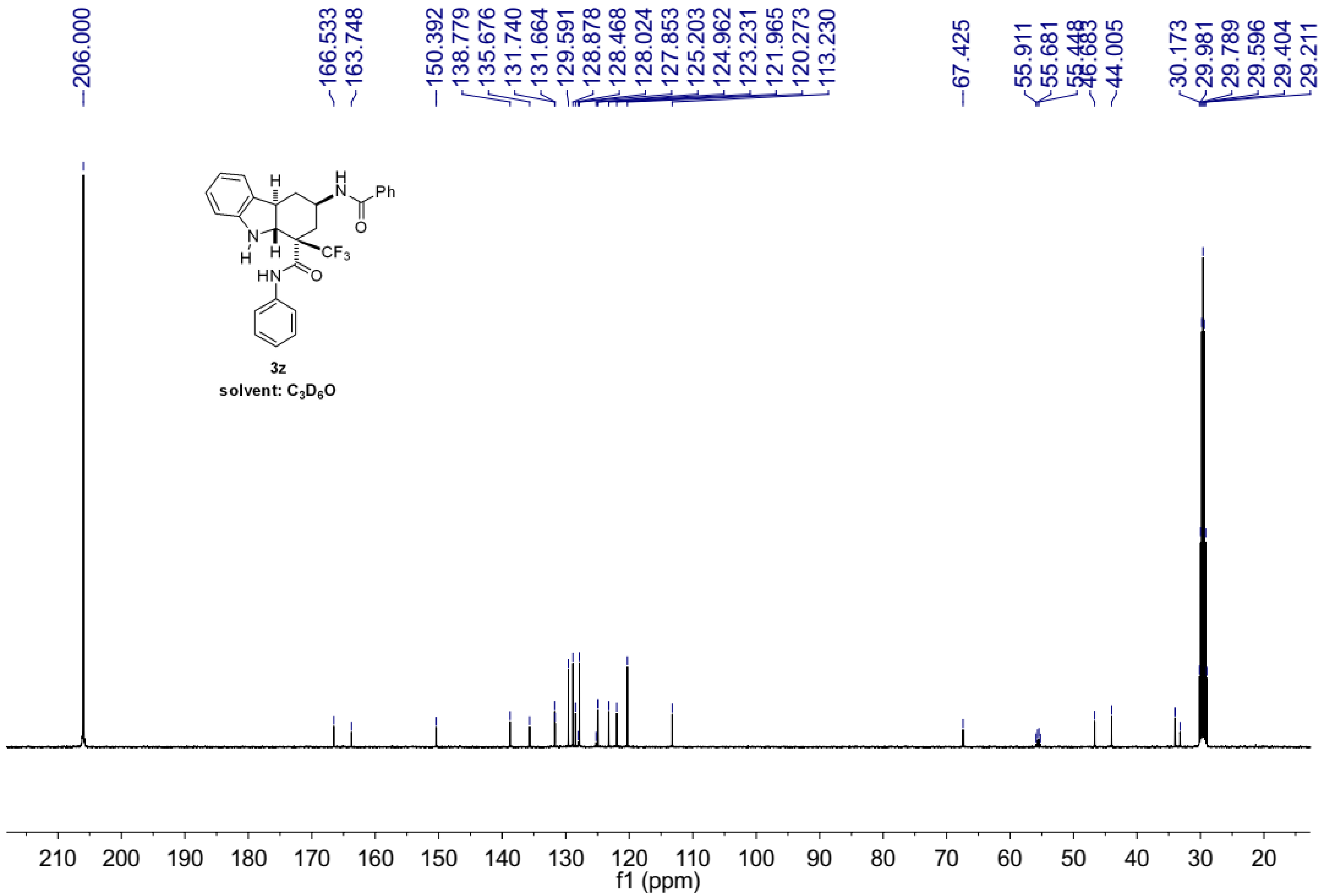

Supplementary Figure 108. ^{13}C NMR spectrum for compound **3z**

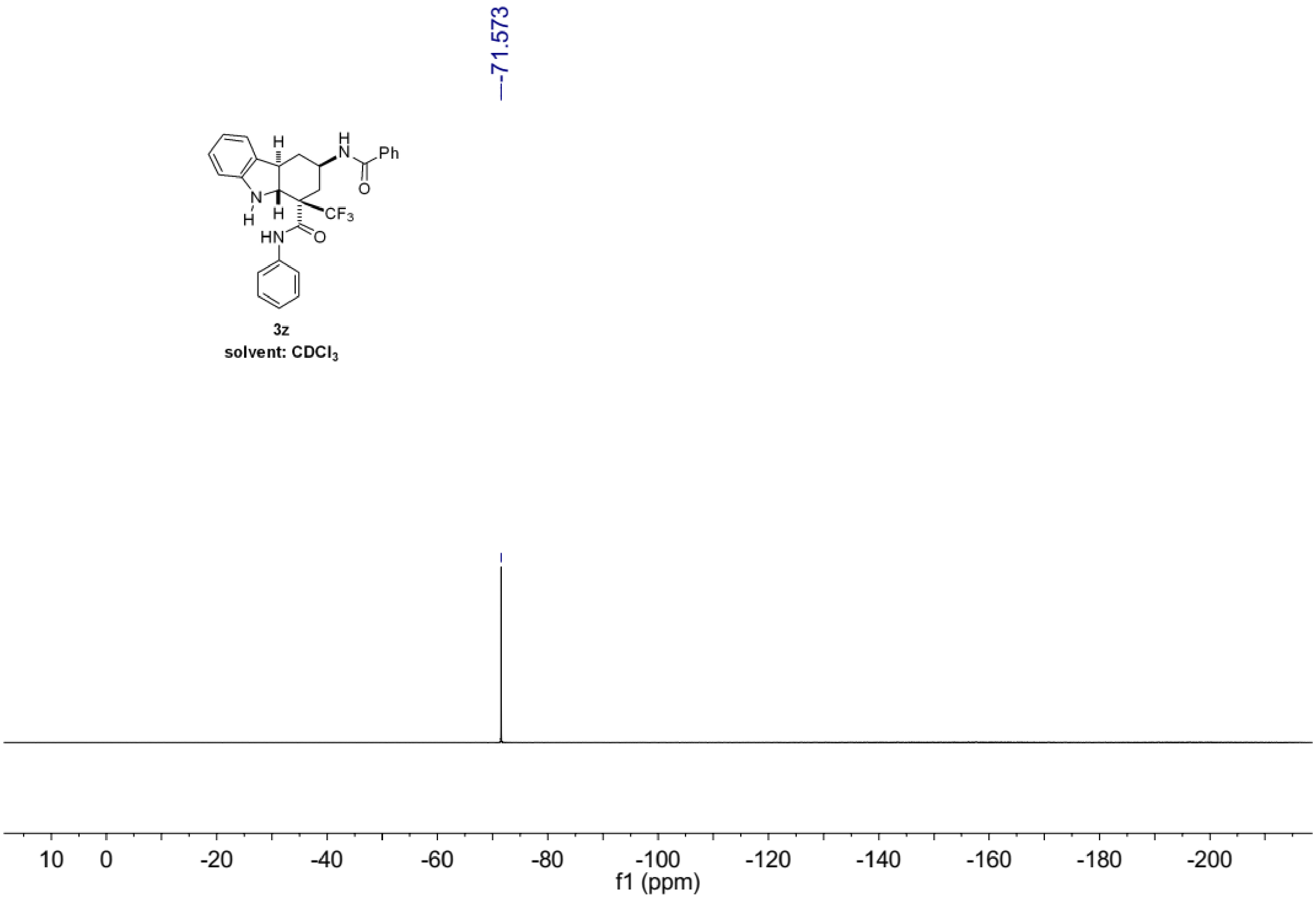

Supplementary Figure 109. ^{19}F NMR spectrum for compound **3z**

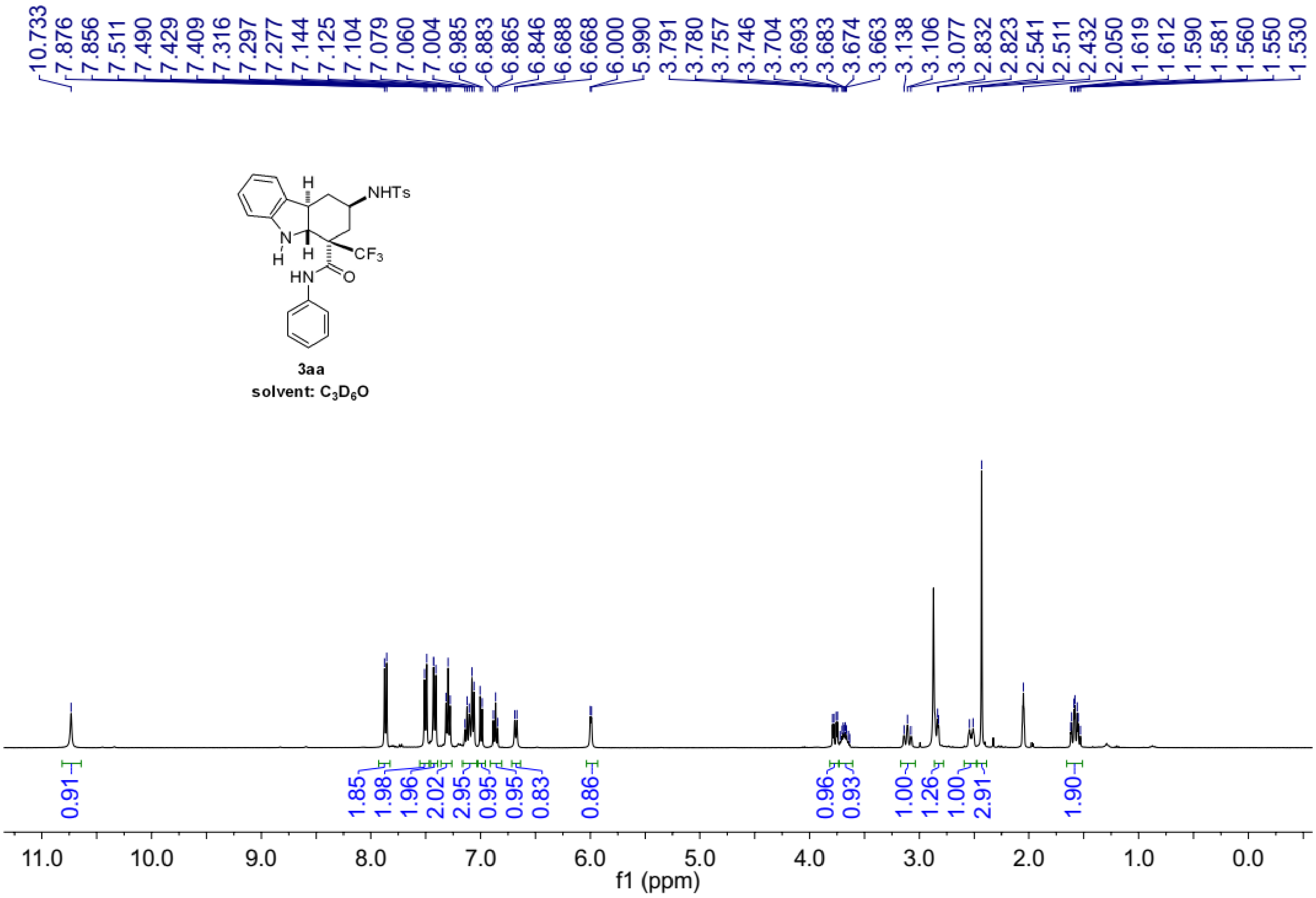

Supplementary Figure 110. ^{1}H NMR spectrum for compound **3aa**

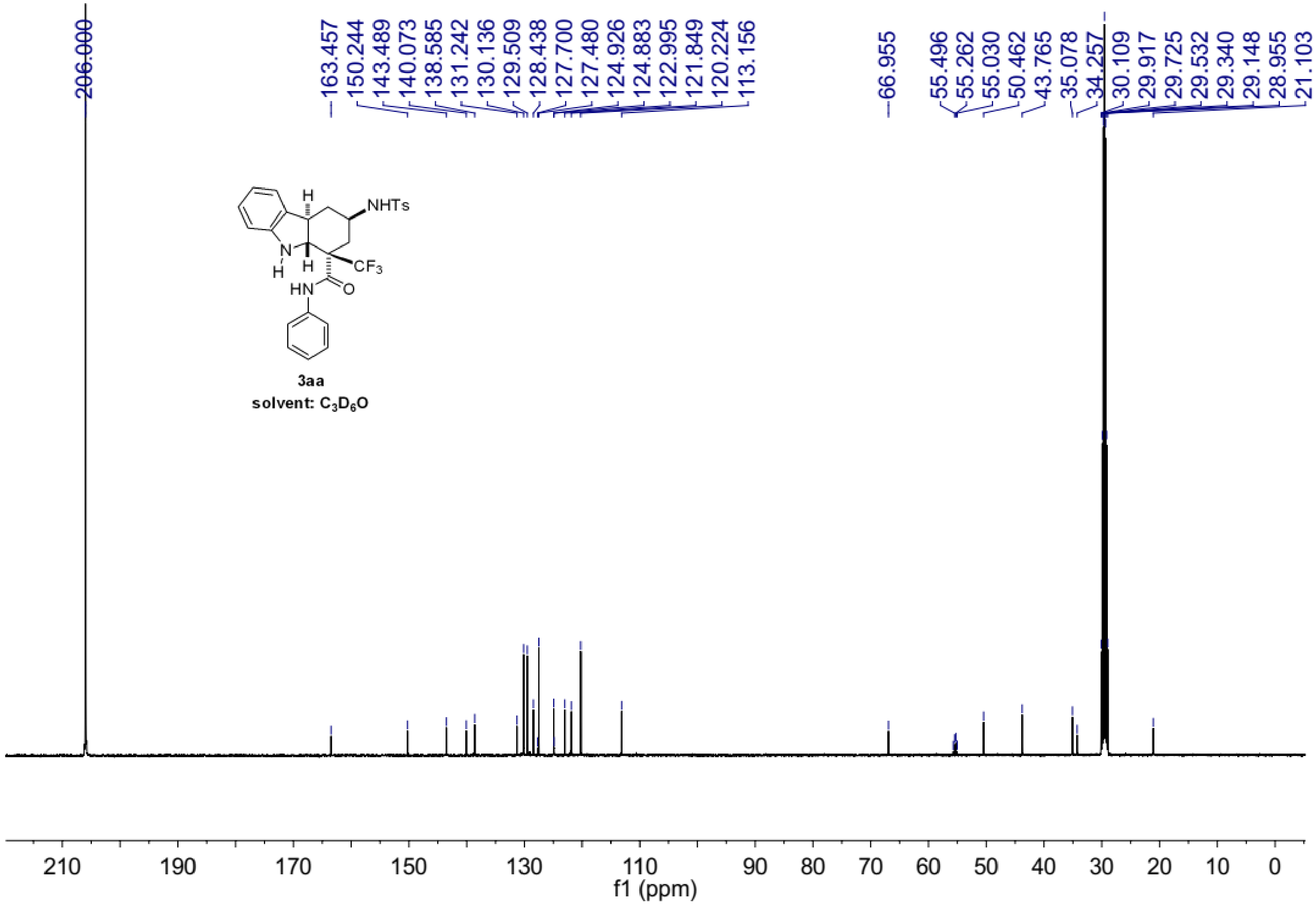

Supplementary Figure 111. ^{13}C NMR spectrum for compound **3aa**

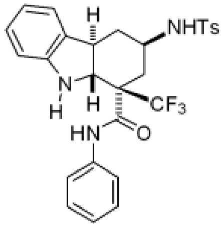

3aa
solvent: $CDCl_3$

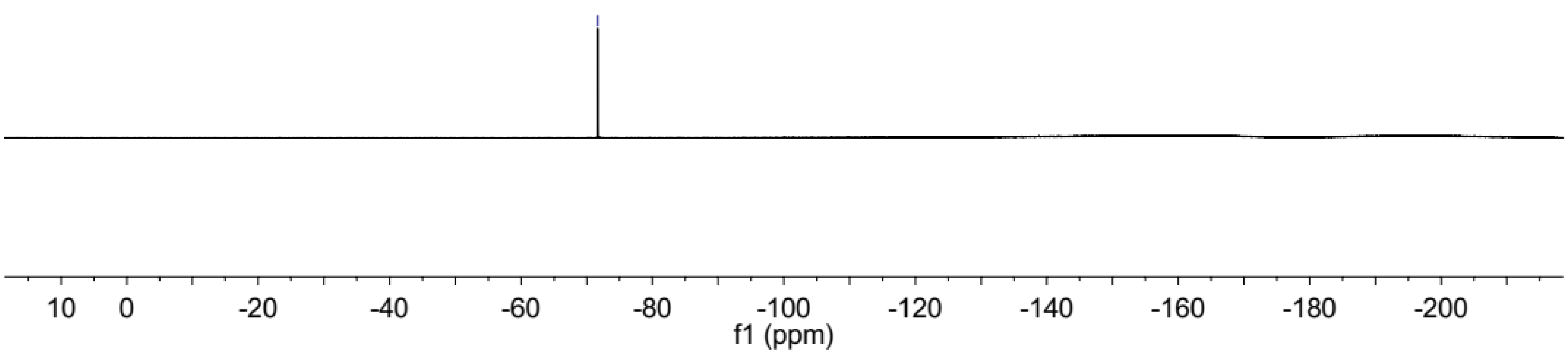

Supplementary Figure 112. ^{19}F NMR spectrum for compound **3aa**

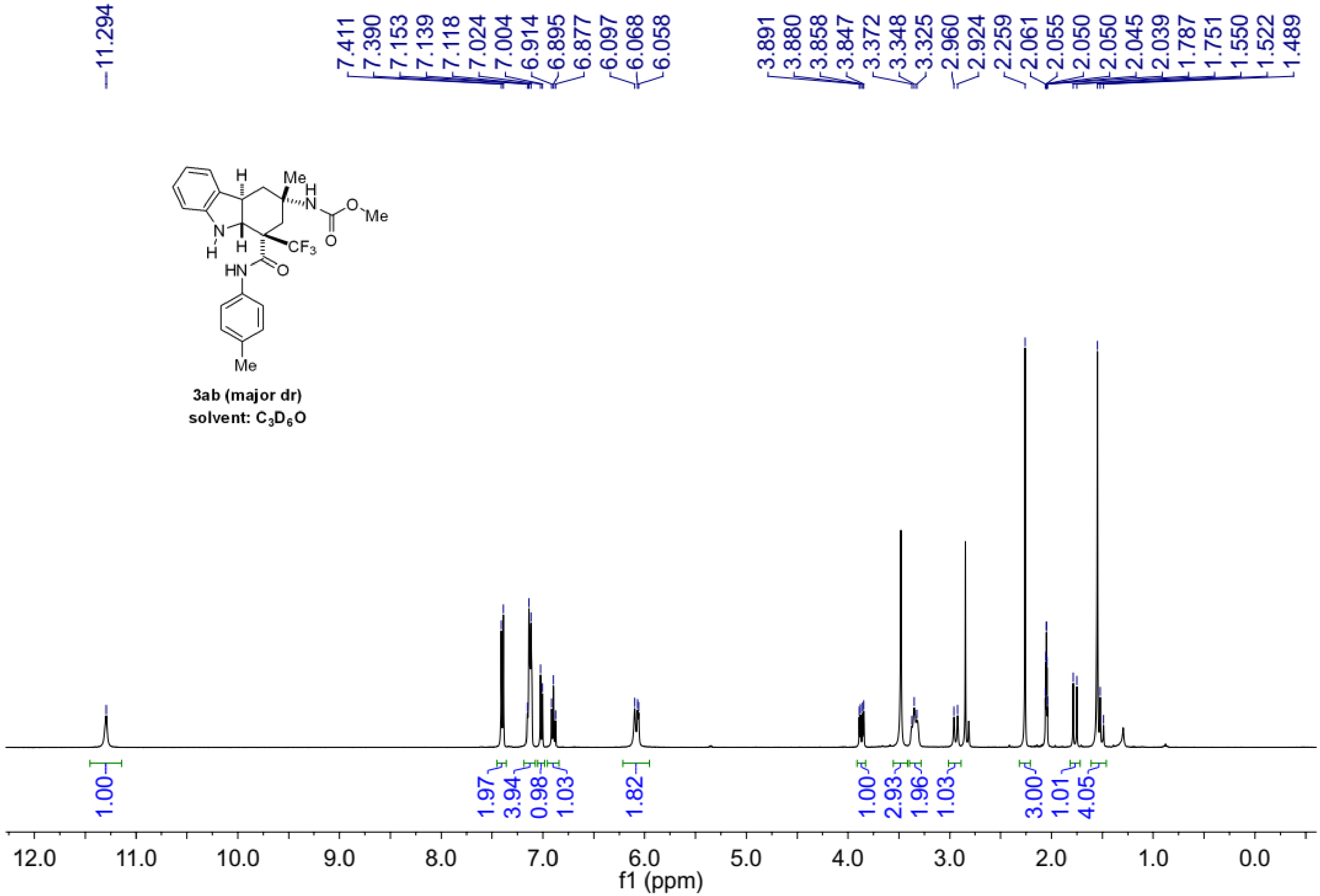

Supplementary Figure 113. ^{1}H NMR spectrum for compound **3ab**

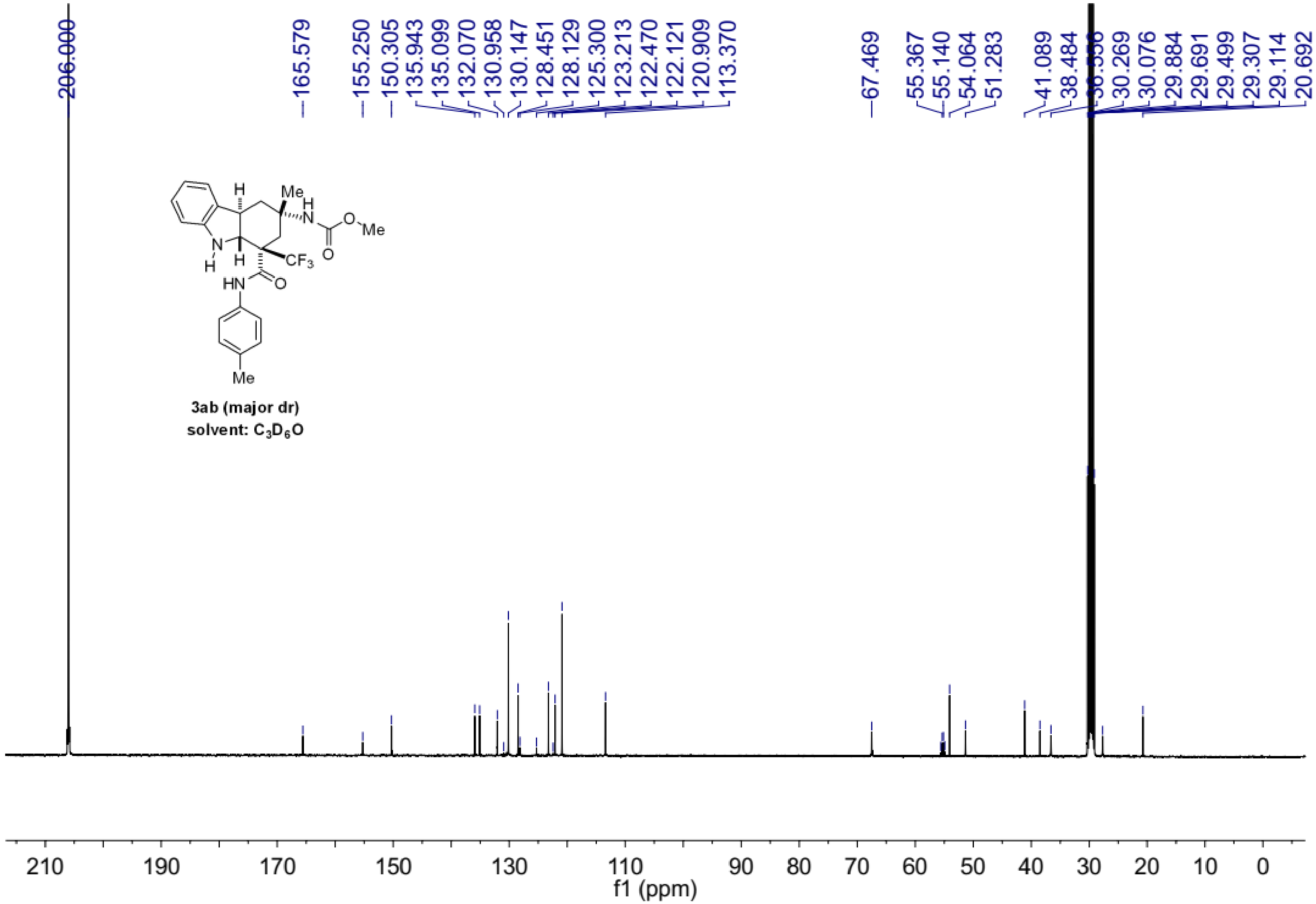

Supplementary Figure 114. ^{13}C NMR spectrum for compound **3ab**

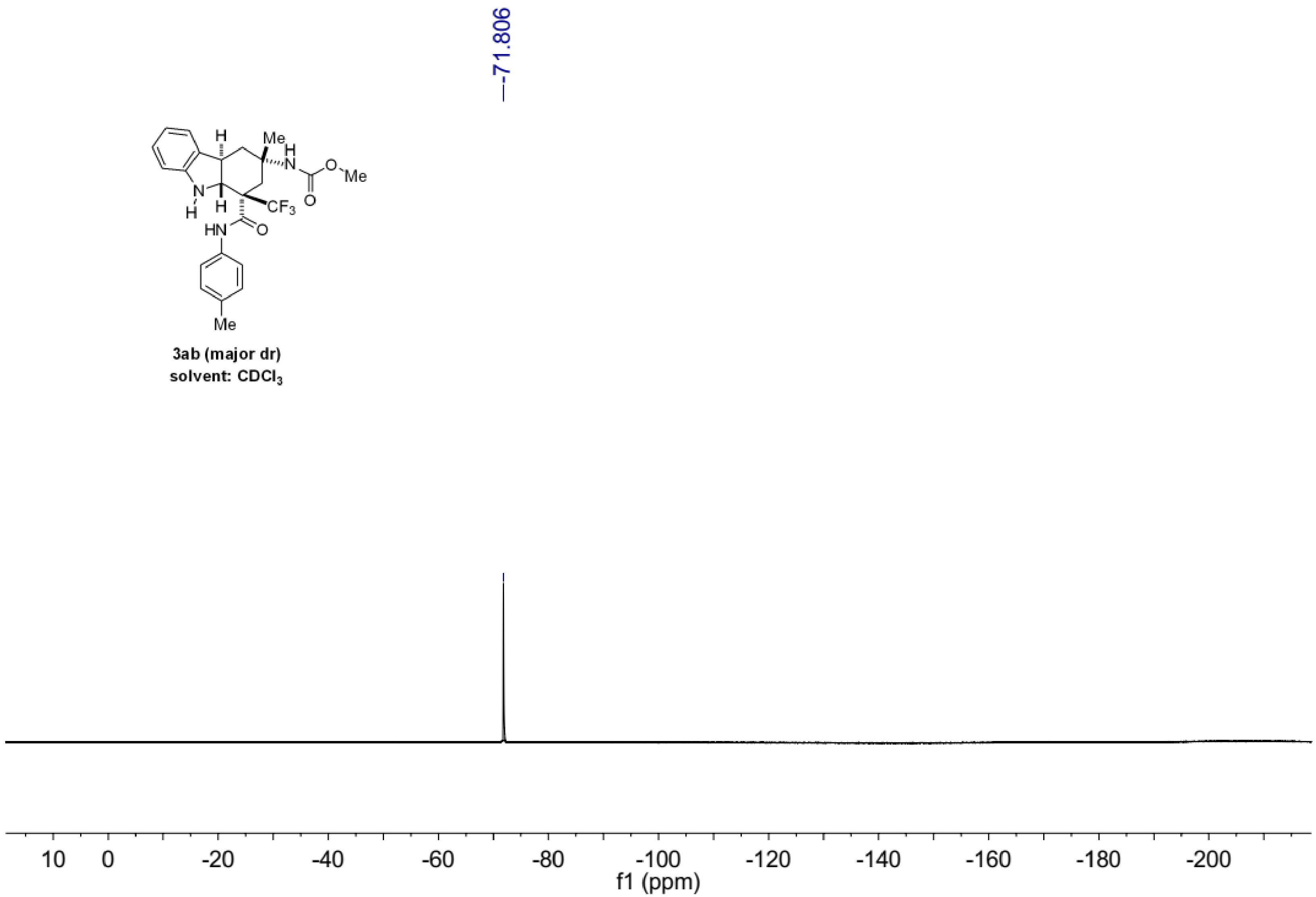

Supplementary Figure 115. ^{19}F NMR spectrum for compound **3ab**

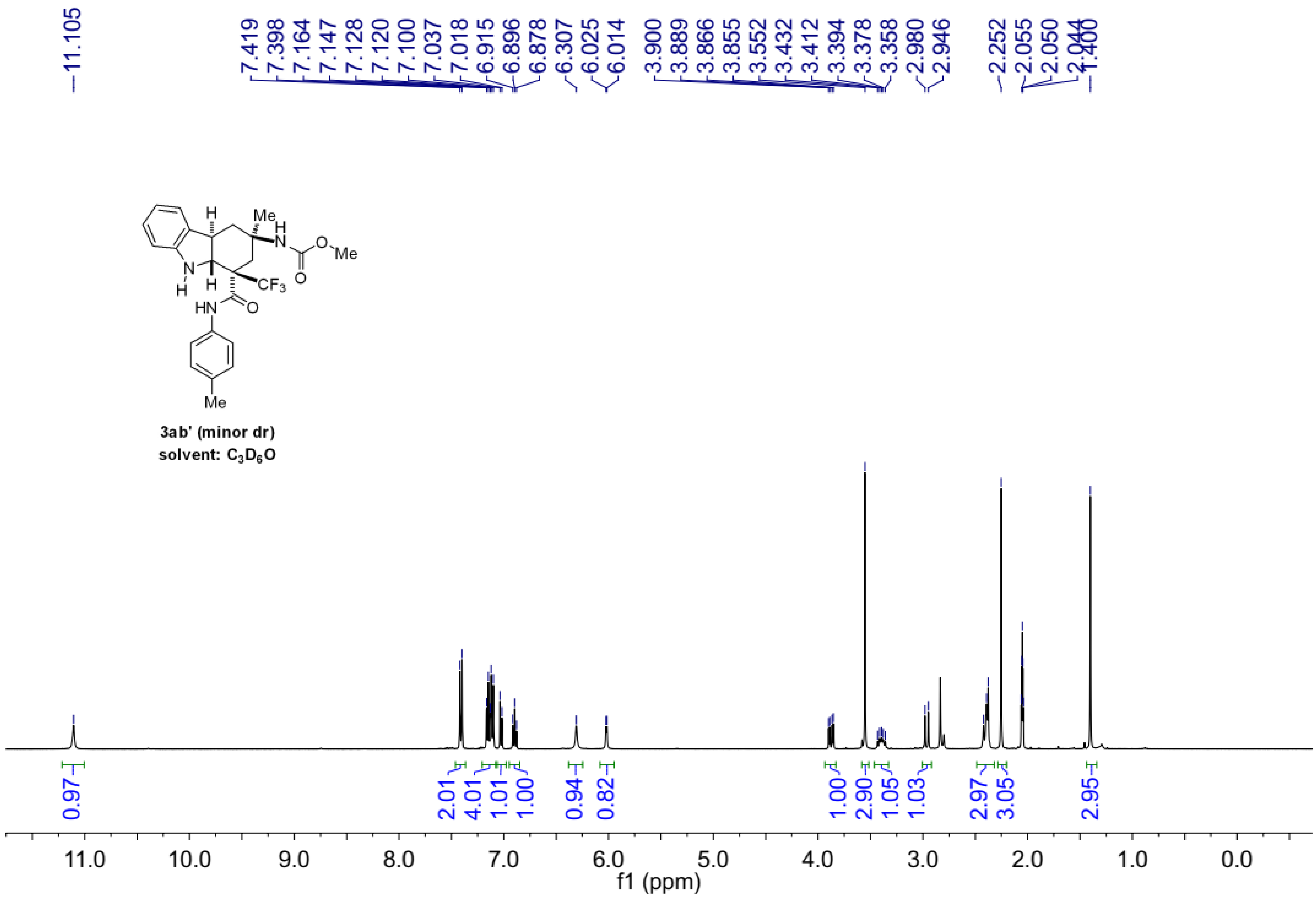

Supplementary Figure 116. ^{1}H NMR spectrum for compound **3ab’**

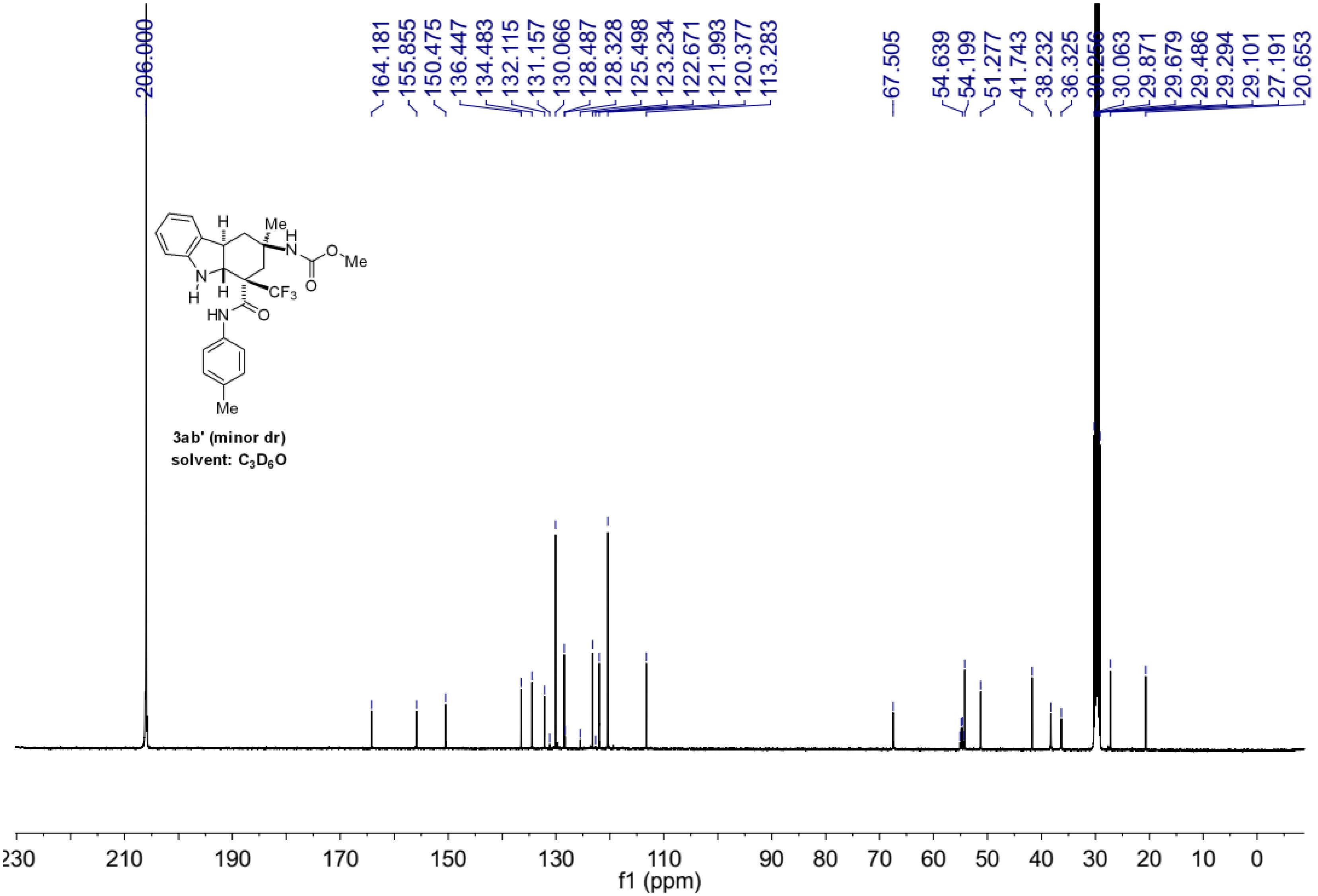

Supplementary Figure 117. ^{13}C NMR spectrum for compound **3ab’**

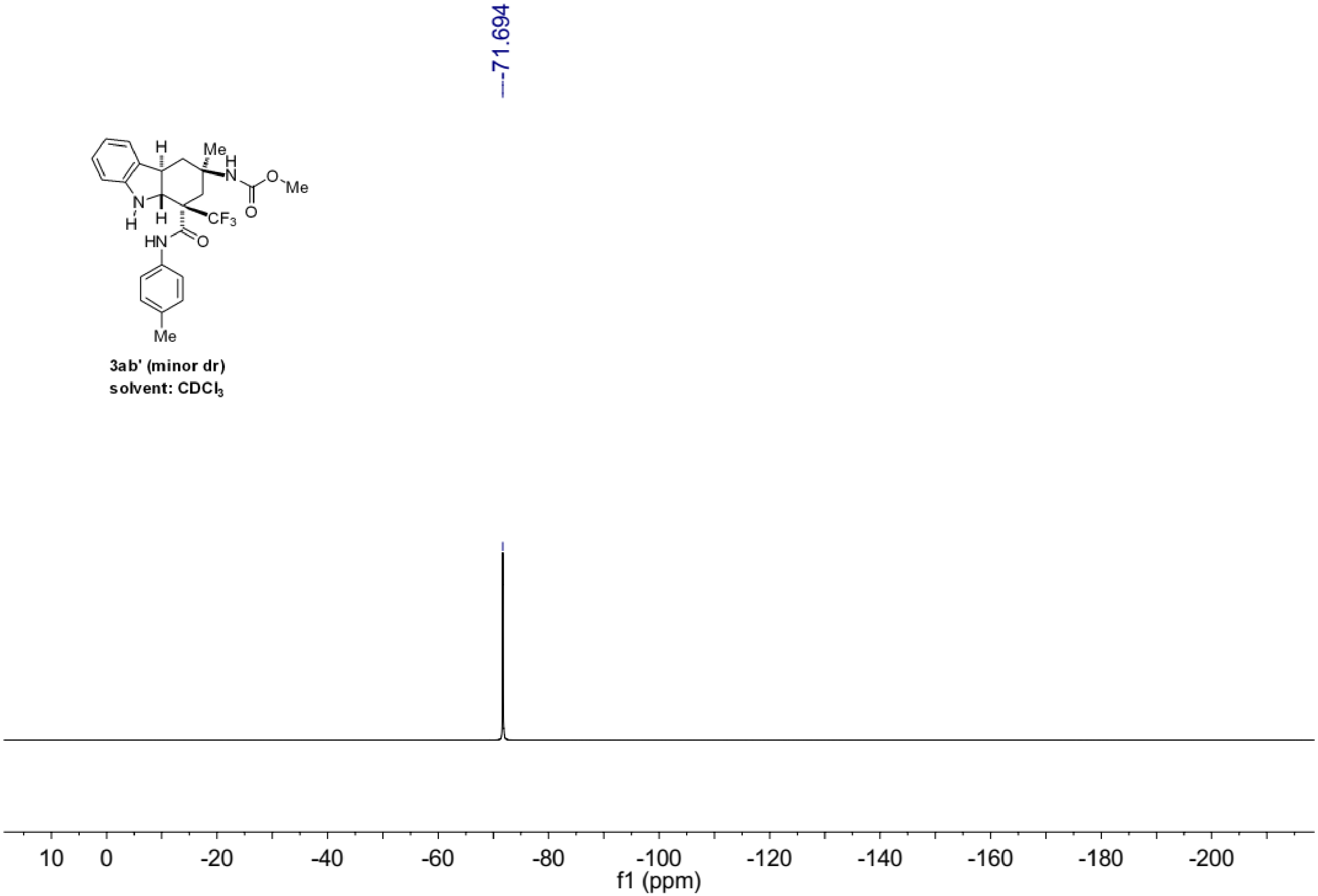

Supplementary Figure 118. ^{19}F NMR spectrum for compound **3ab'**

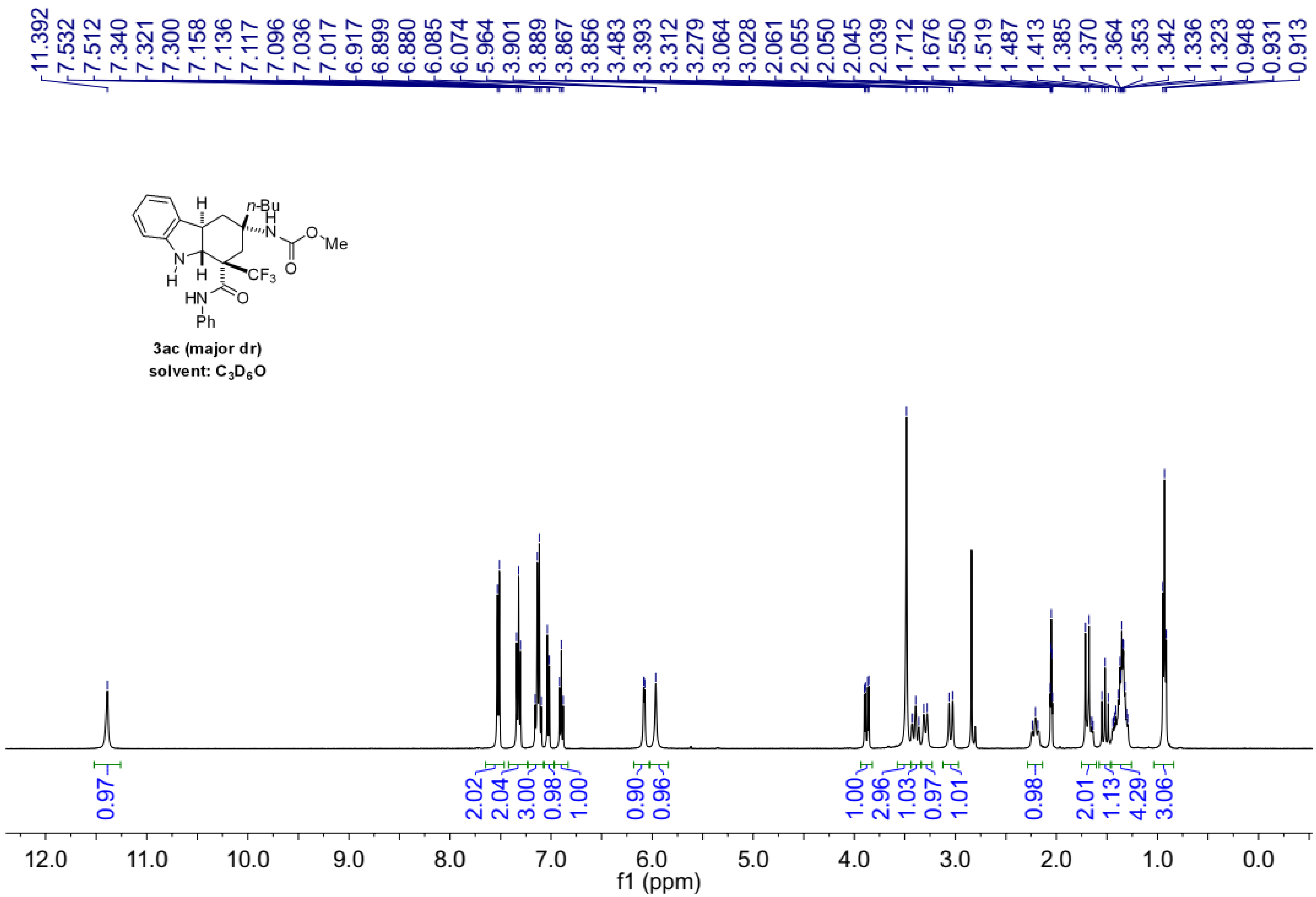

Supplementary Figure 119. ^{1}H NMR spectrum for compound **3ac**

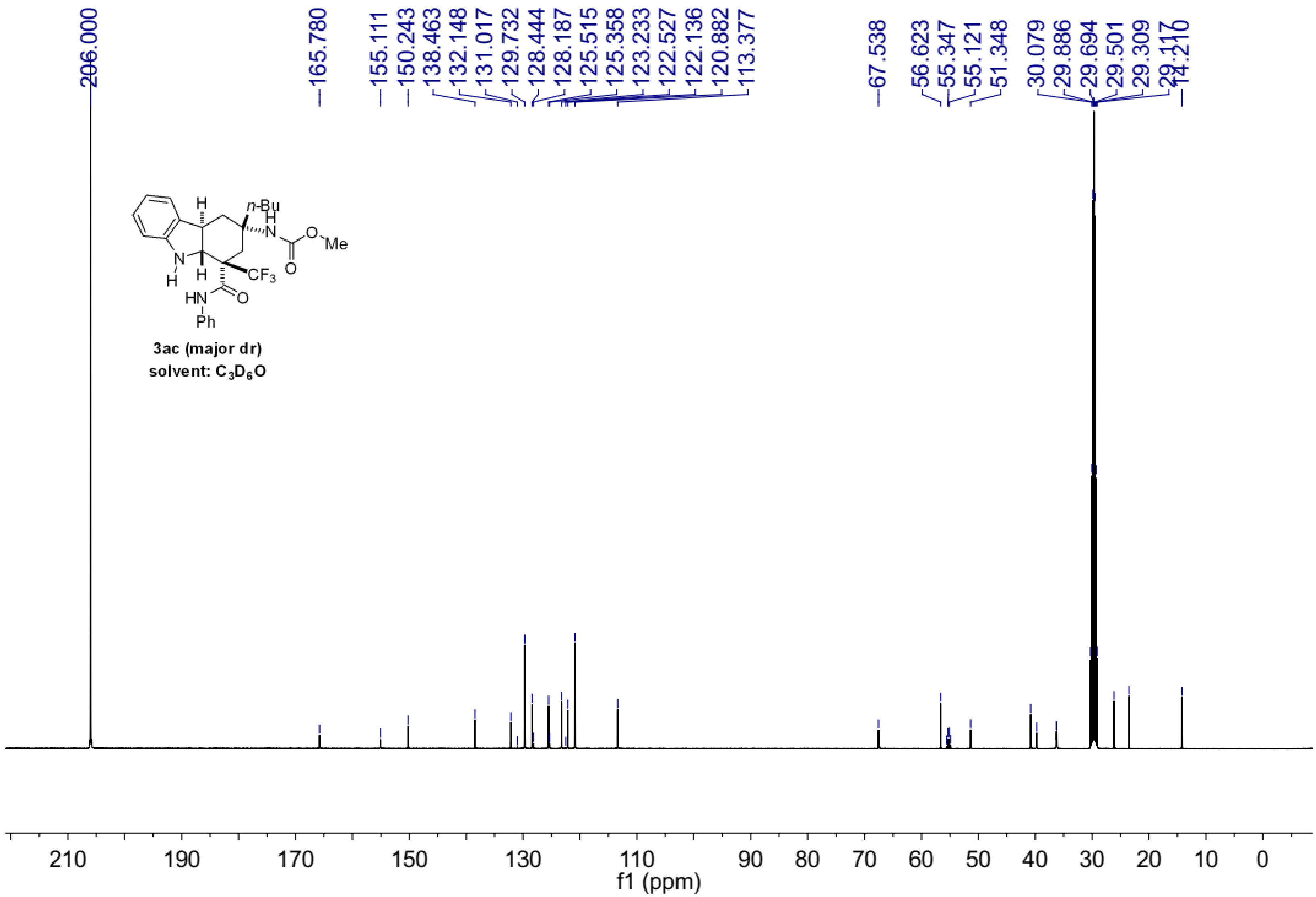

Supplementary Figure 120. ^{13}C NMR spectrum for compound **3ac**

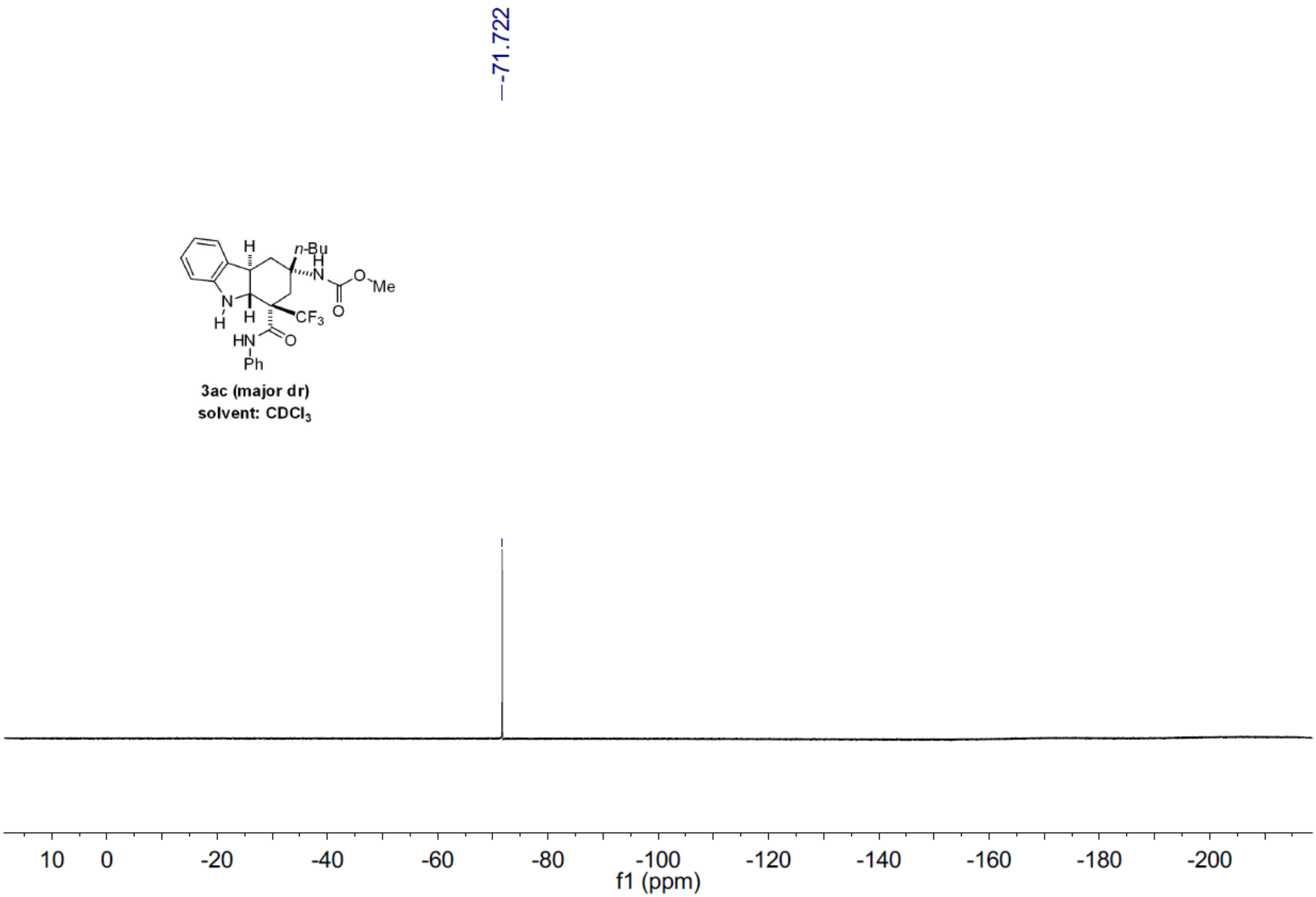

Supplementary Figure 121. ^{19}F NMR spectrum for compound **3ac**

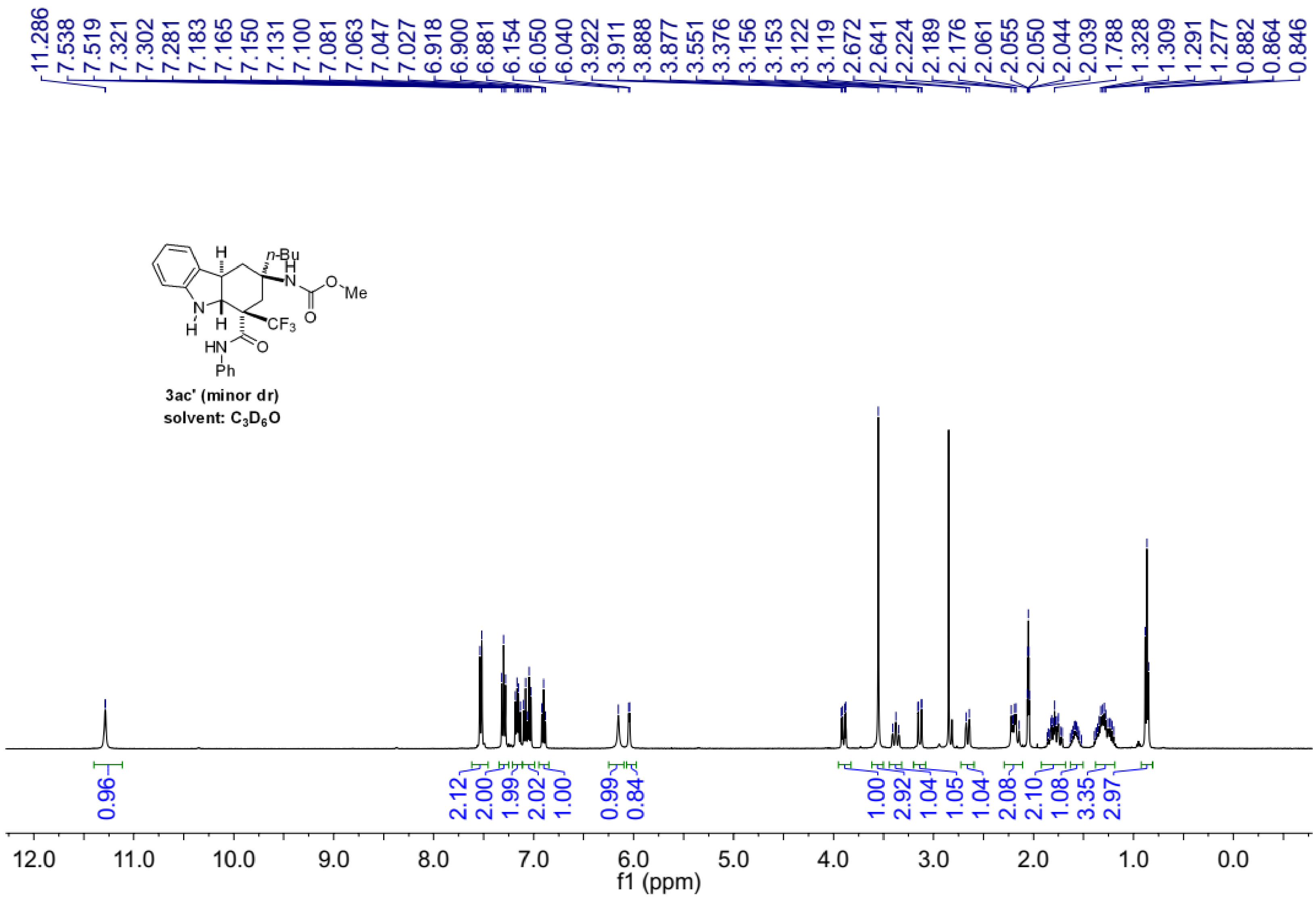

Supplementary Figure 122. ^{1}H NMR spectrum for compound **3ac'**

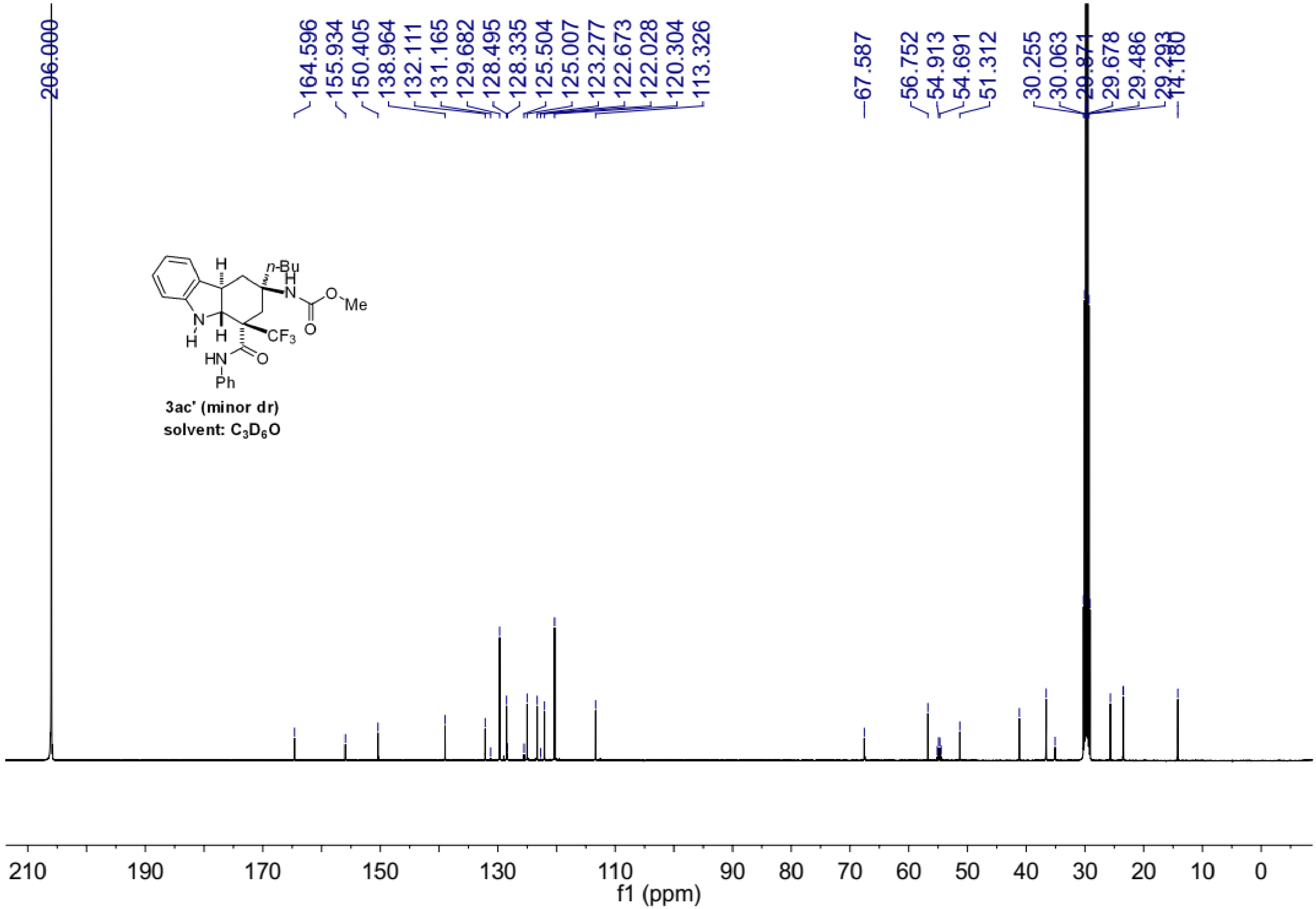

Supplementary Figure 123. ^{13}C NMR spectrum for compound **3ac'**

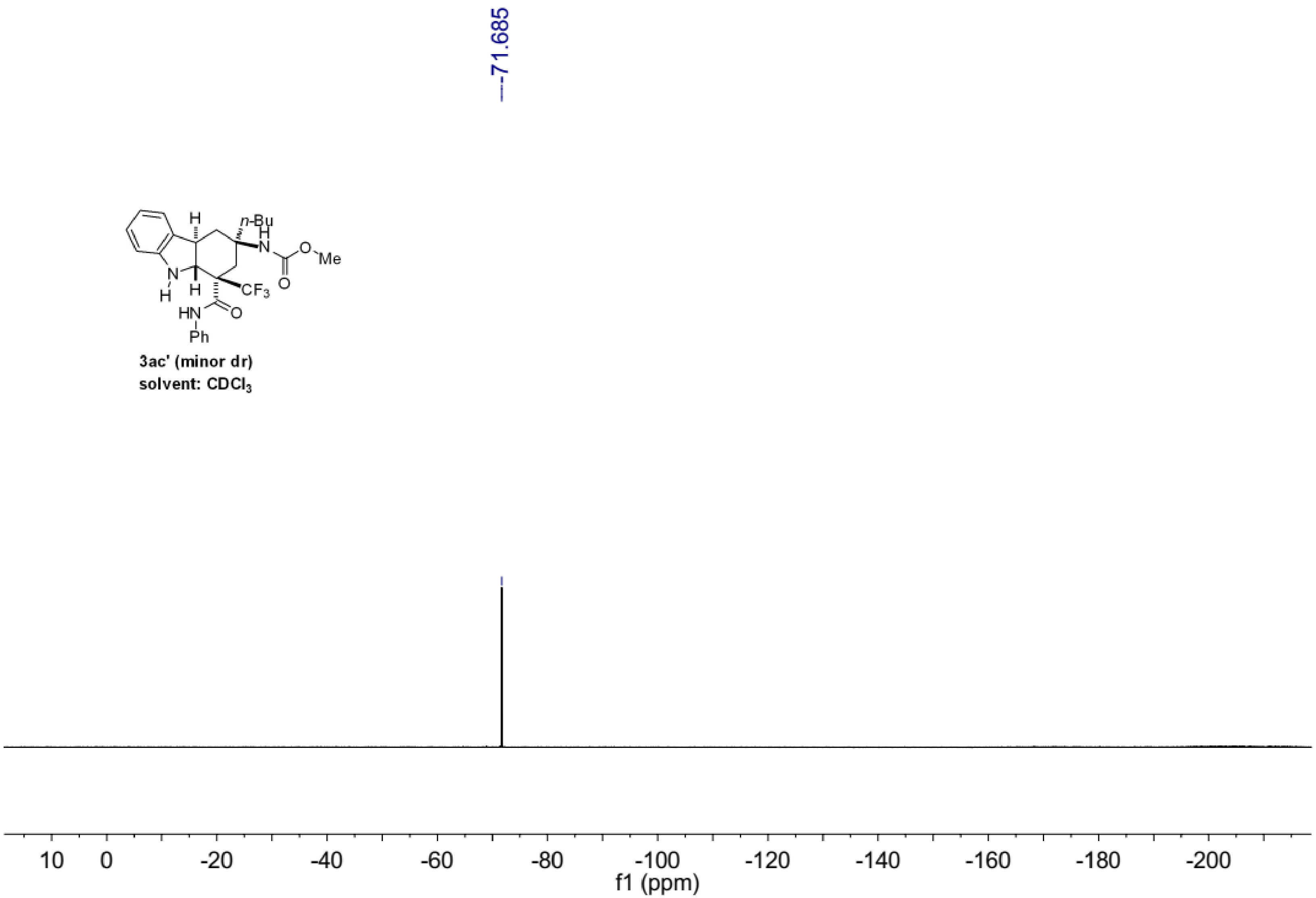

Supplementary Figure 124. ^{19}F NMR spectrum for compound **3ac'**

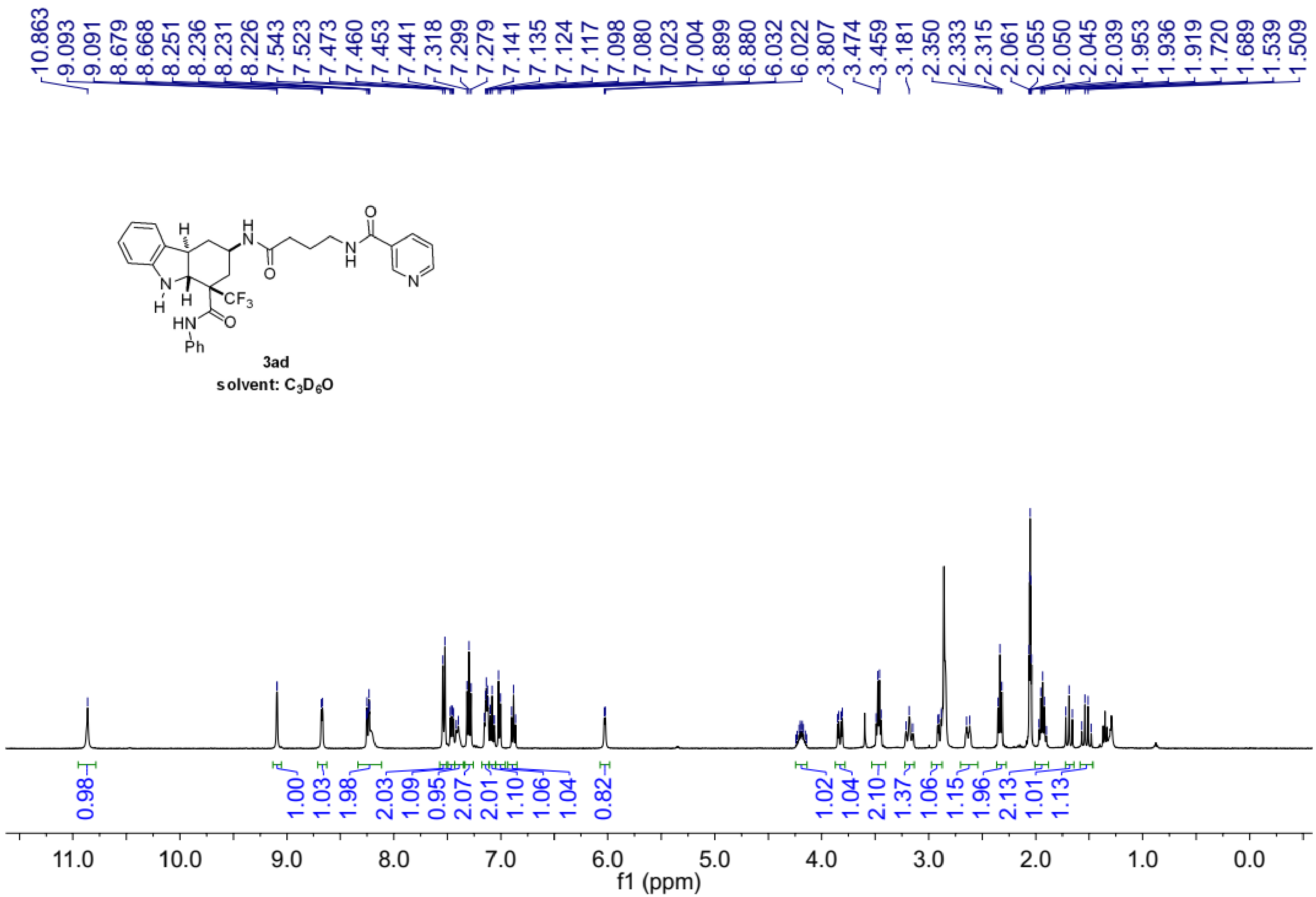

Supplementary Figure 125. ^{1}H NMR spectrum for compound **3ad**

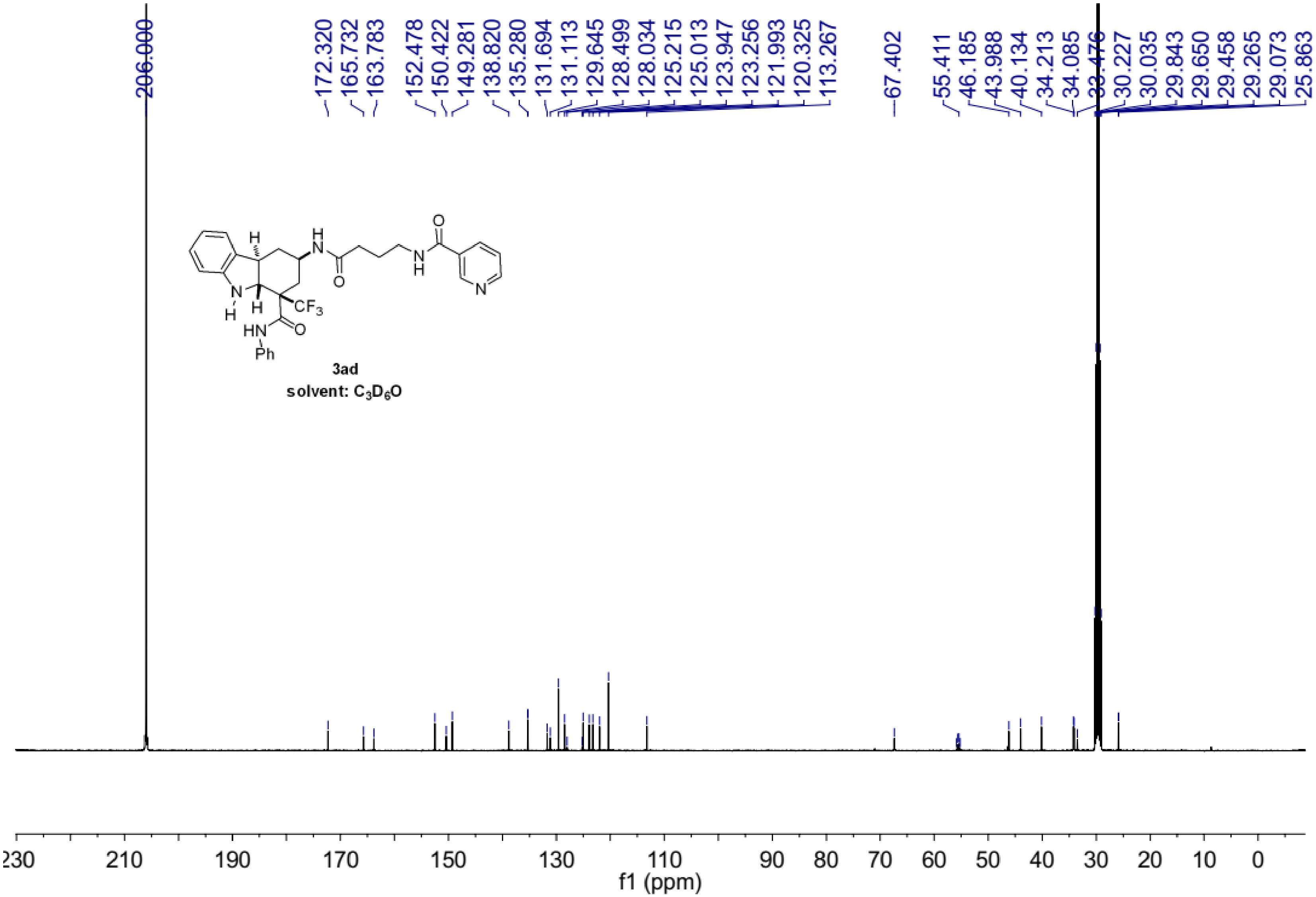

Supplementary Figure 126. ^{13}C NMR spectrum for compound **3ad**

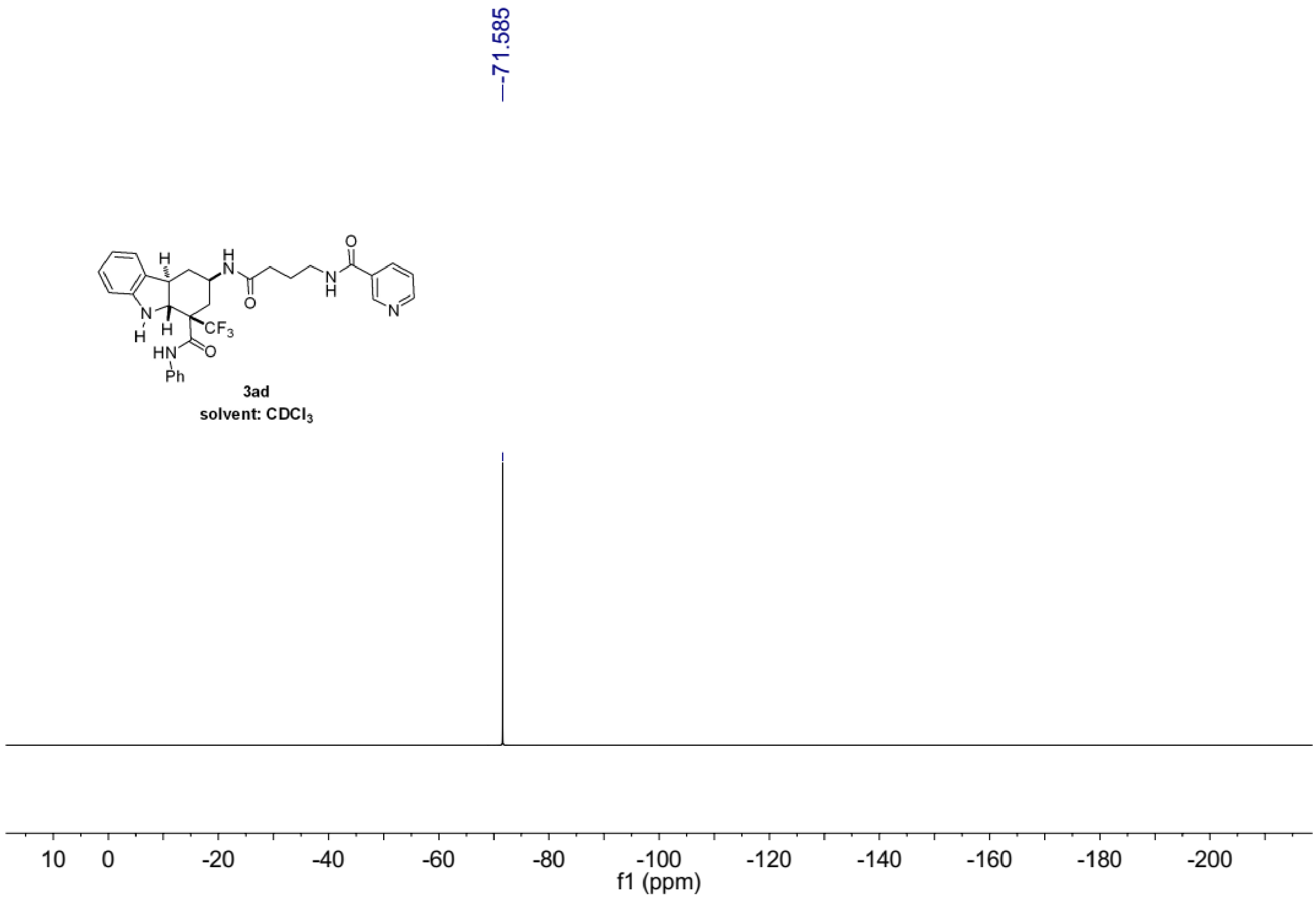

Supplementary Figure 127. ^{19}F NMR spectrum for compound **3ad**

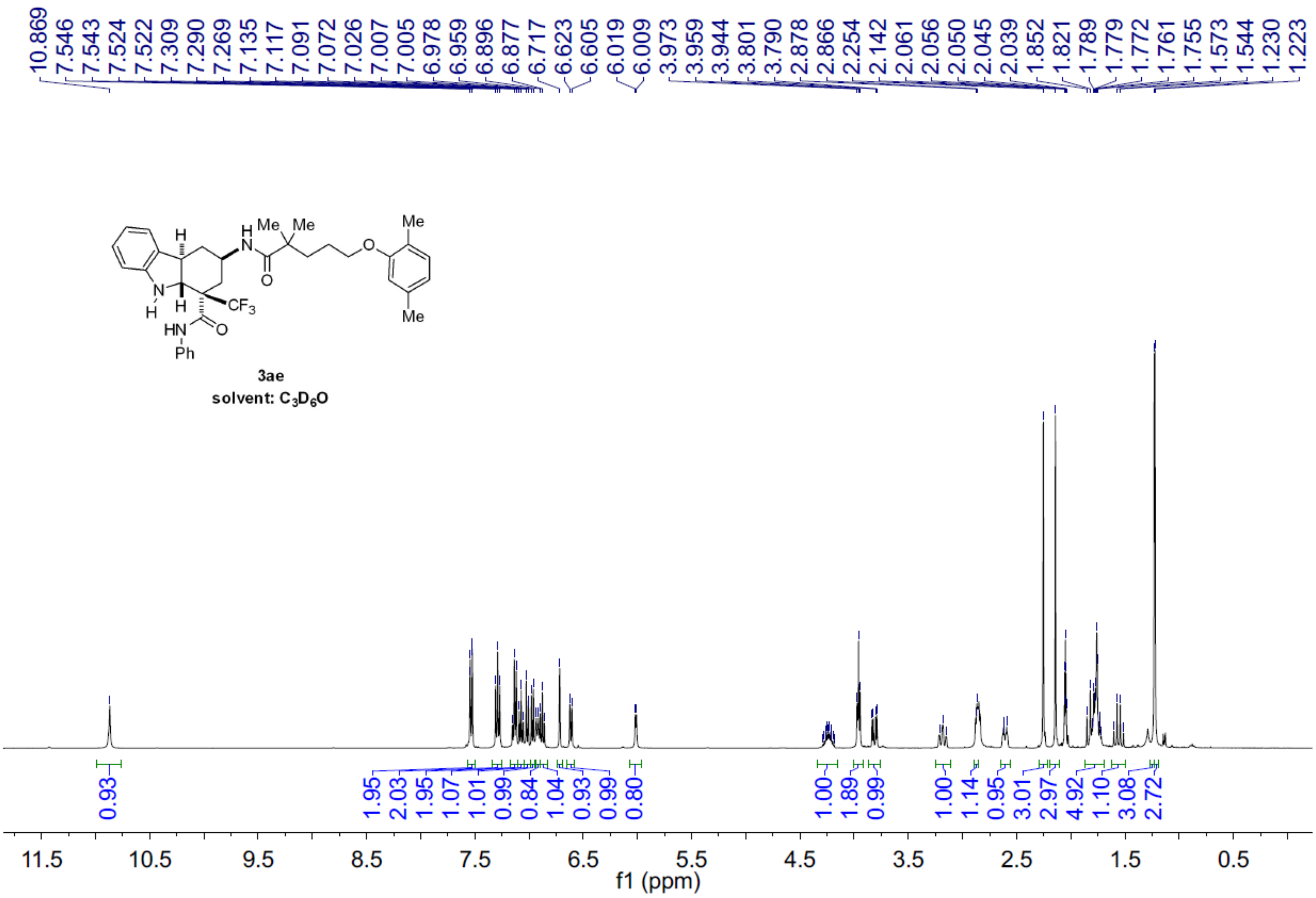

Supplementary Figure 128. ^{1}H NMR spectrum for compound **3ae**

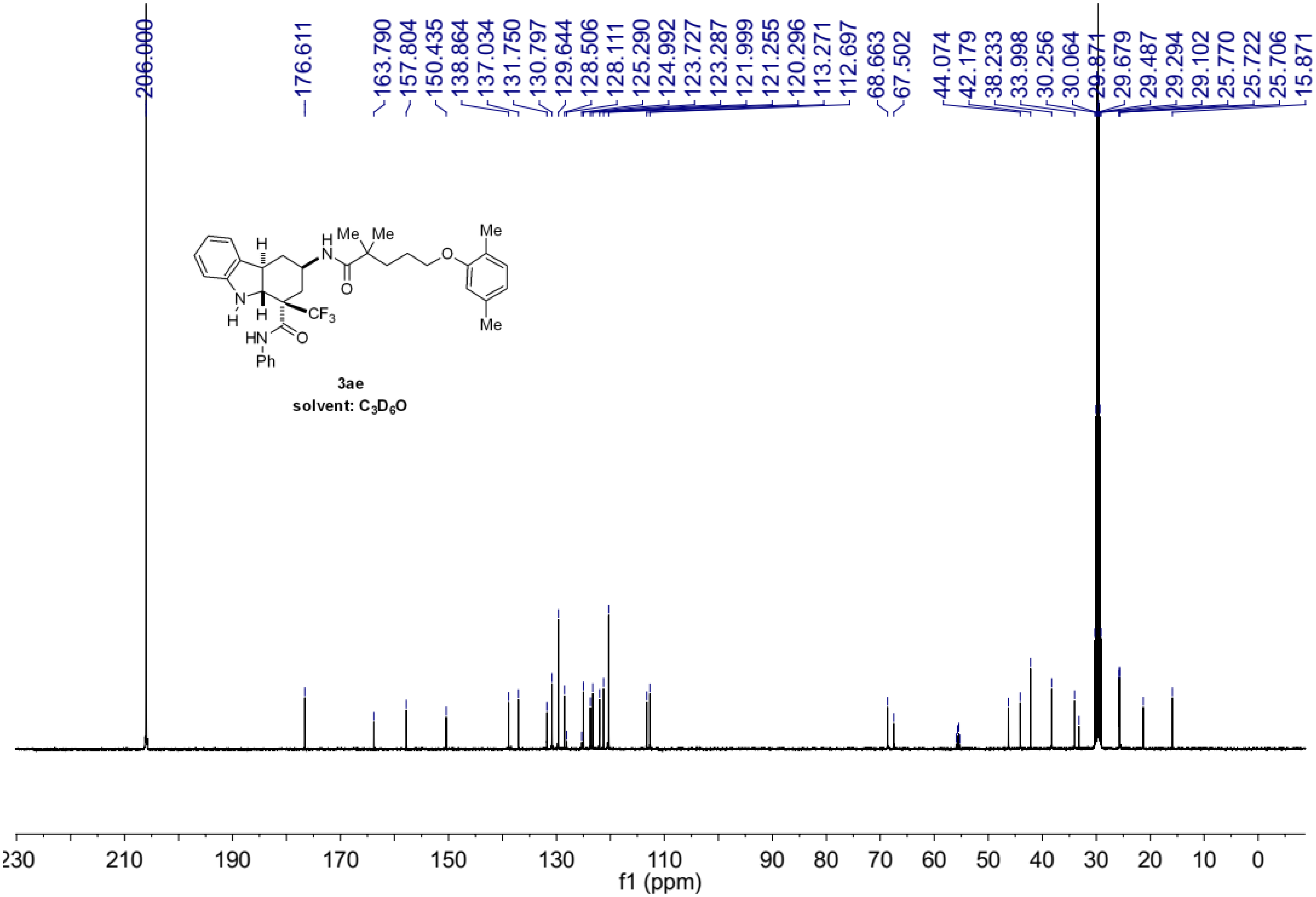

Supplementary Figure 129. ^{13}C NMR spectrum for compound **3ae**

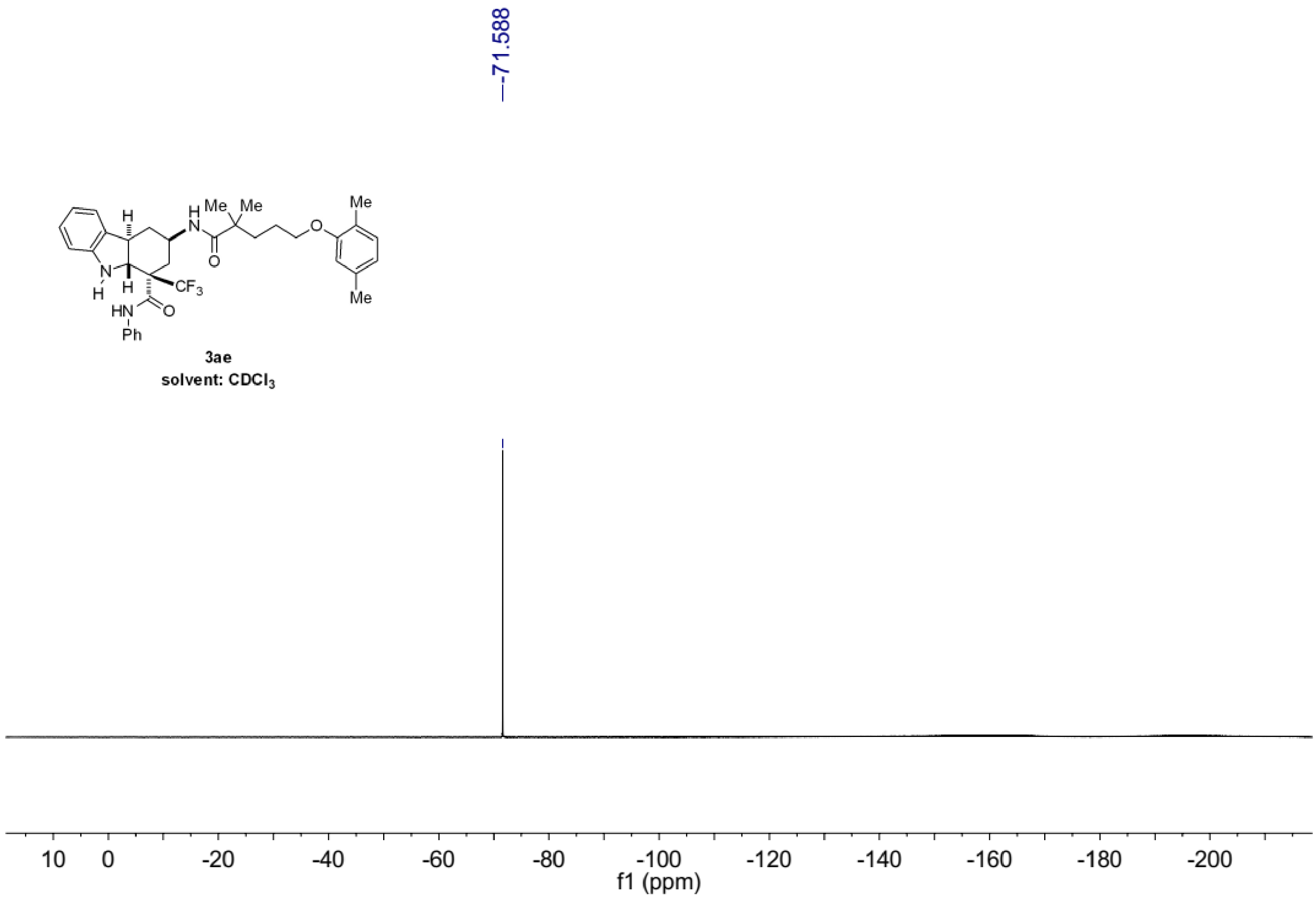

Supplementary Figure 130. ^{19}F NMR spectrum for compound **3ae**

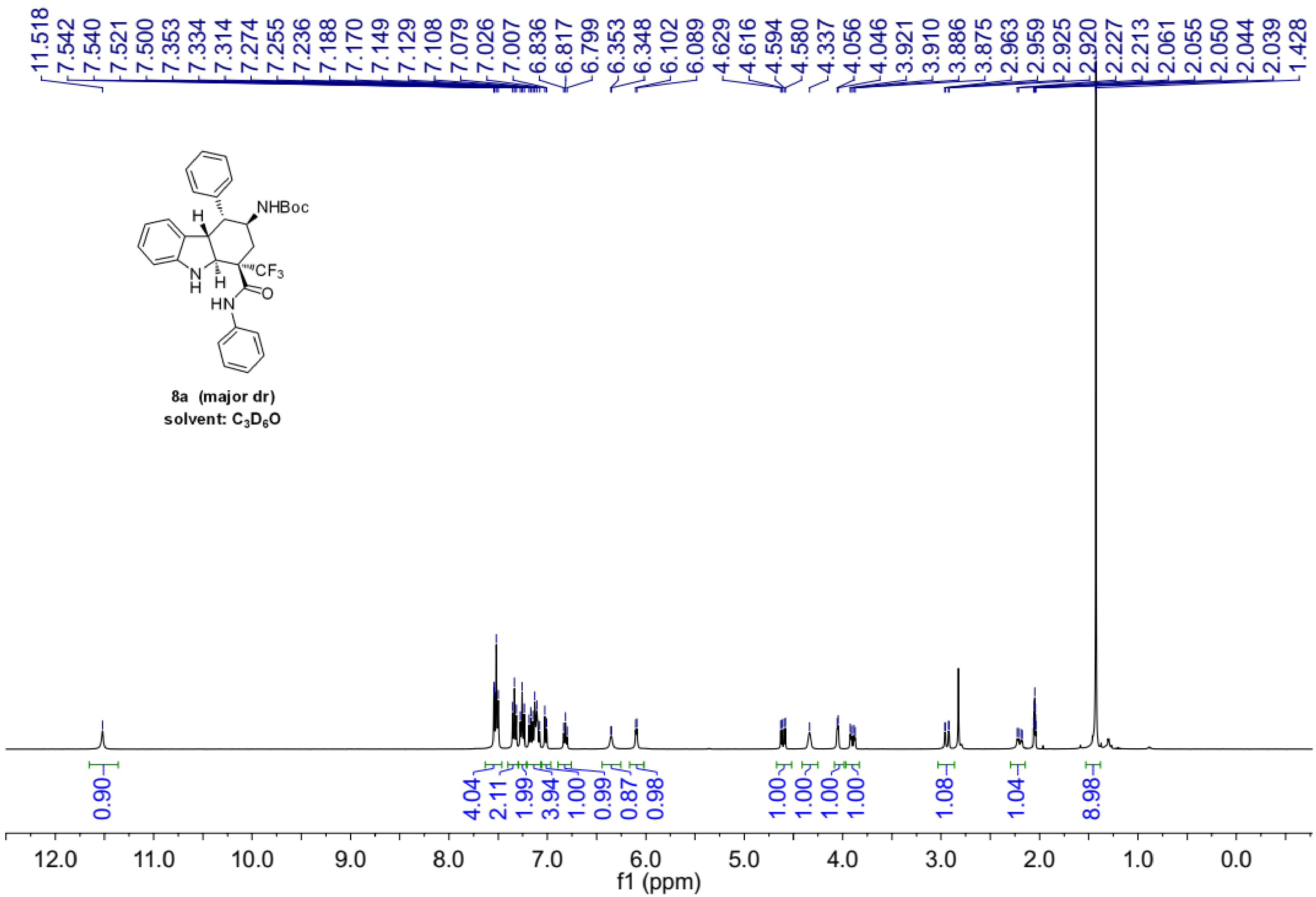

Supplementary Figure 131. ^{1}H NMR spectrum for compound **8a**

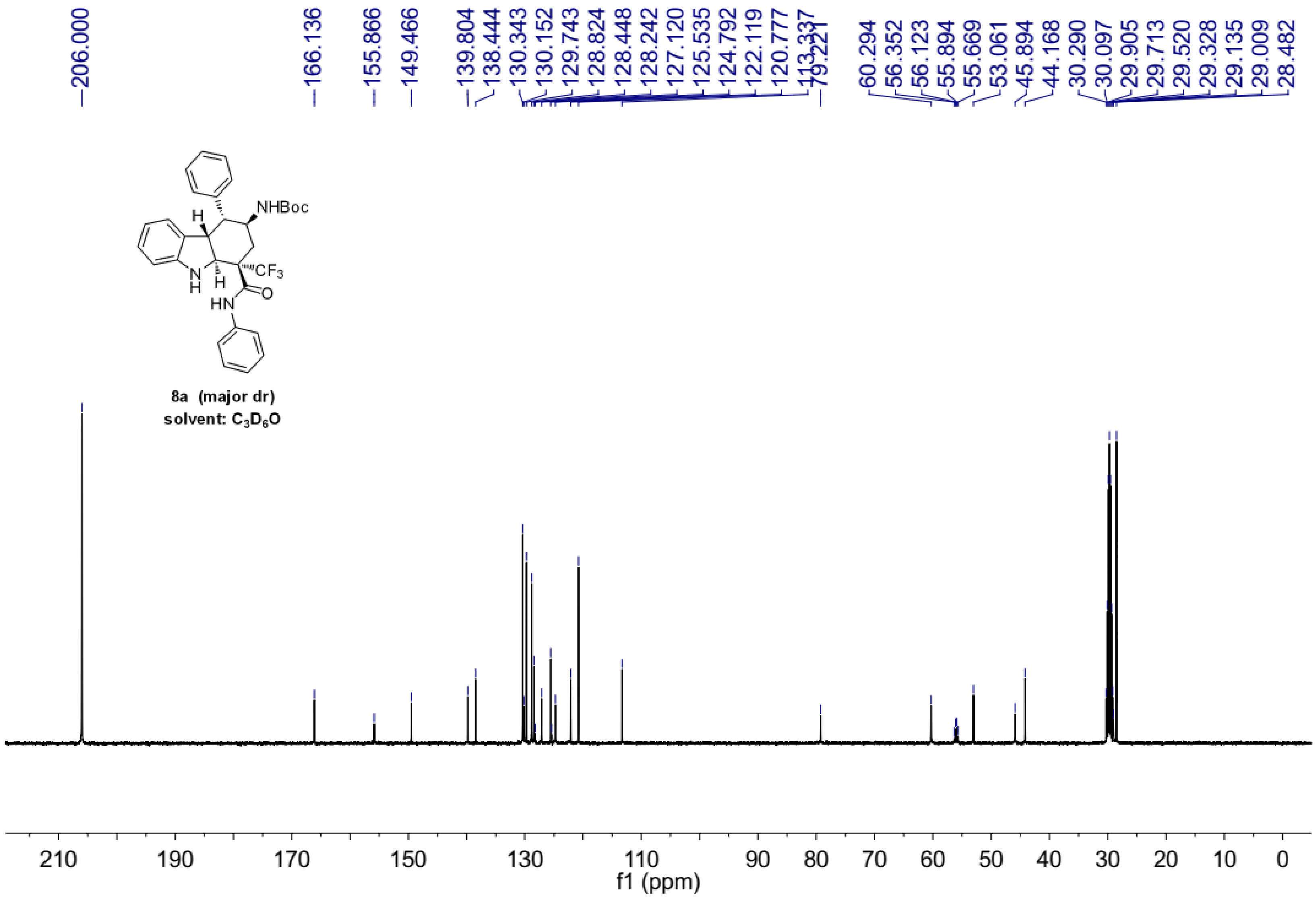

Supplementary Figure 132. ^{13}C NMR spectrum for compound **8a**

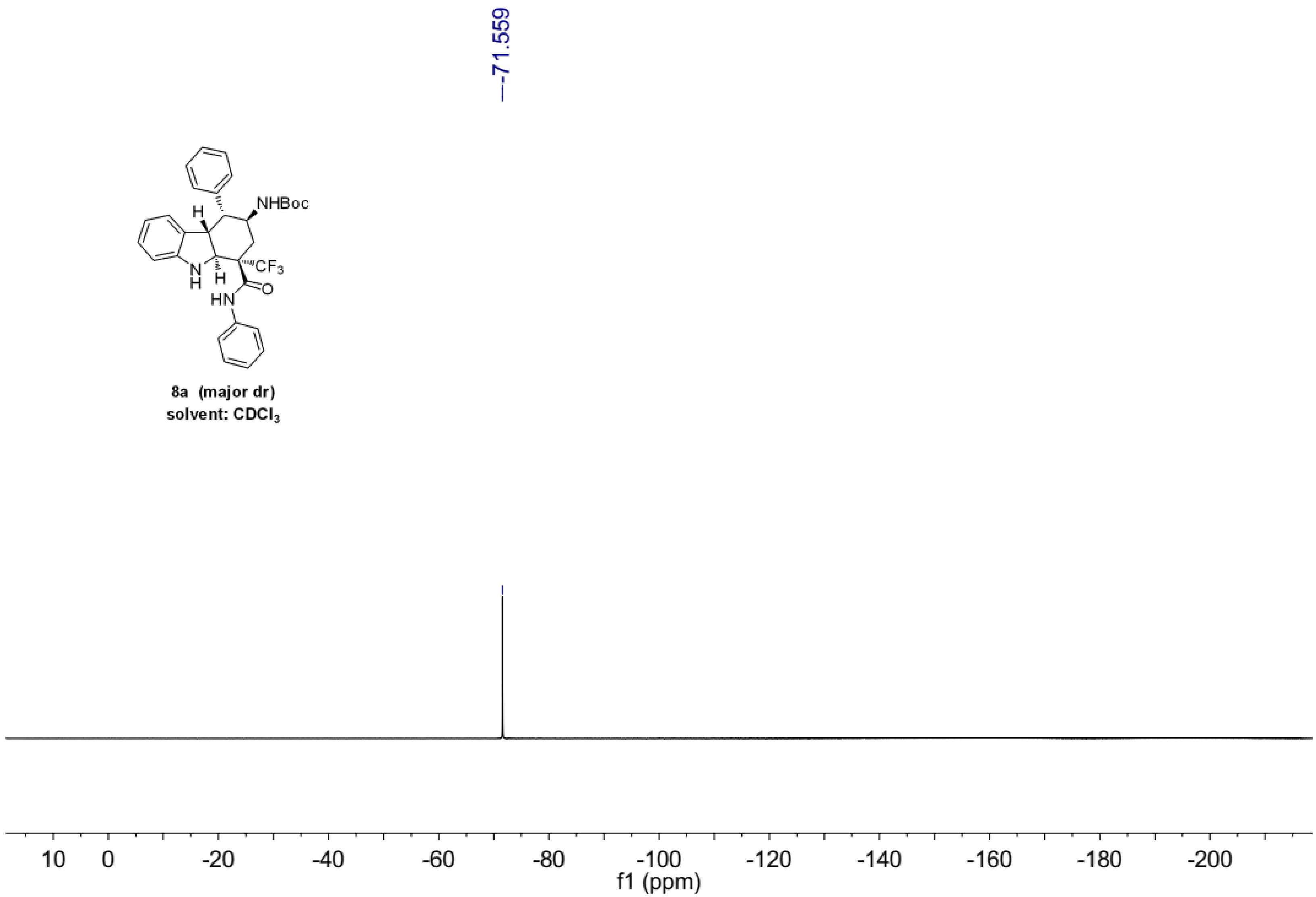

Supplementary Figure 133. ^{19}F NMR spectrum for compound **8a**

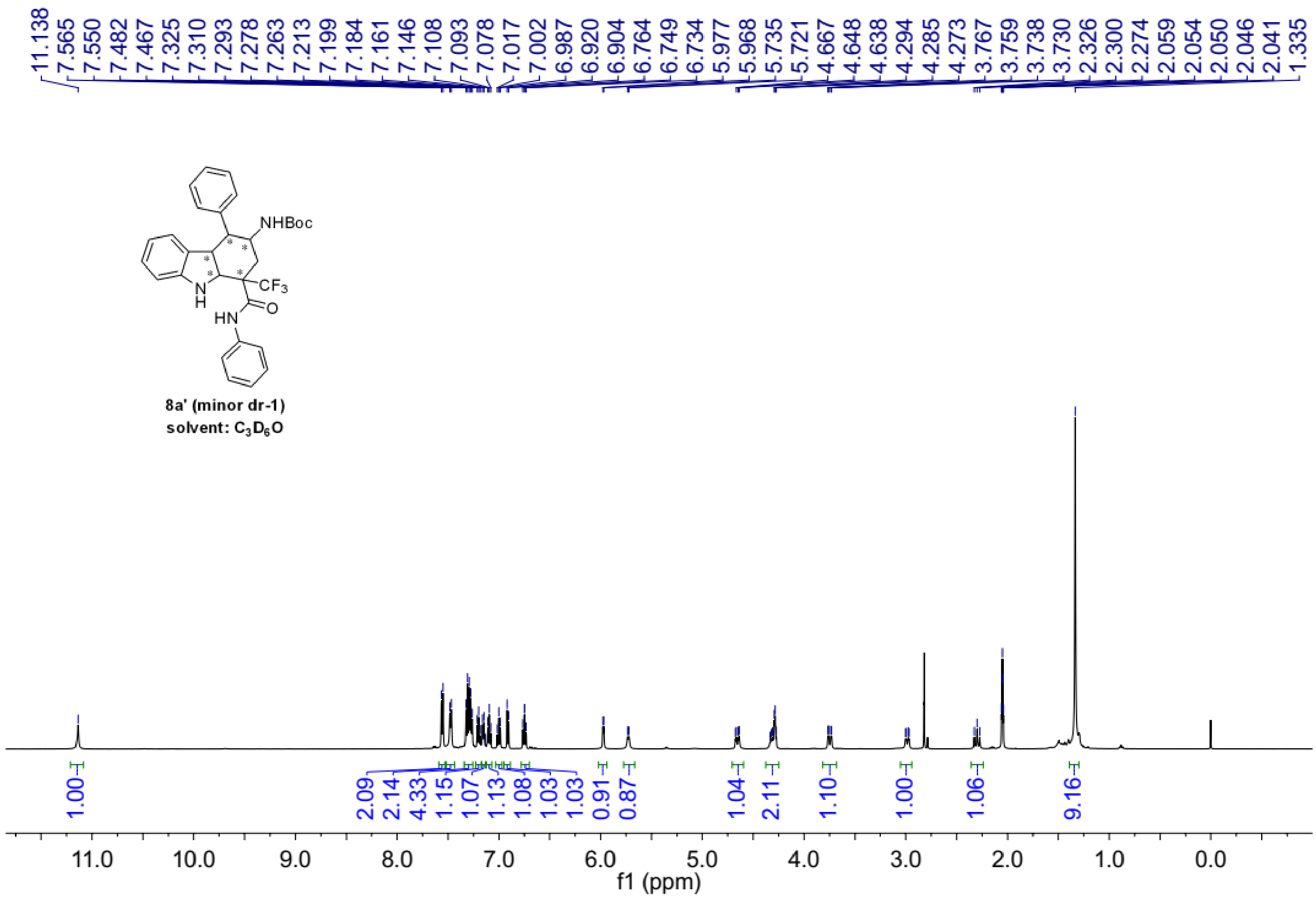

Supplementary Figure 134. ^{1}H NMR spectrum for compound **8a’**

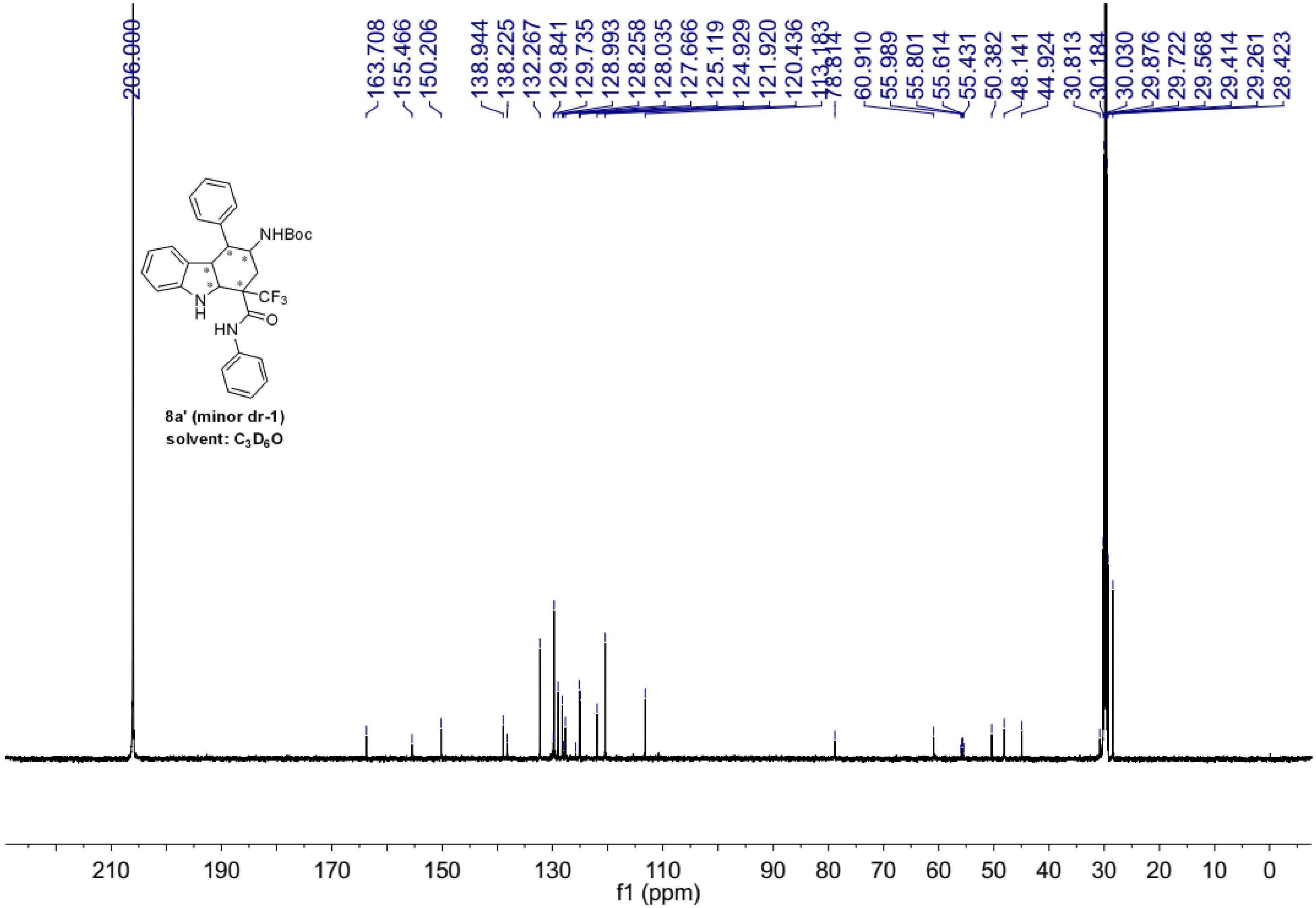

Supplementary Figure 135. ^{13}C NMR spectrum for compound **8a'**

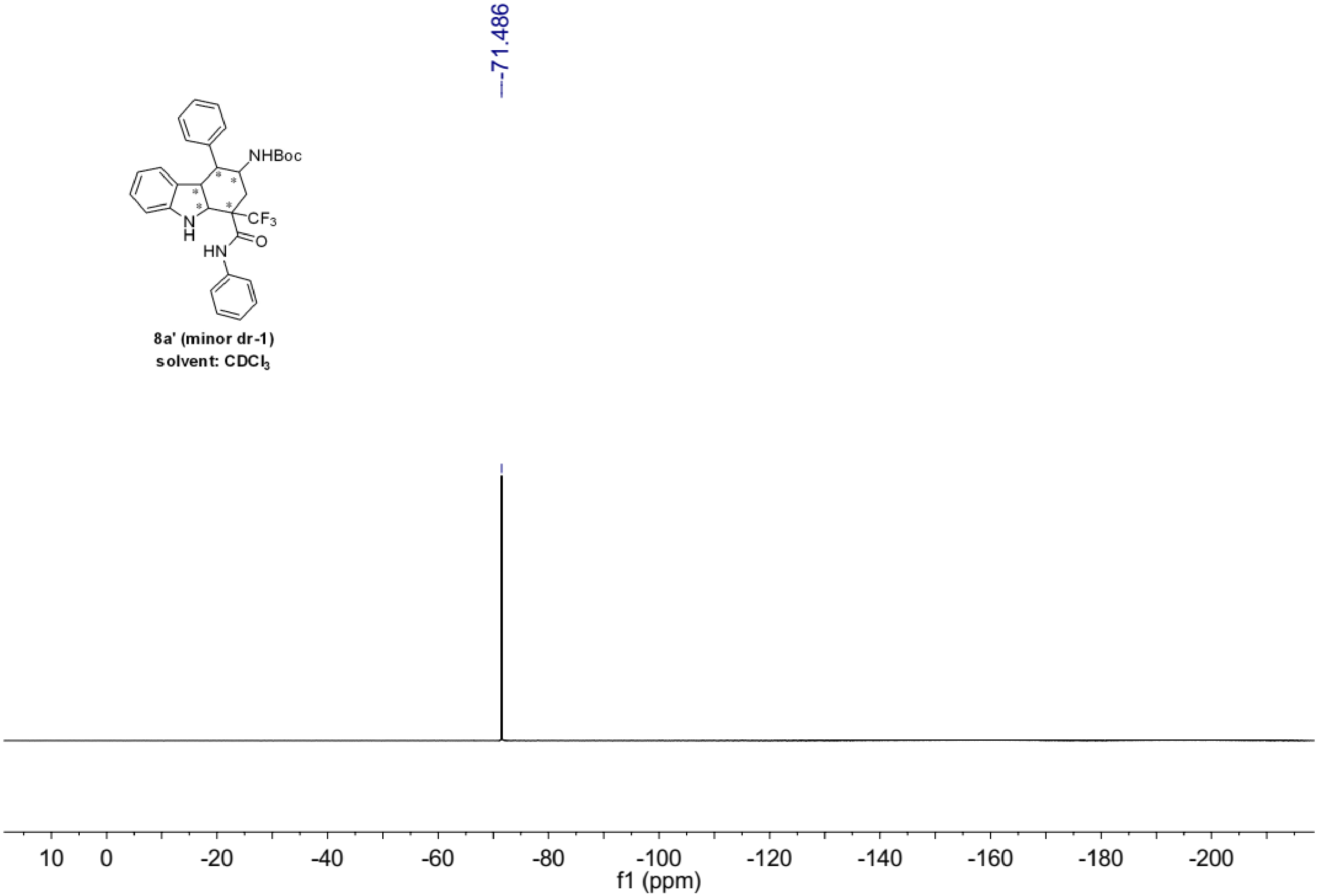

Supplementary Figure 136. ^{19}F NMR spectrum for compound **8a'**

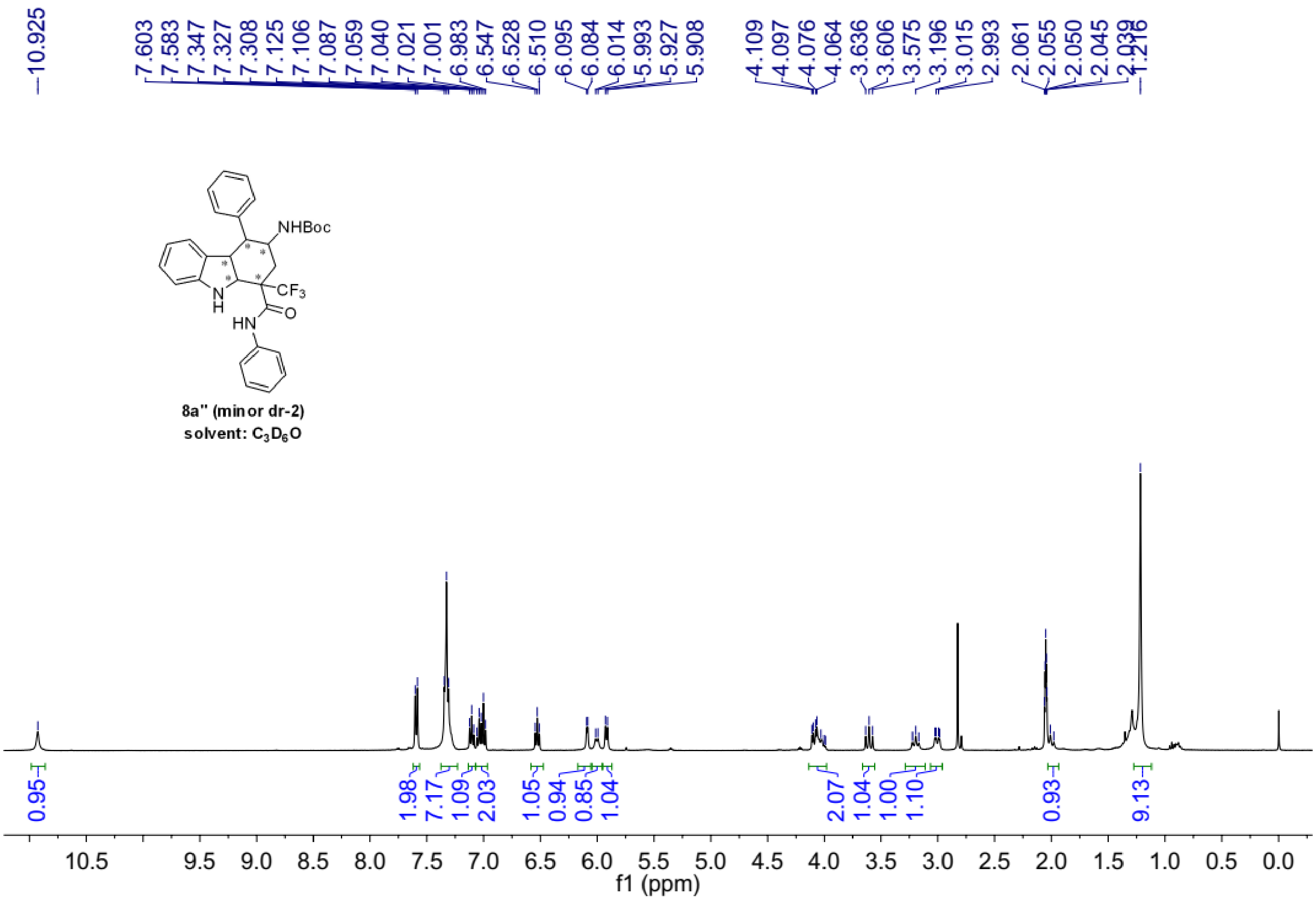

Supplementary Figure 137. ^{1}H NMR spectrum for compound **8a''**

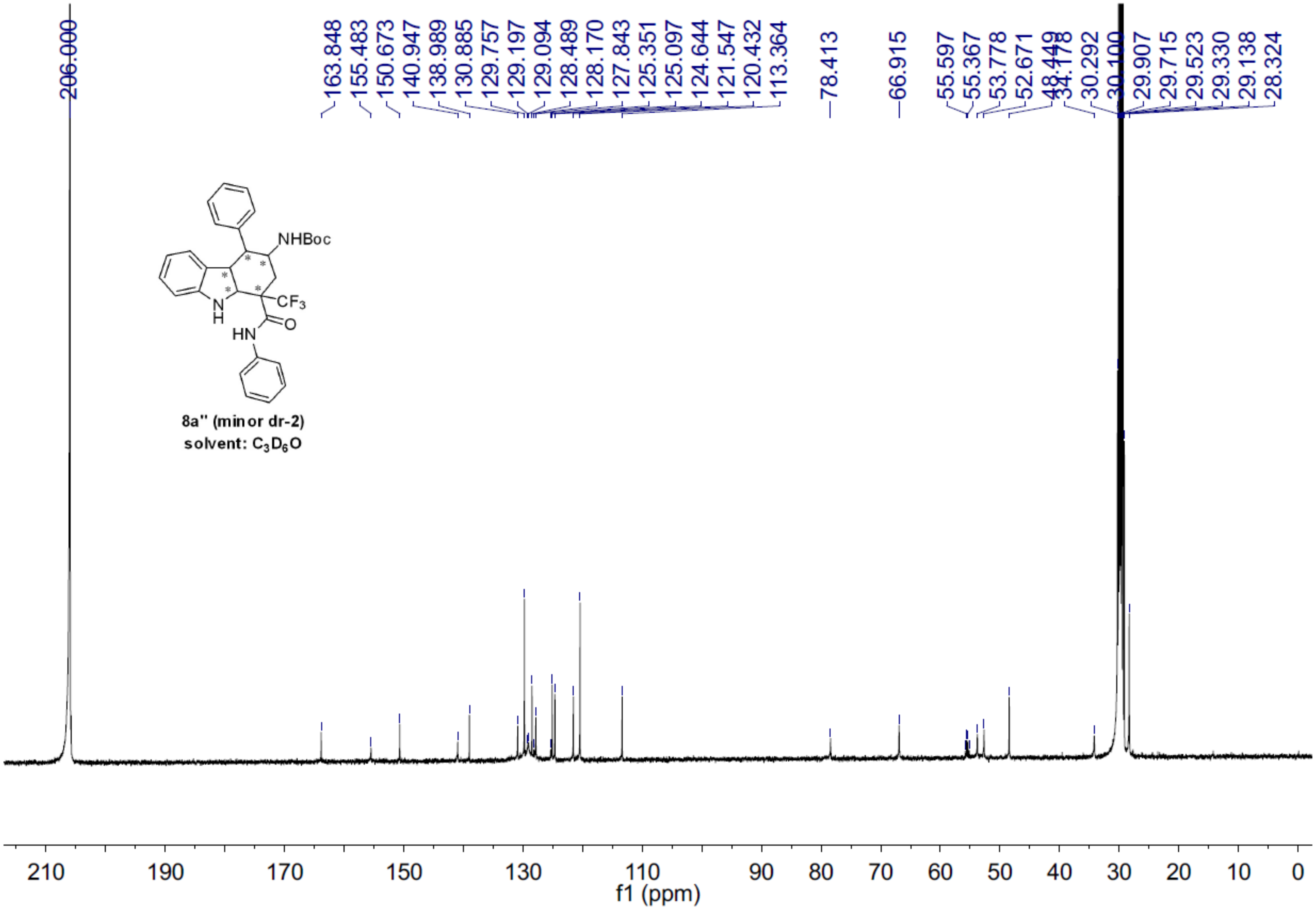

Supplementary Figure138. ^{13}C NMR spectrum for compound **8a’’**

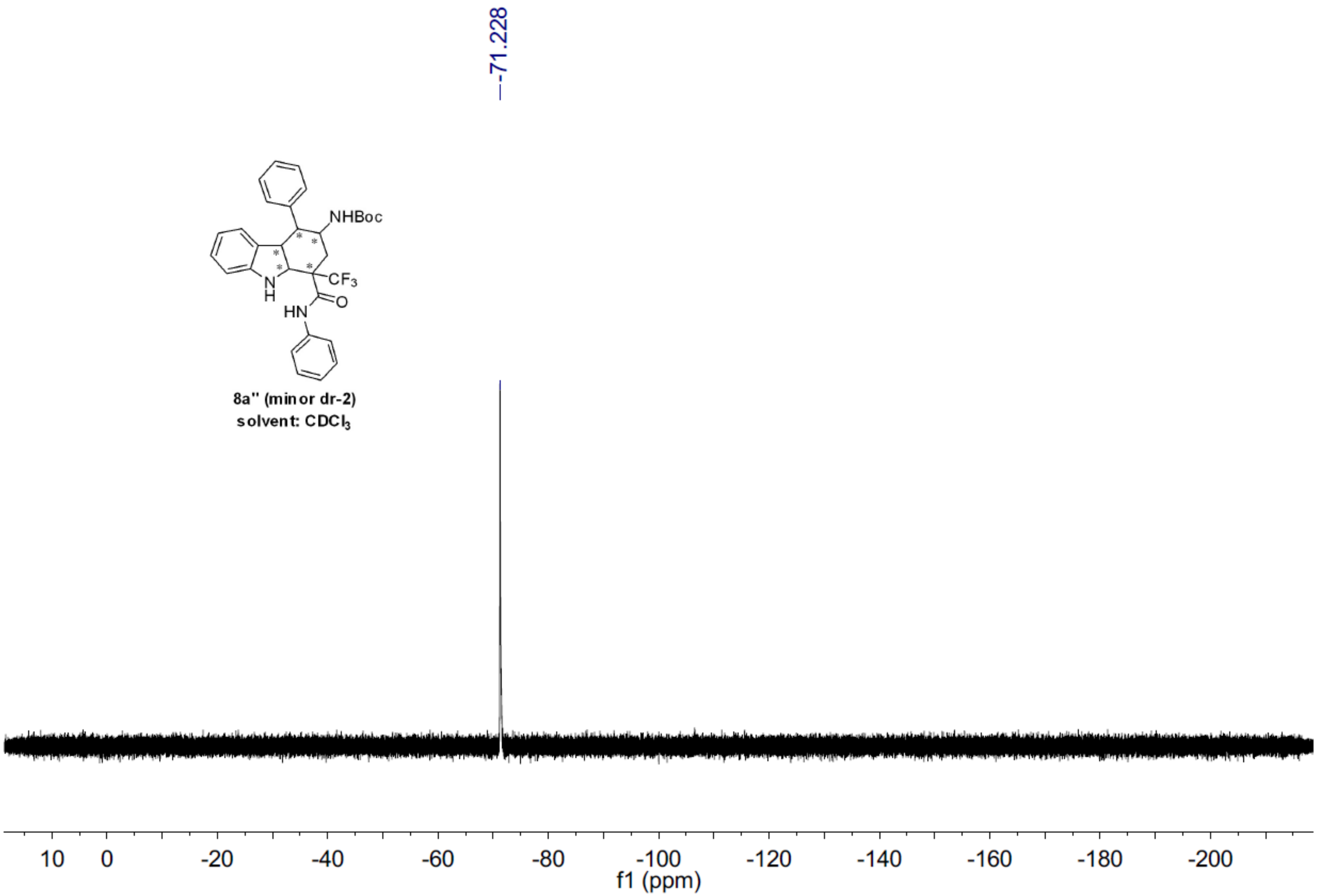

Supplementary Figure 139. ^{19}F NMR spectrum for compound **8a''**

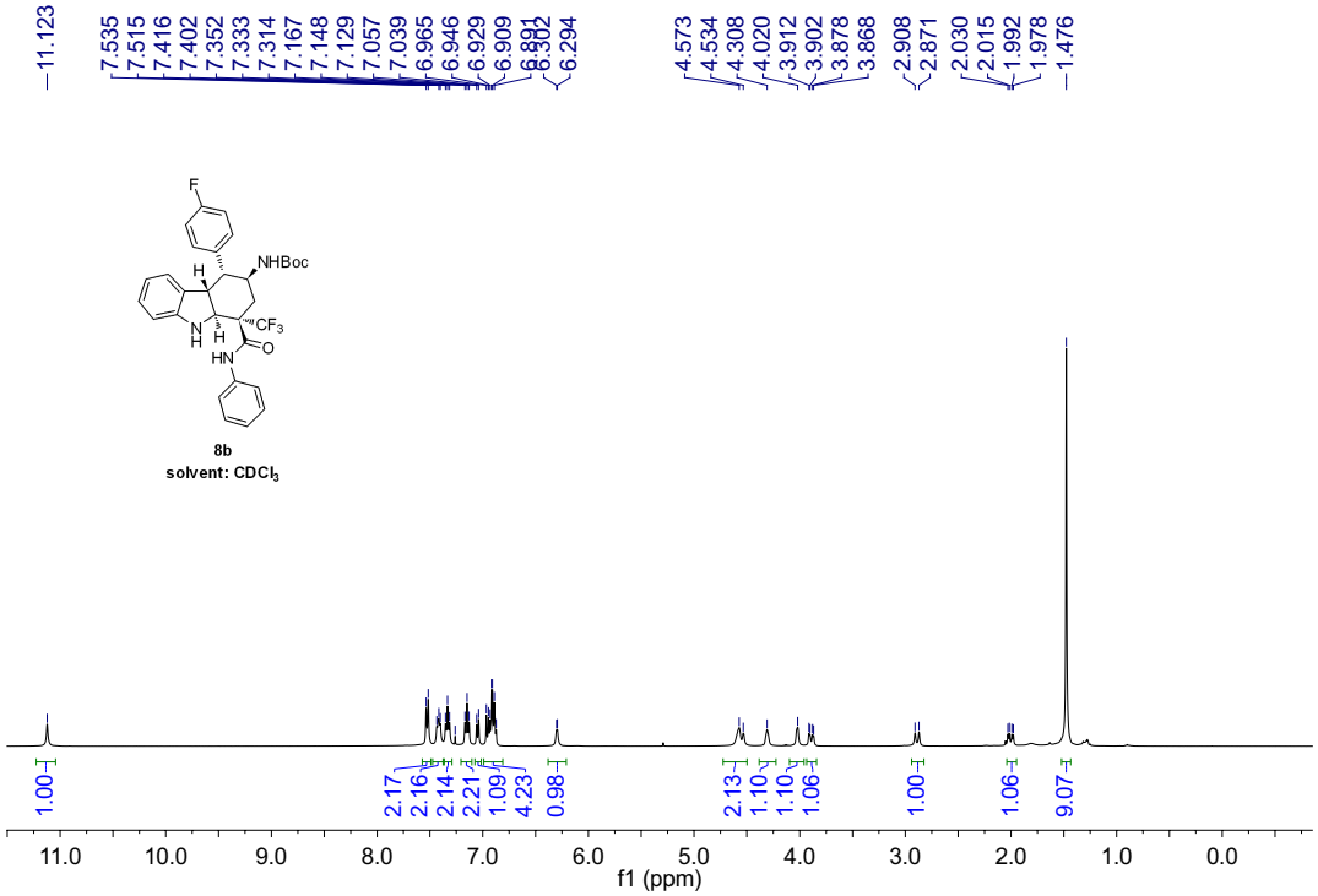

Supplementary Figure 140. ^{1}H NMR spectrum for compound **8b**

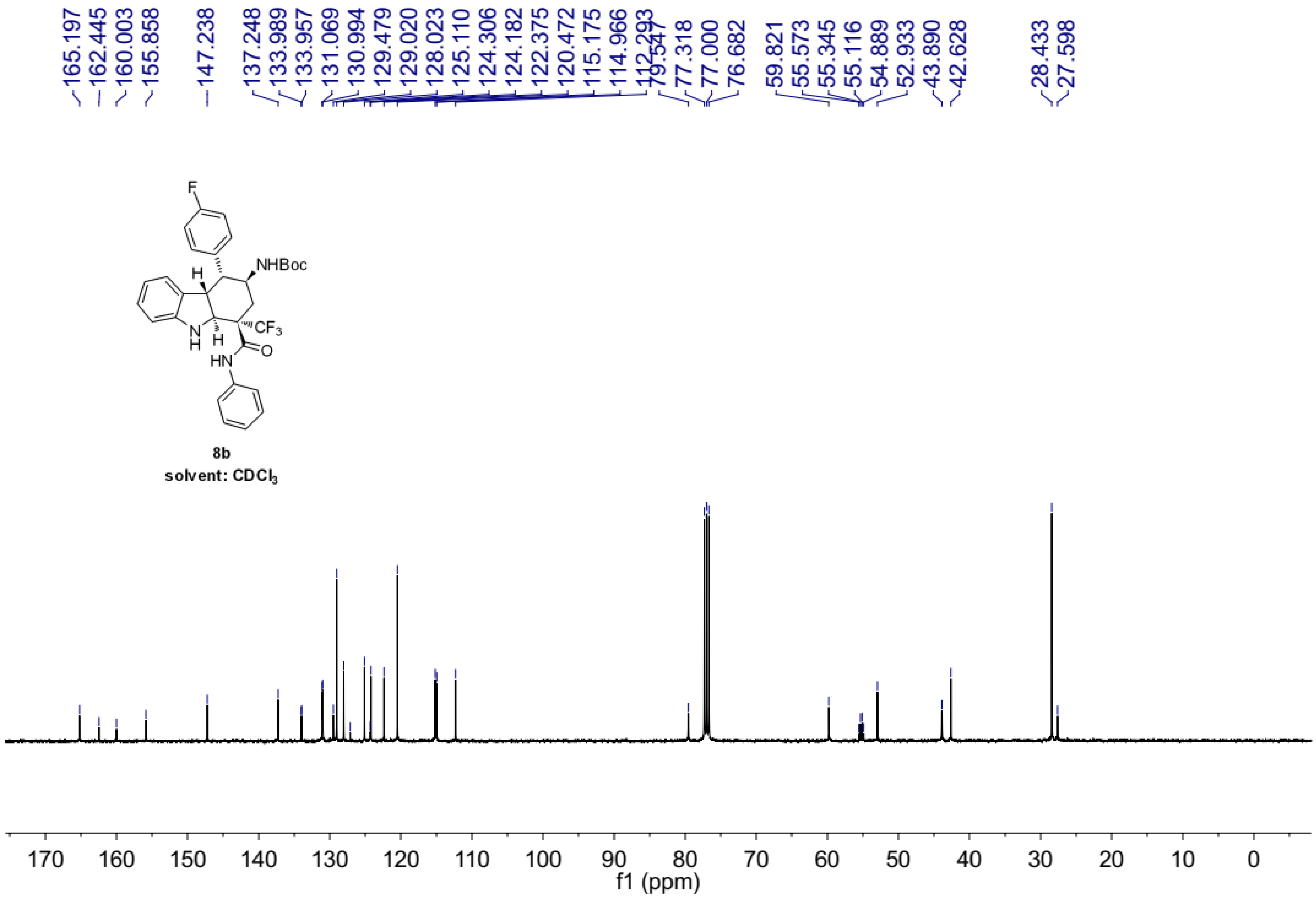

Supplementary Figure 141. ^{13}C NMR spectrum for compound **8b**

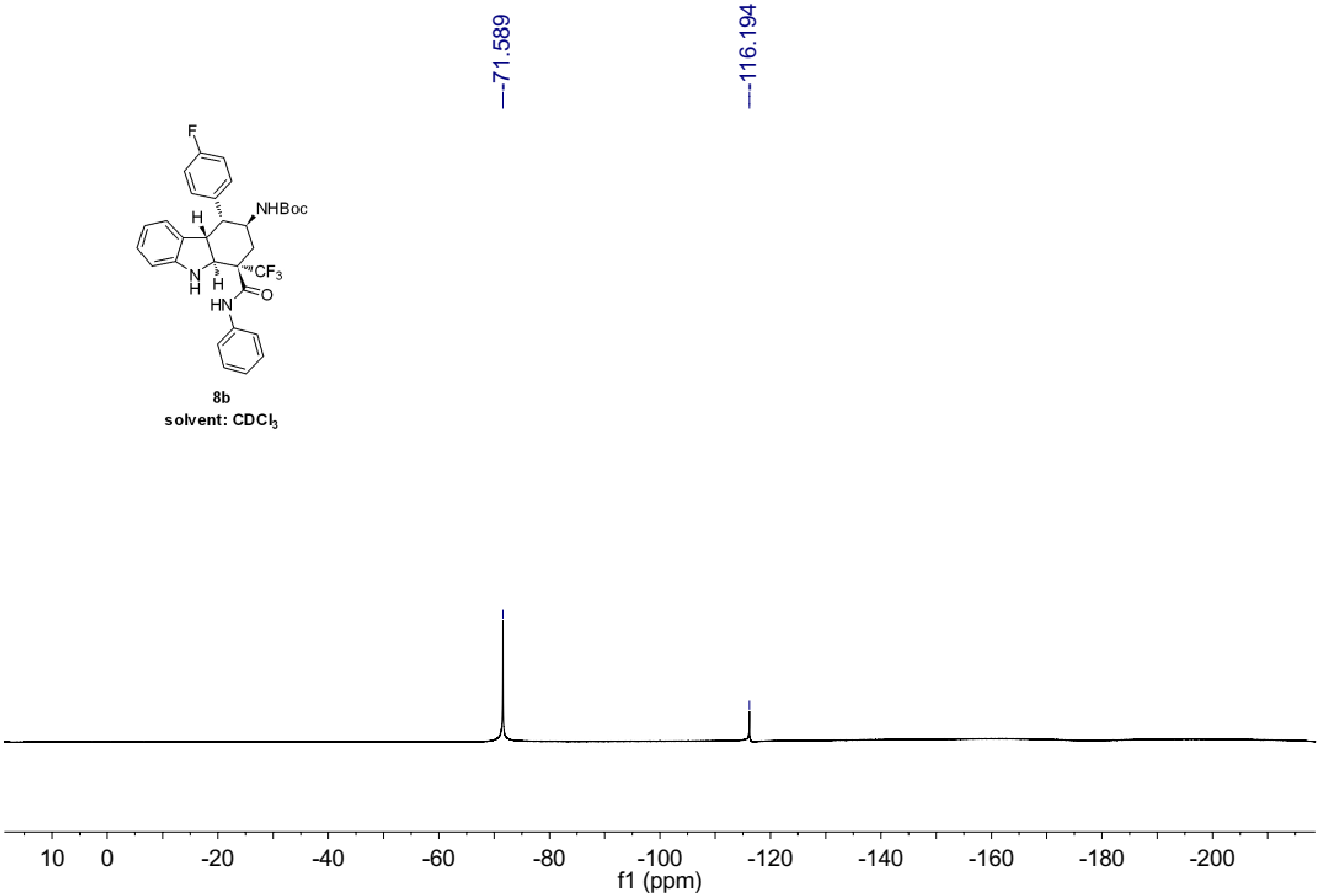

Supplementary Figure 142. ^{19}F NMR spectrum for compound **8b**

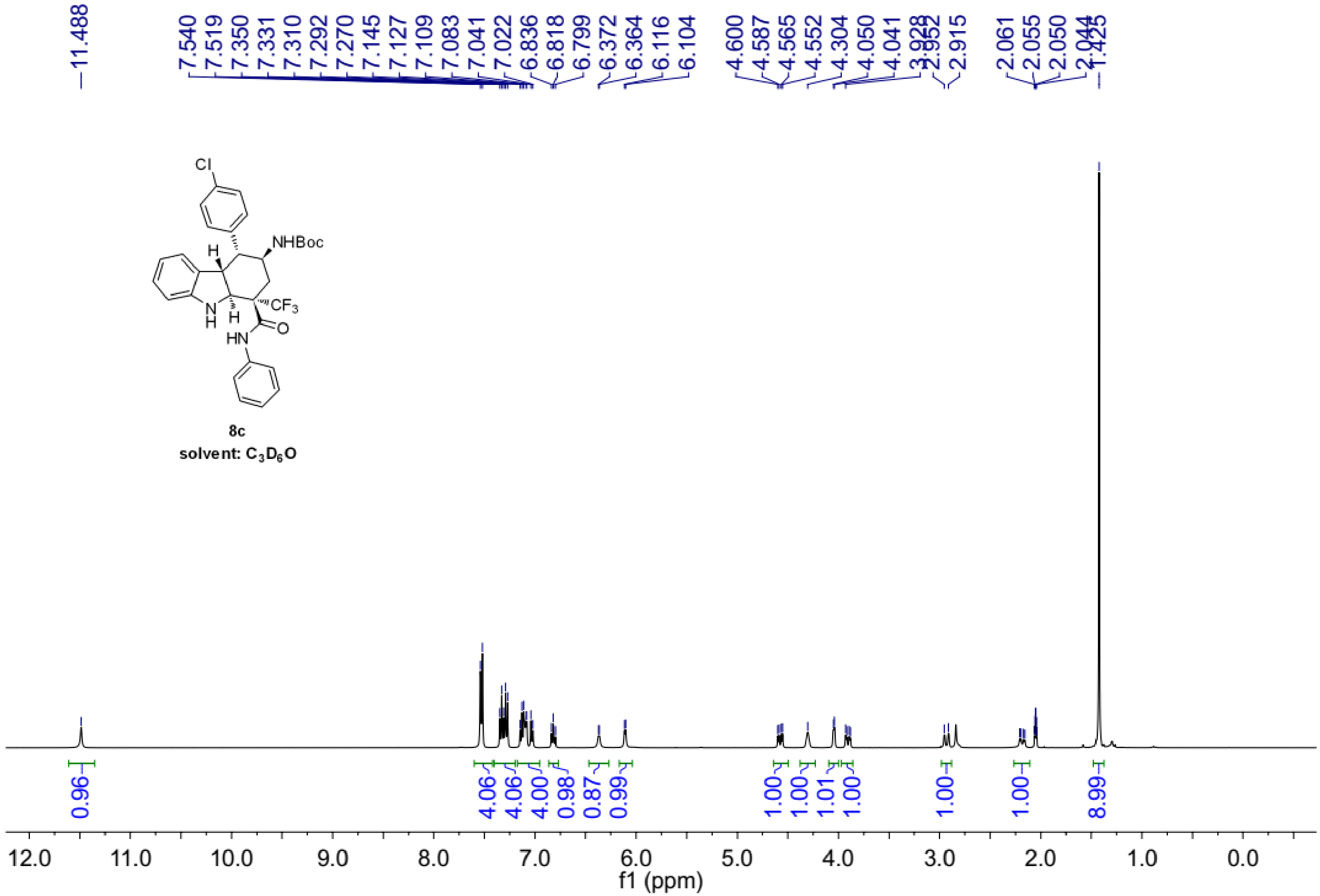

Supplementary Figure 143. ^{1}H NMR spectrum for compound **8c**

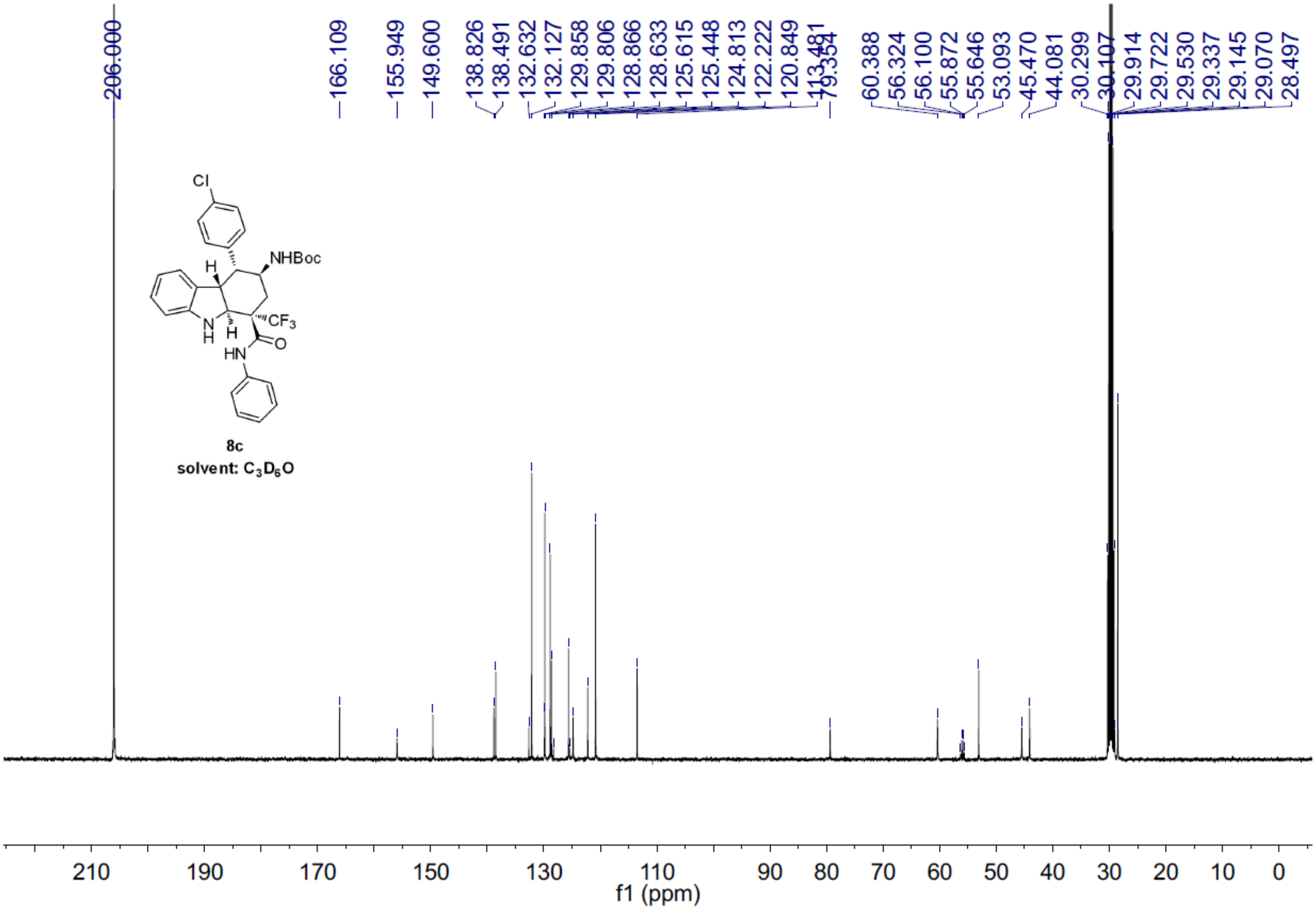

Supplementary Figure 144. ^{13}C NMR spectrum for compound **8c**

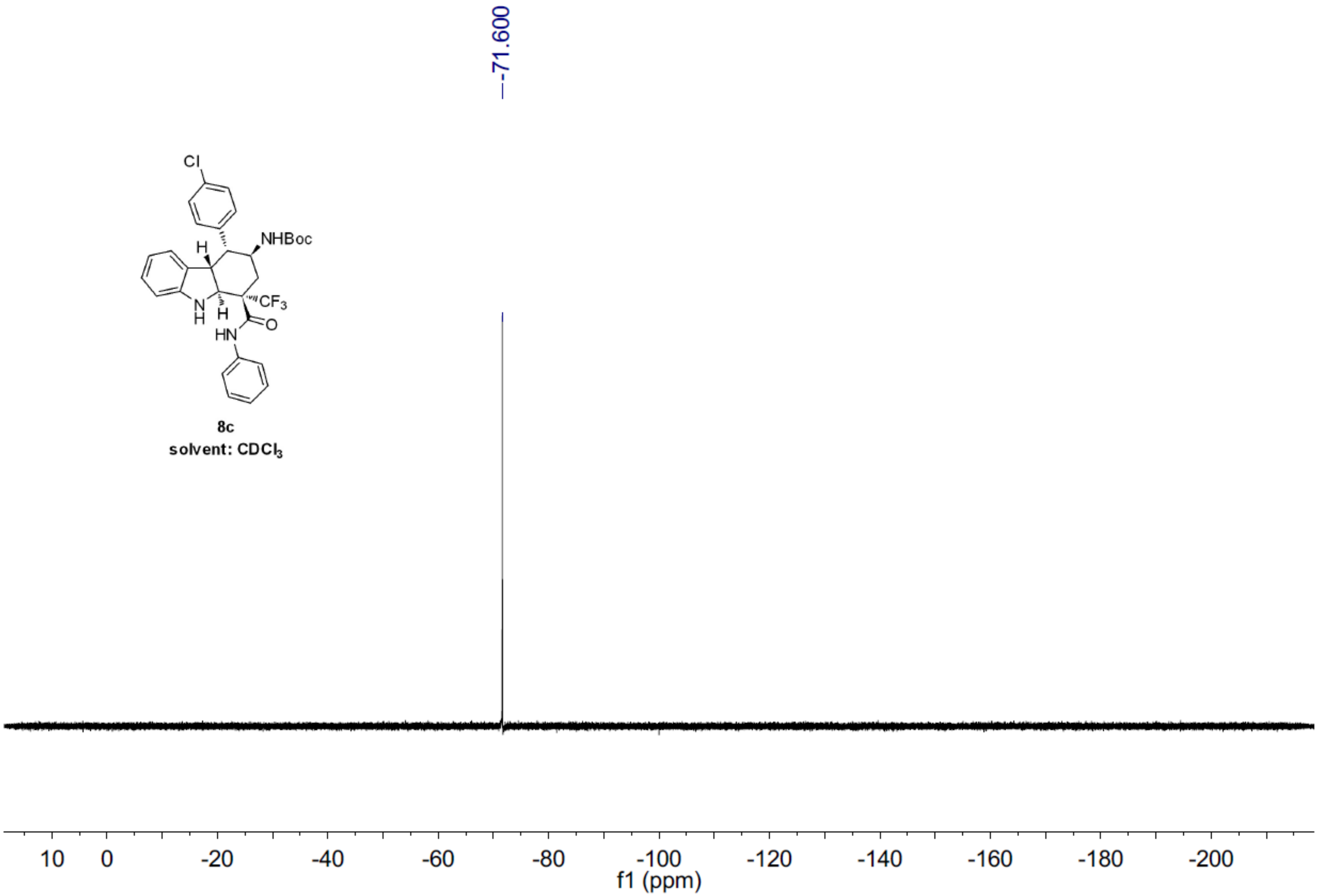

Supplementary Figure 145. ^{19}F NMR spectrum for compound **8c**

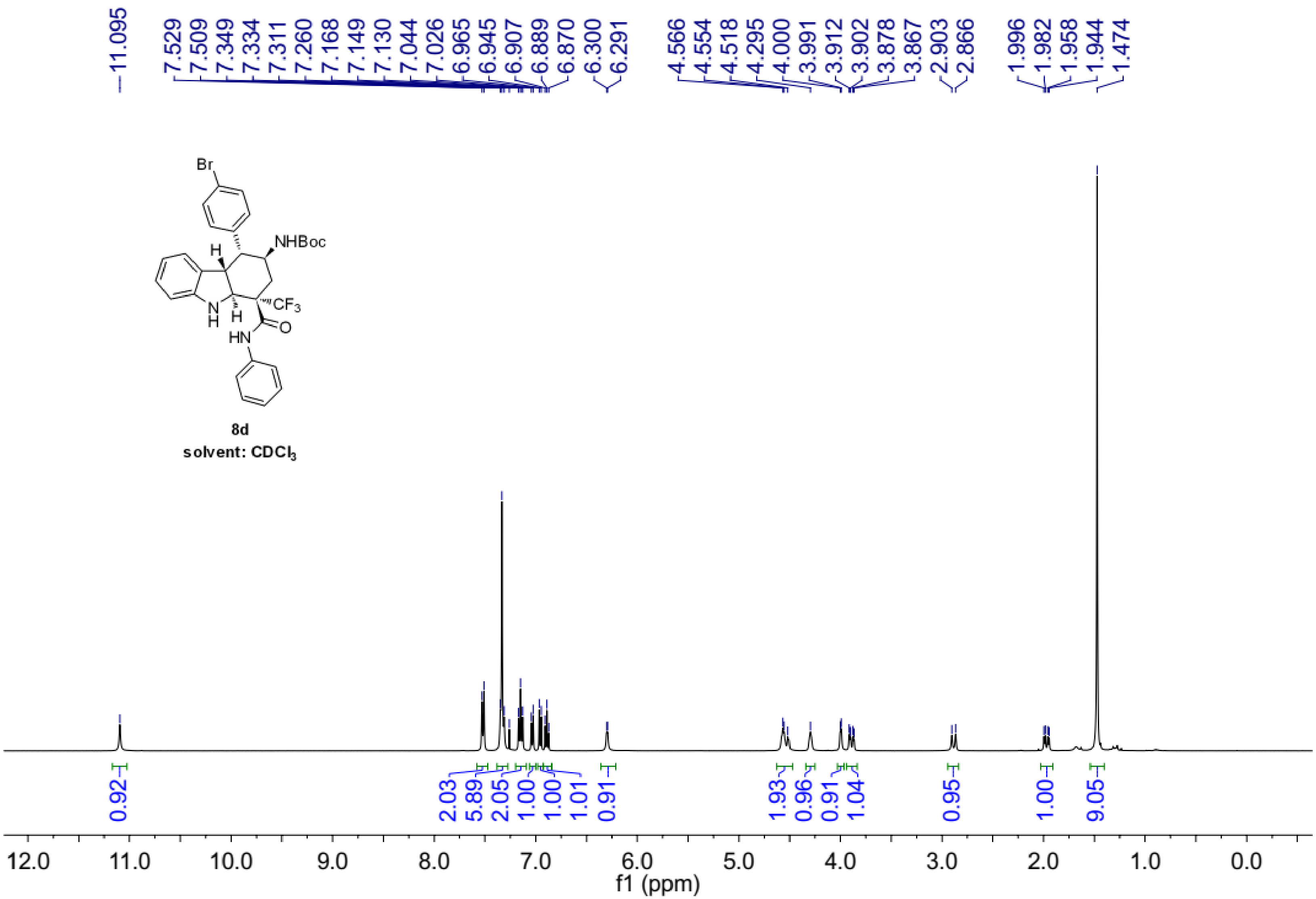

Supplementary Figure 146. ^{1}H NMR spectrum for compound **8d**

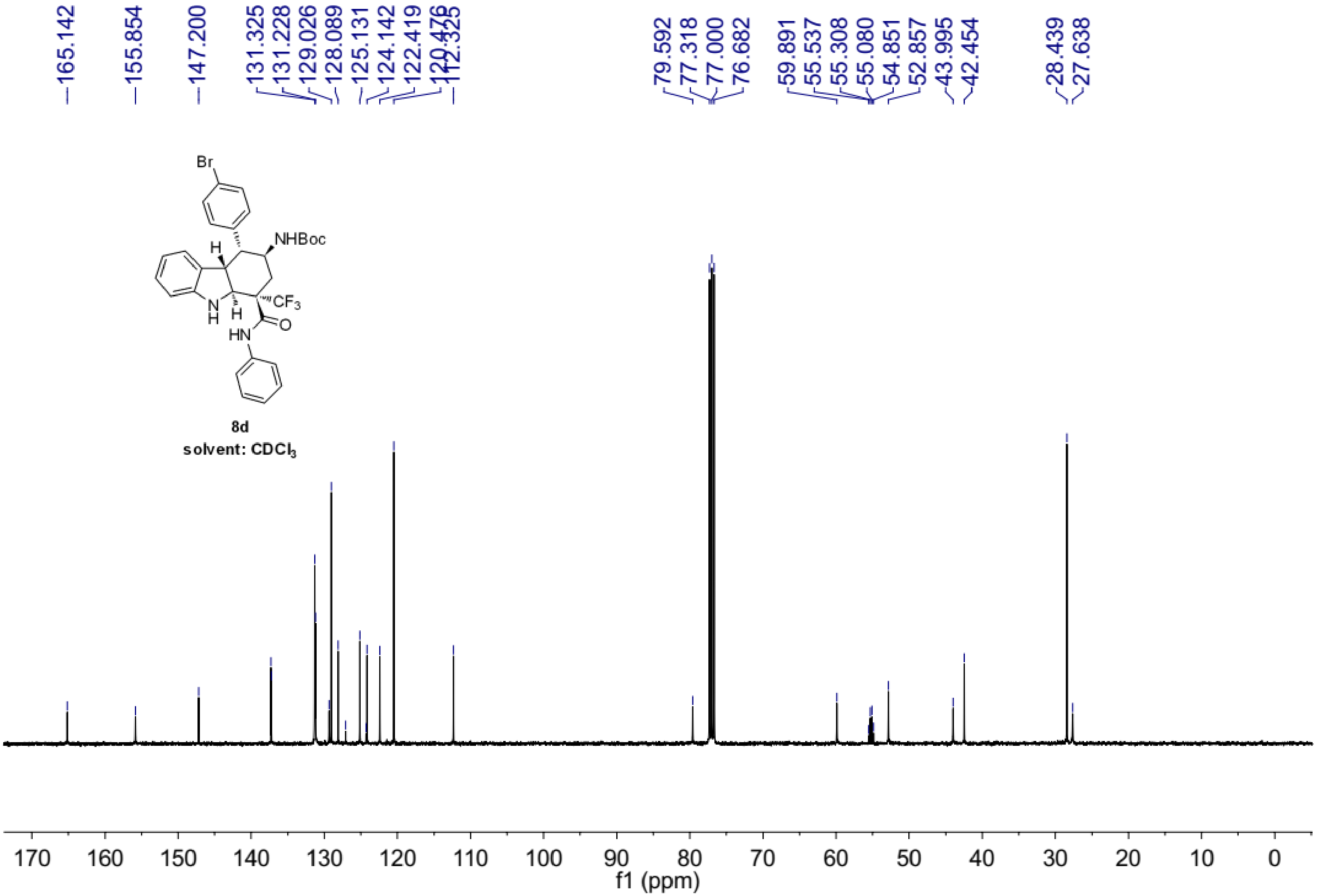

Supplementary Figure 147. ^{13}C NMR spectrum for compound **8d**

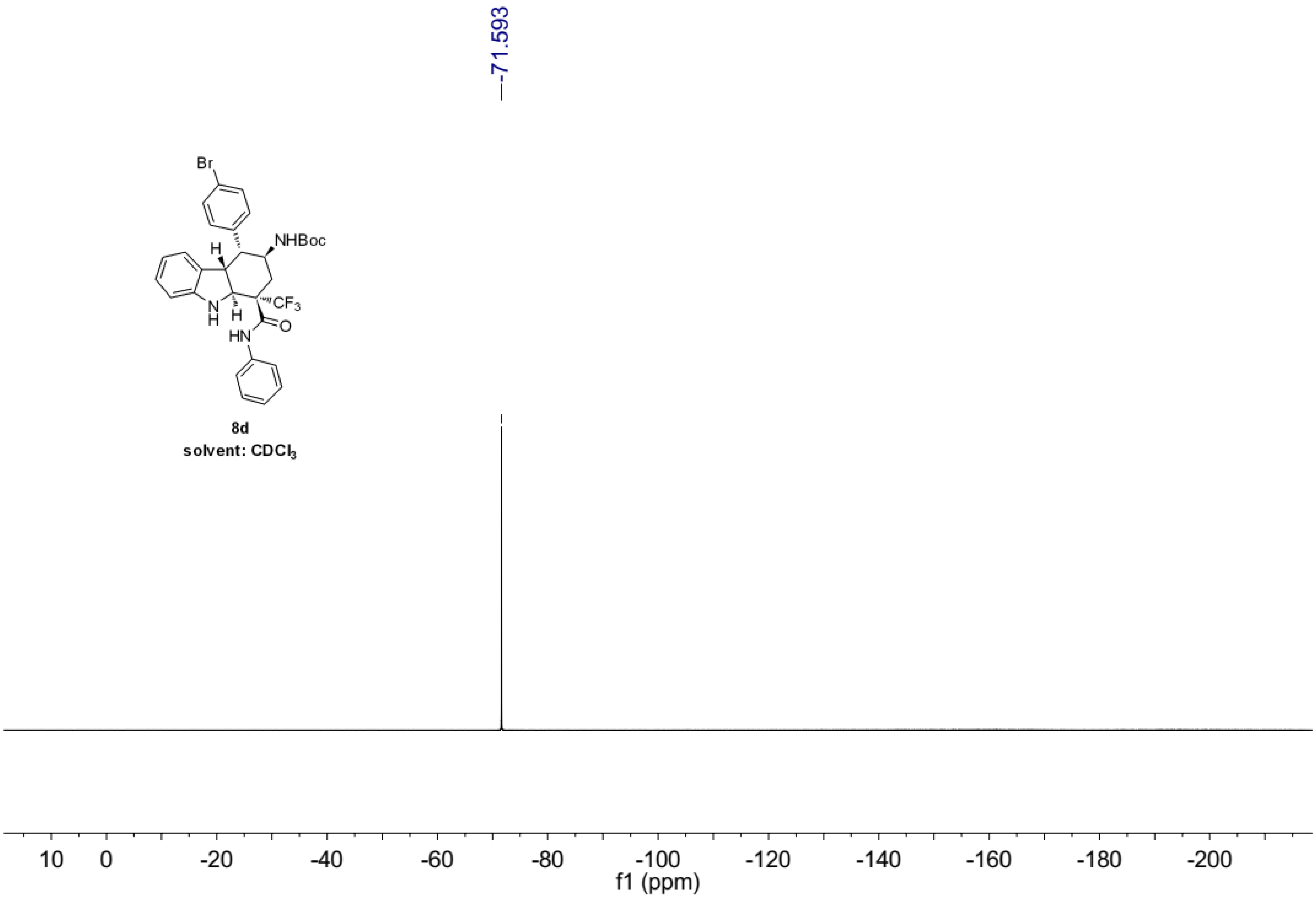

Supplementary Figure 148. ^{19}F NMR spectrum for compound **8d**

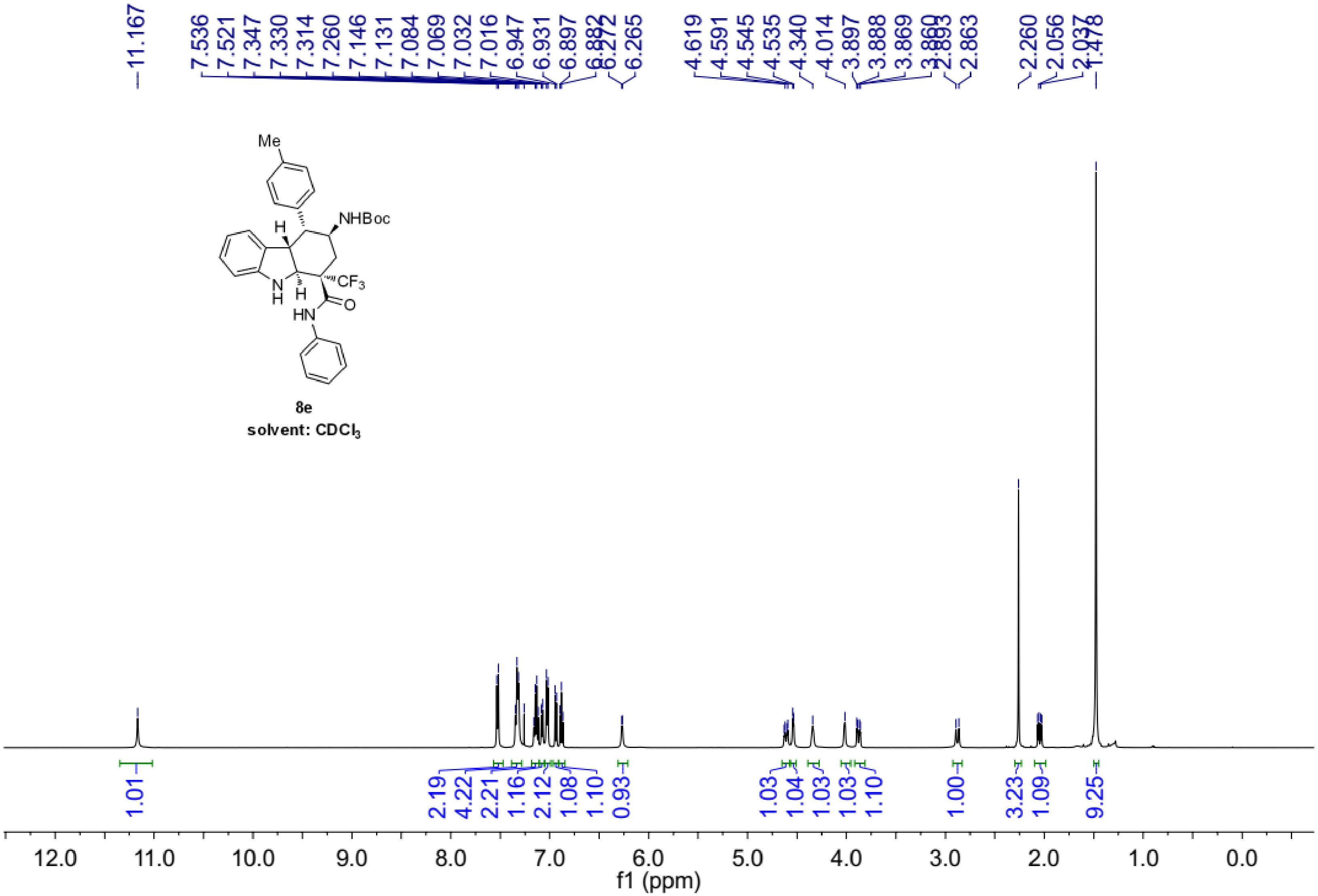

Supplementary Figure 149. ^{1}H NMR spectrum for compound **8e**

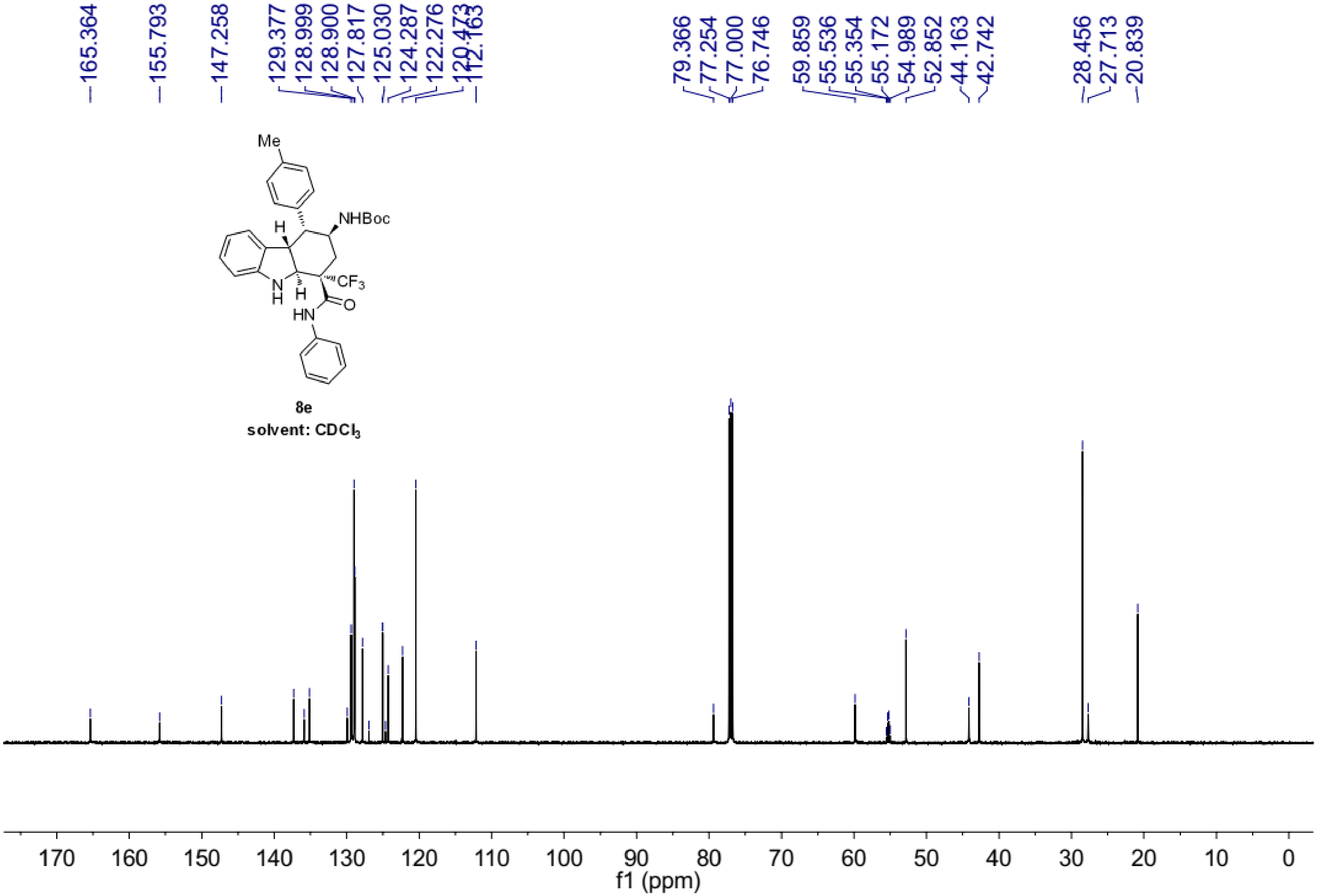

Supplementary Figure 150. ^{13}C NMR spectrum for compound **8e**

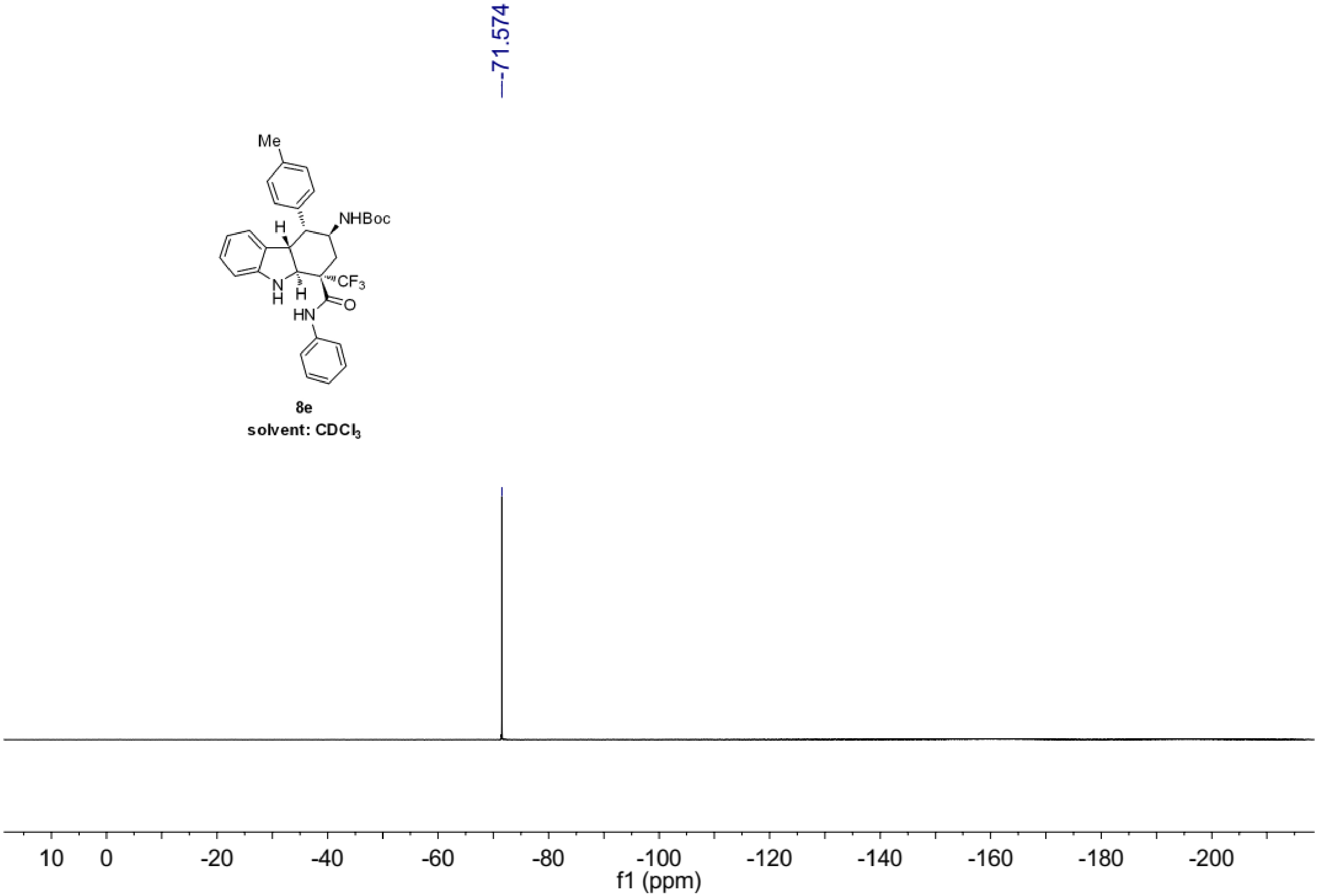

Supplementary Figure 151. ^{19}F NMR spectrum for compound **8e**

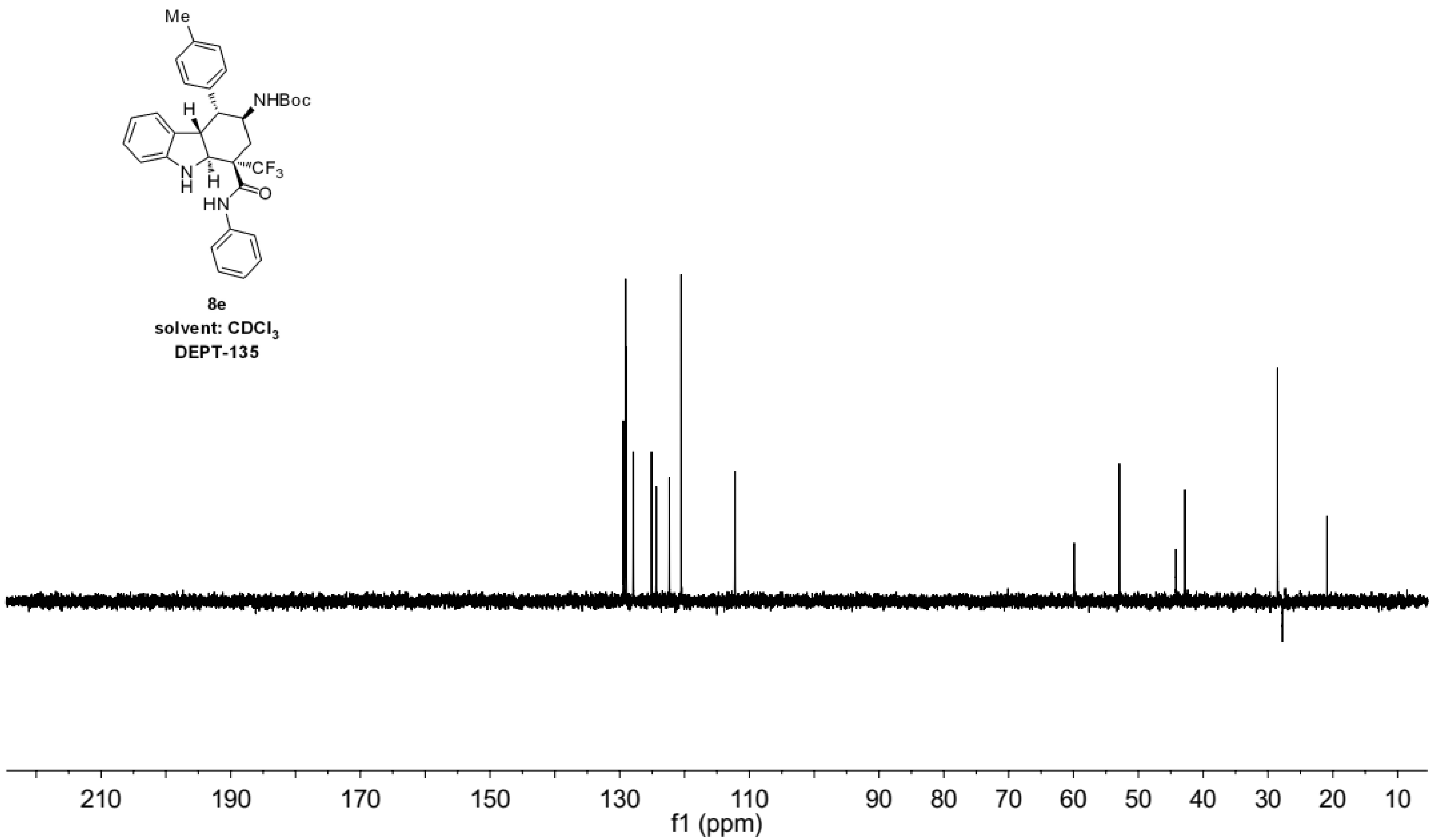

Supplementary Figure 152. DEPT-135 spectrum for compound **8e**

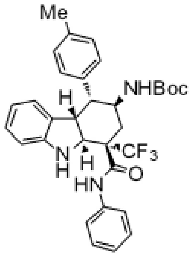

8e
solvent: CDCl$_3$
H-H COSY

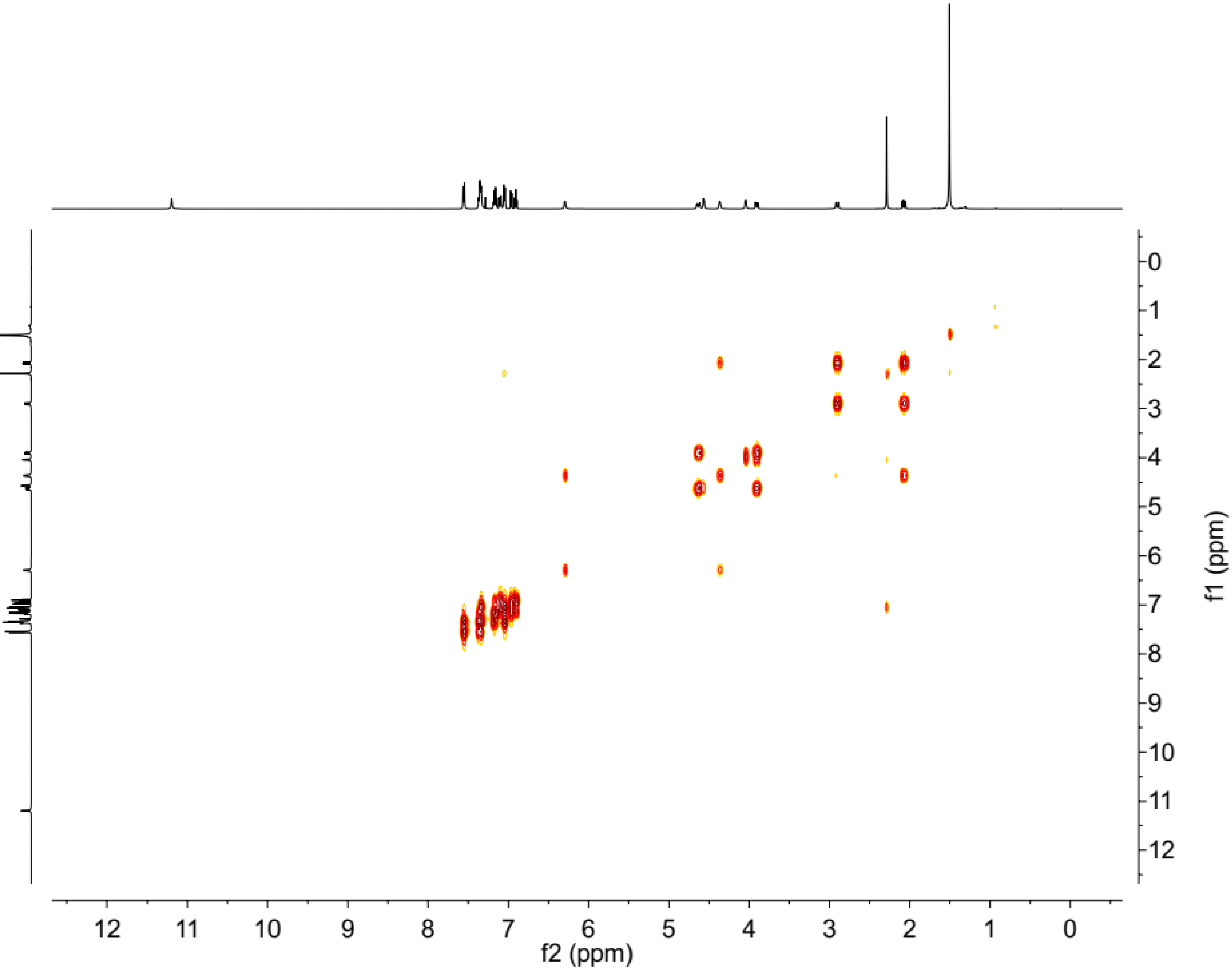

Supplementary Figure 153. H-H COSY spectrum for compound **8e**

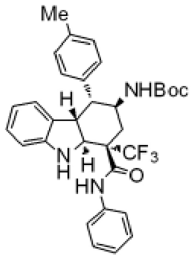

8e
solvent: $CDCl_3$
HMBC

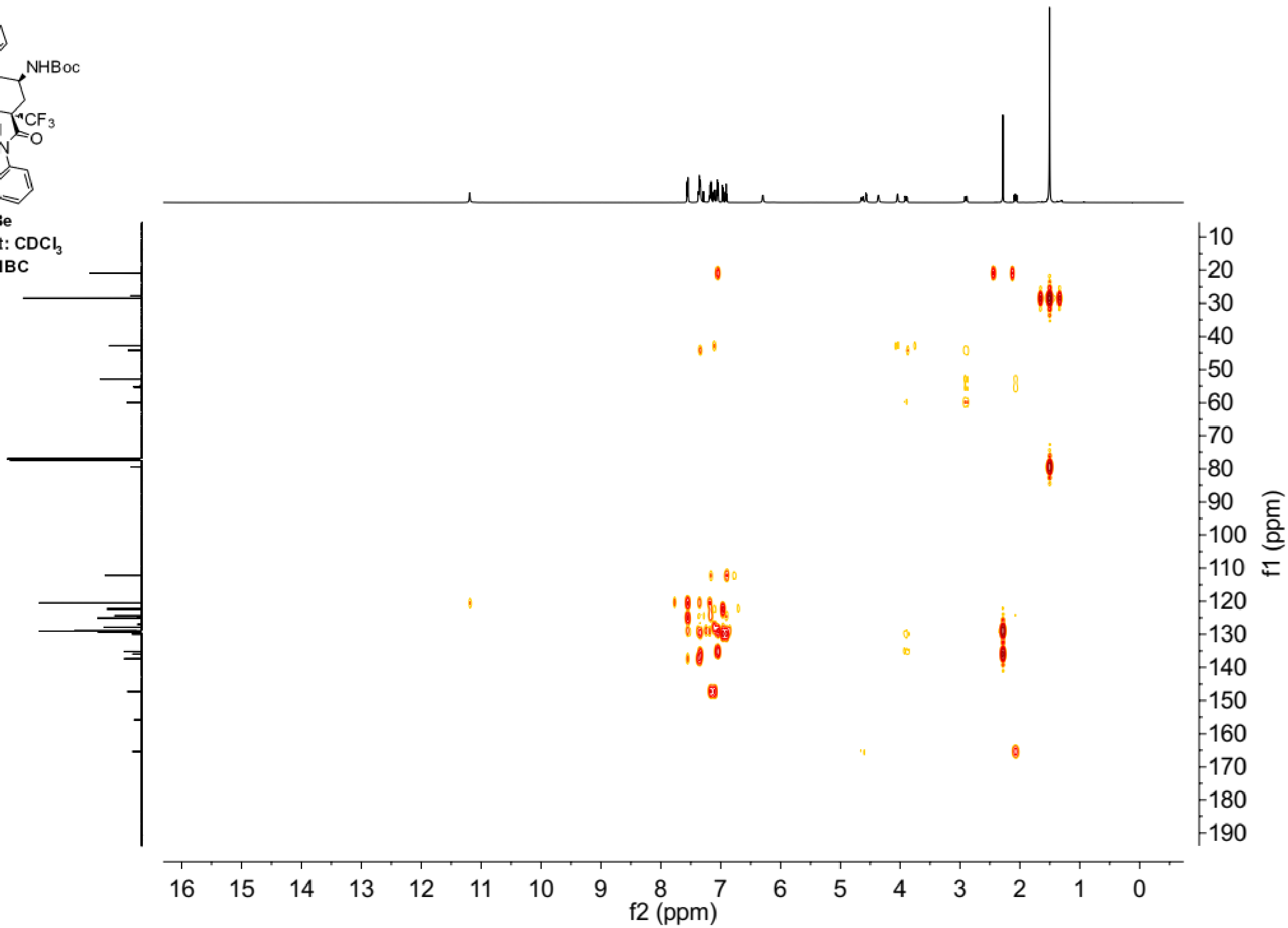

Supplementary Figure 154. HMBC spectrum for compound **8e**

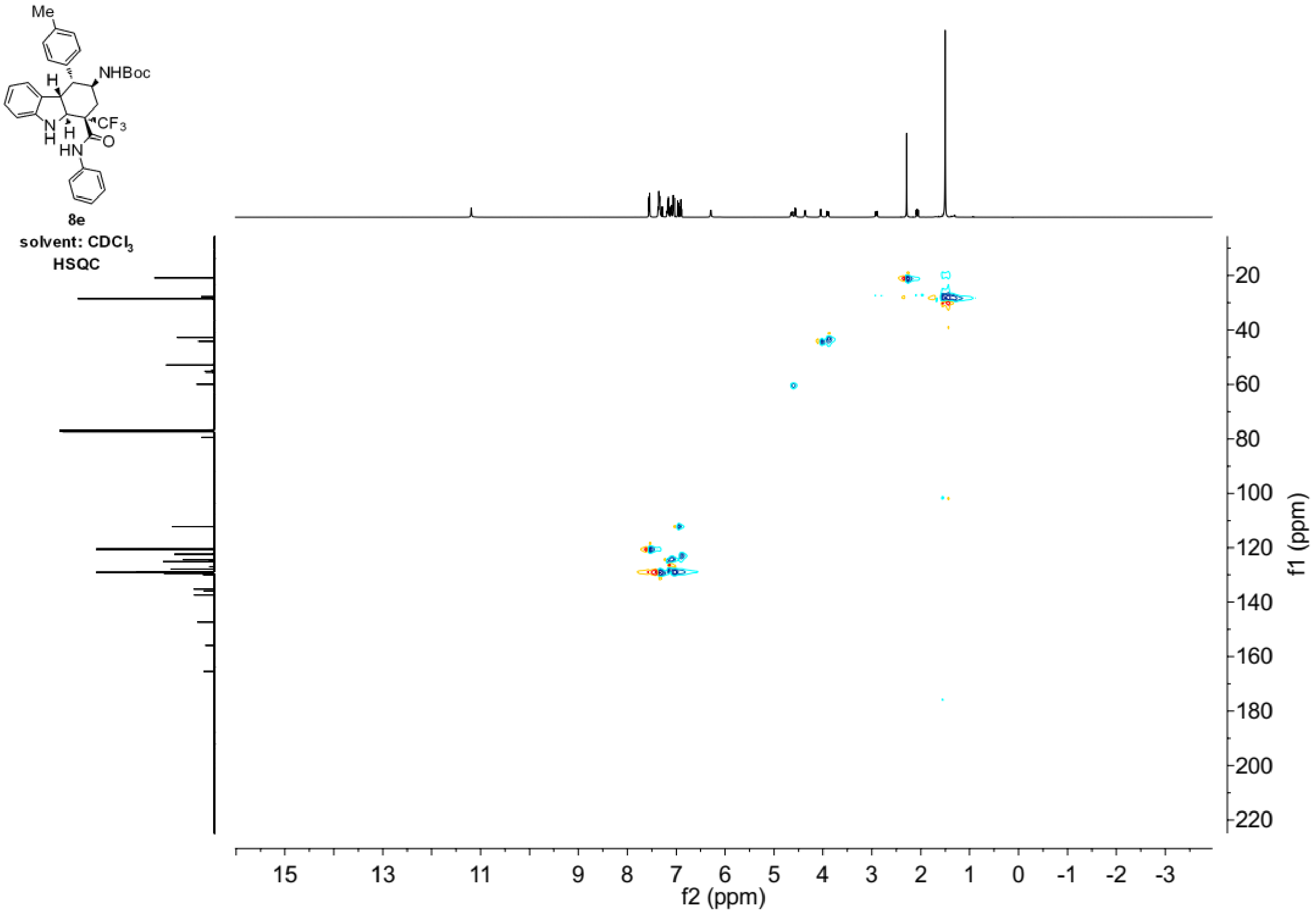

Supplementary Figure 155. HSQC spectrum for compound **8e**

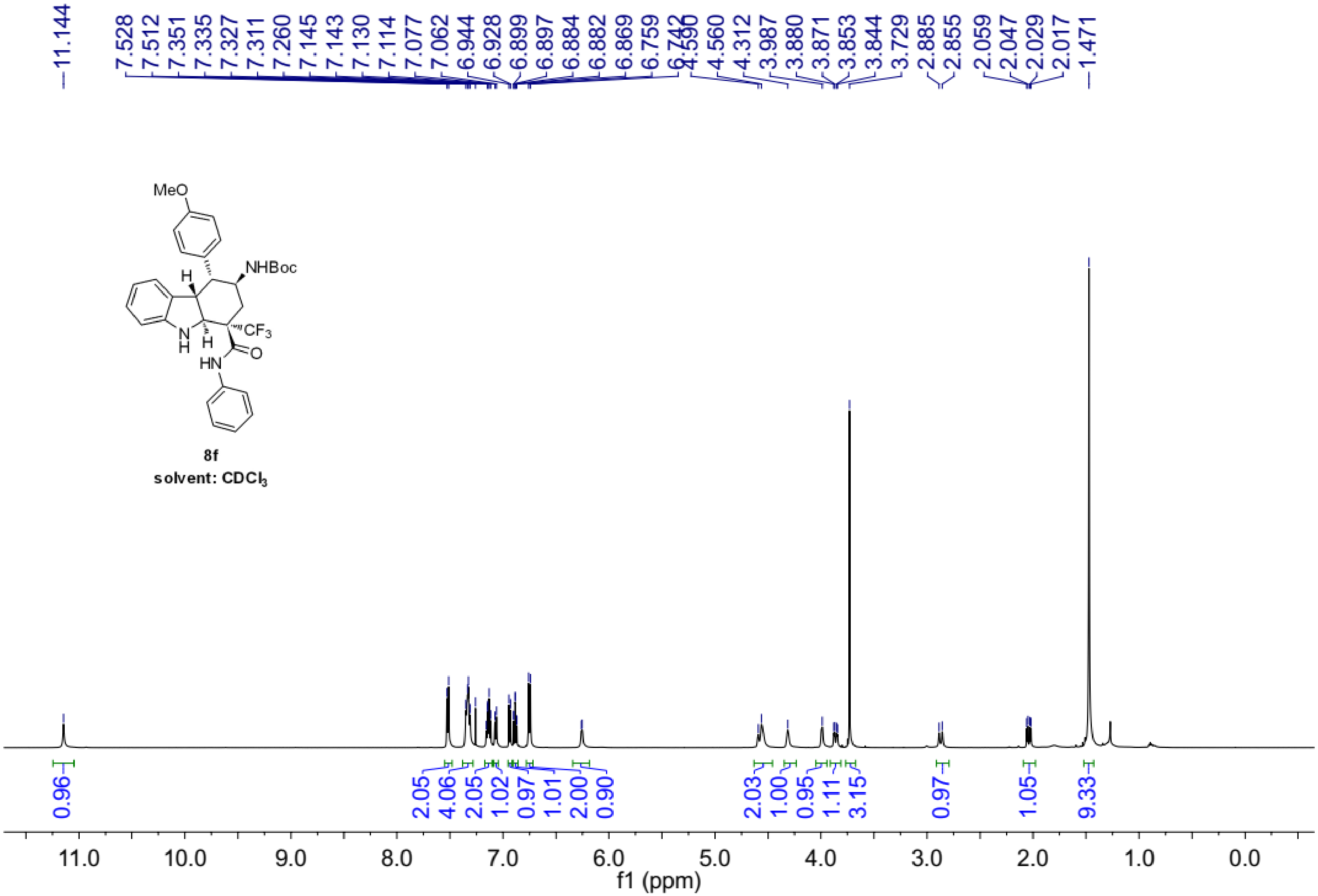

Supplementary Figure 156. ^{1}H NMR spectrum for compound **8f**

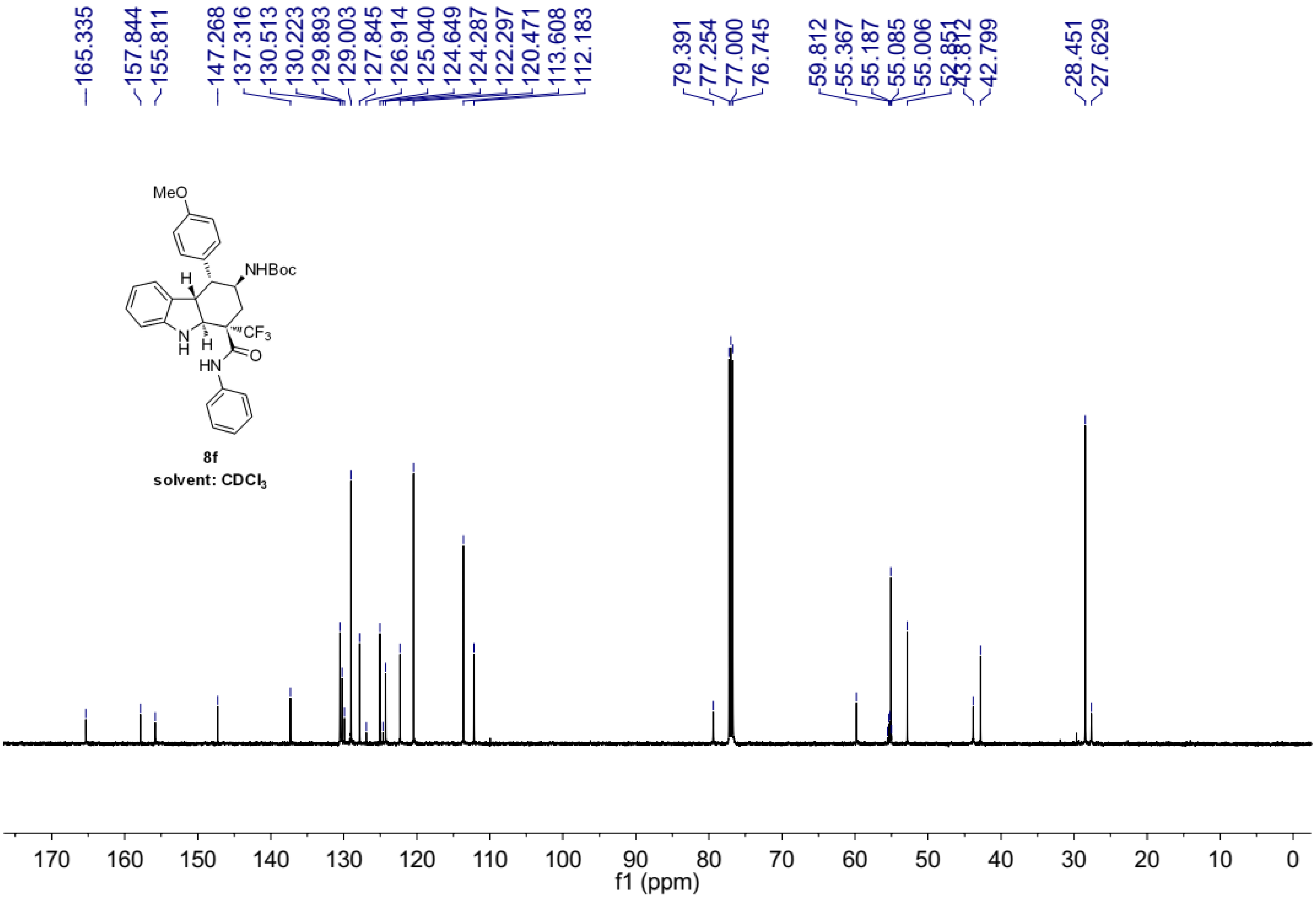

Supplementary Figure 157. ^{13}C NMR spectrum for compound **8f**

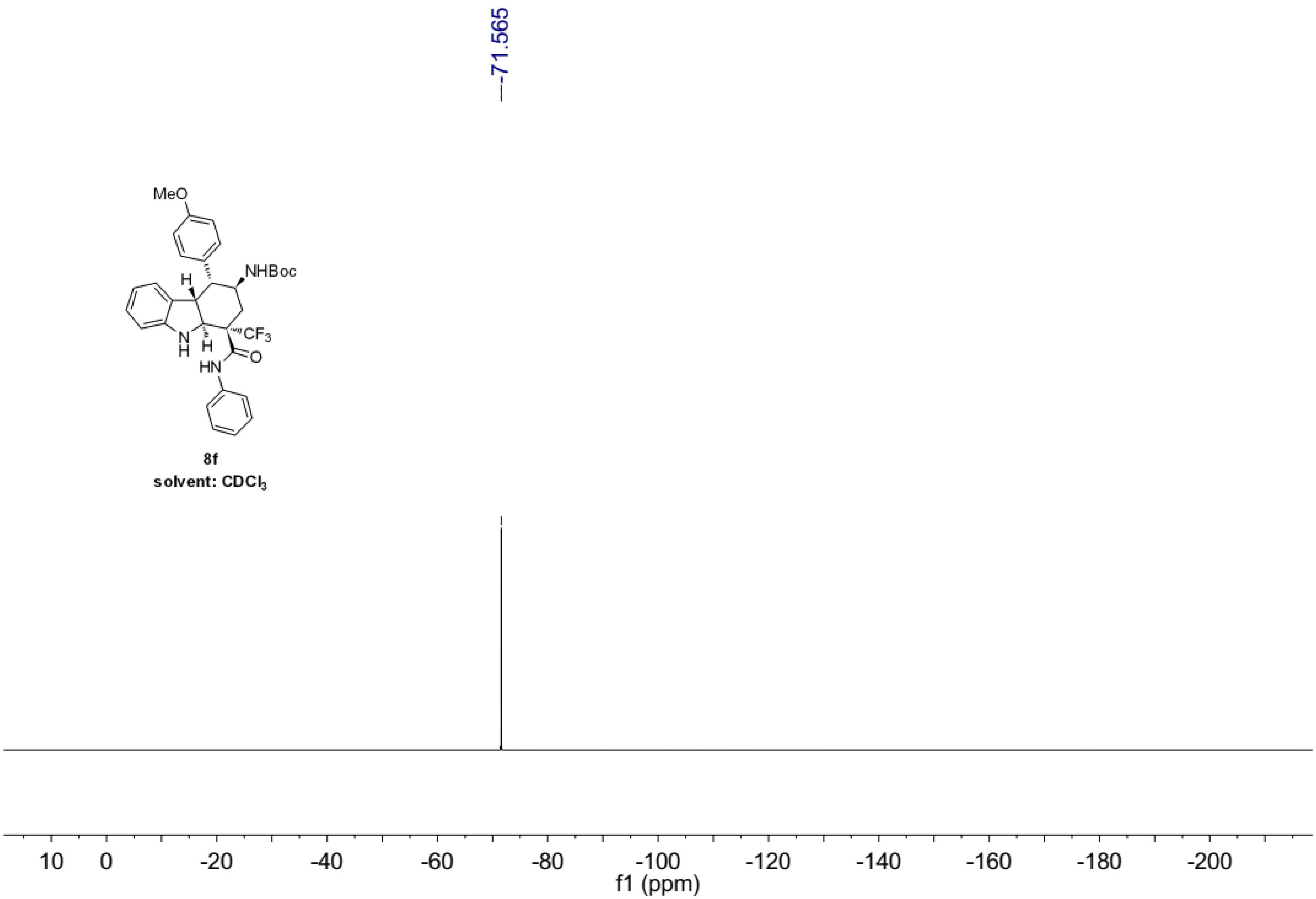

Supplementary Figure 158. ^{19}F NMR spectrum for compound **8f**

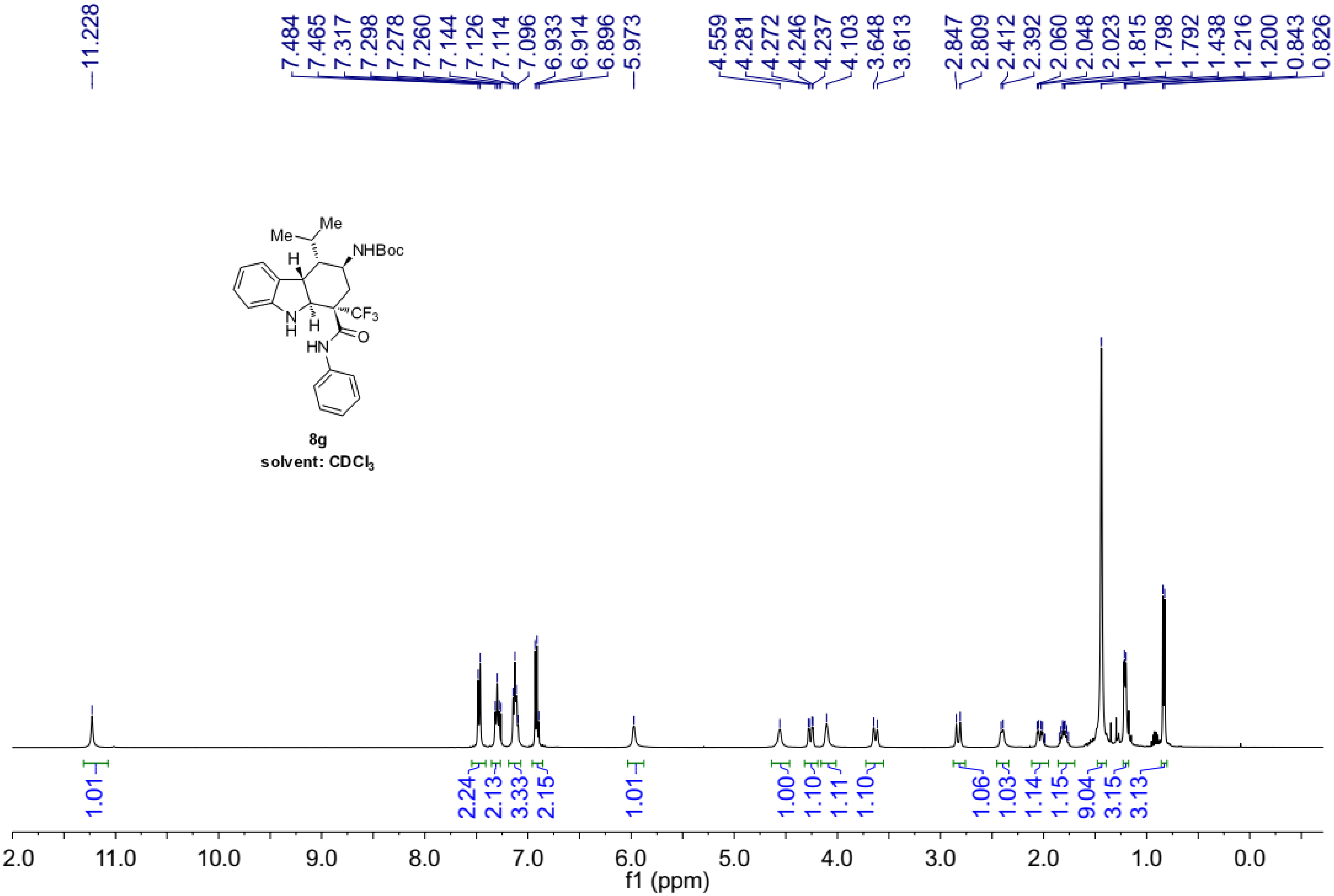

Supplementary Figure 159. ^{1}H NMR spectrum for compound **8g**

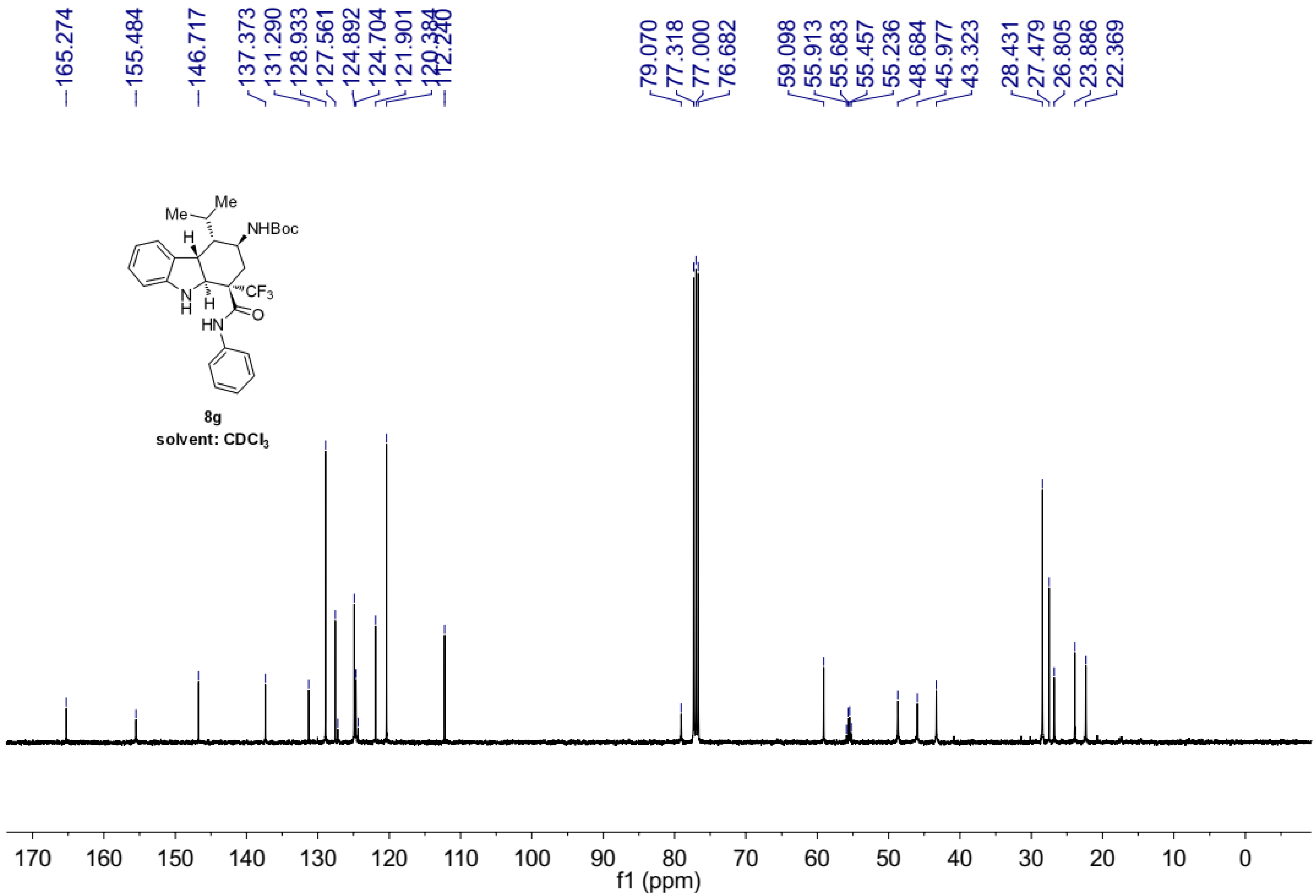

Supplementary Figure 160. ^{13}C NMR spectrum for compound **8g**

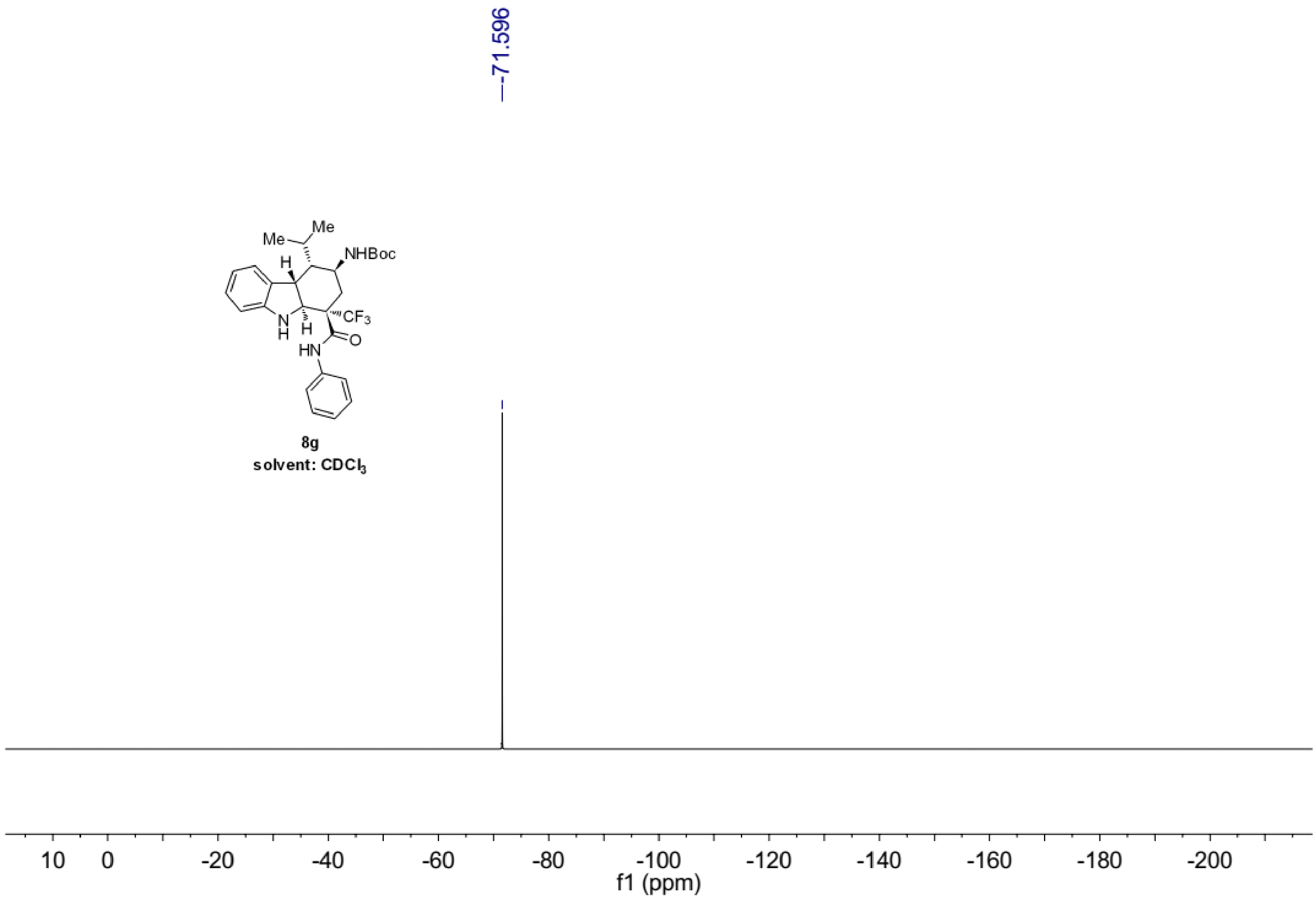

Supplementary Figure 161. ^{19}F NMR spectrum for compound **8g**

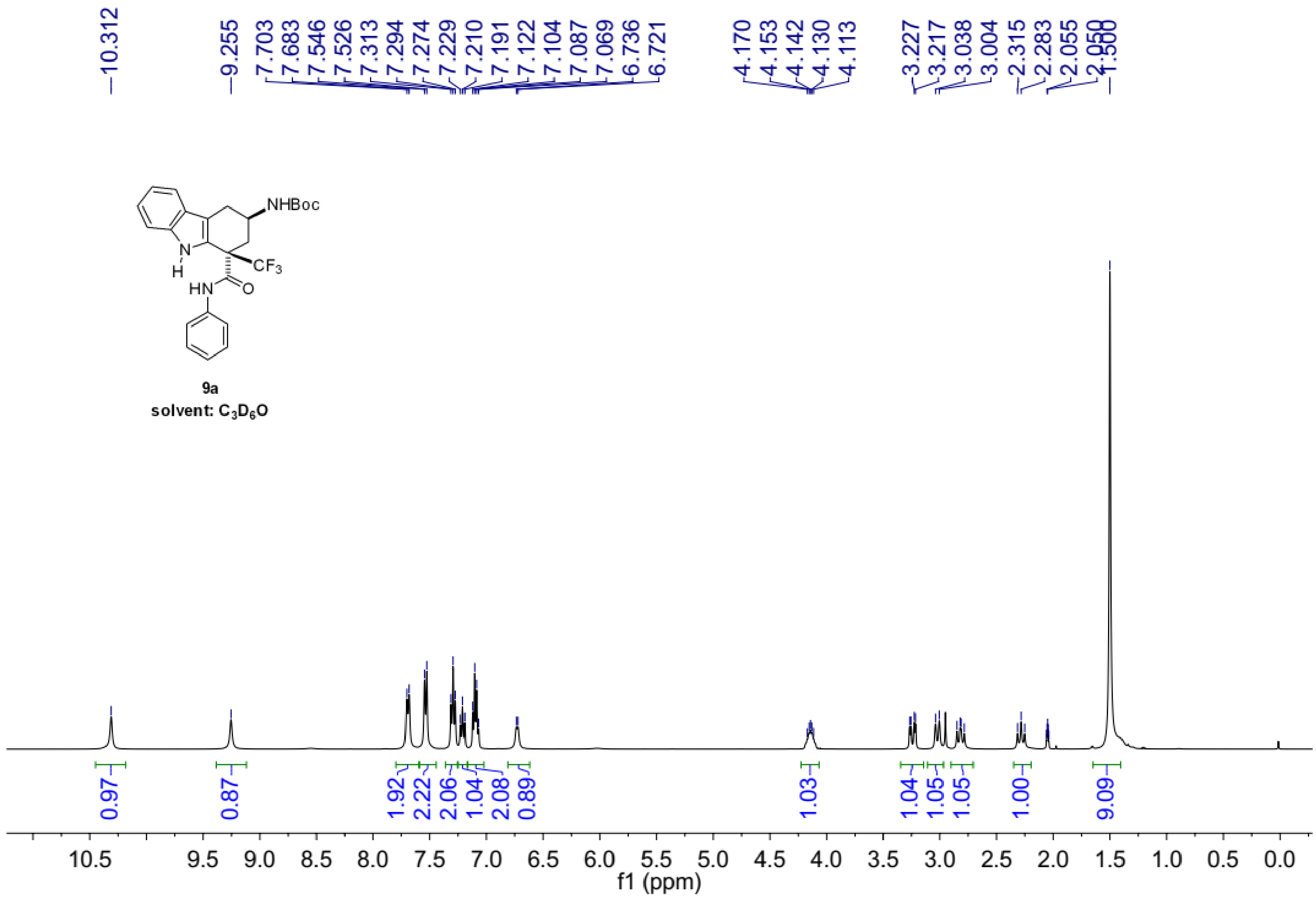

Supplementary Figure 162. ^{1}H NMR spectrum for compound **9a**

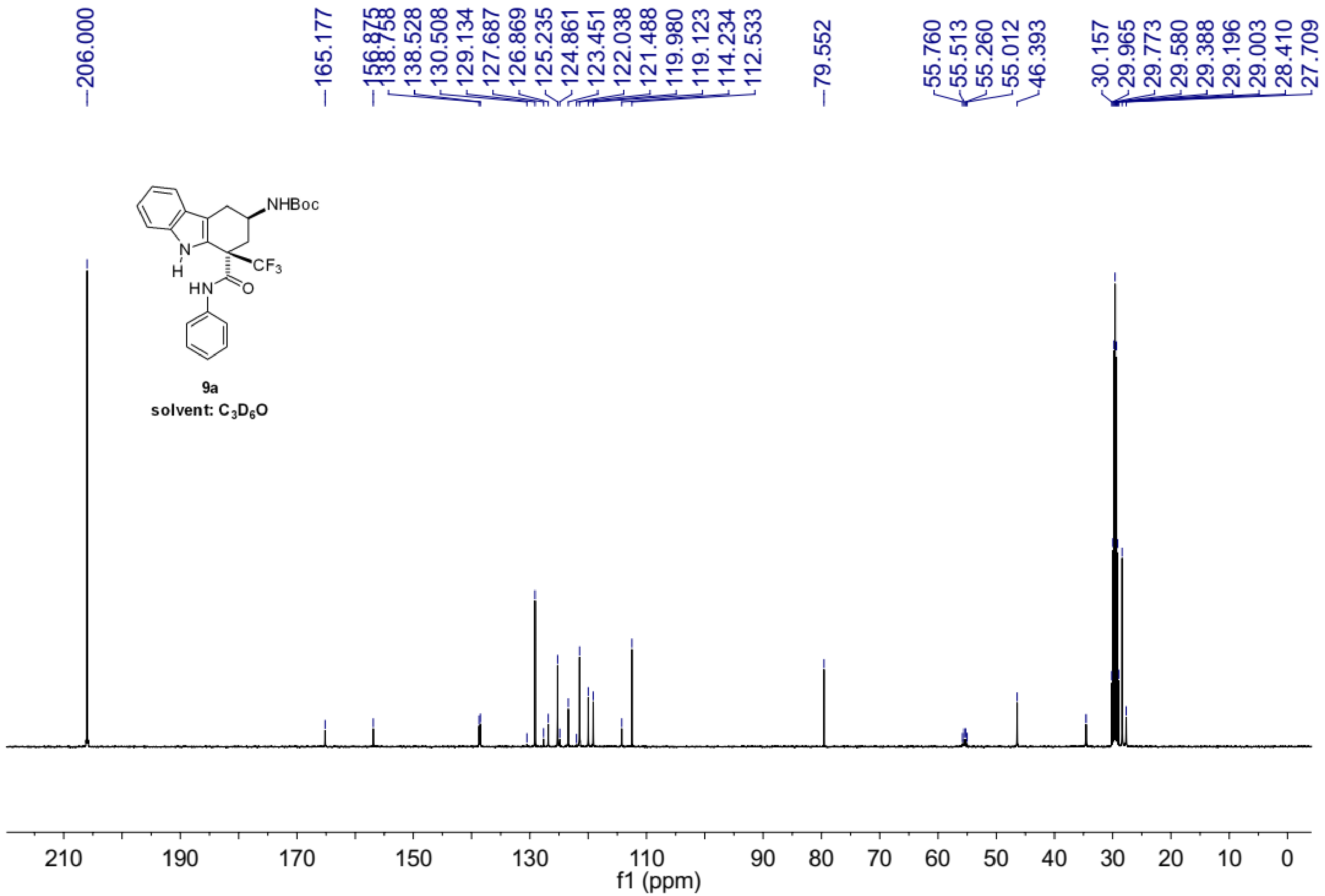

Supplementary Figure 163. ^{13}C NMR spectrum for compound **9a**

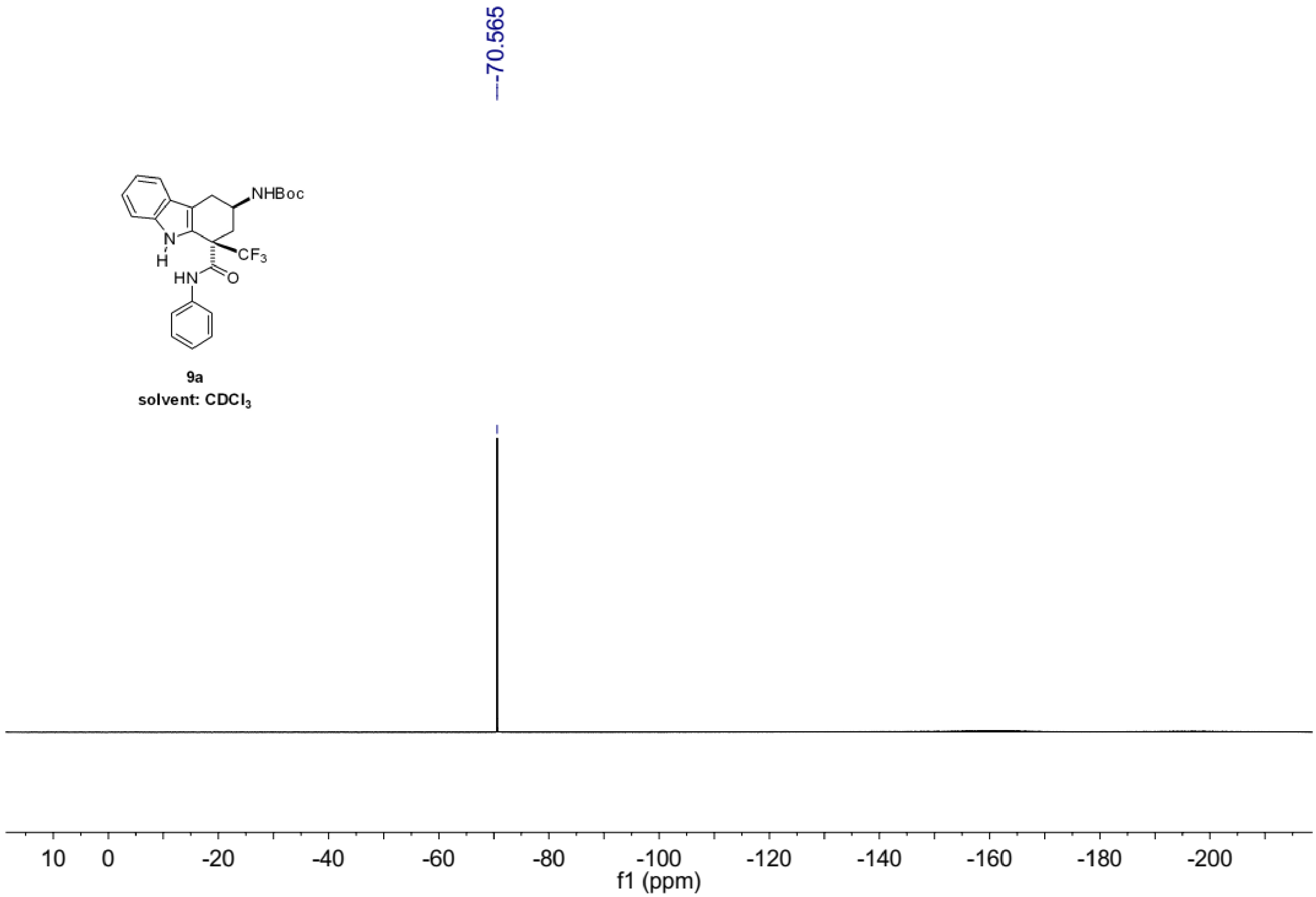

Supplementary Figure 164. ^{19}F NMR spectrum for compound **9a**

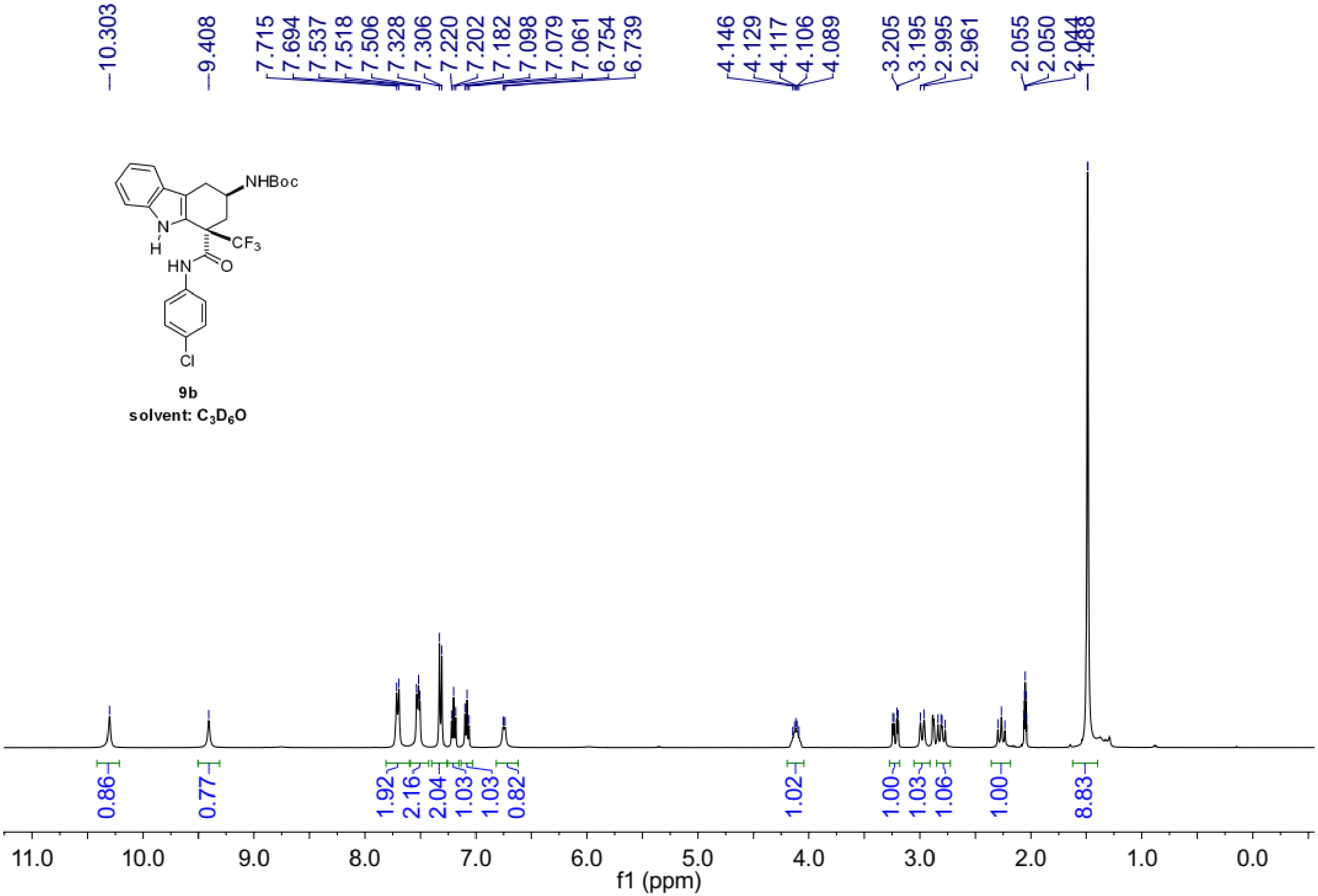

Supplementary Figure 165. ^{1}H NMR spectrum for compound **9b**

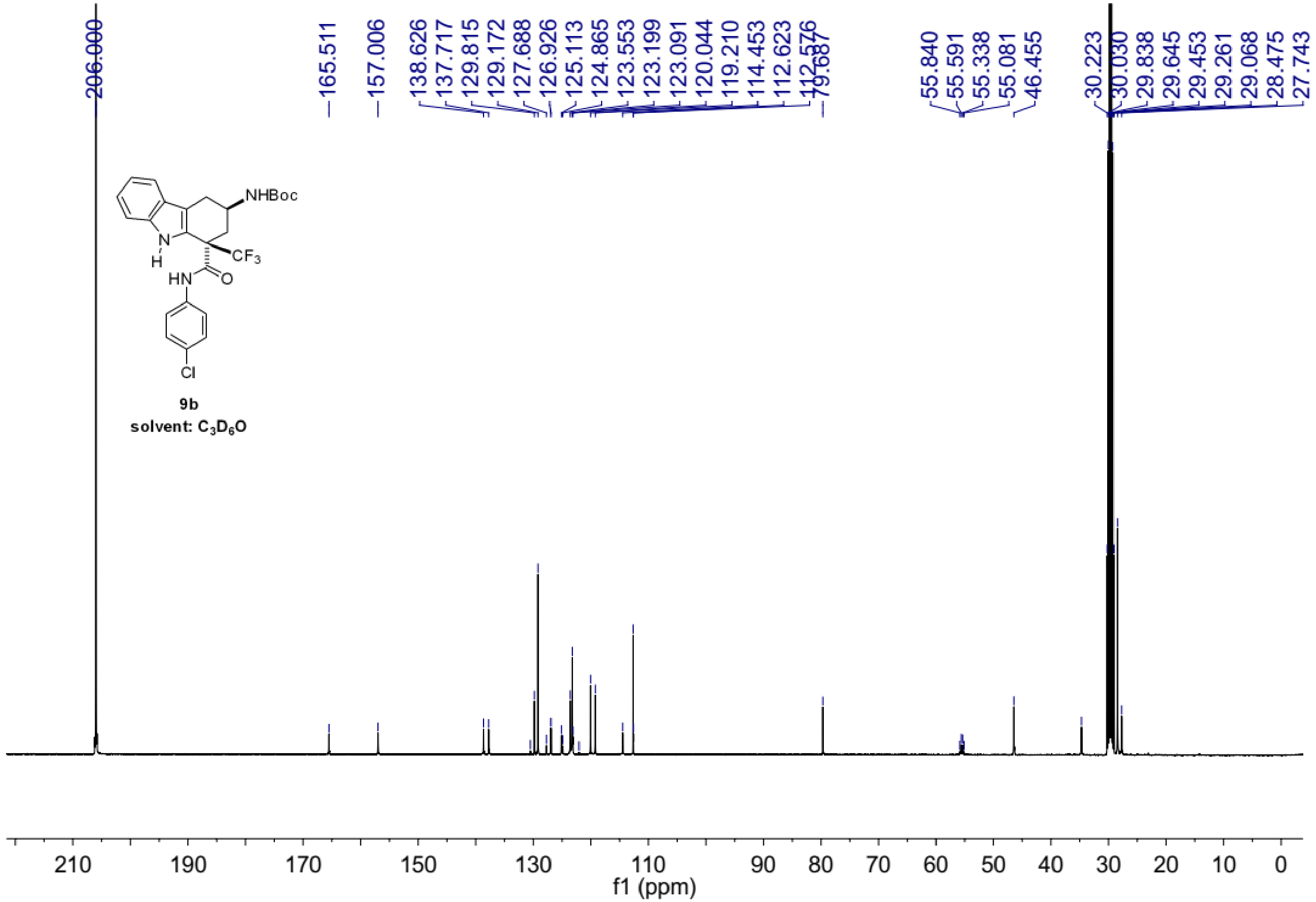

Supplementary Figure 166. ^{13}C NMR spectrum for compound **9b**

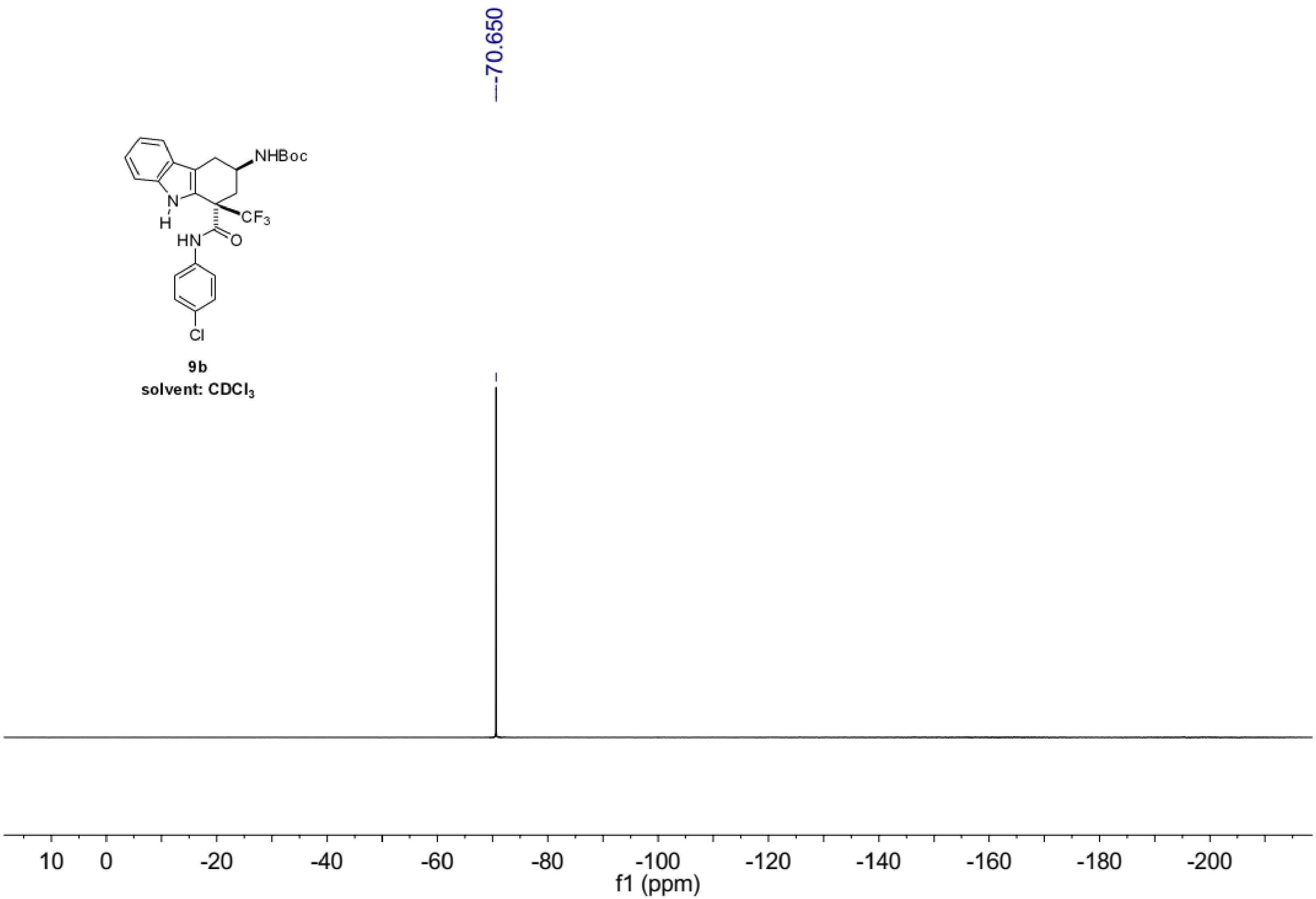

Supplementary Figure 167. ^{19}F NMR spectrum for compound **9b**

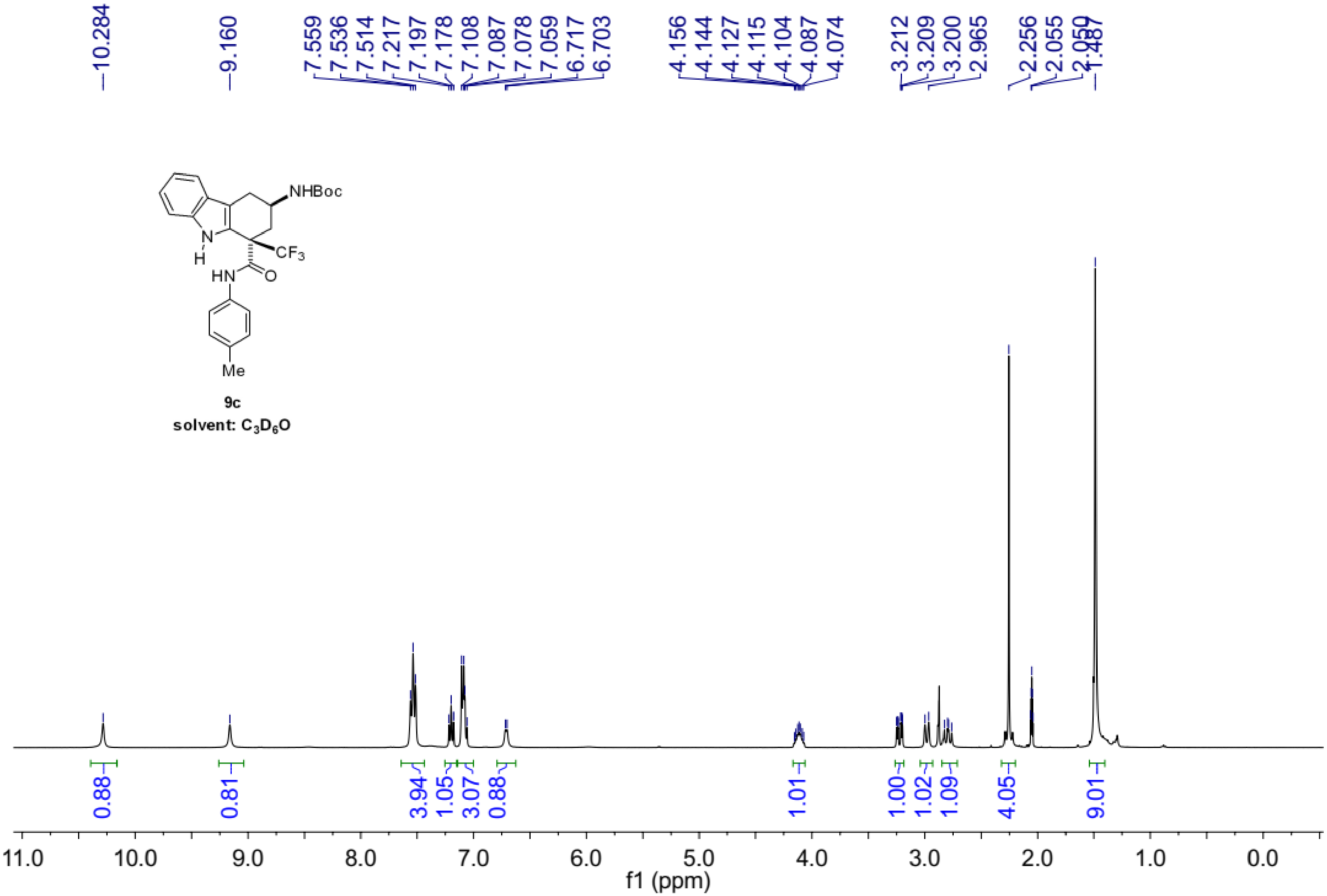

Supplementary Figure 168. ^{1}H NMR spectrum for compound **9c**

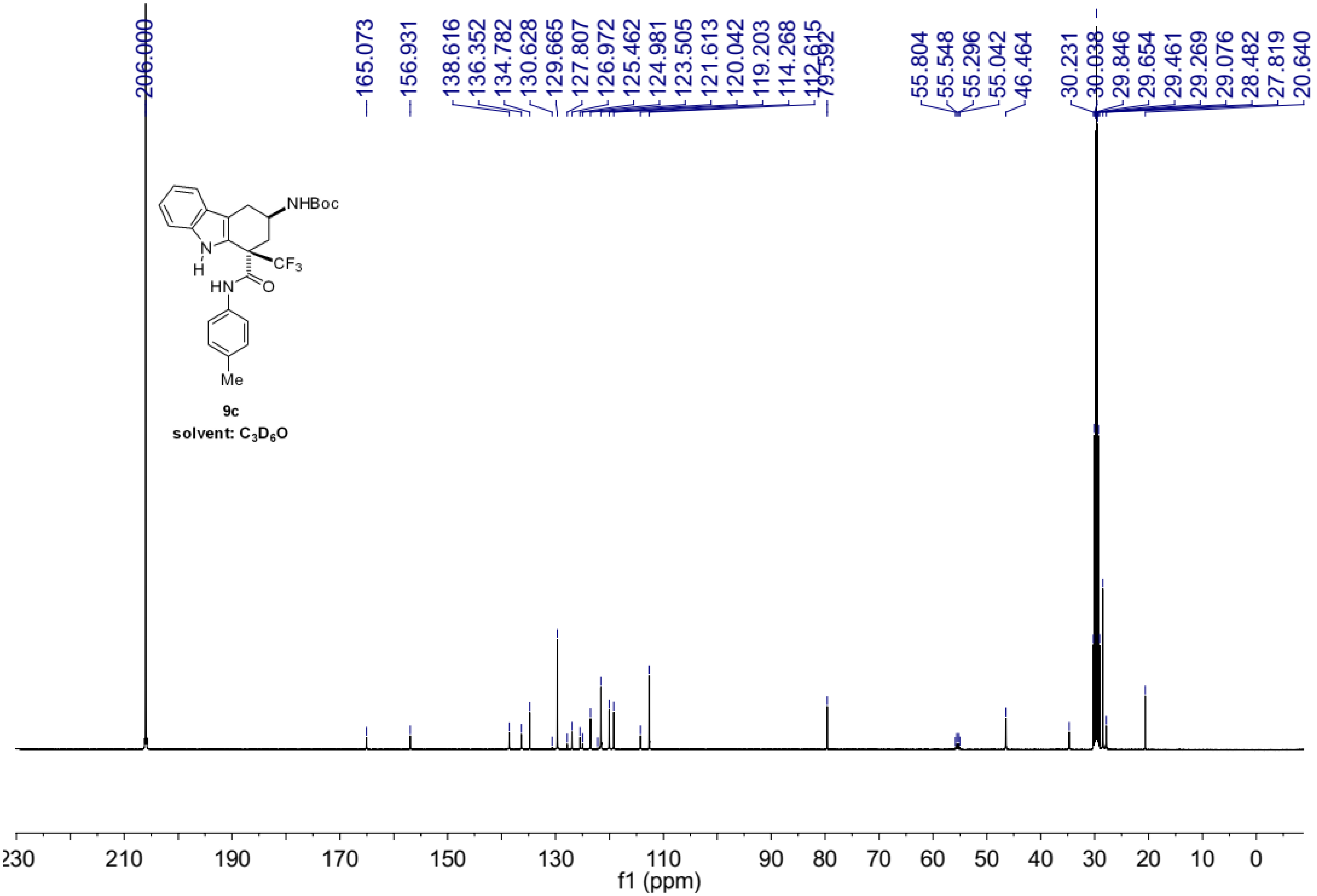

Supplementary Figure 169. ^{13}C NMR spectrum for compound **9c**

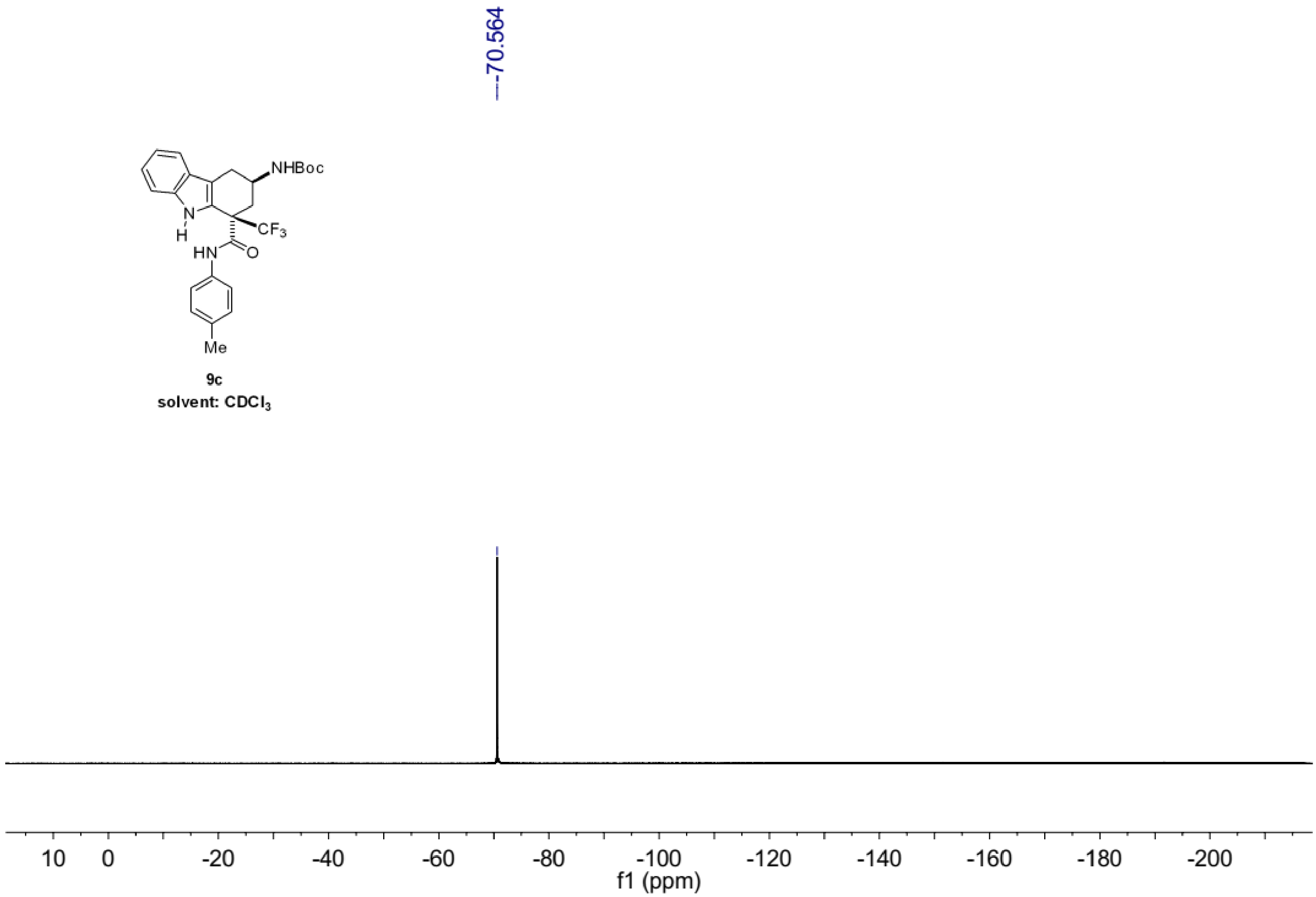

9c
solvent: $CDCl_3$

Supplementary Figure 170. ^{19}F NMR spectrum for compound **9c**

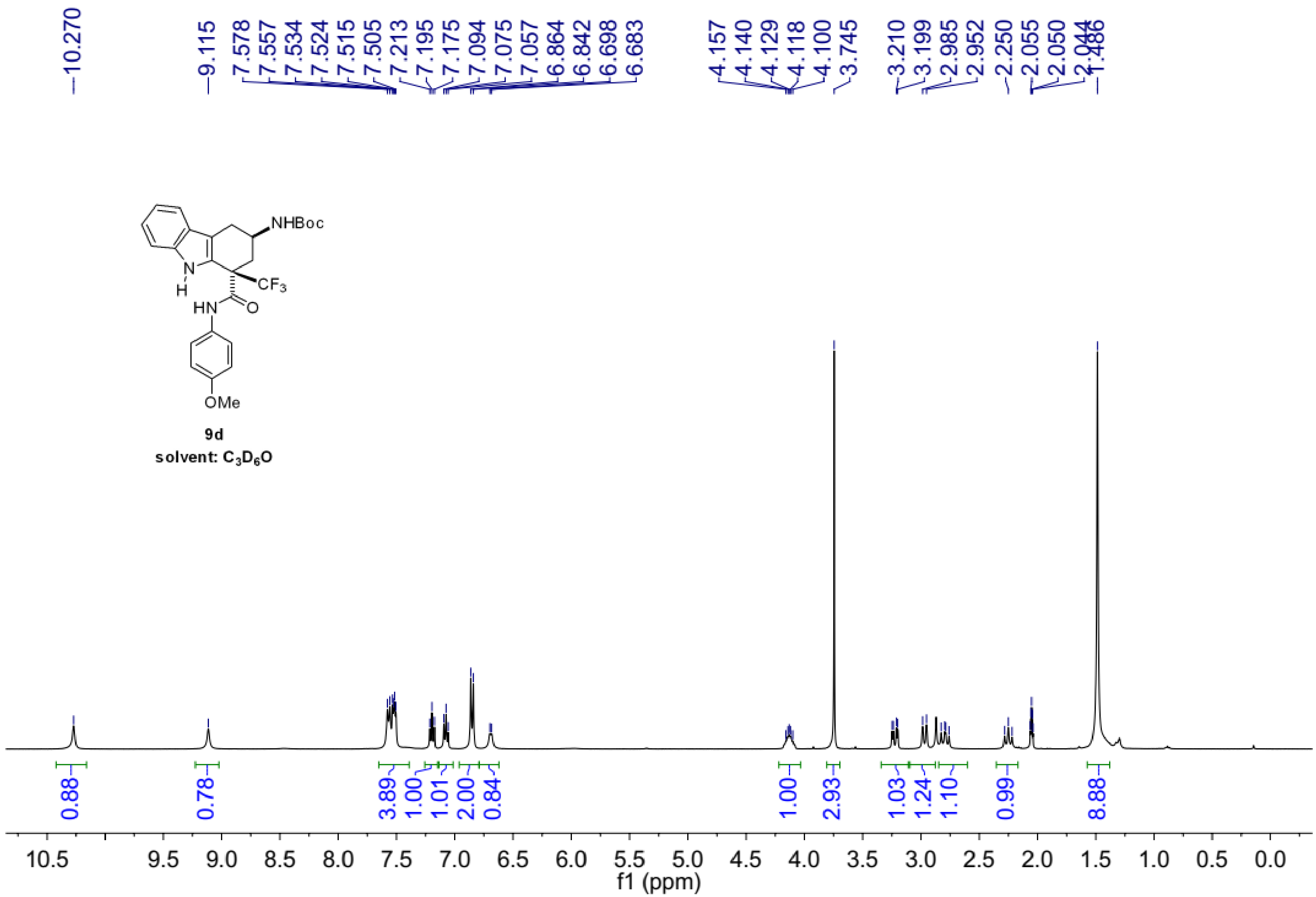

Supplementary Figure 171. ^{1}H NMR spectrum for compound **9d**

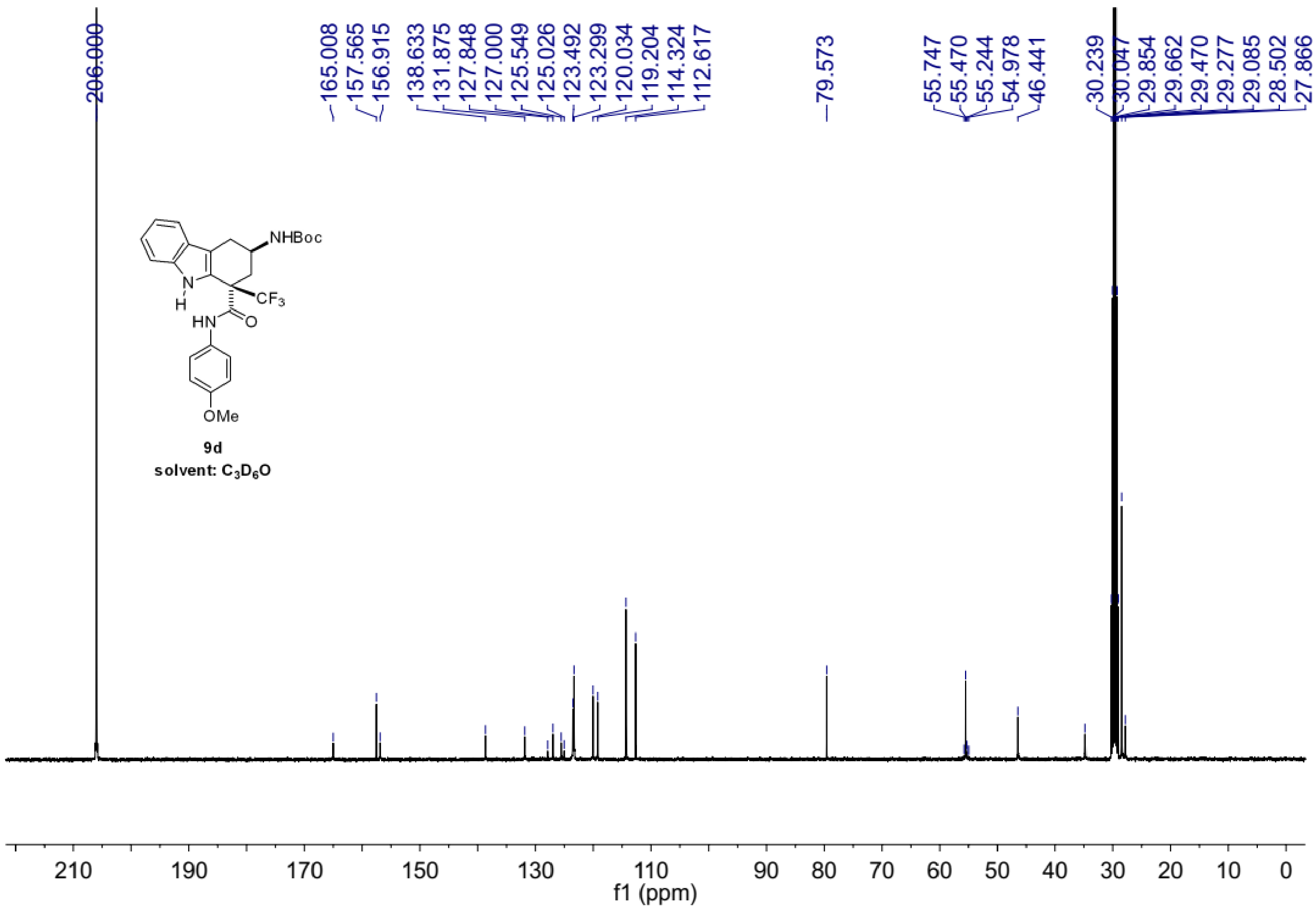

Supplementary Figure 172. ^{13}C NMR spectrum for compound **9d**

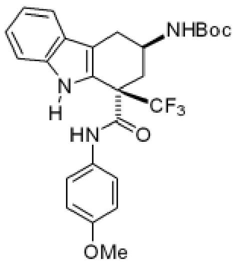

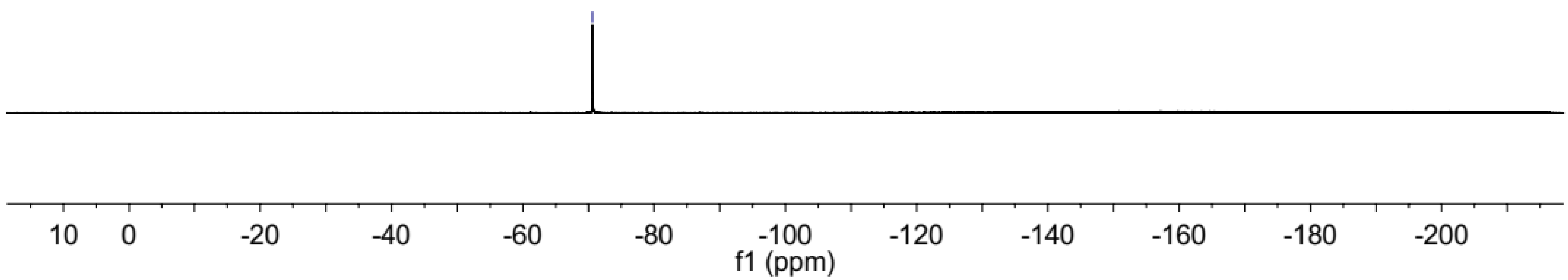

Supplementary Figure 173. ^{19}F NMR spectrum for compound **9d**

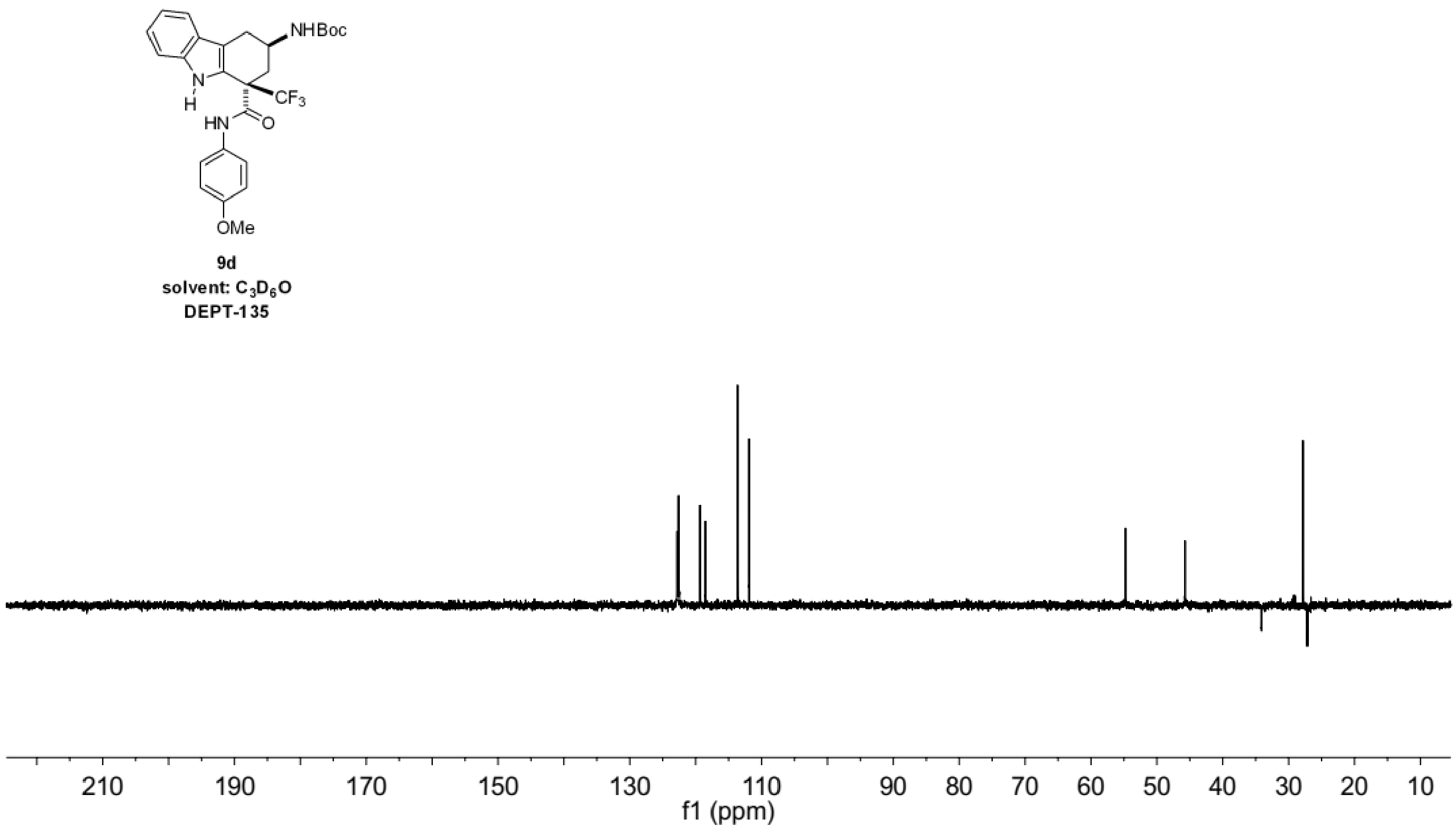

Supplementary Figure 174. DEPT-135 spectrum for compound **9d**

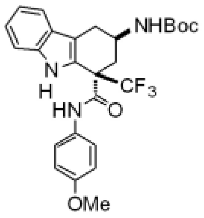

9d
solvent: C_3D_6O
H-H COSY

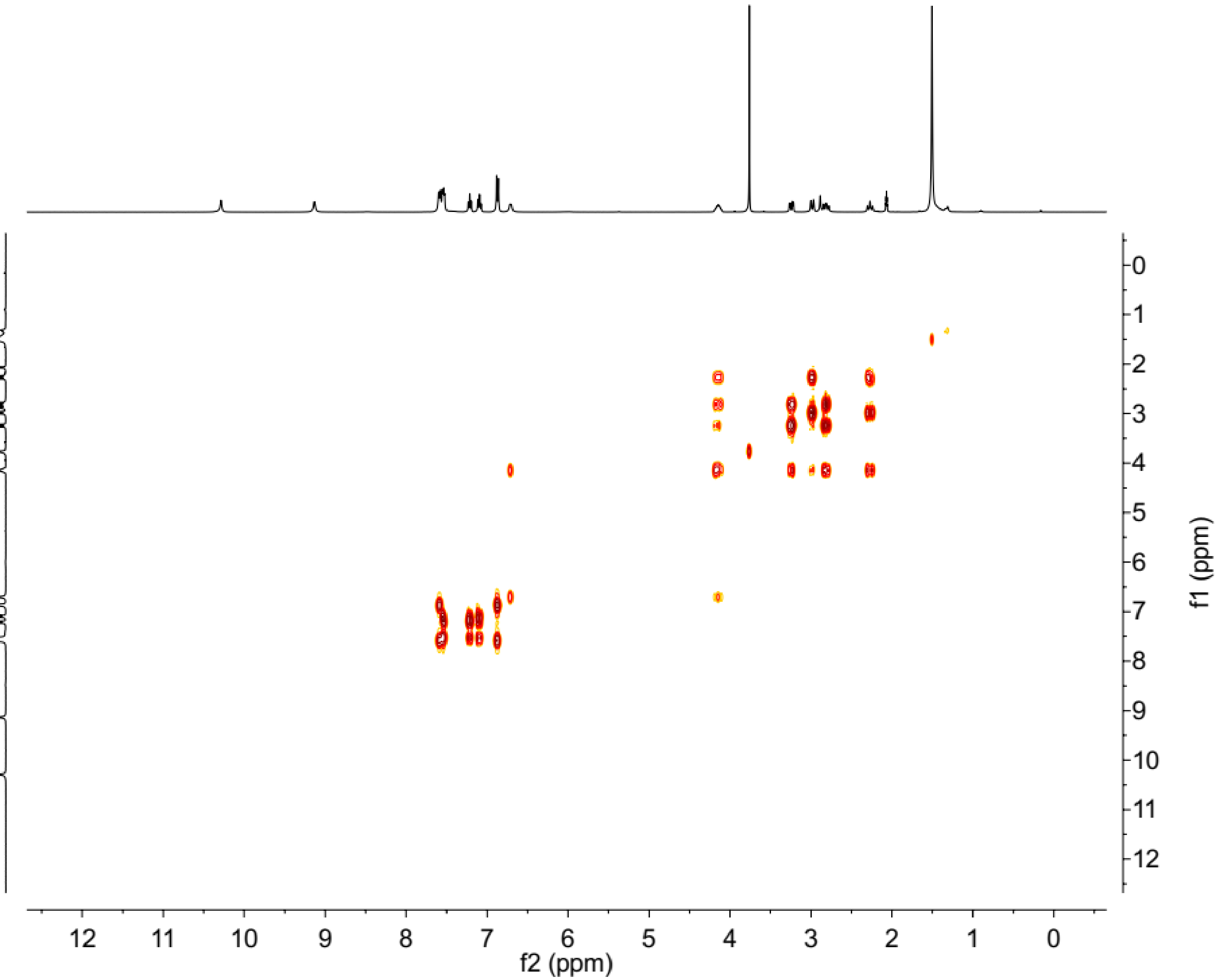

Supplementary Figure 175. H-H COSY spectrum for compound **9d**

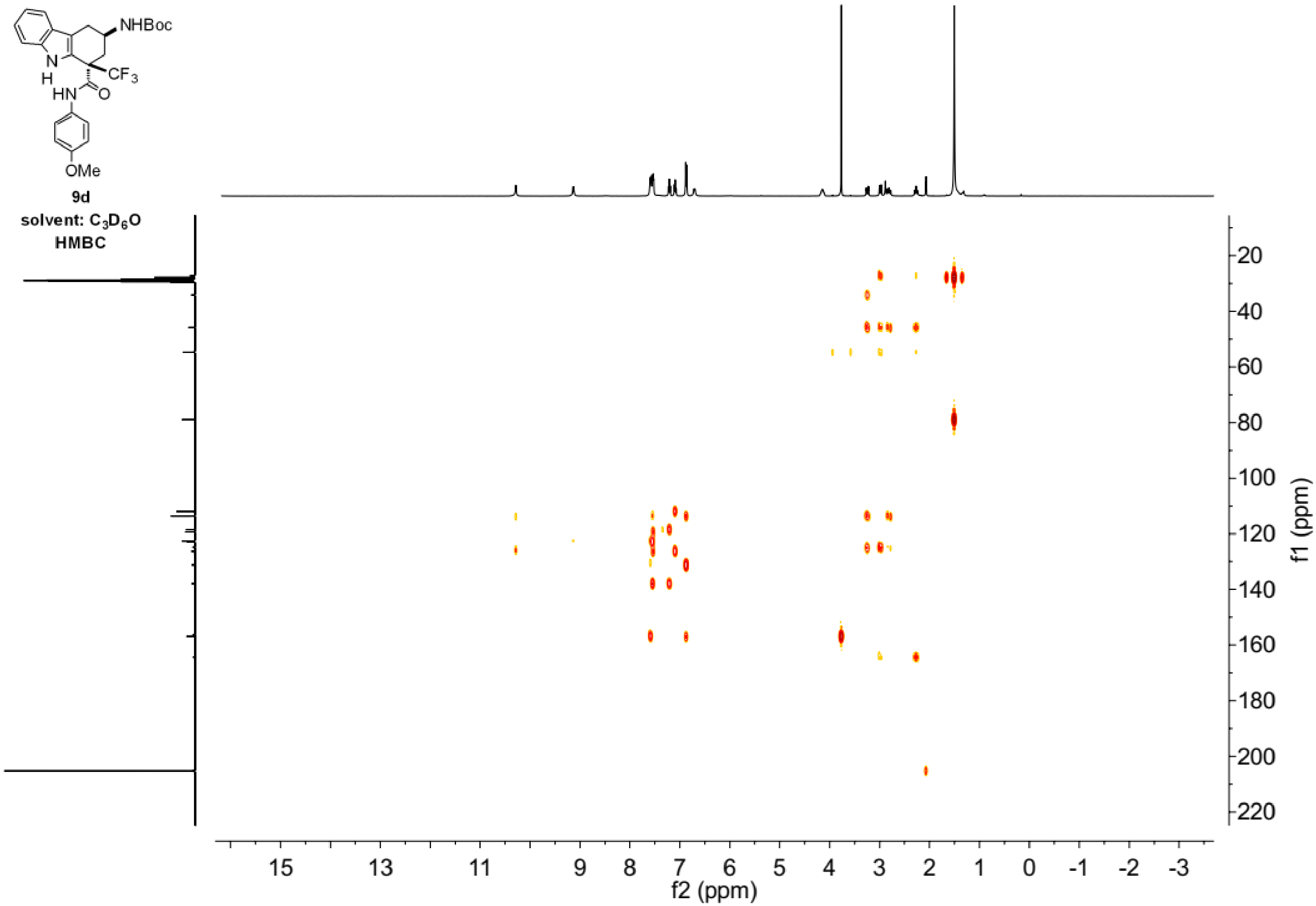

Supplementary Figure 176. HMBC spectrum for compound **9d**

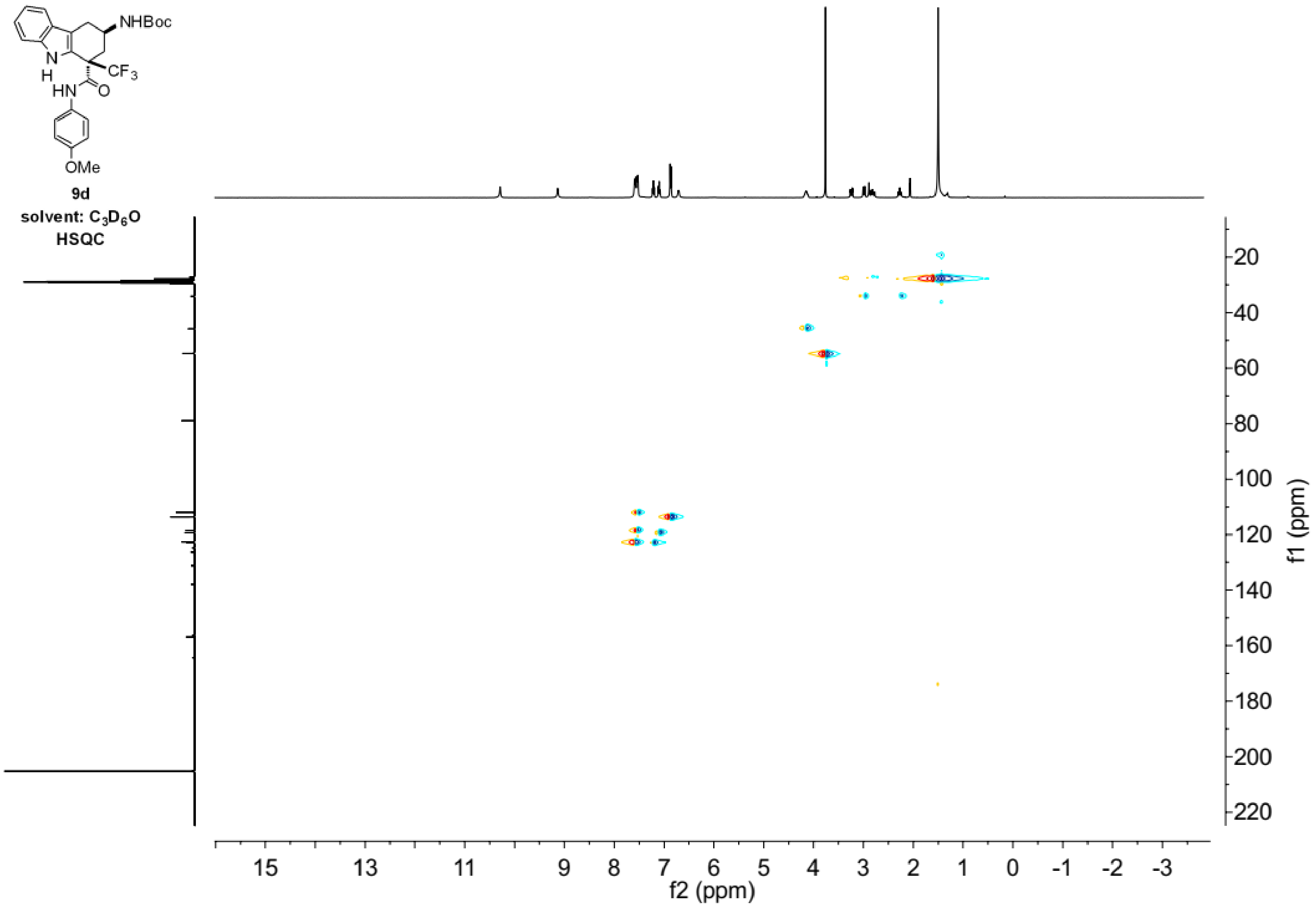

Supplementary Figure 177. HSQC spectrum for compound **9d**

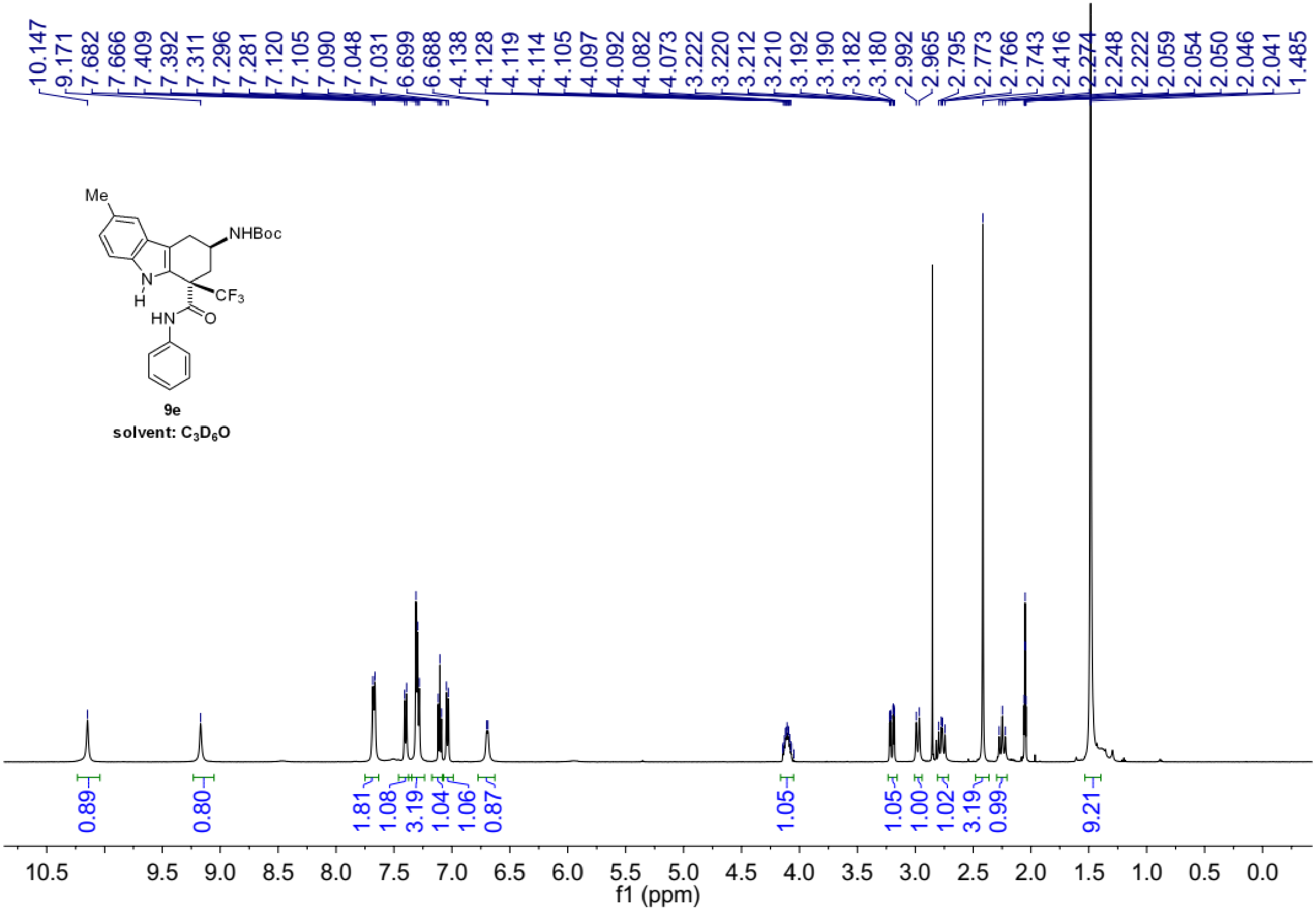

Supplementary Figure 178. ^{1}H NMR spectrum for compound **9e**

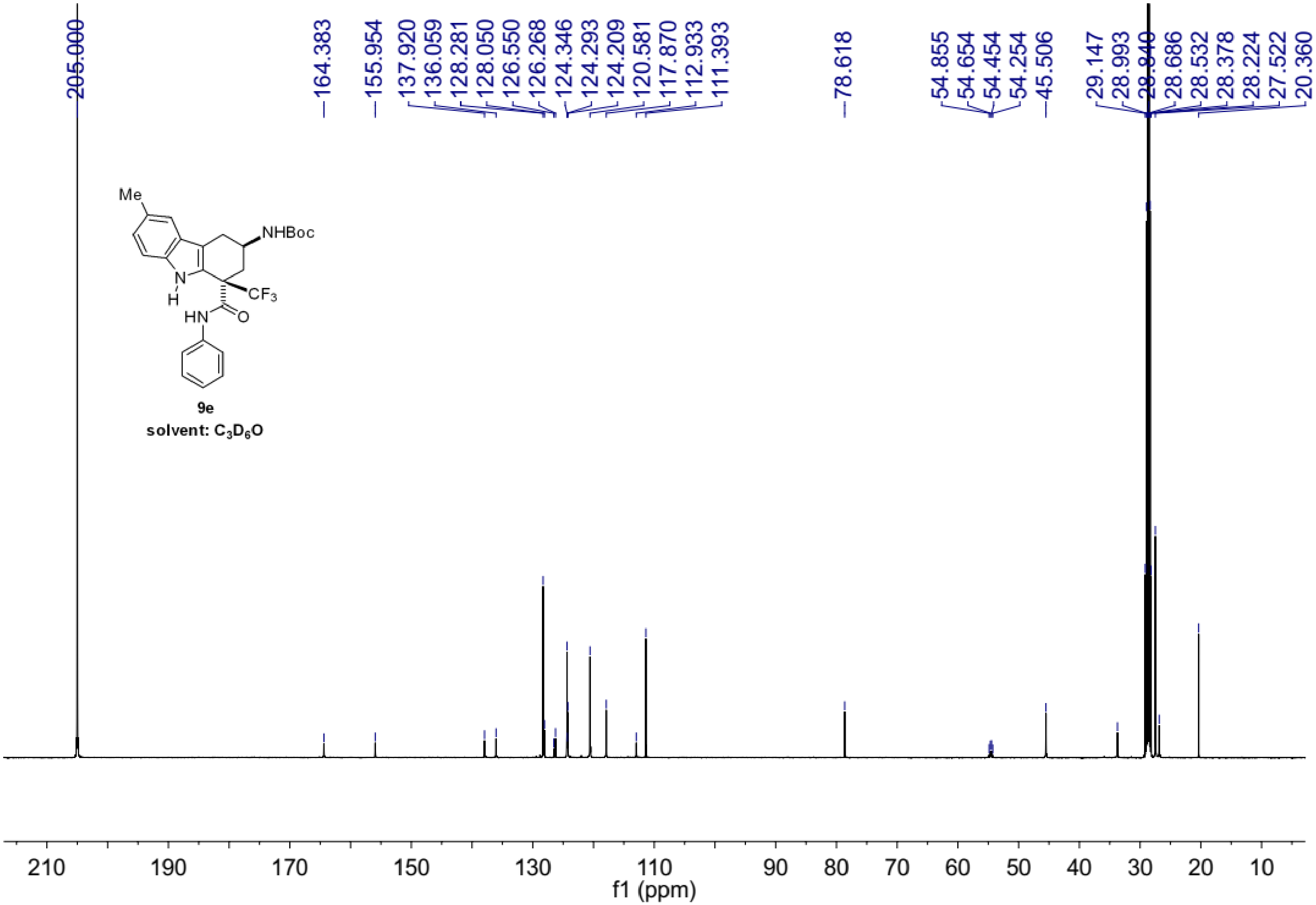

Supplementary Figure 179. ^{13}C NMR spectrum for compound **9e**

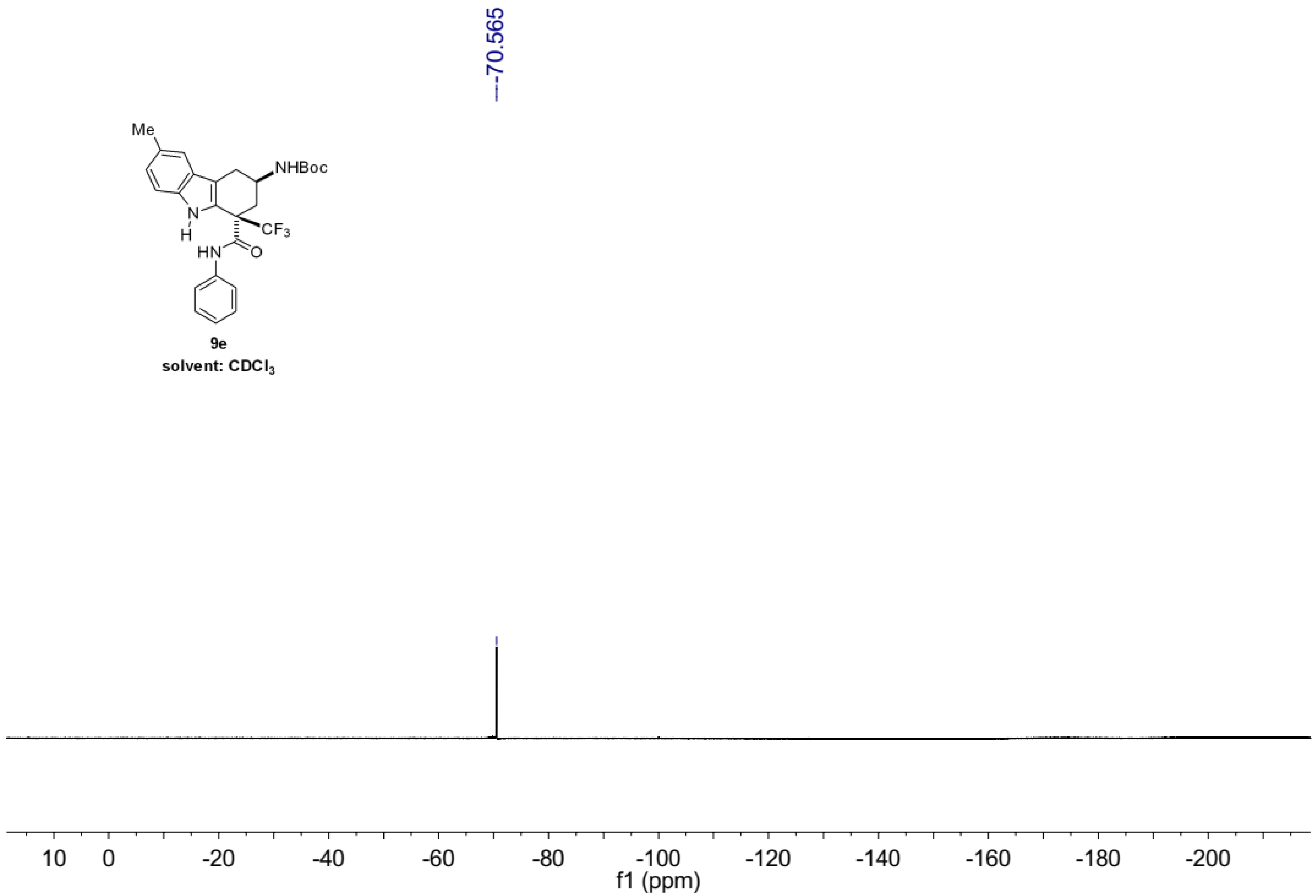

Supplementary Figure 180. ^{19}F NMR spectrum for compound **9e**

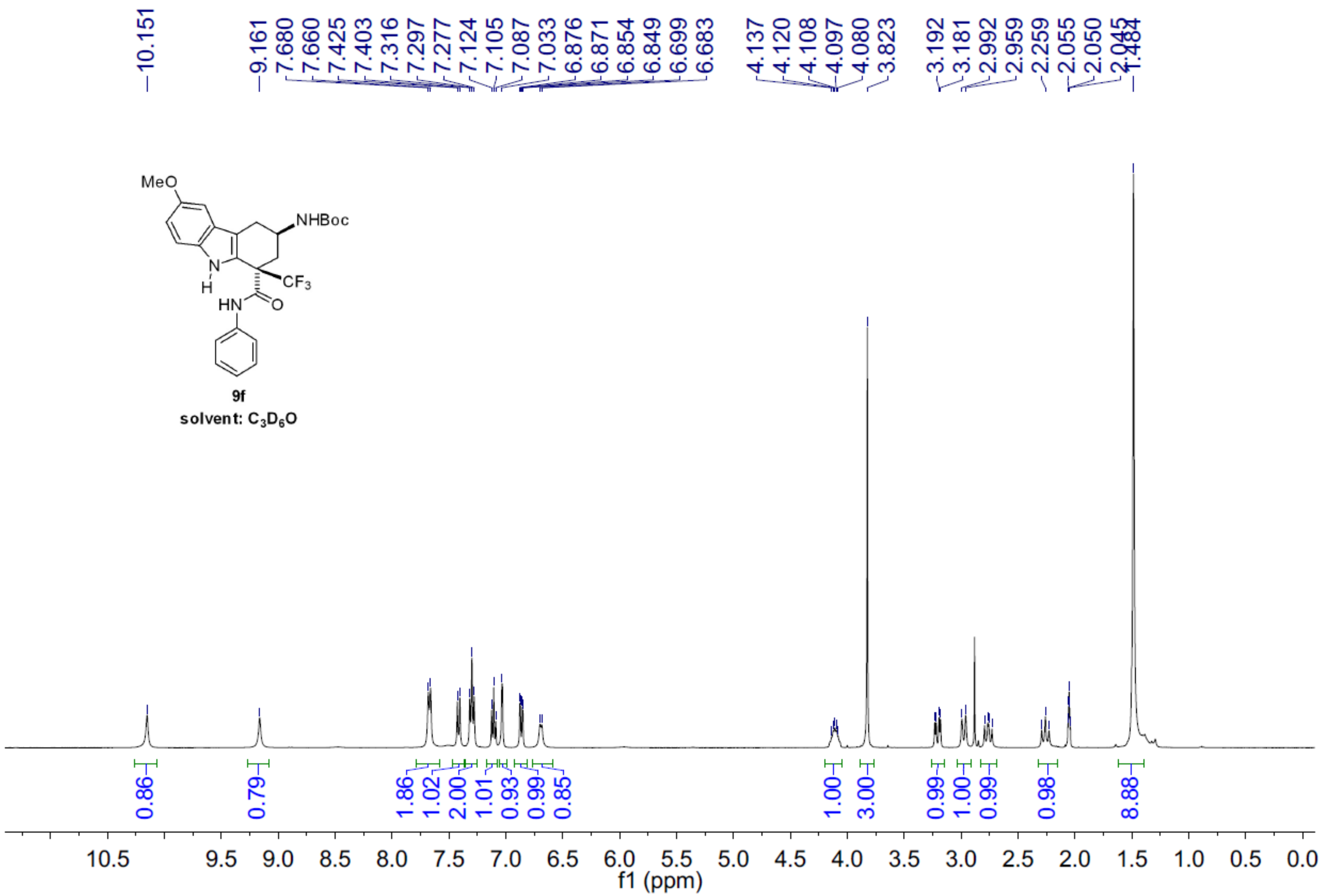

Supplementary Figure 181. ^{1}H NMR spectrum for compound **9f**

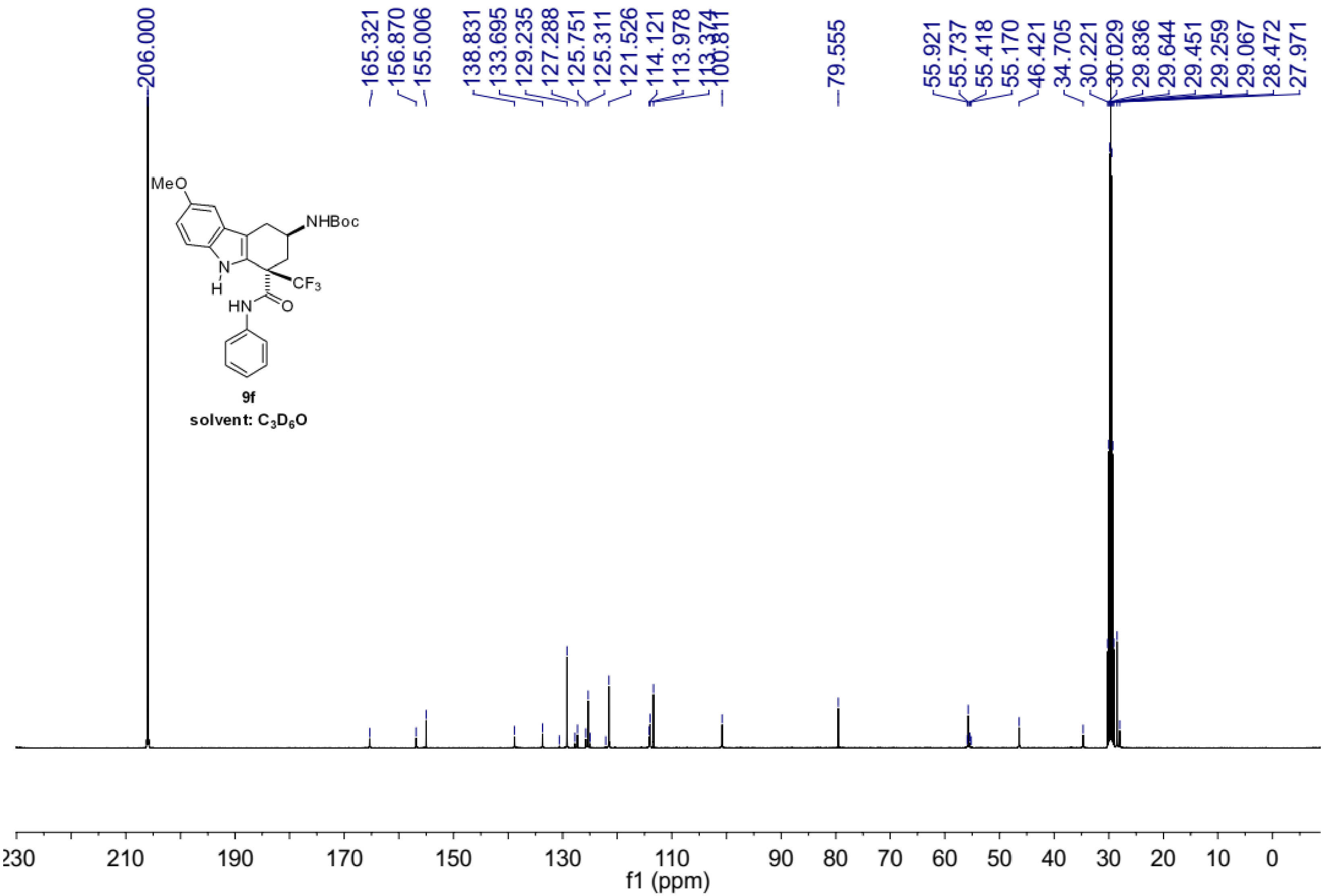

Supplementary Figure 182. ^{13}C NMR spectrum for compound **9f**

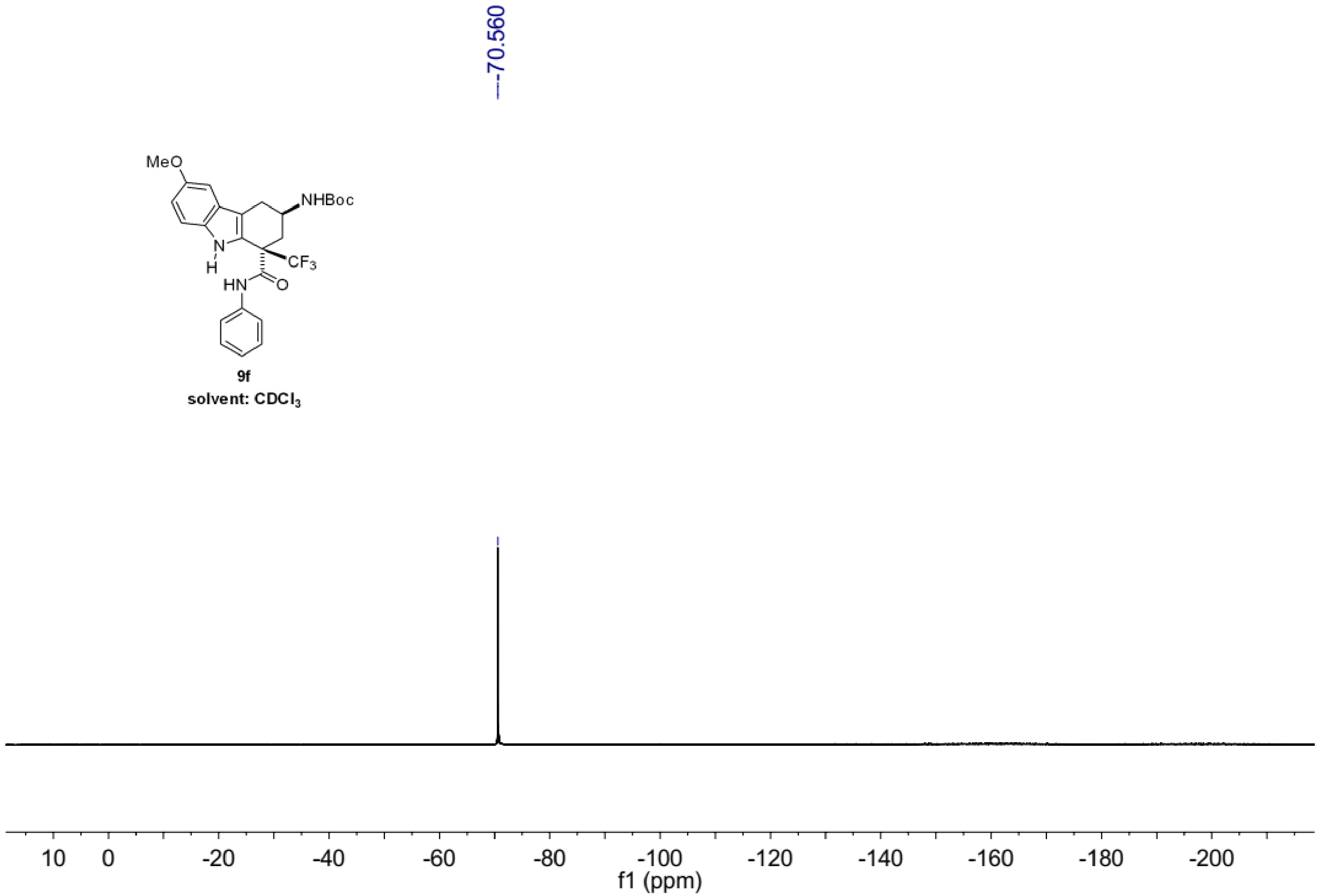

Supplementary Figure 183. ^{19}F NMR spectrum for compound **3f**

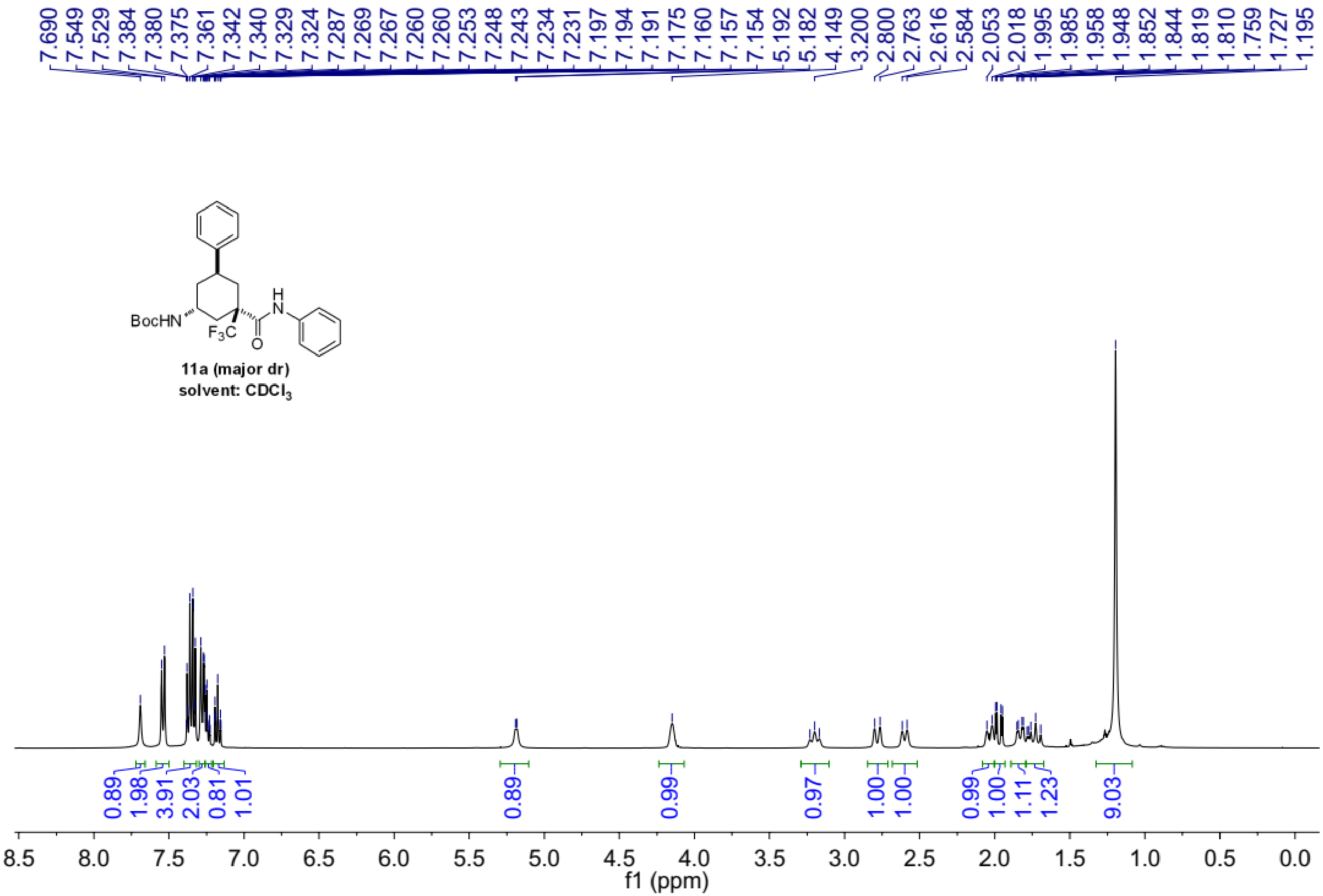

Supplementary Figure 184. ^{1}H NMR spectrum for compound **11a** (major dr)

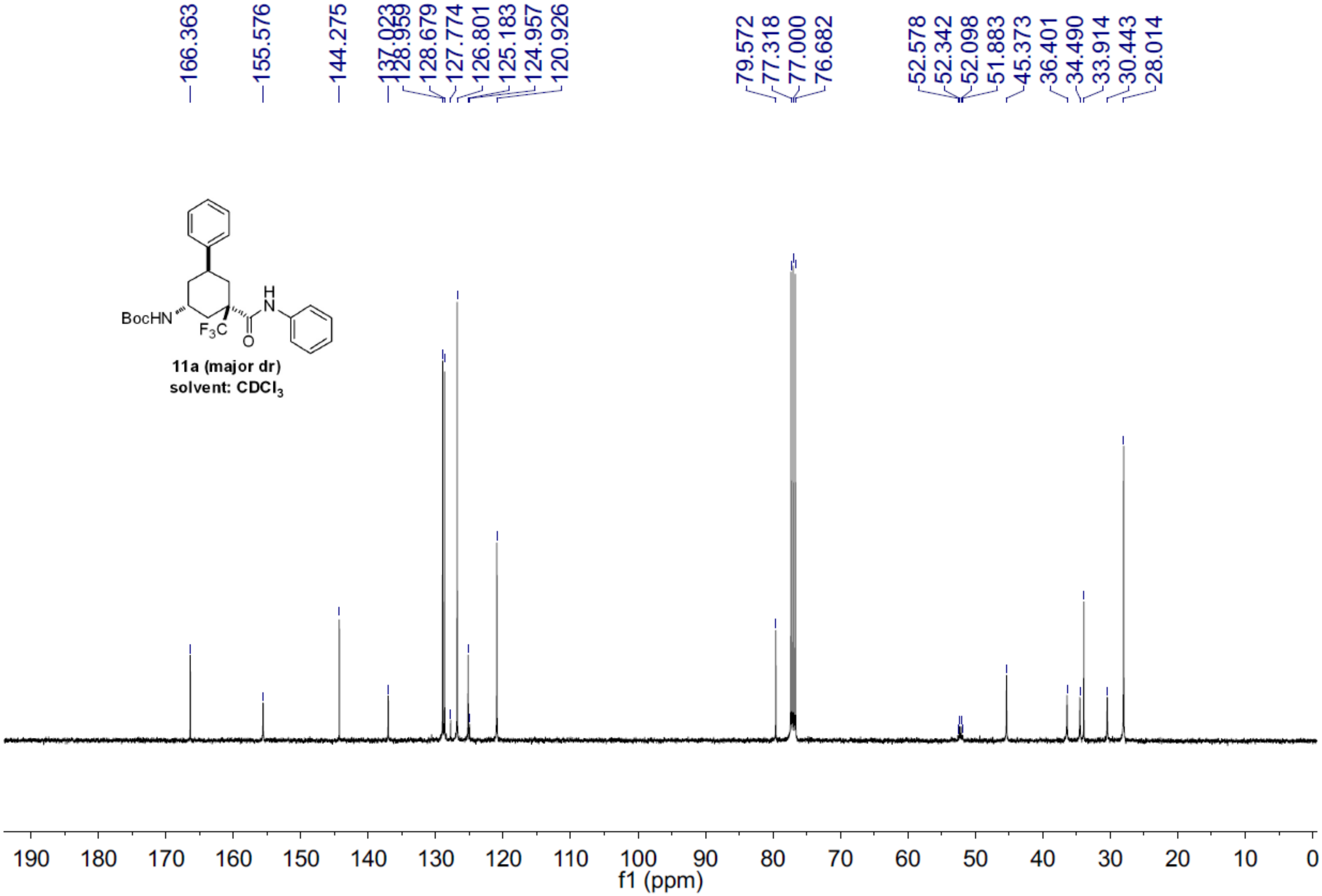

Supplementary Figure 185. ^{13}C NMR spectrum for compound **11a** (major dr)

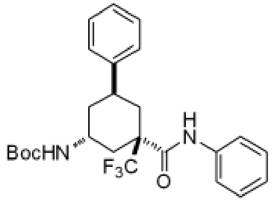

11a (major dr)
solvent: $CDCl_3$

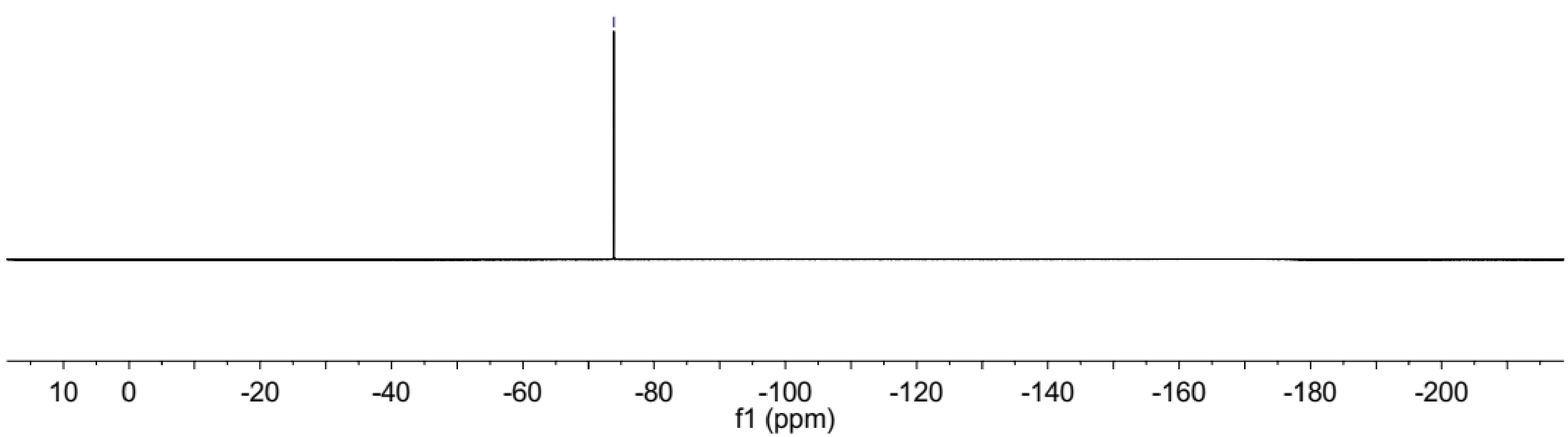

Supplementary Figure 186. ^{19}F NMR spectrum for compound **11a** (major dr)

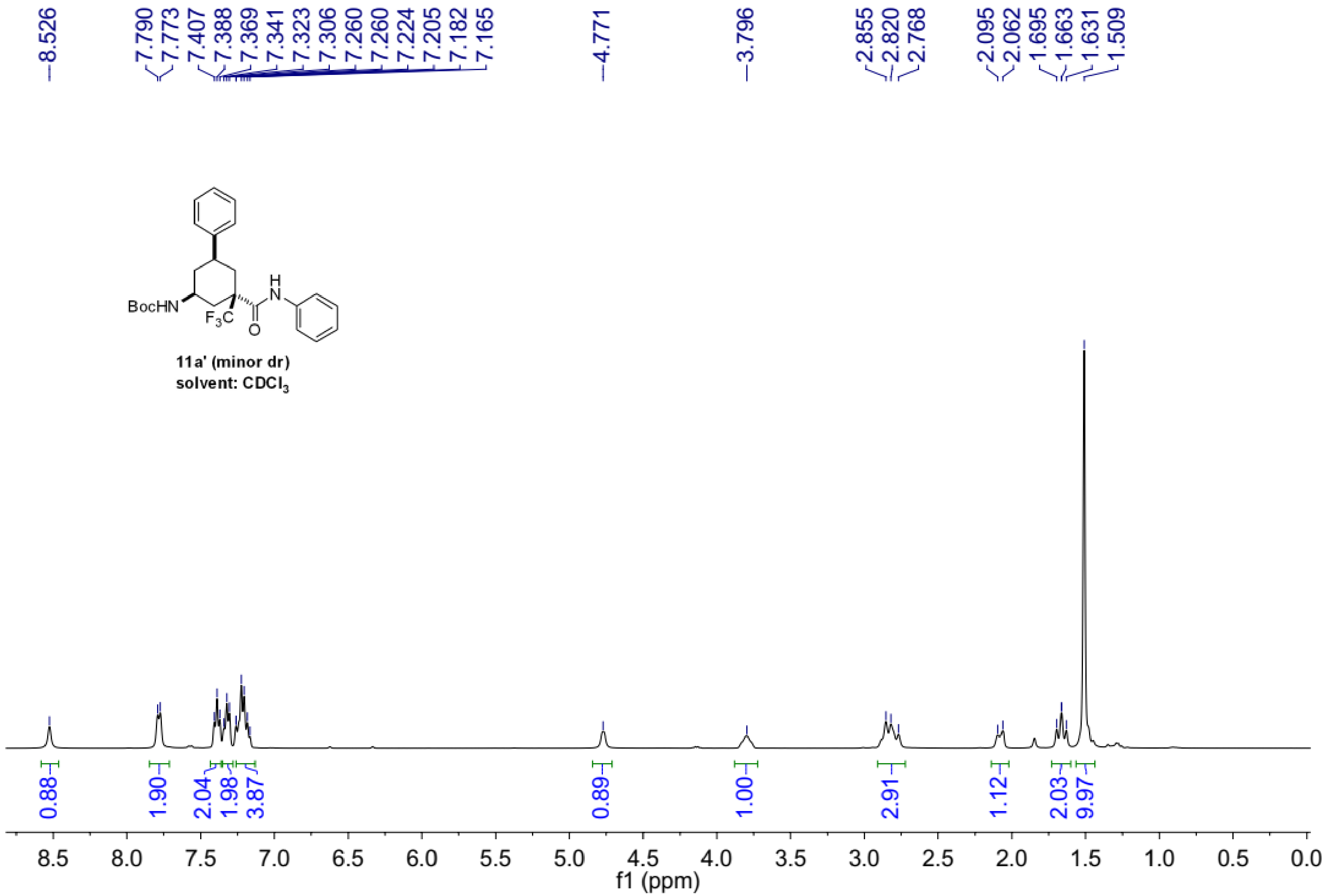

Supplementary Figure 187. ^{1}H NMR spectrum for compound **11a'** (minor dr)

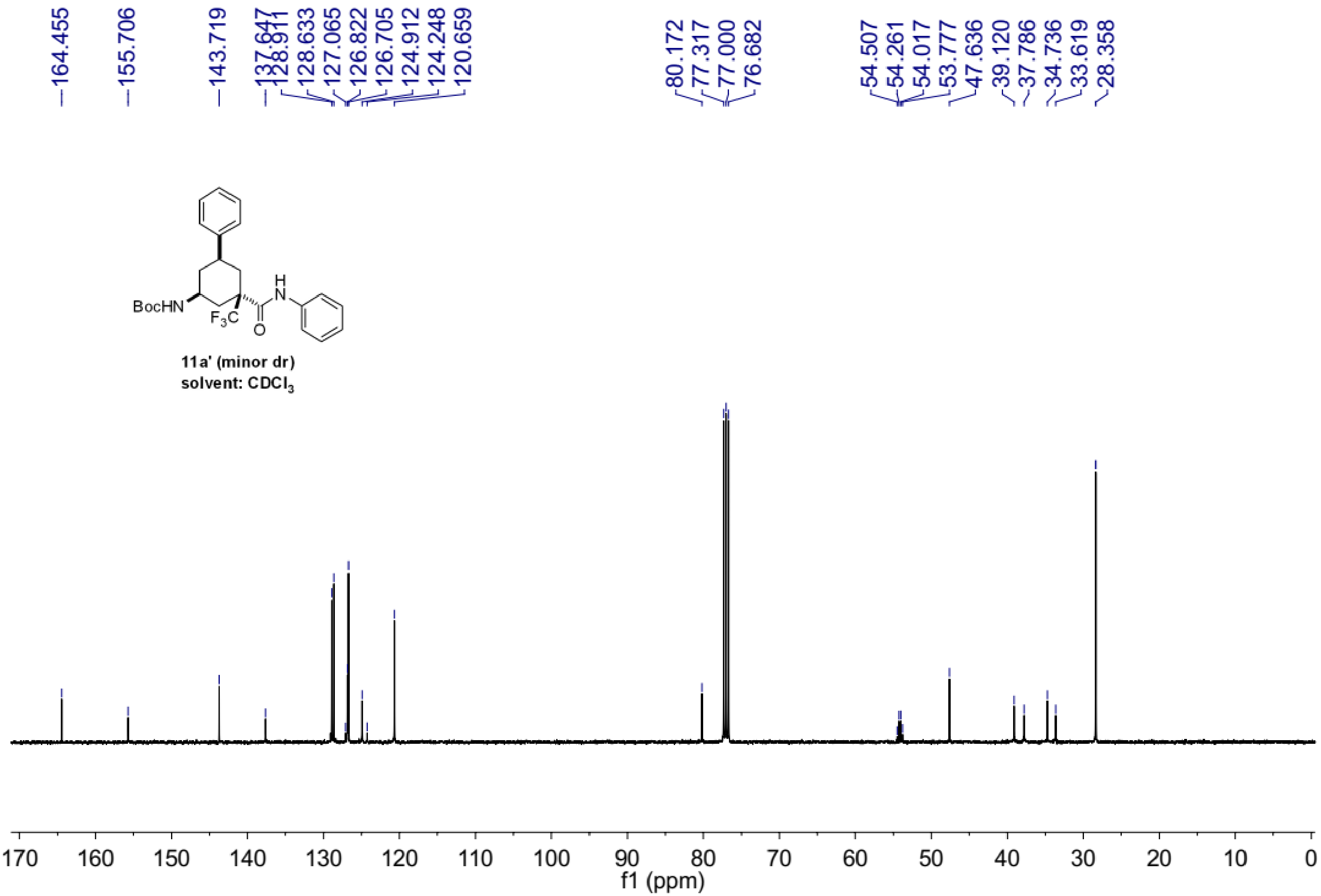

Supplementary Figure 188. ^{13}C NMR spectrum for compound **11a'** (minor dr)

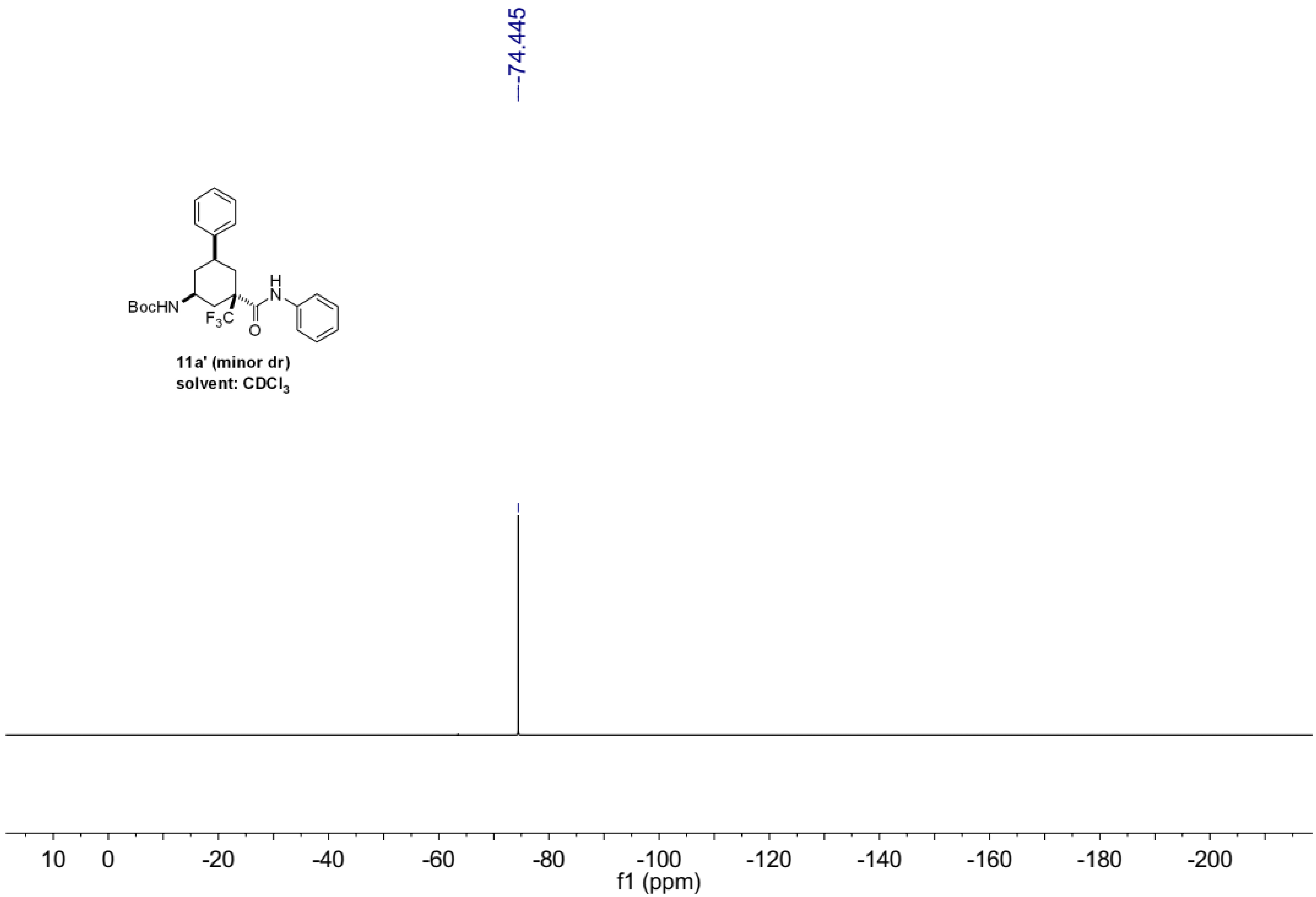

Supplementary Figure 189. ^{19}F NMR spectrum for compound **11a’** (minor dr)

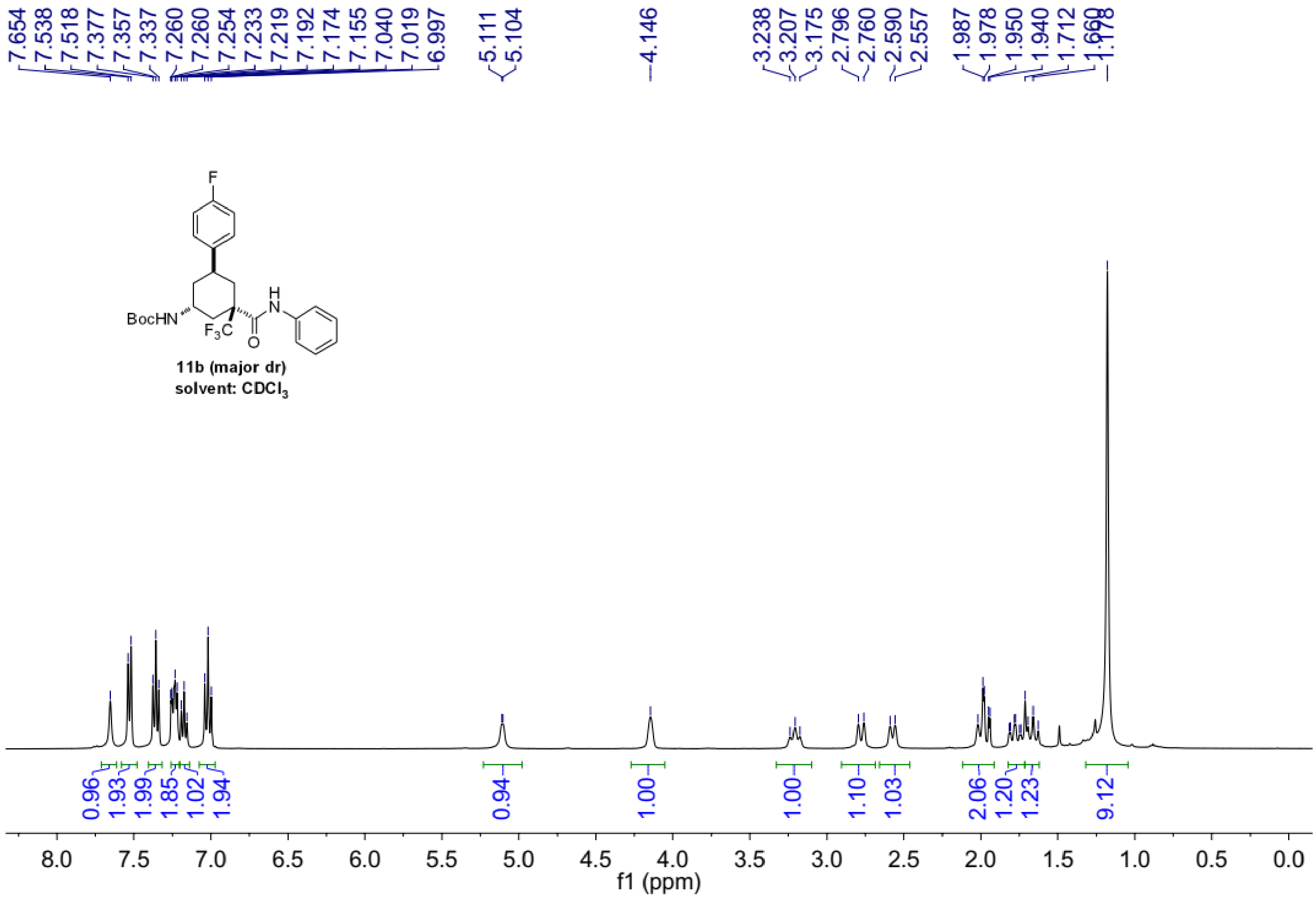

Supplementary Figure 190. ^{1}H NMR spectrum for compound **11b** (major dr)

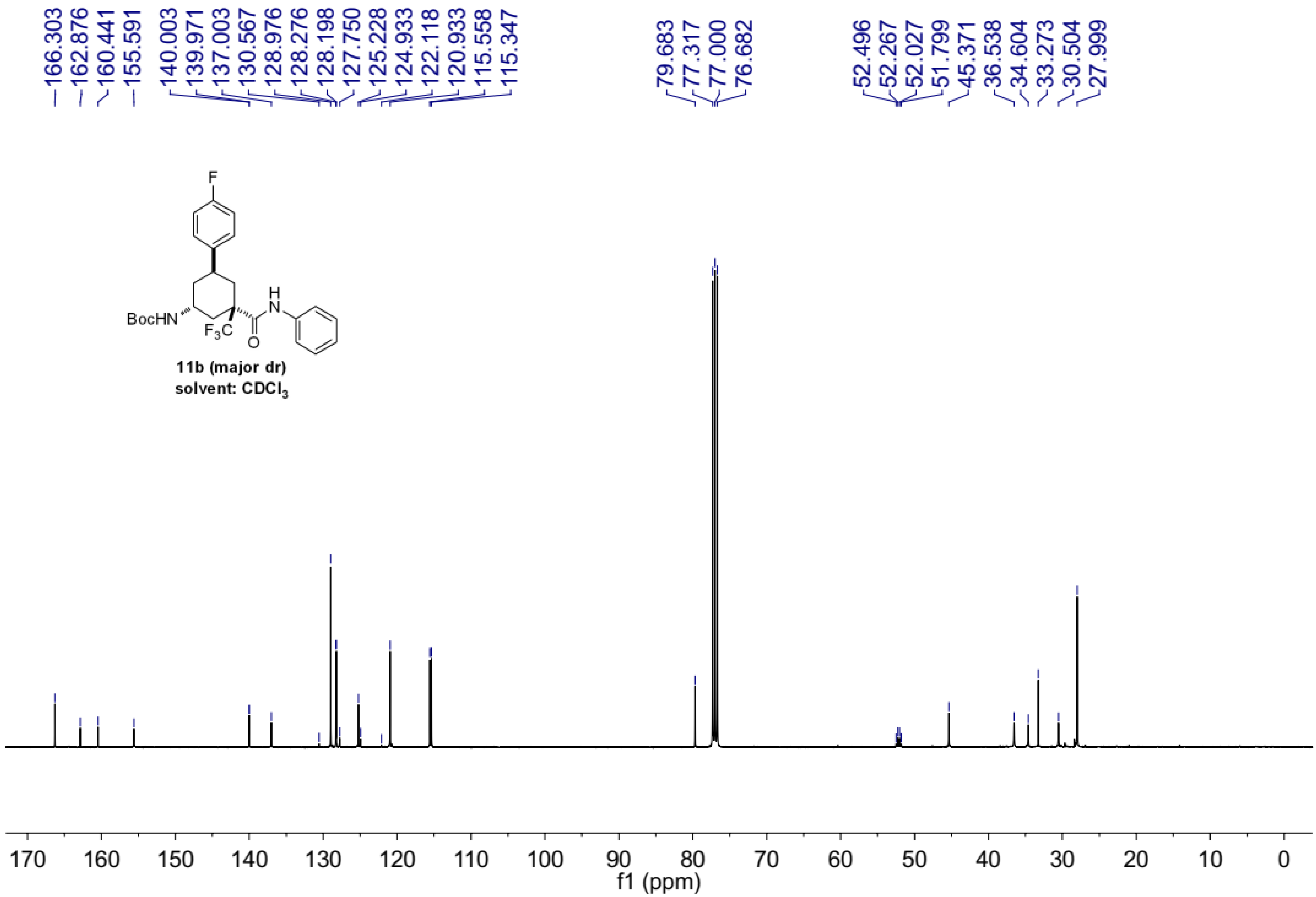

Supplementary Figure 191. ^{13}C NMR spectrum for compound **11b** (major dr)

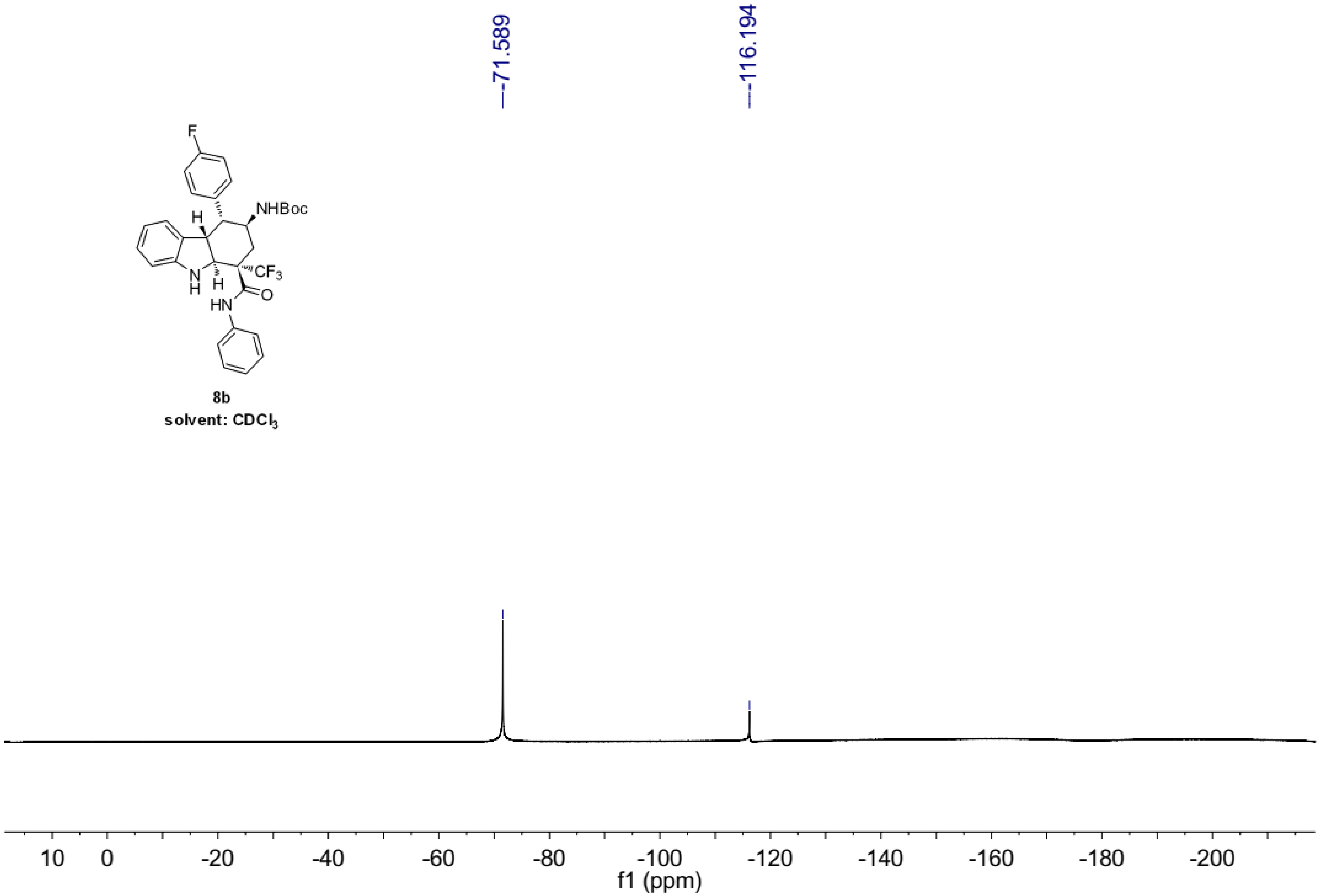

Supplementary Figure 192. ^{19}F NMR spectrum for compound **11b** (major dr)

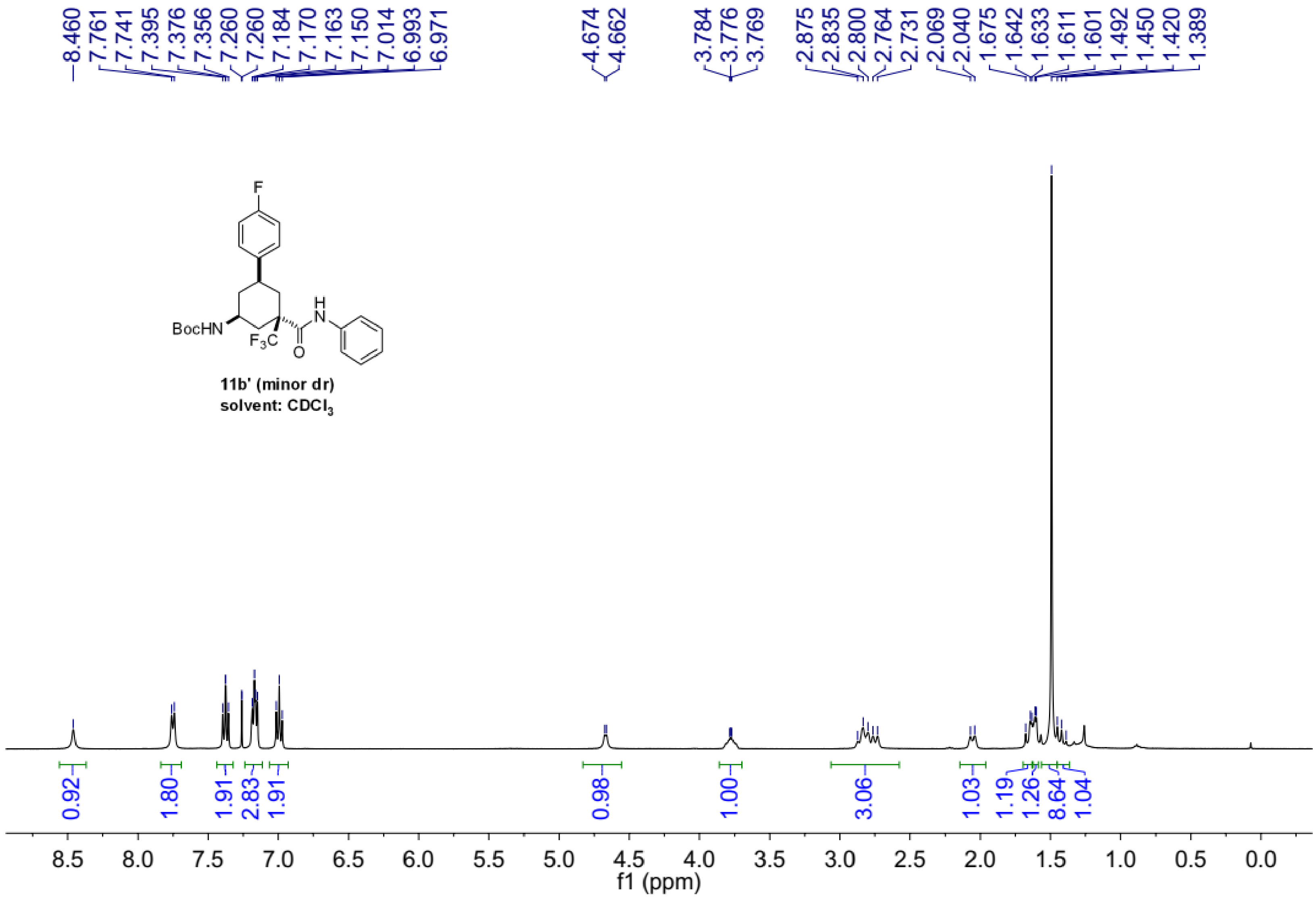

Supplementary Figure 193. ^{1}H NMR spectrum for compound **11b'** (minor dr)

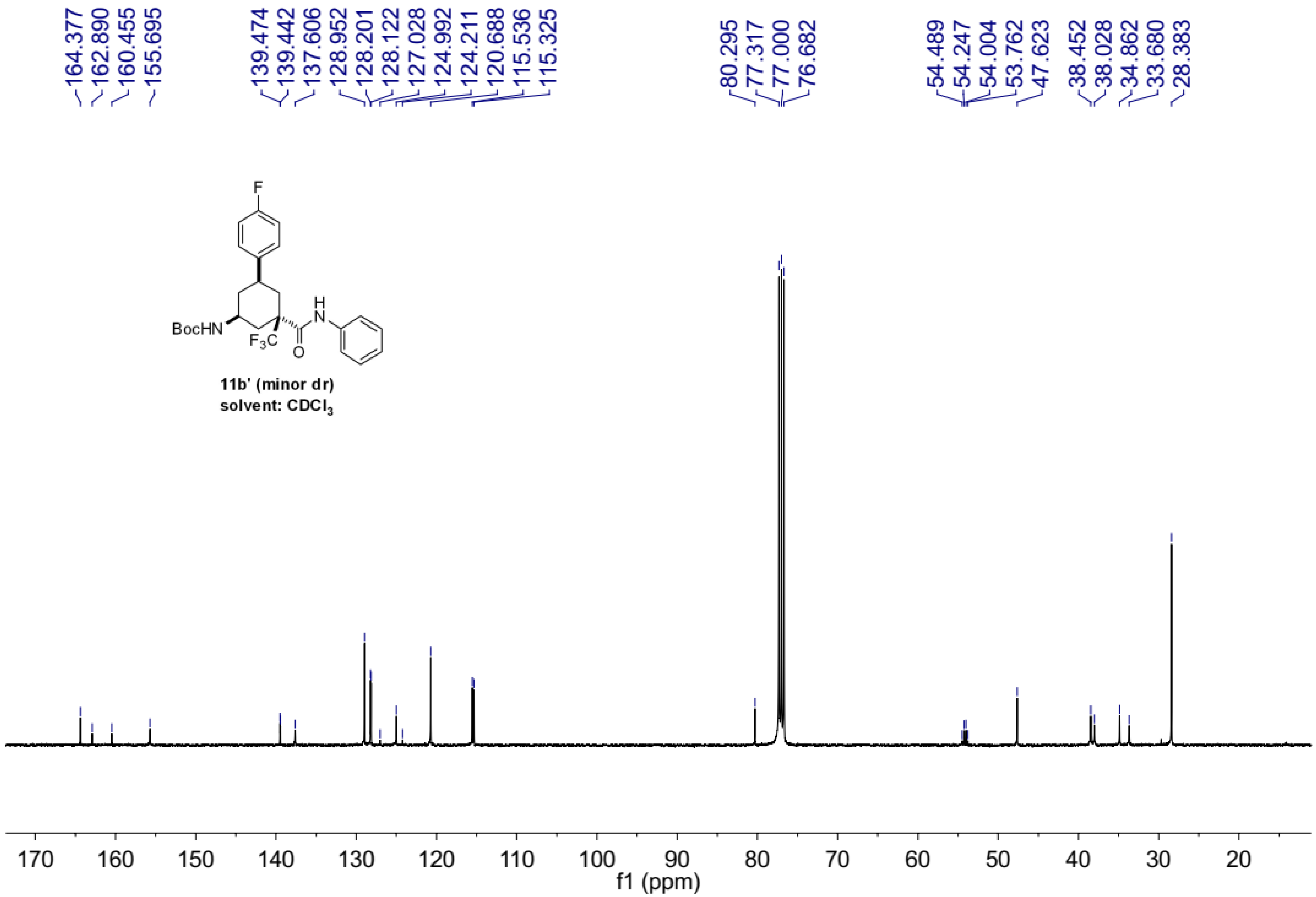

Supplementary Figure 194. ^{13}C NMR spectrum for compound **11b'** (minor dr)

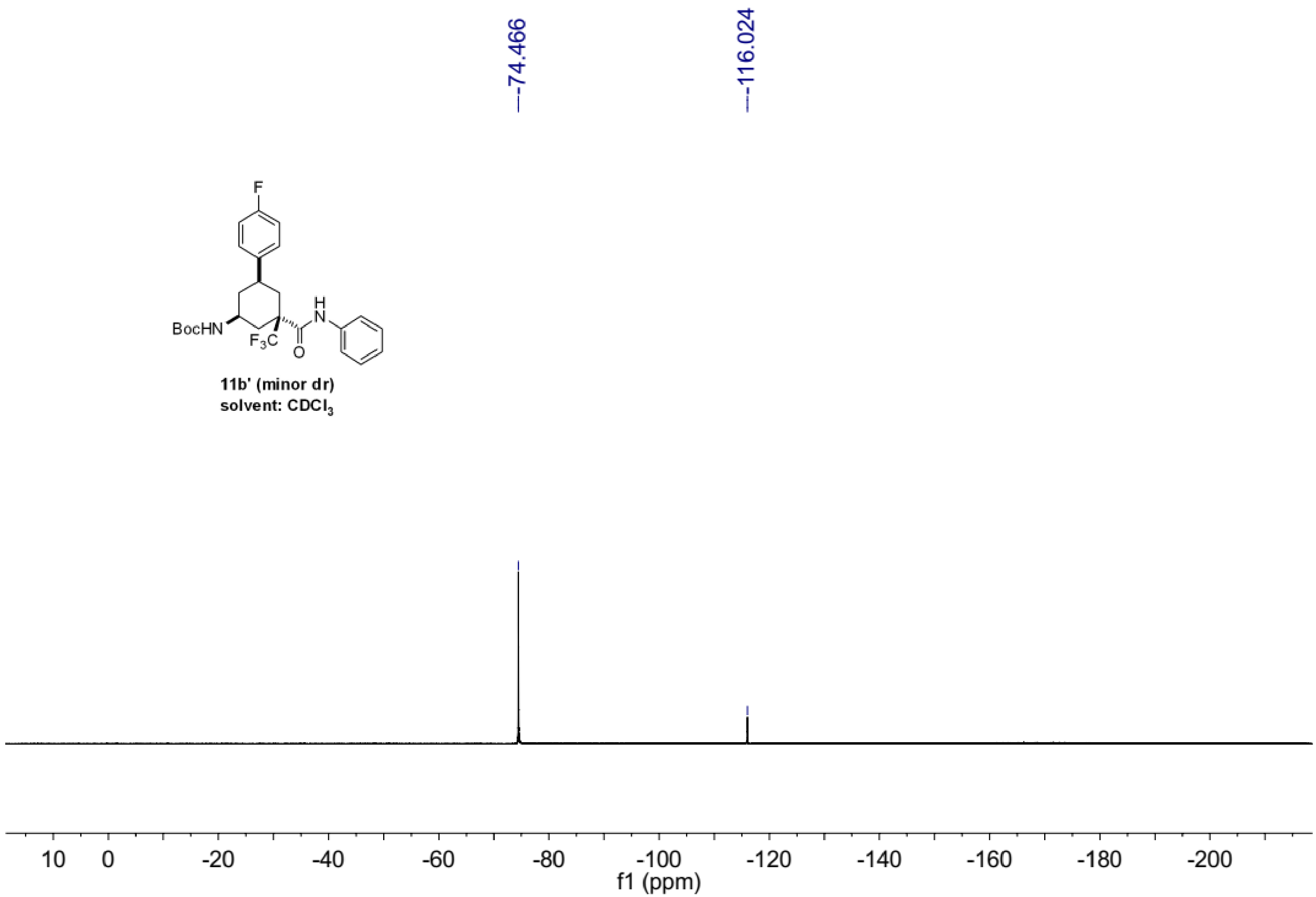

Supplementary Figure 195. ^{19}F NMR spectrum for compound **11b'** (minor dr)

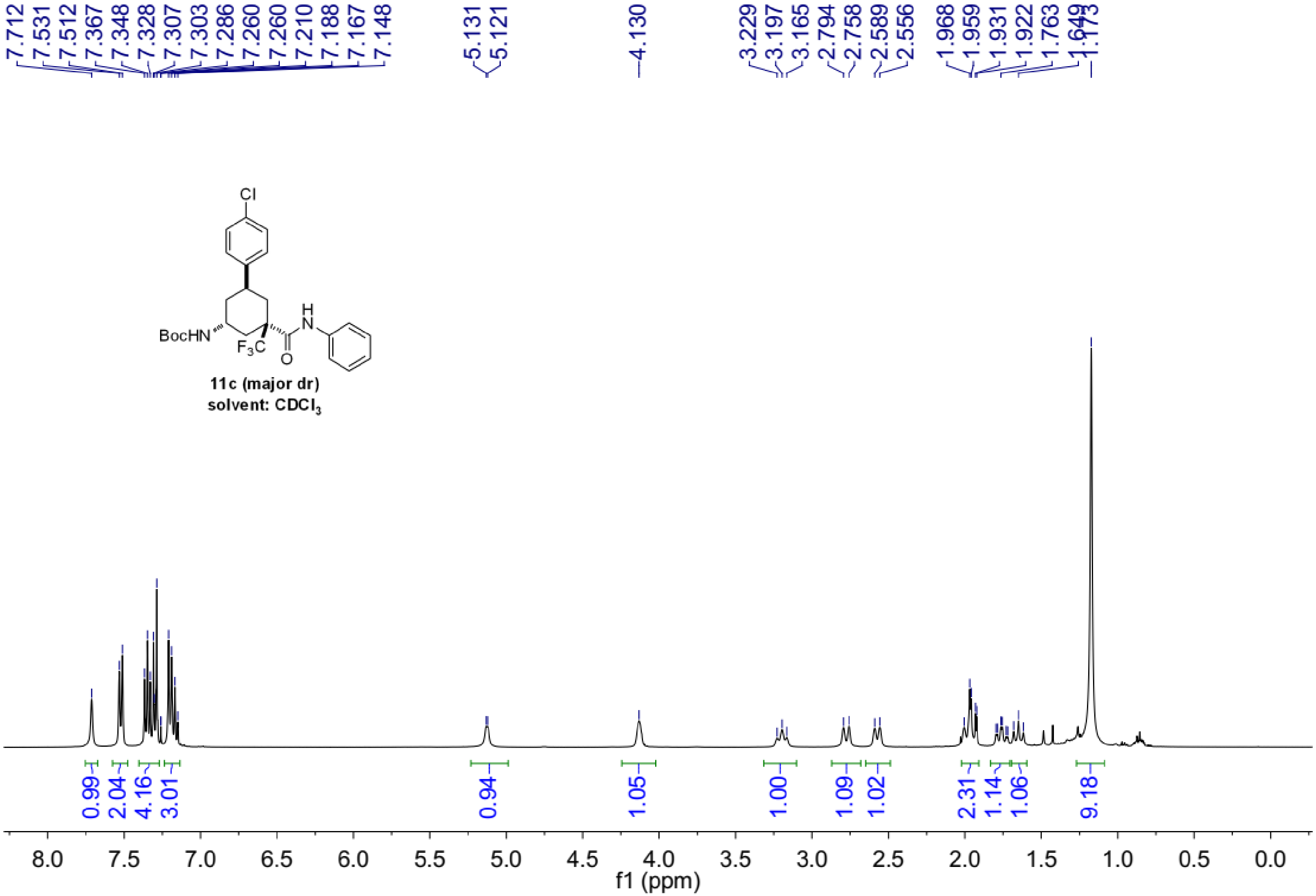

Supplementary Figure 196. ^{1}H NMR spectrum for compound **11c** (major dr)

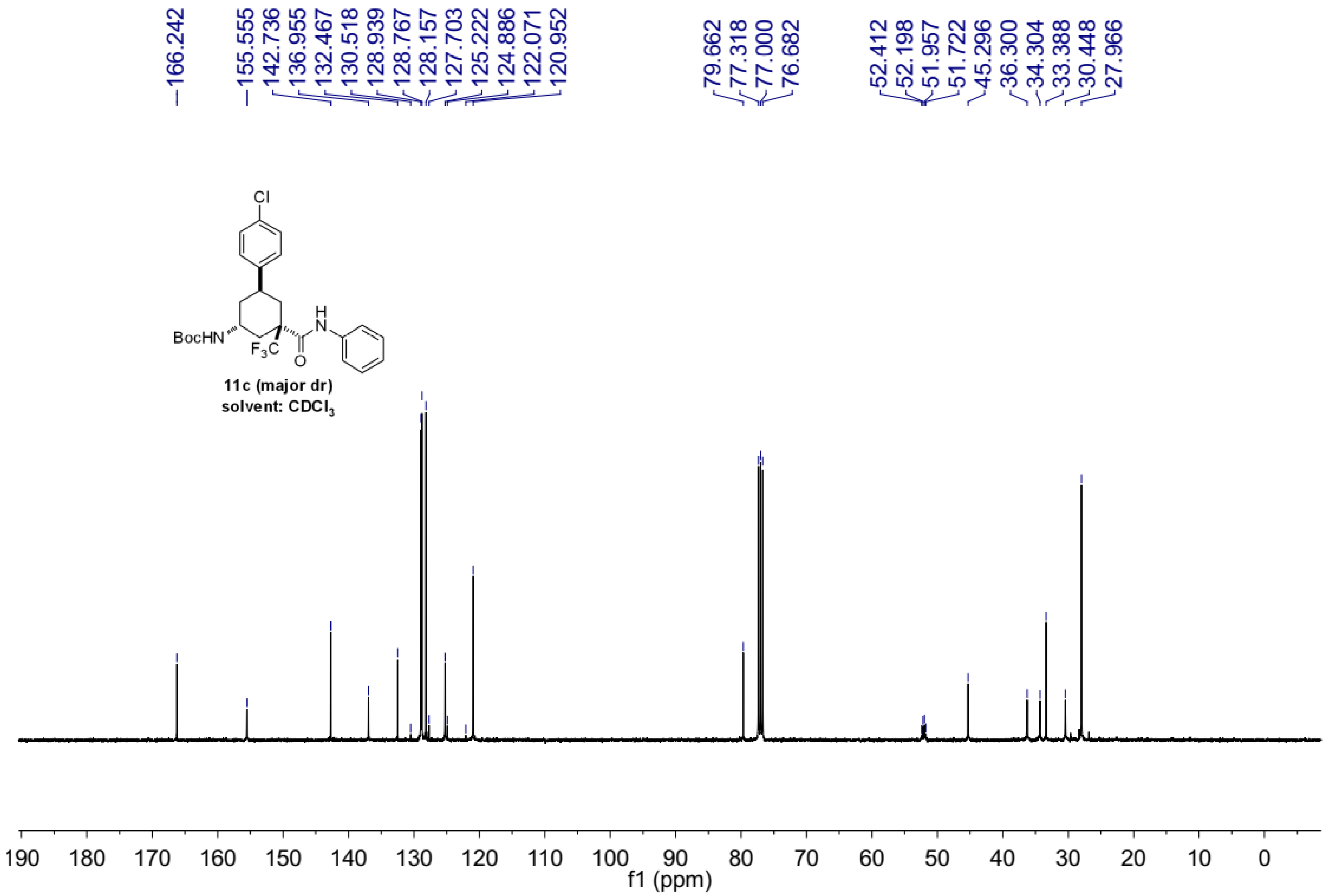

Supplementary Figure 197. ^{13}C NMR spectrum for compound **11c** (major dr)

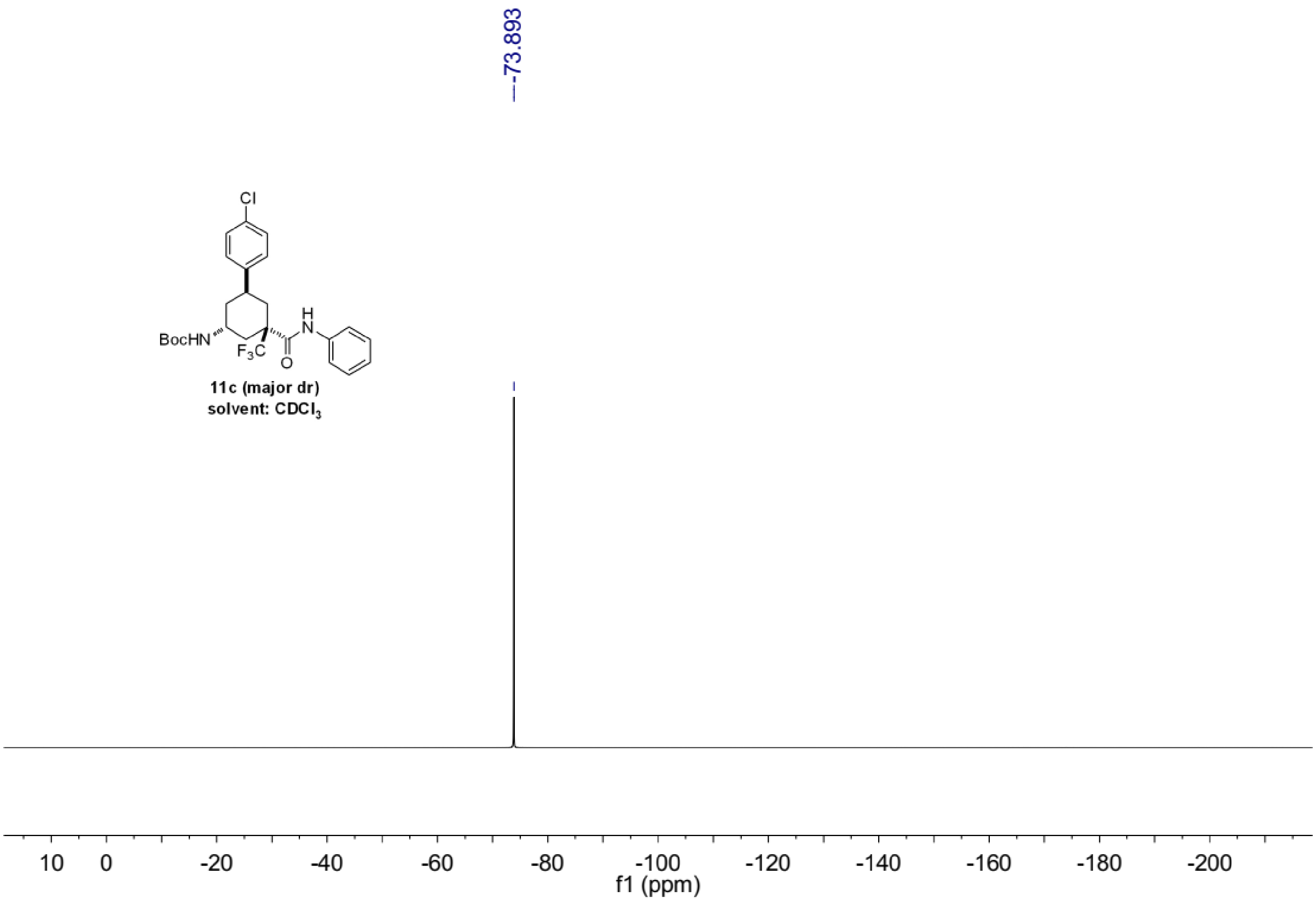

Supplementary Figure 198. ^{19}F NMR spectrum for compound **11c** (major dr)

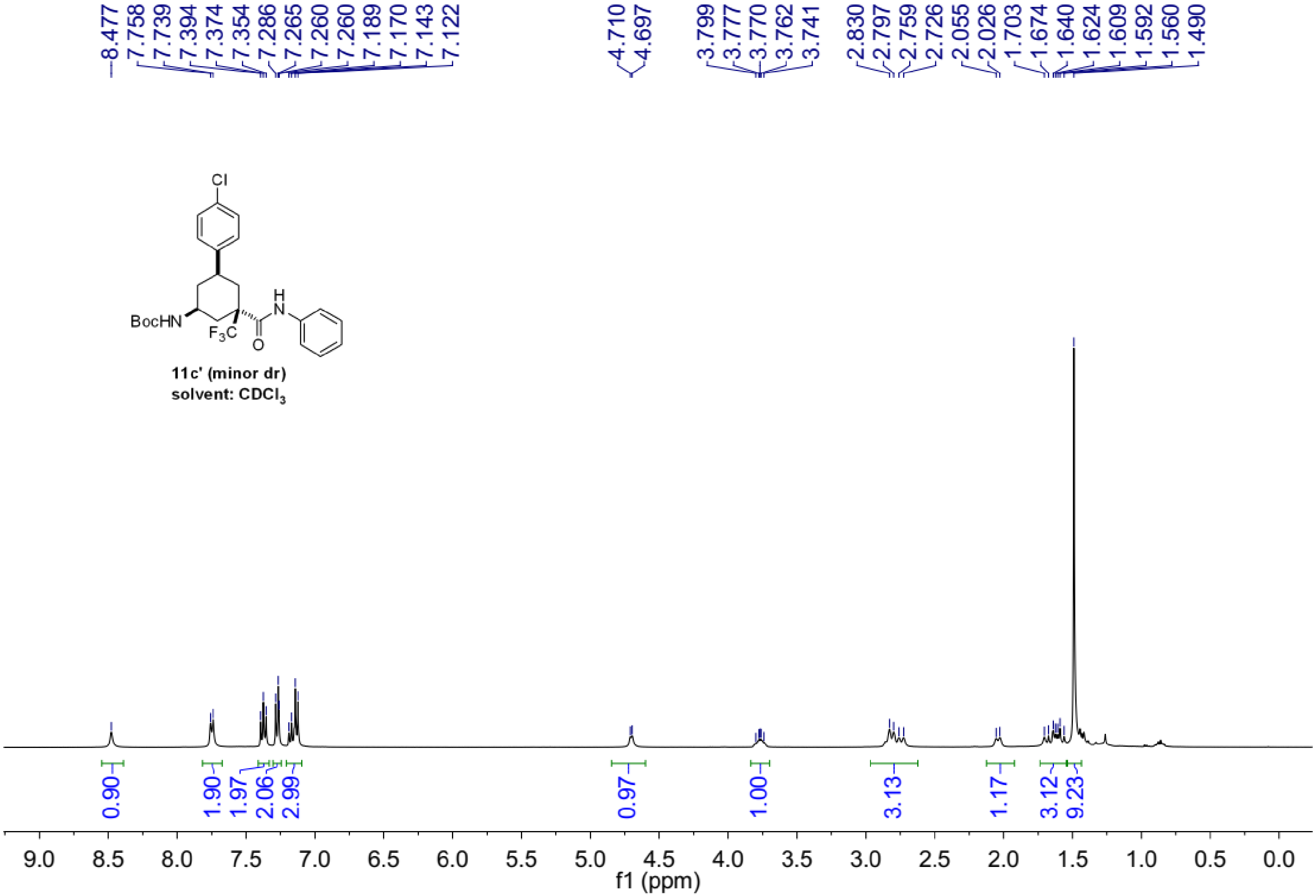

Supplementary Figure 199. ^{1}H NMR spectrum for compound **11c'** (minor dr)

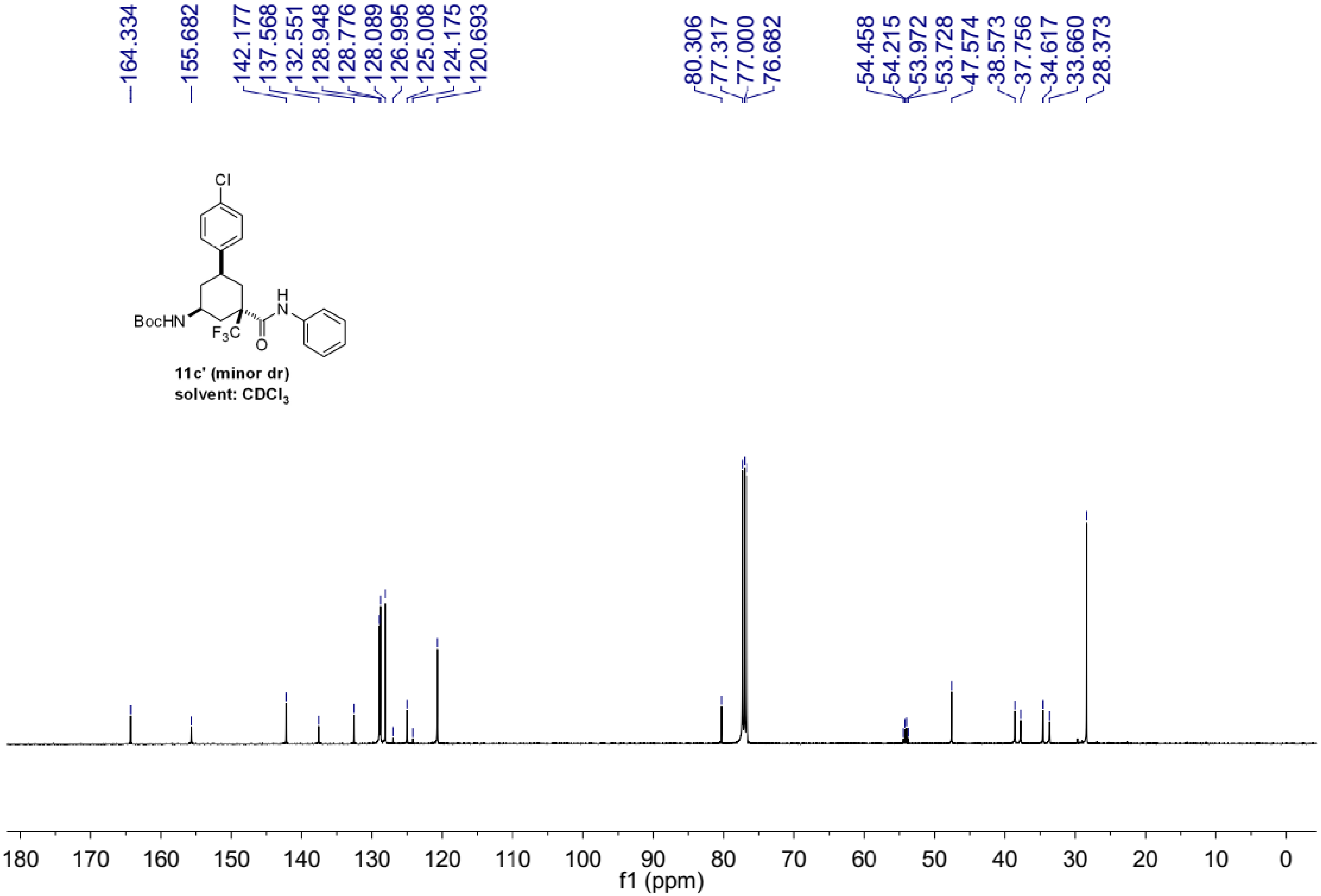

Supplementary Figure 200. ^{13}C NMR spectrum for compound **11c’** (minor dr)

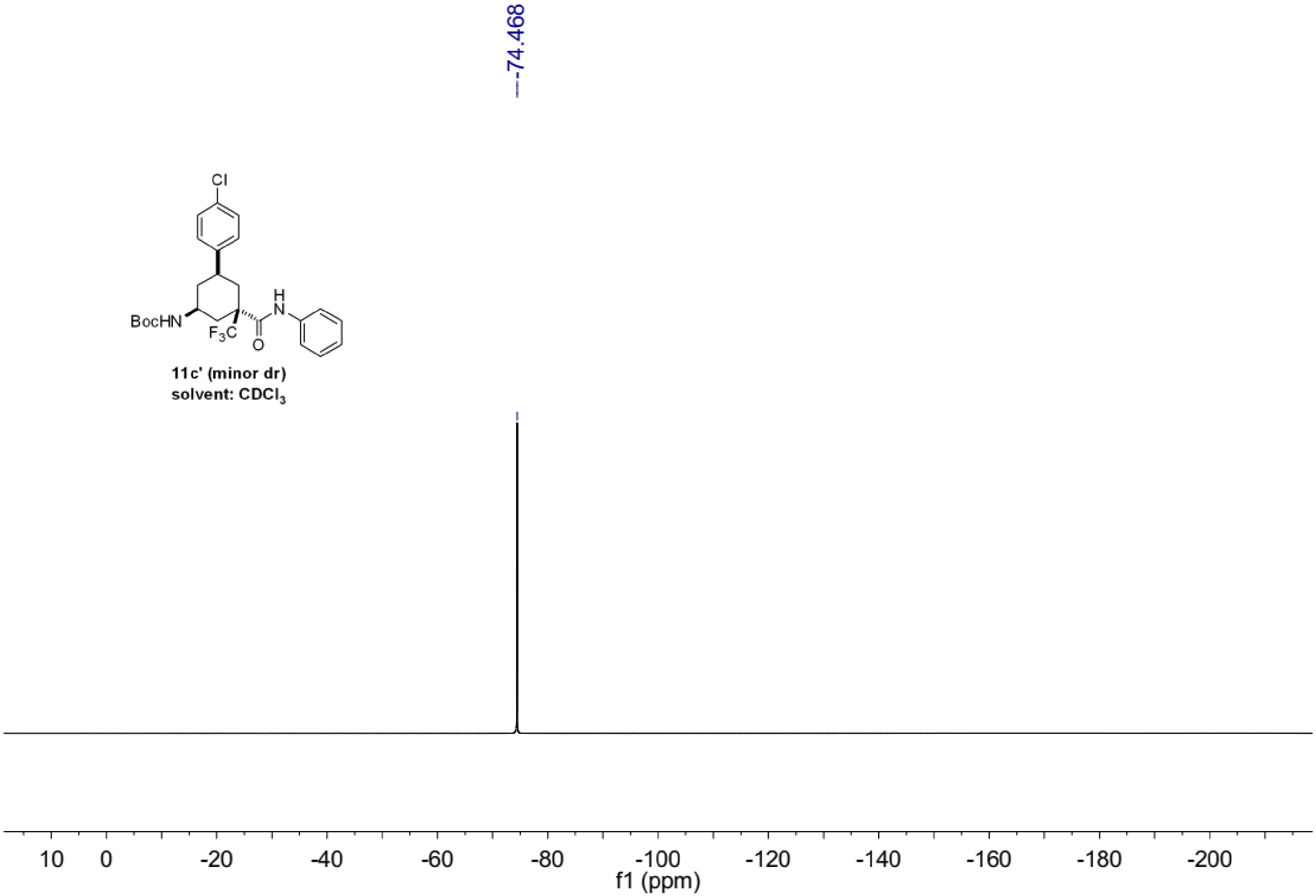

Supplementary Figure 201. ^{19}F NMR spectrum for compound **11c’** (minor dr)

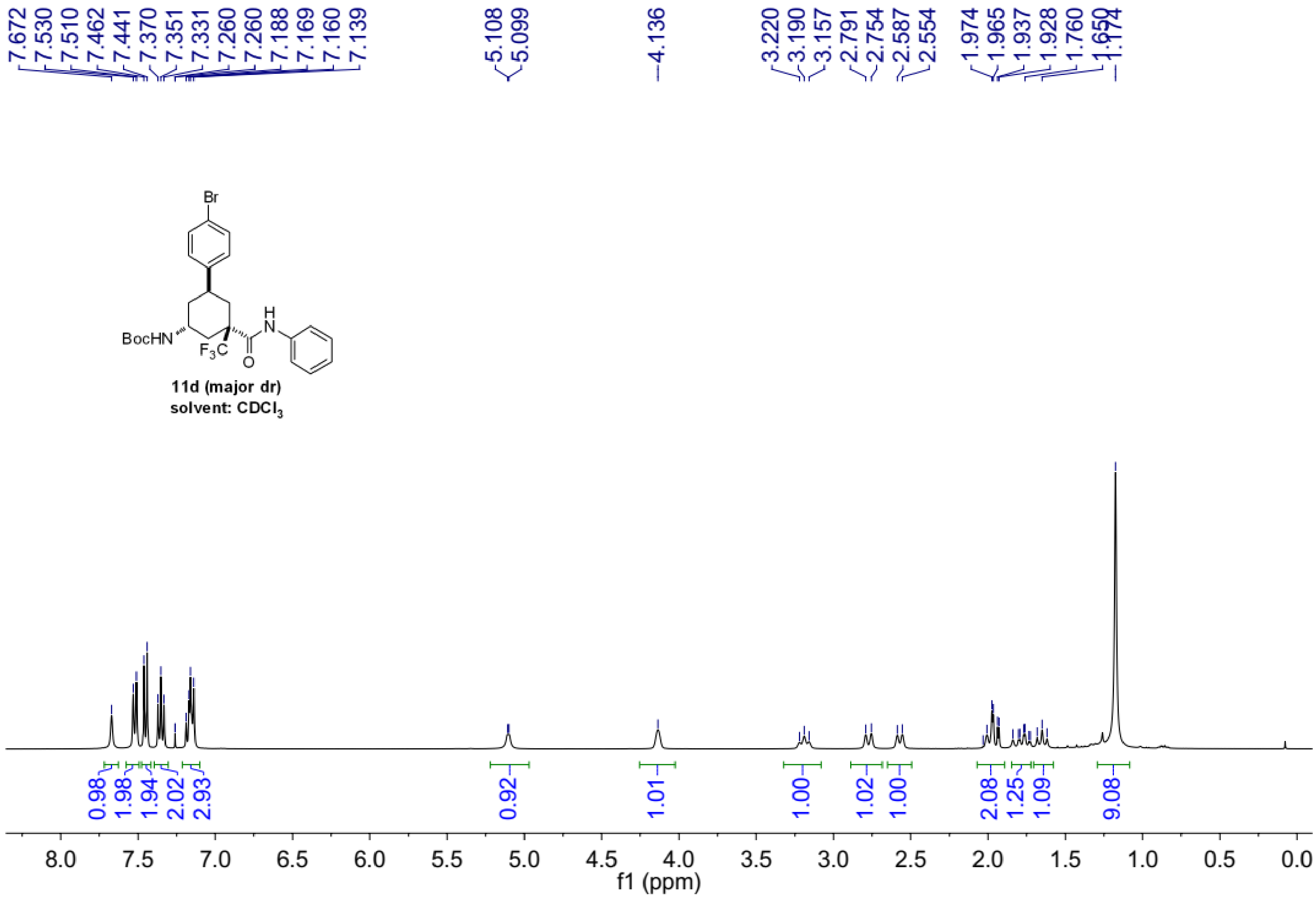

Supplementary Figure 202. ^{1}H NMR spectrum for compound **11d** (major dr)

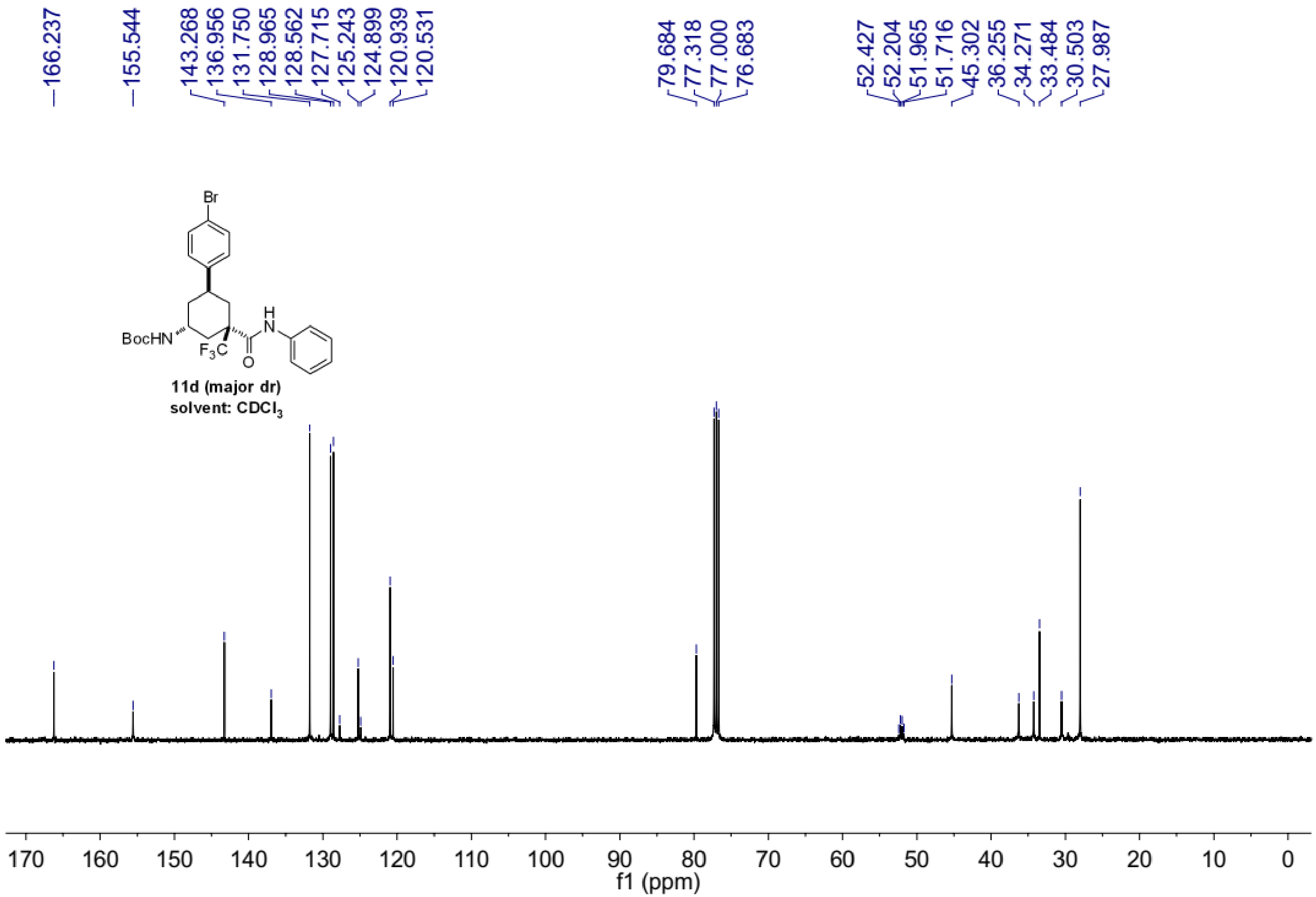

Supplementary Figure 203. ^{13}C NMR spectrum for compound **11d** (major dr)

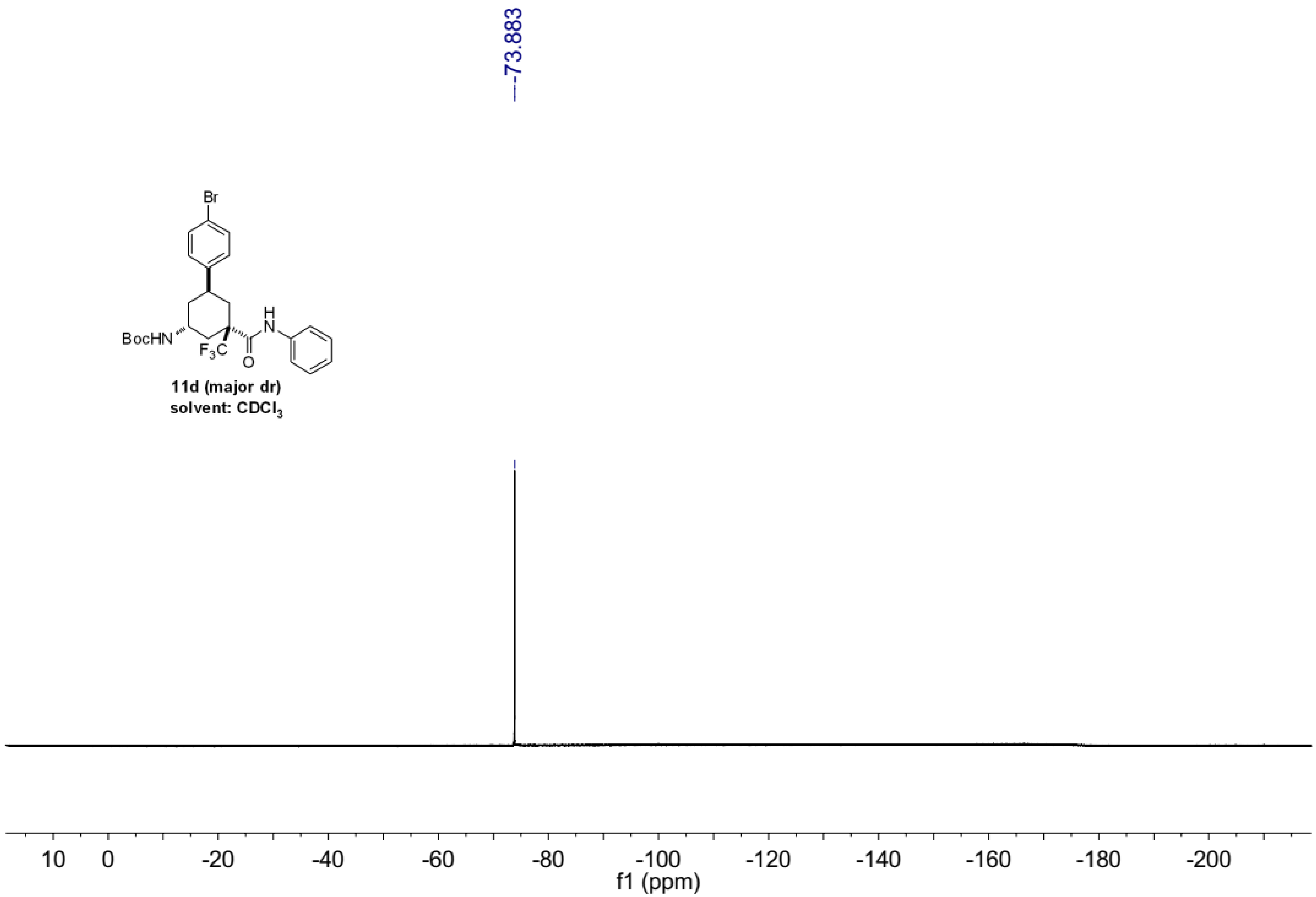

Supplementary Figure 204. ^{19}F NMR spectrum for compound **11d** (major dr)

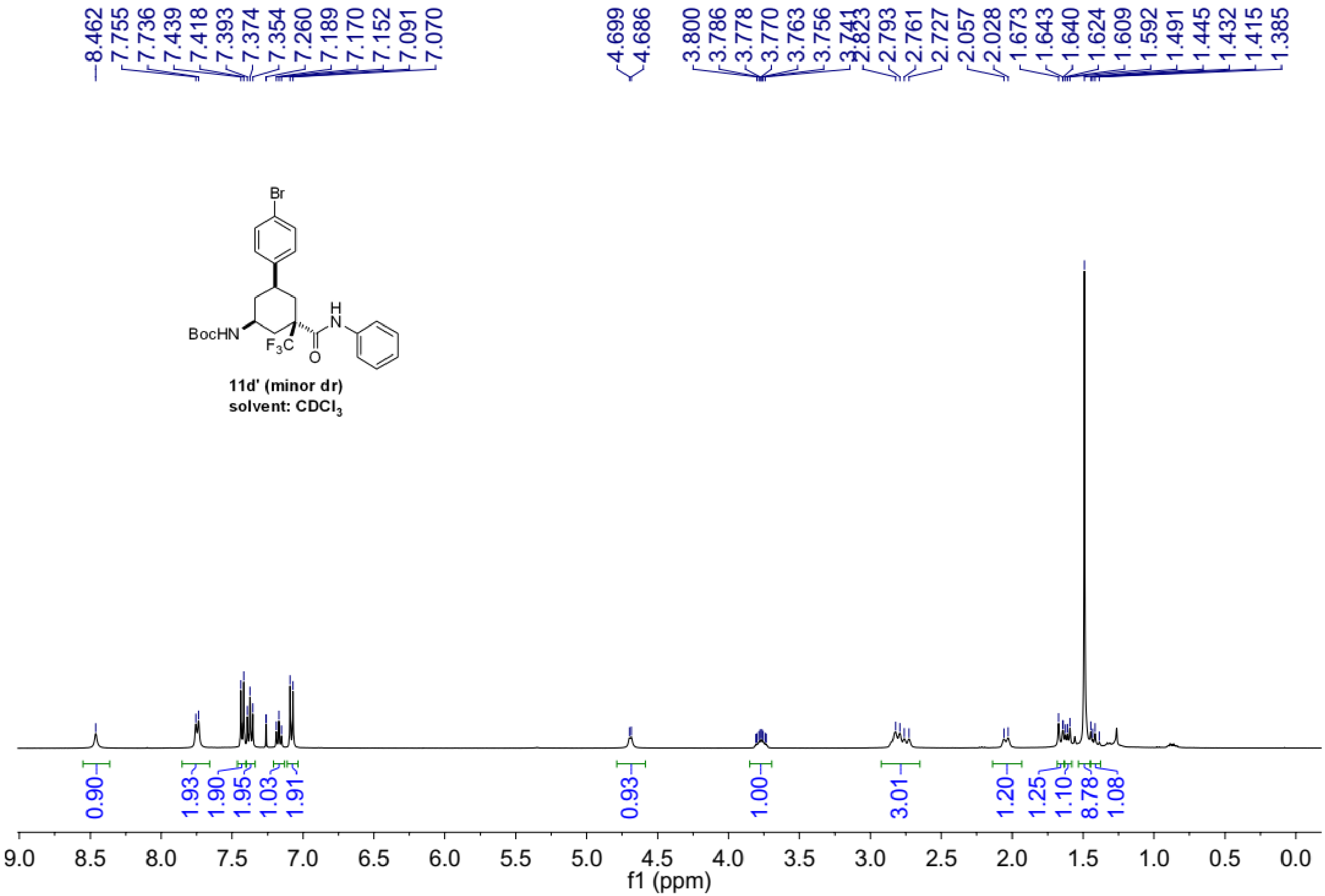

Supplementary Figure 205. ^{1}H NMR spectrum for compound **11d’** (minor dr)

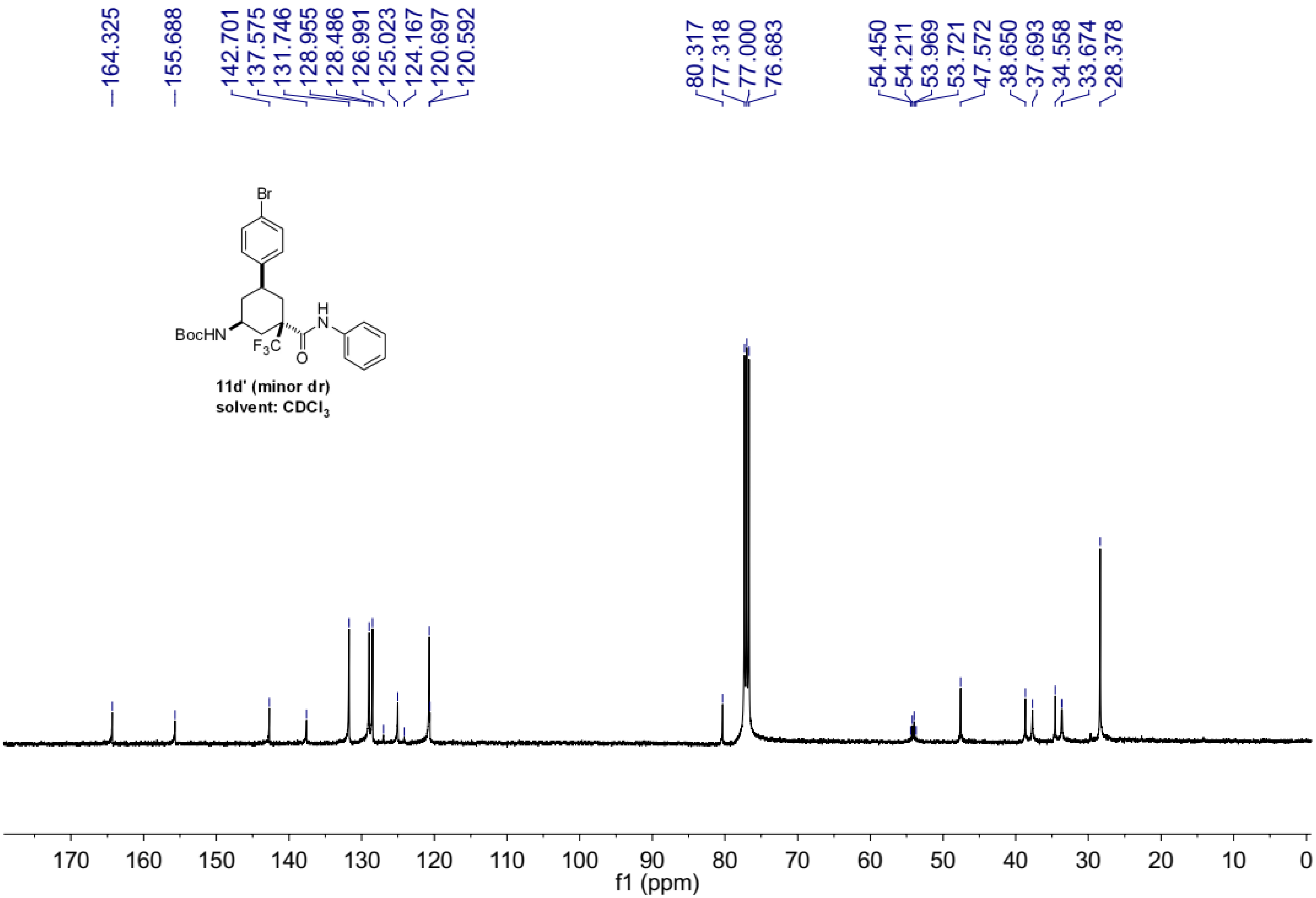

Supplementary Figure 206. ^{13}C NMR spectrum for compound **11d’** (minor dr)

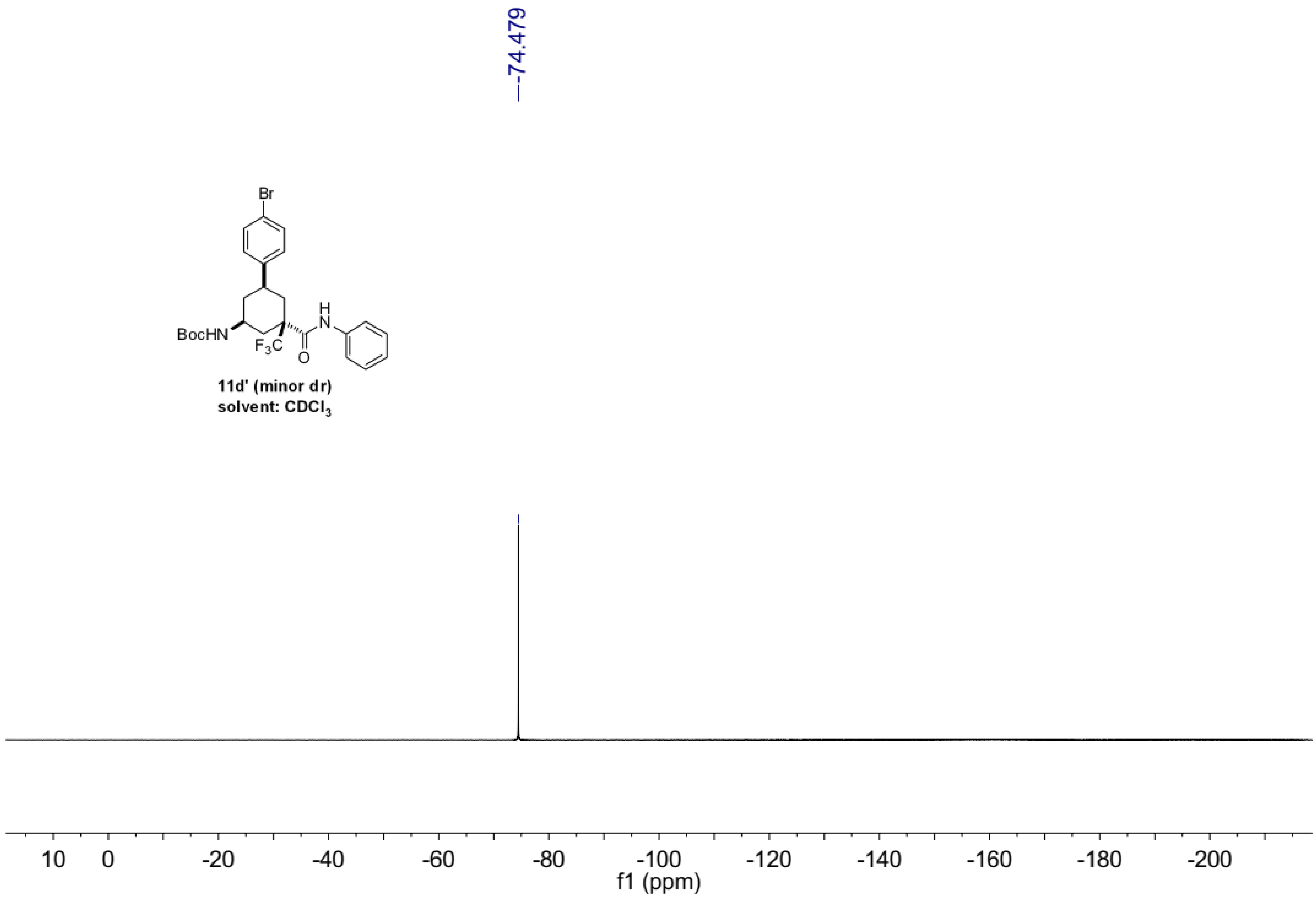

Supplementary Figure 207. ^{19}F NMR spectrum for compound **11d'** (minor dr)

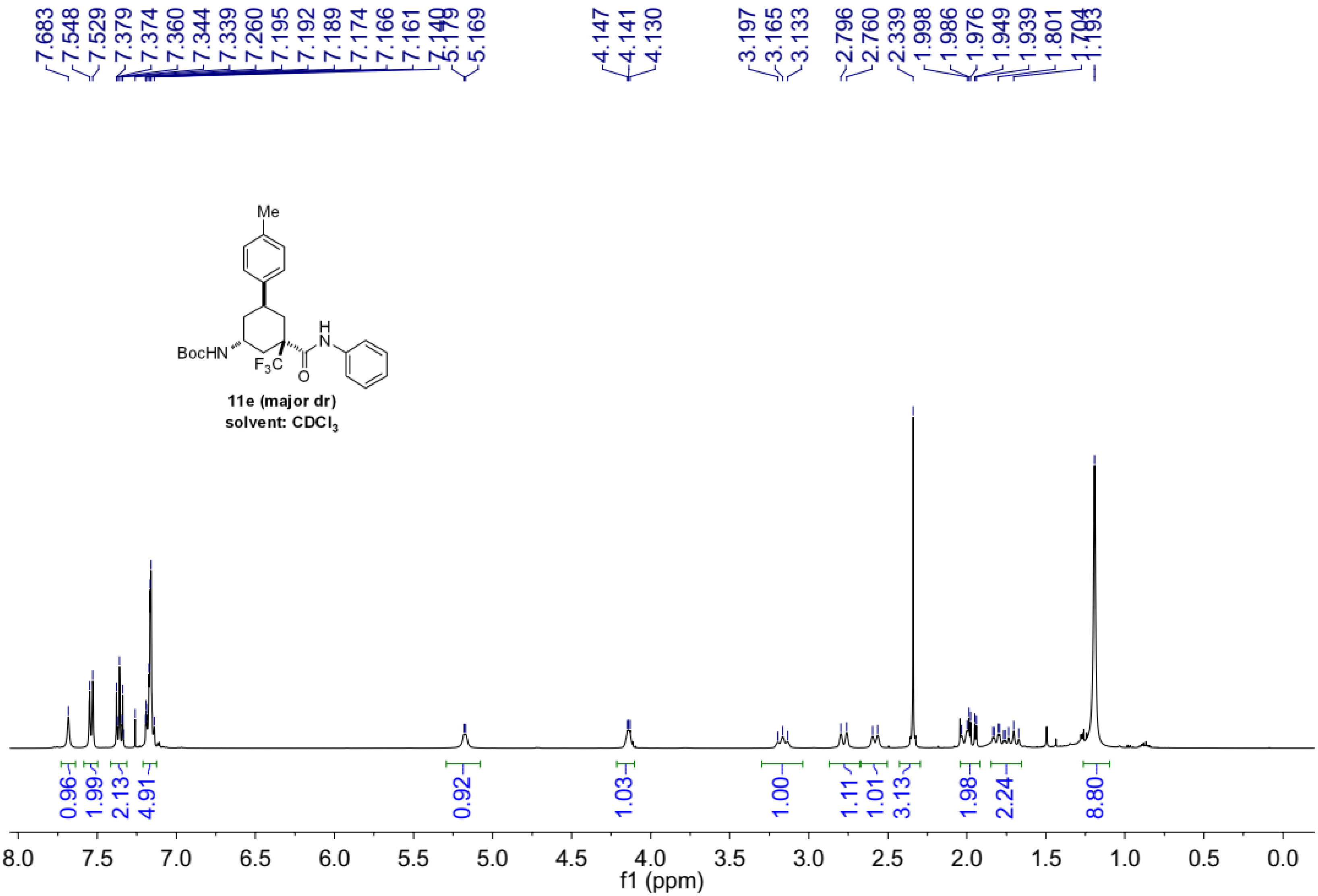

Supplementary Figure 208. ^{1}H NMR spectrum for compound **11e** (major dr)

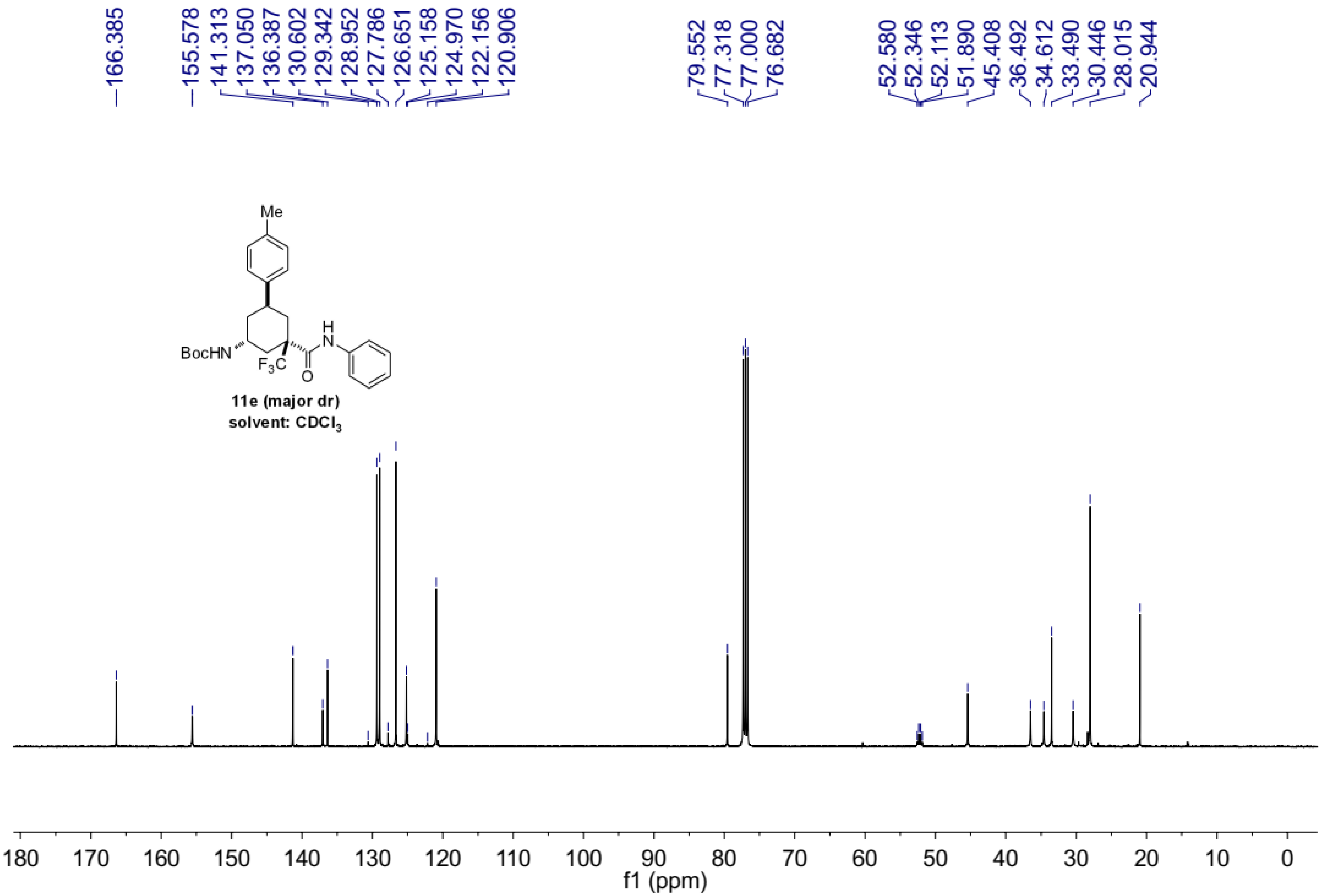

Supplementary Figure 209. ^{13}C NMR spectrum for compound **11e** (major dr)

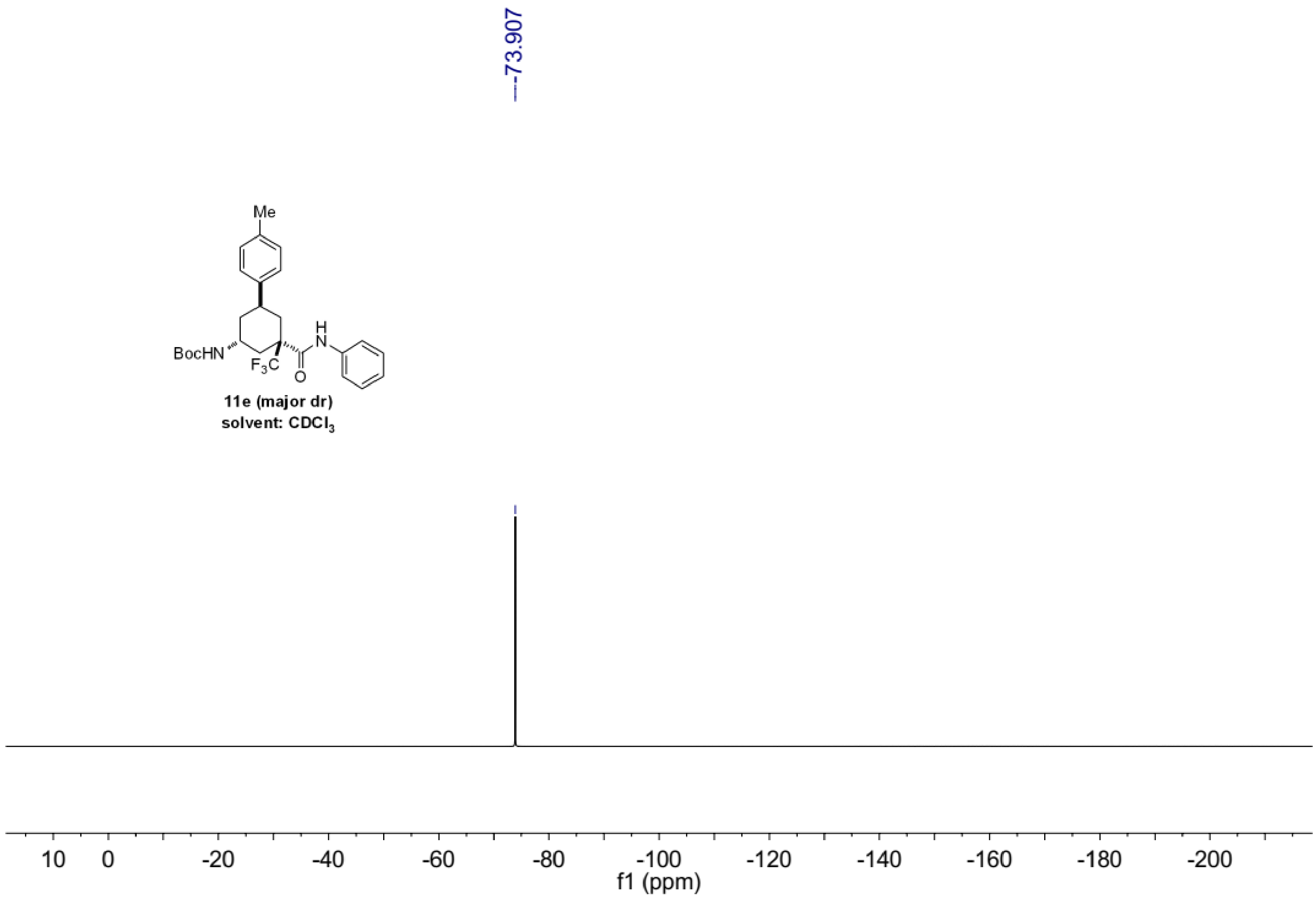

Supplementary Figure 210. ^{19}F NMR spectrum for compound **11e** (major dr)

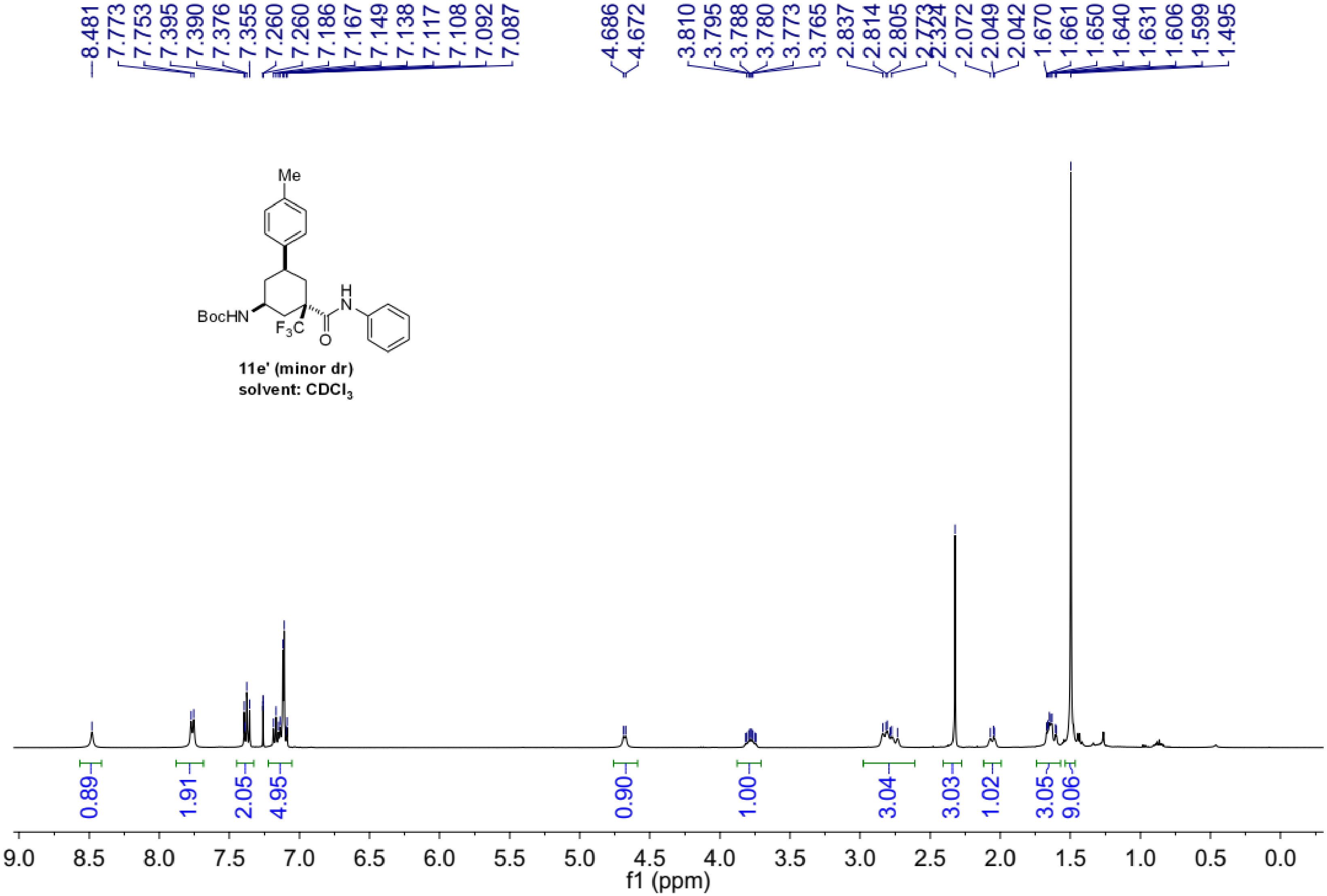

Supplementary Figure 211. ^{1}H NMR spectrum for compound **11e'** (minor dr)

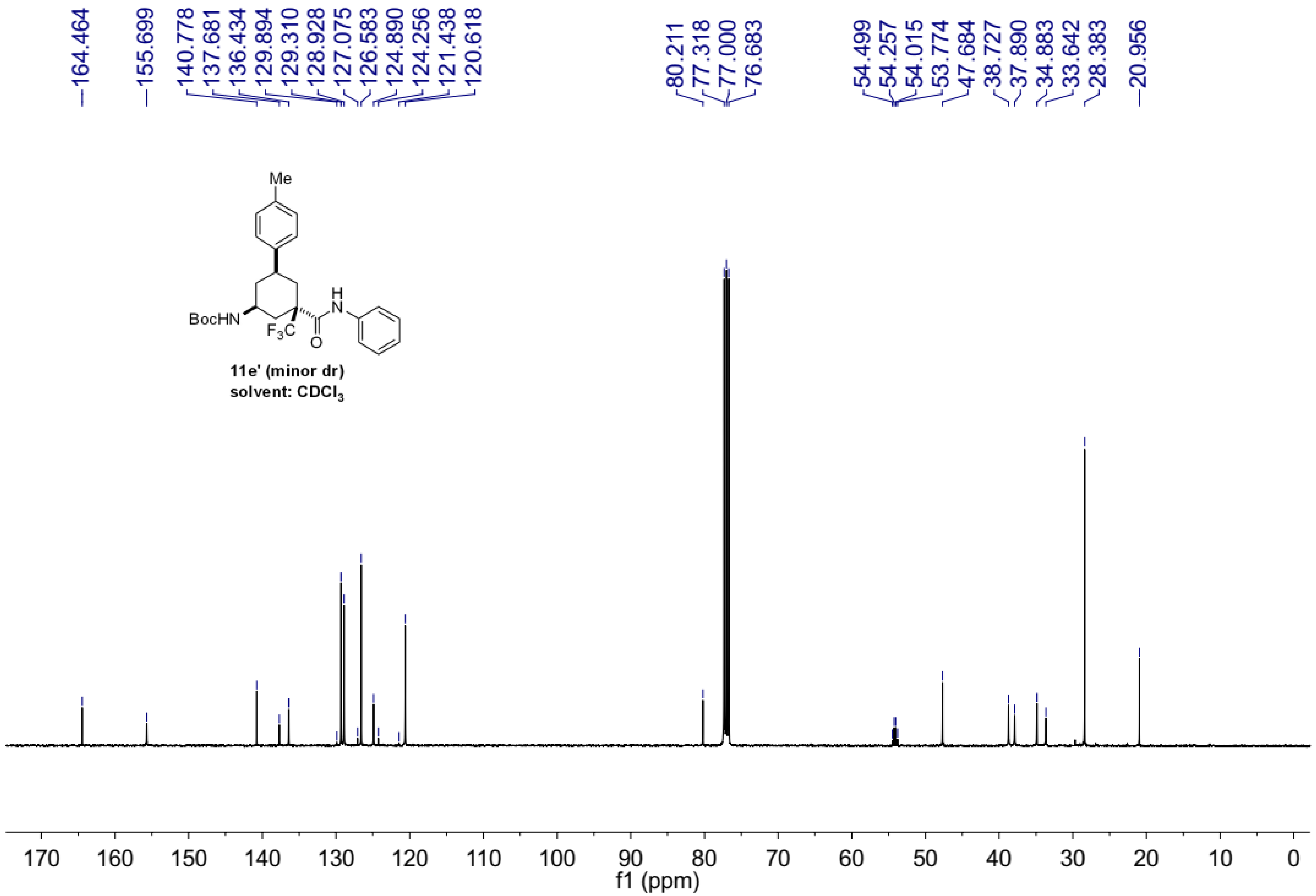

Supplementary Figure 212. ^{13}C NMR spectrum for compound **11e’** (minor dr)

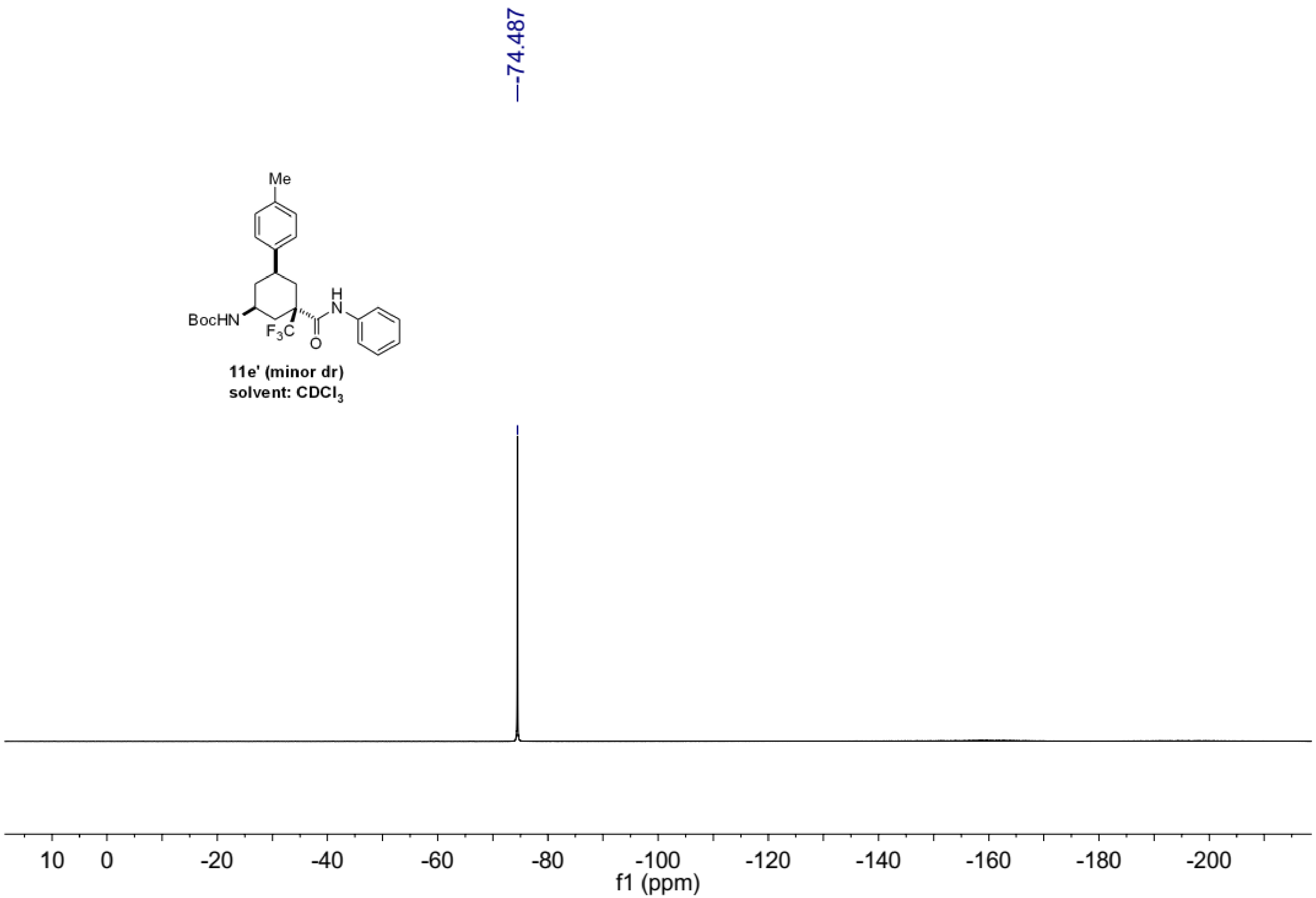

Supplementary Figure 213. ^{19}F NMR spectrum for compound **11e’** (minor dr)

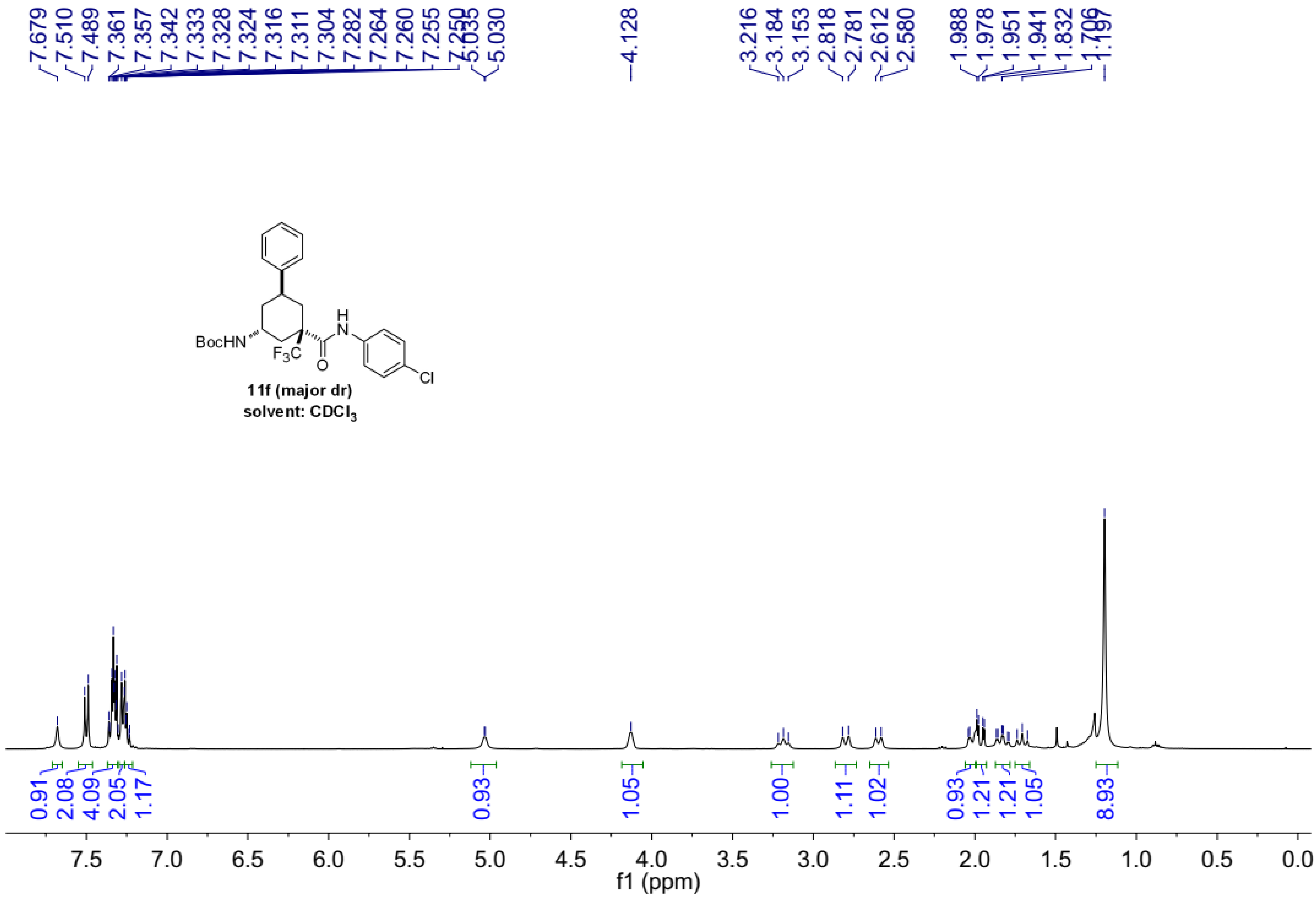

Supplementary Figure 214. ^{1}H NMR spectrum for compound **11f** (major dr)

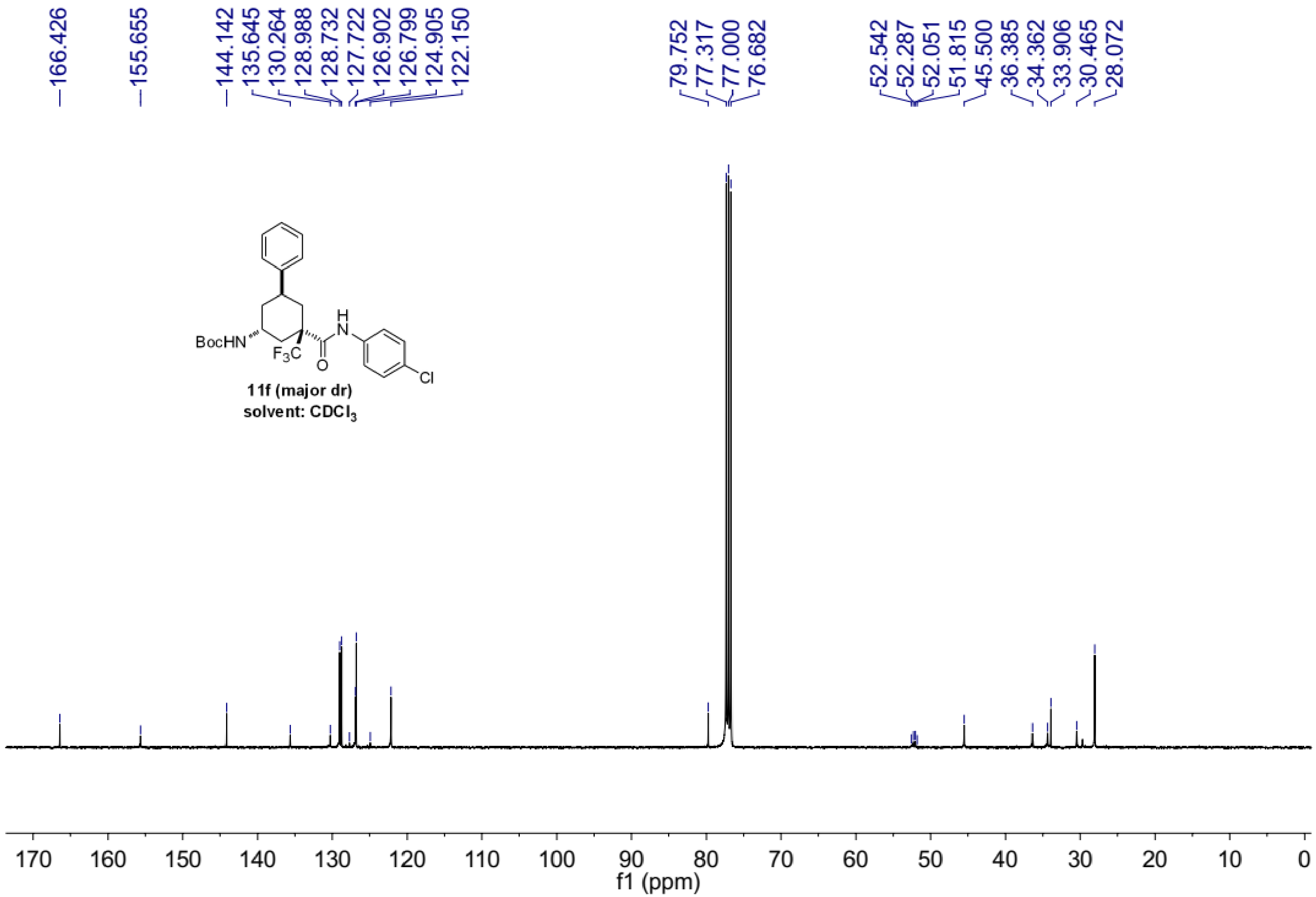

Supplementary Figure 215. ^{13}C NMR spectrum for compound **11f** (major dr)

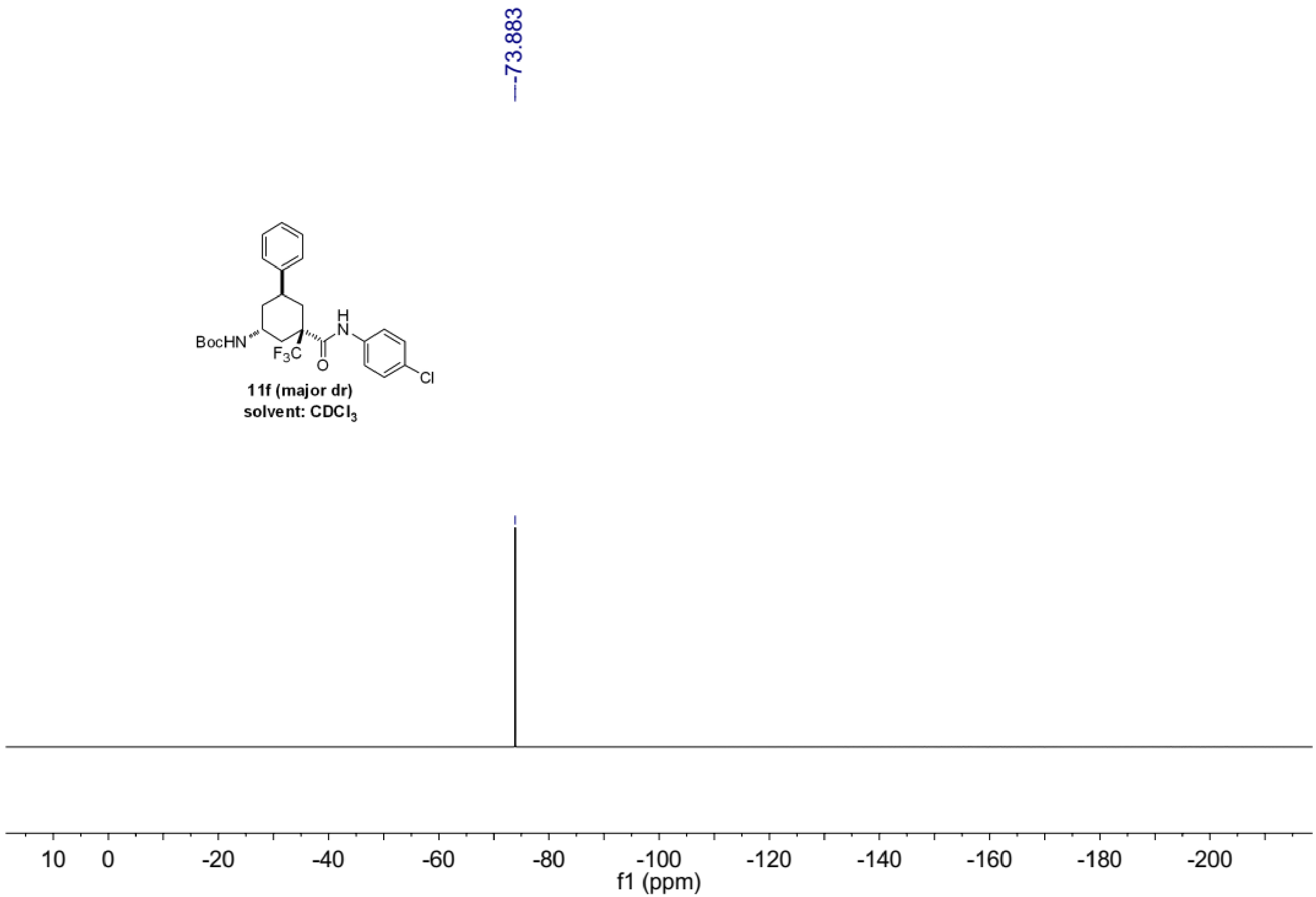

Supplementary Figure 216. ^{19}F NMR spectrum for compound **11f** (major dr)

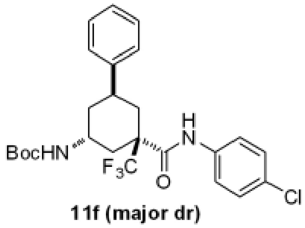

11f (major dr)
solvent: $CDCl_3$
DEPT-135

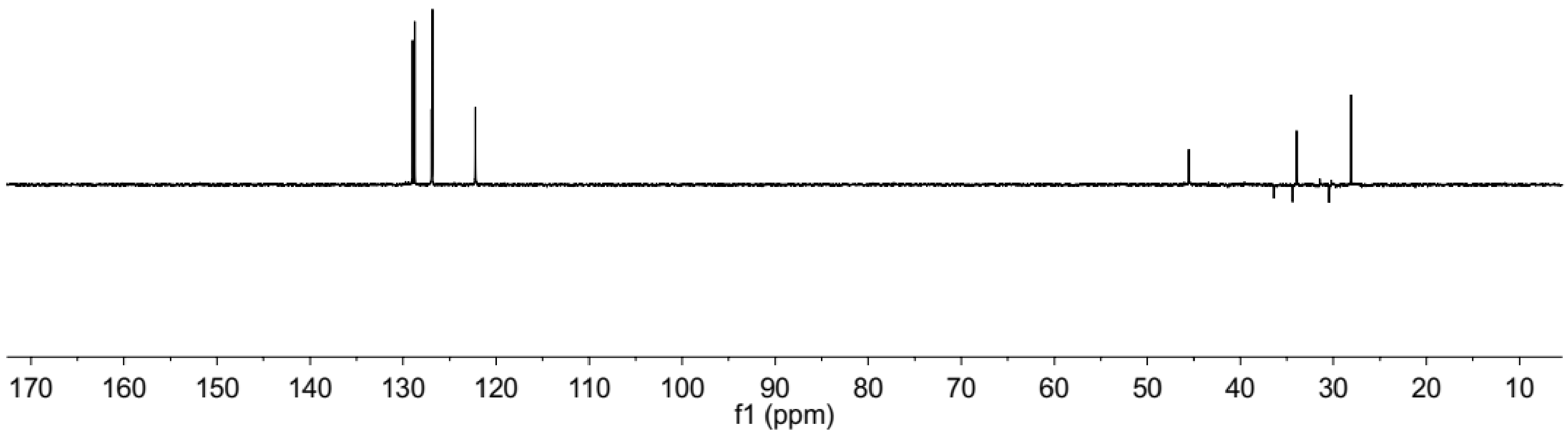

Supplementary Figure 217. DEPT-135 spectrum for compound **11f** (major dr)

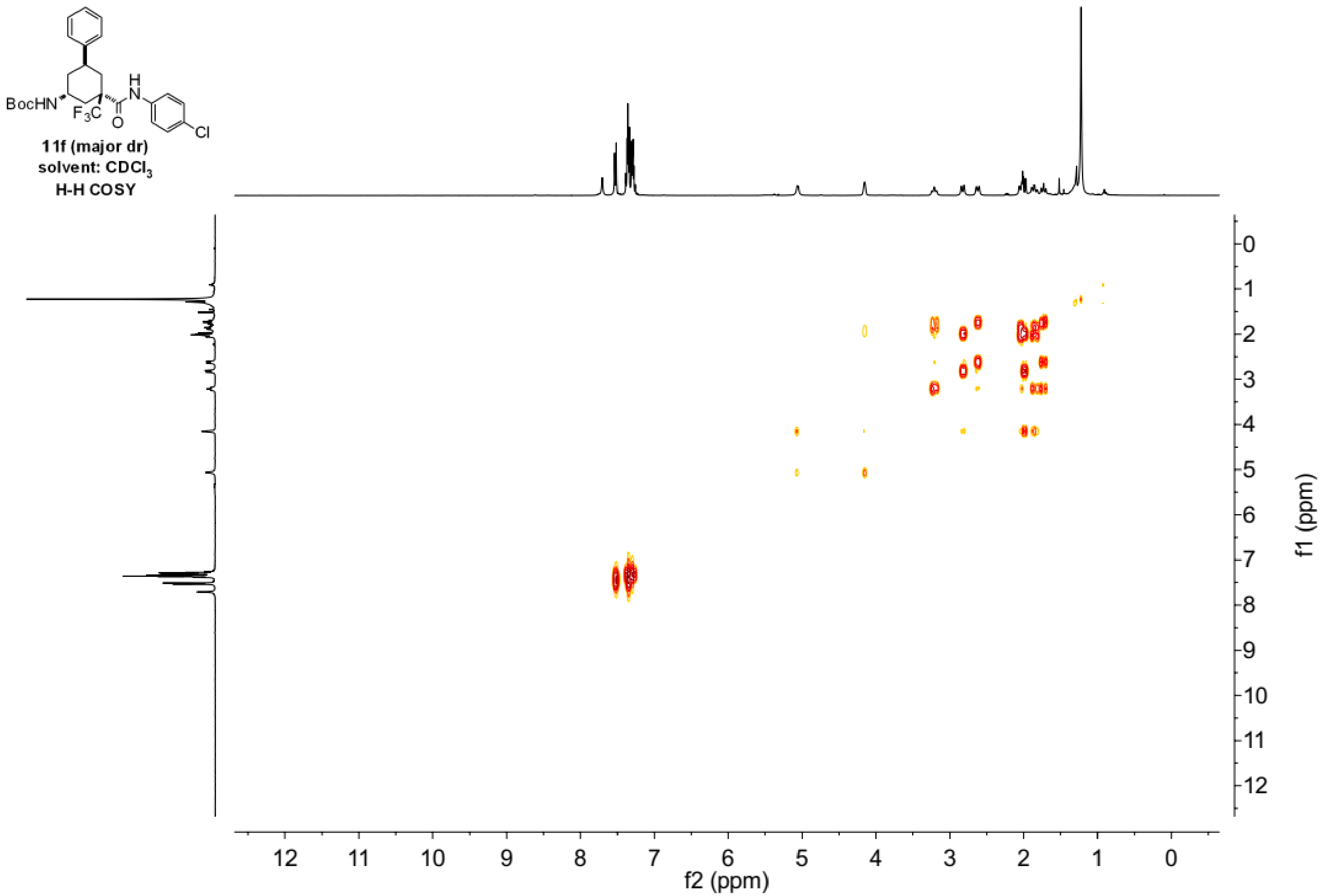

Supplementary Figure 218. H-H COSY spectrum for compound **11f** (major dr)

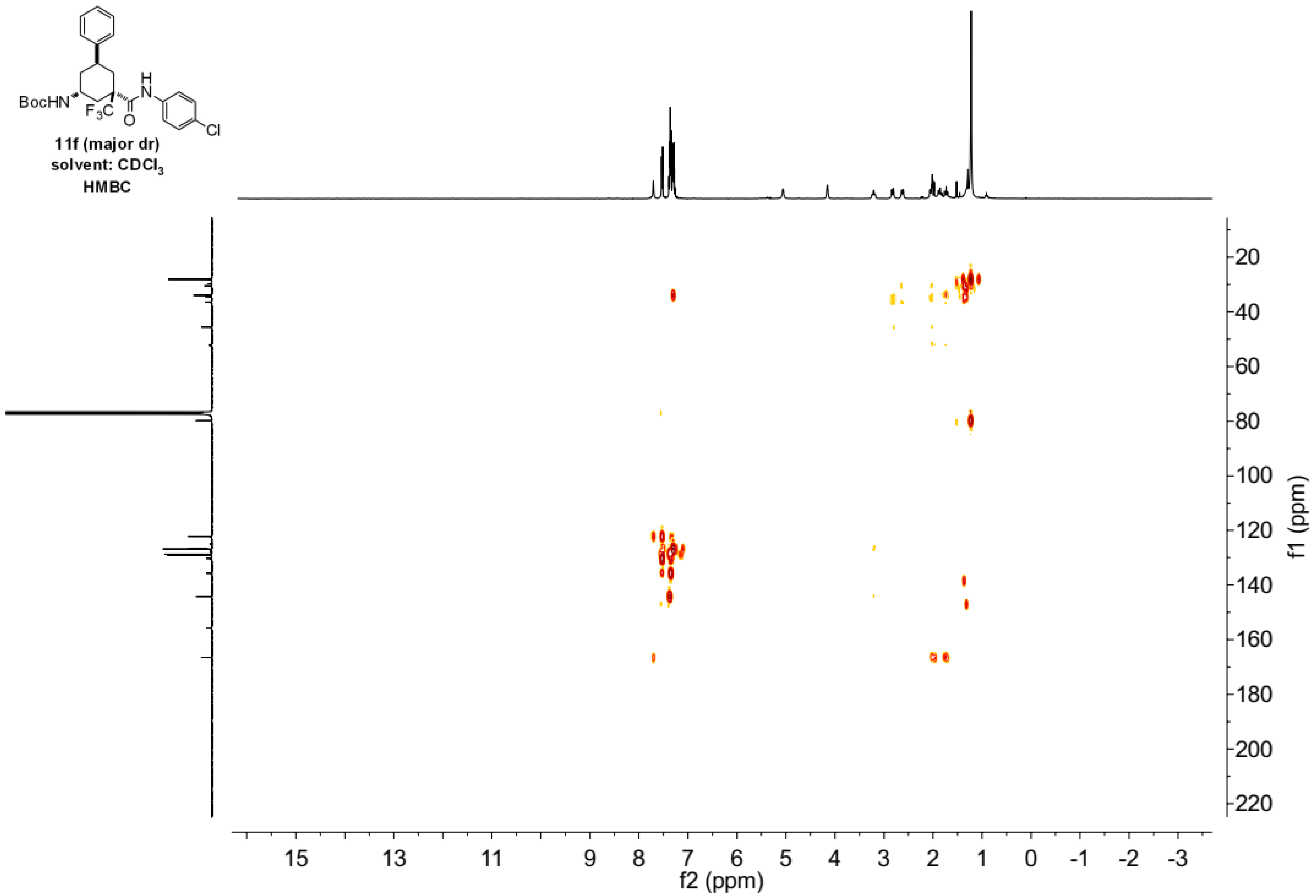

Supplementary Figure 219. HMBC spectrum for compound **11f** (major dr)

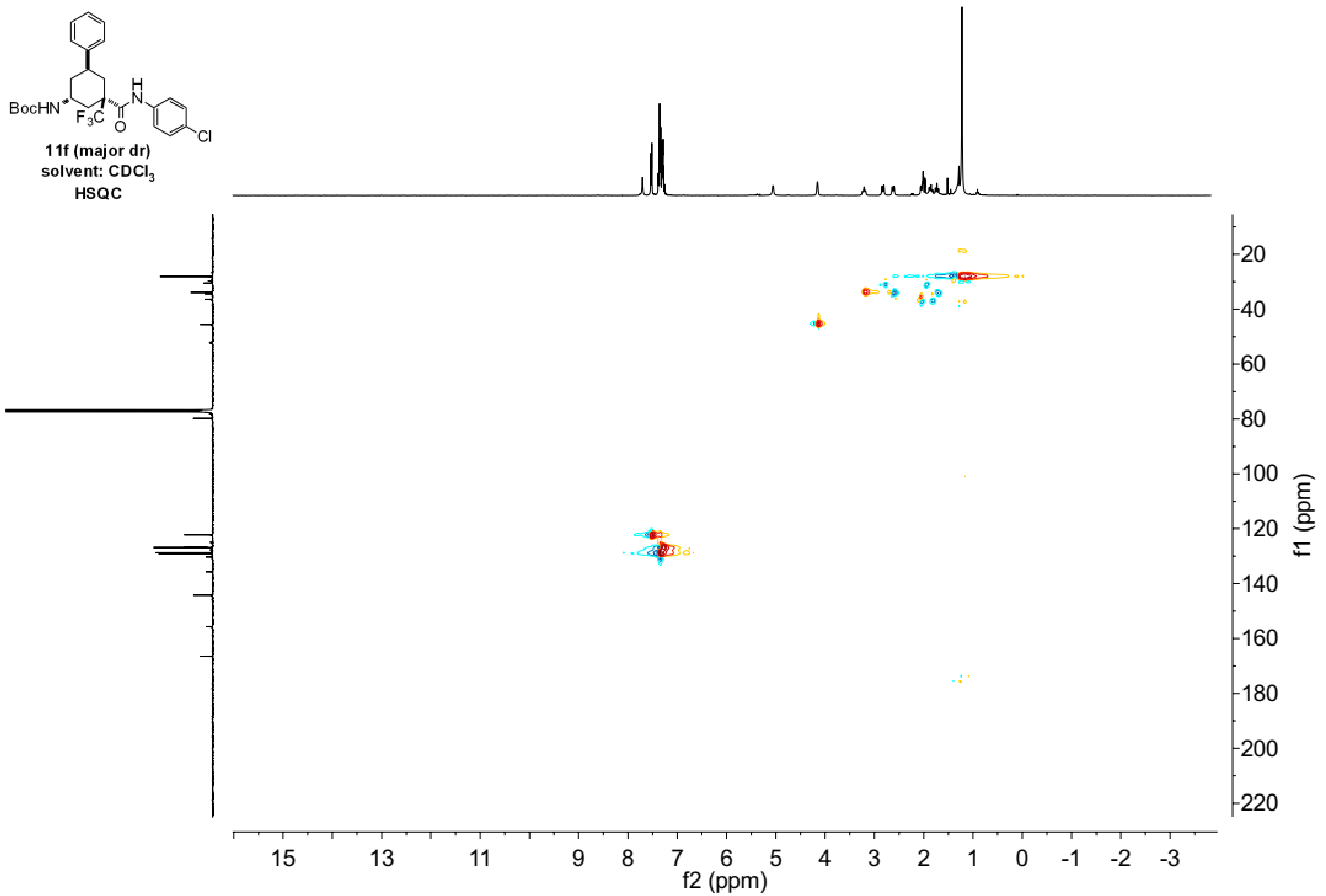

Supplementary Figure 220. HSQC spectrum for compound **11f** (major dr)

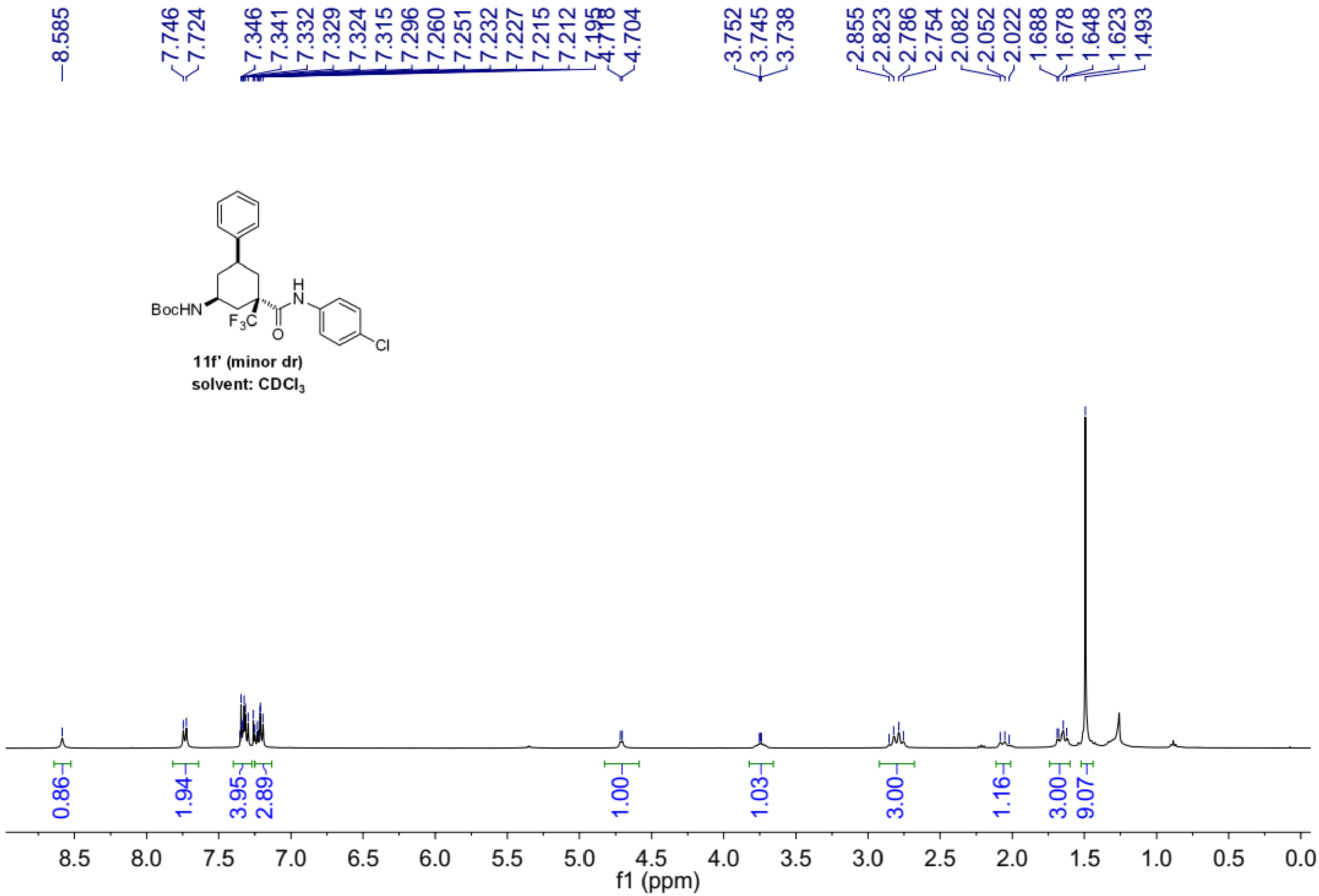

Supplementary Figure 221. ^{1}H NMR spectrum for compound **11f’** (minor dr)

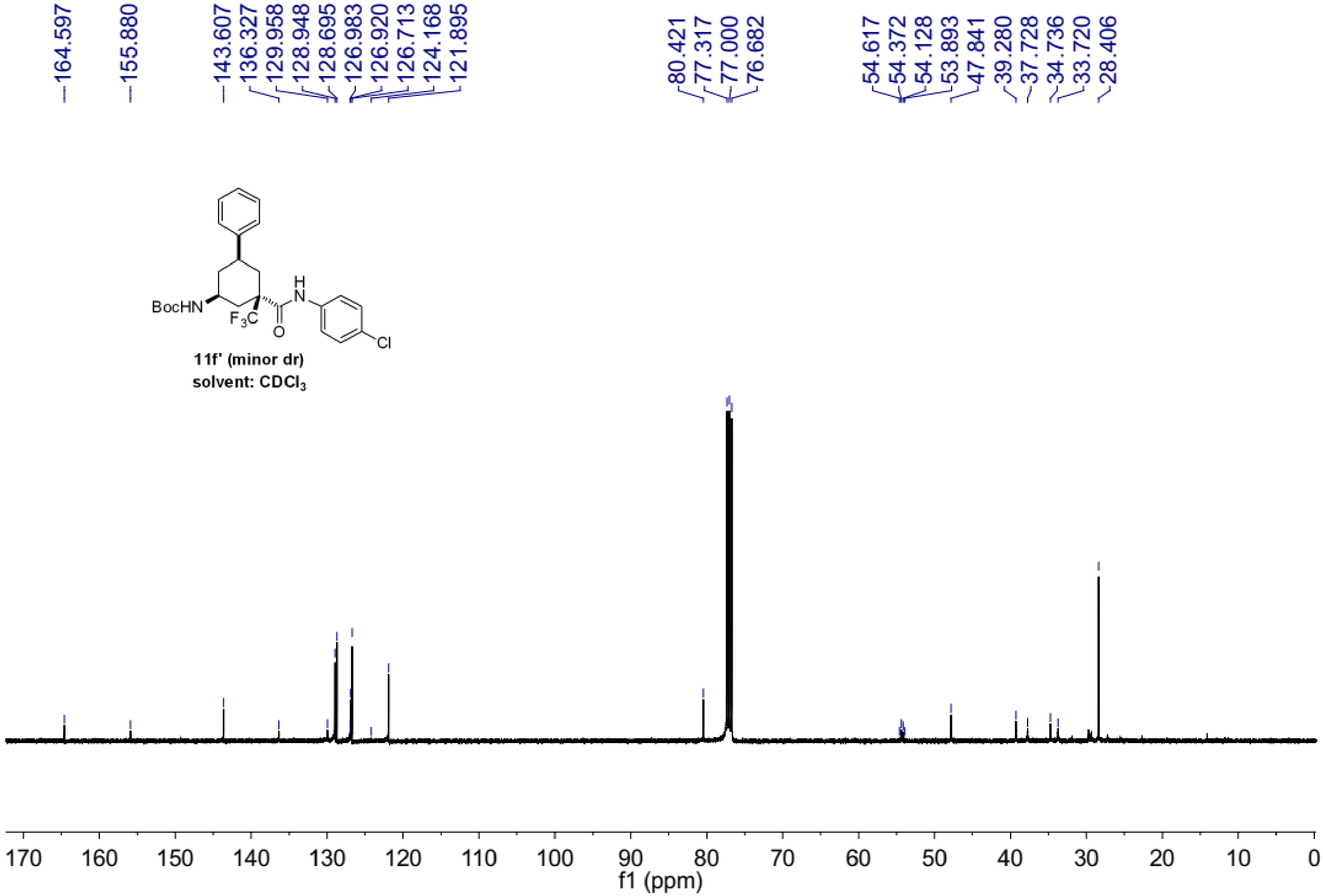

Supplementary Figure 222. ^{13}C NMR spectrum for compound **11f’** (minor dr)

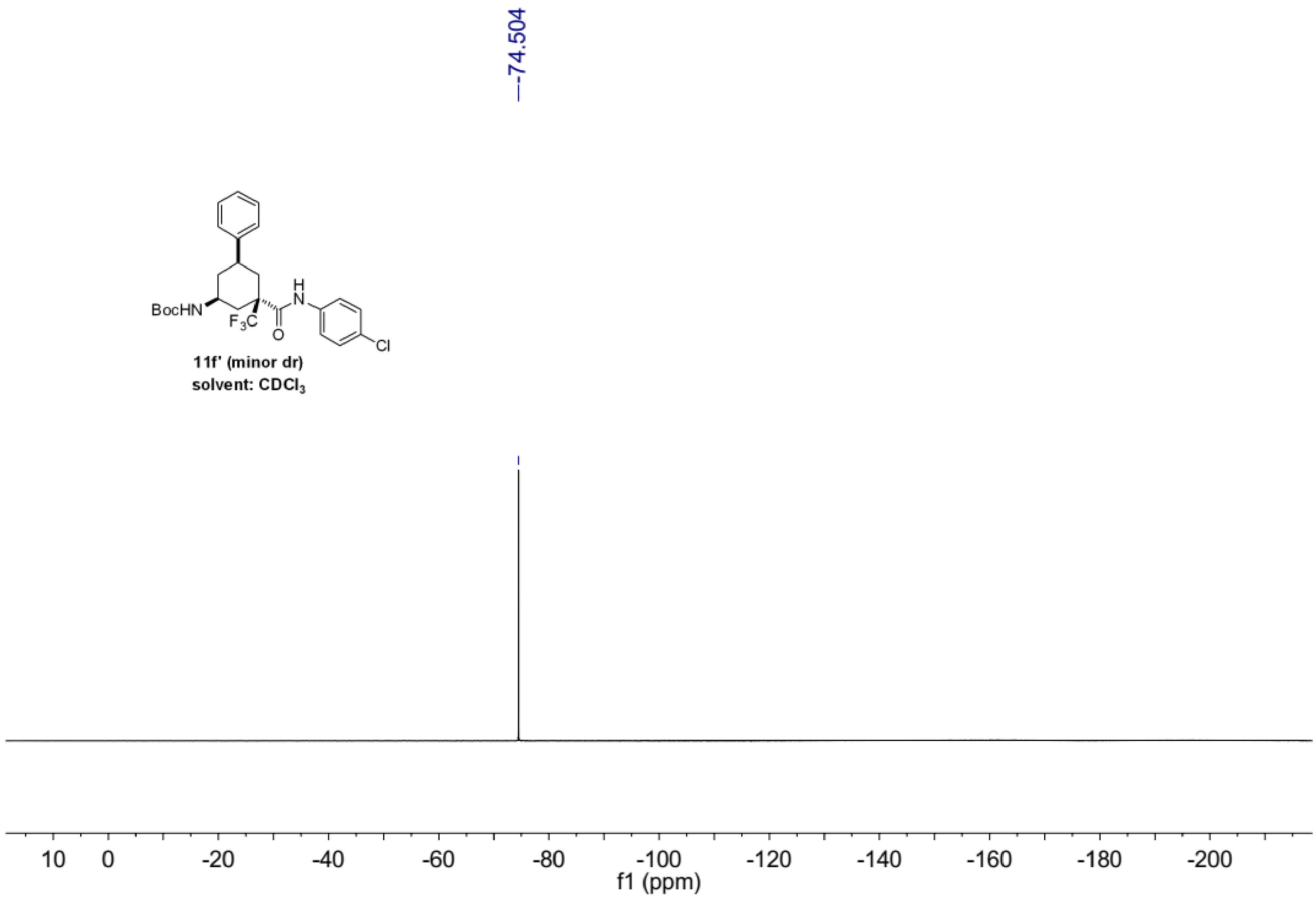

Supplementary Figure 223. ^{19}F NMR spectrum for compound **11f’** (minor dr)

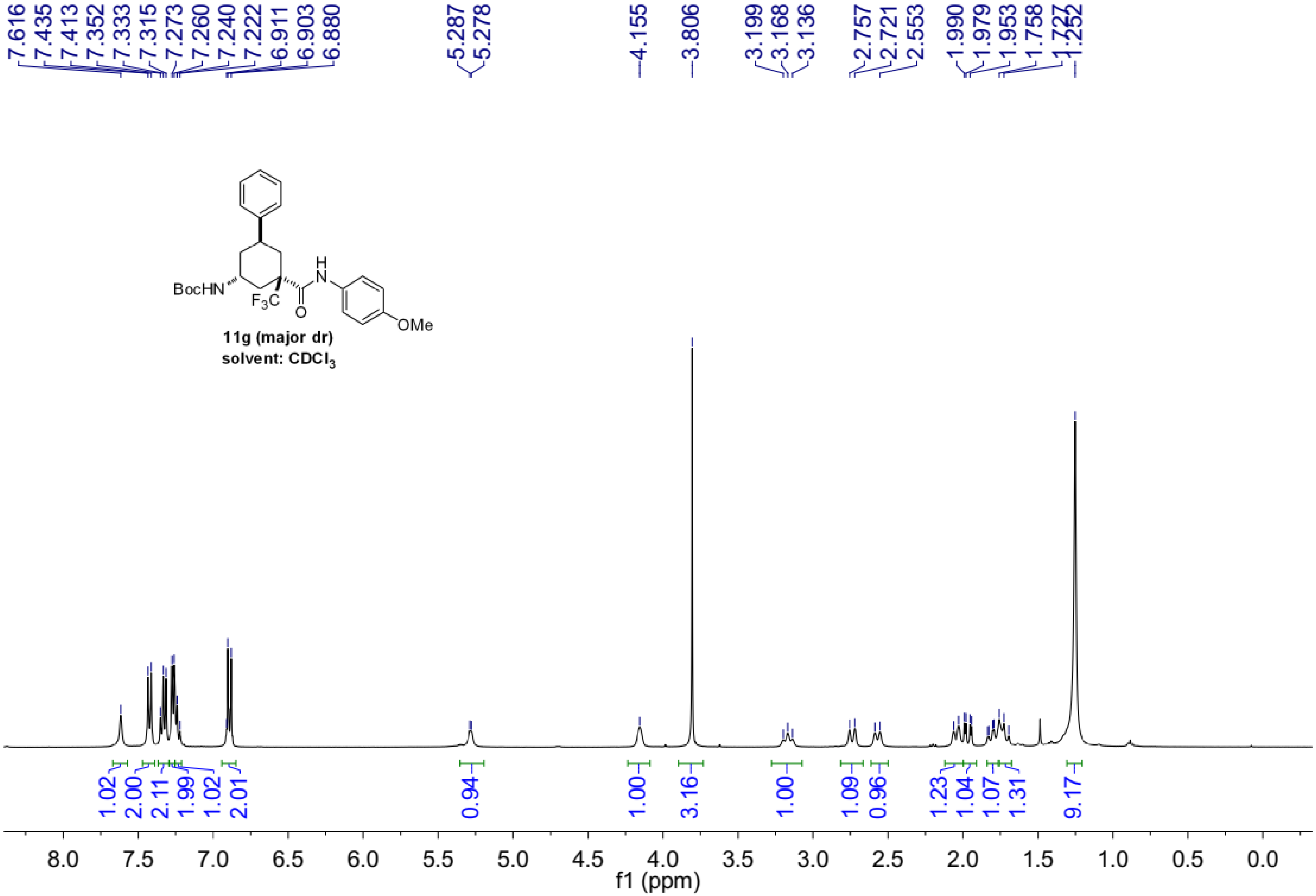

Supplementary Figure 224. ^{1}H NMR spectrum for compound **11g** (major dr)

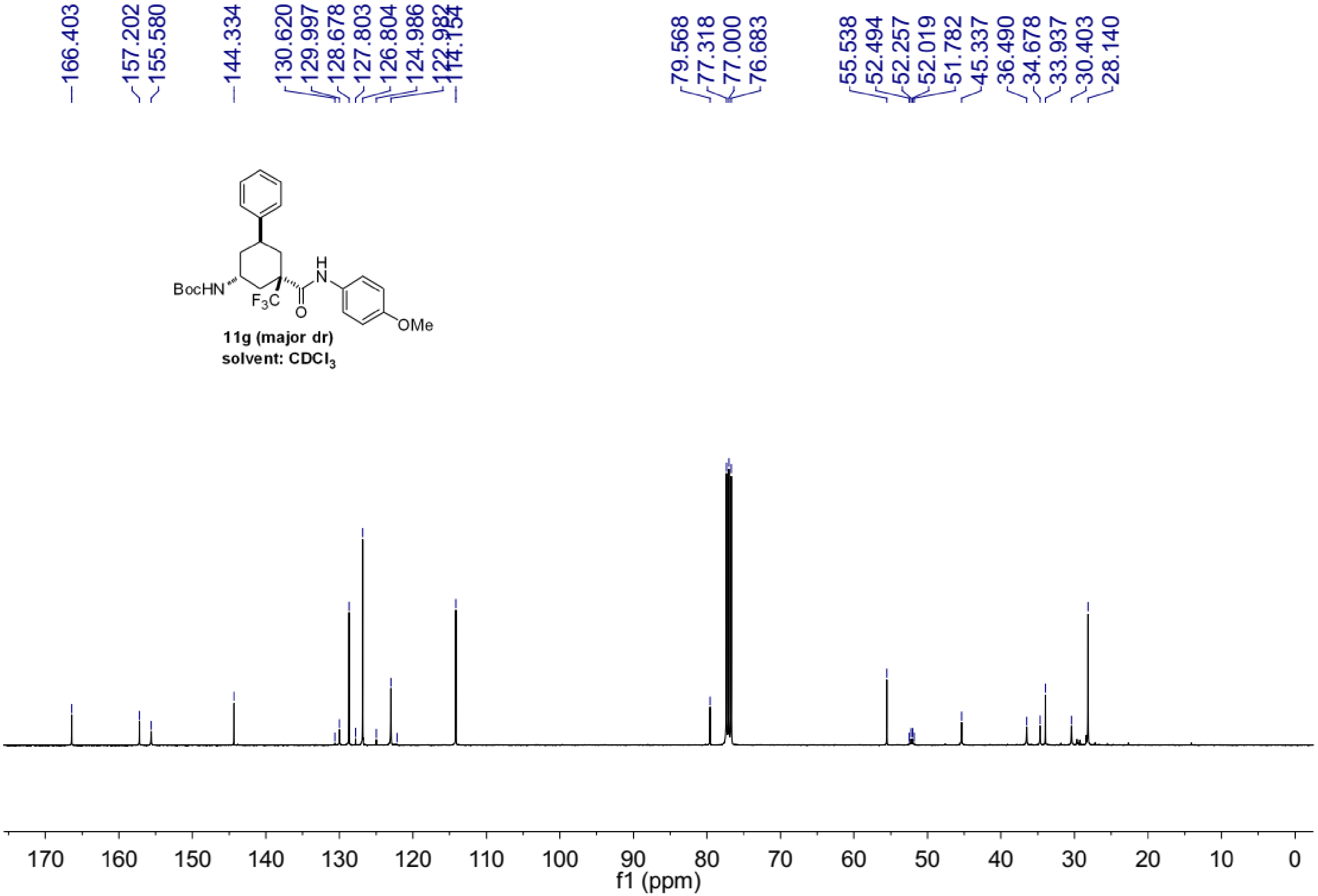

Supplementary Figure 225. ^{13}C NMR spectrum for compound **11g** (major dr)

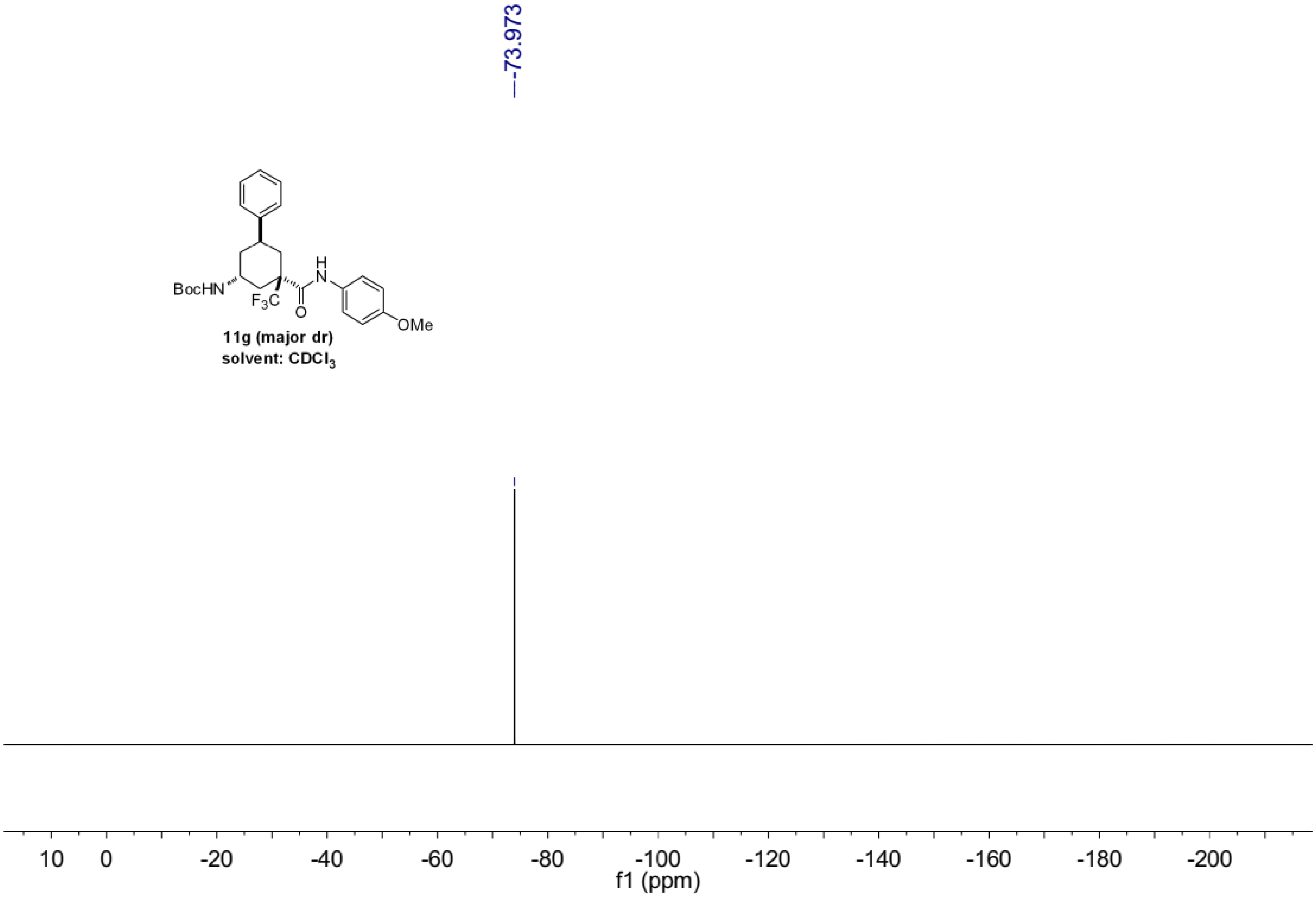

Supplementary Figure 226. ^{19}F NMR spectrum for compound **11g** (major dr)

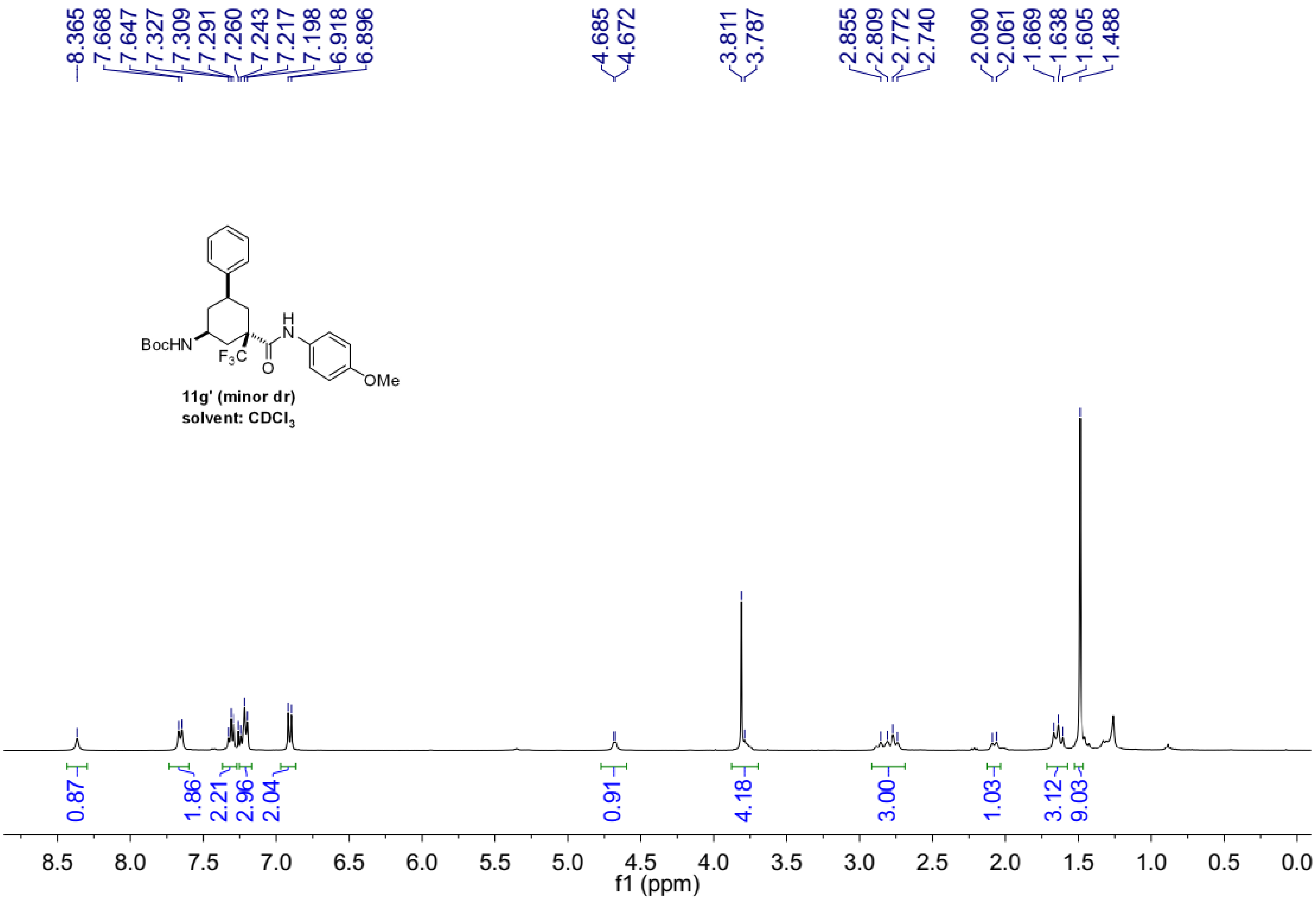

Supplementary Figure 227. ^{1}H NMR spectrum for compound **11g'** (minor dr)

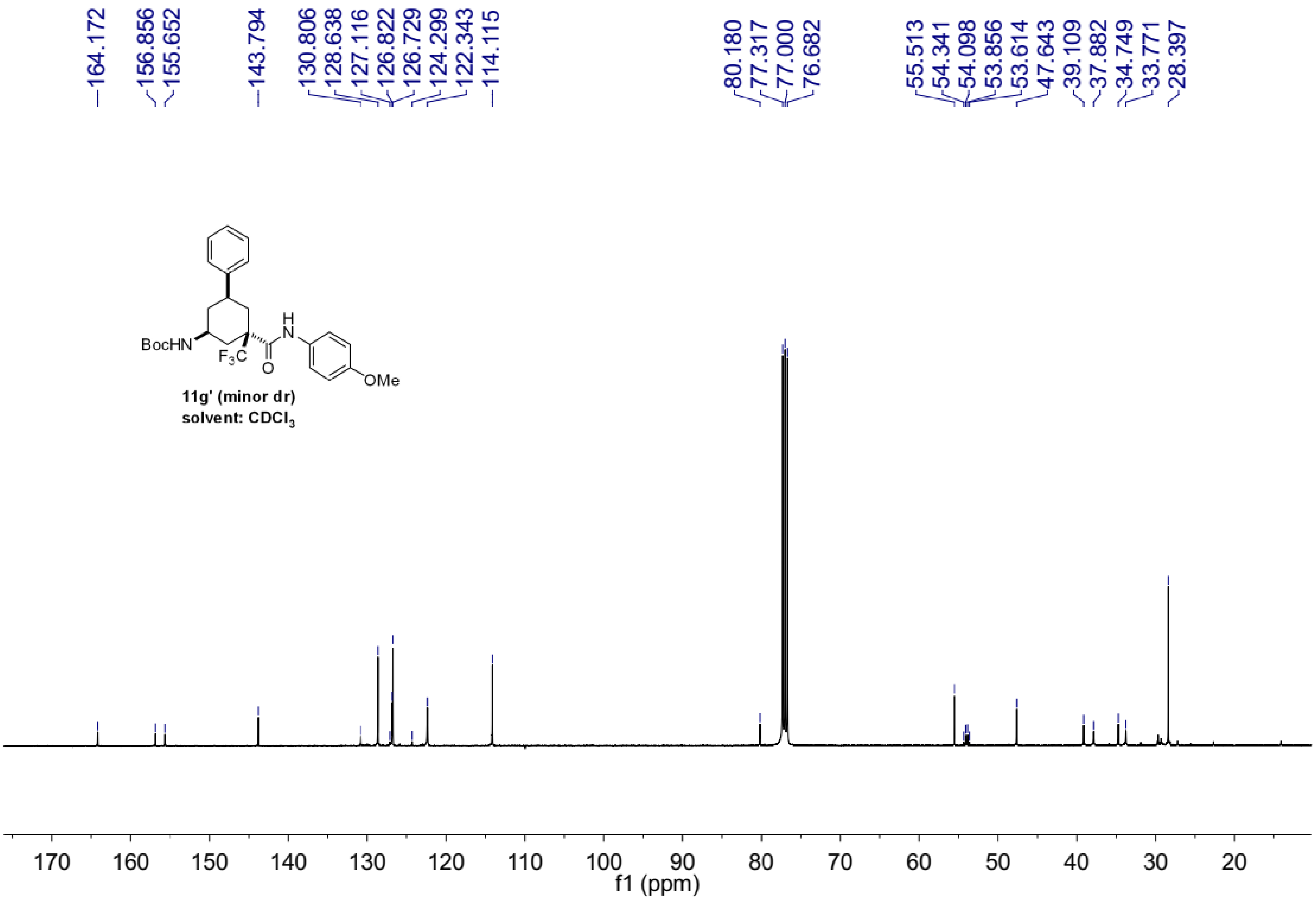

Supplementary Figure 228. ^{13}C NMR spectrum for compound **11g'** (minor dr)

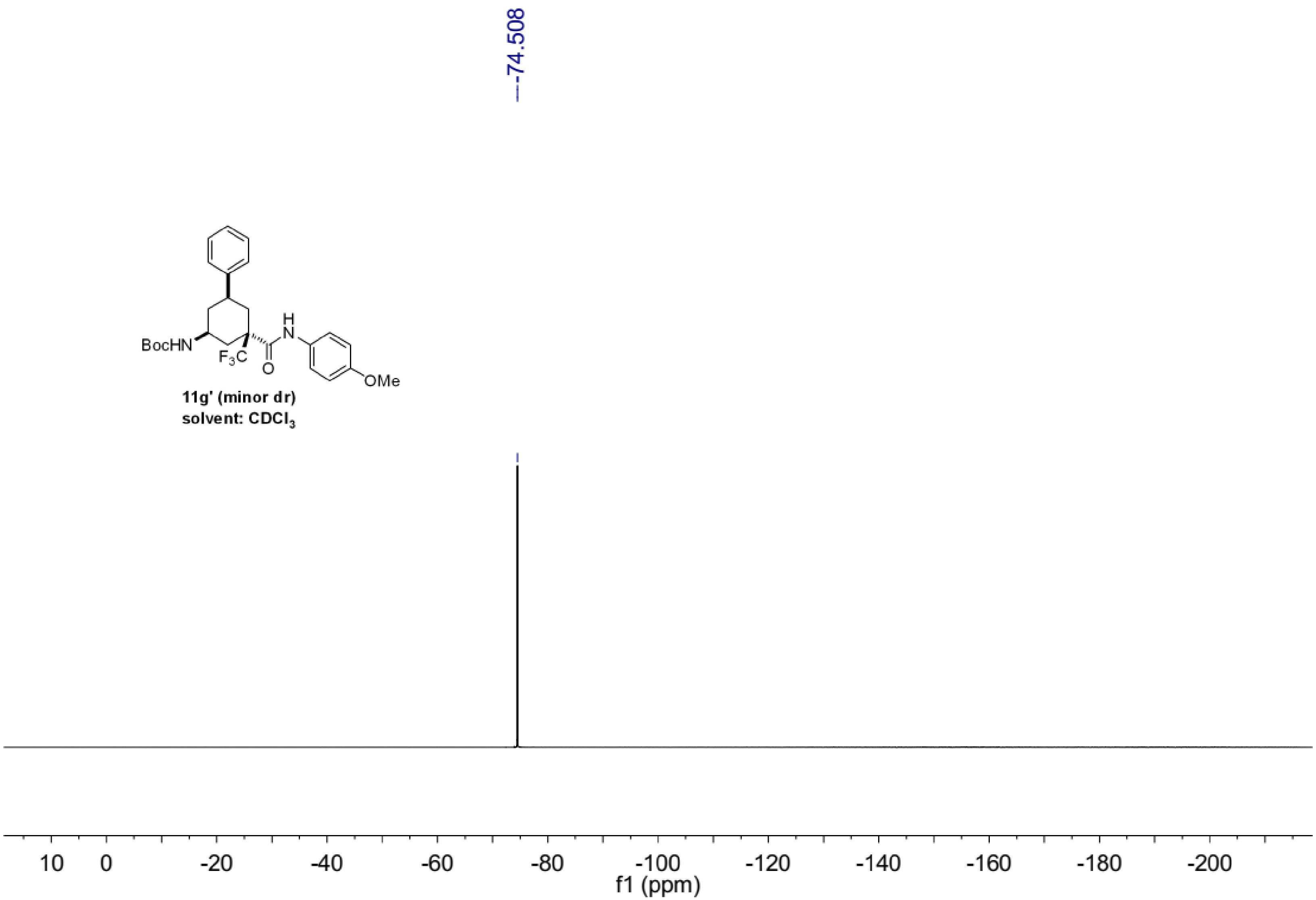

Supplementary Figure 229. ^{19}F NMR spectrum for compound **11g’** (minor dr)

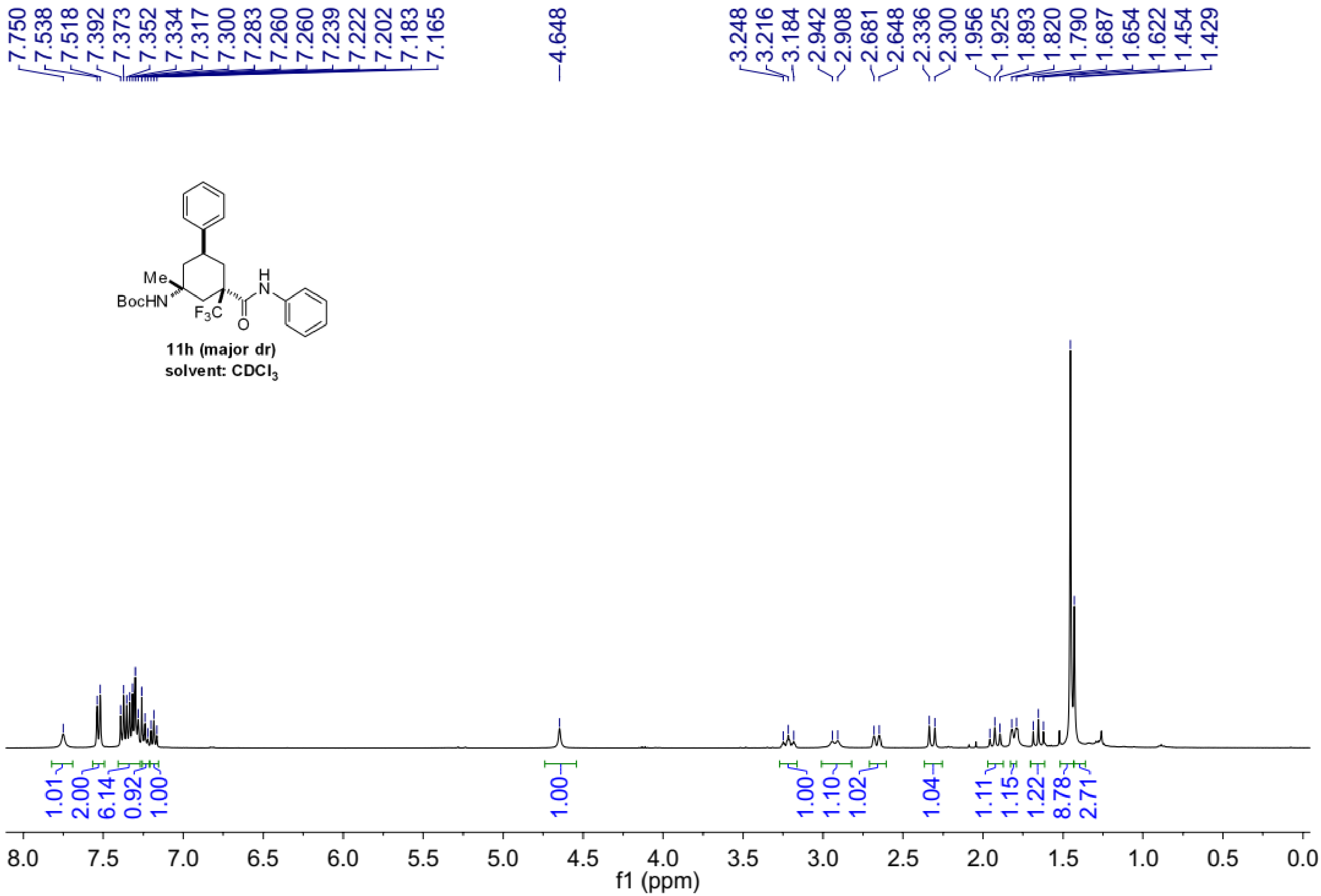

Supplementary Figure 230. ^{1}H NMR spectrum for compound **11h** (major dr)

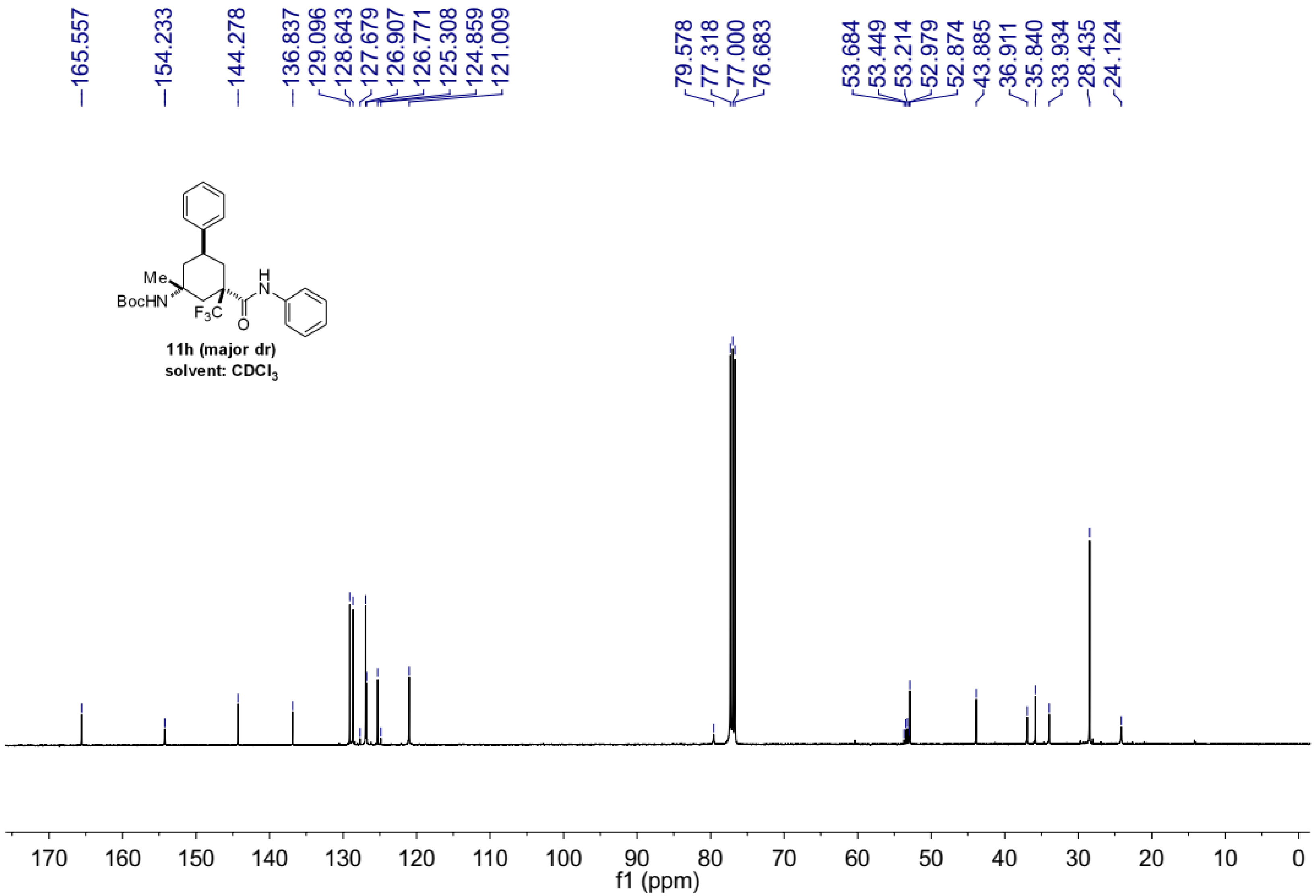

Supplementary Figure 231. ^{13}C NMR spectrum for compound **11h** (major dr)

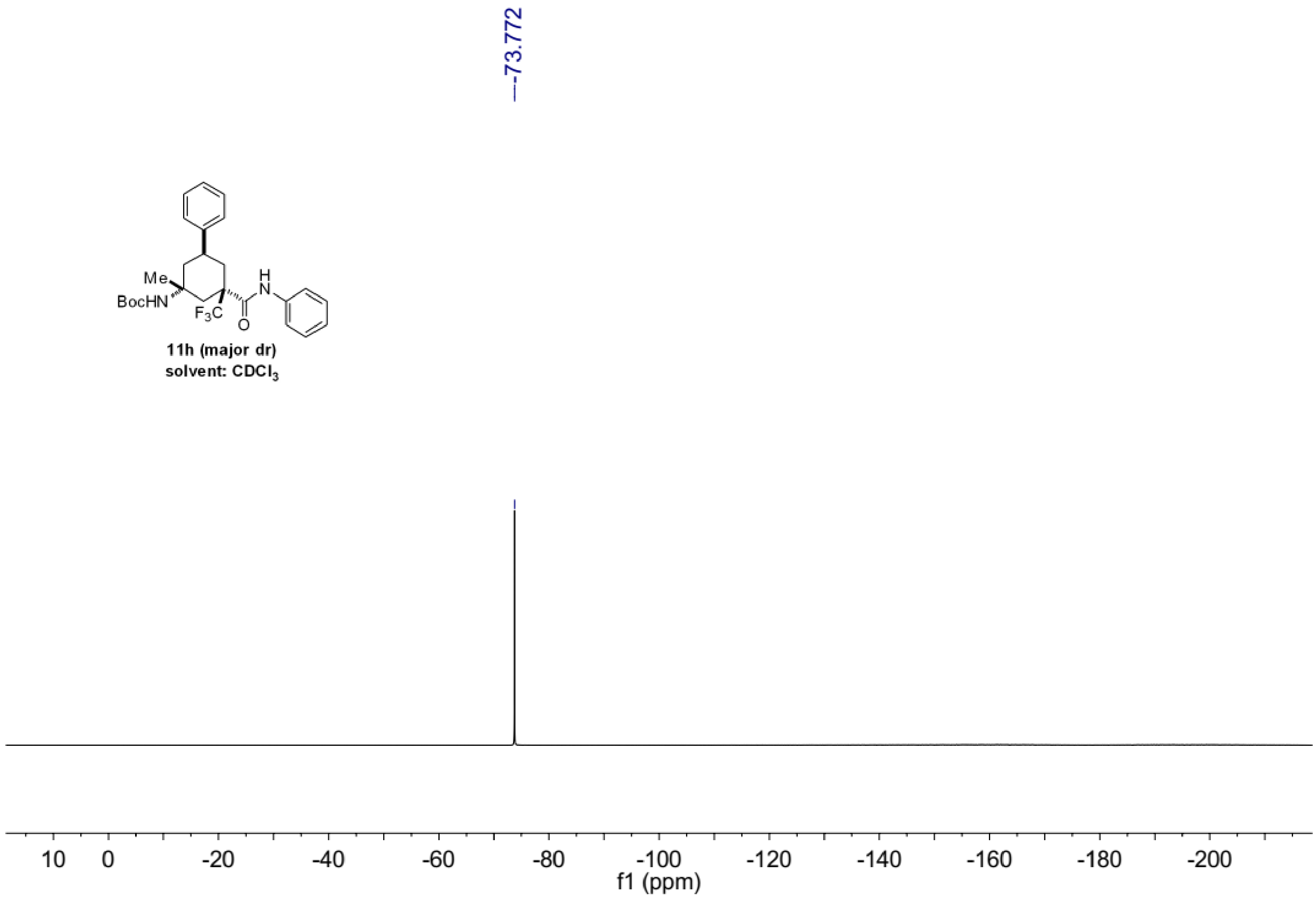

Supplementary Figure 232. ^{19}F NMR spectrum for compound **11h** (major dr)

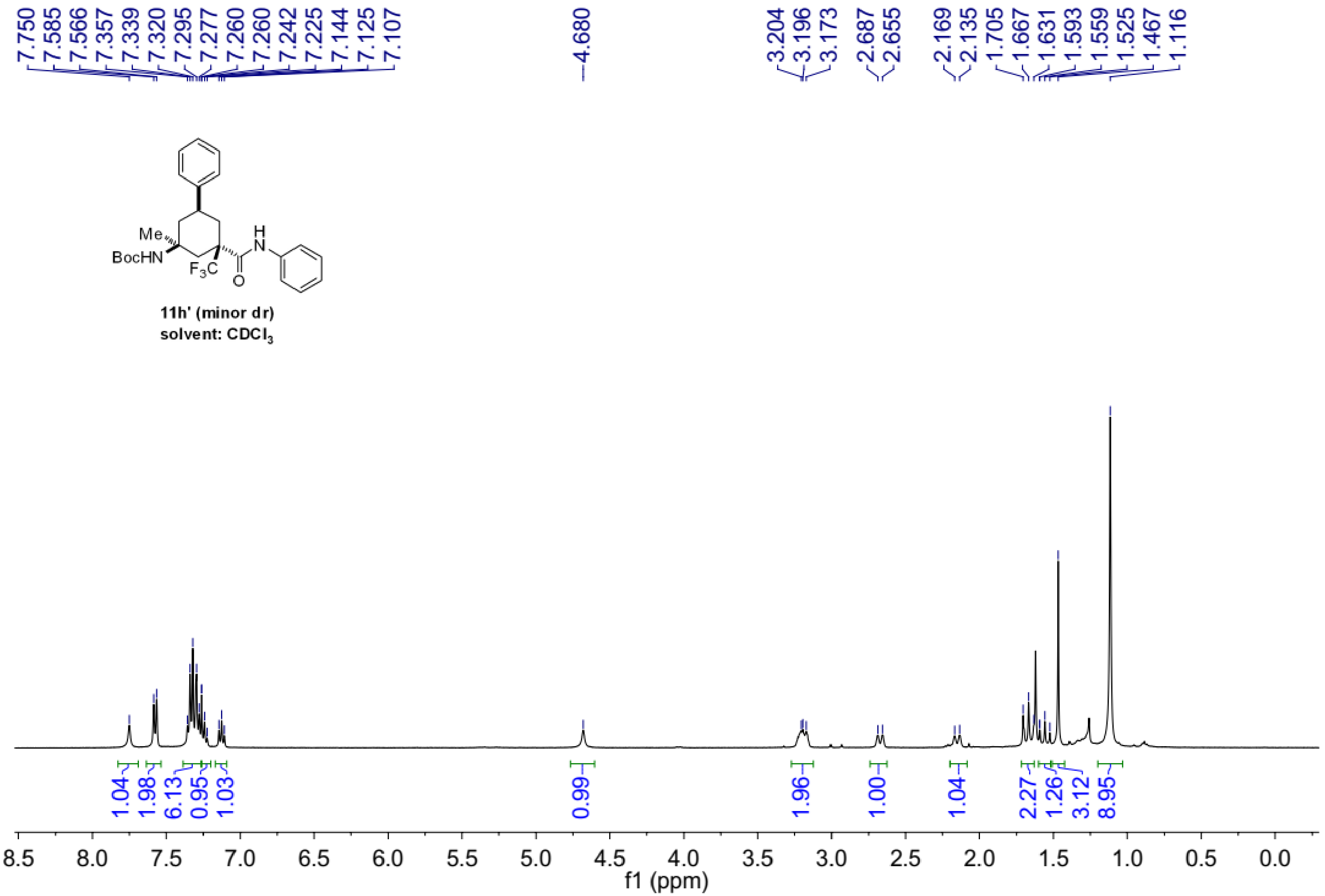

Supplementary Figure 233. ^{1}H NMR spectrum for compound **11h’** (minor dr)

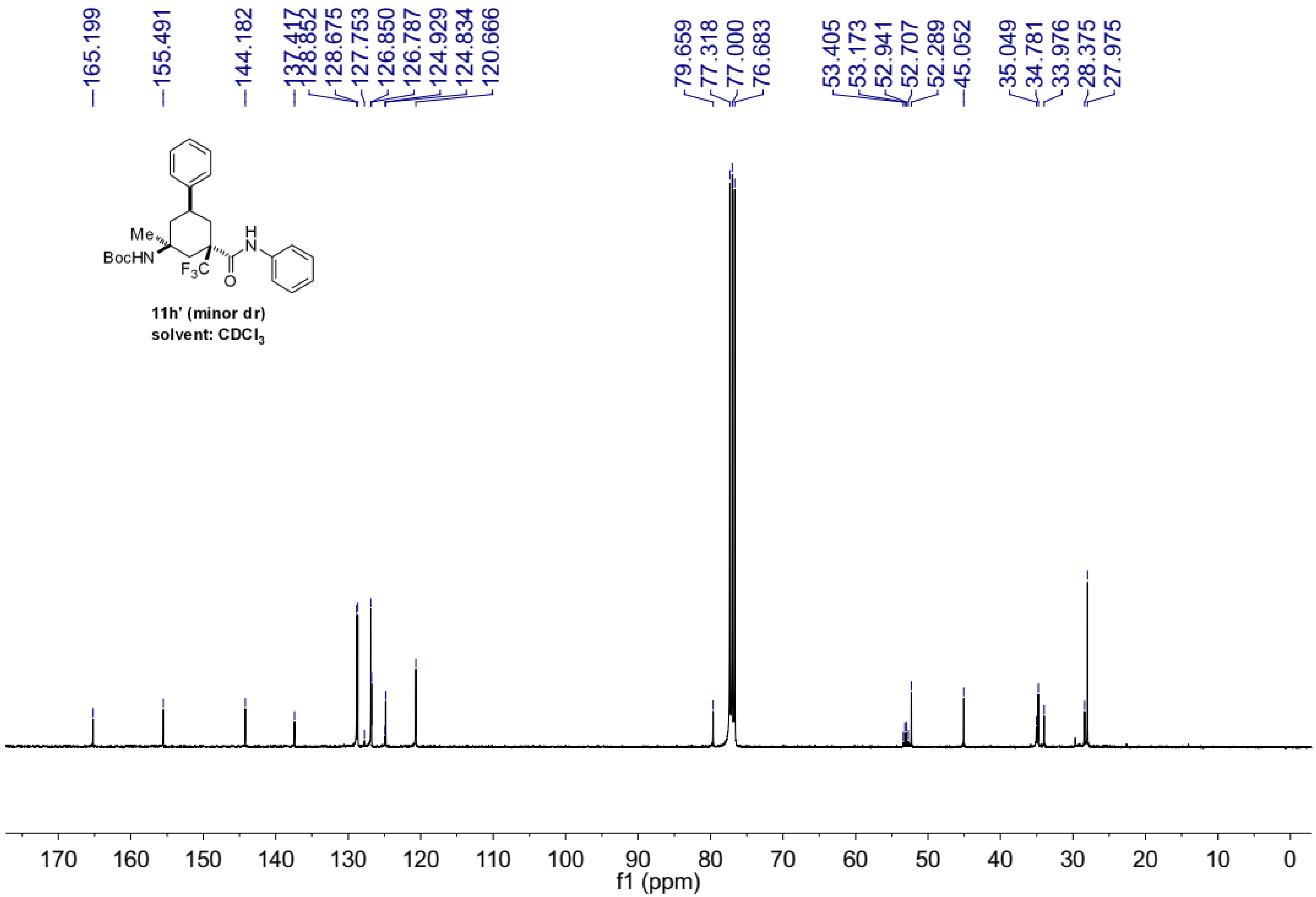

Supplementary Figure 234. ^{13}C NMR spectrum for compound **11h’** (minor dr)

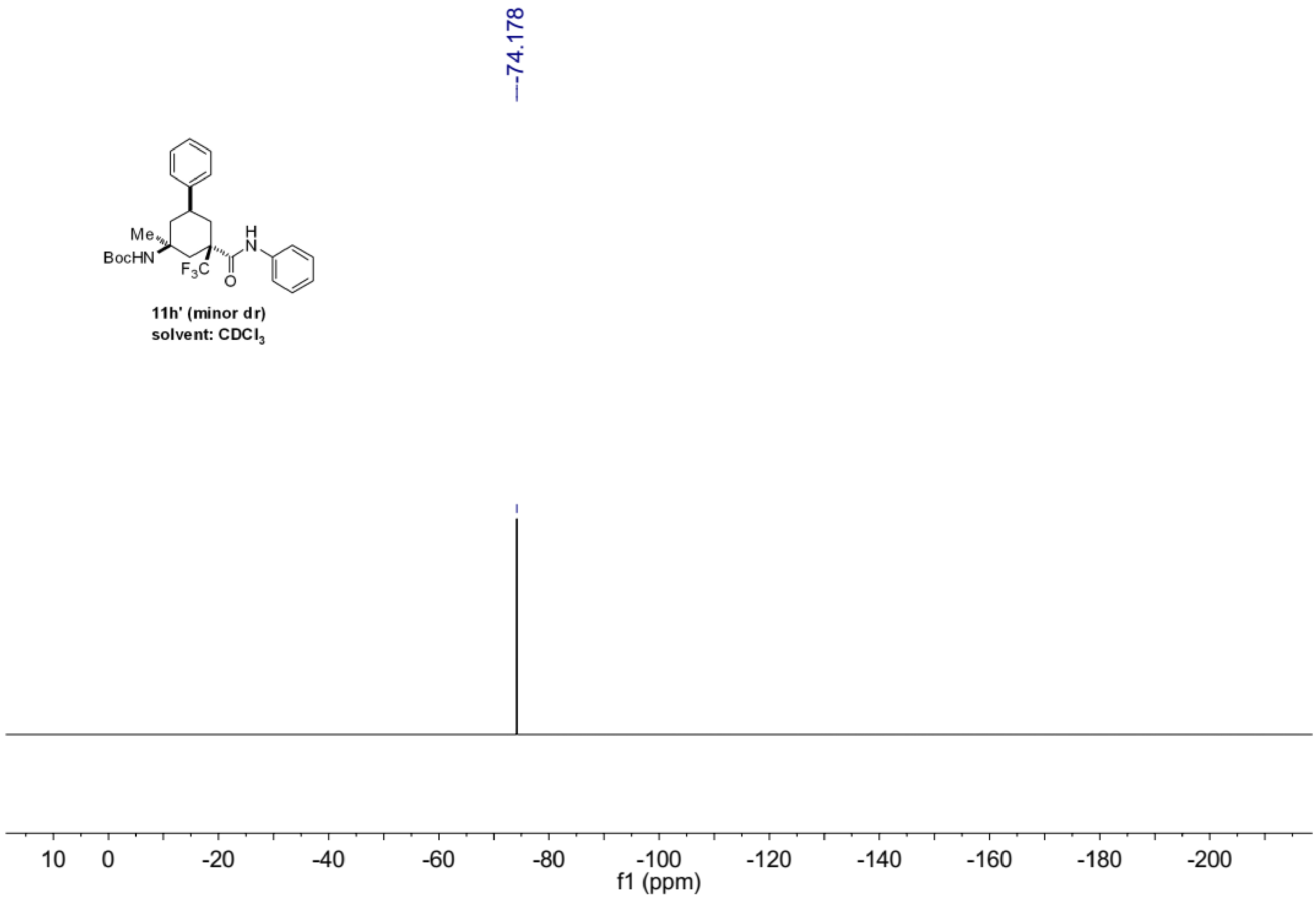

Supplementary Figure 235. ^{19}F NMR spectrum for compound **11h’** (minor dr)

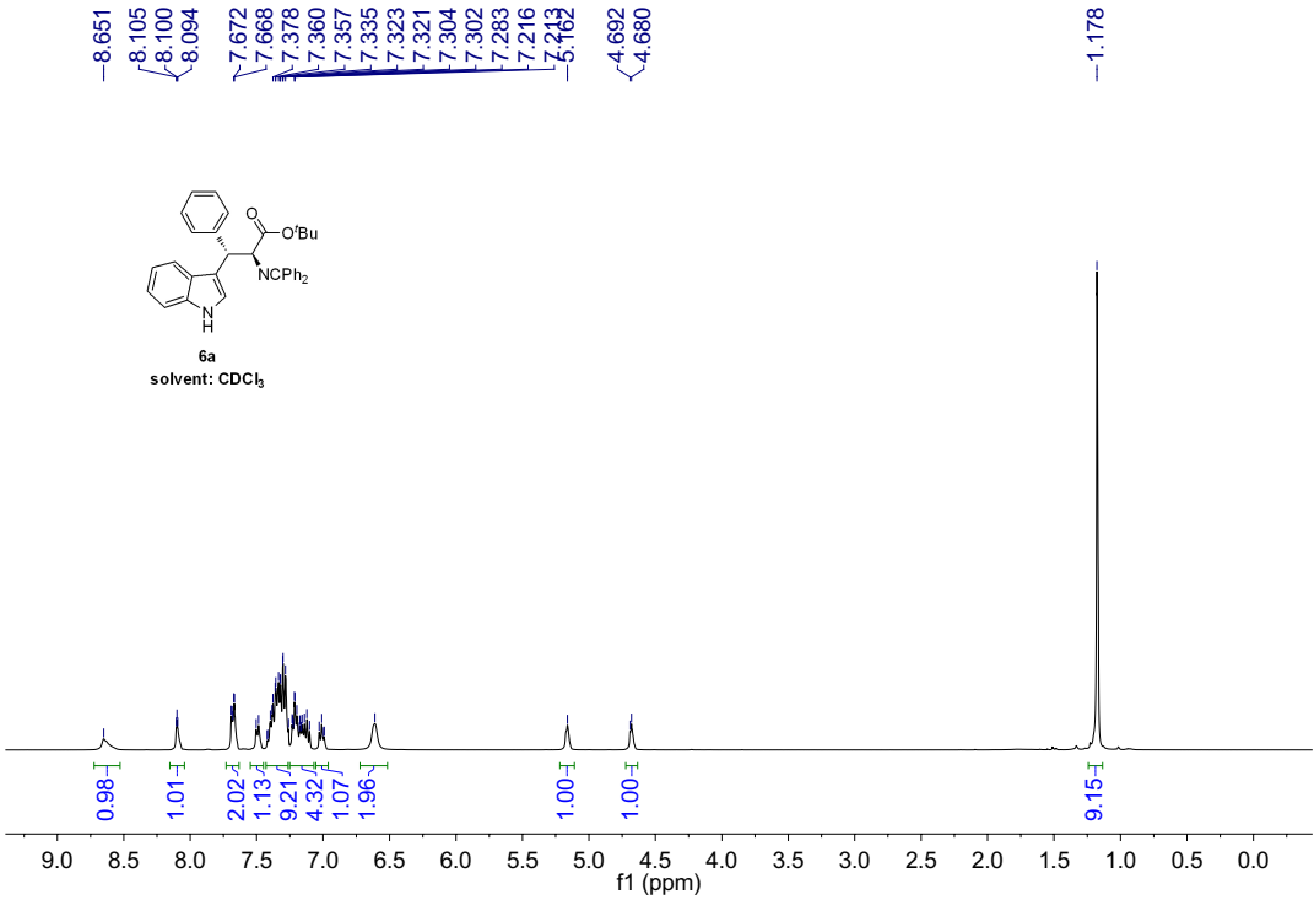

6a
solvent: $CDCl_3$

Supplementary Figure 236. ^{1}H NMR spectrum for compound **6a**

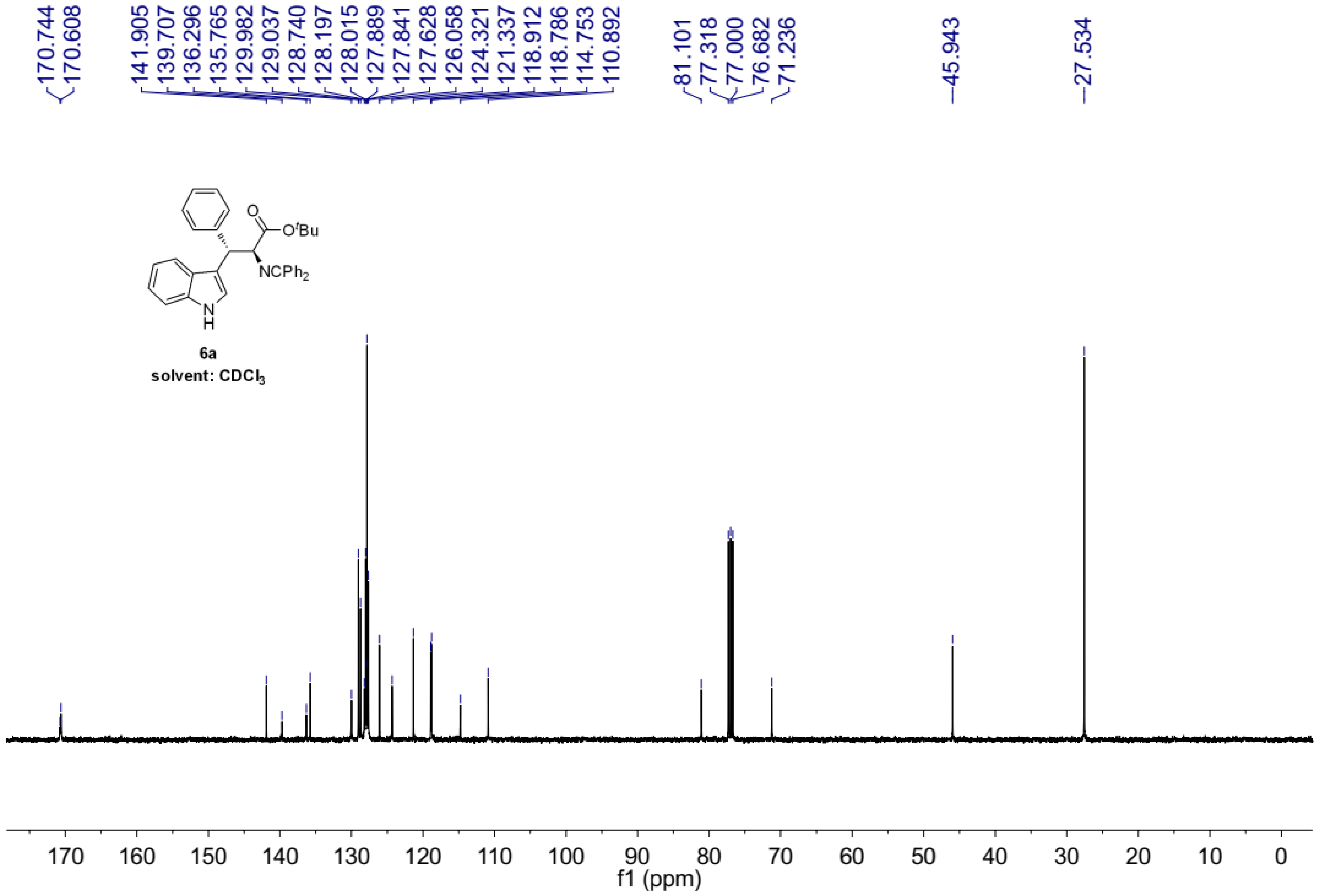

Supplementary Figure 237. ^{13}C NMR spectrum for compound **6a**

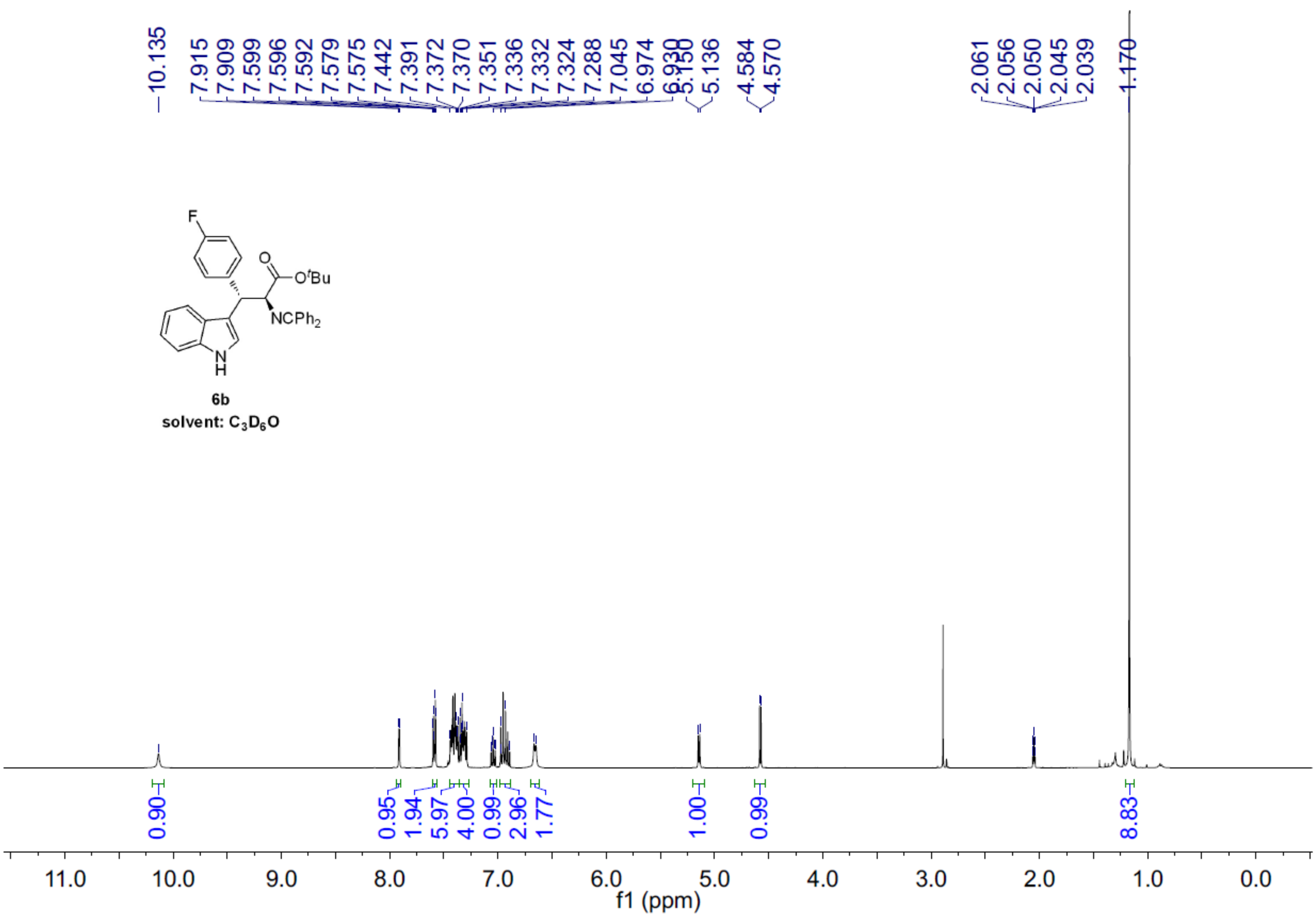

Supplementary Figure 238. ^{1}H NMR spectrum for compound **6b**

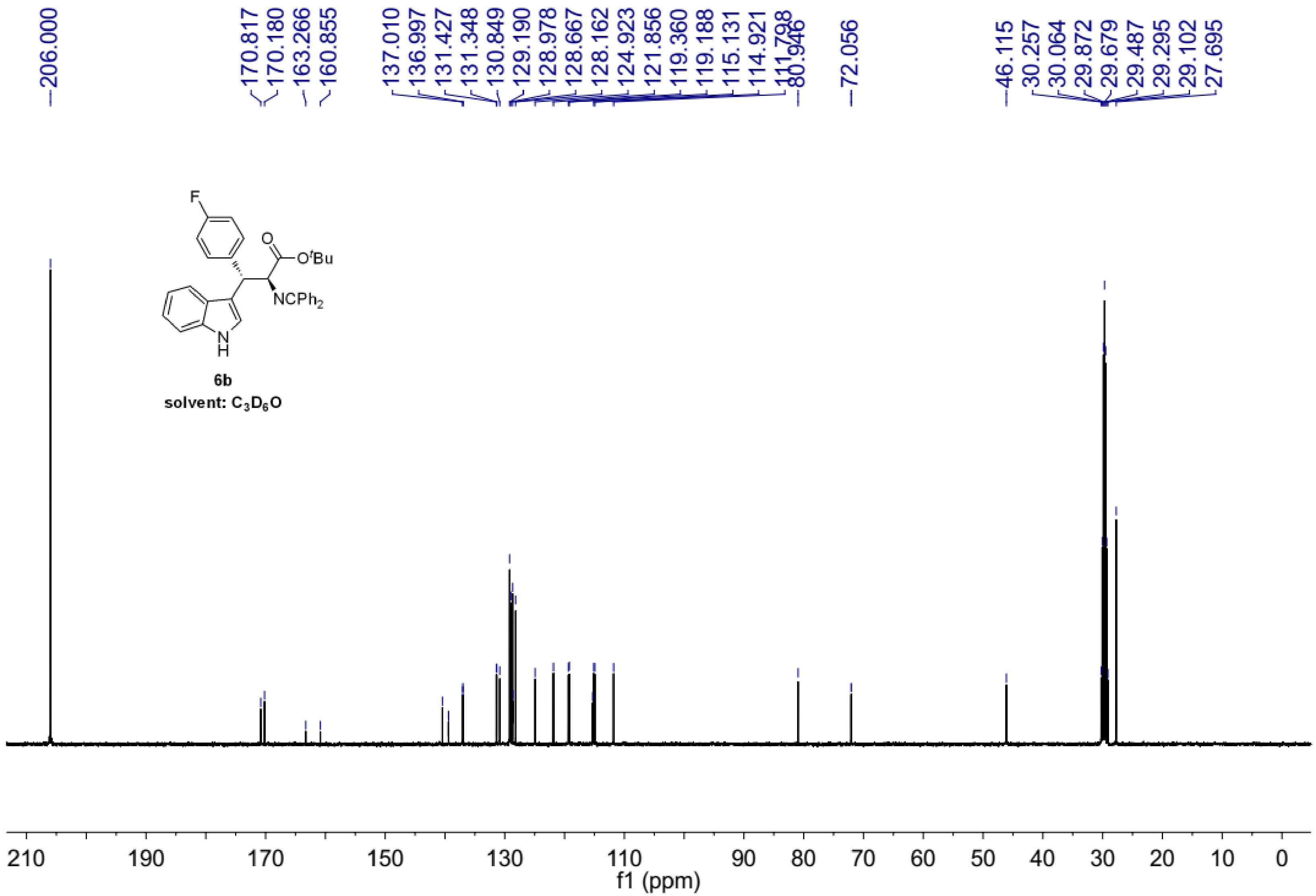

Supplementary Figure 239. ^{13}C NMR spectrum for compound **6b**

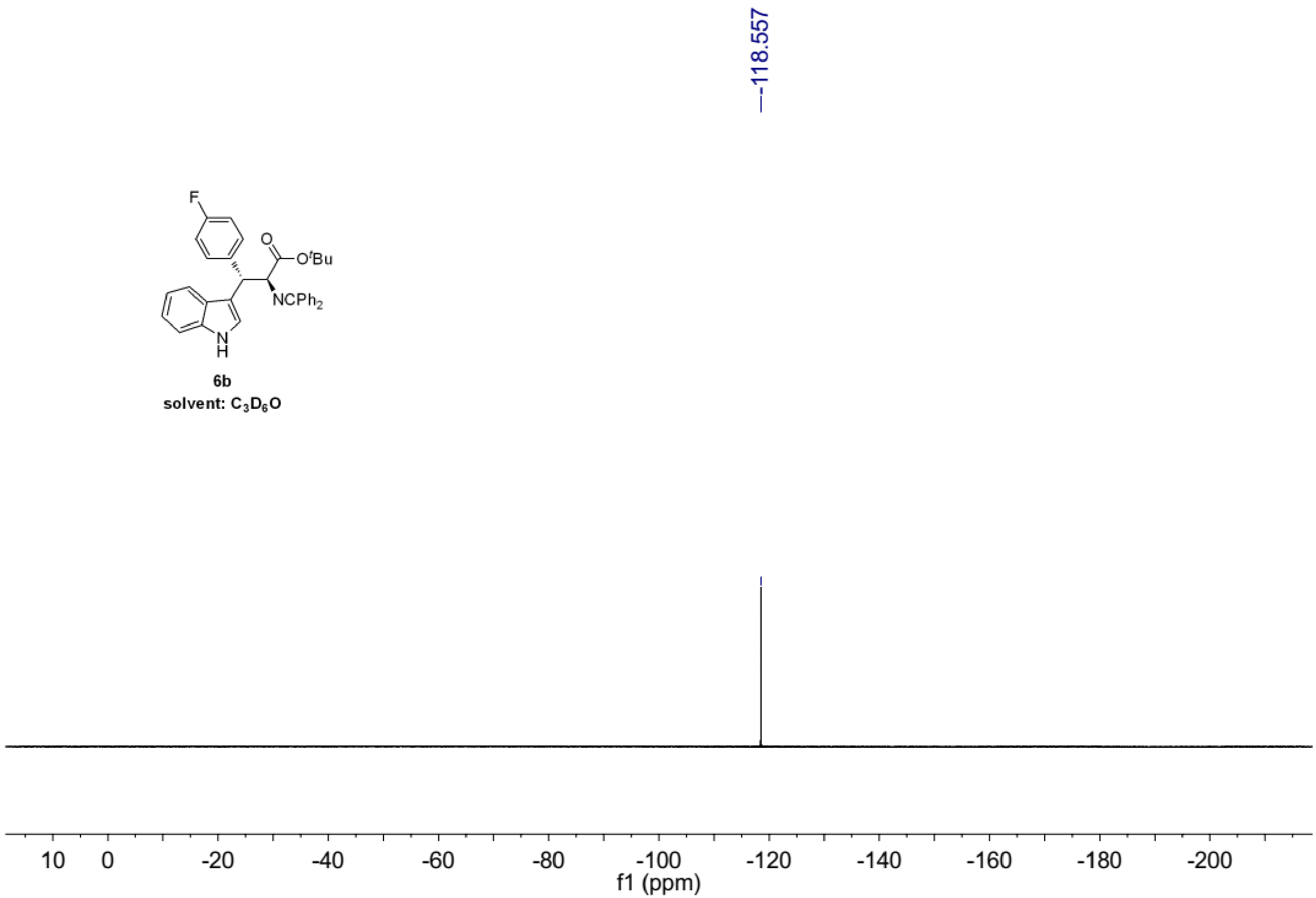

Supplementary Figure 240. ^{19}F NMR spectrum for compound **6b**

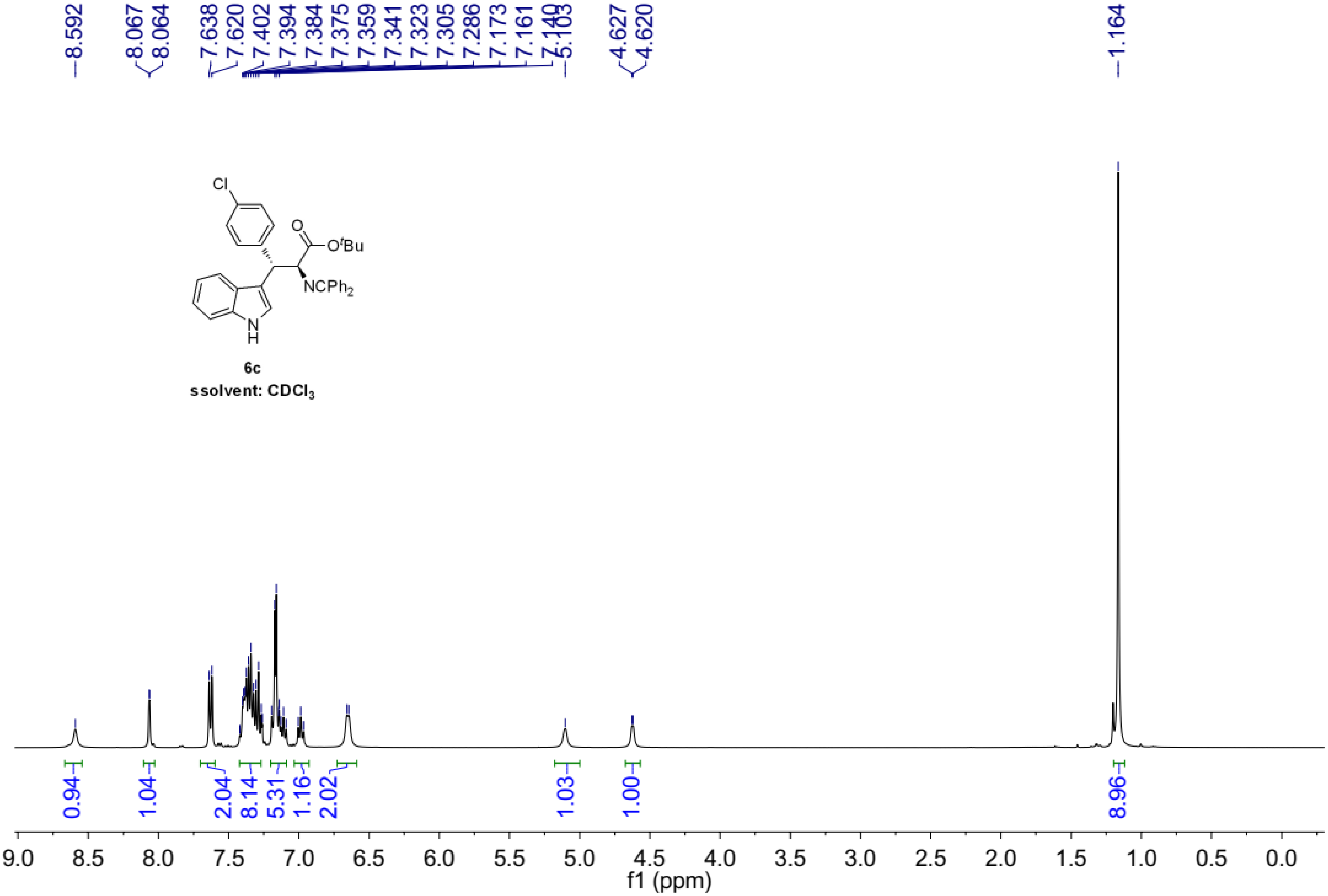

Supplementary Figure 241. ^{1}H NMR spectrum for compound **6c**

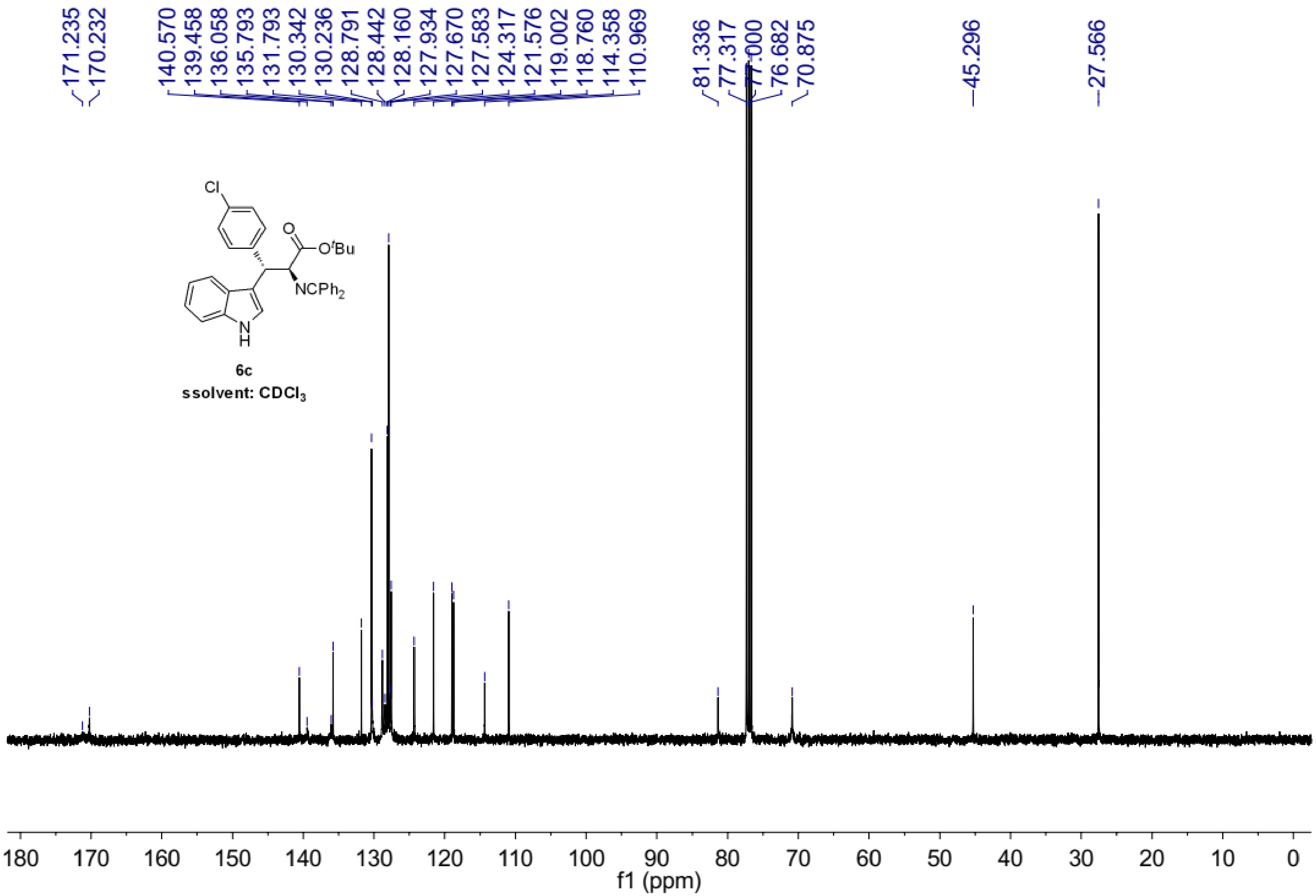

Supplementary Figure 242. ^{13}C NMR spectrum for compound **6c**

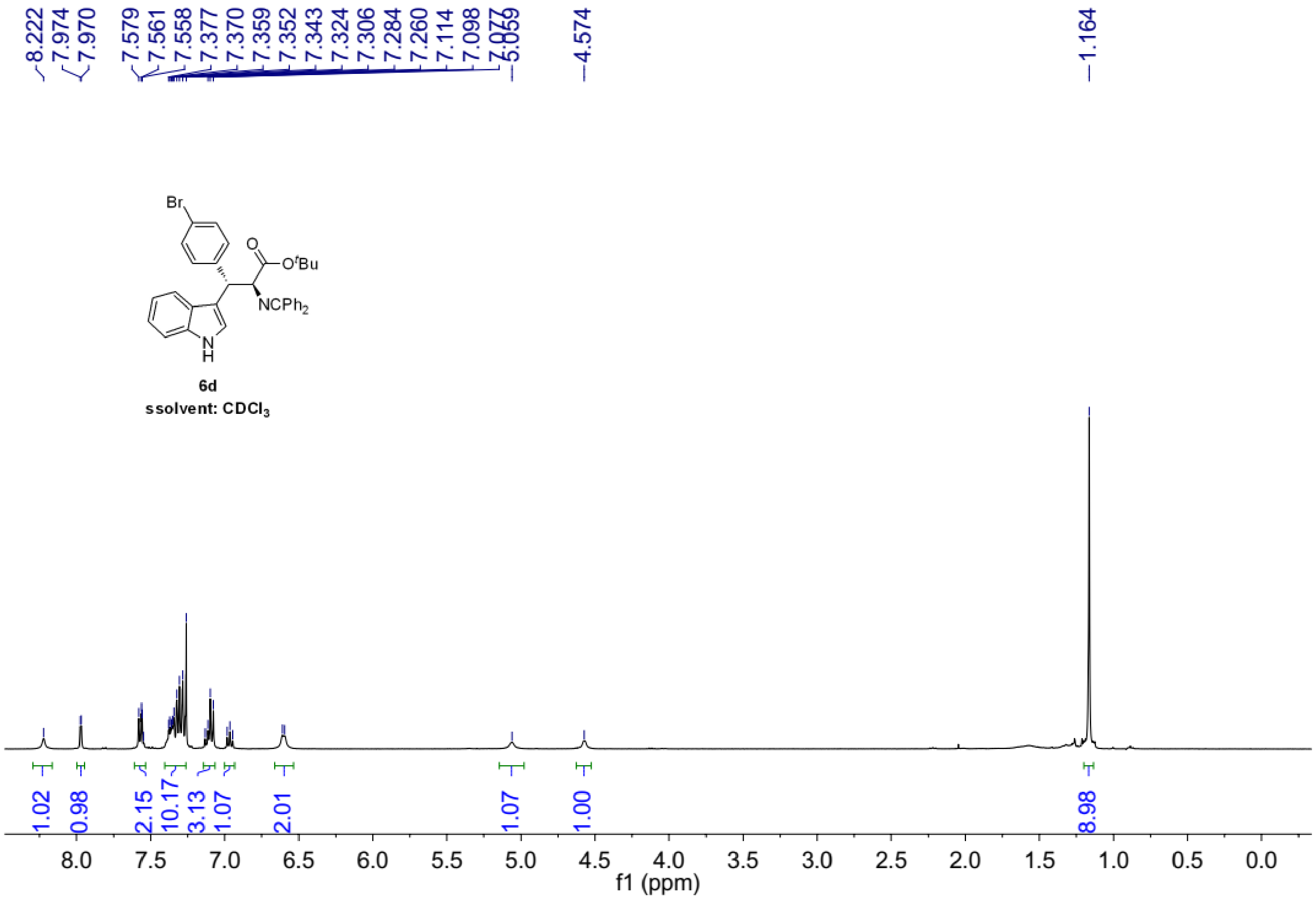

Supplementary Figure 243. ^{1}H NMR spectrum for compound **6d**

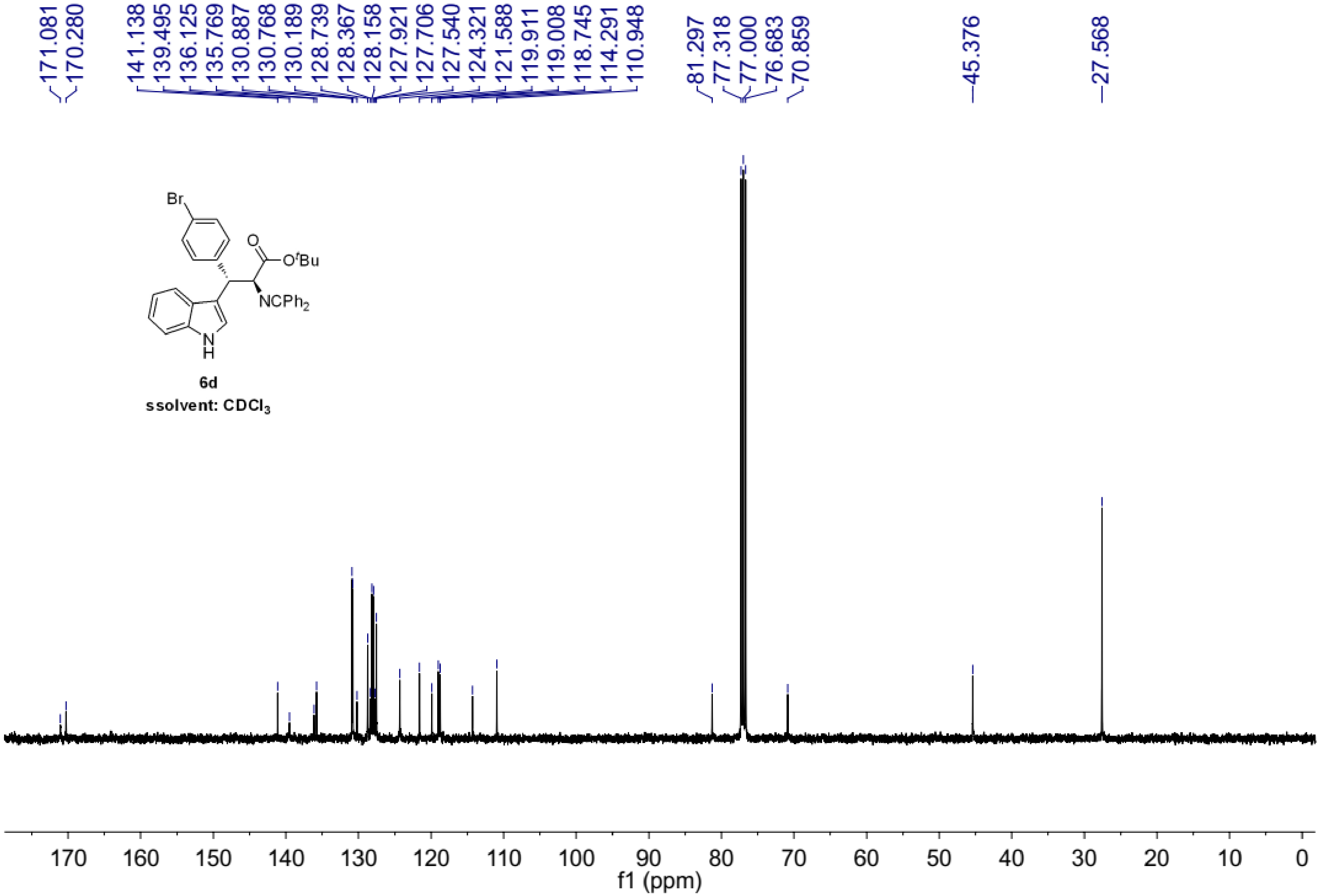

Supplementary Figure 244. ^{13}C NMR spectrum for compound **6d**

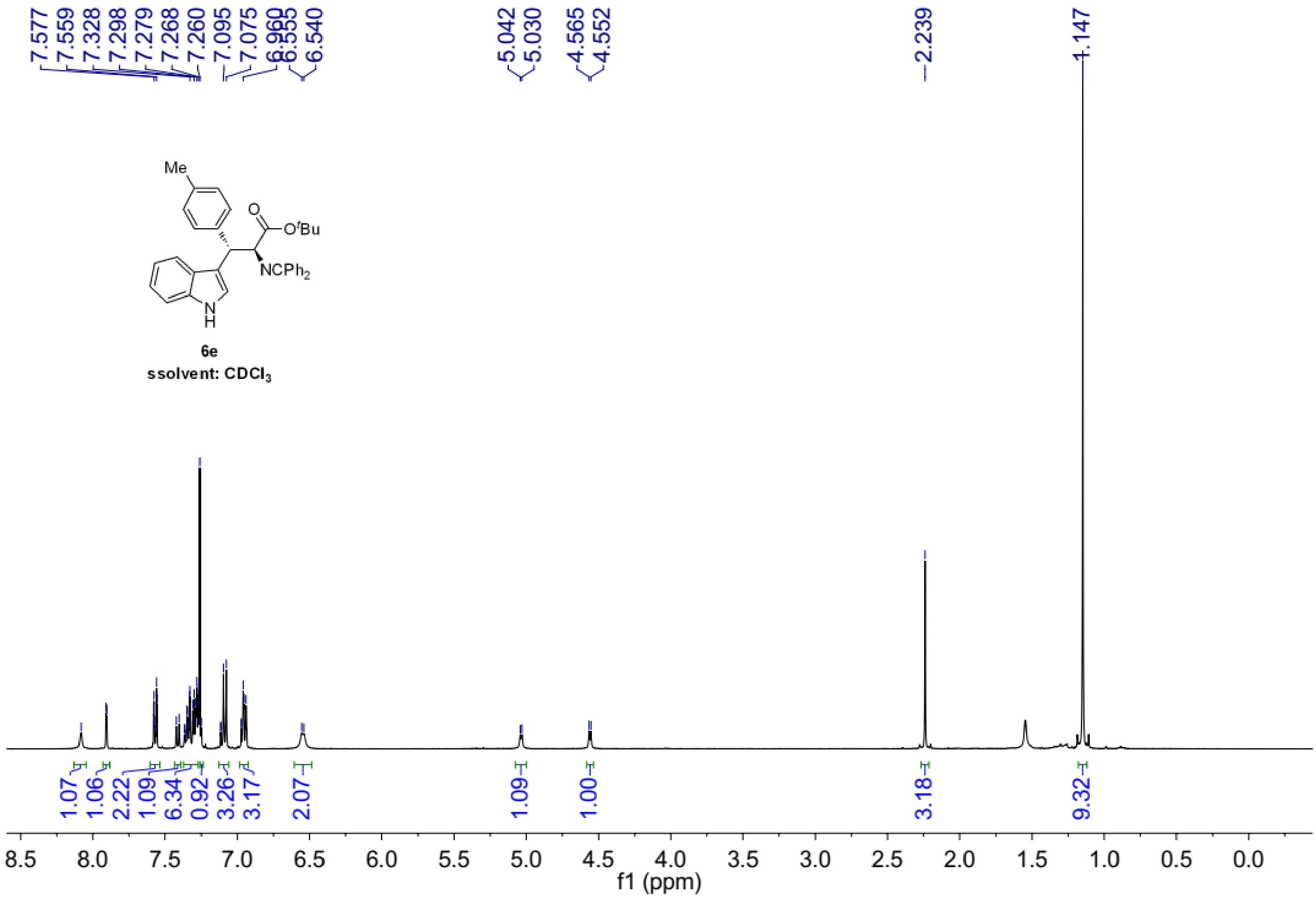

Supplementary Figure 245. ^{1}H NMR spectrum for compound **6e**

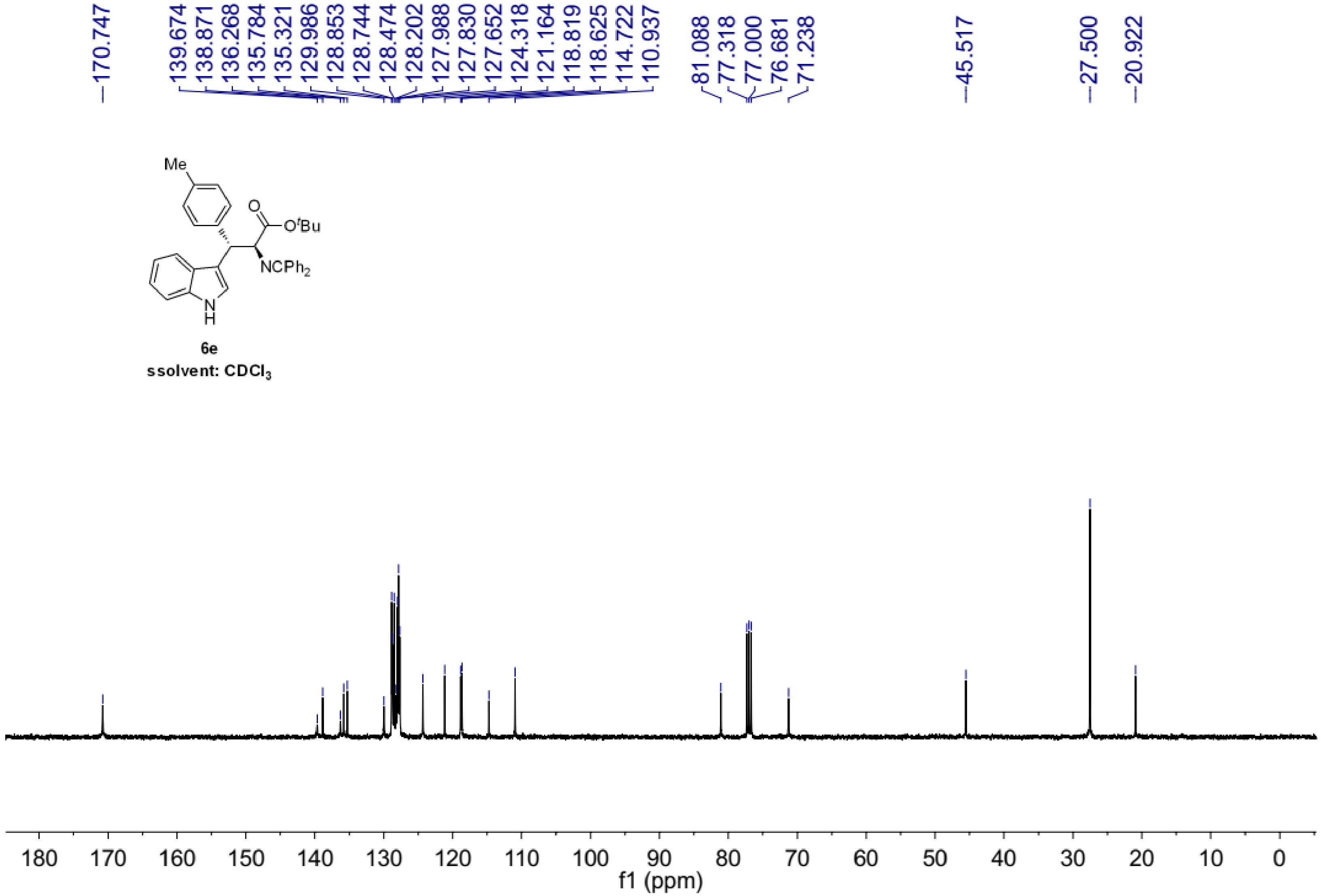

Supplementary Figure 246. ^{13}C NMR spectrum for compound **6e**

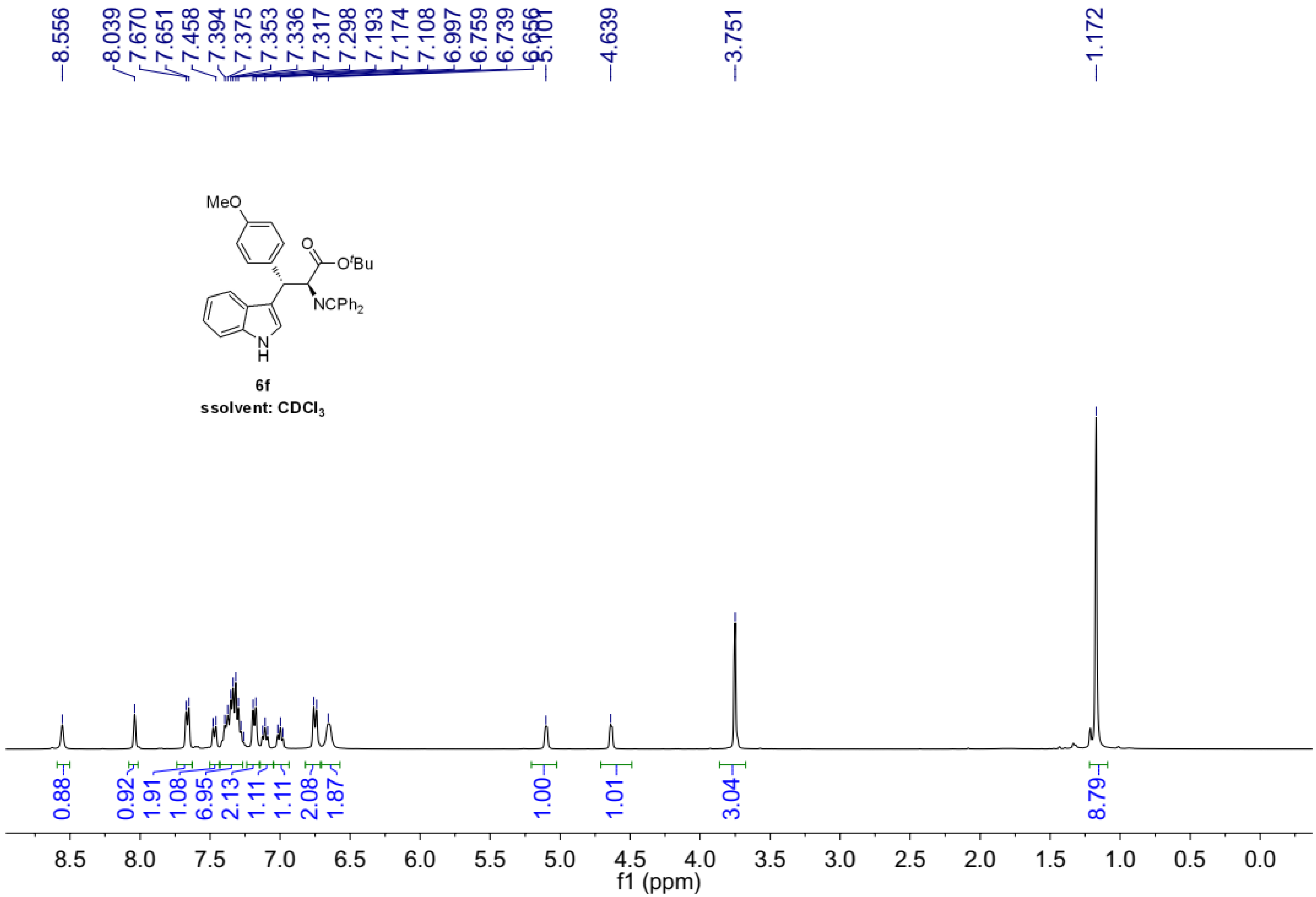

Supplementary Figure 247. ^{1}H NMR spectrum for compound **6f**

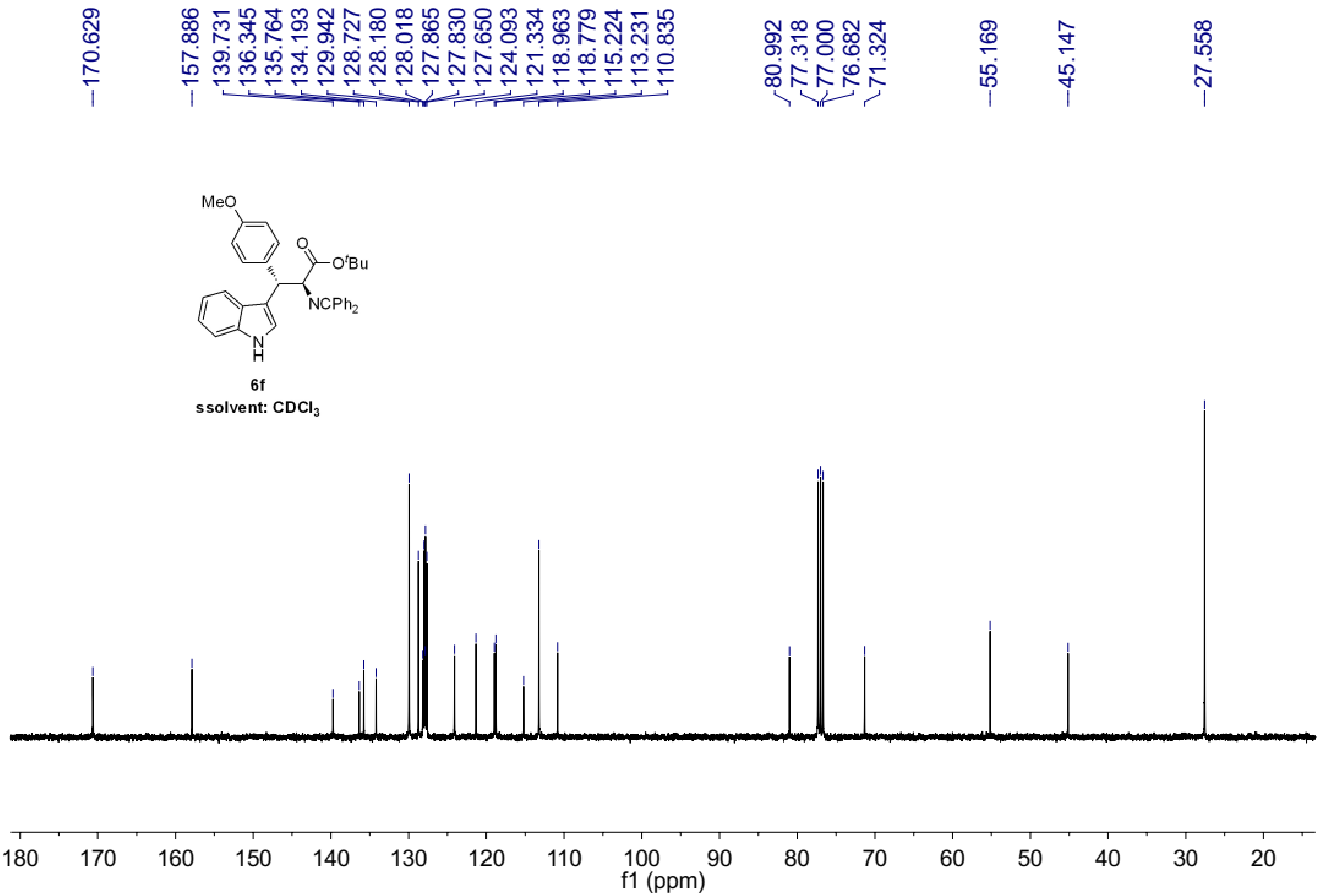

Supplementary Figure 248. ^{13}C NMR spectrum for compound **6f**

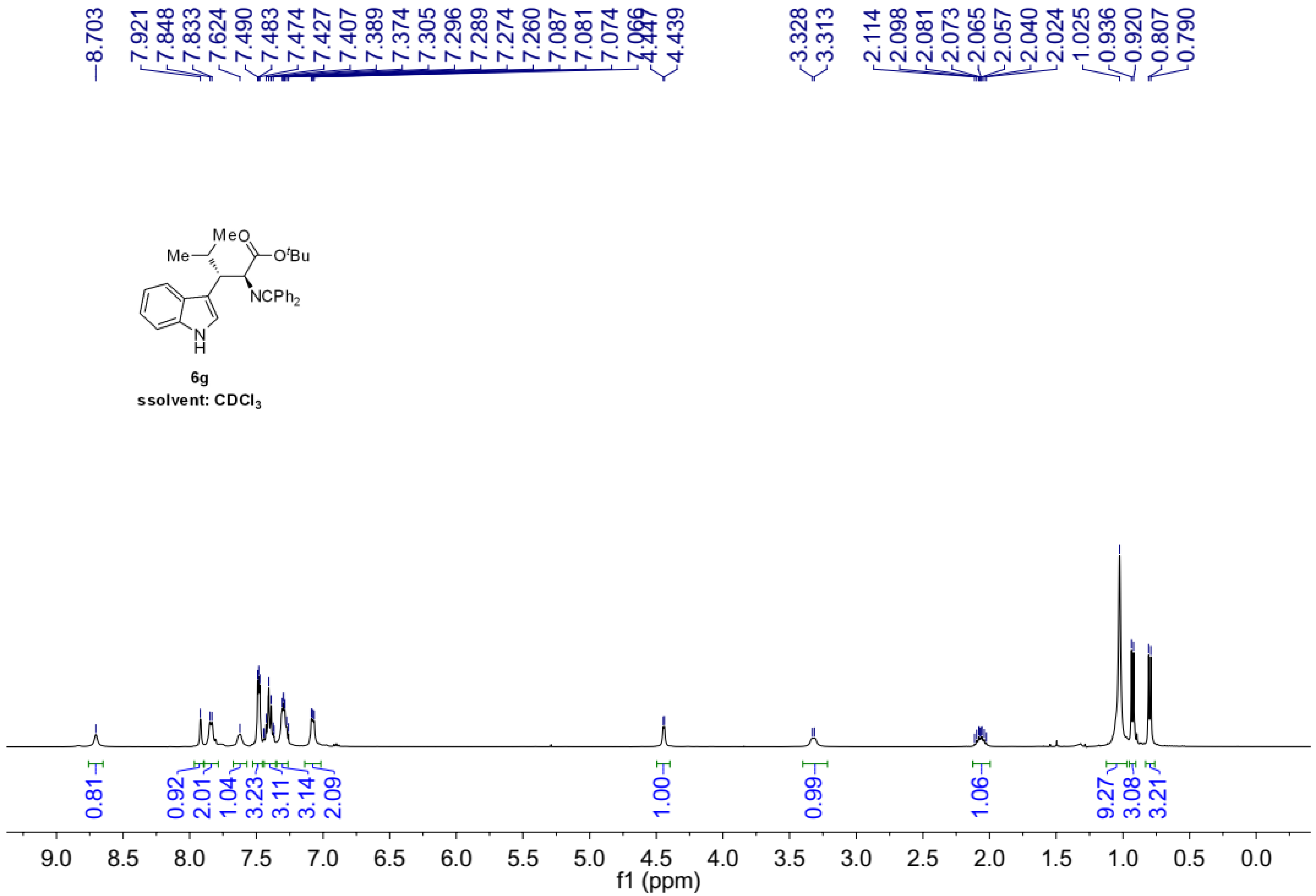

Supplementary Figure 249. ^{1}H NMR spectrum for compound **6g**

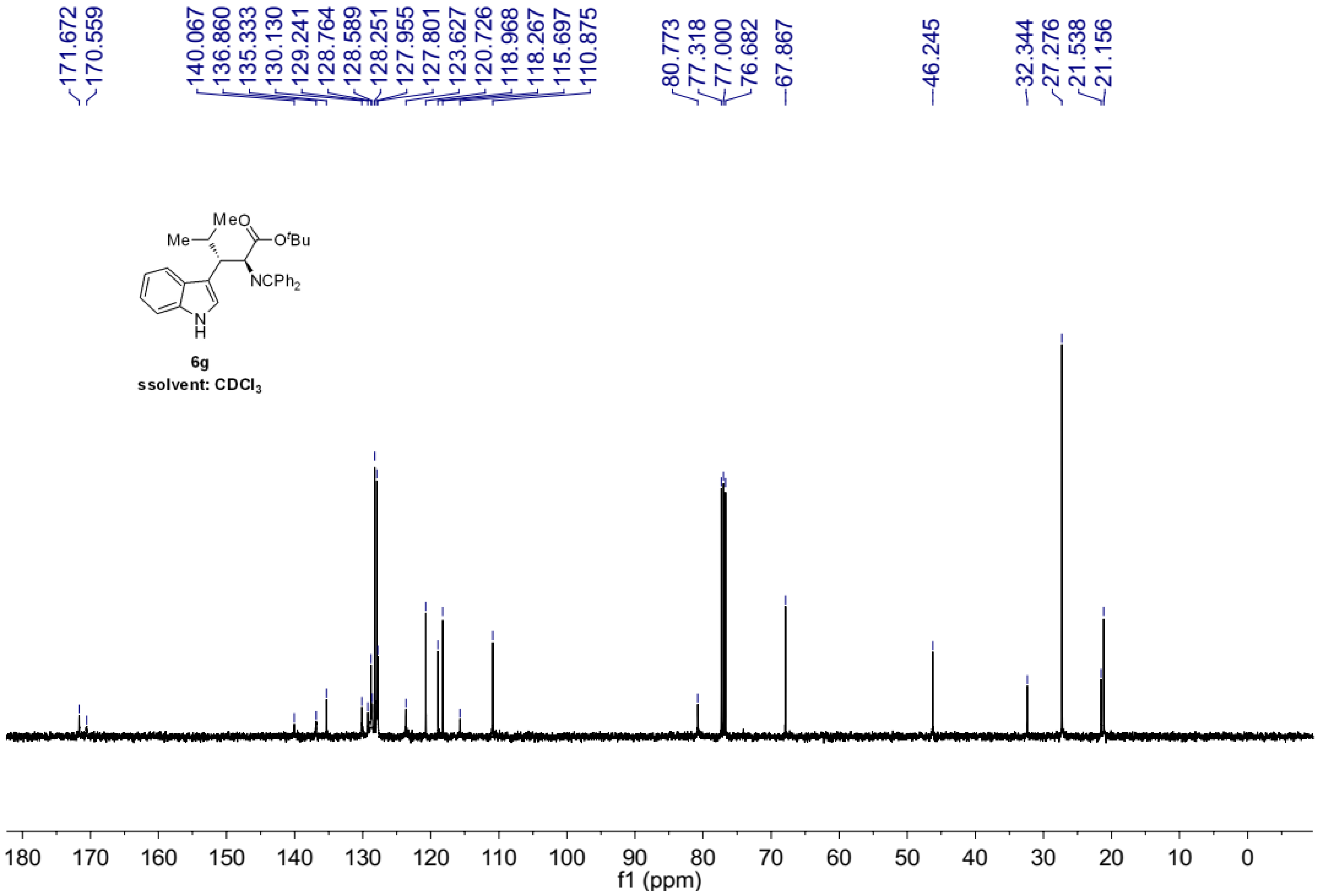

Supplementary Figure 250. ^{13}C NMR spectrum for compound **6g**

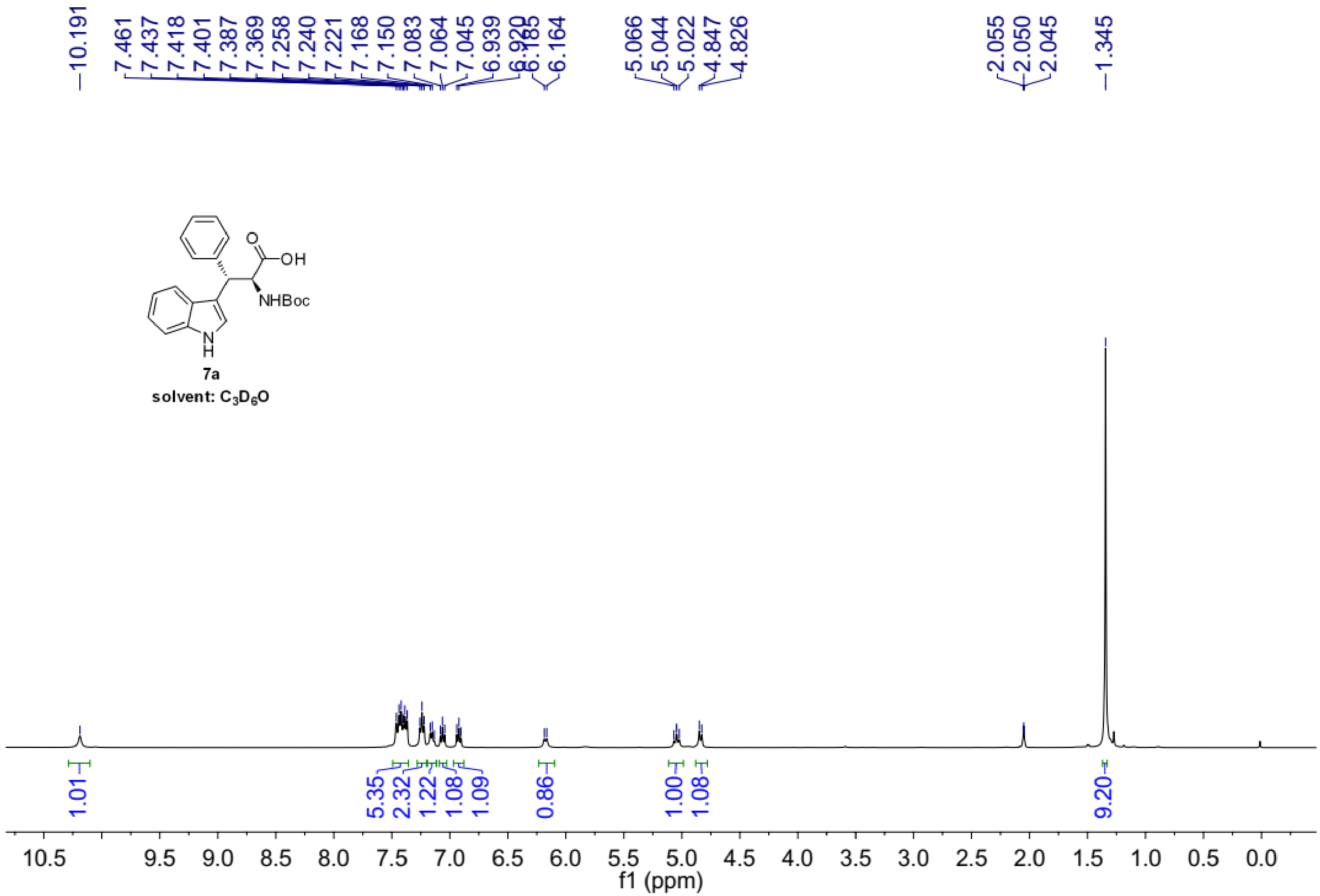

Supplementary Figure 251. ^{1}H NMR spectrum for compound **7a**

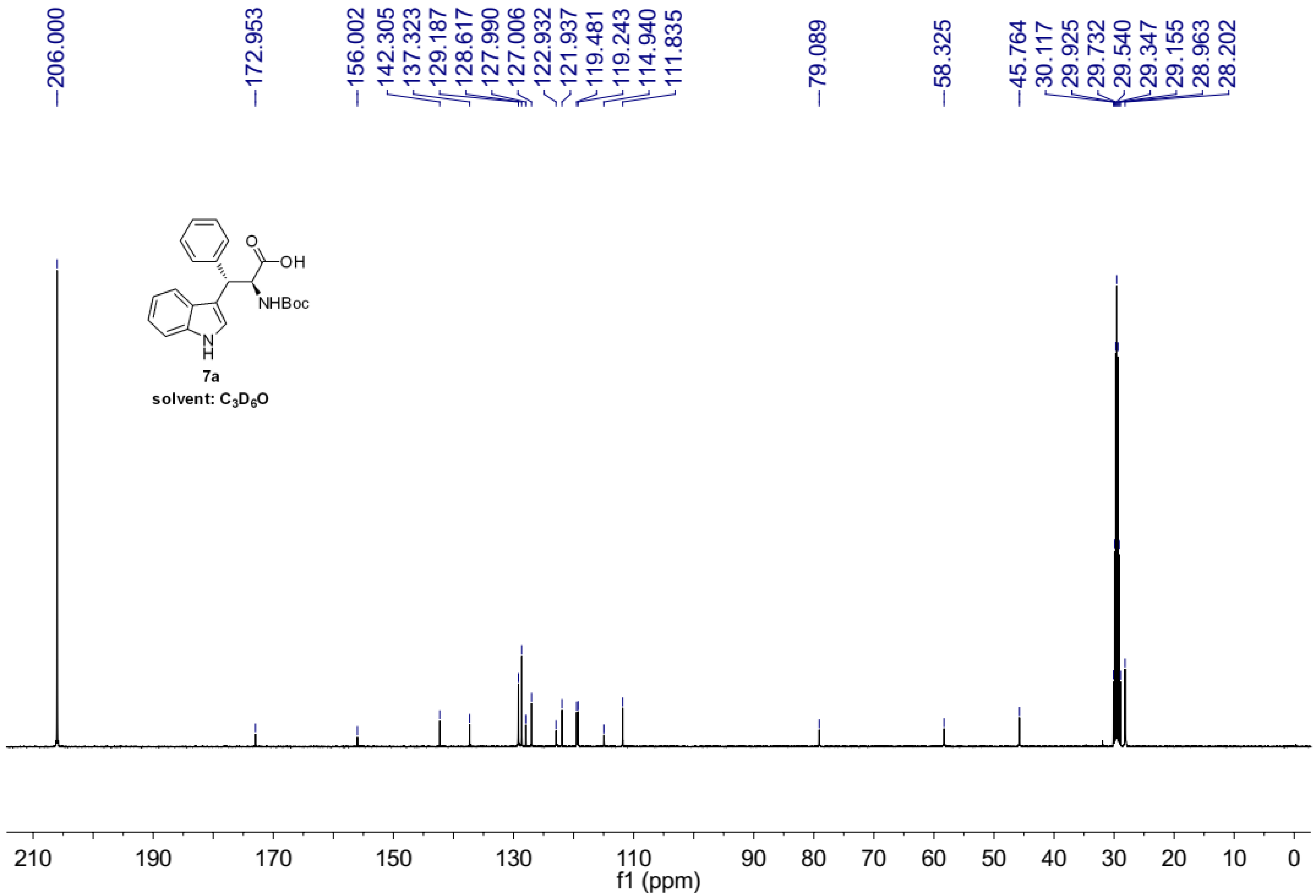

Supplementary Figure 252. ^{13}C NMR spectrum for compound **7a**

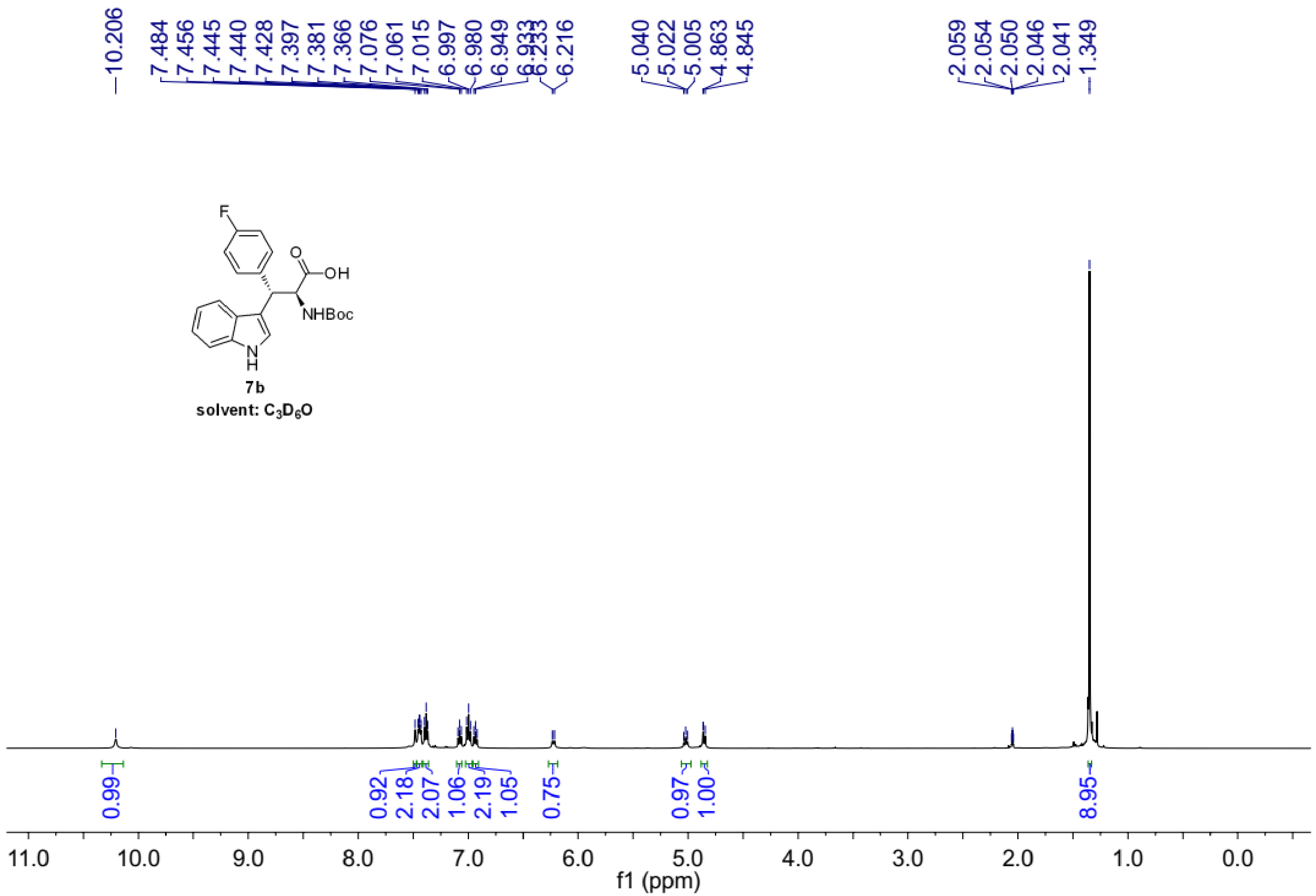

Supplementary Figure 253. ^{1}H NMR spectrum for compound **7b**

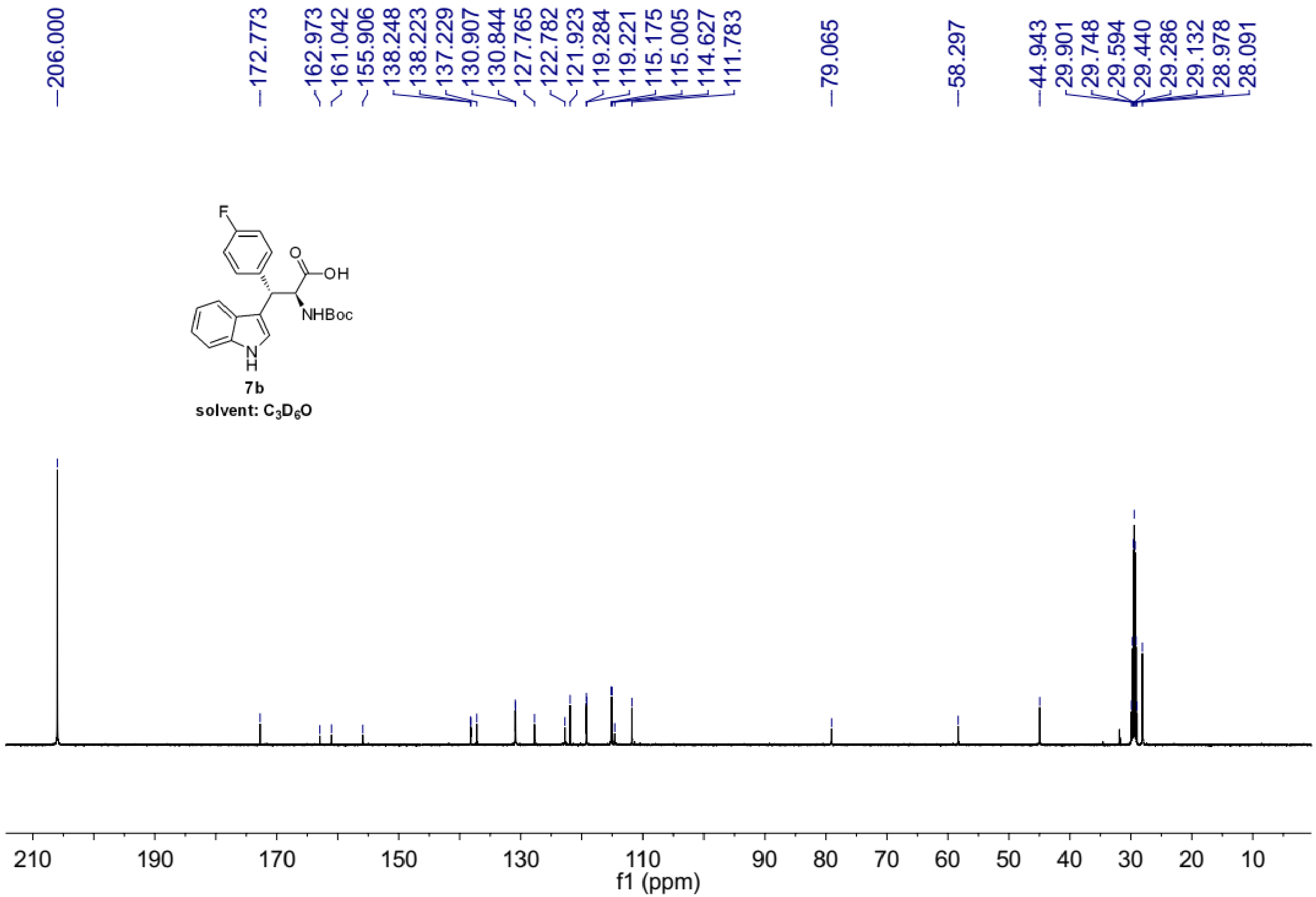

Supplementary Figure 254. ^{13}C NMR spectrum for compound **7b**

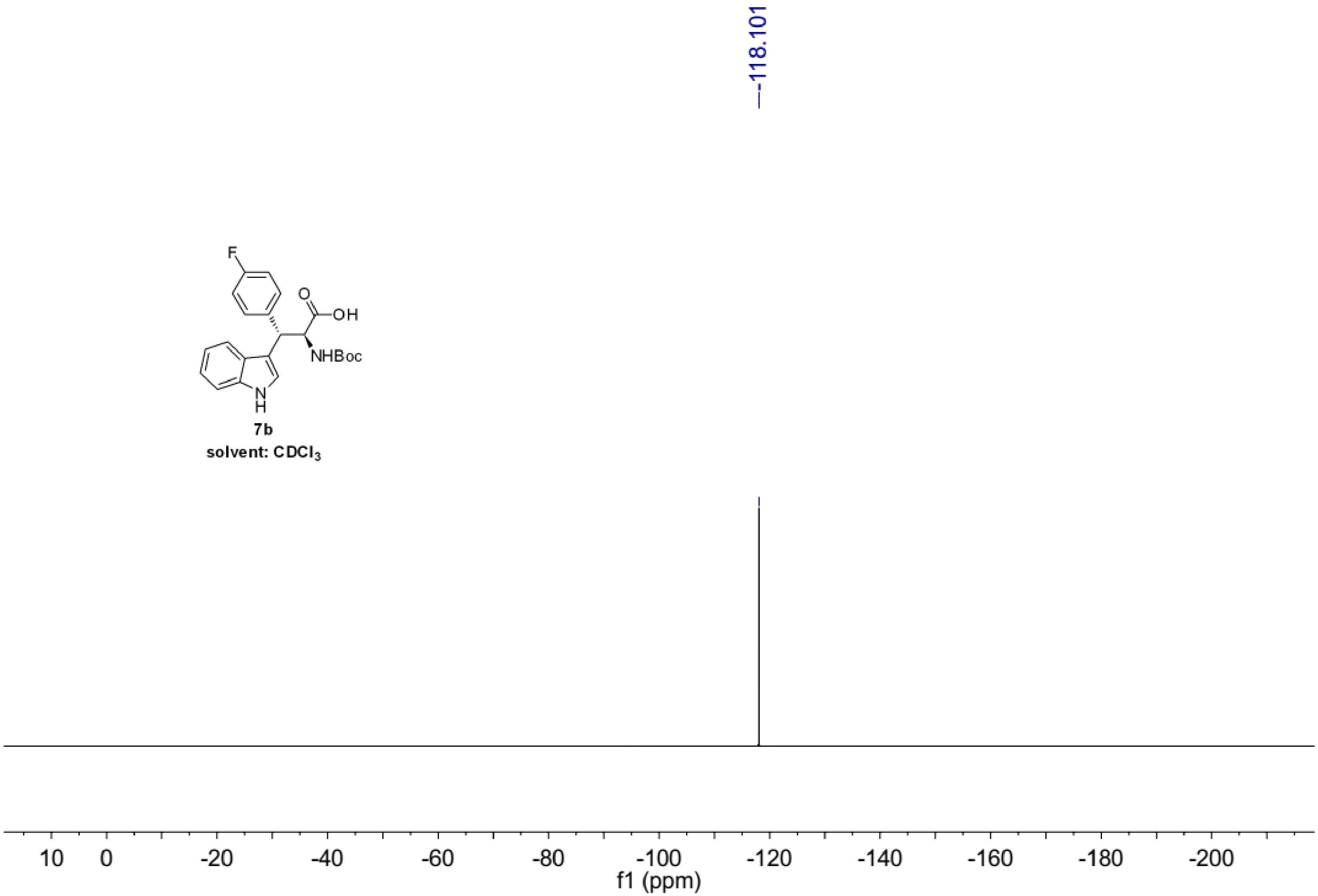

Supplementary Figure 255. ^{19}F NMR spectrum for compound **7b**

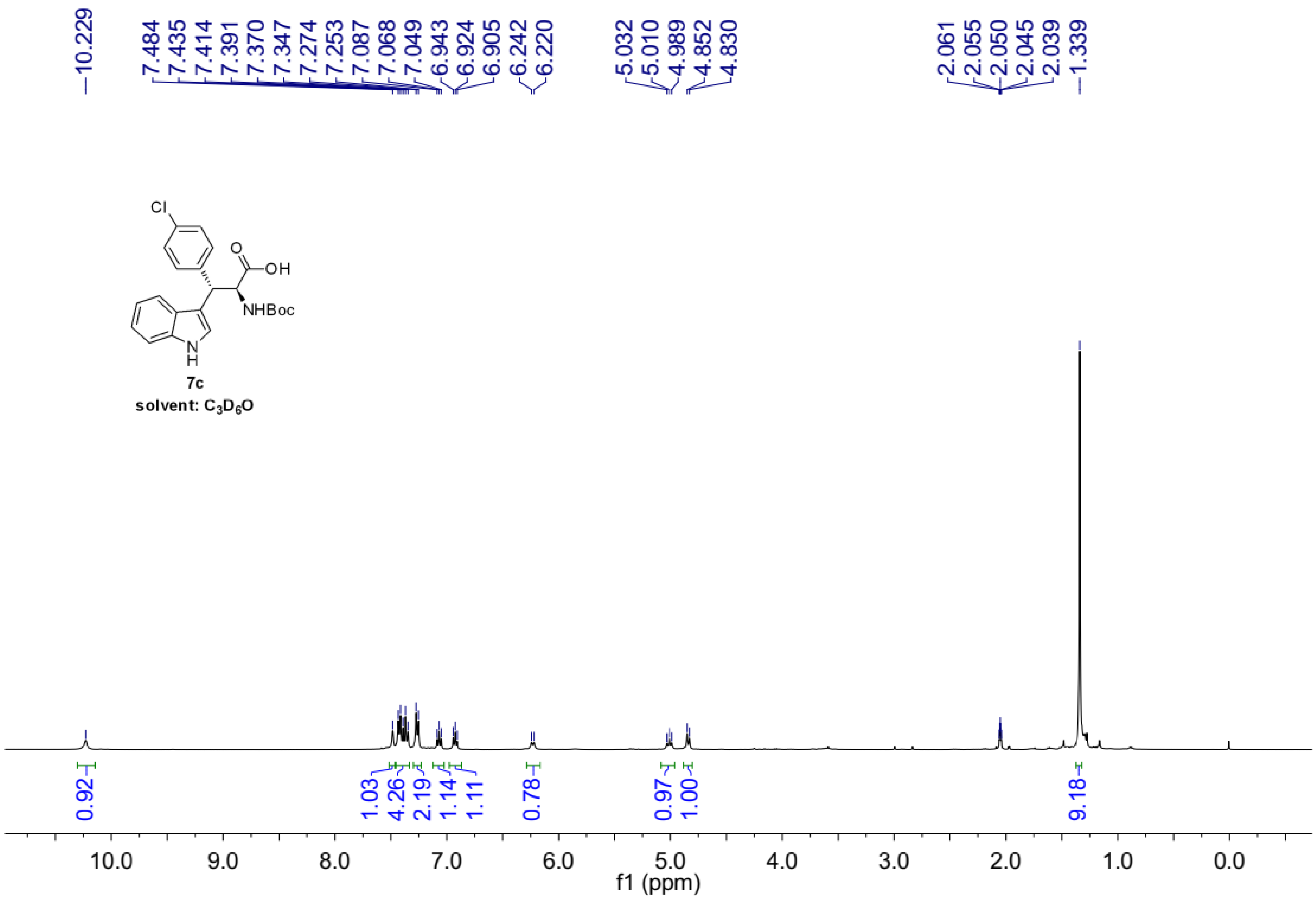

Supplementary Figure 256. ^{1}H NMR spectrum for compound **7c**

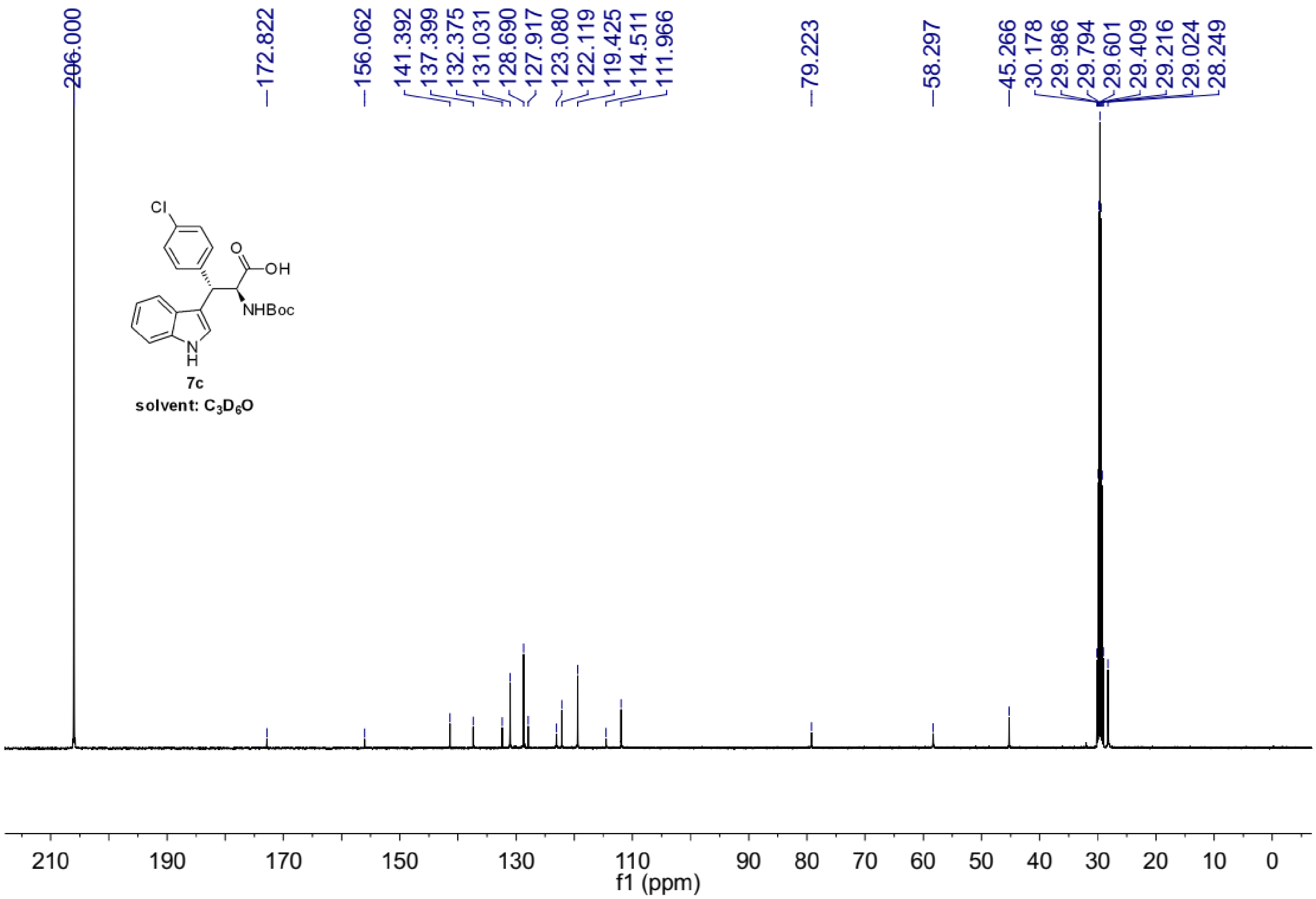

Supplementary Figure 257. ^{13}C NMR spectrum for compound **7c**

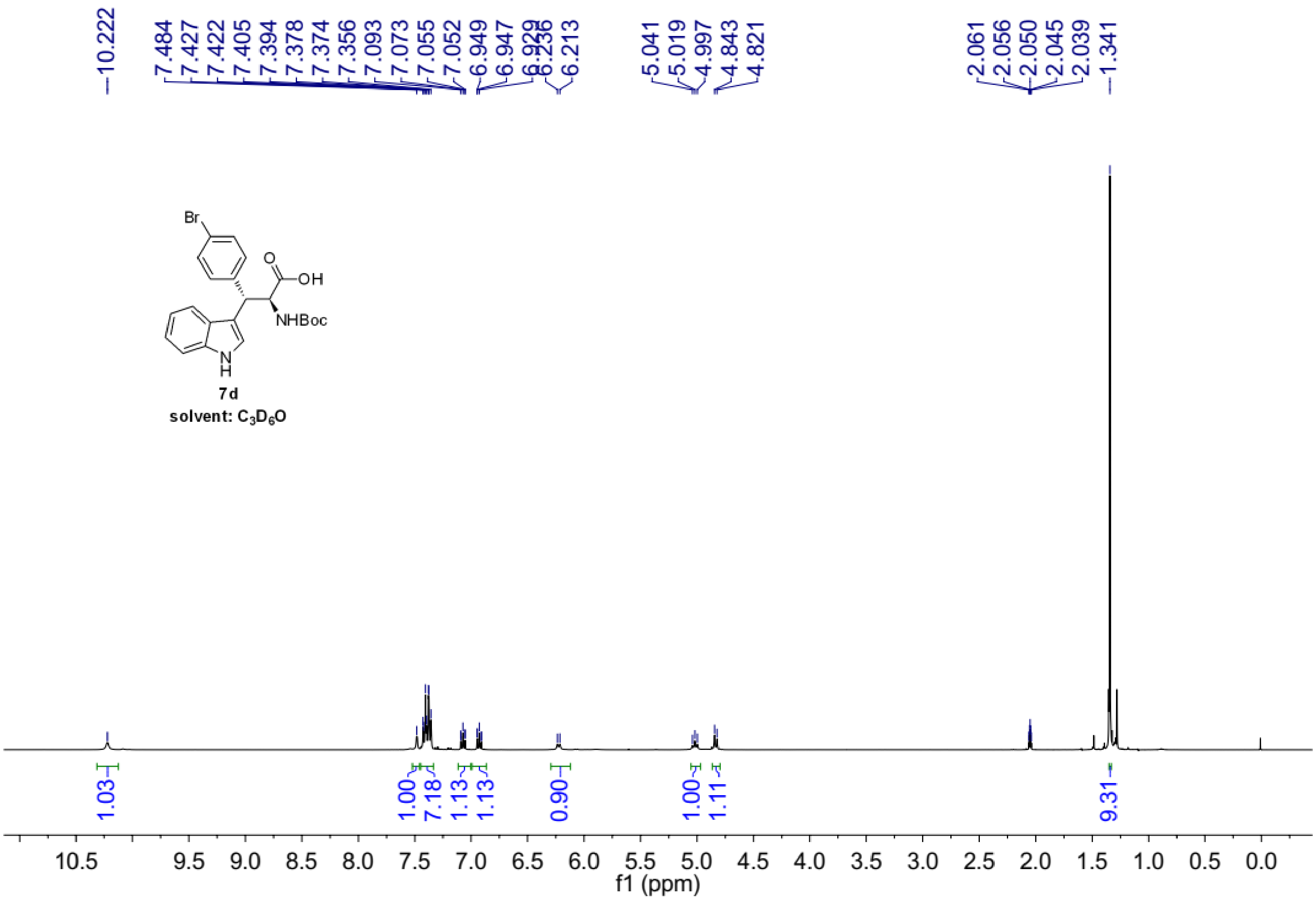

Supplementary Figure 258. ^{1}H NMR spectrum for compound **7d**

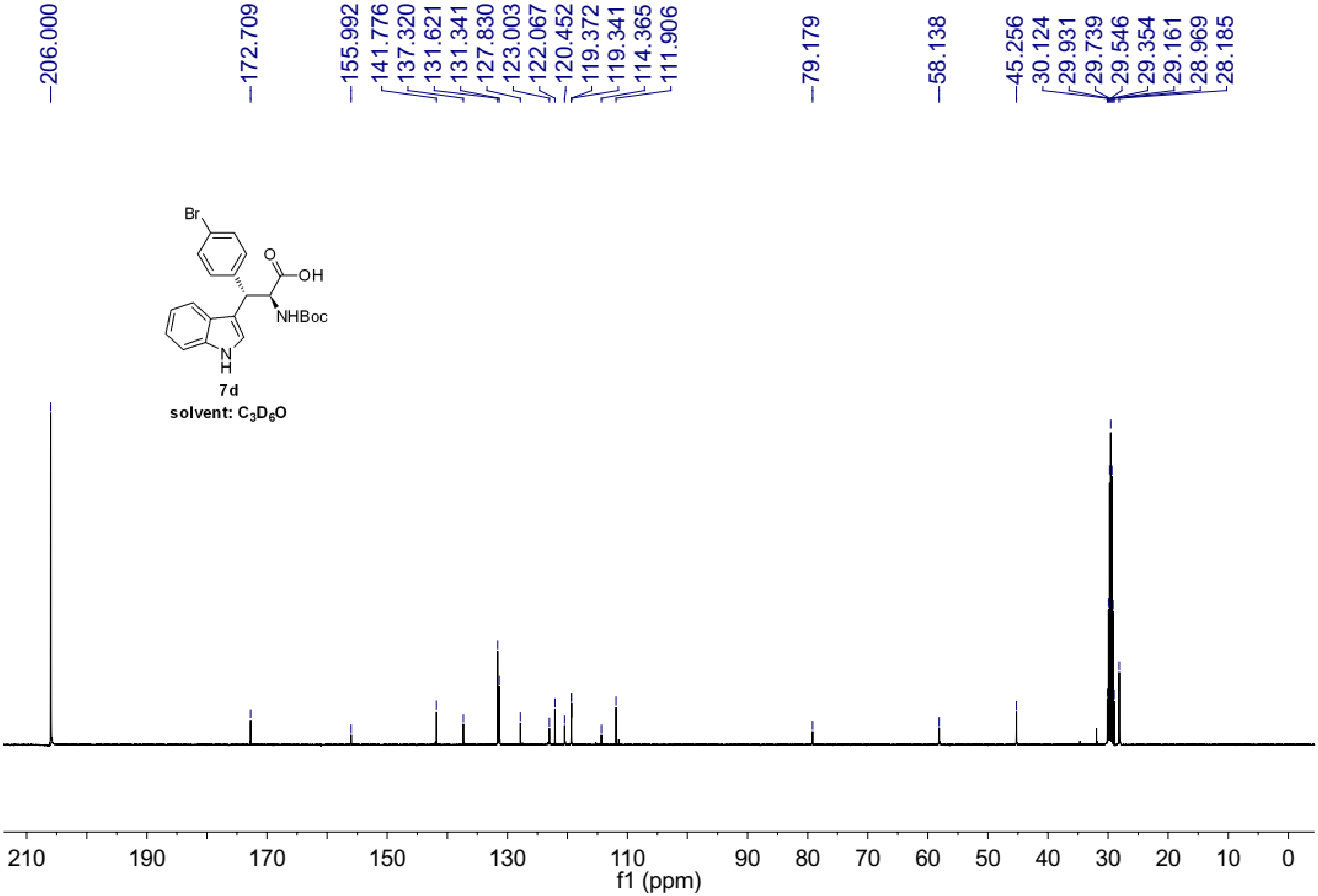

Supplementary Figure 259. ^{13}C NMR spectrum for compound **7d**

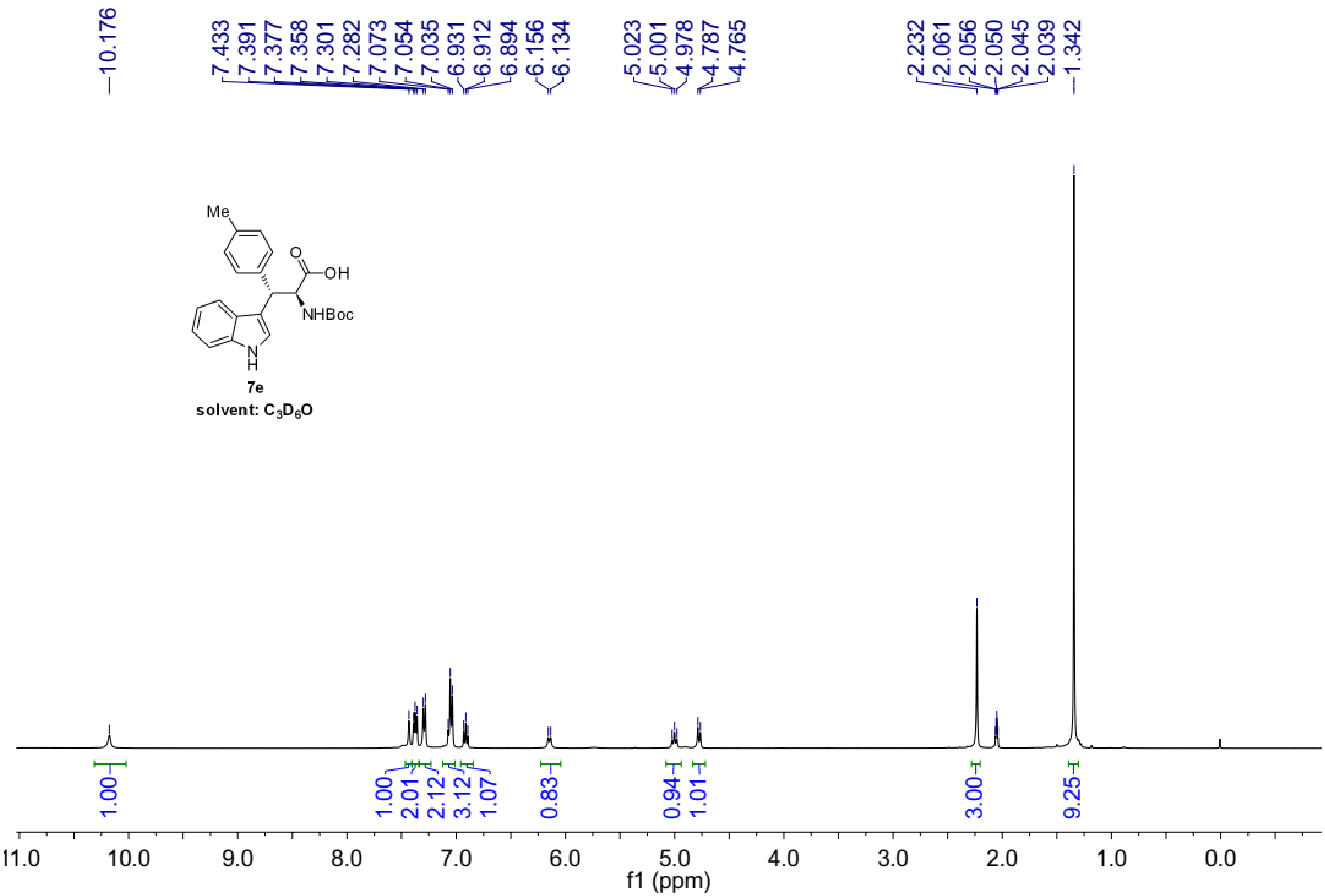

Supplementary Figure 260. ^{1}H NMR spectrum for compound **7e**

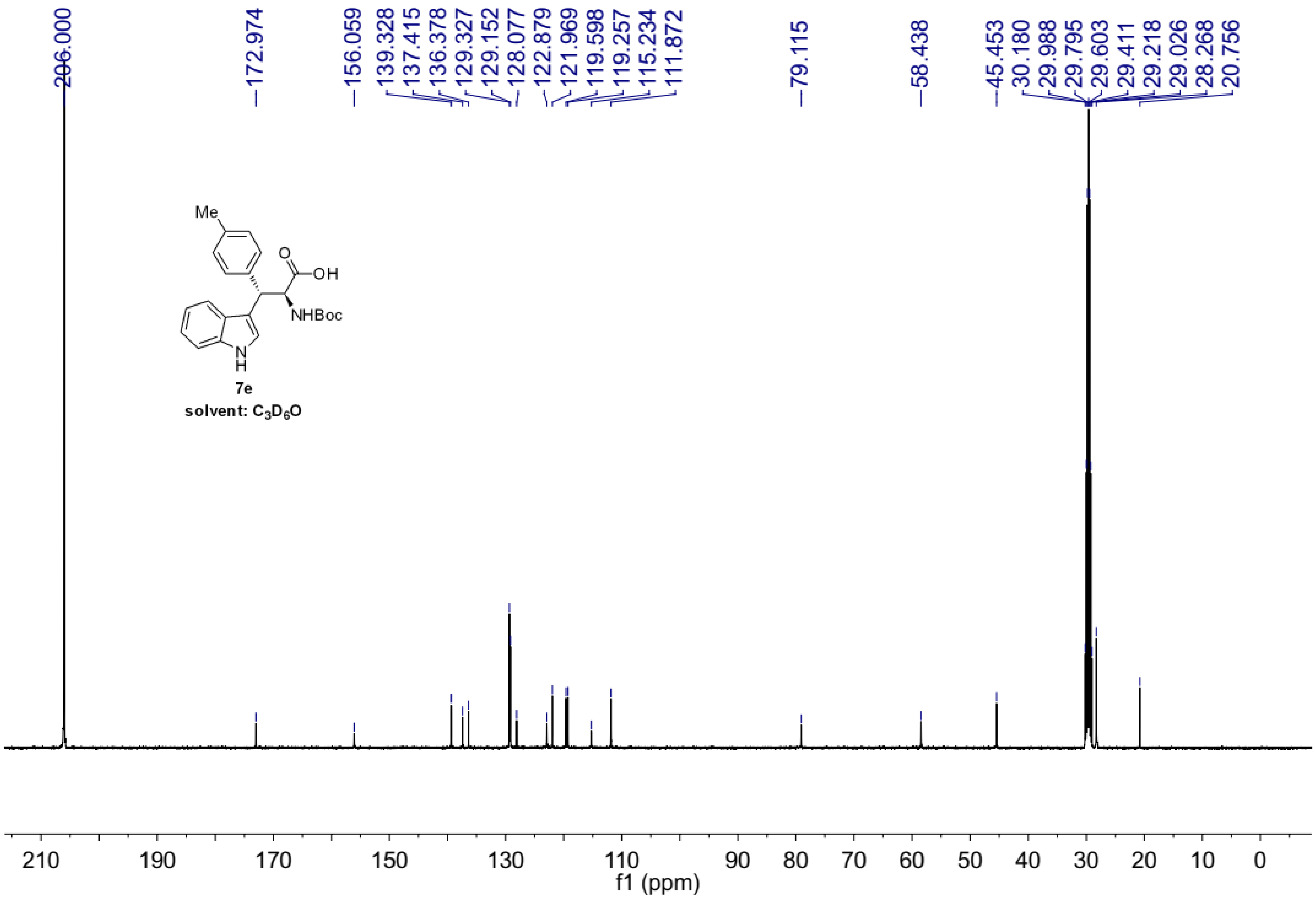

Supplementary Figure 261. ^{13}C NMR spectrum for compound **7e**

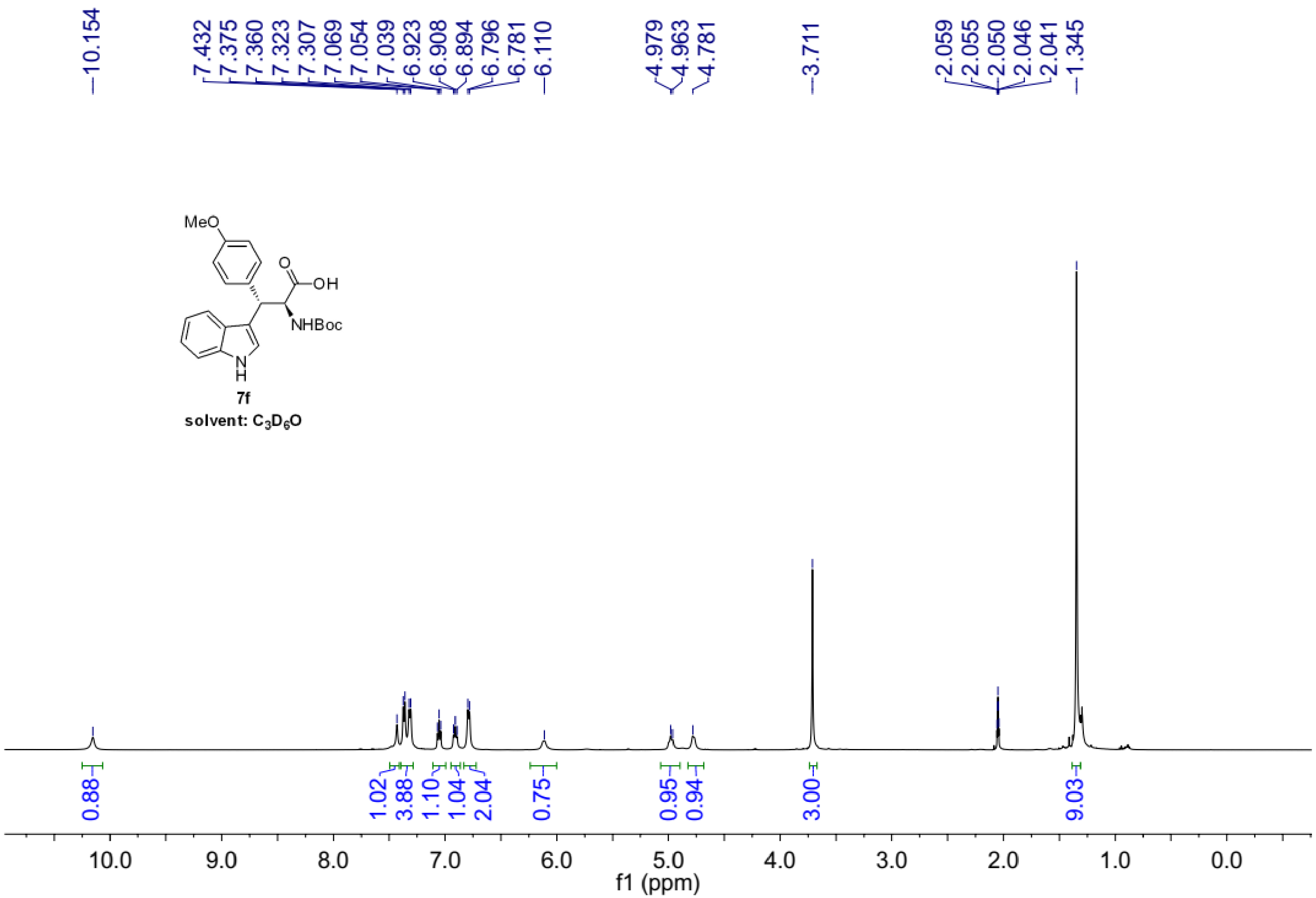

Supplementary Figure 262. ^{1}H NMR spectrum for compound **7f**

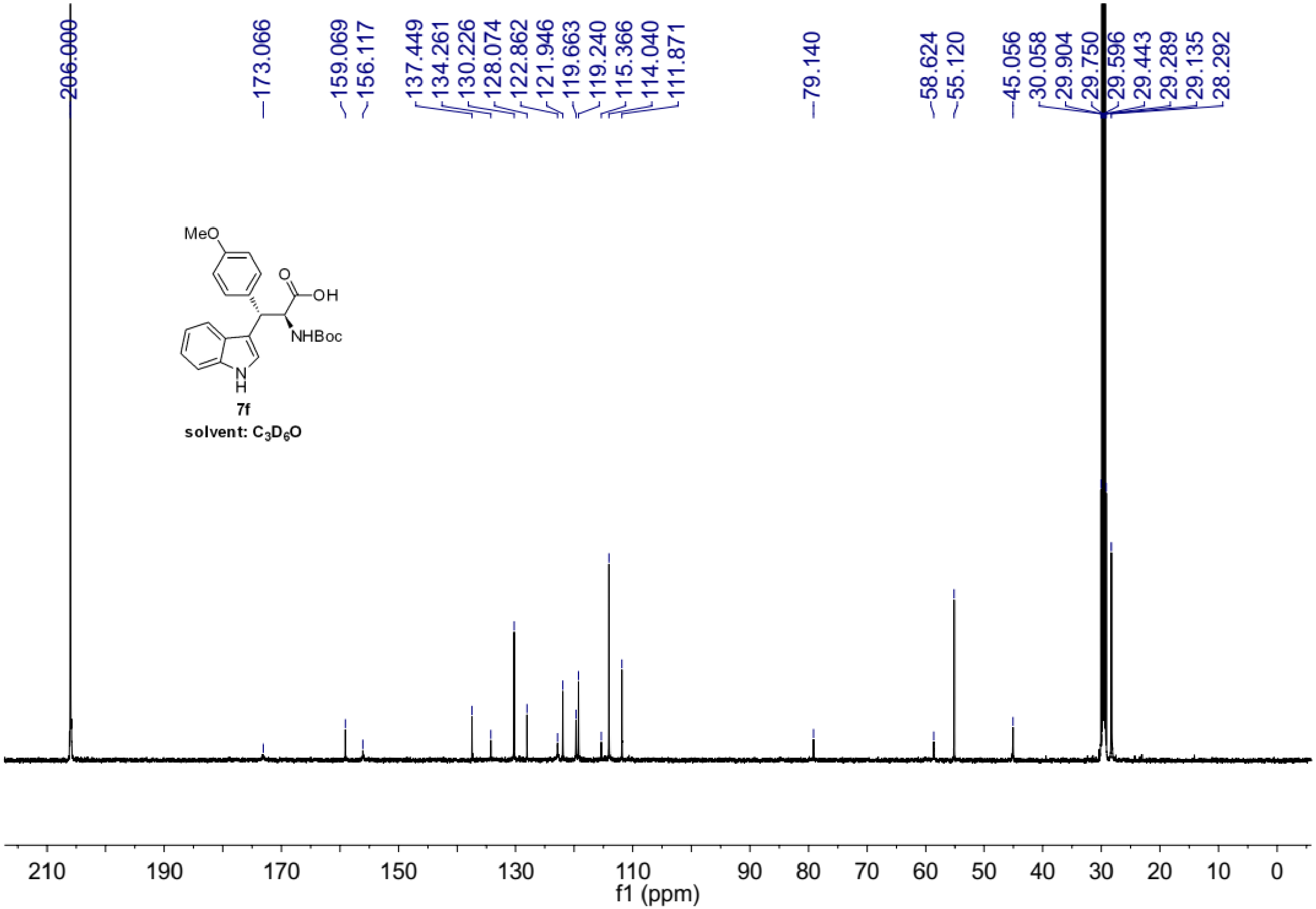

Supplementary Figure 263. ^{13}C NMR spectrum for compound **7f**

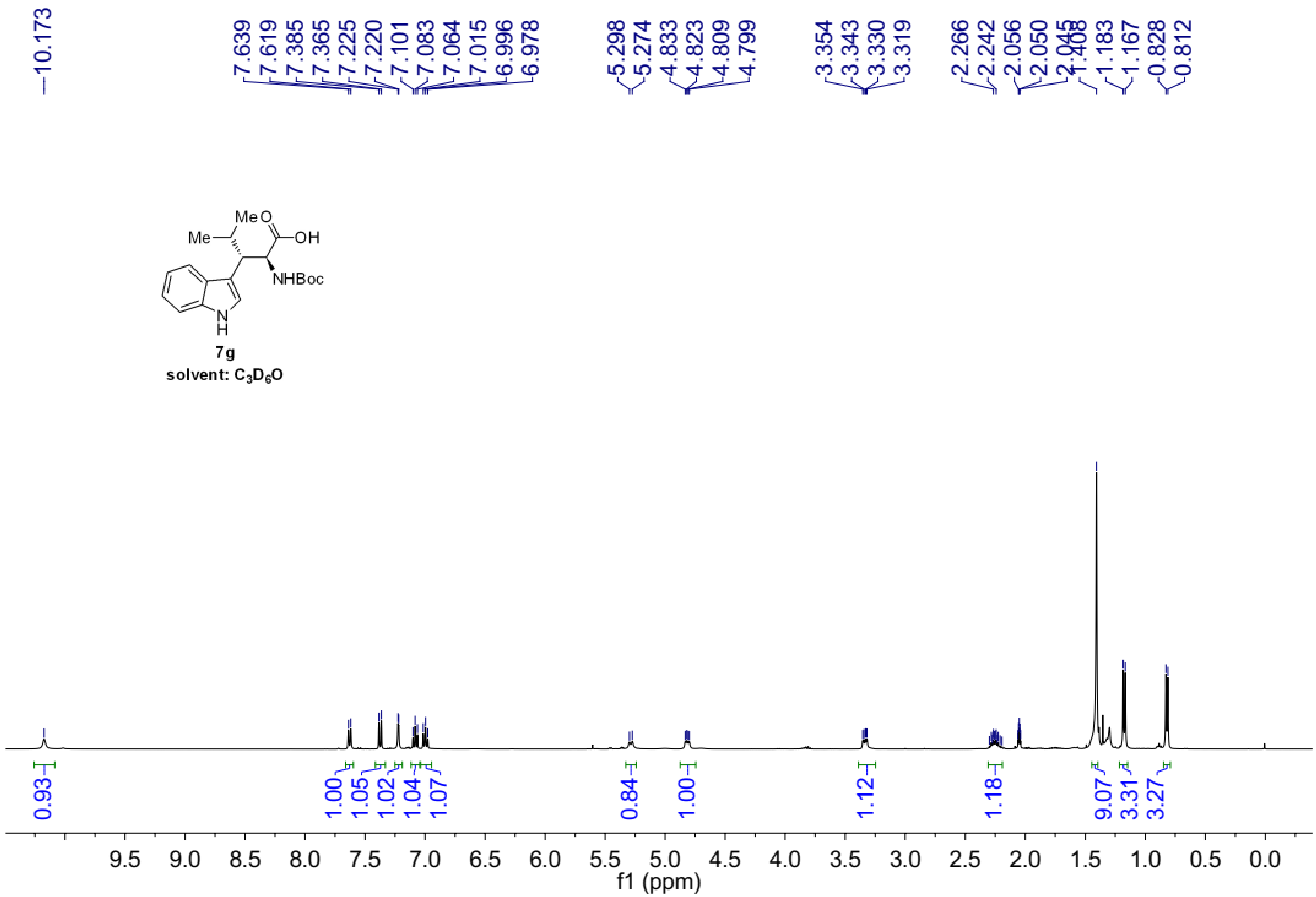

Supplementary Figure 264. ^{1}H NMR spectrum for compound **7g**

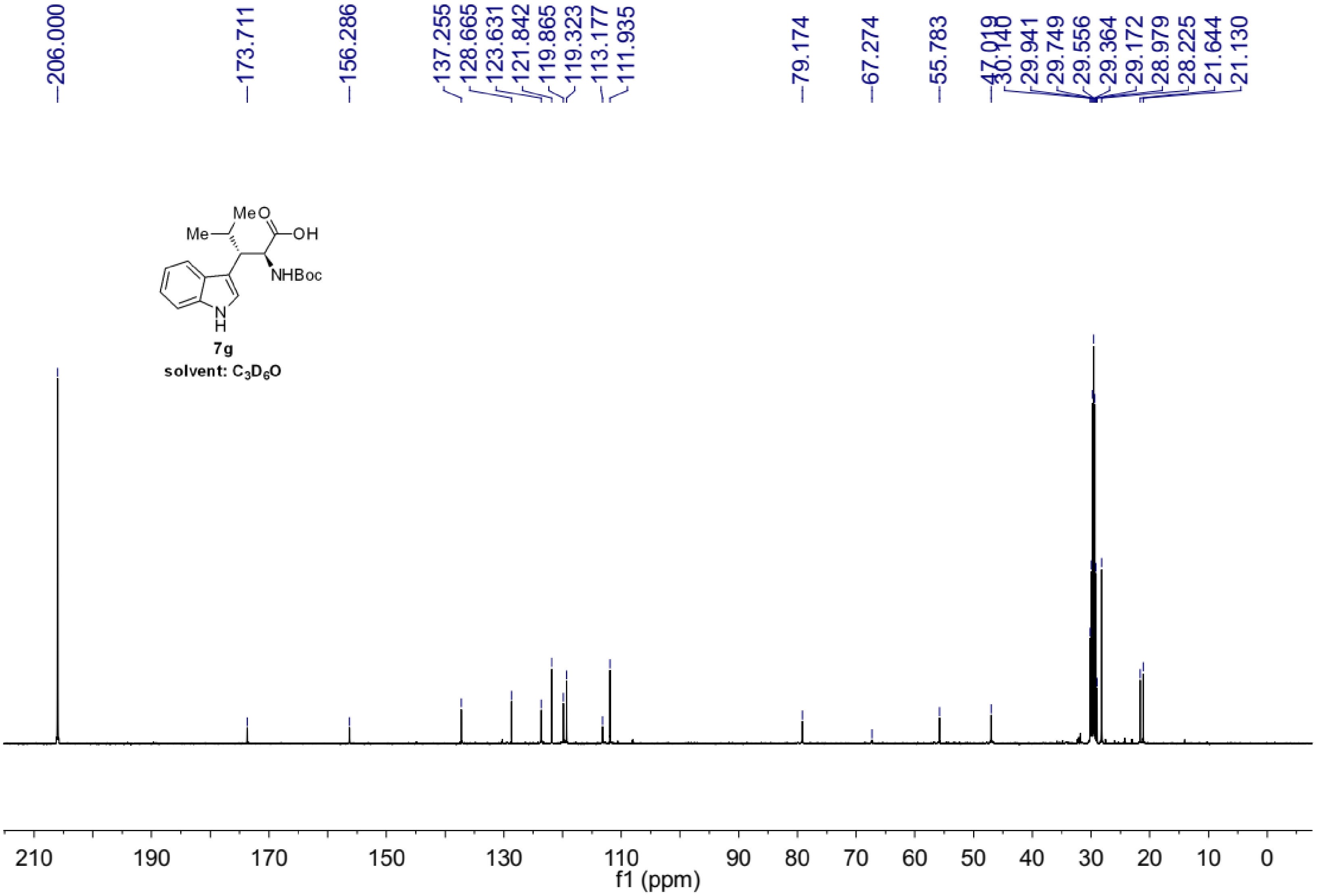

Supplementary Figure 265. ^{13}C NMR spectrum for compound **7g**

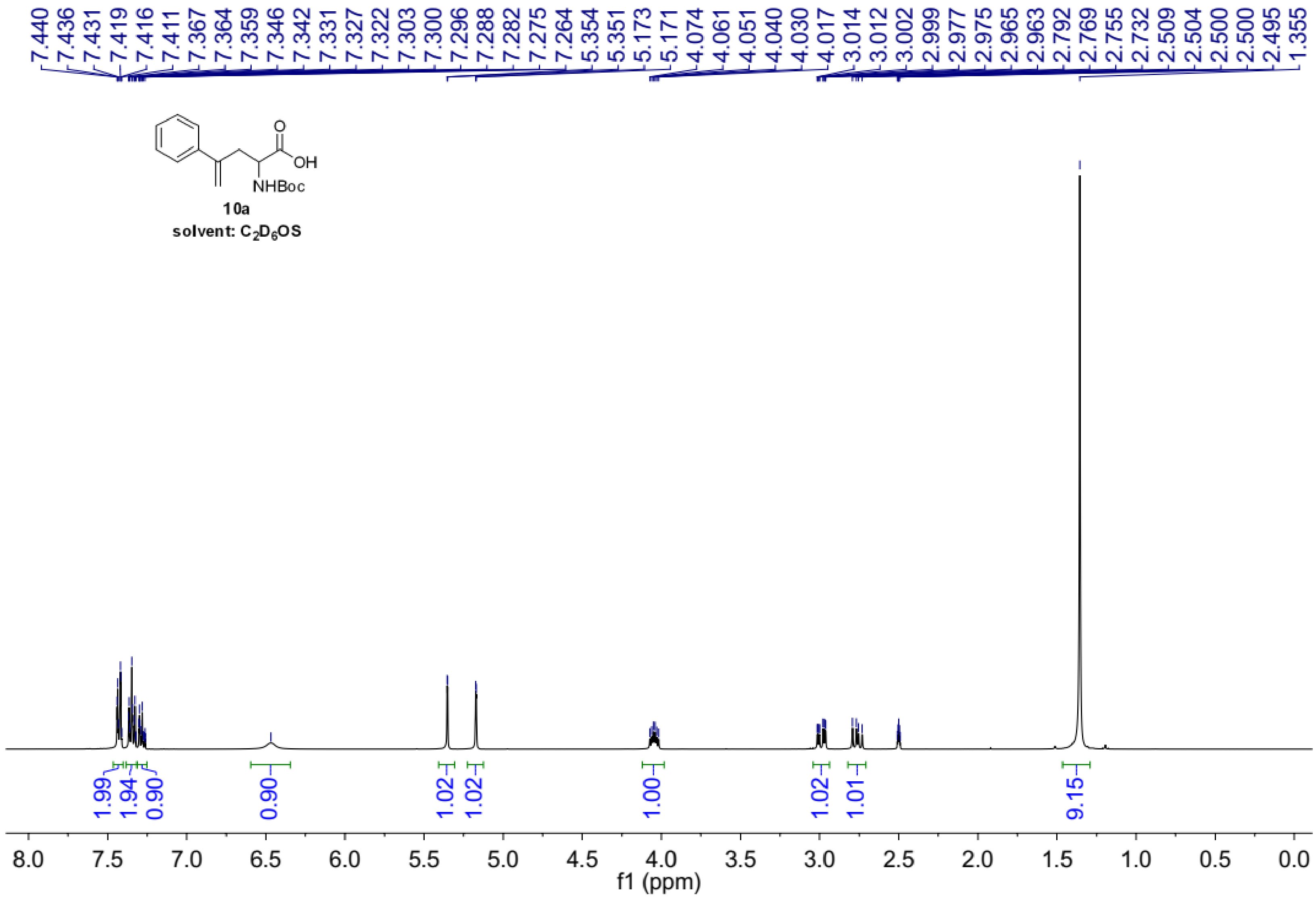

Supplementary Figure 266. ^{1}H NMR spectrum for compound **10a**

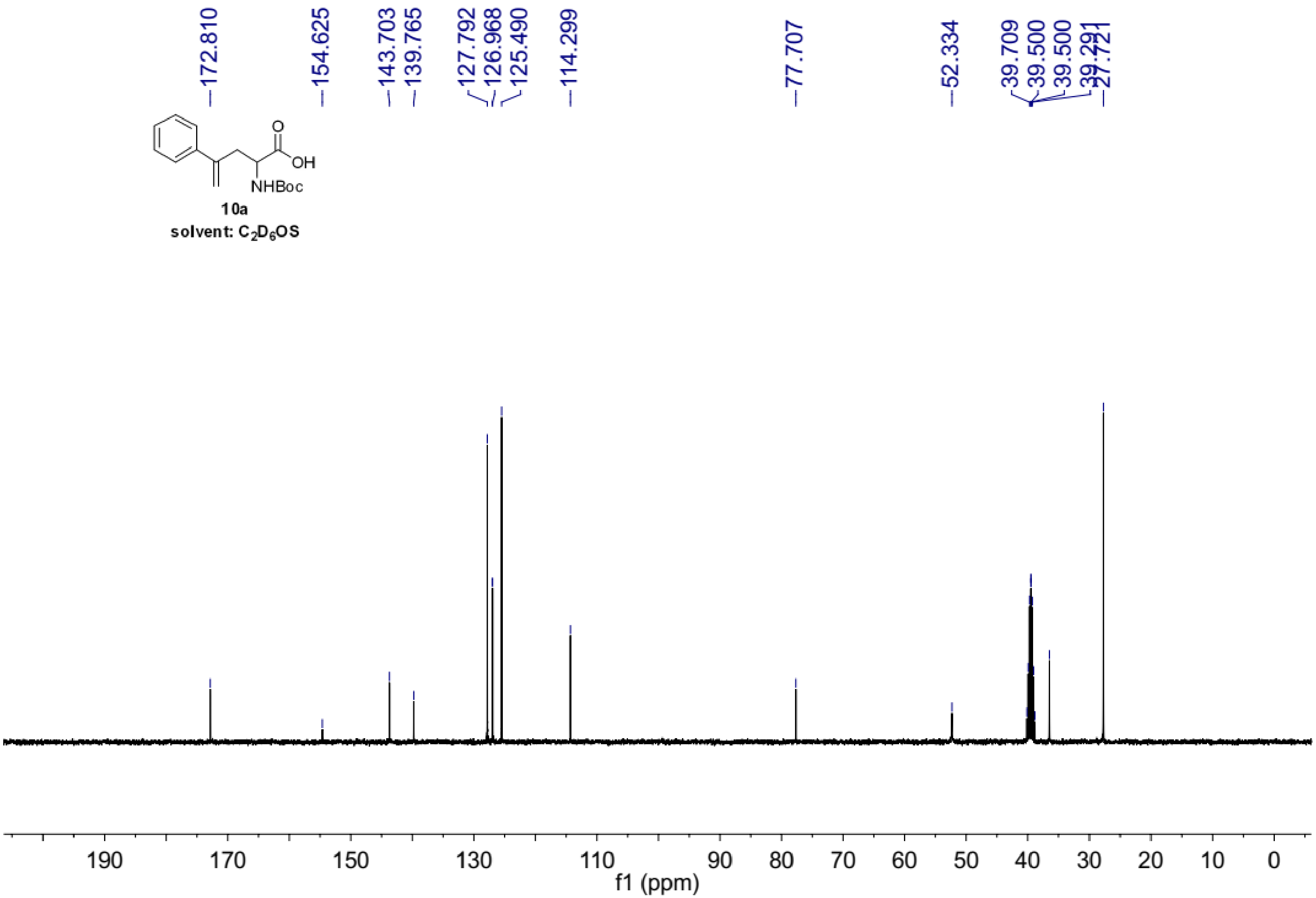

Supplementary Figure 267. ^{13}C NMR spectrum for compound **10a**

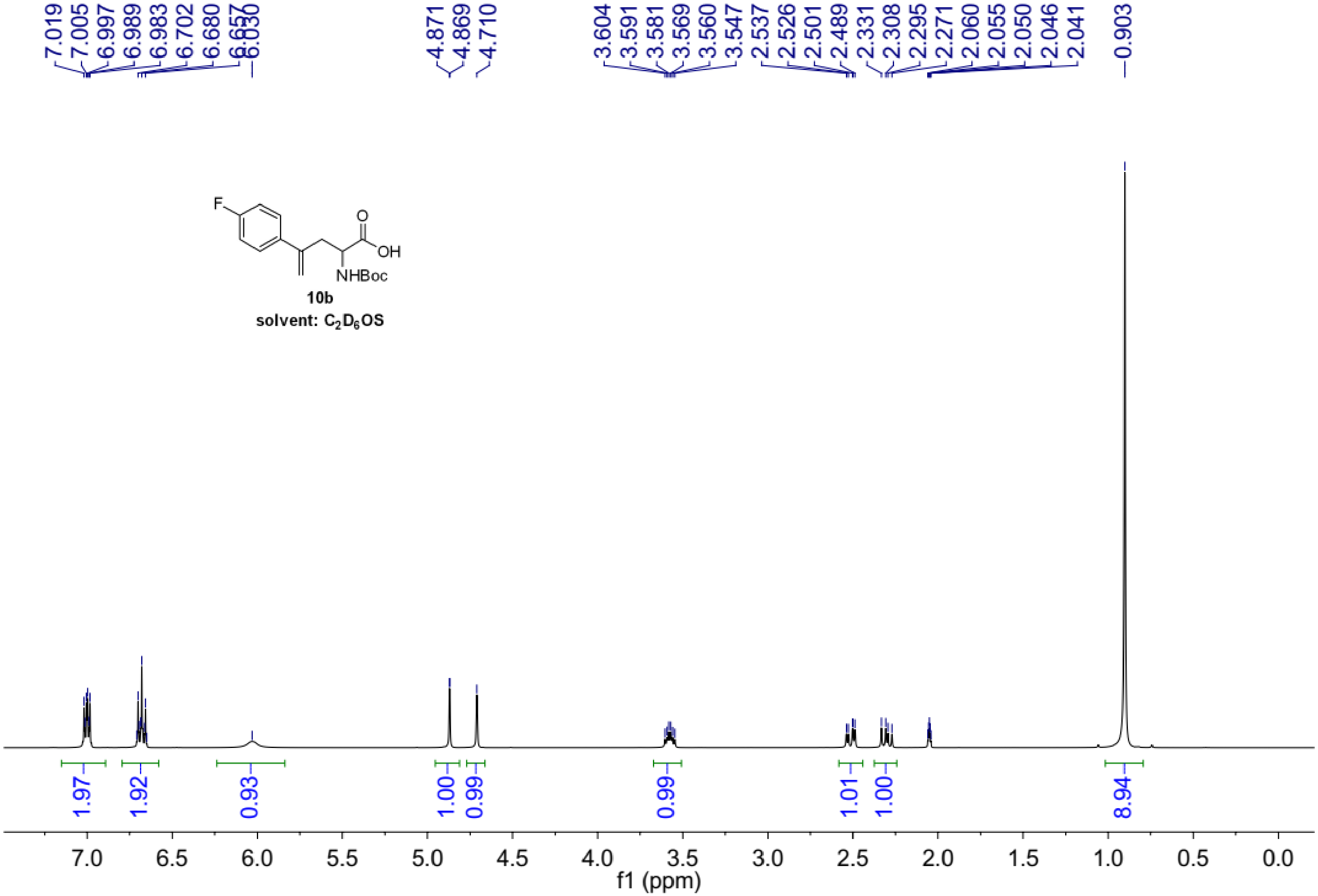

Supplementary Figure 268. ^{1}H NMR spectrum for compound **10b**

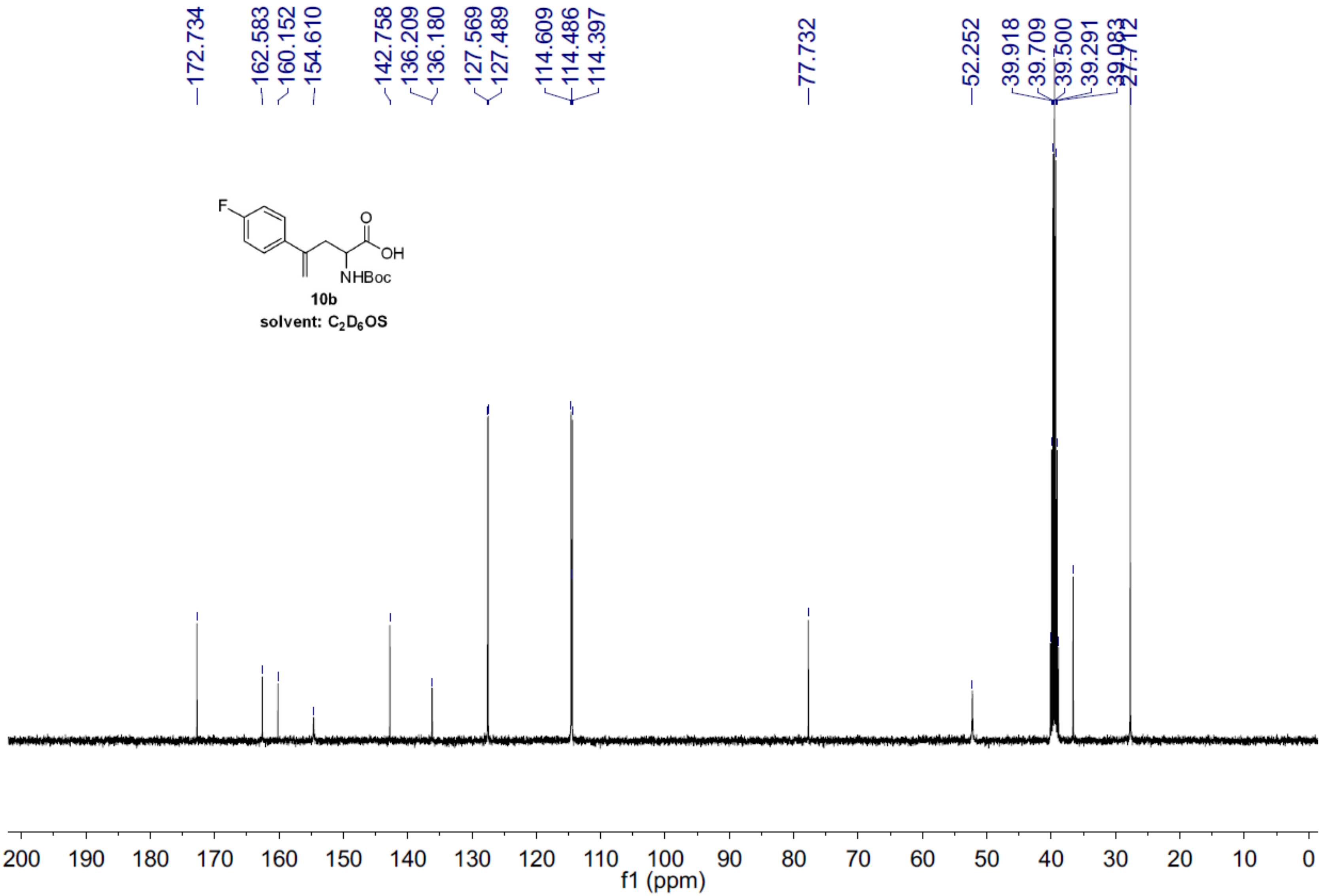

Supplementary Figure 269. ^{13}C NMR spectrum for compound **10b**

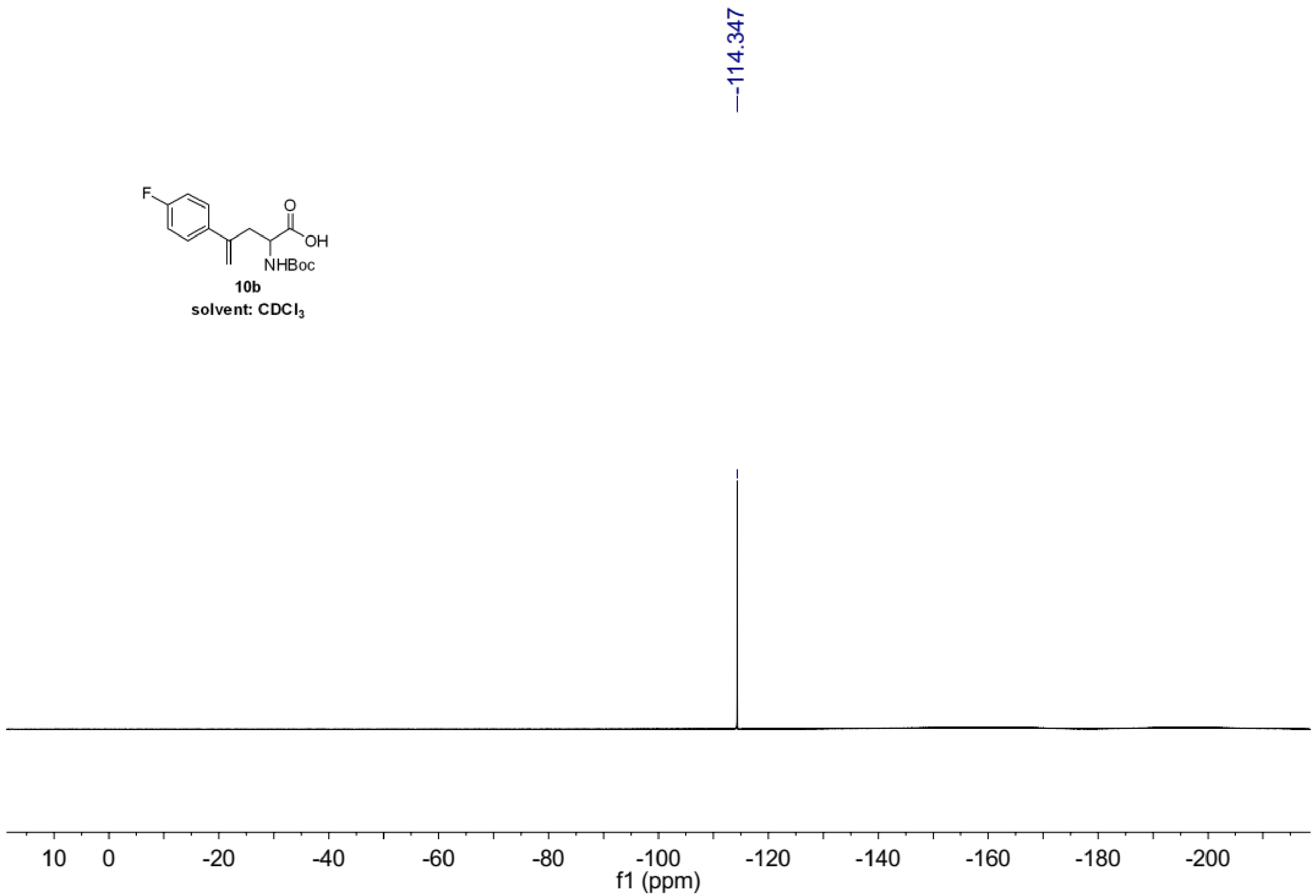

Supplementary Figure 270. ^{19}F NMR spectrum for compound **10b**

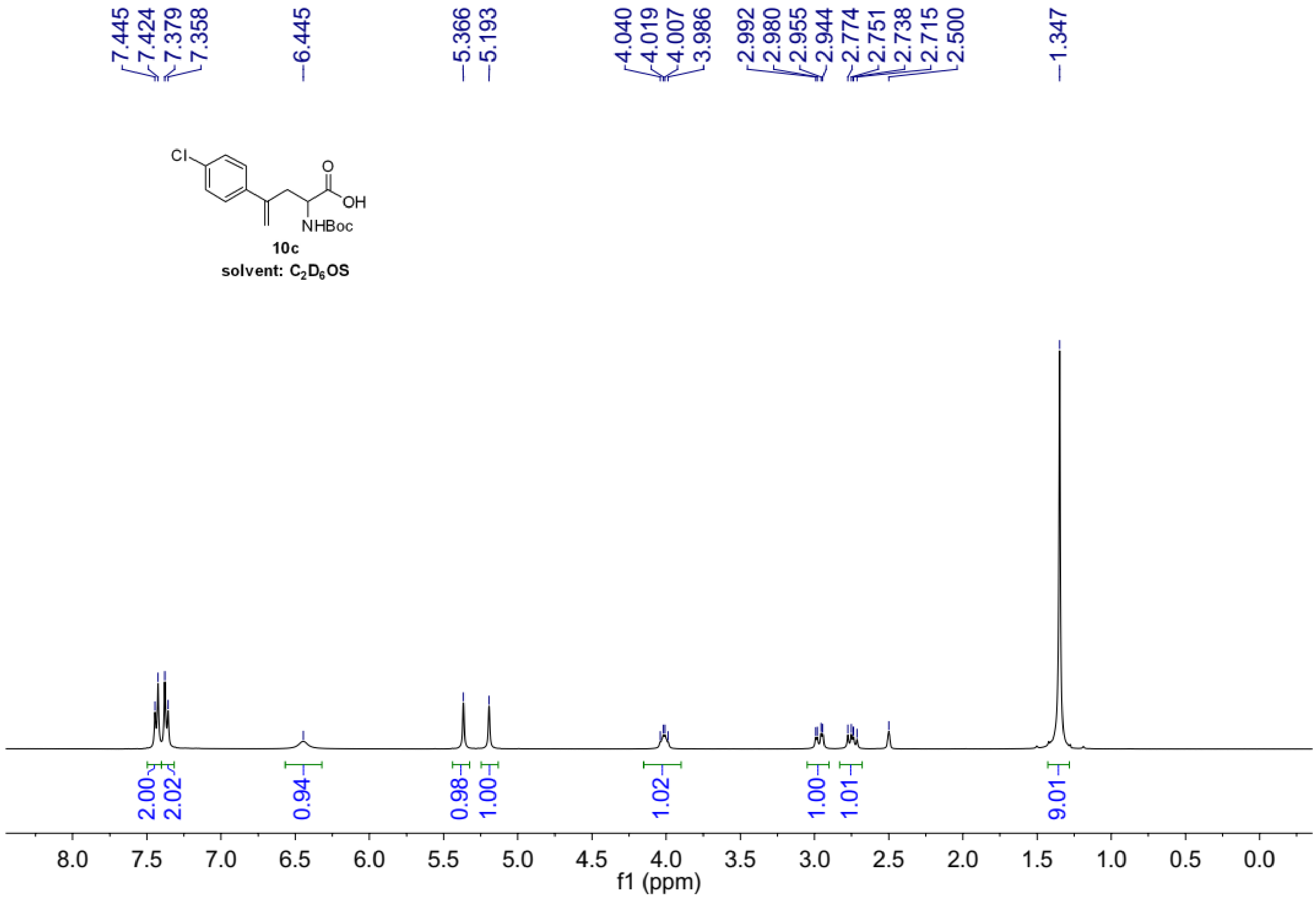

Supplementary Figure 271. ^{1}H NMR spectrum for compound **10c**

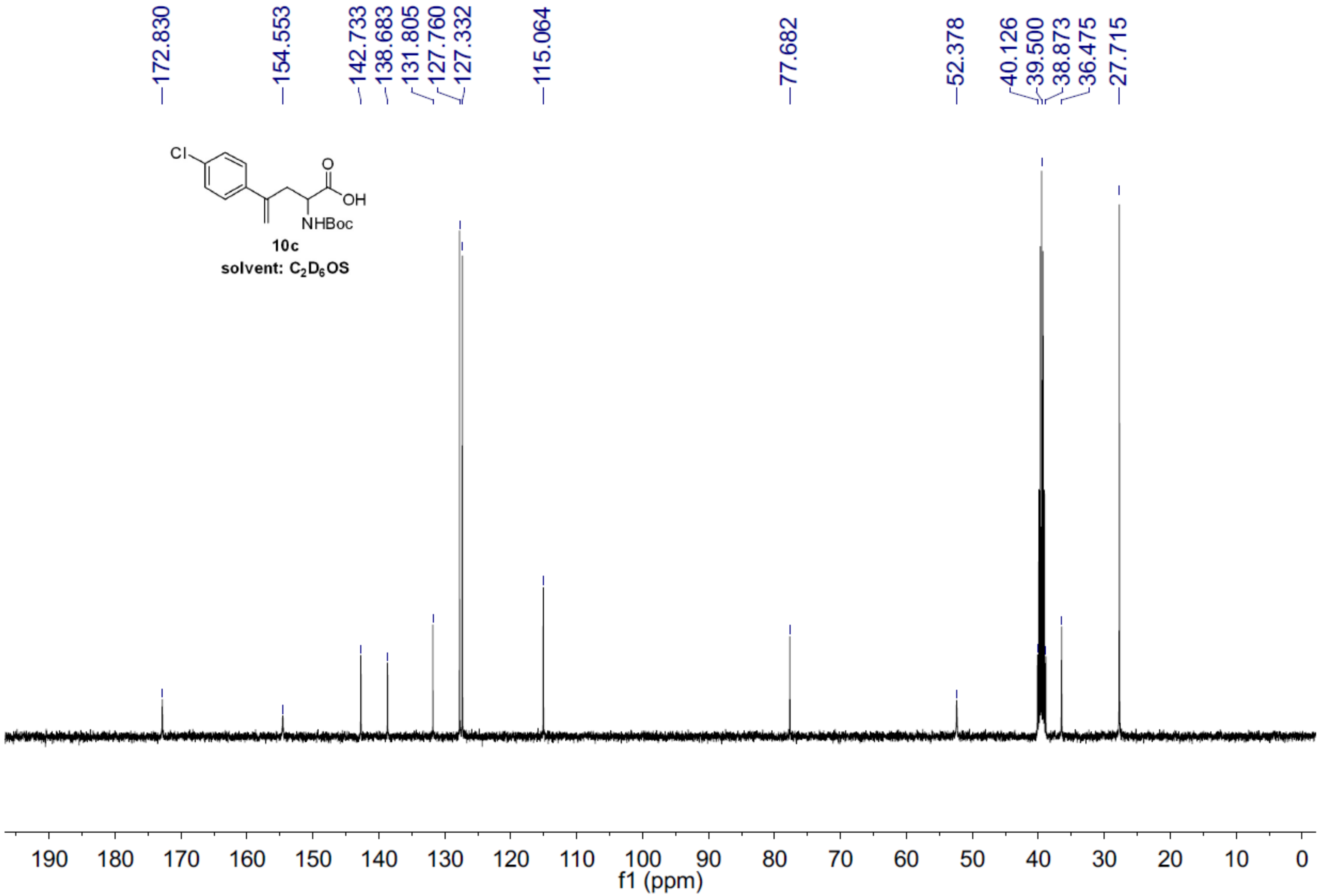

Supplementary Figure 272. ^{13}C NMR spectrum for compound **10c**

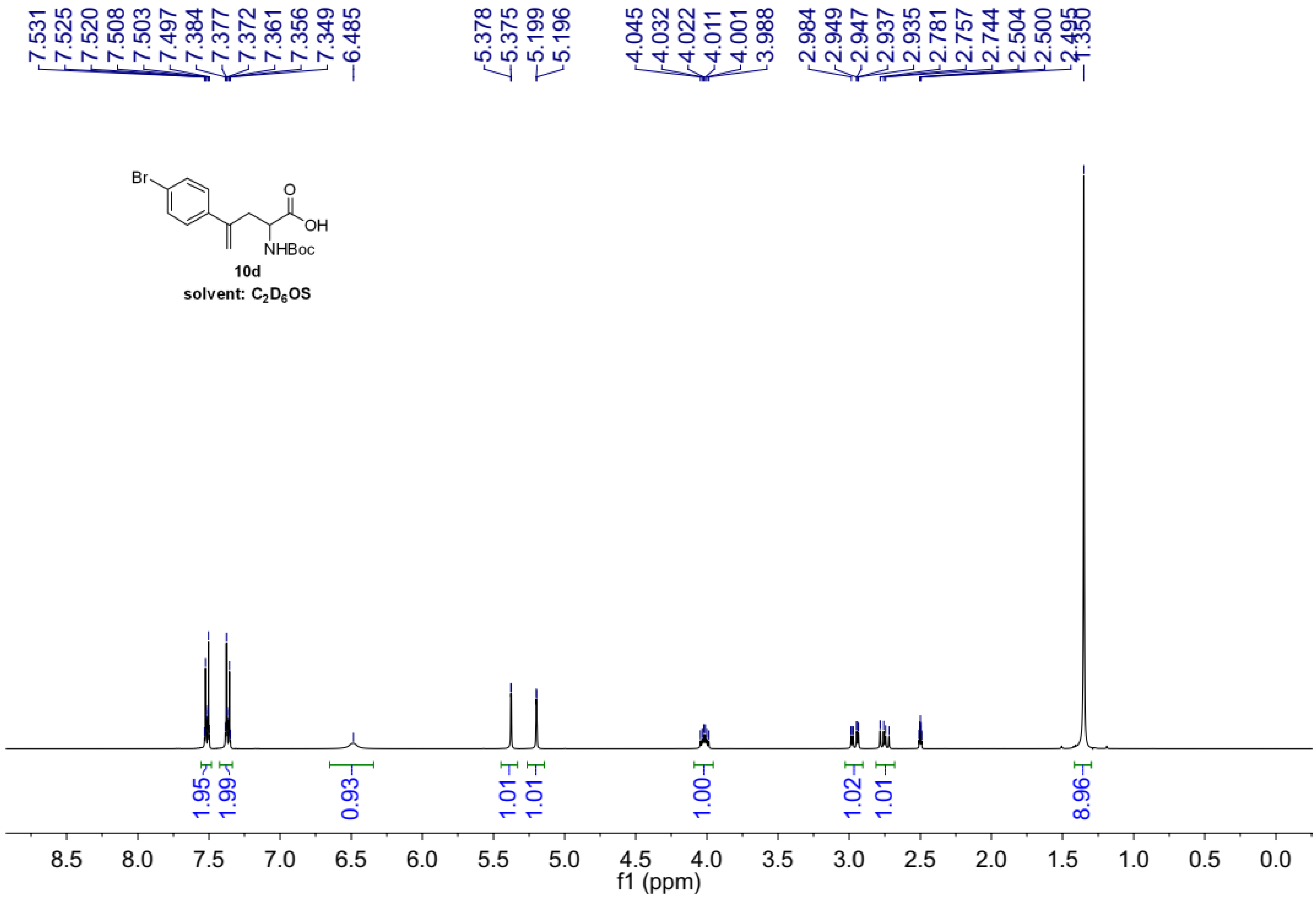

Supplementary Figure 273. ^{1}H NMR spectrum for compound **10d**

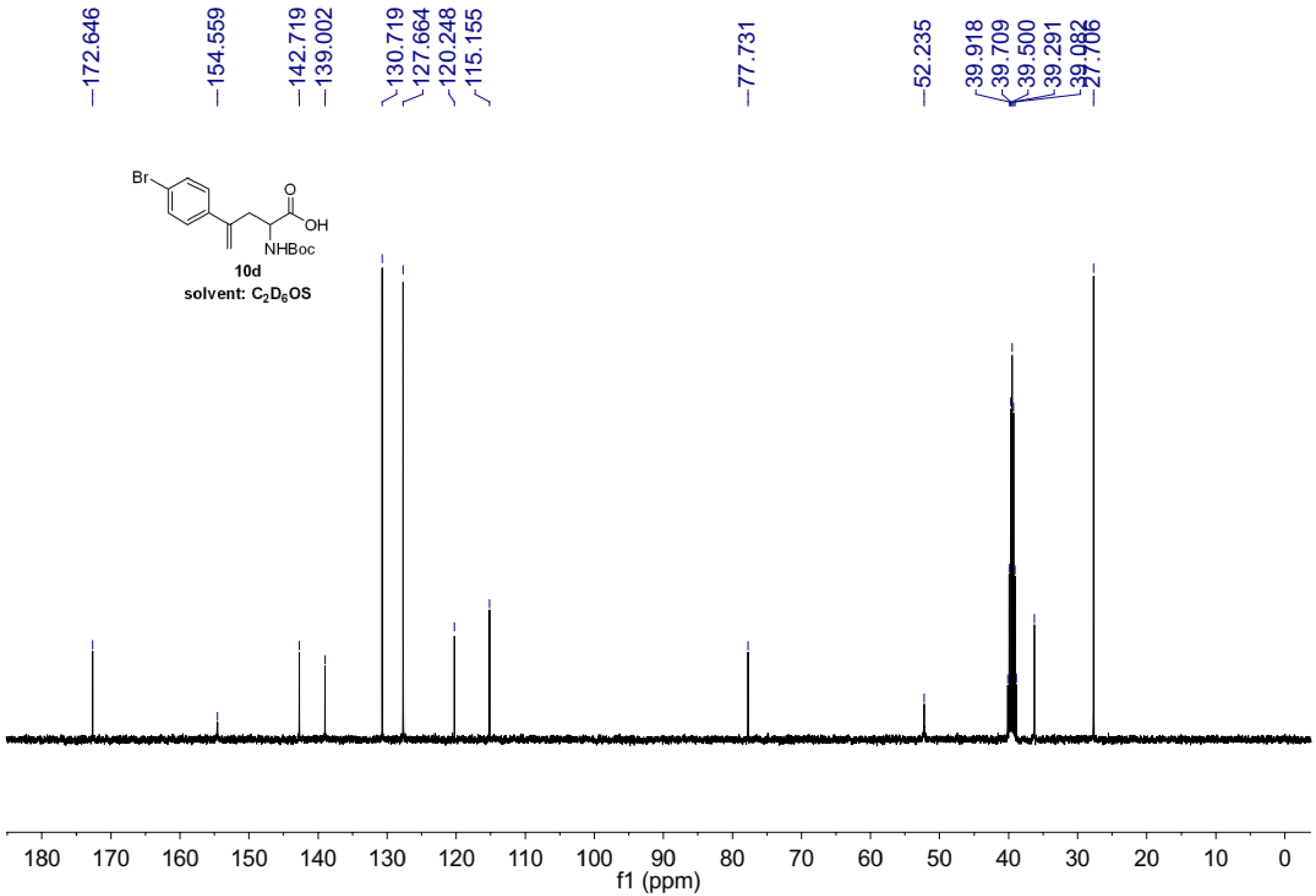

Supplementary Figure 274. ^{13}C NMR spectrum for compound **10d**

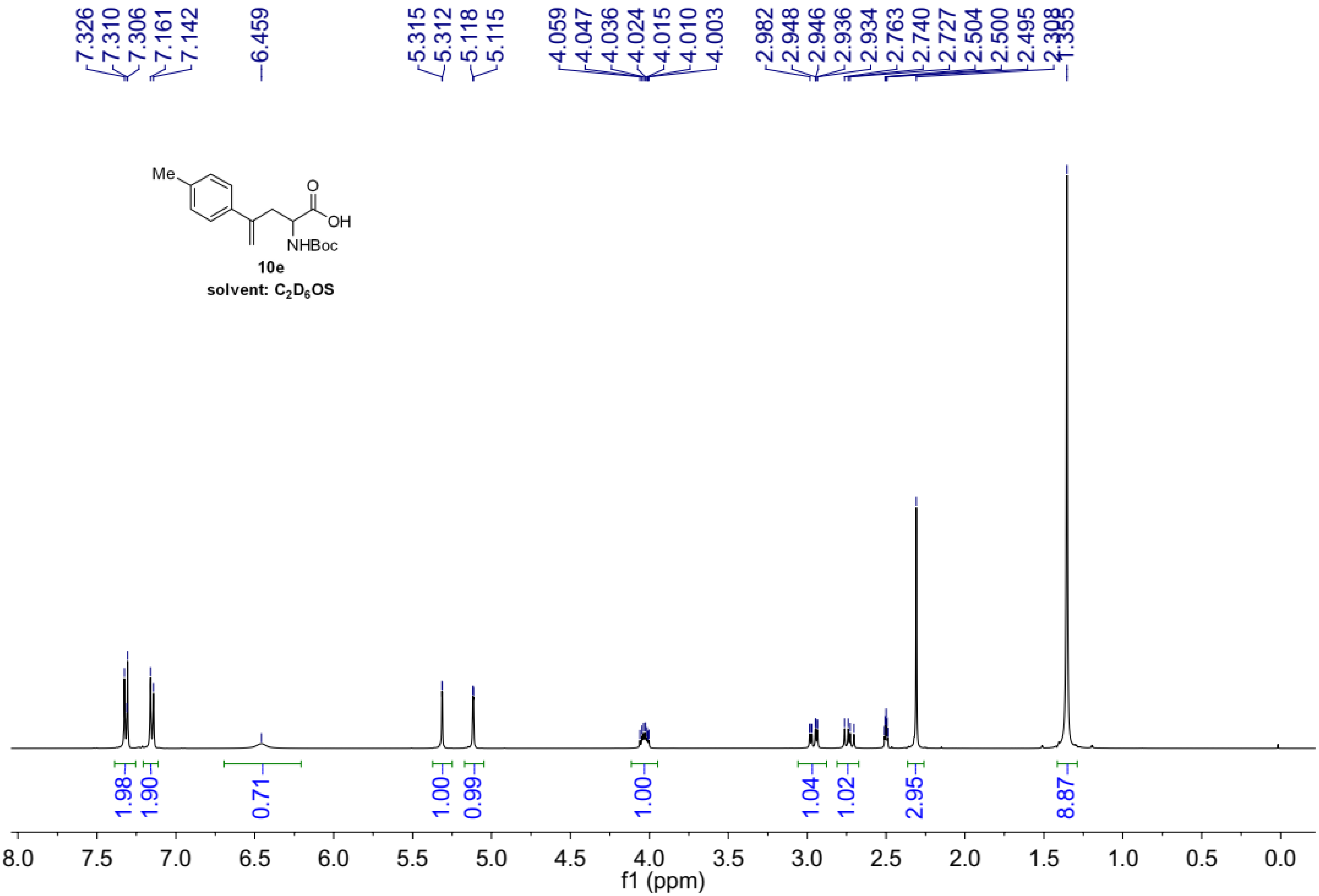

Supplementary Figure 275. ^{1}H NMR spectrum for compound **10e**

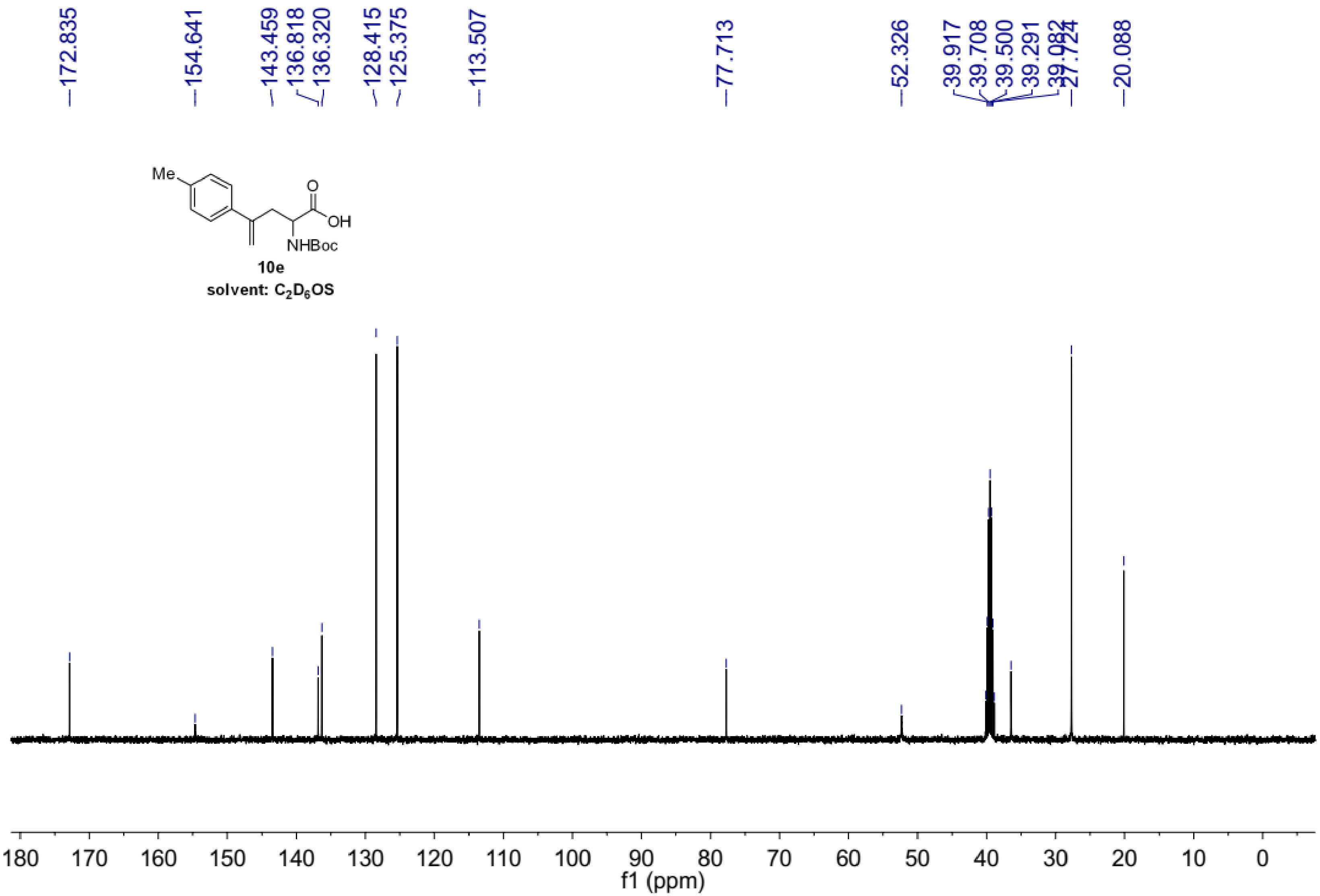

Supplementary Figure 276. ^{13}C NMR spectrum for compound **10e**

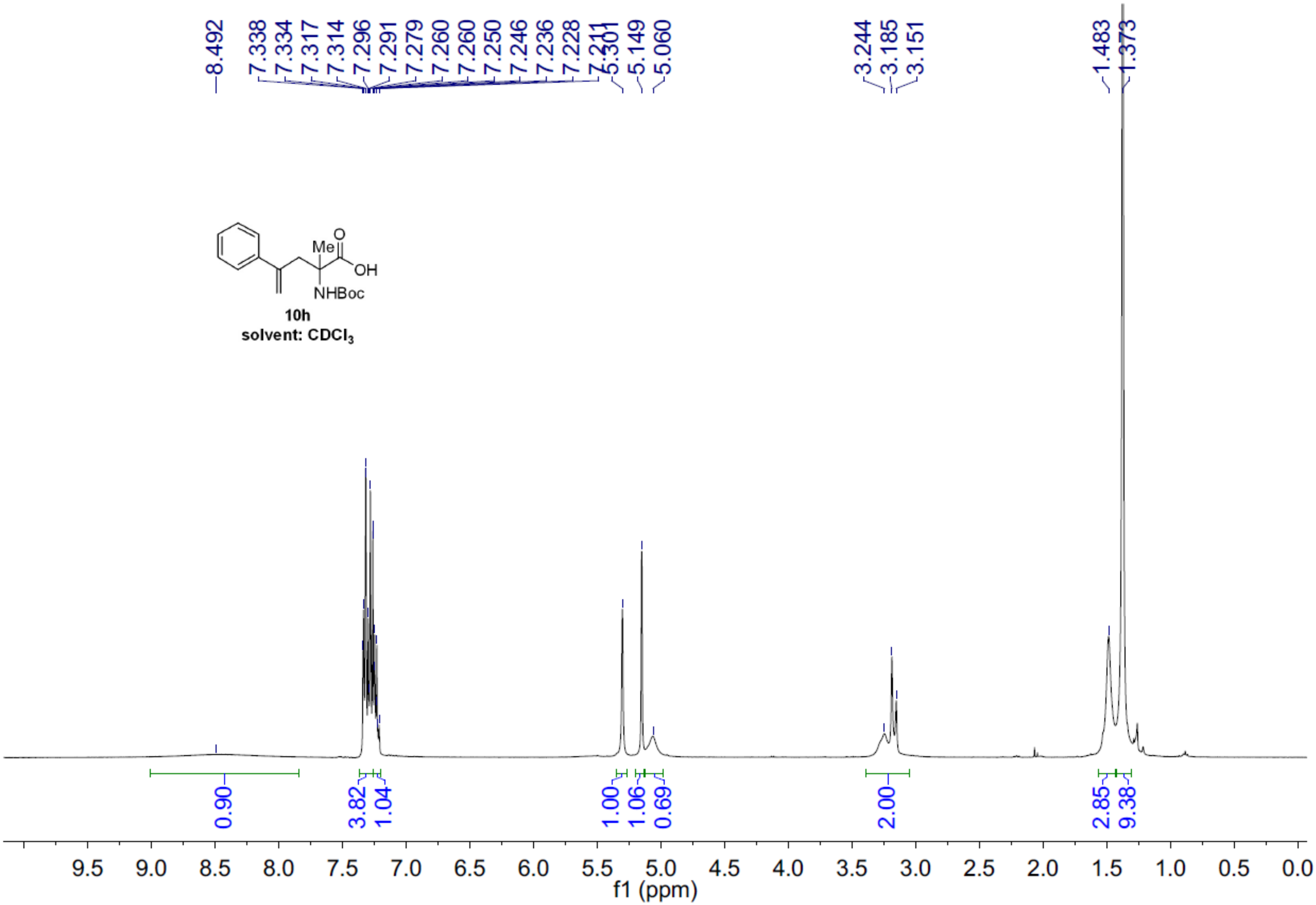

Supplementary Figure 277. ^{1}H NMR spectrum for compound **10h**

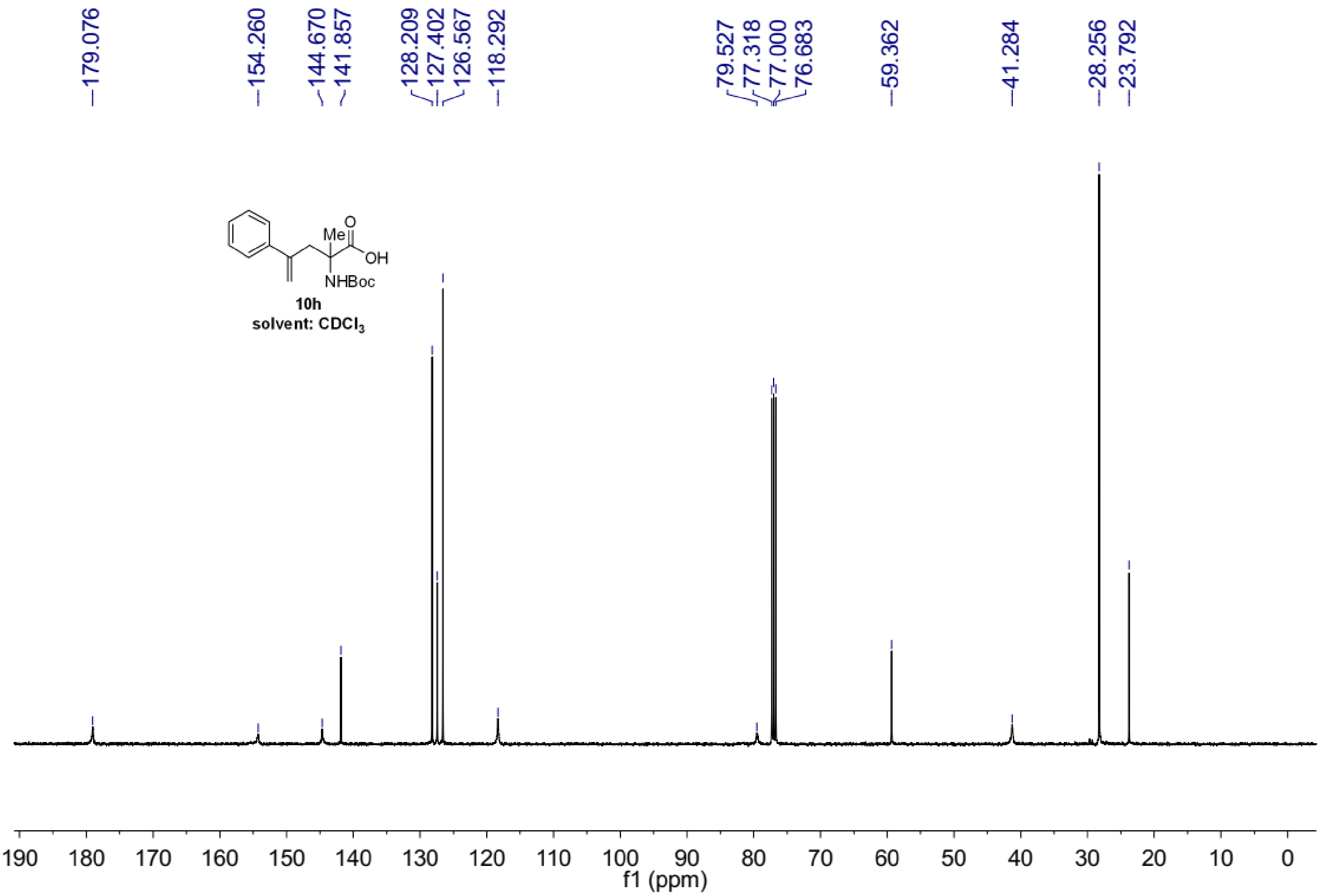

Supplementary Figure 278. ^{13}C NMR spectrum for compound **10h**

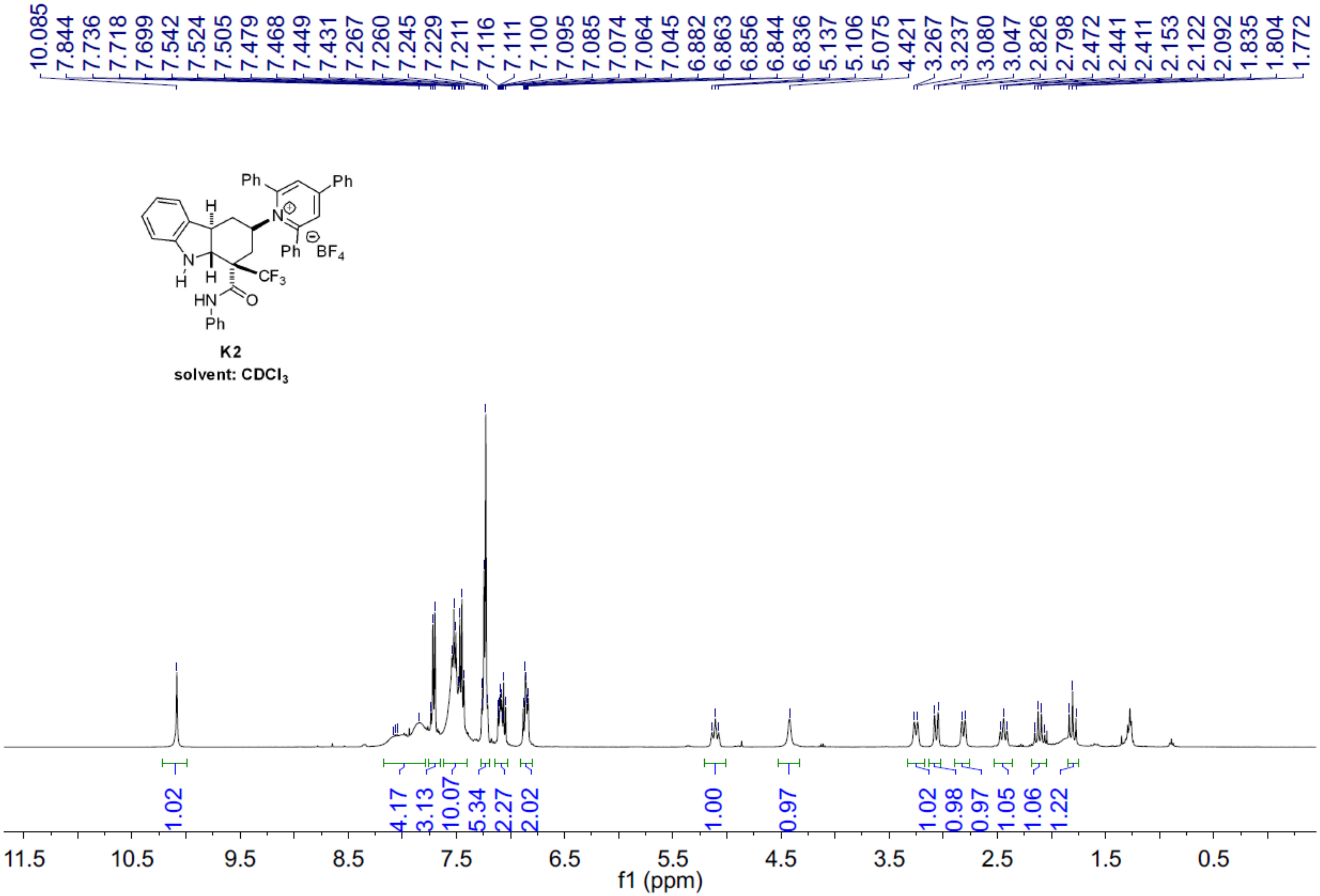

Supplementary Figure 279. ^1H NMR spectrum for compound **K2**

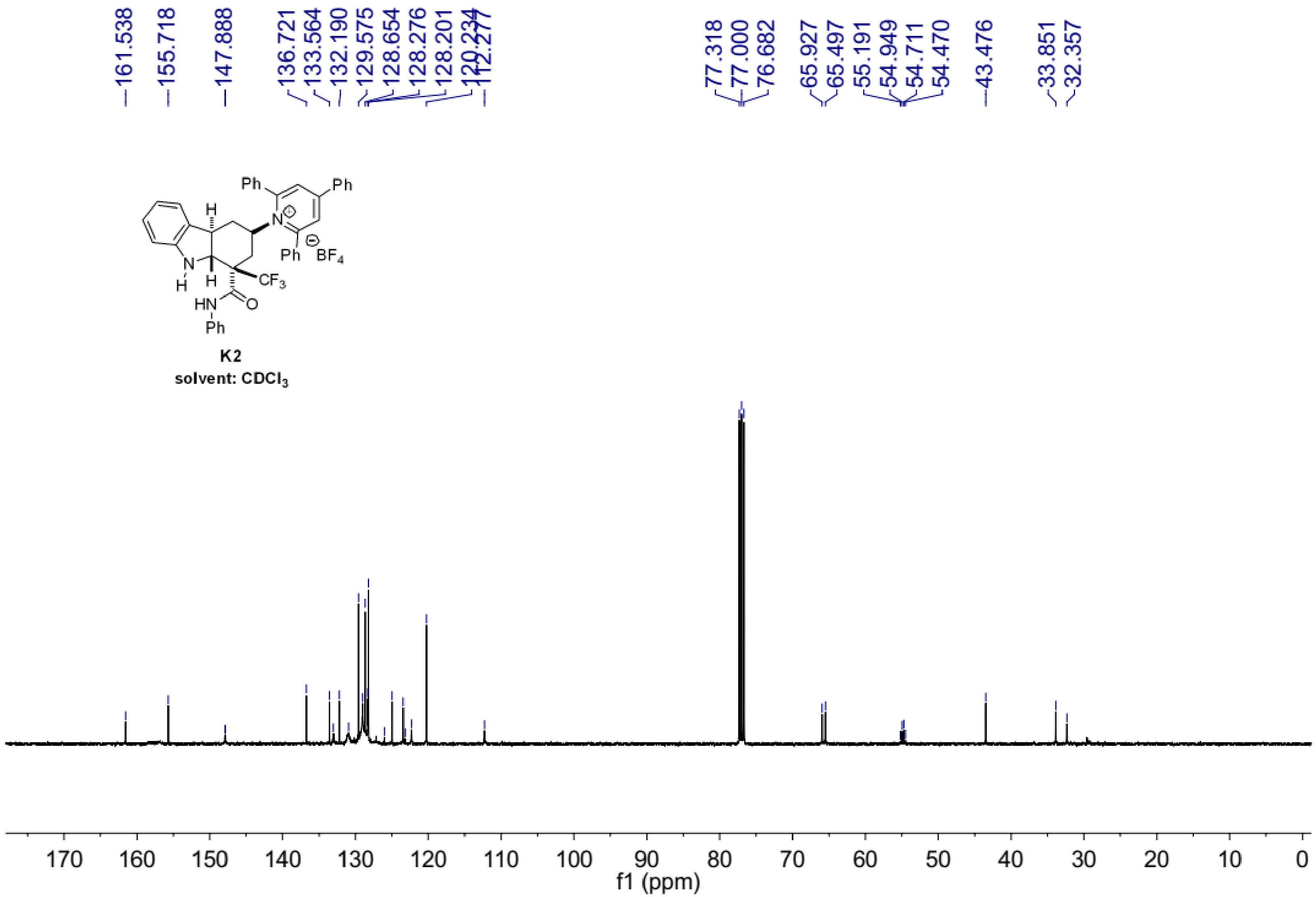

Supplementary Figure 280. ^{13}C NMR spectrum for compound **K2**

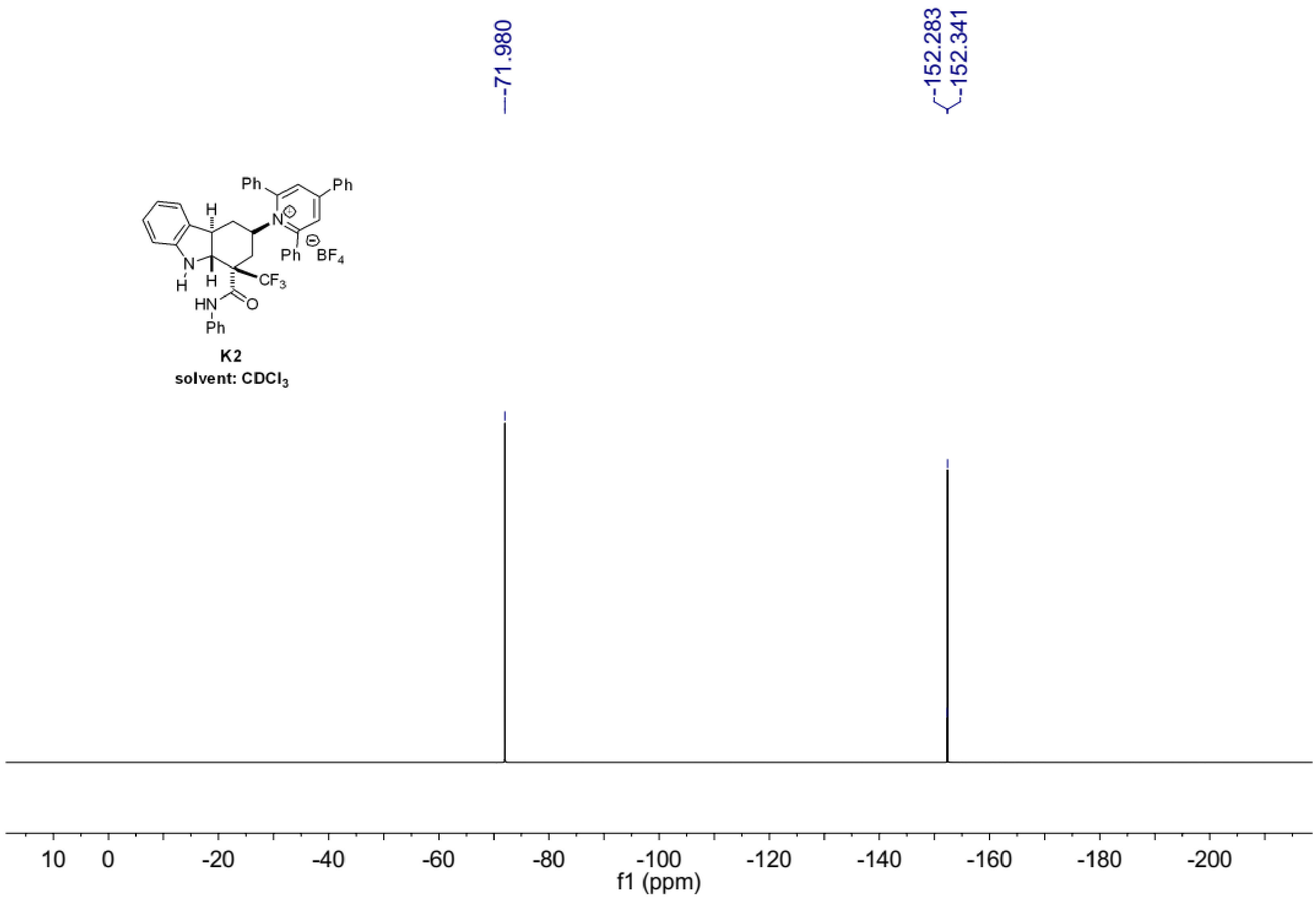

Supplementary Figure 281. ^{19}F NMR spectrum for compound **K2**

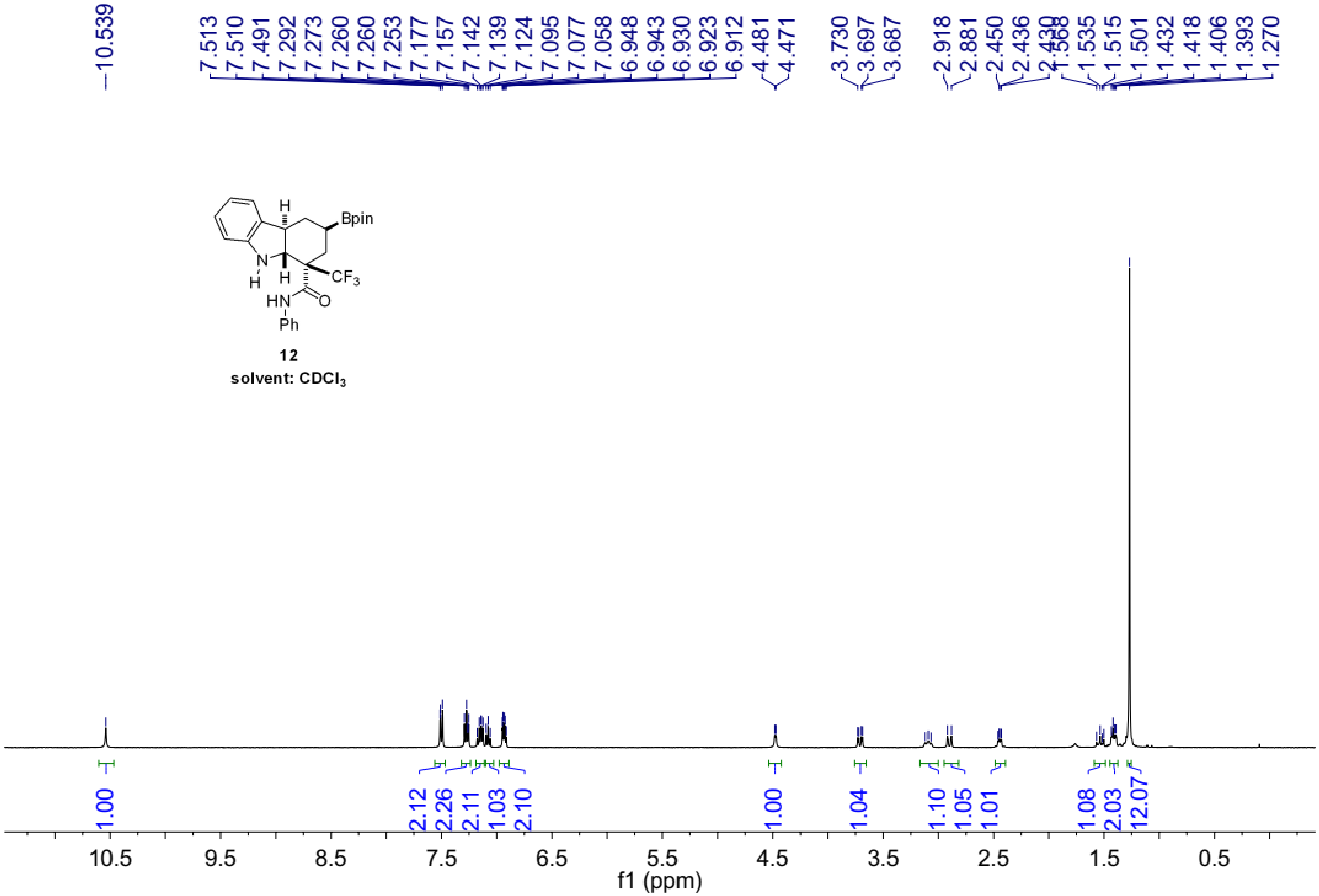

Supplementary Figure 282. ^{1}H NMR spectrum for compound **12**

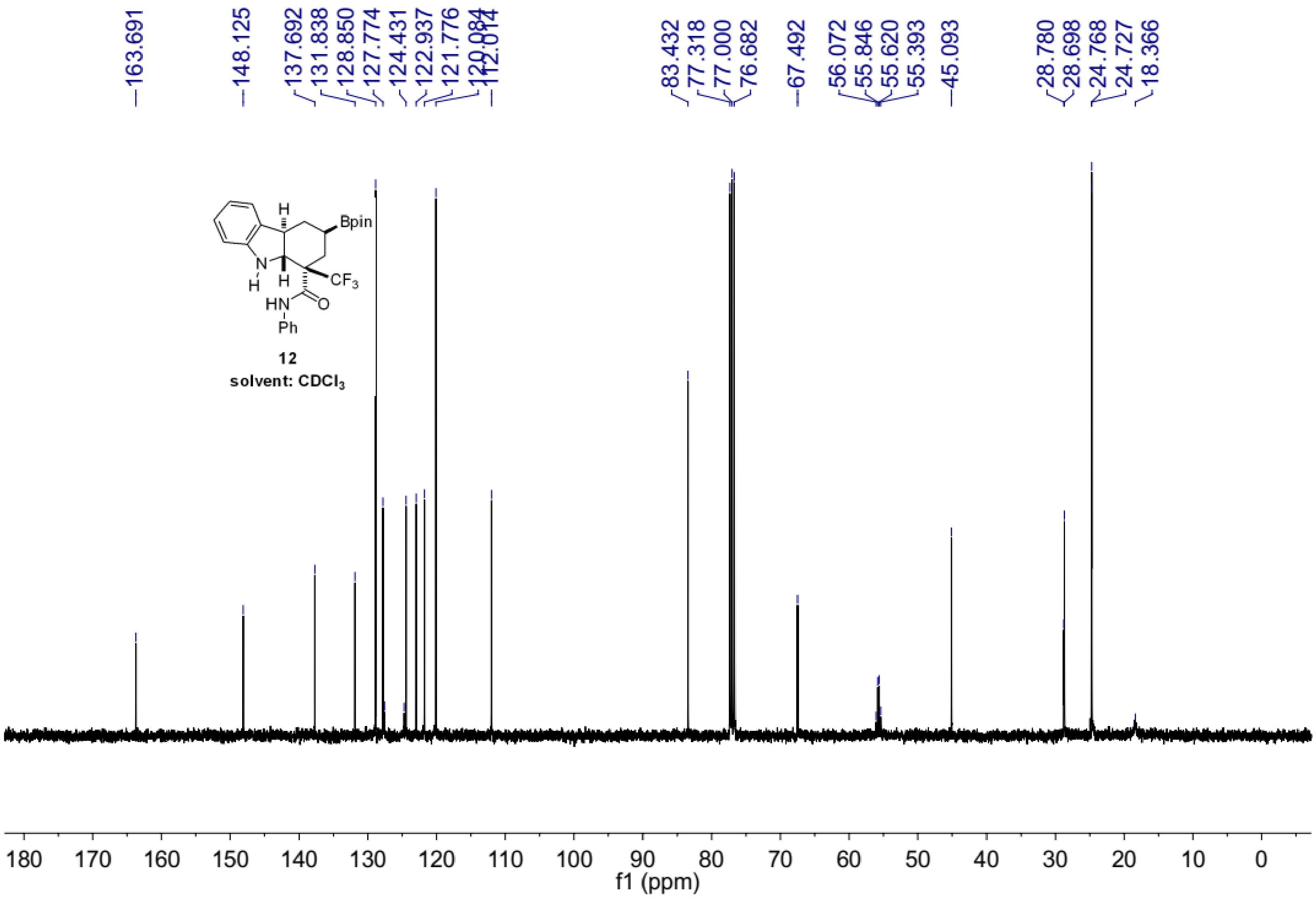

Supplementary Figure 283. ${}^{13}C$ NMR spectrum for compound **12**

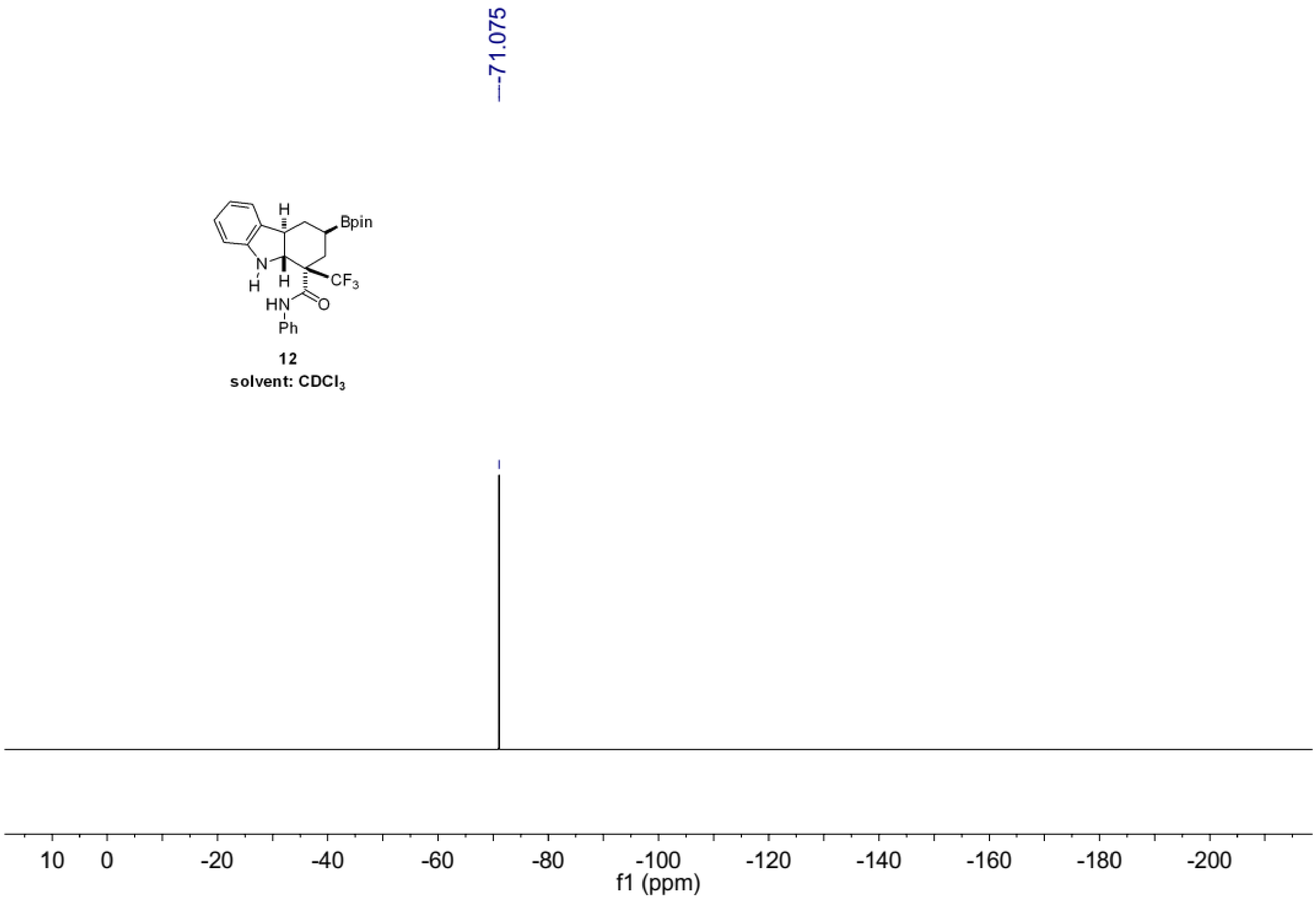

Supplementary Figure 284. ^{19}F NMR spectrum for compound **12**

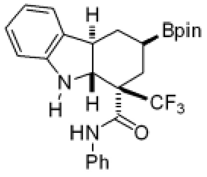

12
solvent: $CDCl_3$

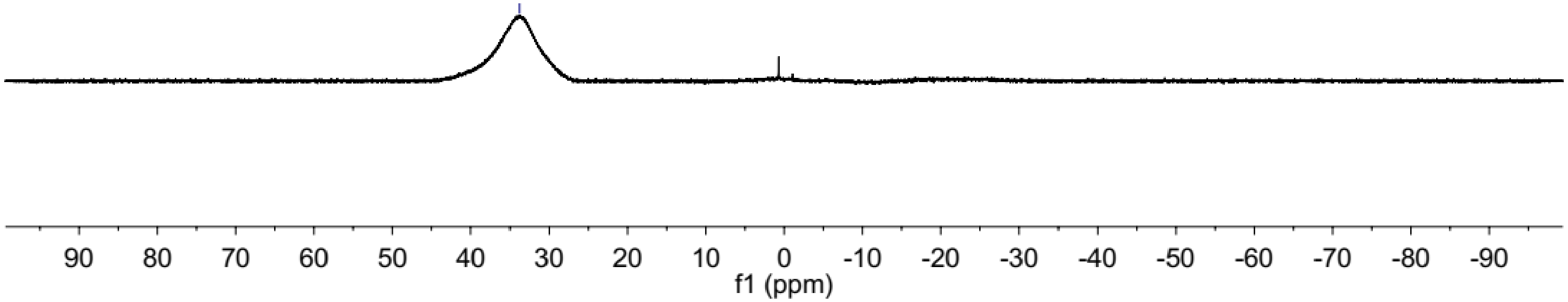

Supplementary Figure 285. ^{11}B NMR spectrum for compound **12**

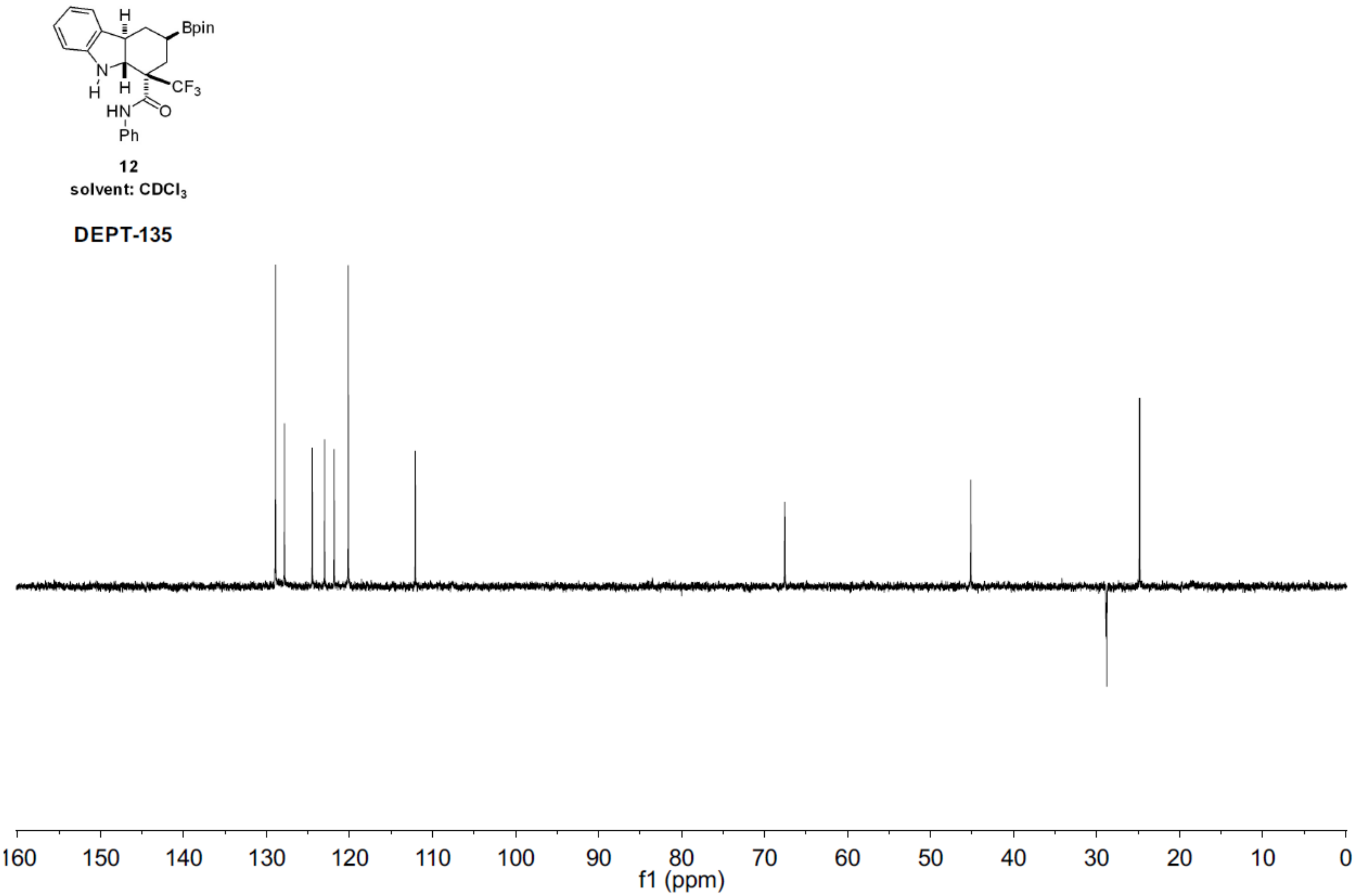

Supplementary Figure 286. DEPT-135 spectrum for compound **12**

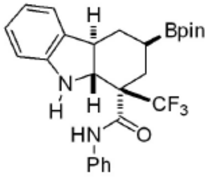

12
solvent: $CDCl_3$

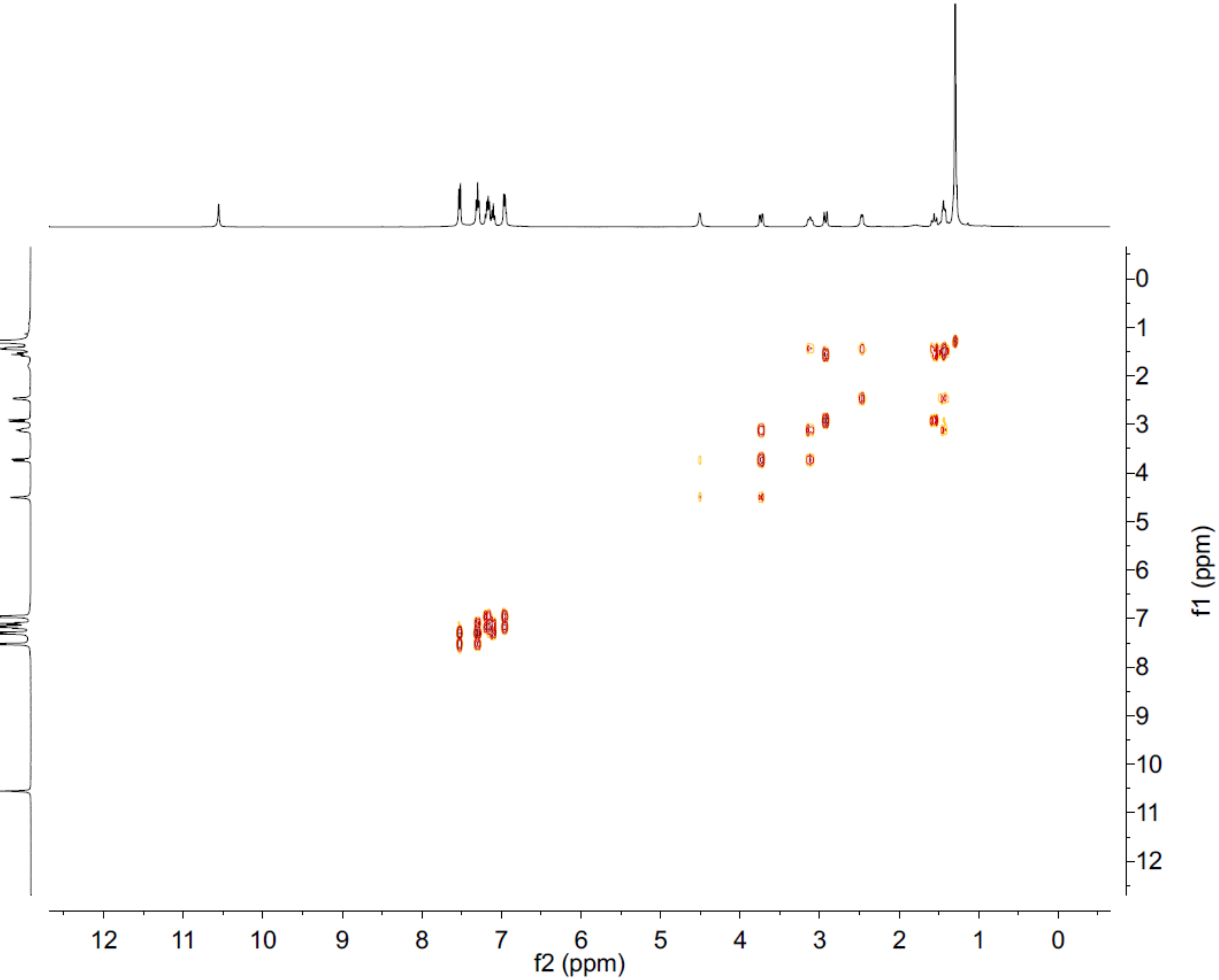

Supplementary Figure 287. H-H COSY spectrum for compound **12**

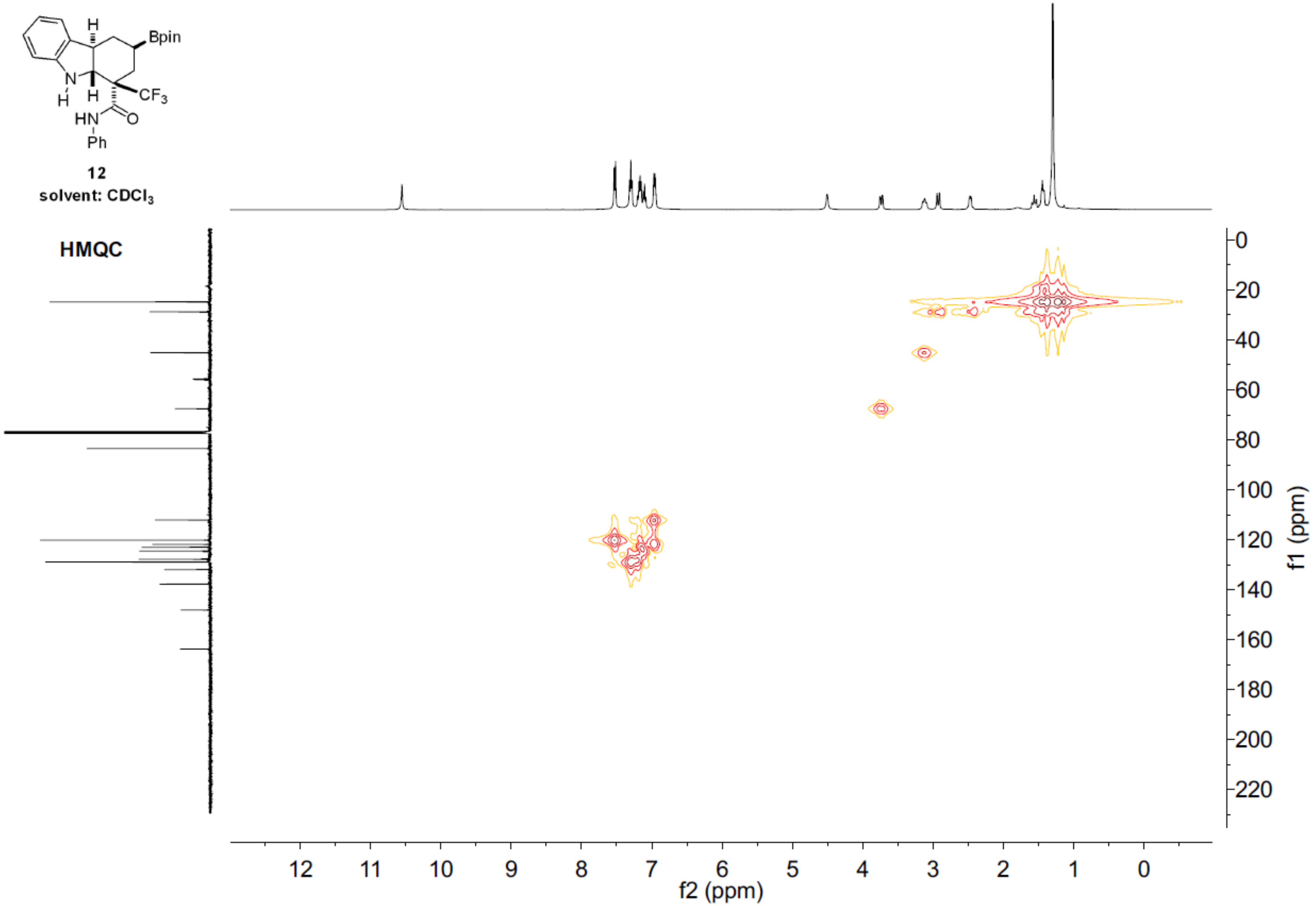

Supplementary Figure 288. HMQC spectrum for compound **12**

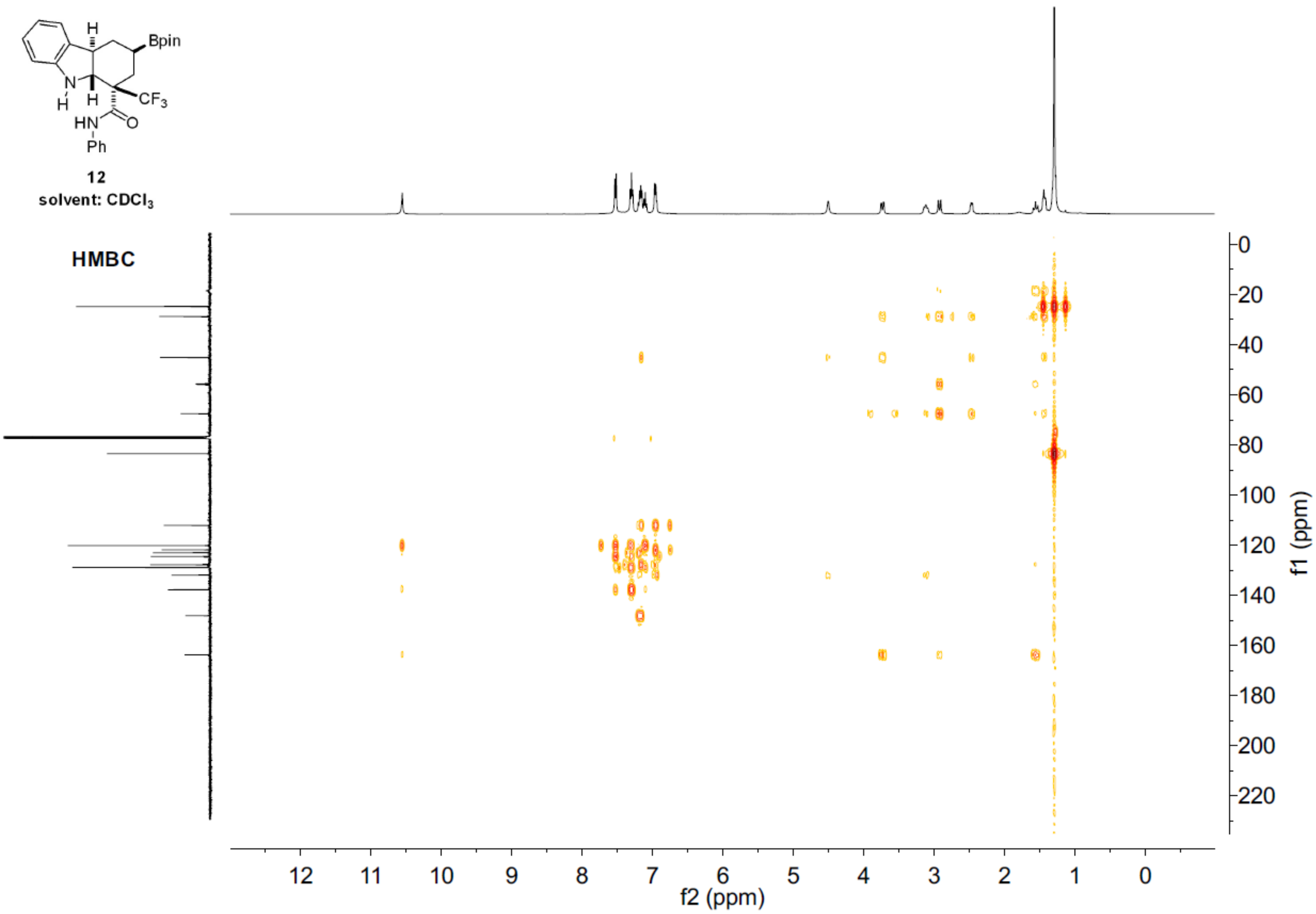

Supplementary Figure 289. HMBC spectrum for compound **12**

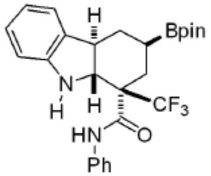

12
solvent: $CDCl_3$

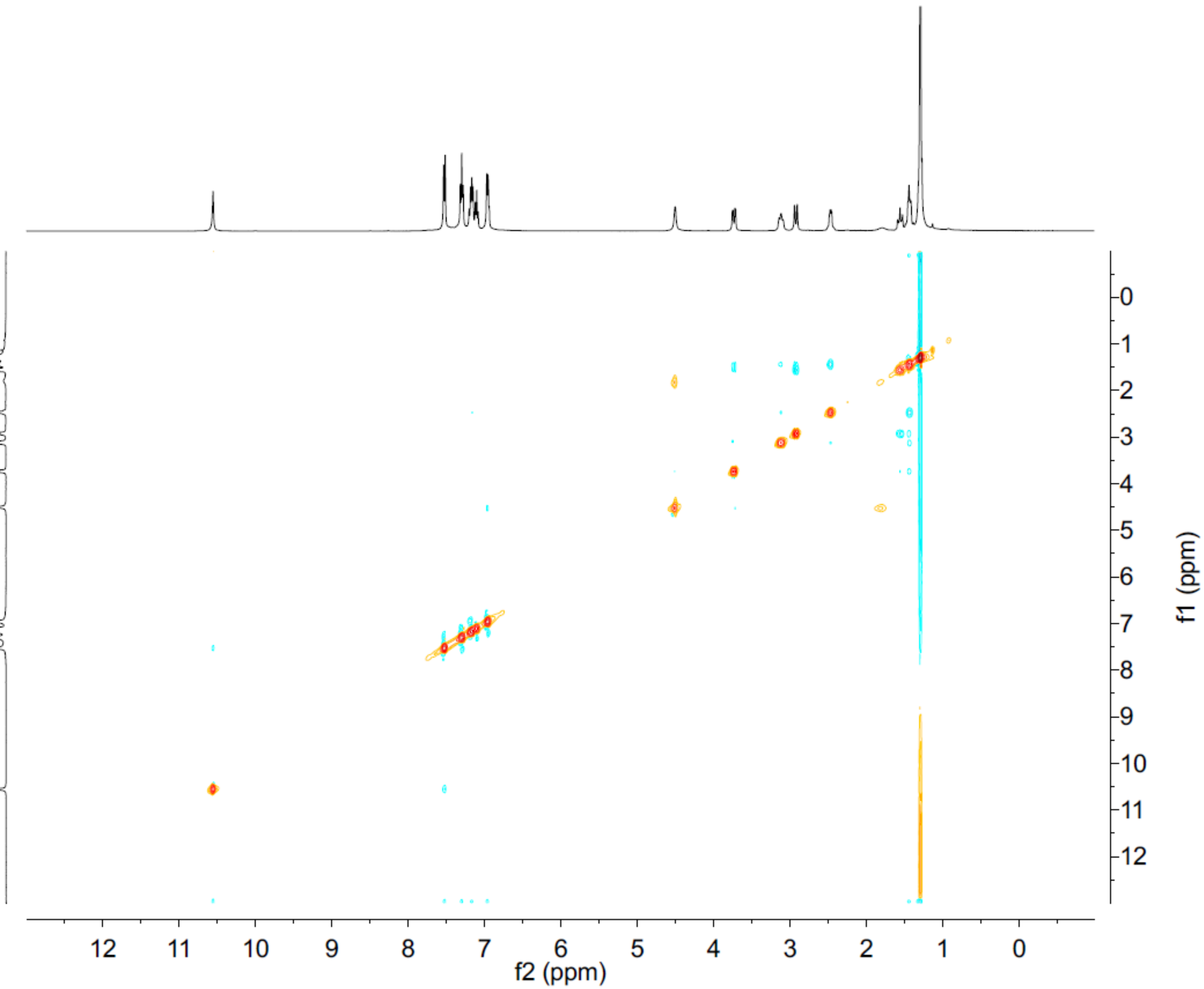

Supplementary Figure 290. NOE spectrum for compound **12**

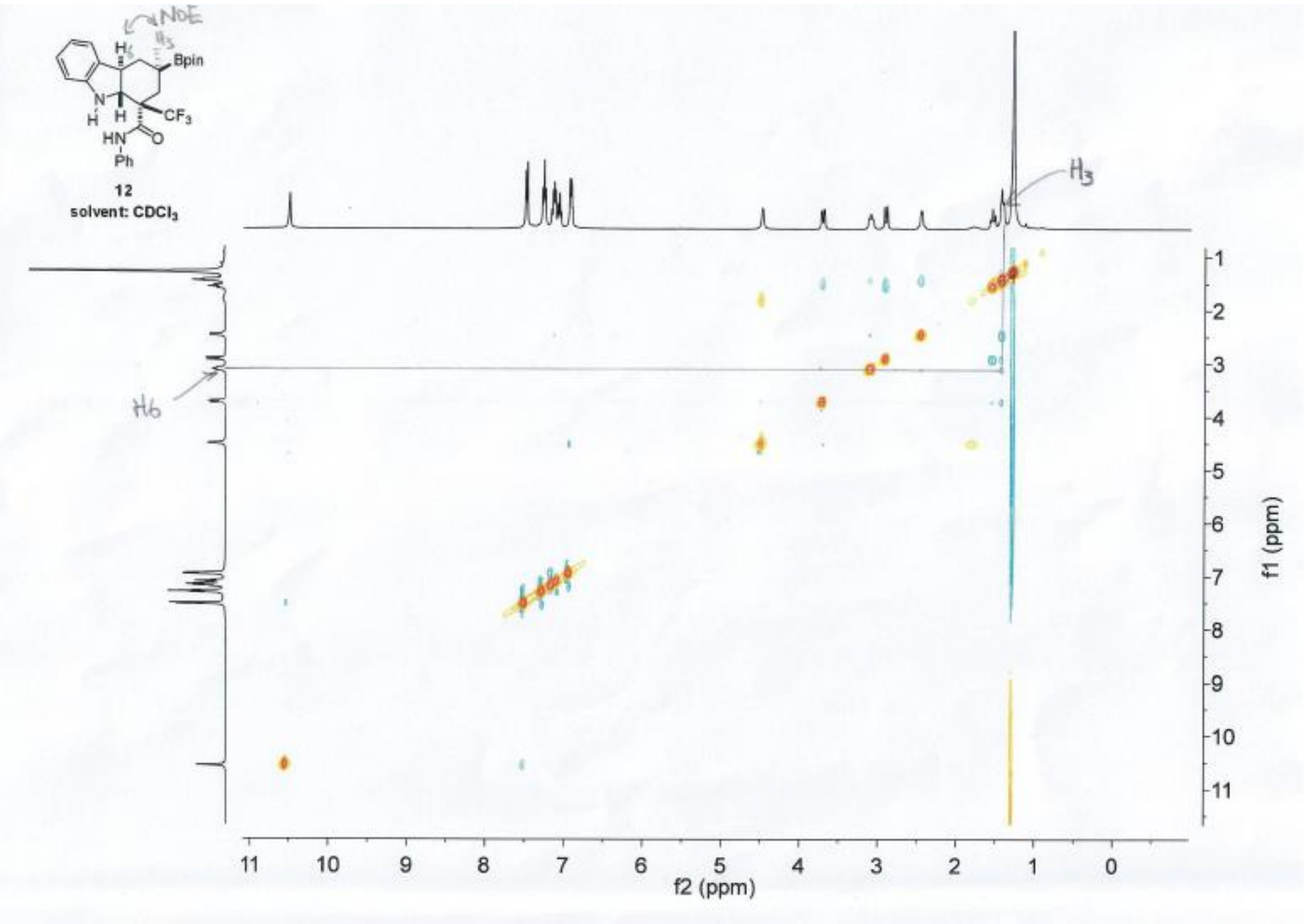

Supplementary Figure 291. NOE spectrum for compound **12**

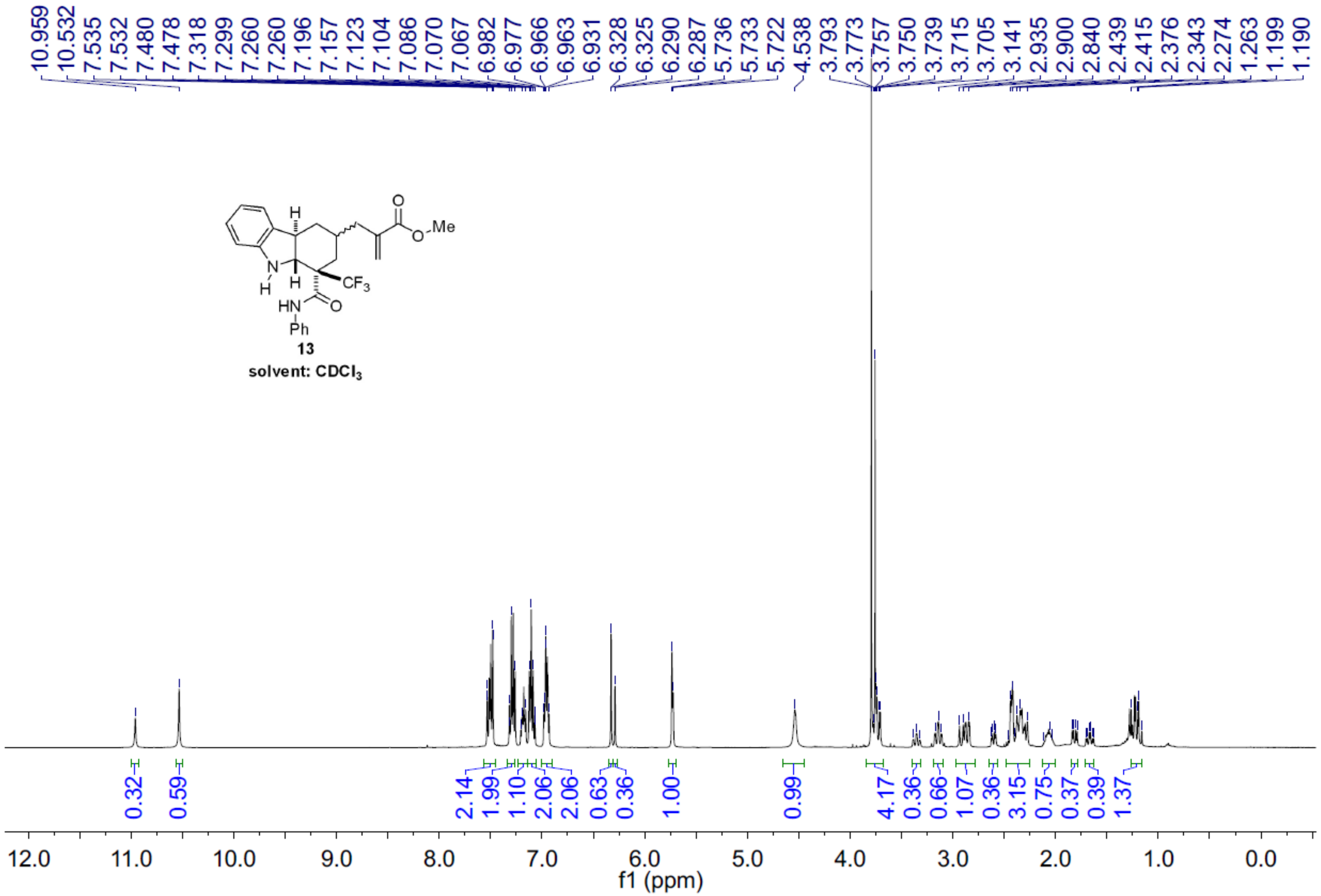

Supplementary Figure 292. ^{1}H NMR spectrum for compound **13**

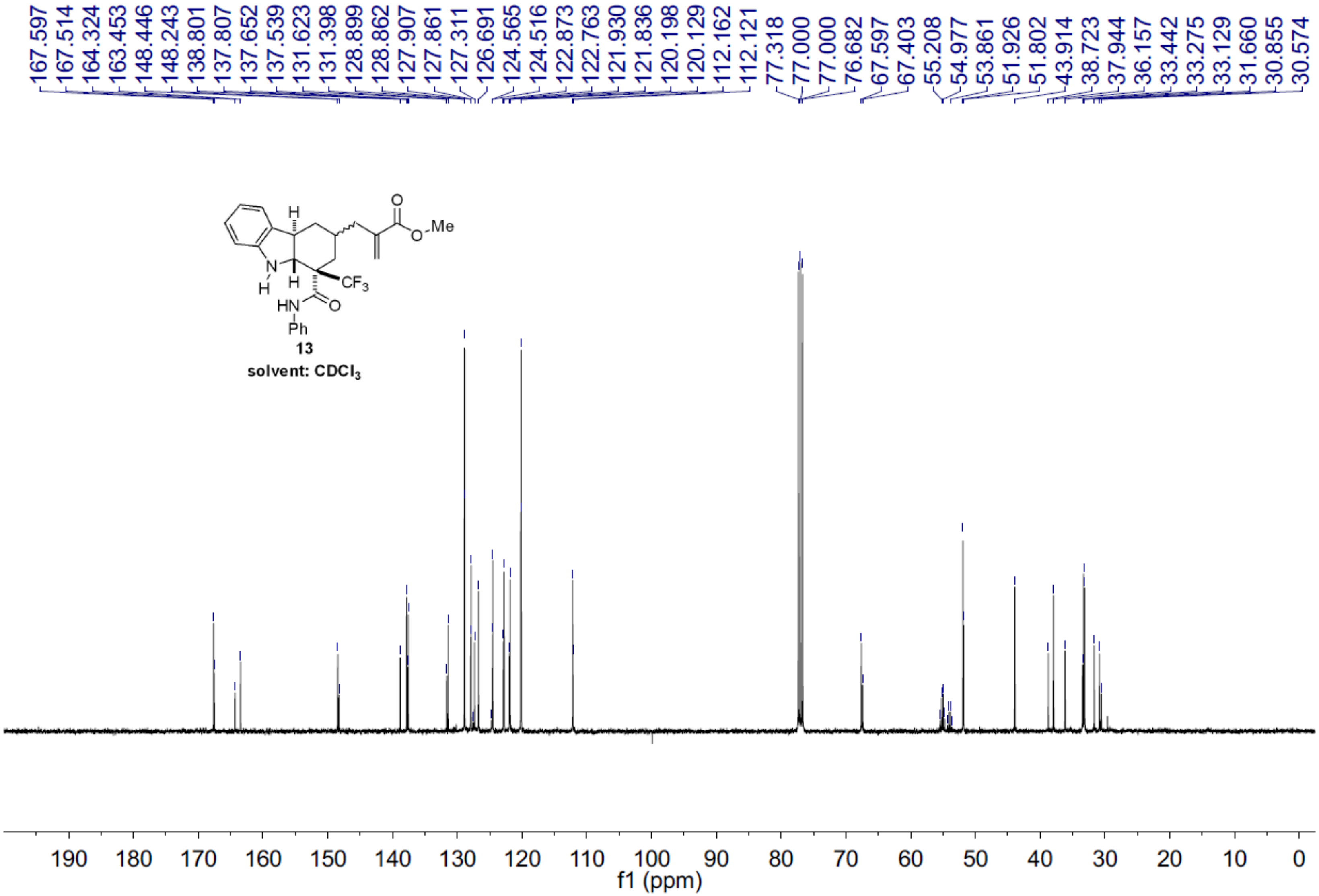

Supplementary Figure 293. ^{13}C NMR spectrum for compound **13**

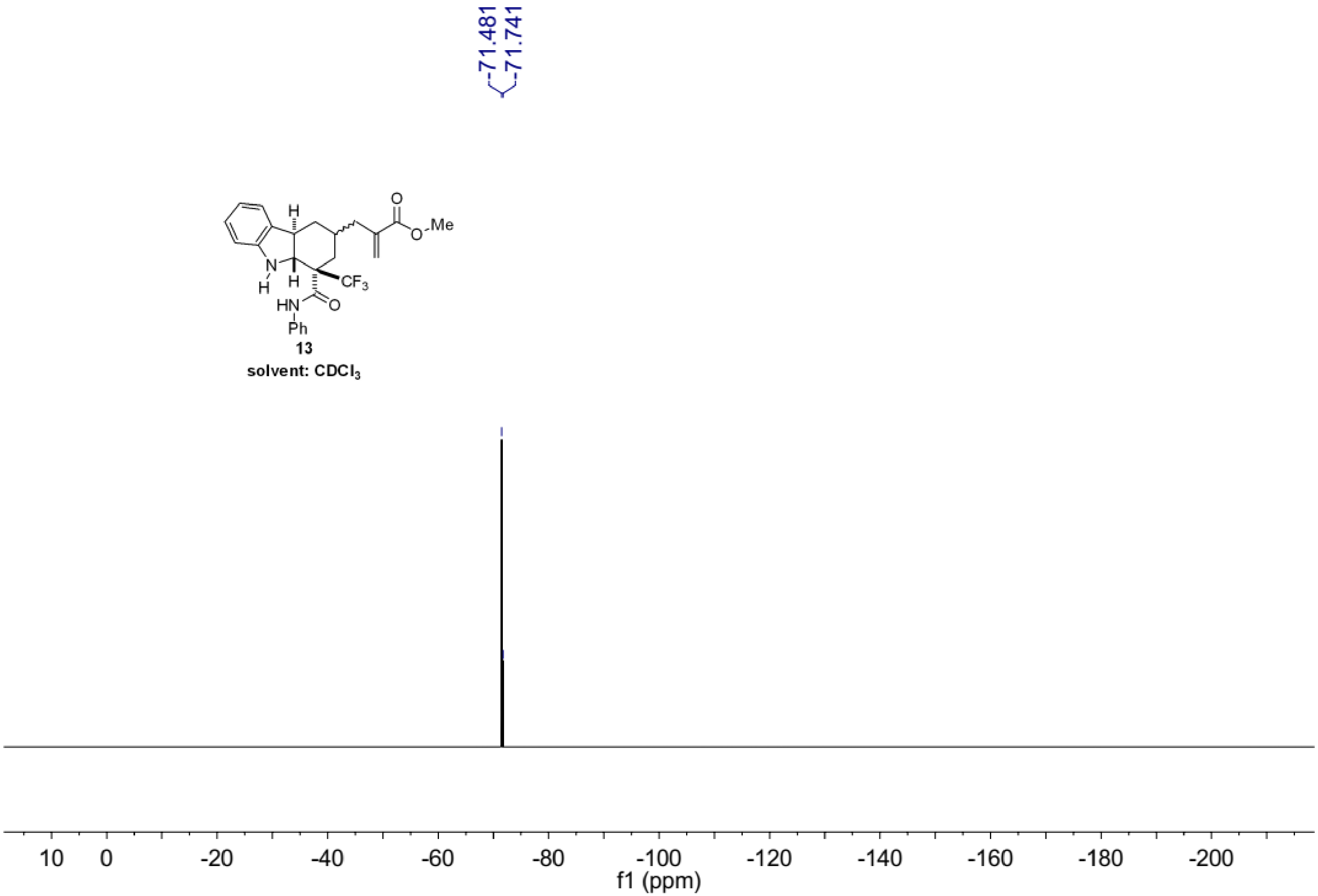

Supplementary Figure 294. ^{19}F NMR spectrum for compound **13**

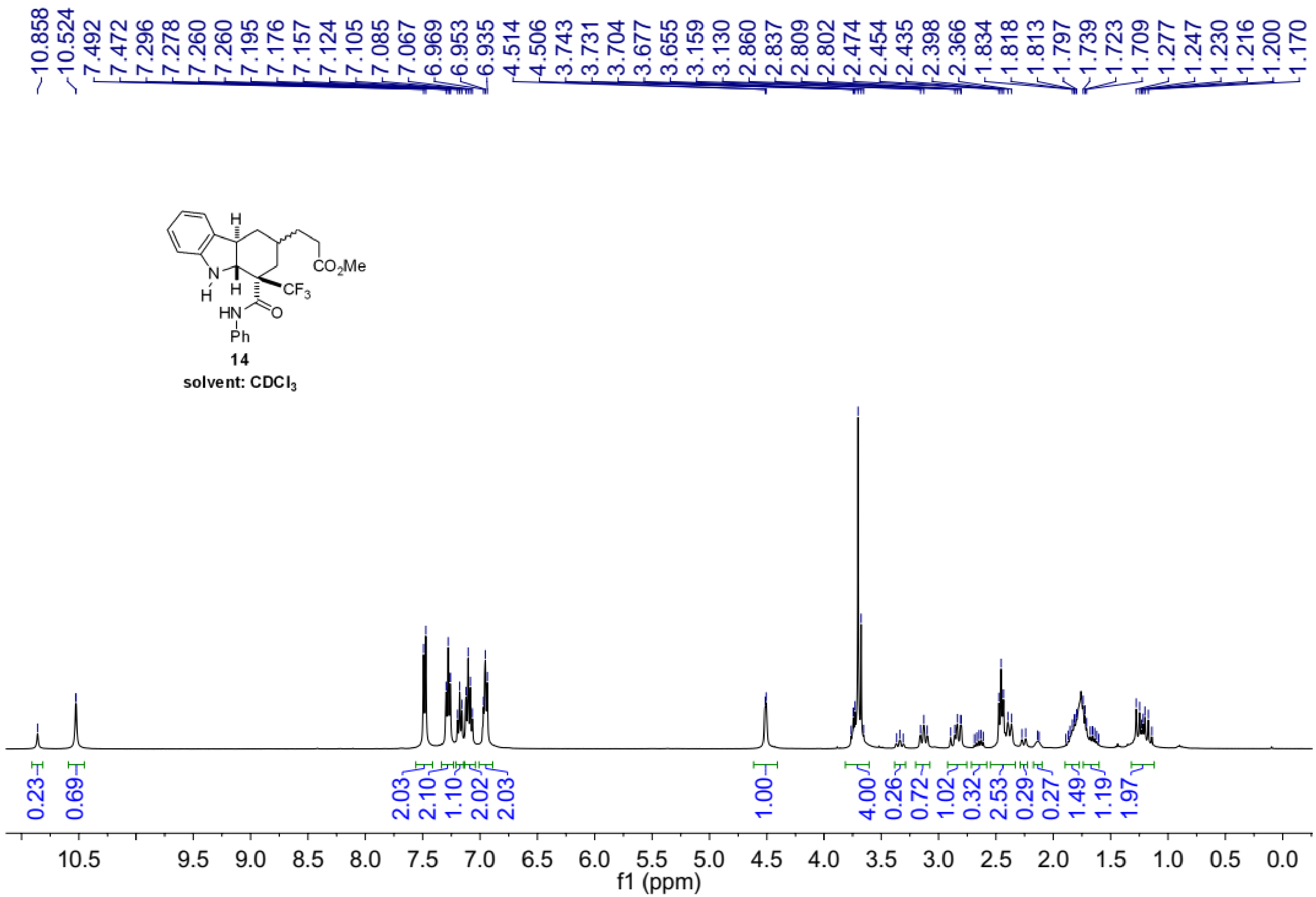

Supplementary Figure 295. ^{1}H NMR spectrum for compound **14**

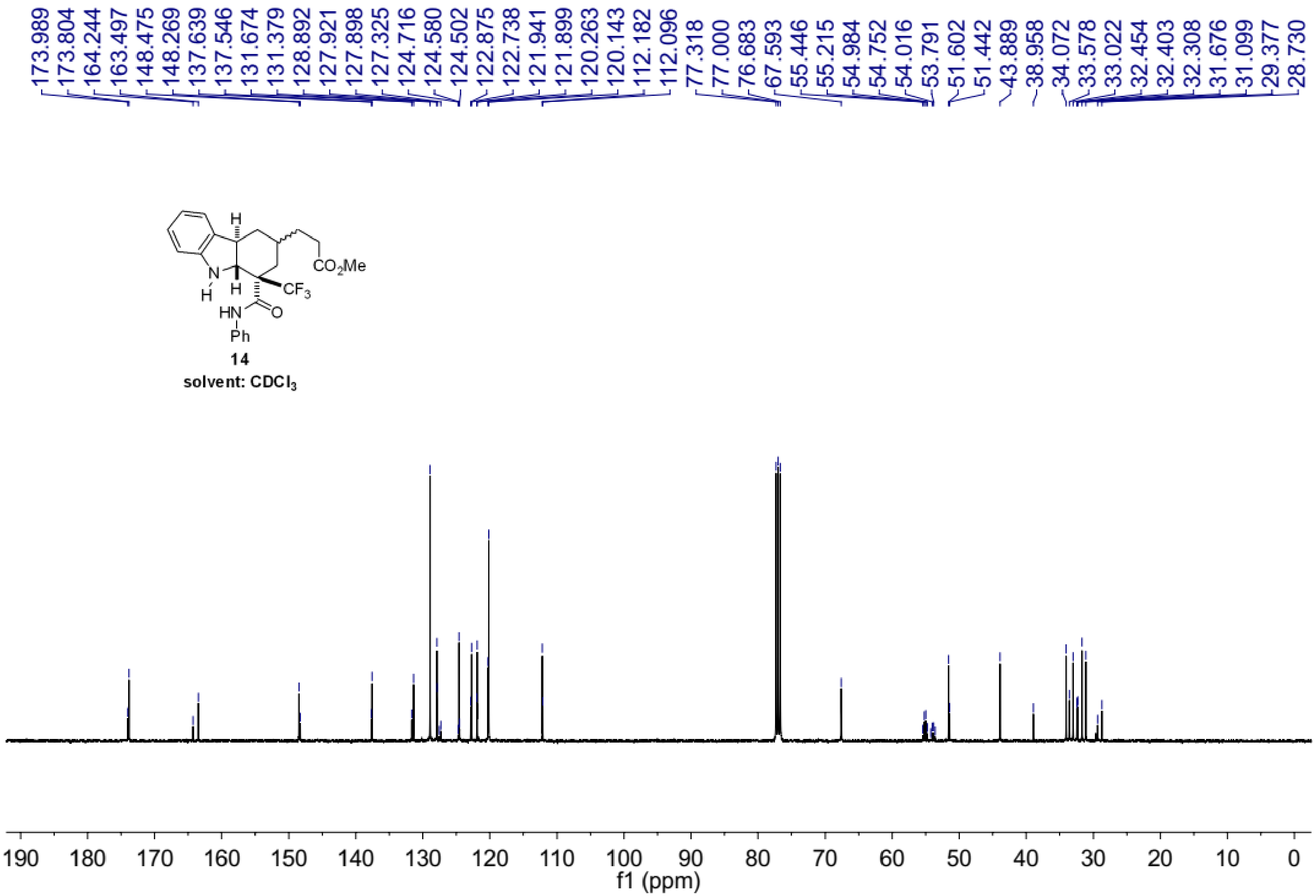

Supplementary Figure 296. ^{13}C NMR spectrum for compound **14**

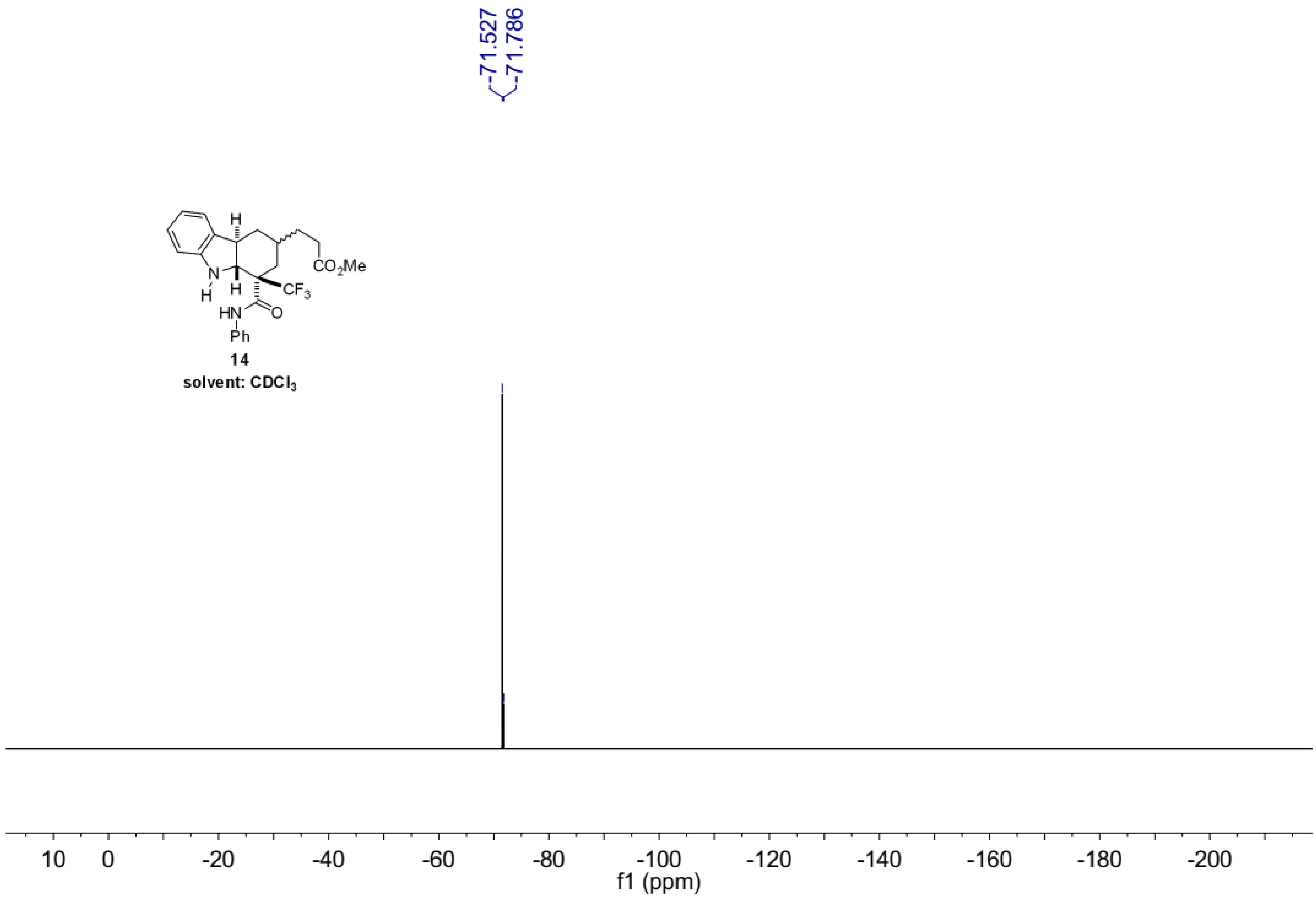

Supplementary Figure 297. ^{19}F NMR spectrum for compound **14**

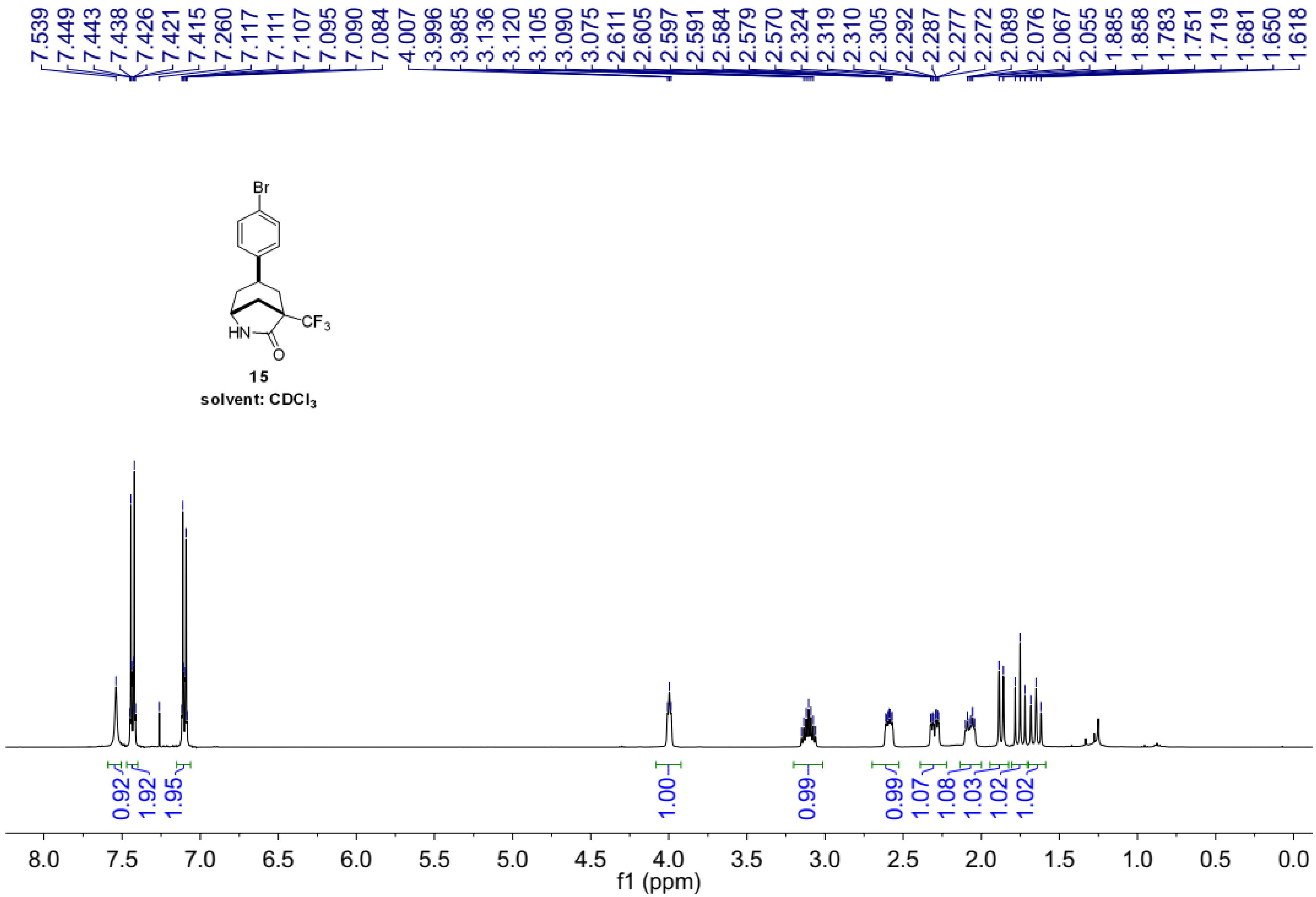

Supplementary Figure 298. ^{1}H NMR spectrum for compound **15**

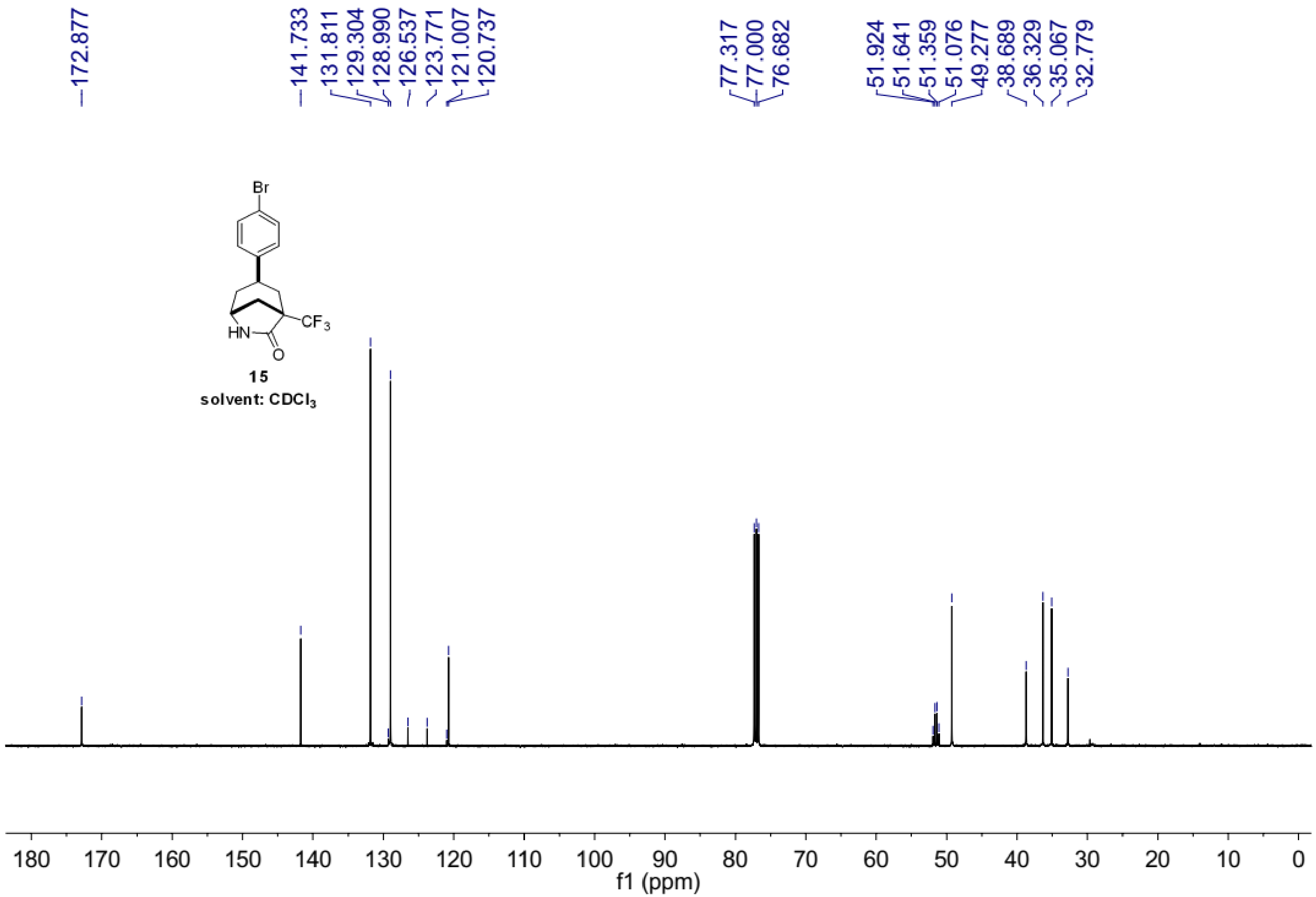

Supplementary Figure 299. ^{13}C NMR spectrum for compound **15**

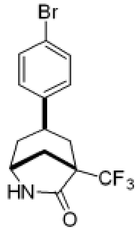

15
solvent: $CDCl_3$

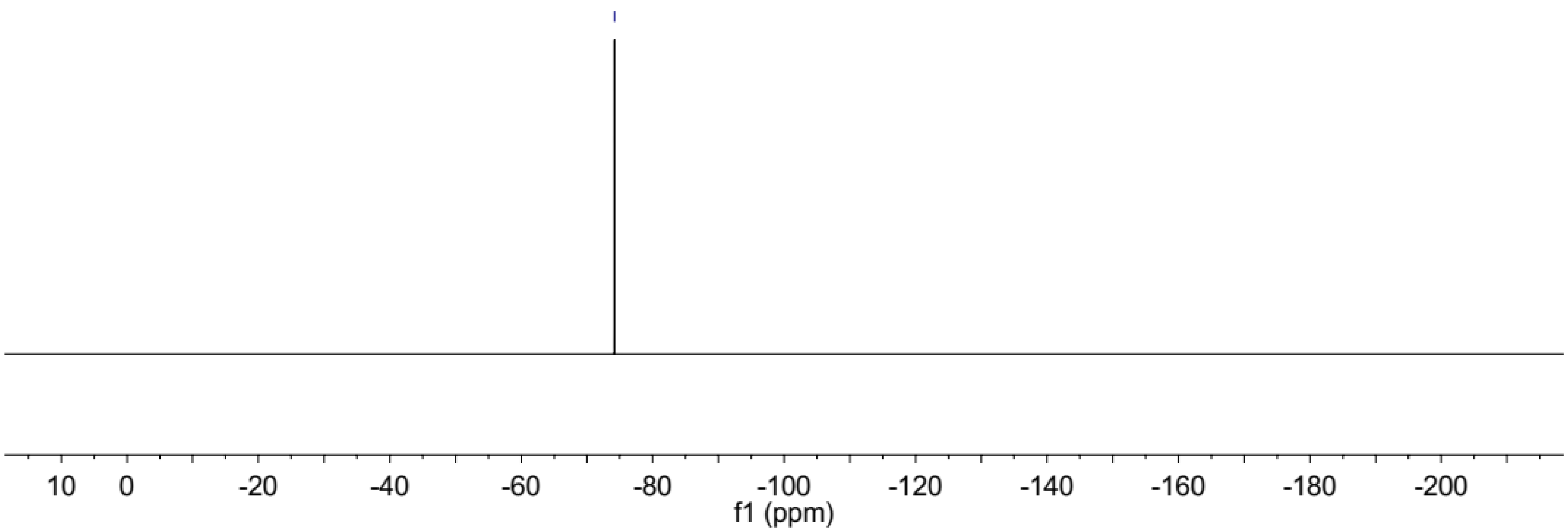

Supplementary Figure 300. ^{19}F NMR spectrum for compound **15**

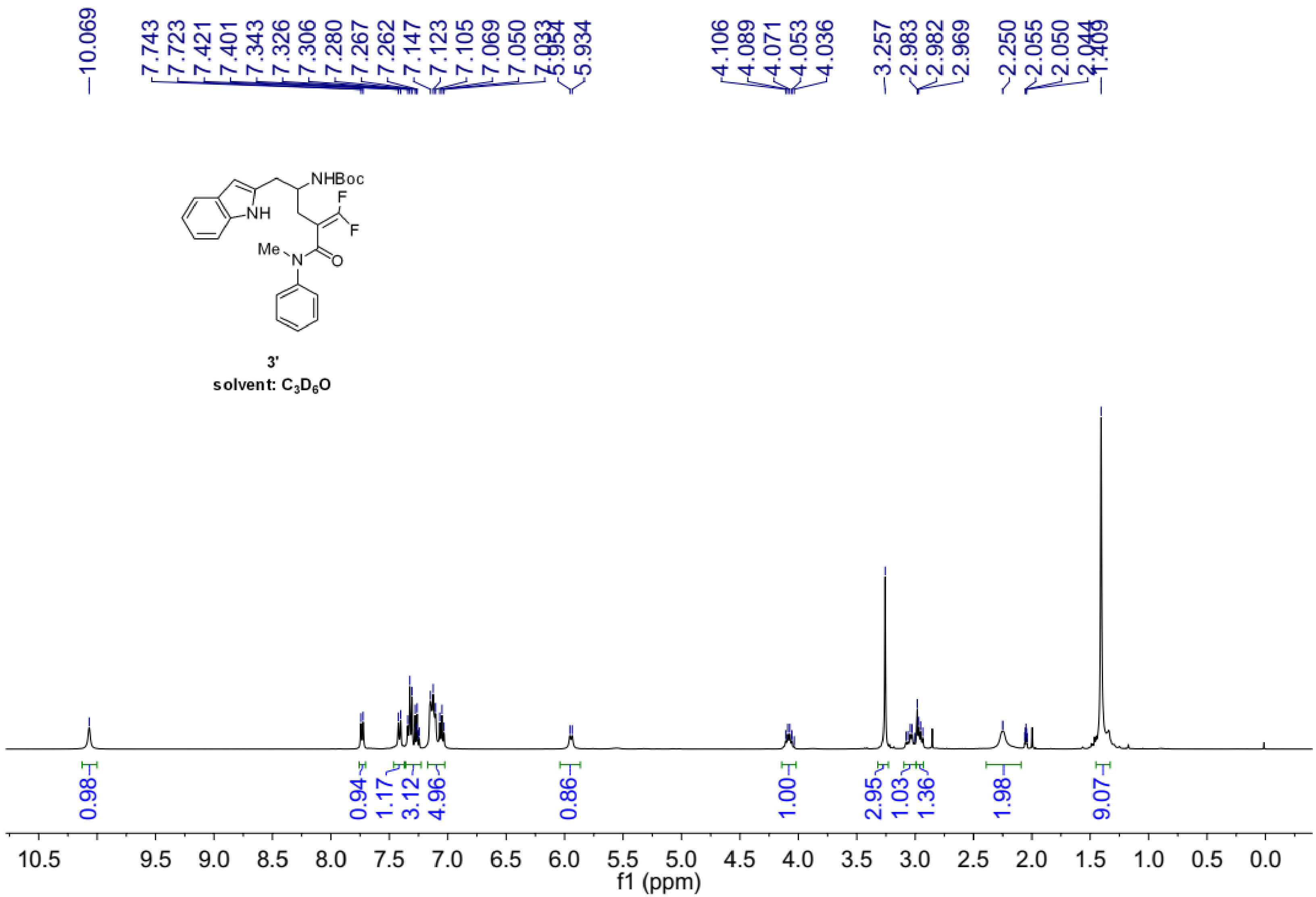

Supplementary Figure 301. ^{1}H NMR spectrum for compound **3’**

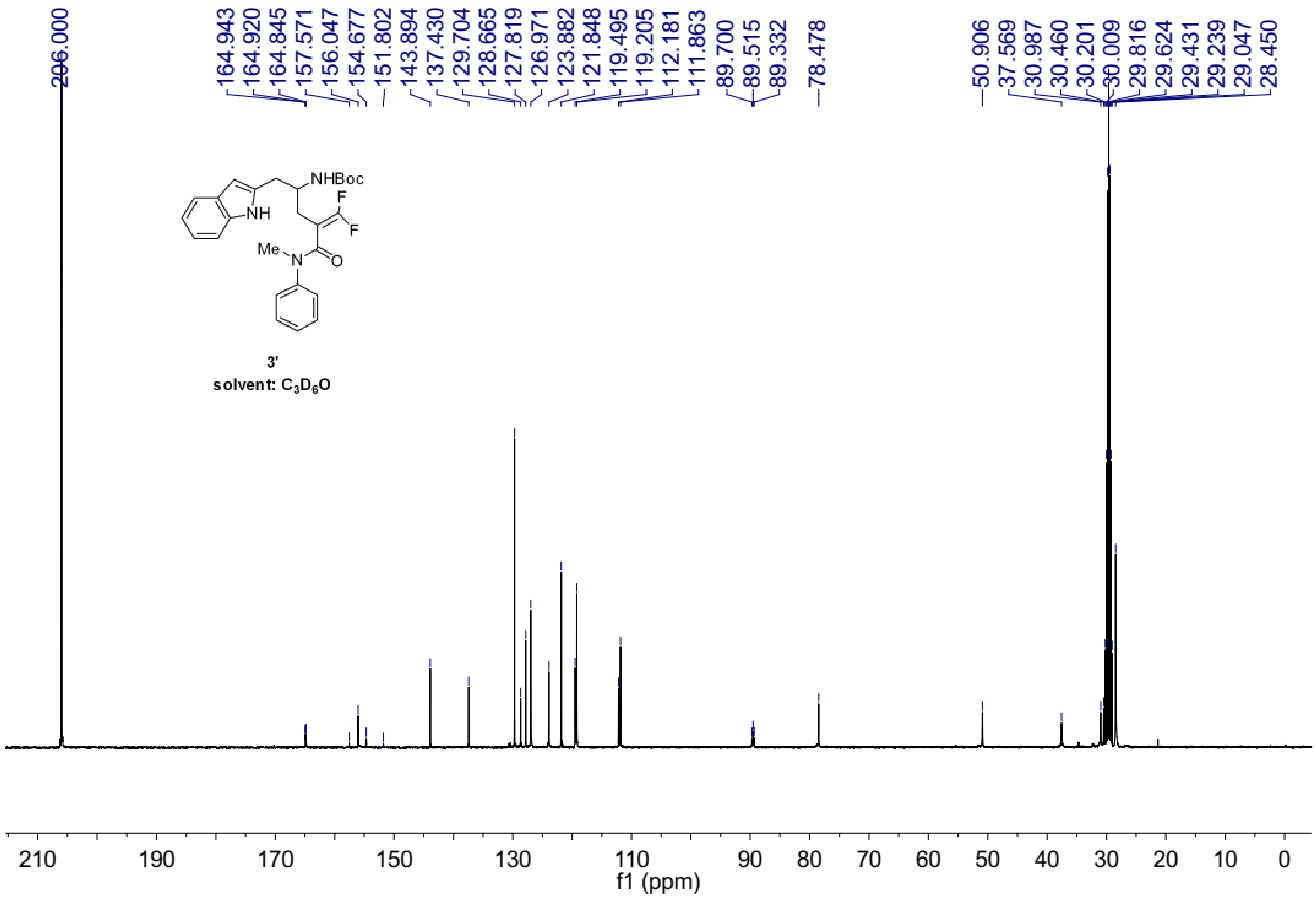

Supplementary Figure 302. ^{13}C NMR spectrum for compound **3’**

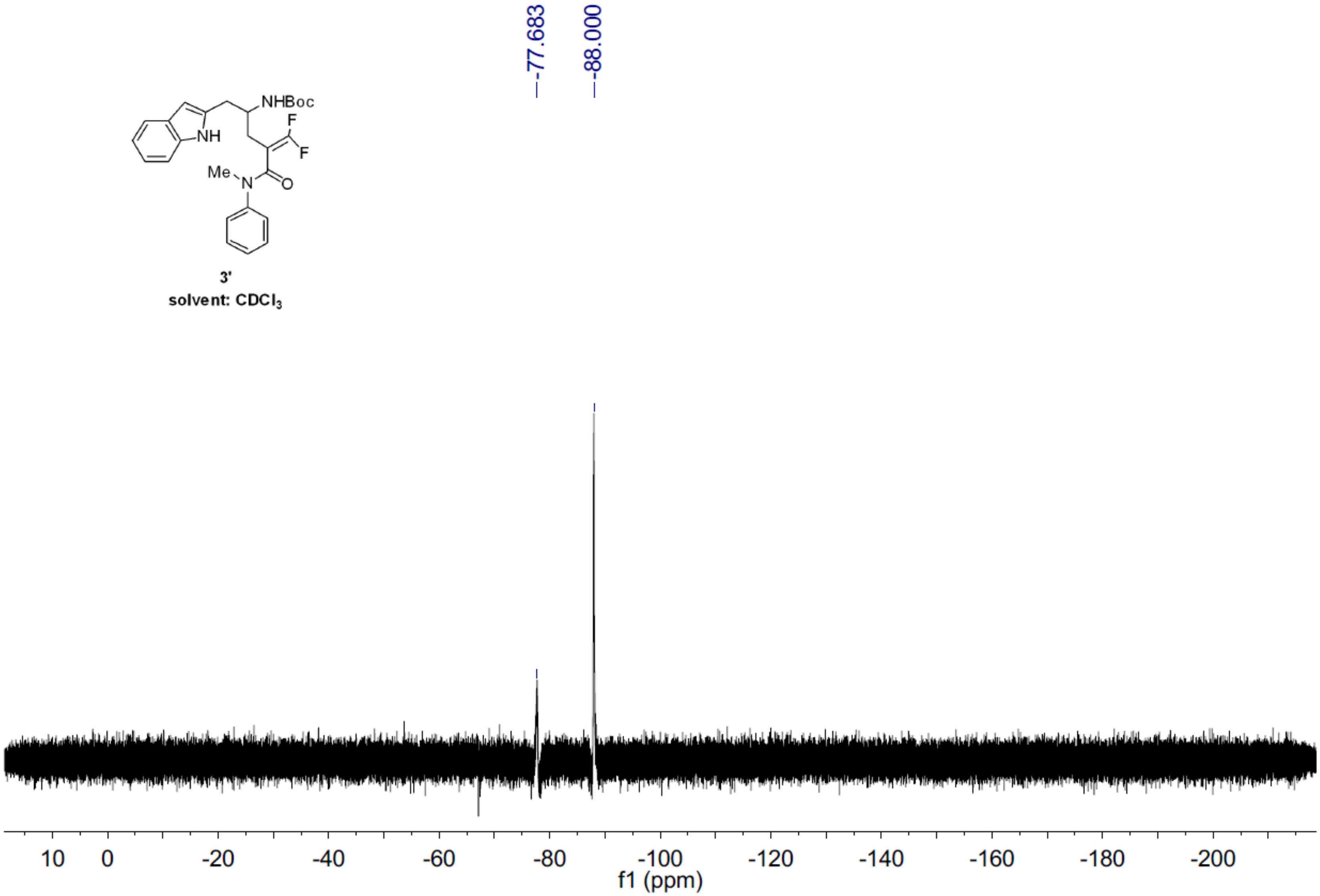

3'
solvent: $CDCl_3$

Supplementary Figure 303. ^{19}F NMR spectrum for compound **3'**

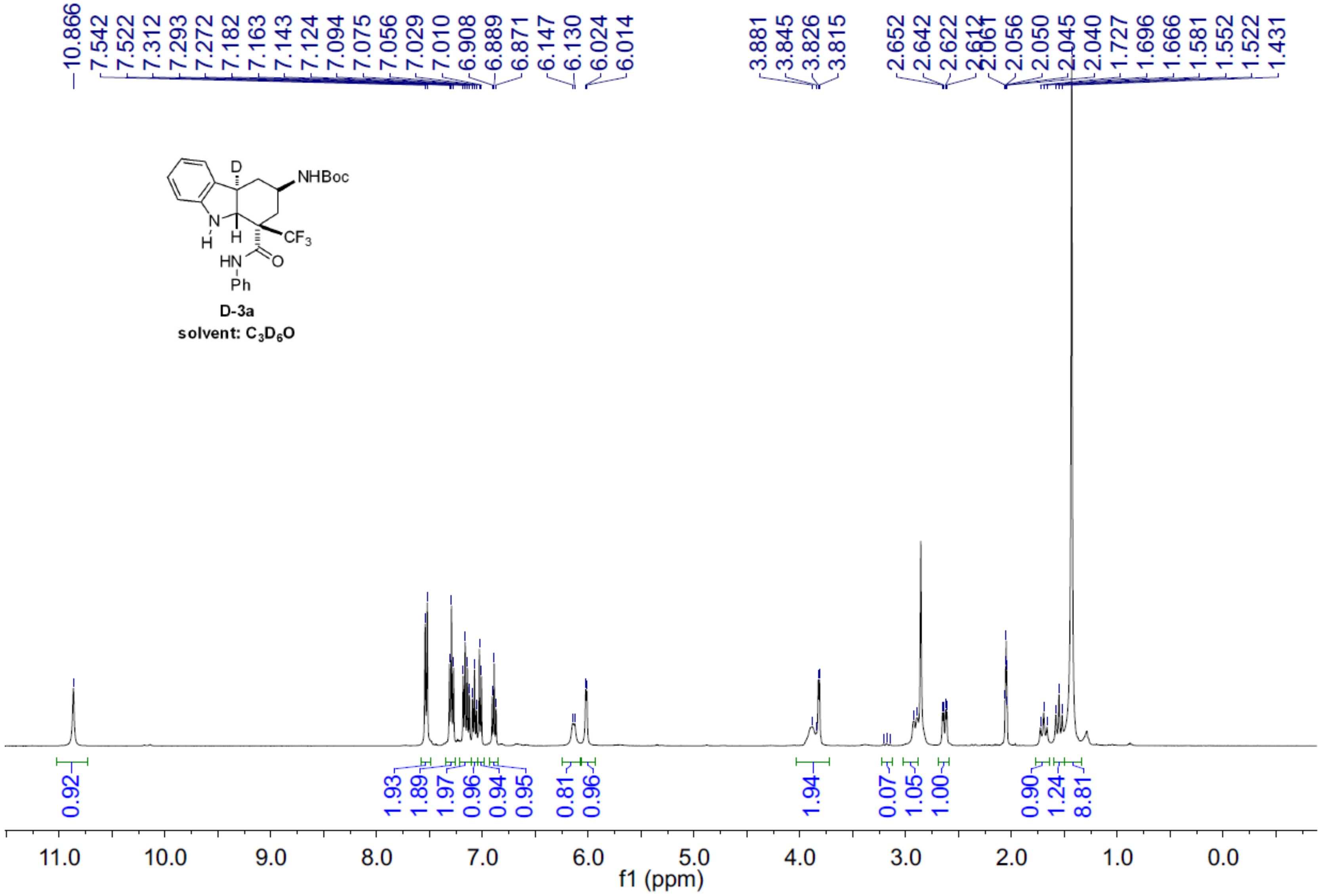

Supplementary Figure 304. ^{1}H NMR spectrum for compound D-**3a**

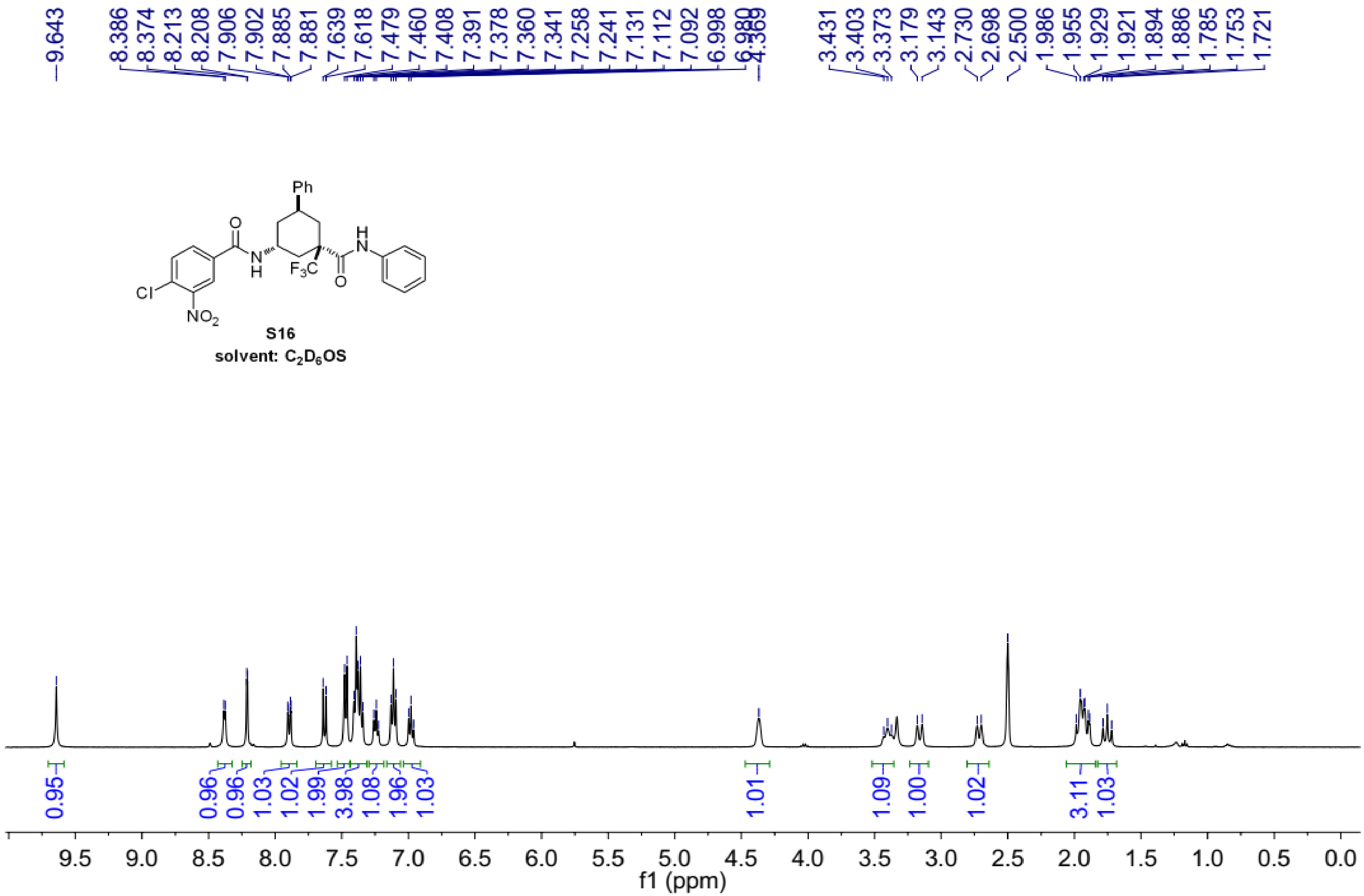

Supplementary Figure 305. ^{1}H NMR spectrum for compound **S16**

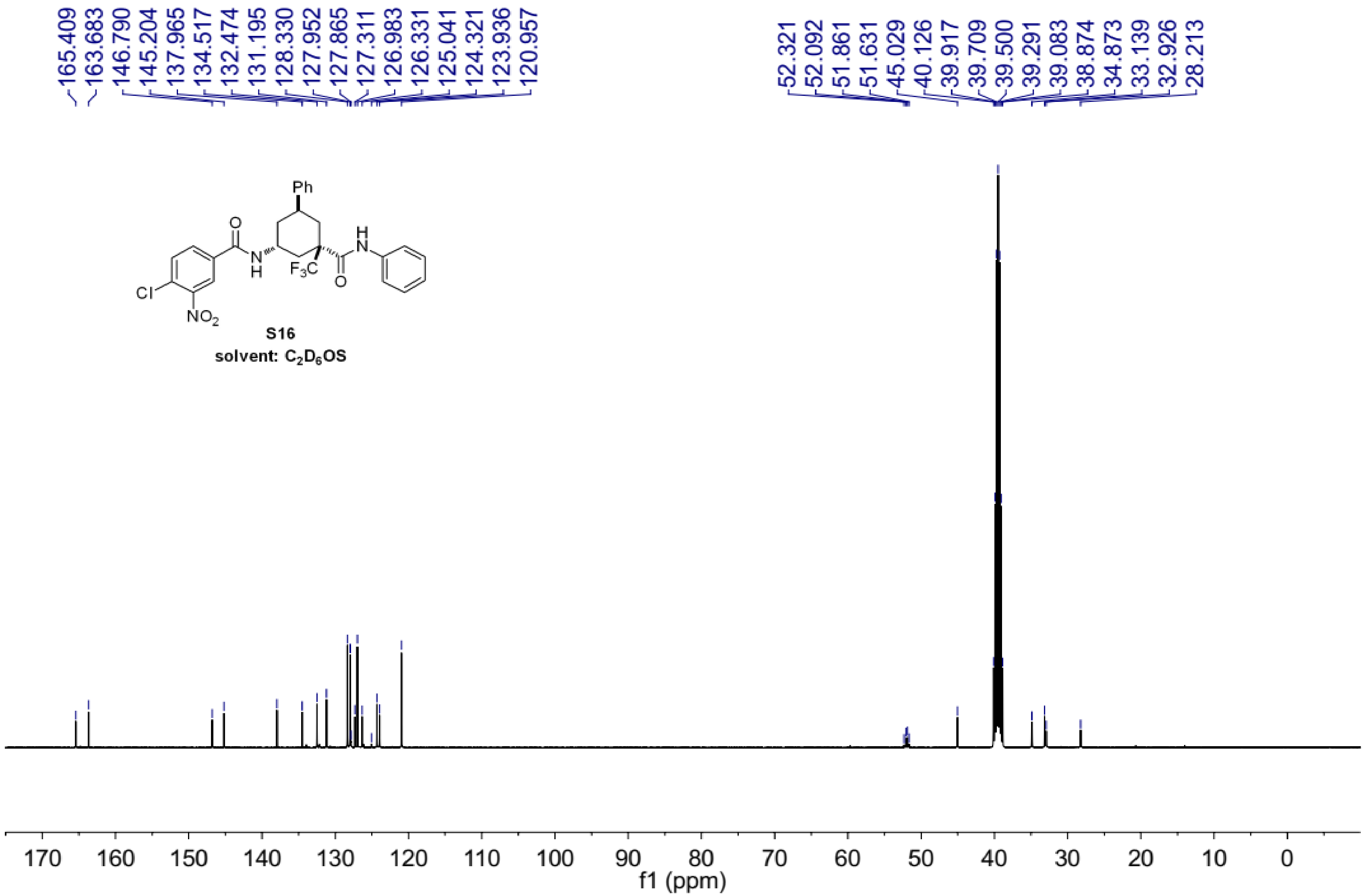

Supplementary Figure 306. ^{13}C NMR spectrum for compound **S16**

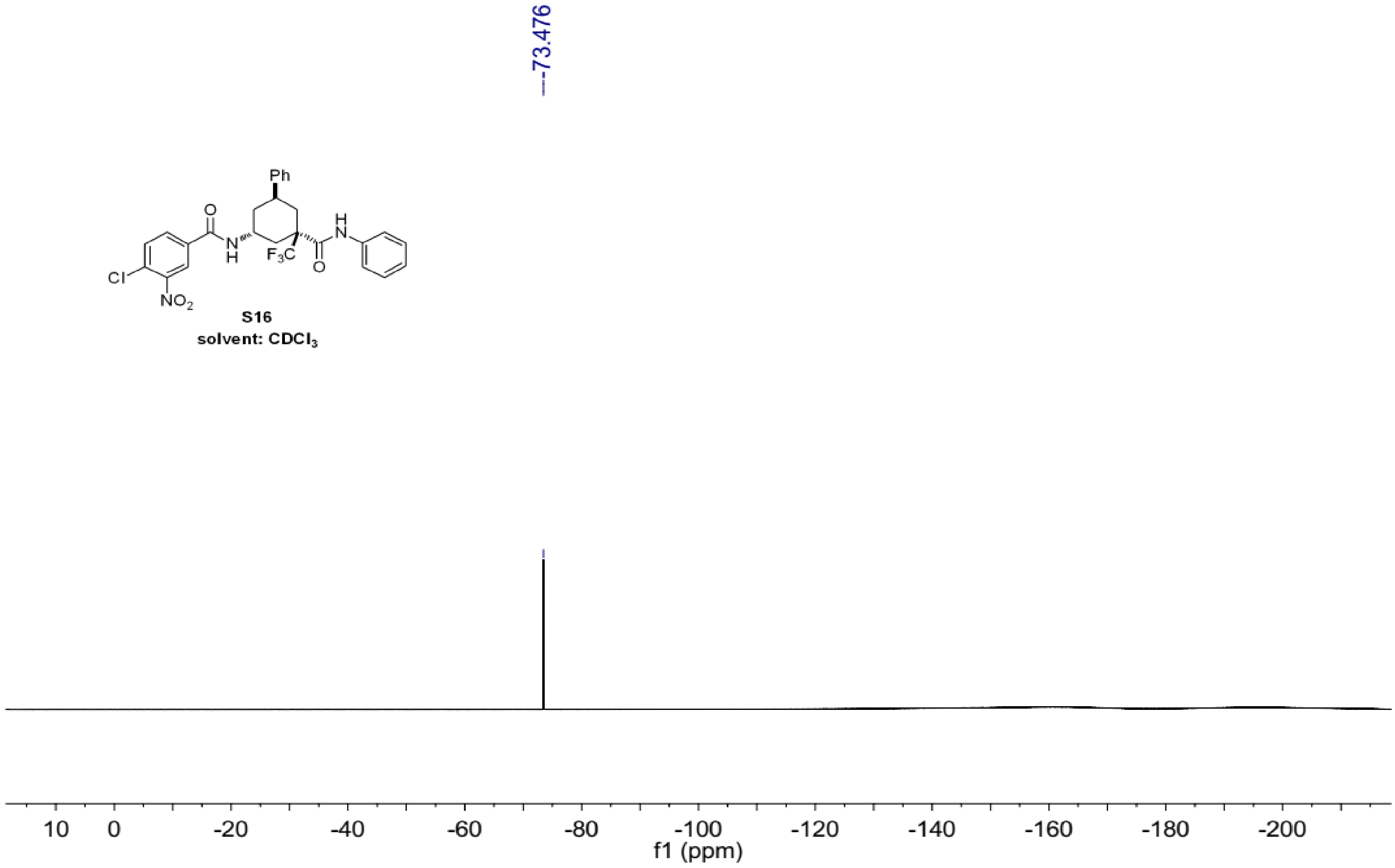

Supplementary Figure 307. ^{19}F NMR spectrum for compound **S16**

8. References.

1. Sommer, K. & Williams, R. M. Studies on Paraherquamide Biosynthesis: Synthesis of Deuterium-Labeled 7-Hydroxy-Pre-Paraherquamide, a Putative Precursor of Paraherquamides A, E, and F. *Tetrahedron* **65**, 3246–3260 (2009).
2. Shao, C., Shi, G., Zhang, Y., Pan, S. & Guan, X. Palladium-Catalyzed C–H Ethoxycarbonyldifluoromethylation of Electron-Rich Heteroarenes. *Org. Lett.* **17**, 2652–2655 (2015).
3. Roy, A. D., Goss, R. J. M., Wagner, G. K. & Winn, M. Development of Fluorescent Aryltryptophans by Pd Mediated Cross-Coupling of Unprotected Halotryptophans in Water. *Chem. Commun*. 4831–4833 (2008).
4. Li, J.-T., Sun, S.-F. & Sun, M.-X. Improved Synthesis of 3-(Dialkylaminomethyl)-Indole in Acetic Acid Aqueous Solution Under Ultrasound Irradiation. *Ultrasonics sonochemistry*. **18,** 42–44 (2011).
5. Jones, D. T., Artman, III G. D. & Williams, R. M. Coupling of Activated Esters to Gramines in the Presence of Ethyl Propiolate Under Mild Conditions. *Tetrahedron Lett*. **48**, 1291–1294 (2007).
6. Füller, J. J. et al. Biosynthesis of Violacein, Structure and Function of l-Tryptophan Oxidase VioA from Chromobacterium Violaceum. *J. Biol. Chem.* **291**, 20068–20084 (2016).
7. Zhang, L. & John, M. F. A Facile Method for the Asymmetric Synthesis of .alpha.-Methyltryptophan. *J. Org. Chem*. **60**, 5719–5720 (1995).
8. Ihara, H. & Kumamoto, K. *PCT Int. Appl.* **WO2008126665**, 23 Oct 2008.
9. Zheng, B.-H., Ding, C.-H., Hou, X.-L. & Dai, L.-X. Ag-Catalyzed Diastereo- and Enantioselective Synthesis of β-Substituted Tryptophans from Sulfonylindoles. *Org. Lett*. **12**, 1688−1691 (2010).
10. Akiyama, T., Takaya, J. & Kagoshima, H. Brønsted Acid-Catalyzed Mannich-Type Reactions in Aqueous Media. *Adv. Synth. Catal*. **344**, 338–347 (2002).
11. Li, J., Kong, M., Qiao, B., Lee, R., Zhao, X. & Jiang, Z. Formal Enantioconvergent Substitution of Alkyl Halides via Catalytic Asymmetric Photoredox Radical Coupling. *Nat. Commun*. **9**, 2445 (2018). DOI: 10.1038/s41467-018-04885-3.
12. Chen, L.-Y., Chen, J.-R., Cheng, H.-G., Lu, L.-Q. & Xiao, W.-J. Enantioselective Synthesis of Tetrahydrofuran Derivatives by Sequential Henry Reaction and Iodocyclization of γ,δ-Unsaturated Alcohols. *Eur. J. Org. Chem*. 4714–4719 (2014).
13. Litman, Z. C., Sharma, A. & Hartwig, J. F. Oxidation of Hindered Allylic C–H Bonds with Applications to the Functionalization of Complex Molecules. *ACS. Catal*. **7**, 1998−2001 (2017).
14. Wu, J., He, L., Noble, A. & Aggarwal, V. K. Photoinduced Deaminative Borylation of Alkylamines. *J. Am. Chem. Soc.* **140**, 10700−10704 (2018).
15. Wu, J., Grant, P. S., Li, X., Noble, A. & Aggarwal, V. K. Catalyst-Free Deaminative Functionalizations of Primary Amines by Photoinduced Single-Electron Transfer. *Angew. Chem. Int. Ed.* **58**, 5697−5701 (2019).
